# Supplementary material for: Thioester-mediated RNA aminoacylation and peptidyl-RNA synthesis in water
Source: Nature. 2025 Aug 27;644(8078):933–44. doi: 10.1038/s41586-025-09388-y (PMC12390845; doi:10.1038/s41586-025-09388-y)
Supplement: Supplementary file 1 — This Supplementary Information file (PDF) includes Supplementary Discussion, General Experimental Procedures A–M, Supplementary Figs. 1–264 (nuclear magnetic resonance (NMR) spectra, gel images, reaction time course graphs), Supplementary Tables 1–51 (reaction scope, optimization, mass spectrometry (MS) data) and Supplementary References. [file 41586_2025_9388_MOESM1_ESM.pdf]

nature portfolio

https://doi.org/10.1038/s41586-025-09388-y

Supplementary information

Thioester-mediated RNA aminoacylation and peptidyl-RNA synthesis in water

In the format provided by the authors and unedited

Supplementary Information for

Thioester-mediated RNA-aminoacylation and peptidyl-RNA synthesis in water

Jyoti Singh, Benjamin Thoma, Daniel Whitaker, Max Satterly Webley, Yuan Yao and Matthew W. Powner*

Department of Chemistry, University College London, 20 Gordon Street, London, WC1H 0AJ, UK

*Correspondence to: matthew.powner@ucl.ac.uk

Table of Contents

MATERIALS AND METHODS 7

RESULTS AND DATA 9

Reaction of thioester 1^{e}_{Aaa} with nucleophiles 9
General Procedure A – reactivity of **L-1**$_{Ala}$ with potential nucleophiles 9
α-Aminoacyl-thioester dimerisation at specified pHs 9
Reaction of α-aminoacyl thiol with nucleophiles 12
Reaction of L-alanine thioester L-**1**$_{Ala}$ with glycerol **19** 13
Reaction of L-alanine thioester L-**1**$_{Ala}$ with L-alanine L-**7**$_{Ala}$ 14
Reaction of L-alanine thioester L-**1**$_{Ala}$ with L-alanine amide L-**10**$_{Ala}$ 15
Reaction of L-alanine thioester L-**1**$_{Ala}$ with glycine nitrile **8**$_{Gly}$ 16
Reaction of L-alanine thioester L-**1**$_{Ala}$ with leucine nitrile **8**$_{Leu}$ 18
Reaction of L-alanine thioester L-**1**$_{Ala}$ with L-alanylglycine **13**$_{AlaGly}$ 19
Reaction of L-alanine thioester L-**1**$_{Ala}$ with L, L, L-alanylalanylalanine **Ala$_3$** 20
Reaction of L-alanine thioester L-**1**$_{Ala}$ with L-cysteine L-**7**$_{Cys}$ 21
Competition reaction of serine **7**$_{Ser}$ with alanine thioester L-**1**$_{Ala}$ 22
Reaction of L-alanine thioester L-**1**$_{Ala}$ with 3-mercaptopropanol **5f** 23
Reaction of L-alanine thioester L-**1**$_{Ala}$ with 2-mercaptoethanol **5g** 25
Synthesis of α-aminoacyl ester **14**$_{Ala}$ from alanine nitrile **8**$_{Ala}$ and 3-mercaptopropanol **5f** at different pHs 27
Synthesis of α-aminoacyl ester **14**$_{Ala}$ from the reaction of alanine nitrile **8**$_{Ala}$ with 3-mercaptopropanol **5f** 28
Synthesis of α-aminoacyl ester **14**$_{Ser}$ from the reaction of serine nitrile **8**$_{Ser}$ with 3-mercaptopropanol **5f** 30
Synthesis of α-aminoacyl ester **14**$_{Pro}$ from the reaction of proline nitrile **8**$_{Pro}$ with 3-mercaptopropanol **5f** 32

Synthesis of α-amino thioacids 12$_{Aaa}$ from α-amino thioesters 1$_{Aaa}$ 34
General Procedure B: Synthesis of α-amino thioacids **12**$_{Aaa}$ from α-amino thioesters **1**$_{Aaa}$ 34
Synthesis of glycine thioacid **12**$_{Gly}$ from glycine thioester **1**$_{Gly}$ 35
Synthesis of alanine thioacid **12**$_{Ala}$ from L-alanine thioester **1**$_{Ala}$ 36
Synthesis of leucine thioacid **12**$_{Leu}$ from L-leucine thioester **1**$_{Leu}$ 37
Synthesis of arginine thioacid **12**$_{Arg}$ from arginine thioester **1**$_{Arg}$ 39
Synthesis of proline thioacid **12**$_{Pro}$ from L-proline thioester **1**$_{Pro}$ 41
Synthesis of lysine thioacid **12**$_{Lys}$ from L-lysine thioester **1**$_{Lys}$ 43
Synthesis of valine thioacid **12**$_{Val}$ from L-valine thioester **1**$_{Val}$ 45
Synthesis of phenylalanine thioacid **12**$_{Phe}$ from L-phenylalanine thioester **1**$_{Phe}$ 47
Synthesis of serine thioacid **12**$_{Ser}$ from serine thioester **1**$_{Ser}$ 49
Thiol exchange to form **1**$^{n}_{Ala}$ from alanine thioester **1**$^{e}_{Ala}$ at pH 6.5 50

Thioester stability at different pH 51

Determination of Thioester L-1Aaae pK$_{aH}$ 53

Thioester-mediated aminoacylation of nucleoside and nucleotide monomers 54
General procedure C – Formation of aminoacyl nucleosides **17** by the reaction of aminoacyl thiols **1** and nucleosides or nucleotides 54
Chemo- and Regioselectivity for aminoacylation of nucleosides upon reaction with thioester **1**$^{e}_{Ala}$ 54
Aminoacylation of nucleoside **16C** with aminoacyl thiol **1**$_{Ala}$ at pH 6.5 56
Aminoacylation of nucleoside **16G** with aminoacyl thiol **1**$_{Ala}$ at pH 6.5 58
Aminoacylation of nucleoside **16A** with aminoacyl thiol **1**$_{Ala}$ at pH 6.5 59
Aminoacylation of nucleoside **21A** with aminoacyl thiol **1**$_{Ala}$ at pH 6.5 61
Aminoacylation of nucleoside **22A** with aminoacyl thiol **1**$_{Ala}$ at pH 6.5 62

Aminoacylation of nucleoside **23U** with aminoacyl thiol $\mathbf{1}_{\mathbf{Ala}}$ at pH 6.5 63
Aminoacylation of nucleoside **20A** with aminoacyl thiol $\mathbf{1}_{\mathbf{Ala}}$ at pH 6.5 64
Aminoacylation of nucleoside **24A** with aminoacyl thiol $\mathbf{1}_{\mathbf{Ala}}$ at pH 6.5 65
Aminoacylation of nucleoside **25A** with aminoacyl thiol $\mathbf{1}_{\mathbf{Ala}}$ at pH 6.5 66
Aminoacylation of nucleoside **26U** with aminoacyl thiol $\mathbf{1}_{\mathbf{Ala}}$ at pH 6.5 67
Aminoacylation of nucleoside **26G** with aminoacyl thiol $\mathbf{1}_{\mathbf{Ala}}$ at pH 6.5 68
Aminoacylation of nucleoside **26C** with aminoacyl thiol $\mathbf{1}_{\mathbf{Ala}}$ at pH 6.5 69
Aminoacylation of nucleoside **26A** with aminoacyl thiol $\mathbf{1}_{\mathbf{Ala}}$ at pH 6.5 70
Aminoacylation of nucleoside **27A** with aminoacyl thiol $\mathbf{1}_{\mathbf{Ala}}$ at pH 6.5 71
Aminoacylation of nucleoside **28A** with aminoacyl thiol $\mathbf{1}_{\mathbf{Ala}}$ at pH 6.5 72
Aminoacylation of nucleoside **29A** with aminoacyl thiol $\mathbf{1}_{\mathbf{Ala}}$ at pH 6.5 73
Aminoacylation of nucleoside **30A** with aminoacyl thiol $\mathbf{1}_{\mathbf{Ala}}$ at pH 6.5 74
Aminoacylation of nucleoside **31A** with aminoacyl thiol $\mathbf{5}_{\mathbf{Ala}}$ at pH 6.5 75
Aminoacylation of nucleoside **32A** with aminoacyl thiol $\mathbf{1}_{\mathbf{Ala}}$ at pH 6.5 76
Aminoacylation of nucleoside **49U** with aminoacyl thiol $\mathbf{1}_{\mathbf{Ala}}$ at pH 6.5 77
Aminoacylation of nucleoside **15A** with aminoacyl thiol $\mathbf{1}_{\mathbf{Ala}}$ at pH 6.5 78
Aminoacylation of nucleosides **16C** and **15A** with aminoacyl thiol $\mathbf{1}_{\mathbf{Ala}}$ at pH 6.5 80
Aminoacylation of uridine (**16U**) with aminoacyl thiol $\mathbf{1}_{\mathbf{Ala}}$ at different pHs 81
Diketopiperazine $\mathbf{6}_{\mathbf{AlaAla}}$ formation at different pHs 85
Aminoacylation of uridine (**16U**) with thioester $\mathbf{1}_{\mathbf{Ala}}$ at different nucleoside concentrations at pH 6.5 86
Aminoacylation of uridine (**16U**) with different concentrations of thioester $\mathbf{1}_{\mathbf{Ala}}$ 87
Side chain compatibility for aminoacylation of uridine (**16U**) by thioesters $\mathbf{1}_{\mathbf{Aaa}}$ 89
Synthesis of aminoacyl-uridine $\mathbf{17^U}_{\mathbf{Ala}}$ upon incubating uridine (**16U**) with thioester $\mathbf{1}_{\mathbf{Ala}}$ 91
Synthesis of aminoacyl-uridine $\mathbf{17^U}_{\mathbf{Gly}}$ upon incubating uridine (**16U**) with thioester $\mathbf{1}_{\mathbf{Gly}}$ 92
Synthesis of aminoacyl-uridine $\mathbf{17^U}_{\mathbf{Leu}}$ upon incubating uridine (**16U**) with thioester $\mathbf{1}_{\mathbf{Leu}}$ 94
Synthesis of aminoacyl-uridine $\mathbf{17^U}_{\mathbf{Pro}}$ upon incubating uridine (**16U**) with thioester $\mathbf{1}_{\mathbf{Pro}}$ 95
Synthesis of aminoacyl-uridine $\mathbf{17^U}_{\mathbf{Glu}}$ upon incubating uridine (**16U**) with thioester $\mathbf{1}_{\mathbf{Glu}}$ 96
Synthesis of aminoacyl-uridine $\mathbf{17^U}_{\mathbf{Gln}}$ upon incubating uridine (**16U**) with thioester $\mathbf{1}_{\mathbf{Gln}}$ 97
Synthesis of aminoacyl-uridine $\mathbf{17^U}_{\mathbf{Met}}$ upon incubating uridine (**16U**) with thioester $\mathbf{1}_{\mathbf{Met}}$ 98
Synthesis of aminoacyl-uridine $\mathbf{17^U}_{\mathbf{Arg}}$ upon incubating uridine (**16U**) with thioester $\mathbf{1}_{\mathbf{Arg}}$ 100
Synthesis of aminoacyl-uridine $\mathbf{17^U}_{\mathbf{Lys}}$ upon incubating uridine (**16U**) with thioester $\mathbf{1}_{\mathbf{Lys}}$ 102
Synthesis of aminoacyl-uridine $\mathbf{17^U}_{\mathbf{His}}$ upon incubating uridine (**16U**) with thioester $\mathbf{1}_{\mathbf{His}}$ 104
Synthesis of aminoacyl-uridine $\mathbf{17^U}_{\mathbf{Val}}$ upon incubating uridine (**16U**) with thioester $\mathbf{1}_{\mathbf{Val}}$ 105
Synthesis of aminoacyl-uridine $\mathbf{17^U}_{\mathbf{Phe}}$ upon incubating uridine (**16U**) with thioester $\mathbf{1}_{\mathbf{Phe}}$ 106
Synthesis of aminoacyl-uridine $\mathbf{17^U}_{\mathbf{Ser}}$ upon incubating uridine (**16U**) with thioester $\mathbf{1}_{\mathbf{Ser}}$ 107
Synthesis of aminoacyl-uridine $\mathbf{17^U}_{\mathbf{Pip}}$ upon incubating uridine (**16U**) with thioester $\mathbf{1}_{\mathbf{Pip}}$ 108
Synthesis of aminoacyl-uridine $\mathbf{17^U}_{\mathbf{Ala}}$ under eutectic conditions upon incubating uridine (**16U**) with thioester $\mathbf{1}_{\mathbf{Ala}}$ 109
Synthesis of aminoacyl-adenosine $\mathbf{17^A}_{\mathbf{Arg}}$ upon incubating adenosine (**16A**) with thioester $\mathbf{1}_{\mathbf{Arg}}$ 111
Synthesis of cyclic guanidine **33** 112
Synthesis of aminoacyl-adenosine $\mathbf{17^A}_{\mathbf{Arg}}$ upon incubating adenosine (**16A**) with cyclic arginine **33** 114
Synthesis of aminoacyl-adenosine 17^A_{Arg} upon incubating adenosine 16A with varying amounts of cyclic arginine 33 115
Coupling of cyclic arginine **33** with 2-mercaptoethanesulfonate **5c** at pH 6.5 and room temperature 117
Aminoacylation of uridine (**16U**) upon reaction with different thioester $\mathbf{1^n}_{\mathbf{Ala}}$ 118
Competition reaction of uridine (**16A**) with thioester $\mathbf{1}_{\mathbf{Ala}}$ and amido thioester $\mathbf{39}_{\mathbf{Ala}}$ 119

Aminoacylation of RNA oligomers 120
Purification of RNA oligomers 120
Polyacrylamide gel electrophoresis (PAGE) procedure 120
General procedure D: Analysis of non-template-directed aminoacylation of oligonucleotides 120
General procedure E: Analysis of template-directed aminoacylation of oligonucleotides 120
Sequence nomenclature 121
General Procedure F: Non-template-directed aminoacylation of oligonucleotides by aminoacyl thiols **1** 122
General Procedure G: Template-directed aminoacylation of oligonucleotides by aminoacyl thiols **1** 122
General Procedure H: Aminoacylation in a nicked duplex construct with aminoacyl thiols **1** 122
Diastereoselective aminoacylation of nucleosides with thioesters L-$\mathbf{1}_{\mathbf{Ala}}$ and/or D-$\mathbf{1}_{\mathbf{Ala}}$ 132

Ribozyme-assisted aminoacylation 138
Aminoacylation with other electrophiles 140
MALDI-MS oligomer aminoacylation data 143

Formation of thioesters 1 from *N*-carboxyanhydrides 4 .... **144**
General procedure I for the formation of thioesters **1** in the reaction of thiols **5** with N-carboxyanhydrides $\mathbf{4_{aa}}$ 144
Formation of thioester $\mathbf{1_{Ala}}$ upon incubation thiols **5** with $\mathbf{4_{Ala}}$ at pH 5.0 144
Formation of thioester $\mathbf{1^{c}_{Aaa}}$ from $\mathbf{4_{Ala}}$ with 2-mercaptoethane sulfonate (**5c**) at different pHs and room temperature 146
Formation of thioester $\mathbf{1^{c}_{Aaa}}$ from $\mathbf{4_{Leu}}$ with 2-mercaptoethane sulfonate (**5c**) at different concentrations and room temperature 147
Thioester $\mathbf{1^{c}_{Aaa}}$ formation from the reaction of NCA $\mathbf{4_{Aaa}}$ with 2-mercaptoethanesulfonate **5c** 148
Formation of aminoacyl thiol $\mathbf{1_{Gly}}$ from the reaction of NCA $\mathbf{4_{Gly}}$ with 2-mercaptoethanesulfonate **5c** at pH 5 . 149
Formation of aminoacyl thiol $\mathbf{1_{Ala}}$ from the reaction of NCA $\mathbf{4_{Ala}}$ with 2-mercaptoethanesulfonate **5c** at pH 5.. 150
Formation of aminoacyl thiol $\mathbf{1_{Val}}$ from the reaction of NCA $\mathbf{4_{Val}}$ with 2-mercaptoethanesulfonate **5c** at pH 5.. 151
Formation of aminoacyl thiol $\mathbf{1_{Leu}}$ from the reaction of NCA $\mathbf{4_{Leu}}$ with 2-mercaptoethanesulfonate **5c** at pH 5 152
Formation of aminoacyl thiol $\mathbf{1_{Ser}}$ from the reaction of NCA $\mathbf{4_{ser}}$ with 2-mercaptoethanesulfonate **5c** at pH 5 .. 153
Formation of aminoacyl thiol $\mathbf{1_{Phe}}$ from the reaction of NCA $\mathbf{4_{Phe}}$ with 2-mercaptoethanesulfonate **5c** at pH 5 154
Formation of aminoacyl thiol $\mathbf{1_{Glu}}$ from the reaction of NCA $\mathbf{4_{Glu}}$ with 2-mercaptoethanesulfonate **5c** at pH 5. 155
Formation of aminoacyl thiol $\mathbf{1_{Asp}}$ from the reaction of NCA $\mathbf{4_{Asp}}$ with 2-mercaptoethanesulfonate **5c** at pH 5 156
Formation of aminoacyl thiol $\mathbf{1_{Glx}}$ from the reaction of NCA $\mathbf{4_{Glx}}$ with 2-mercaptoethanesulfonate **5c** at pH 5 . 158

Thiol-mediated aminoacylation of nucleosides 16 from *N*-carboxyanhydrides 4 .... **160**
General Procedure J for thiol catalysed aminoacylation of adenosine (**16A**) from NCA **4** and 2-mercaptoethanesulfonate (**5c**) 160
Formation of aminoacyl-adenosine $\mathbf{17^{A}_{Aaa}}$ from the reaction of adenosine (**16A**) with NCA $\mathbf{4_{Aaa}}$ in the presence of thiol **5c** 160
Thiol **5c**-catalysed formation of $\mathbf{17^{A}_{Ala}}$ from the reaction of adenosine (**16A**) with NCA $\mathbf{4_{Ala}}$ 161
Thiol **5c**-catalysed formation of $\mathbf{17^{A}_{Gly}}$ from the reaction of adenosine (**16A**) with NCA $\mathbf{4_{Gly}}$ 162
Thiol **5c**-catalysed formation of $\mathbf{17^{A}_{Leu}}$ from the reaction of adenosine (**16A**) with NCA $\mathbf{4_{Leu}}$ 163
Thiol **5c**-catalysed formation of $\mathbf{17^{A}_{Val}}$ from the reaction of adenosine (**16A**) with NCA $\mathbf{4_{Val}}$ 164
Thiol **5c**-catalysed formation of $\mathbf{17^{A}_{Ser}}$ from the reaction of adenosine (**16A**) with NCA $\mathbf{4_{Ser}}$ 165
Thiol **5c**-catalysed formation of $\mathbf{17^{A}_{Phe}}$ from the reaction of adenosine (**16A**) with NCA $\mathbf{4_{Phe}}$ 167
Thiol **5c**-catalysed formation of $\mathbf{17^{A}_{Glu}}$ from the reaction of adenosine (**16A**) with NCA $\mathbf{4_{Glu}}$ 168
Thiol **5c**-catalysed formation of $\mathbf{17^{A}_{Glx}}$ from the reaction of adenosine (**16A**) with NCA $\mathbf{4_{Glx}}$ 169
Thiol **5c**-catalysed formation of $\mathbf{17^{A}_{Asp}}$ from the reaction of adenosine (**16A**) with NCA $\mathbf{4_{Asp}}$ 170
Thiol **3c**-catalysed formation of $\mathbf{18^{A}_{Glu}}$ from the reaction of adenosine (**17A**) with aspartic anhydride $\mathbf{35_{Asp}}$ 173
Formation of aminoacyl thiol $\mathbf{1_{Asp}}$ from the reaction of aspartic anhydride $\mathbf{34_{Asp}}$ with 2-mercaptoethanesulfonate **5c** at pH 6.5 175
Formation of thioester $\mathbf{1_{Ala}}$ upon incubation of aminoacyl adenylate $\mathbf{2_{Ala}}$ with 3-mercaptopropionic acid **5b** 177

Preparation of synthetic Aminoacyl thioester L-$\mathbf{1^{n}_{Aaa}}$ **178**
General Procedure for synthetic amino aminoacyl thioester 178

Supplementary Discussion **188**

Observed pathways of aminonitrile reaction with thiols **188**

Formation of aminoacyl thiol $\mathbf{1_{Aaa}}$ **190**
General Procedure K – Formation of aminoacyl thiols **1** by the reaction of thiols **5** and aminonitriles **8** 190
Reaction of thiol **5** with alanine nitrile $\mathbf{5_{Ala}}$ 190
Reaction of alanine nitrile $\mathbf{8_{Ala}}$ with 3-mercaptopropanoic acid **5b** at specified pHs and room temperature 191
Reaction of alanine nitrile $\mathbf{8_{Ala}}$ with specified stoichiometry of 3-mercaptopropanoic acid **5b** 193
Reaction of alanine nitrile $\mathbf{8_{Ala}}$ with 3-mercaptopropanoic acid **5b** 195
Reaction of alanine nitrile $\mathbf{8_{Ala}}$ with 2-mercaptoethanesulfonate **5c** 197
Reaction of alanine nitrile $\mathbf{8_{Ala}}$ with N-acetylcysteamine **5d** 199
Reaction of alanine nitrile $\mathbf{8_{Ala}}$ with pantetheine **5a** 201

Reaction of alanine nitrile **8**$_{Ala}$ with 3-mercaptopropanoic acid **5b** at pH 7 and -7 °C ... 203
Side chain compatibility for the formation of thioesters **1**$^{b}_{Aaa}$ from the reaction of α-aminonitrile **8**$_{Aaa}$ with 3-mercaptopropanoic acid **5b** ... 204
Reaction of glycine nitrile **8**$_{Gly}$ with 3-mercaptopropanoic acid **5b** ... 205
Reaction of proline nitrile **8**$_{Pro}$ with 3-mercaptopropanoic acid **5b** ... 207
Reaction of leucine nitrile **8**$_{Leu}$ with 3-mercaptopropanoic acid **5b** ... 209
Reaction of methionine nitrile **8**$_{Met}$ with 3-mercaptopropanoic acid **5b** ... 211
Reaction of phenylalanine nitrile **8**$_{Phe}$ with 3-mercaptopropanoic acid **5b** ... 213
Reaction of serine nitrile **8**$_{Ser}$ with 3-mercaptopropanoic acid **5b** ... 215
Reaction of valine nitrile **8**$_{Val}$ with 3-mercaptopropanoic acid **5b** ... 217
Preparative synthesis of lysine nitrile **8**$_{Lys}$ ... 219
Reaction of lysine nitrile **8**$_{Lys}$ with 2-mercaptopropanoic acid **5b** ... 223
Reaction of pipecolic nitrile **8**$_{Pip}$ with 3-mercaptopropanoic acid **5b** ... 225
Reaction of arginine nitrile **8**$_{Arg}$ with 3-mercaptopropanoic acid **5b** ... 227
Competition reaction of alanine nitrile **8**$_{Ala}$ and N-acetylated alanine nitrile **38**$_{Ala}$ with 3-mercaptopropanoic acid **5b** ... 229
Competition reaction of alanine nitrile **8**$_{Ala}$ and lactonitrile **36** with 3-mercaptopropanoic acid **5b** ... 230
Competition reaction of α-alanine nitrile **8**$_{Ala}$ and β-alanine nitrile **37** with 3-mercaptopropanoic acid **5b** ... 232
Reaction of L-alanine nitrile **8**$_{Ala}$ with 3-mercaptopropanoic acid **5b** at pD 4 ... 235

Formation of peptidyl RNA 40 from aminoacyl RNA 17 ... 238
General Procedure L – Formation of peptidyl RNA **40** by the reaction of α-aminoacyl RNA **17** with α-amidothioacid **11** ... 238
General Procedure M – Formation of peptidyl RNA **40** by the reaction of α-aminoacyl RNA **17** with α-amidothioacid **11** ... 238
Peptidyl RNA **40**$^{n}_{Aaa}$ amidosynthesis ... 239
High-resolution mass spectrometry data for peptidyl-RNA **40**$^{n}_{Aaa}$ ... 240
Synthesis of peptidyl RNA **40**$^{A}_{ArgGlyAc}$ from aminoacyl-RNA **17**$^{A}_{Arg}$ with α-amidothioacid **11**$_{Gly}$ ... 241
Synthesis of peptidyl RNA **40**$^{G}_{ArgGlyAc}$ from aminoacyl-RNA **17**$^{G}_{Arg}$ with α-amidothioacid **11**$_{Gly}$ ... 242
Synthesis of peptidyl RNA **40**$^{U}_{ArgGlyAc}$ from aminoacyl-RNA **17**$^{U}_{Arg}$ with α-amidothioacid **11**$_{Gly}$ ... 243
Synthesis of peptidyl RNA **40**$^{C}_{ArgGlyAc}$ from aminoacyl-RNA **17**$^{C}_{Arg}$ with α-amidothioacid **11**$_{Gly}$ ... 244
Synthesis of peptidyl RNA **40**$^{A}_{ArgGlyGlyAc}$ from aminoacyl-RNA **17**$^{A}_{Arg}$ with α-amidothioacid **11**$_{GlyGly}$ ... 246
Synthesis of peptidyl RNA **40**$^{A}_{ArgGlyGlyGlyAc}$ from aminoacyl-RNA **17**$^{A}_{Arg}$ with α-amidothioacid **11**$_{GlyGlyGly}$ ... 247
Synthesis of peptidyl RNA **40**$^{A}_{ArgAlaAlaAc}$ from aminoacyl-RNA **17**$^{A}_{Arg}$ with α-amidothioacid **11**$_{AlaAla}$ ... 248
Synthesis of peptidyl RNA **40**$^{A}_{ArgProAlaAc}$ from aminoacyl-RNA **17**$^{A}_{Arg}$ with α-amidothioacid **11**$_{AlaPro}$ ... 250
Synthesis of peptidyl RNA **40**$^{A}_{ArgGlyMetAc}$ from aminoacyl-RNA **17**$^{A}_{Arg}$ with α-amidothioacid **11**$_{MetGly}$ ... 251
Synthesis of peptidyl RNA **40**$^{A}_{ArgPheAc}$ from aminoacyl-RNA **17**$^{A}_{Arg}$ with α-amidothioacid **11**$_{Phe}$ ... 252
Synthesis of peptidyl RNA **40**$^{A}_{ArgValAc}$ from aminoacyl-RNA **17**$^{A}_{Arg}$ with α-amidothioacid **11**$_{Val}$ ... 253
Synthesis of peptidyl RNA **40**$^{A}_{ArgMetAc}$ from aminoacyl-RNA **17**$^{A}_{Arg}$ with α-amidothioacid **11**$_{Met}$ ... 255
Synthesis of peptidyl RNA **42**$^{A}_{ArgGlyAc}$ from aminoacyl-RNA **41**$^{A}_{Arg}$ with α-amidothioacid **11**$_{Gly}$ ... 256
Synthesis of peptidyl RNA **42**$^{A}_{ArgGlyGlyAc}$ from aminoacyl-RNA **41**$^{A}_{Arg}$ with α-amidothioacid **11**$_{GlyGly}$ ... 257
Synthesis of peptidyl RNA **42**$^{A}_{ArgGlyGlyGlyAc}$ from aminoacyl-RNA **41**$^{A}_{Arg}$ with α-amidothioacid **11**$_{GlyGlyGly}$ ... 258
Synthesis of peptidyl RNA **40**$^{A}_{ArgGlyAc}$ from aminoacyl-RNA **17**$^{A}_{Arg}$ with α-amidothioacid **11**$_{Gly}$ ... 259
Synthesis of peptidyl RNA **40**$^{A}_{ArgGlyGlyGlyAc}$ from aminoacyl-RNA **17**$^{A}_{Arg}$ with α-amidothioacid **11**$_{GlyGlyGly}$ ... 261
Synthesis of peptidyl RNA **40**$^{A}_{ArgGlyAc}$ from aminoacyl-RNA **17**$^{C}_{Arg}$ with α-amidothioacid **11**$_{Gly}$ ... 262
Synthesis of peptidyl RNA **40**$^{A}_{ArgGlyAc}$ from aminoacyl-RNA **17**$^{U}_{Arg}$ with α-amidothioacid **11**$_{Gly}$ ... 263
One pot synthesis of peptidyl RNA **40**$^{A}_{ArgGlyAc}$ ... 264
One pot synthesis of peptidyl RNA **40**$^{A}_{ArgGlyAc}$ ... 265
One pot synthesis of peptidyl RNA **40**$^{A}_{AlaGlyAc}$ ... 266
Synthesis of peptidyl thioester **39**$_{GlyAlaAc}$ in presence of adenosine **16A** ... 267
Competition reaction adenosine with thioester **1**$_{Ala}$ and peptidyl thioester **39** in presence of adenosine **16A** ... 268
Synthesis of peptidyl RNA **40**$^{A}_{GlyGlyAc}$ from aminoacyl-RNA **17**$^{A}_{Gly}$ with α-amidothioacid **11**$_{Gly}$ ... 270
Synthesis of peptidyl RNA **40**$^{A}_{GluGlyAc}$ from aminoacyl-RNA **17**$^{A}_{Glu}$ with α-amidothioacid **11**$_{Gly}$ ... 272
Synthesis of peptidyl RNA **40**$^{A}_{AlaGlyAc}$ from aminoacyl-RNA **17**$^{A}_{Ala}$ with α-amidothioacid **11**$_{Gly}$ at pD 6.5 ... 274
Synthesis of peptidyl RNA **40**$^{A}_{LeuGlyAc}$ from aminoacyl-RNA **17**$^{A}_{Leu}$ with α-amidothioacid **11**$_{Gly}$ at pD 6.5 ... 275
Synthesis of peptidyl RNA **40**$^{A}_{SerGlyAc}$ from aminoacyl-RNA **17**$^{A}_{Ser}$ with α-amidothioacid **11**$_{Gly}$ at pH 6.0 ... 276
Synthesis of peptidyl RNA **40**$^{A}_{GlyValAc}$ from aminoacyl-RNA **17**$^{A}_{Gly}$ with α-amidothioacid **11**$_{Val}$ at pH 6.0 ... 277

Synthesis of peptidyl RNA **40$^{A}_{LysGlyAc}$** from aminoacyl-RNA **17$^{A}_{Lys}$** with α-amidothioacid **11$_{Gly}$** at pH 6.0..........279
Amine presence blocks acylation of **16A** with α-amidothioacid **11$_{Gly}$** at pH 6.0..283

Materials and Methods

Reagents and solvents were obtained and used without further purification, unless specified, from the following commercial sources: Fluorochem, Alfa Aesar, Acros Organics, Apollo Scientific, Merck, Fisher Scientific, VWR International, Biosynth, Manchester Organics, Lancaster, Molekula, Flukka Honeywell, Tokyo Chemical Industry (TCI), Integrated DNA Technologies (IDT), Santa Cruz Biotechnology. Aminonitriles (**8**),[1] aminoacyl thiols (**1**)[2,3,4] *N*-carboxyanhydrides (**4**)[5,6] and α-amidothioacid (**11**)[1] were prepared by previously reported protocols. Deionised water was obtained from an Elga Option 3 purification system and degassing by three freeze-vacuum-thaw cycles or sonication under vacuum. Thin layer chromatography (TLC) was carried out on Merck aluminium-backed DC 60 F254 0.2 mm pre-coated plates. TLC plates were visualised under ultraviolet light and stained with permanganate solution when required. Solution pH values were measured using a Mettler Toledo Seven Compact pH meter with a Mettler Toledo InLab semi-micro pH probe. Flash column chromatography was performed on a Biotage Isolera One using Kinesis TELOS cartridges. ^{1}H and ^{13}C NMR spectra were recorded on Bruker NMR spectrometers AVANCE Neo 700, equipped with a Bruker room temperature 5 mm multinuclear gradient probe, a 5 mm DCH cryoprobe (700 MHz) or on a AVANCE III 600 equipped with a 5 mm DCH cryoprobe (600 MHz) and a AVANCE Neo 500, equipped with a Bruker room temperature 5 mm multinuclear gradient probe (500 MHz). ^{13}C spectra were proton decoupled. Chemical shifts (δ) are reported in parts per million (ppm) relative to residual solvent peaks, and ^{1}H chemical shifts relative to TMS were calibrated using the residual solvent peak or an internal NMR reference ((methanesulfonyl)methane (MSM), sodium acetate, pentaerythritol (PET)). Nuclear assignments were made using 2D NMR homo- and heteronuclear correlation spectroscopy (^{1}H-^{1}H COSY; ^{1}H-^{13}C HSQC; ^{1}H-^{13}C HMBC). Solvent suppression pulse sequence with pre-saturation and spoil gradients were used to obtain ^{1}H NMR spectra (noesygppr1d, Bruker) and ^{1}H-^{13}C HMBC NMR spectra (hmbcgplpndprqf, Bruker). Coupling constants are reported in hertz (Hz). Spin multiplicities are indicated by symbols: s (singlet); d (doublet); t (triplet); q (quartet); qn (quintet); spt (septet); oct (octet), m (multiplet); obs. (obscured/coincidental signals); app. (apparent), or a combination of these. Infrared spectra (IR) were recorded on a Bruker Alpha FT-IR with a platinum-ATR (attenuated total reflection) attachment as a solid or neat oil/liquid. Absorption maxima are reported in wavenumber (cm^{-1}) and the spectral range was between 400 cm^{-1} and 4000 cm^{-1} with a resolution of 0.01 cm^{-1}. Diastereotopic geminal spin systems are reported as AB or ABX. NMR data are reported as follows: chemical shift (number of protons, multiplicity, coupling constants (J), nuclear assignment). NMR spectra were recorded at 293 K. Mass spectra and accurate mass measurements were recorded on a Waters LCT Premier QTOF connected to a Waters Autosampler Manager 2777C, Thermo Finnigan MAT900, an Agilent LC connected to an Agilent 6510 QTOF mass spectrometer and a Shimadzu-8030 dual-polarity linear MALDI-TOF.

WARNING: Hydrogen cyanide, hydrogen sulfide and their salts are highly toxic poisons by contact, inhalation, and ingestion. The salts generate poisonous hydrogen cyanide (pK_a = 9.2) and hydrogen sulfide (pK_a = 7.0) gas at neutral or acidic pH. Any solutions that contain cyanide, sulfide or compounds that may generate these must be handled in a well-ventilated fume hood with appropriate chemical quenches at hand (such as sodium hypochlorite (bleach)). Material safety data sheets (MSDS) and instructions for personnel handling, exposure, and disposal information must be read and followed. Local safety personnel should be consulted for regulations concerning proper and safe disposal according to local environmental guidelines.

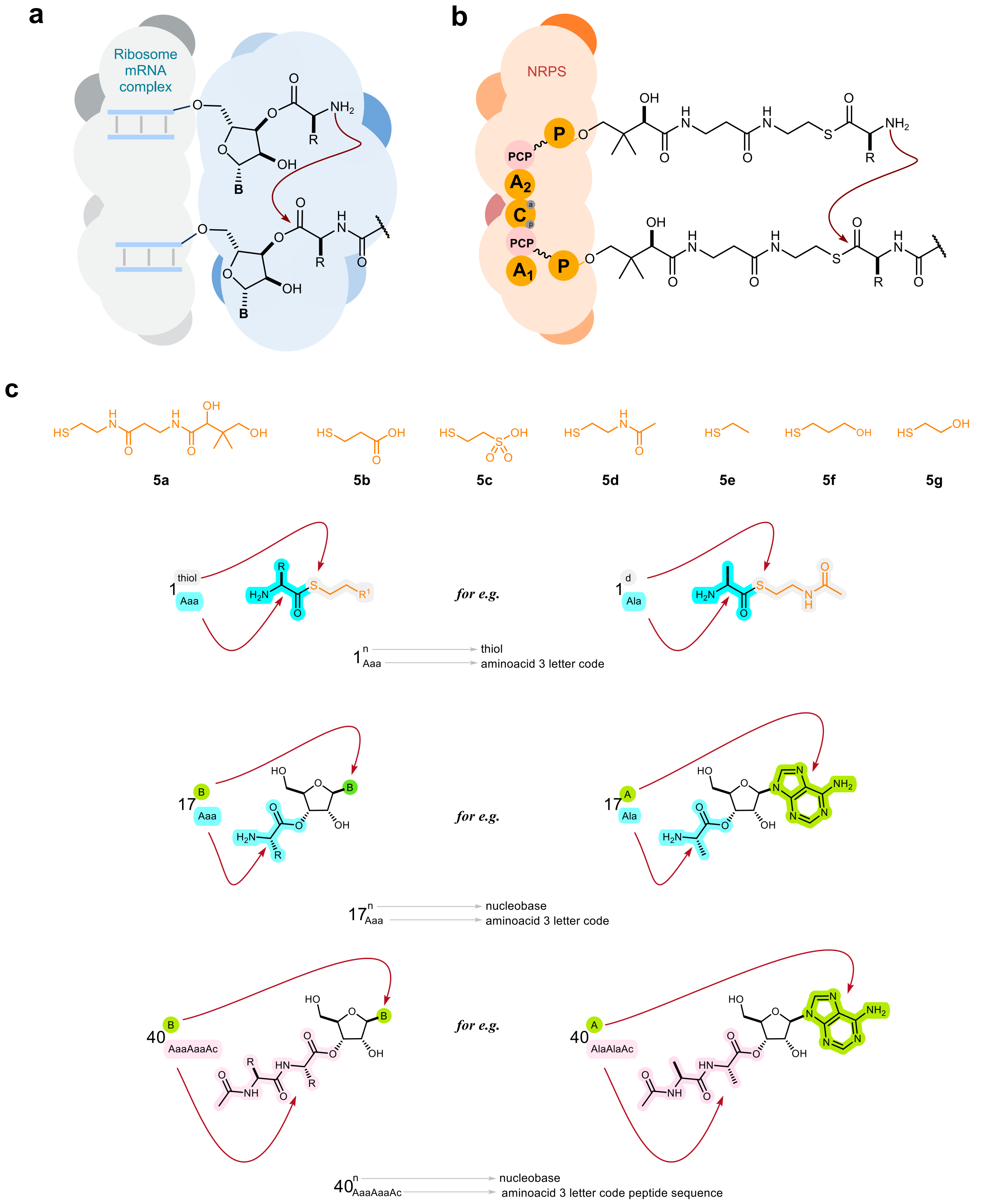

Supplementary Figure 1. ***a)*** *Schematic representation of ribosomal peptide synthesis, which is mediated by aminoacyl-RNA esters.* ***b)*** *Schematic representation of non-ribosomal peptide synthesis, which is mediated by aminoacyl-pantetheine thioesters.* ***c)*** *Thiols (**5a-g**), aminoacyl-thiol (**1**), aminoacyl-RNA (**17**) and peptidyl-RNA (**40**) descriptors. Thiol moieties are designated in aminoacyl thiols (**1**) by superscript letter (a-g as depicted). Amino acid and peptide residues are designated by subscript three letter code (Aaa) in all compounds. Nucleobases moieties are designated by superscript single letter code (A, C, G, U).*

Results and data

Reaction of thioester $\mathbf{1^e_{Aaa}}$ with nucleophiles

*General Procedure A – reactivity of **L-1$_{Ala}$** with potential nucleophiles*

L-Alanine thioester **L-1^e_{Ala}**(0.1 mmol), specified nucleophile (0.15 – 0.25 mmol), and pentaerythritol (PET, 10 mM) were dissolved in degassed H_2O/D_2O (98:2, 0.9 mL). The solution was adjusted to the desired pH with HCl/NaOH and the solution volume was set to 1 mL. The solution was incubated at room temperature, and NMR spectra were periodically acquired and quantified against PET as an internal standard. Products were characterised by ^1H-^{13}C HMBC and spiking with authentic standards where available.

α-Aminoacyl-thioester dimerisation at specified pHs

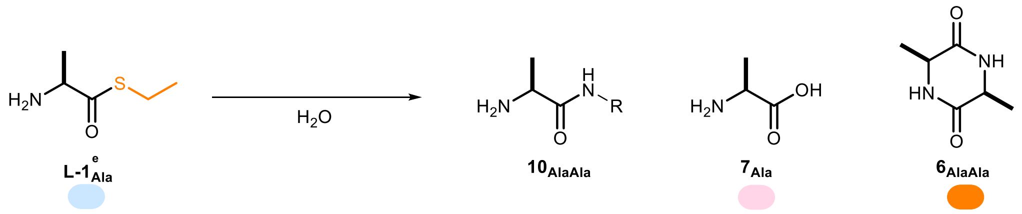

Entry	pH	Buffer (200 mM)	Time (hours)	$\mathbf{6_{AlaAla}}$ (%)	$\mathbf{7_{Ala}}$ (%)	$\mathbf{10_{AlaAla}}$ (%)	**L-1^e_{Ala}** (%)
1	6.5	--	24	0.5	6.5	n.d.	93
			120	1	11	n.d.	88
2	6.5	Imidazole	24	2	16	n.d.	80
			120	2.5	26	n.d.	69
3	7	--	24	2.5	9	n.d.	85
			120	2.5	15	n.d.	80
4	7	Phosphate	24	2	24	n.d.	72
			120	2	48	n.d.	48

*Supplementary Table 1. Yields of alanine-DKP(**6_{AlaAla}**), hydrolysis (**7_{Ala}**) and peptide formation (**10_{AlaAla}, Ala_3**) when L-**1^e_{Ala}** (100 mM) in incubated in the specified buffer (200 mM). Set up following General Procedure A.*

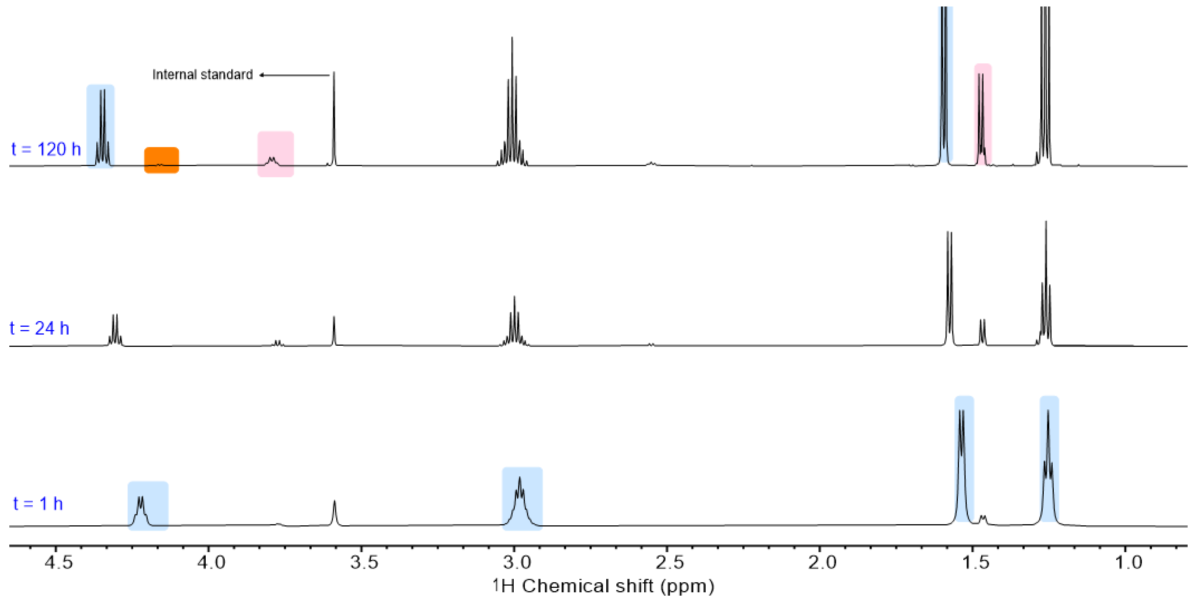

Supplementary Figure 2. ^{1}H NMR (600 MHz, H_2O/D_2O 9:1, noesygppr1d, 1.0 – 4.5 ppm) spectra to show the unbuffered reaction of L-alanine thioester (L-$\mathbf{1}^{e}_{Ala}$, 100 mM) with itself at pH 7, using PET (10 mM) as an internal standard. Set up following General Procedure A.

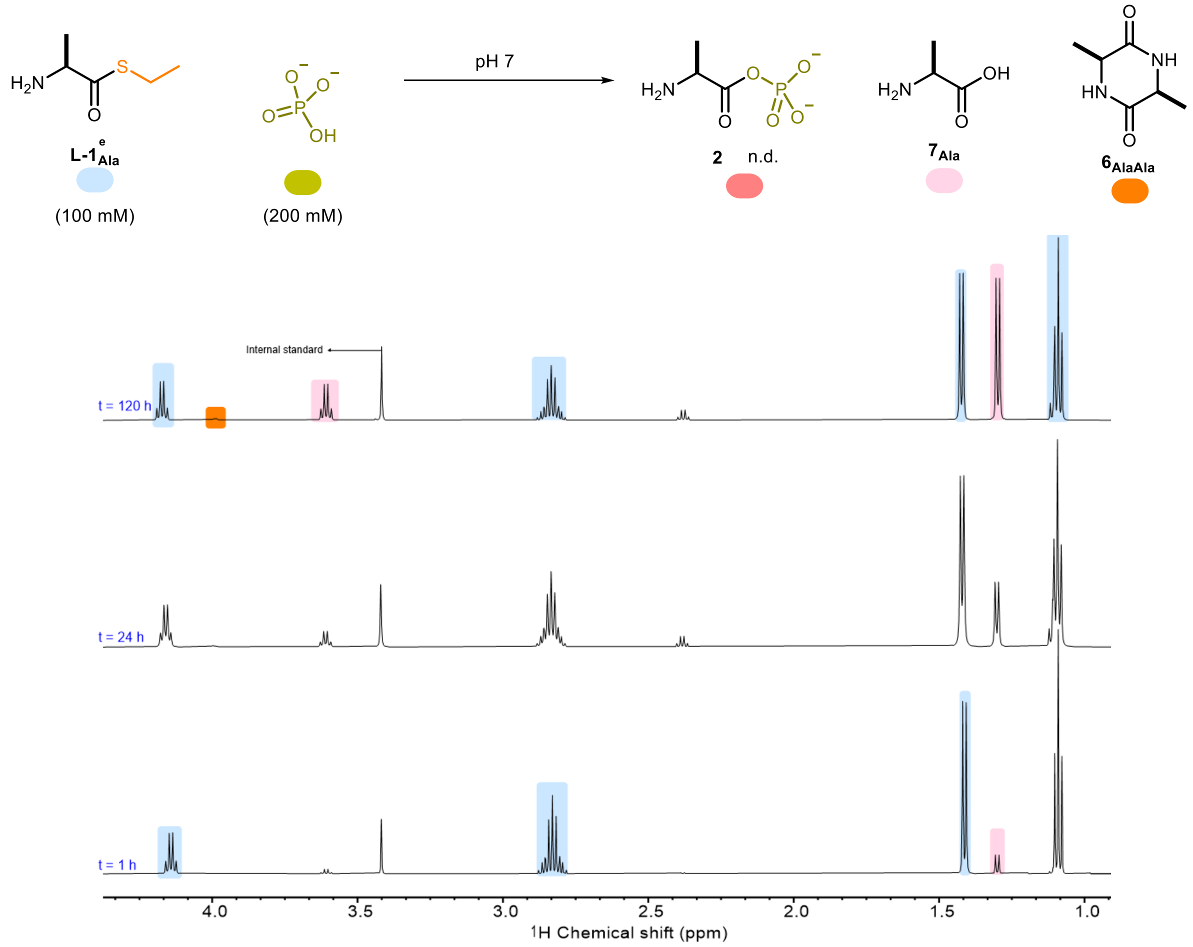

Supplementary Figure 3. ^{1}H NMR (600 MHz, H_2O/D_2O 9:1, noesygppr1d, 1.0 – 4.5 ppm) spectra to show the reaction of L-alanine thioester (L-$\mathbf{1}^{e}_{Ala}$, 100 mM) in NaH_2PO_4 (200 mM) at pH 7, using PET (10 mM) as an internal standard. Set up following General Procedure A.

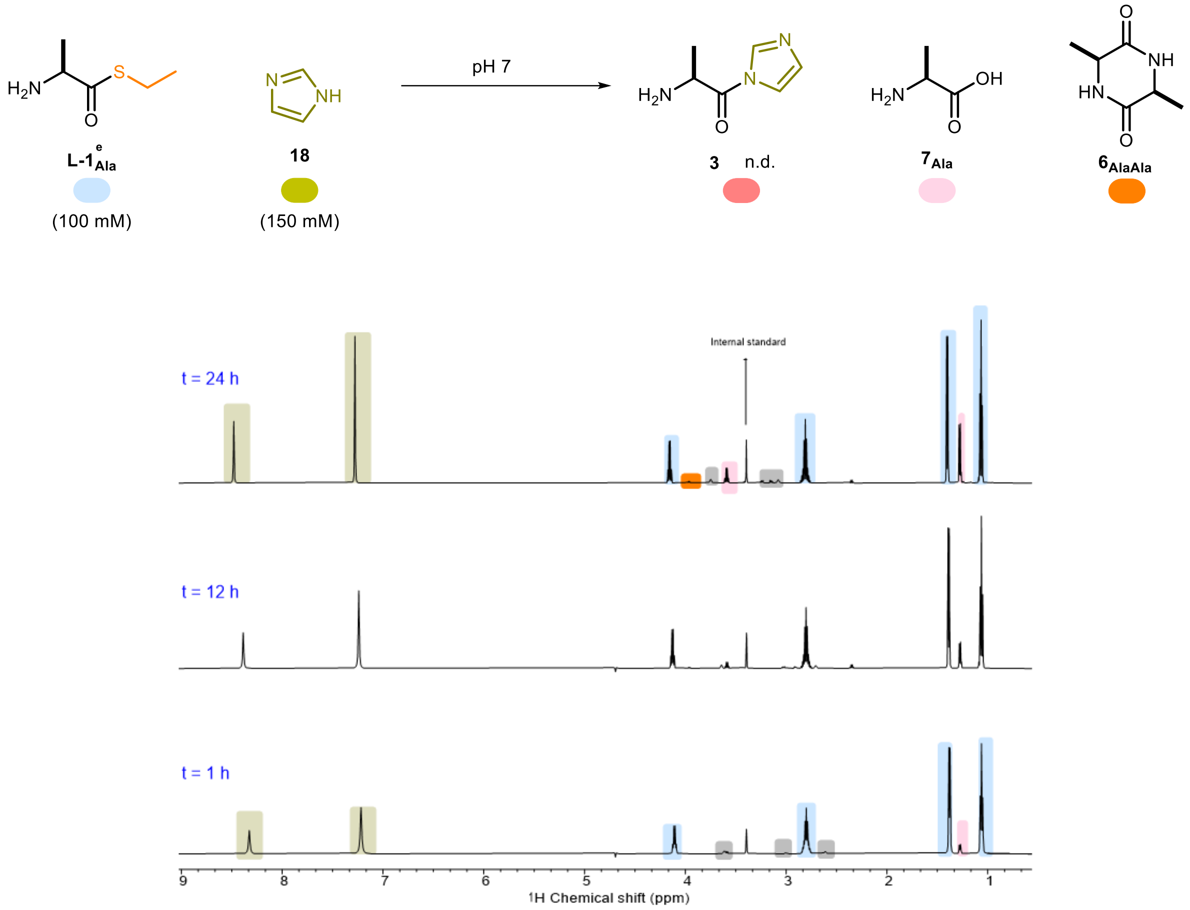

Supplementary Figure 4. *^{1}H NMR (600 MHz, H_2O/D_2O 9:1, noesygppr1d, 1.0 – 9.0 ppm) spectrum shows the reaction of amino alanine thioester (L-$\mathbf{1}^{\mathbf{e}}_{\mathbf{Ala}}$, 100 mM) in imidazole (**18**, 200 mM) at pH 7, using PET (10 mM) as an internal standard. Set up following General Procedure A. Grey squares = contaminated with MES buffer.*

Reaction of α-aminoacyl thiol with nucleophiles

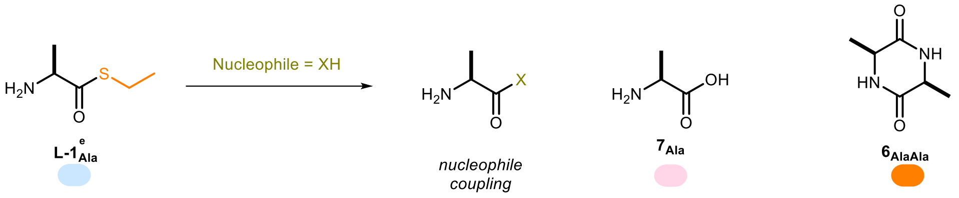

Entry	Nucleophile	mM	Time (hours)	pH	amino acylated product (%)	$\mathbf{1}_{Ala}$ (%)	$\mathbf{9}_{AlaAla}$ (%)
1	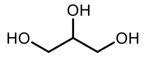 **19**	160	24	7	n.d.	21	4
2	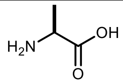 **L-7$_{Ala}$**	150	24	7	n.d.	20	<2
3	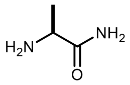 **L-10$_{Ala}$**	150	24	7	n.d.	15	<2
4	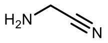 **8$_{Gly}$**	140	12<br>24	7<br>7	8<br>8	6<br>11	obs.[1]
5	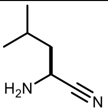 **8$_{Leu}$**	220	24	7	n.d.	60	6
6	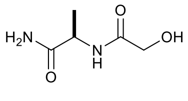 **13$_{AlaGly}$**	150	48	7	n.d.	12	<2
7	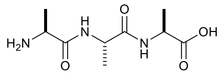 **Ala$_3$**	170	48	7	n.d.	20	<2
8	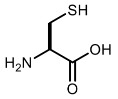 **L-7$_{Cys}$**	150	1	7	93	6	n.d.
9	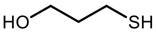 **5f**	150	24	7	53	21	10
10	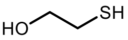 **5g**	150	24	7	37	44*	n.d.

*Supplementary Table 2. % yield of reaction of L-$\mathbf{1}^{e}_{Ala}$ (100 mM) with specified nucleophile (150 – 250 mM) at room temperature. Set up following General Procedure A. n.d. = not detected. obs. = signal obscured by resonance overlap. * Thiirane (14%) is observed.*

[1] The yield for alanine-DKP ($\mathbf{6}_{AlaAla}$) is not reported due to signal overlap. No further attempt has been made to quantify this by-product, as the yield was low and comparable to all other reactions in this series. The yield of H-Ala-Gly-CN ($\mathbf{9}_{AlaGly}$) appeared to be steady-state (8%) over 12–24 hours; this is likely due to slow onward reaction of $\mathbf{13}_{AlaGly}$ with $\mathbf{7}_{Gly}$, catalysed by thiol **5e**. Further investigation of this sluggish dinitrile coupling will be the subject of future work.

*Reaction of L-alanine thioester L-**1**$_{Ala}$ with glycerol **19***

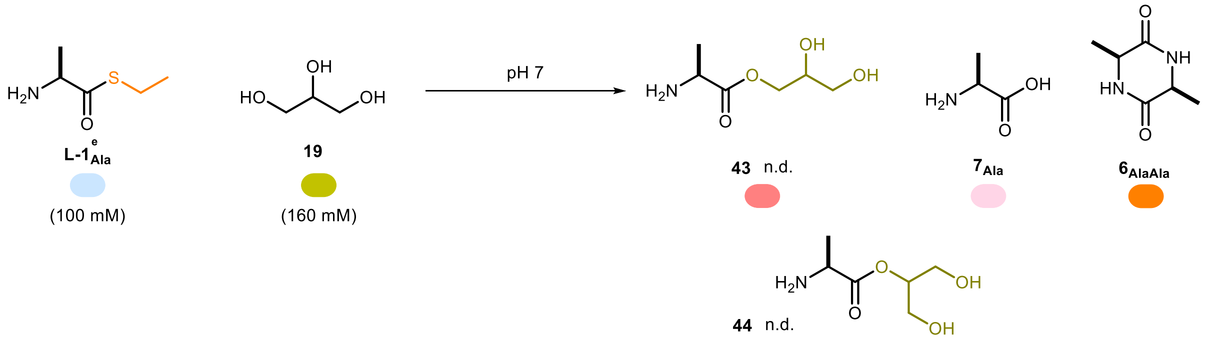

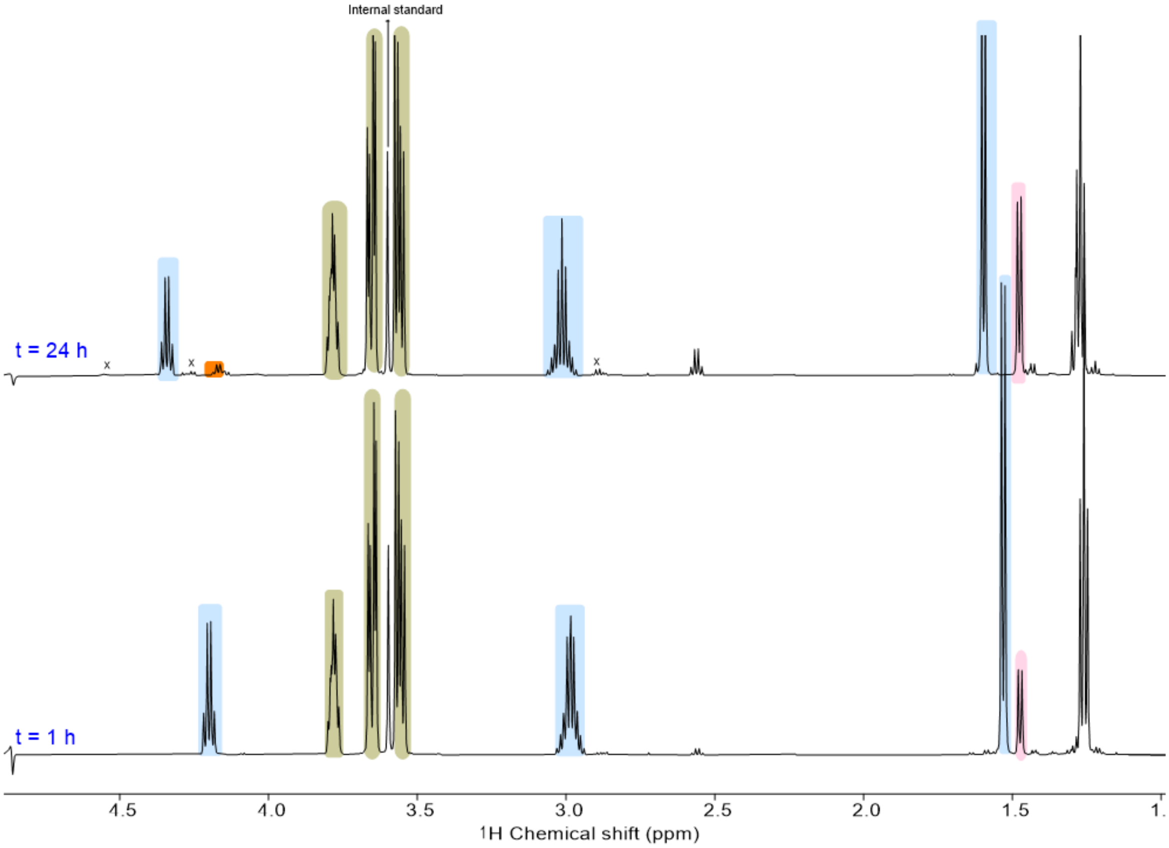

*Supplementary Figure 5. ¹H NMR (600 MHz, H_2O/D_2O 9:1, noesygppr1d, 1.0–4.7 ppm) spectra to show the reaction of L-alanine thioester (**L-1**$^e_{Ala}$, 100 mM) with glycerol (160 mM) at pH 7, using PET (10 mM) as an internal standard. Set up following General Procedure A. x = Tentatively assigned as Ala-Ala-SEt, formed by the reaction of L-**1**$^e_{Ala}$ with itself.*

*Reaction of L-alanine thioester L-**1**$_{Ala}$ with L-alanine L-**7**$_{Ala}$*

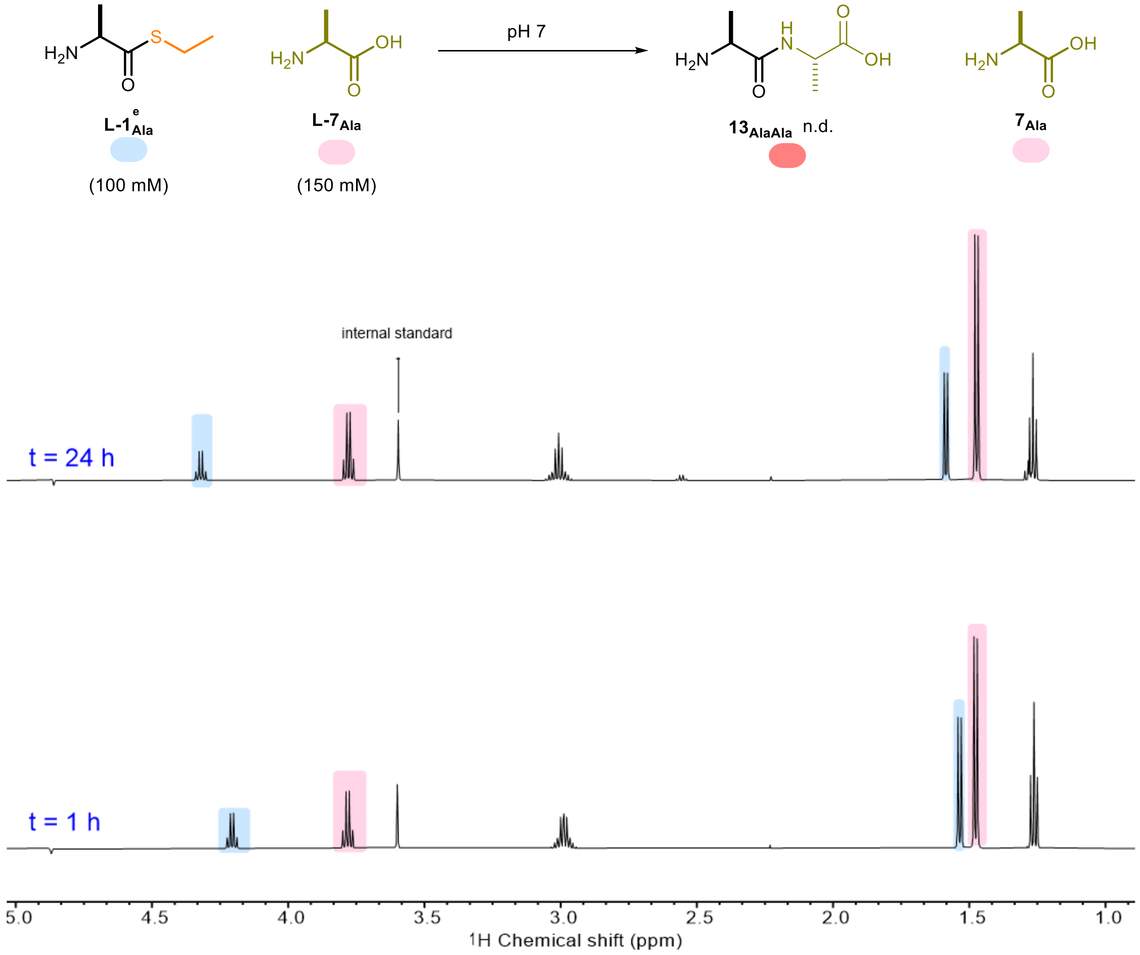

Supplementary Figure 6. [1]*H NMR (600 MHz, H_2O/D_2O 9:1, noesygppr1d, 1.0–5.0 ppm) spectra to show the reaction of L-alanine thioester (L-**1**$^e_{Ala}$, 100 mM) with (L-**7**$_{Ala}$, 150 mM) at pH 7, using PET (10 mM) as an internal standard. Set up following General Procedure A.*

*Reaction of L-alanine thioester L-**1**$_{Ala}$ with L-alanine amide L-**10**$_{Ala}$*

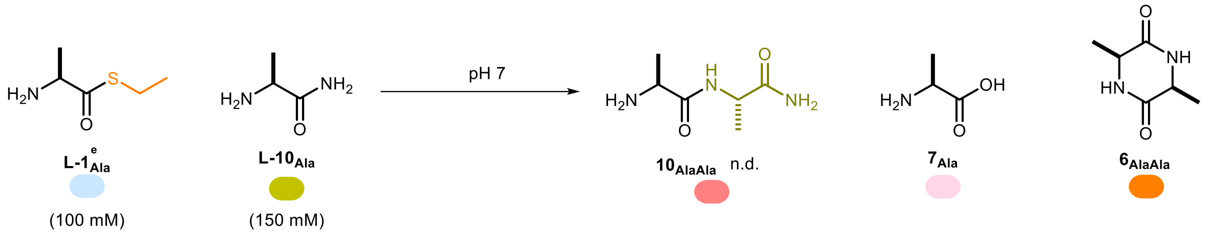

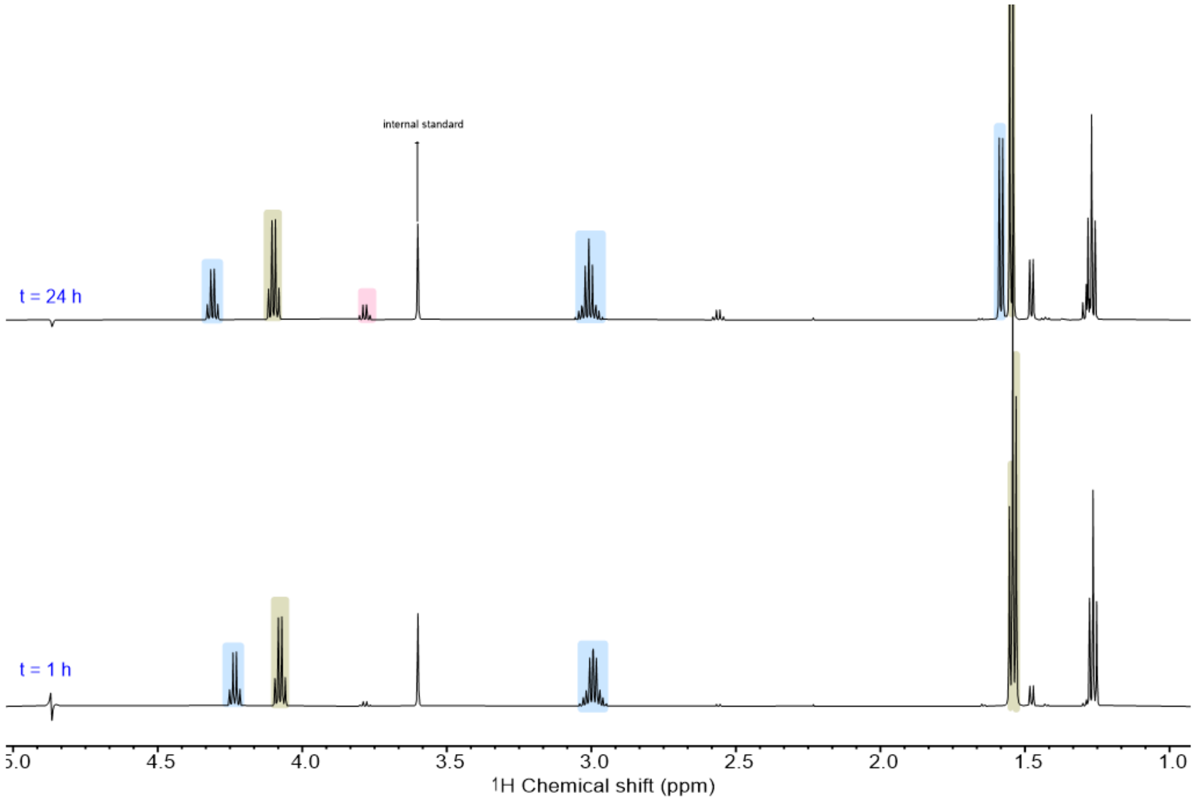

Supplementary Figure 7. *^{1}H NMR (600 MHz, H_2O/D_2O 9:1, noesygppr1d, 1.0–5.0 ppm) spectrum shows the reaction of L-alanine thioester (L-**1**$^{e}_{Ala}$, 100 mM) with L-alanine amide (L-**10**$_{Ala}$, 150 mM) at pH 7, using PET (10 mM) as an internal standard. Set up following General Procedure A.*

Reaction of L-alanine thioester L-$\mathbf{1}_{Ala}$ with glycine nitrile $\mathbf{8}_{Gly}$

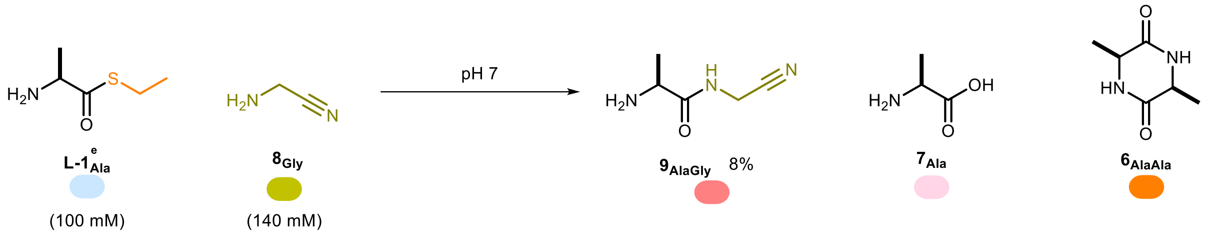

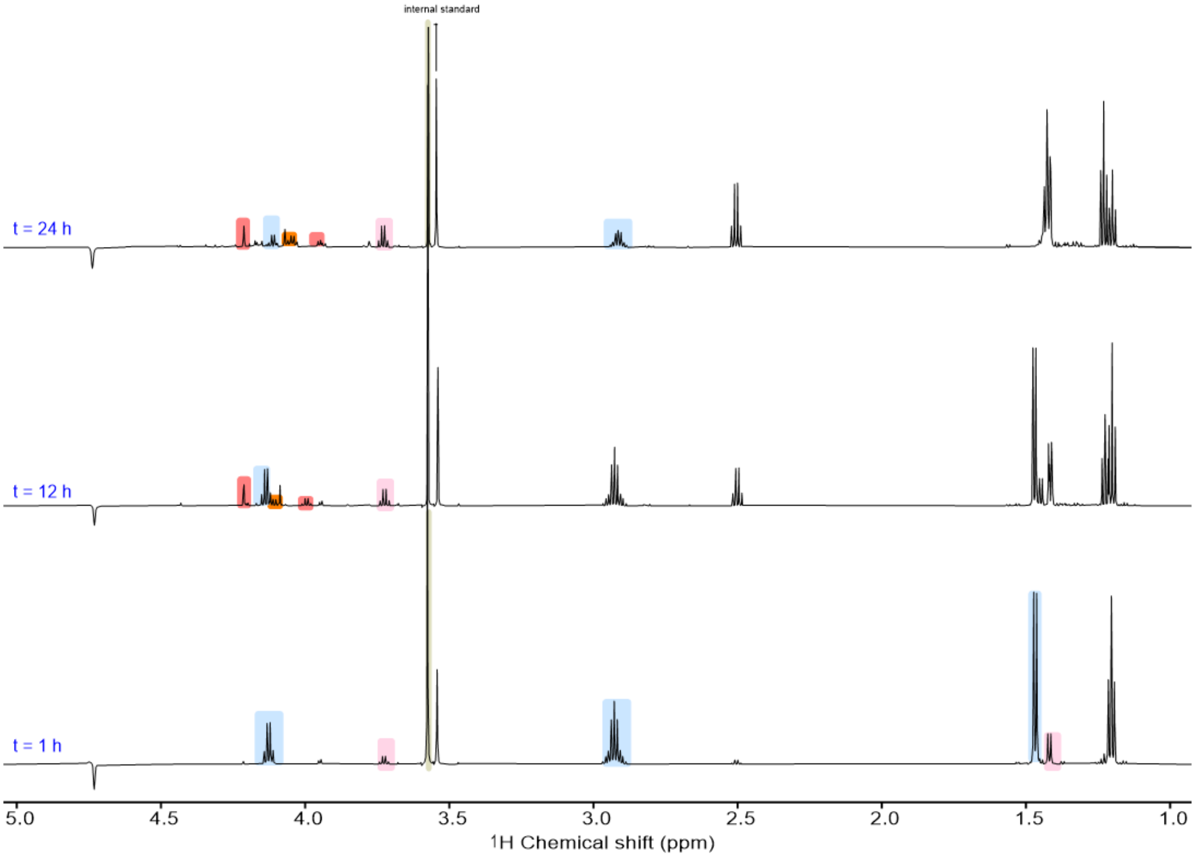

Supplementary Figure 8. ^{1}H NMR (600 MHz, H_2O/D_2O 9:1, noesygppr1d, 1.0 – 5.0 ppm) spectrum shows the reaction of L-alanine thioester (L-$\mathbf{1}^{e}_{Ala}$, 100 mM) with glycine nitrile ($\mathbf{8}_{Gly}$, 140 mM) at pH 7, using PET (10 mM) as an internal standard. Set up following General Procedure A.

^{1}H NMR (600 MHz, H_2O/D_2O 9:1) $\mathbf{9}_{AlaGly}$: δ_H 4.27 (1H, d, J = 1.2 Hz, CONHCH_2CN), 4.06 (1H, q, J = 7.1 Hz, Ala-α-CH(CH_3)).[2]

$\mathbf{6}_{AlaAla}$: δ_H 4.17 (1H, obs., Ala-αCH(CH_3)CONH).

$\mathbf{7}_{Ala}$: δ_H 3.78 (1H, q, J = 7.2 Hz, Ala-αH-COOH.

[2] ^{1}H NMR data and ^{1}H–^{13}C HMBC spectra reported after 12 hours, more signal overlap is observed at 24 hours.

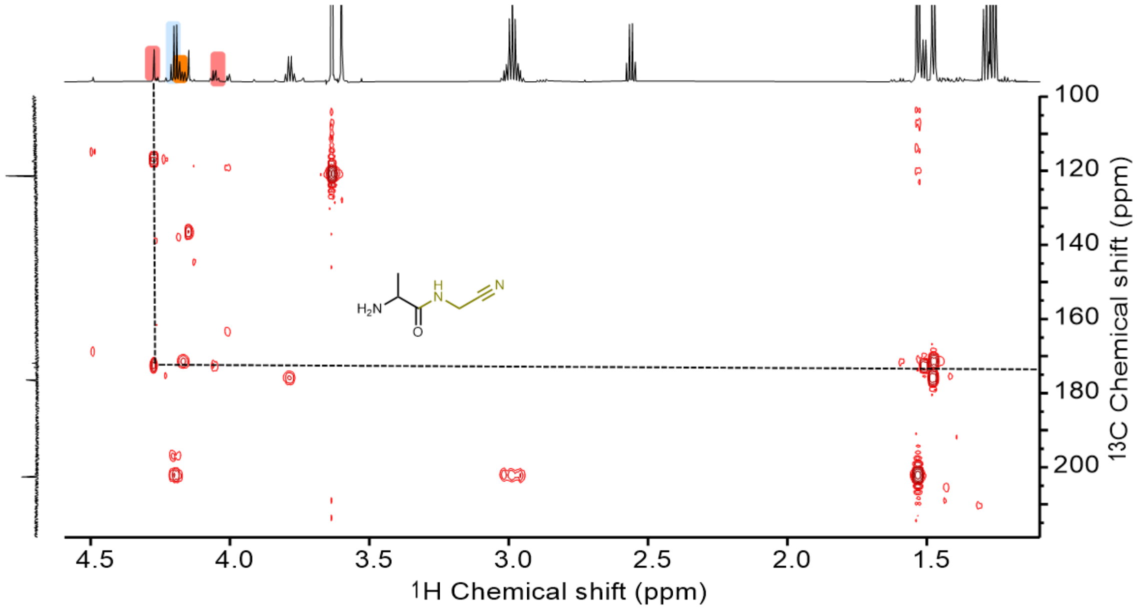

Supplementary Figure 9. ^{1}H–^{13}C *HMBC (*^{1}*H: 600 MHz [1.0 – 4.5 ppm],* ^{13}*C: 176 MHz [100 – 210 ppm],* H_2O/D_2O *9:1) spectrum showing the diagnostic* ^{2}*JCH and* ^{3}*JCH coupling of glycine-a-H and* $-CH_2CN$ *in* $\mathbf{9}_{\mathbf{AlaGly}}$ *at 4.27 ppm with two resonances at 116.5 and 172.8 ppm, which is characteristic for peptidyl nitrile bond formation.*

Reaction of L-alanine thioester L-$\mathbf{1}_{Ala}$ with leucine nitrile $\mathbf{8}_{Leu}$

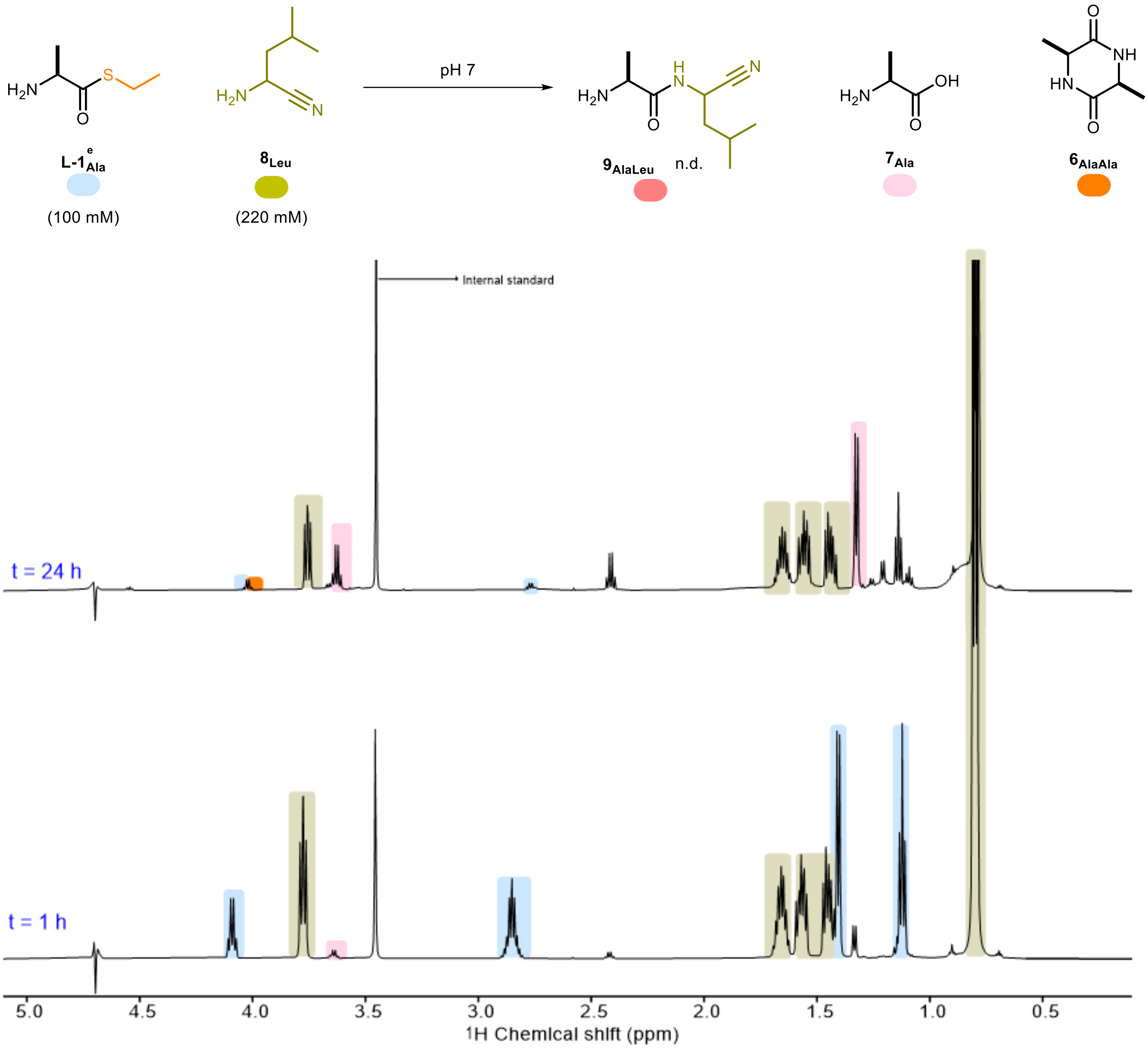

Supplementary Figure 10. ^{1}H NMR (600 MHz, H_2O/D_2O 9:1, noesygppr1d, 0.5 – 5.0 ppm) spectra to show the reaction of L-alanine thioester (L-$\mathbf{1}^e_{Ala}$, 100 mM) with leucine nitrile ($\mathbf{8}_{Leu}$, 220 mM) at pH 7, using PET (20 mM) as an internal standard. Set up following General Procedure A.

Reaction of L-alanine thioester L-$\mathbf{1_{Ala}}$ with L-alanylglycine $\mathbf{13_{AlaGly}}$

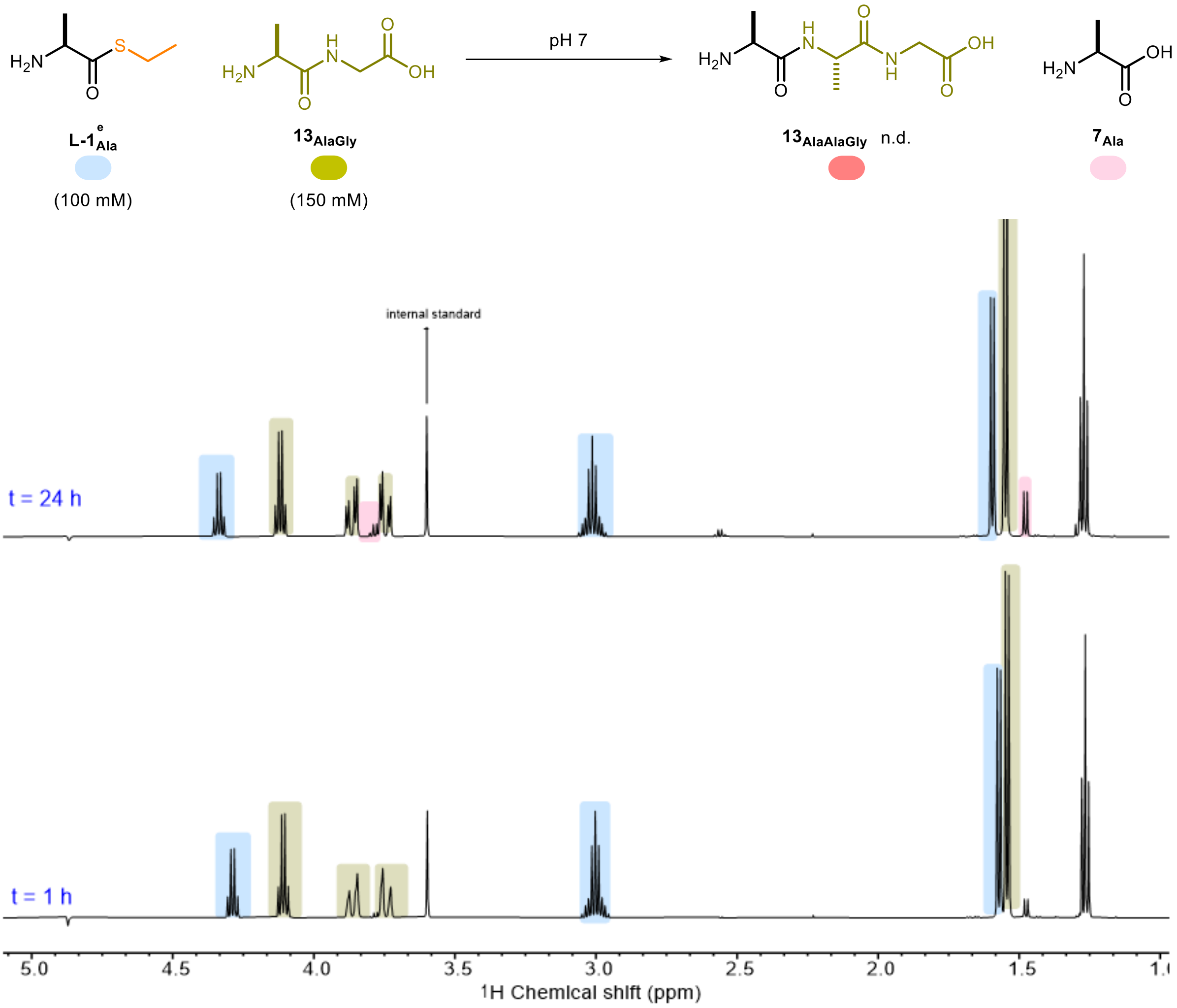

Supplementary Figure 11. ^{1}*H NMR (600 MHz, H_2O/D_2O 9:1, noesygppr1d, 1.0 – 5.0 ppm) spectrum to show the reaction of L-alanine thioester (L-$\mathbf{1^e_{Ala}}$, 100 mM) with $\mathbf{13_{AlaGly}}$ (150 mM) at pH 7, using PET (10 mM) as an internal standard. Set up following General Procedure A.*

*Reaction of L-alanine thioester L-**1**$_{Ala}$ with L, L, L-alanylalanylalanine **Ala$_3$***

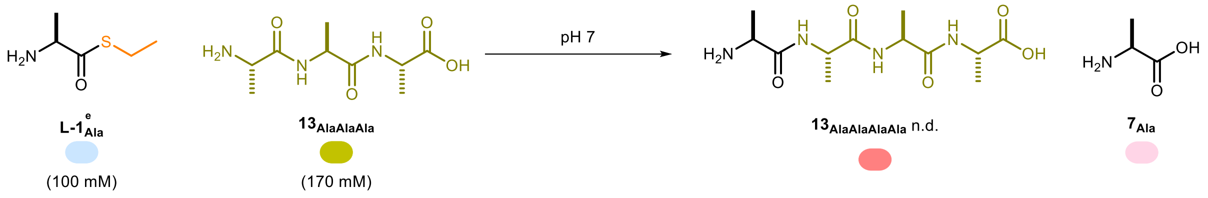

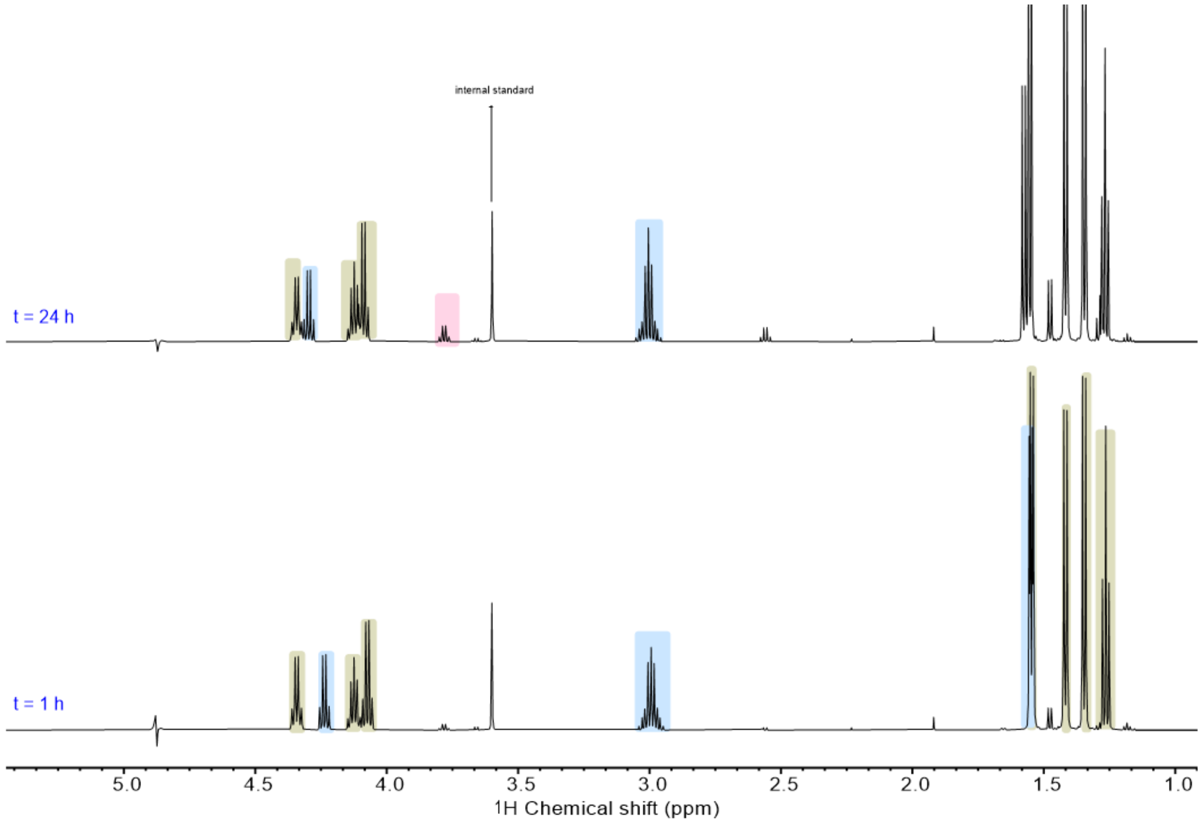

*Supplementary Figure 12. ¹H NMR (600 MHz, H_2O/D_2O 9:1, noesygppr1d, 1.0 – 5.5 ppm) spectra to show the reaction of L-alanine thioester (L-**1**$^e_{Ala}$, 100 mM) with **Ala$_3$** (170 mM) at pH 7, using PET (10 mM) as an internal standard. Set up following General Procedure A.*

*Reaction of L-alanine thioester L-**1**$_{Ala}$ with L-cysteine L-**7**$_{Cys}$*

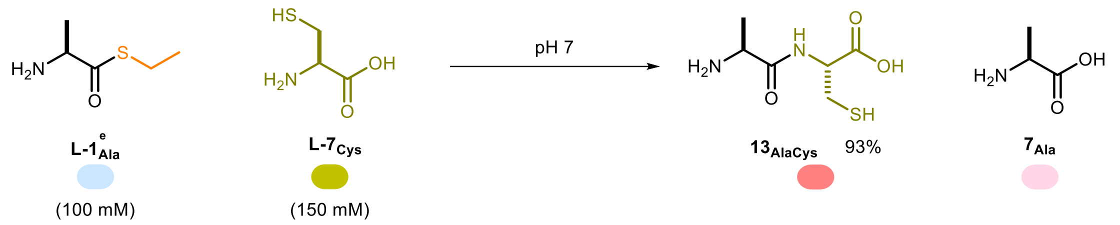

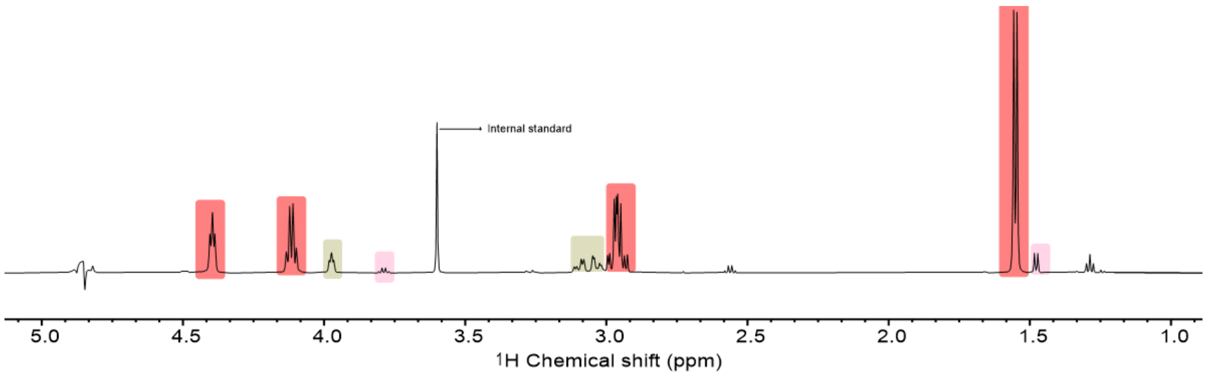

*Supplementary Figure 13. ^{1}H NMR (600 MHz, H_2O/D_2O 9:1, noesygppr1d, 1.0 – 5.0 ppm) spectrum to show the reaction of L-alanine thioester (L-**1**$^{e}_{Ala}$, 100 mM) with L-cysteine (L-**7**$_{Cys}$, 150 mM) at pH 7 after 10 mins, using PET (10 mM) as an internal standard. Set up following General Procedure A.*

^{1}H NMR (600 MHz, H_2O/D_2O 9:1) **13**$_{AlaCys}$, (partial assignment) : δ_H 4.40 (1H, dd, *J* = 6.3, 4.5 Hz, Cys-α-C*H*CH$_2$SH), 4.19 – 4.06 (1H, q, *J* = 7.1 Hz, Ala-α-C*H*(CH3)), 3.00 – 2.91 (2H, m, *J* = 7.1 Hz, α-CHC*H*$_2$SH), 1.55 (3H, d, *J* = 7.1 Hz, Ala-α-CH(C*H*$_3$)).

7$_{Ala}$: δ_H 3.79 (1H, q, *J* = 7.2 Hz, Ala-α*H*-COOH).

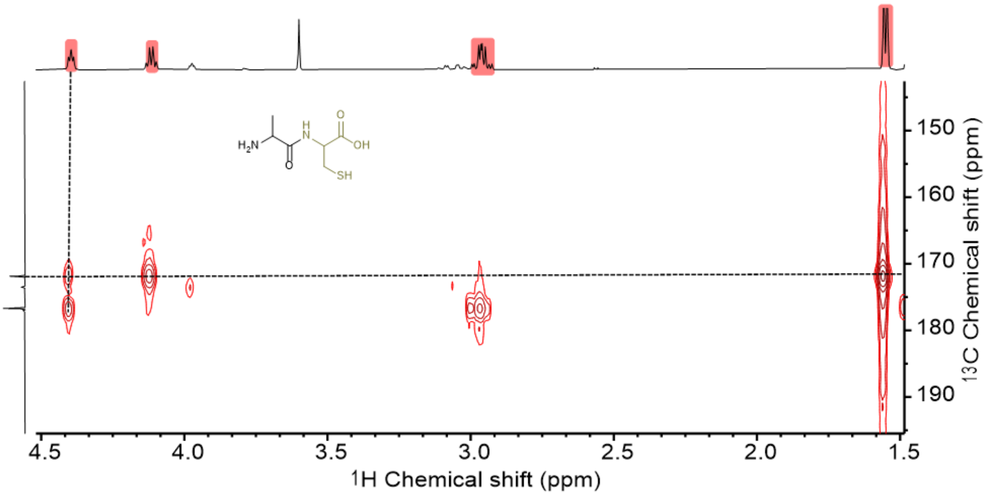

*Supplementary Figure 14. ^{1}H–^{13}C HMBC (^{1}H: 600 MHz [1.5 – 4.5 ppm], ^{13}C: 176 MHz [140 – 207 ppm], H_2O/D_2O 9:1) spectrum showing the diagnostic ^{2}JCH and ^{3}JCH coupling of Cys-aCH-COOH and Cys-aCH-CONH- in ligated product **13**$_{AlaCys}$ at 4.40 ppm with two resonances at 171.8 and 176.6 ppm, which is characteristic of amide bond formation.*

*Competition reaction of serine **7**$_{Ser}$ with alanine thioester L-**1**$_{Ala}$*

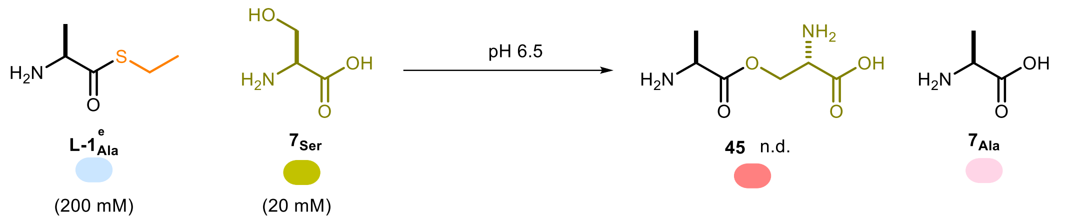

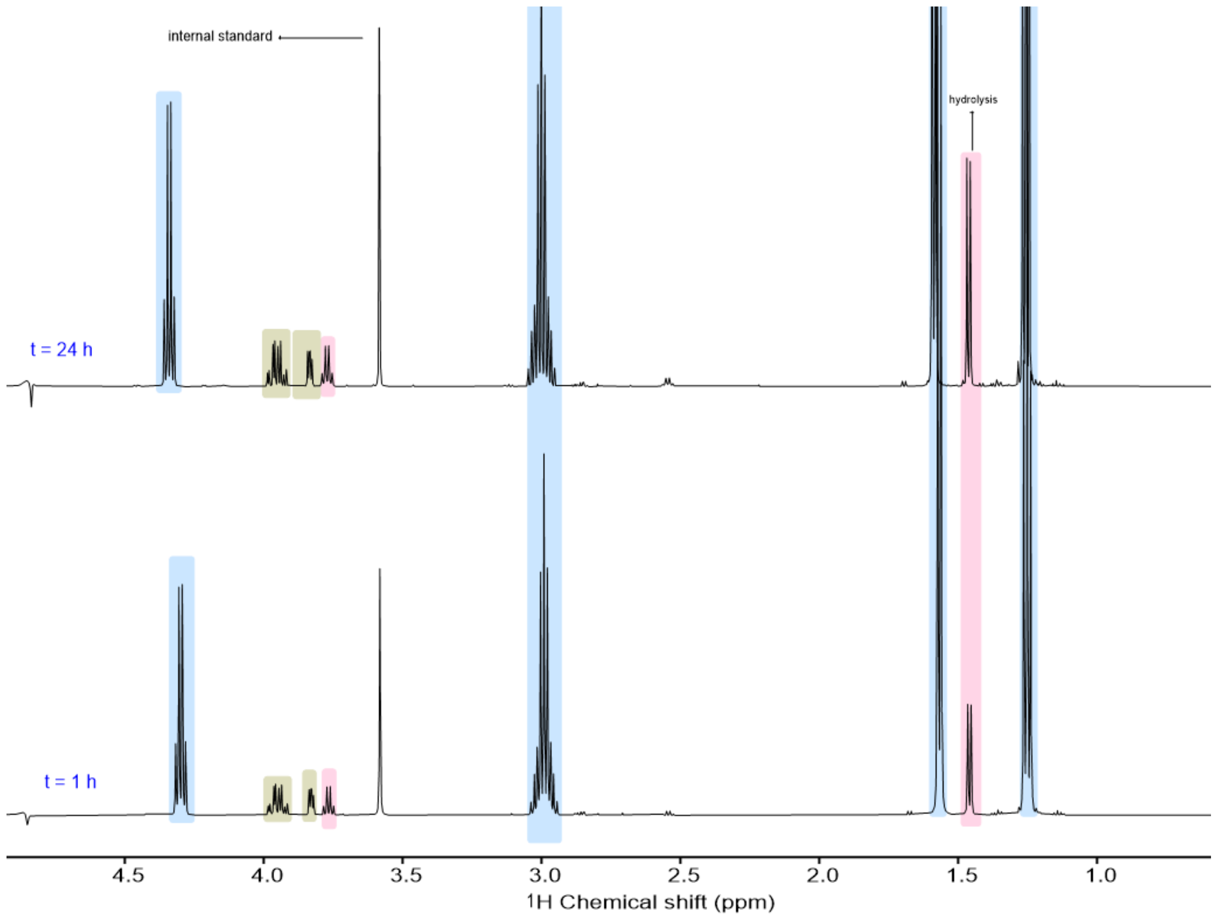

*Supplementary Figure 15: ^{1}H NMR (600 MHz, H_2O/D_2O 9:1, noesygppr1d, 1.0 – 5.0 ppm) spectra to show the reaction of L-alanine thioester (L-**1**$^e_{Ala}$, 200 mM) and serine (**7**$_{Ser}$, 20 mM) at pH 6.5, using PET (10 mM) as an internal standard. Set up following General Procedure A.*

*Reaction of L-alanine thioester L-**1**$_{Ala}$ with 3-mercaptopropanol **5f***

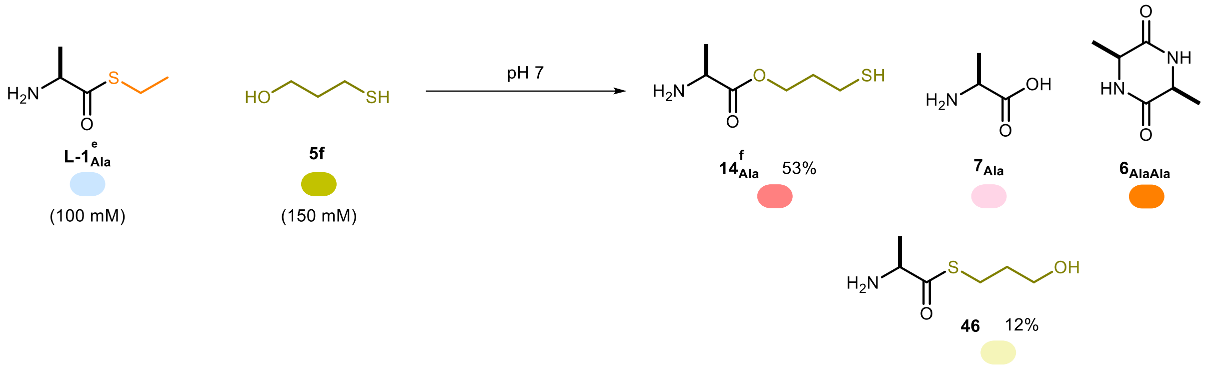

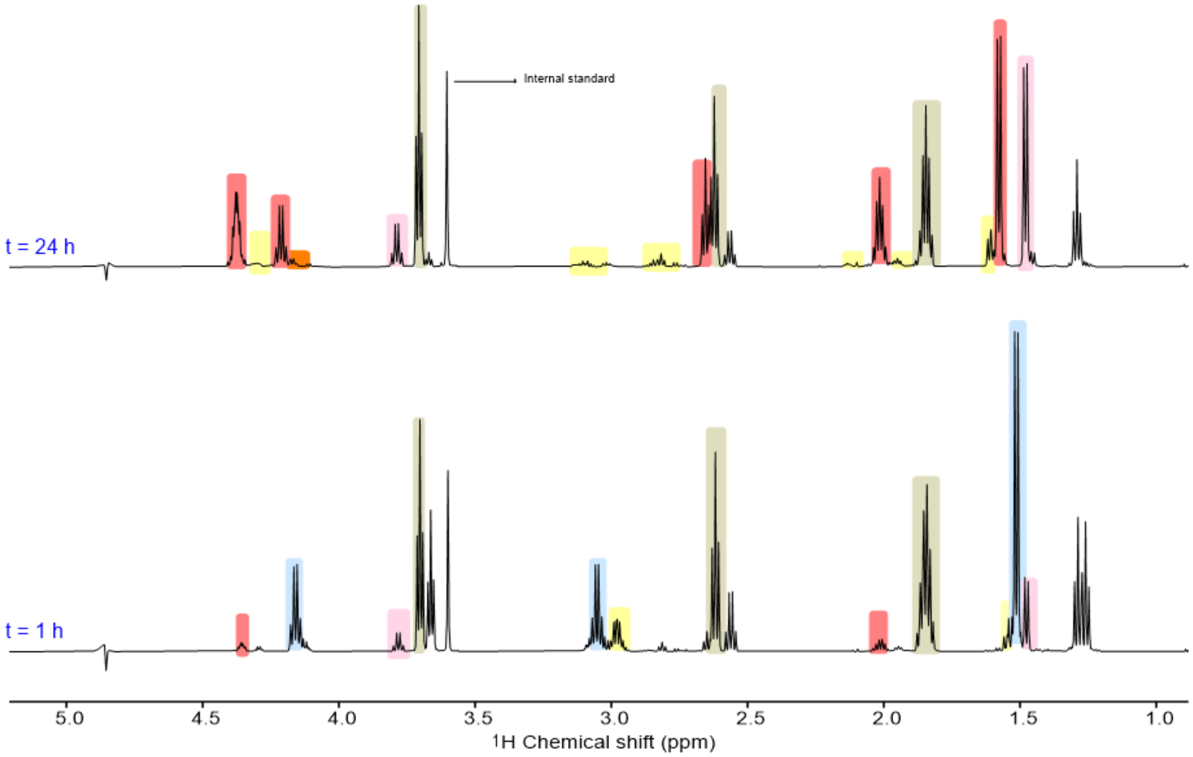

*Supplementary Figure 16. ^{1}H NMR (600 MHz, H_2O/D_2O 9:1, noesygppr1d, 1.0 – 5.0 ppm) spectrum shows the reaction of L-alanine thioester (L-$\mathbf{1}^{e}_{Ala}$, 100 mM) with 3-mercaptopropanol (**5f**, 150 mM) at pH 7, using PET (10 mM) as an internal standard. Set up following General Procedure A.*

^{1}H NMR (600 MHz, H_2O/D_2O 9:1) $\mathbf{14^{f}_{Ala}}$ (partial assignment) : δ_H 4.37 (2H, td, *J* = 6.2, 3.5 Hz, CHCOOC*H*$_2$), 4.21 (1H, q, *J* = 7.3 Hz, α-C*H*COOCH$_2$), 2.65 (2H, t, *J* = 7.1 Hz, COOCH$_2$CH$_2$C*H*$_2$SH), 1.97 – 1.93 (2H, m, CHCOOCH$_2$C*H*$_2$CH$_2$SH), 1.57 (3H, d, J = 7.3 Hz, CH(C*H*$_3$)).

46 (partial assignment) : δ_H 4.33 (1H, overlapped, ala-α-C*H*(CH$_3$)), 3.23 – 2.96 (2H, m, CHCOSC*H*$_2$).

7$_{Ala}$: δ_H 3.78 (1H, q, *J* = 7.2 Hz, ala-α*H*-COOH), 1.48 (3H, d, *J* = 7.2 Hz CH(C*H*$_3$)).

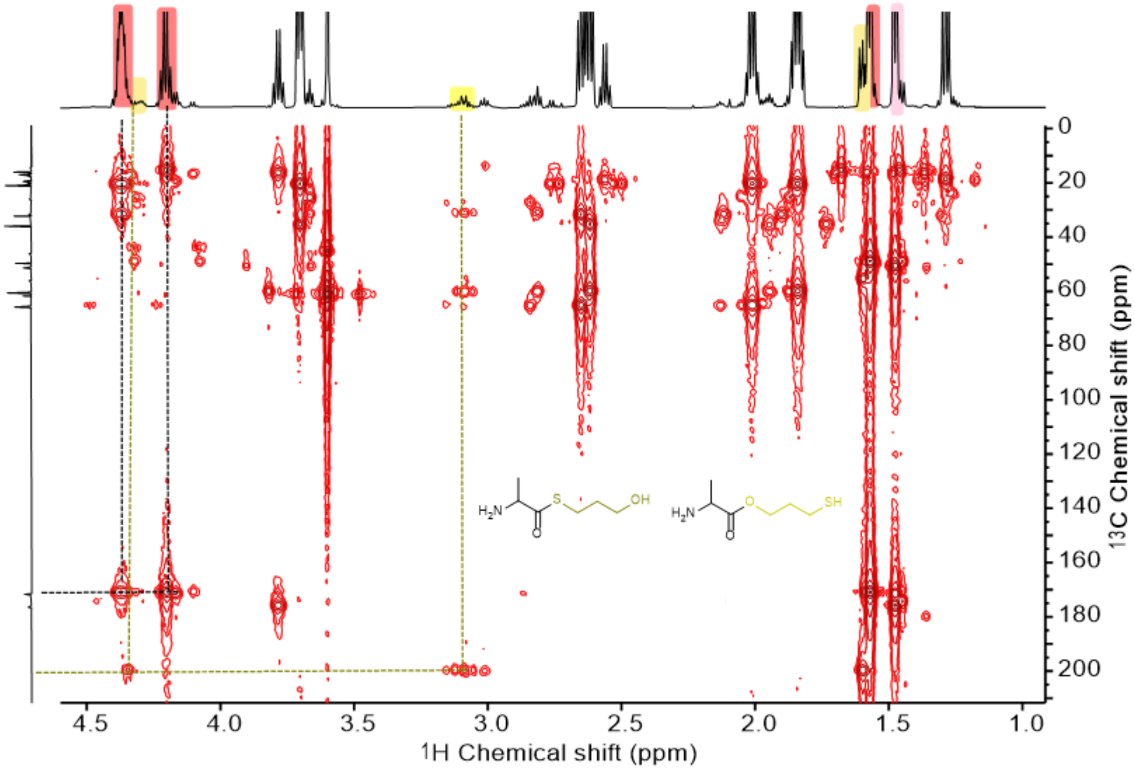

Supplementary Figure 17. *^1H–^{13}C HMBC (^1H: 600 MHz [1.0 – 4.5 ppm], ^{13}C: 176 MHz [0 – 210 ppm], H_2O/D_2O 9:1) spectrum showing the diagnostic ^2JCH and ^3JCH, coupling of alanyl α-H and -OCH_2 in **14^f_{Ala}** at 4.21 and 4.37 with one resonance at 170.8 ppm, which is characteristic peak of aminoacyl ester bond formation **14^f_{Ala}**. A second resonance was also observed, characterized by diagnostic ^2JCH and ^3JCH coupling of the alanyl a-H and -SCH2 groups in **46** at 4.33 and 3.08 ppm, with one resonance at 199.7 ppm, which is characteristic of thioester bond formation in **46**.*

*Reaction of L-alanine thioester L-$\mathbf{1}_{Ala}$ with 2-mercaptoethanol **5g***

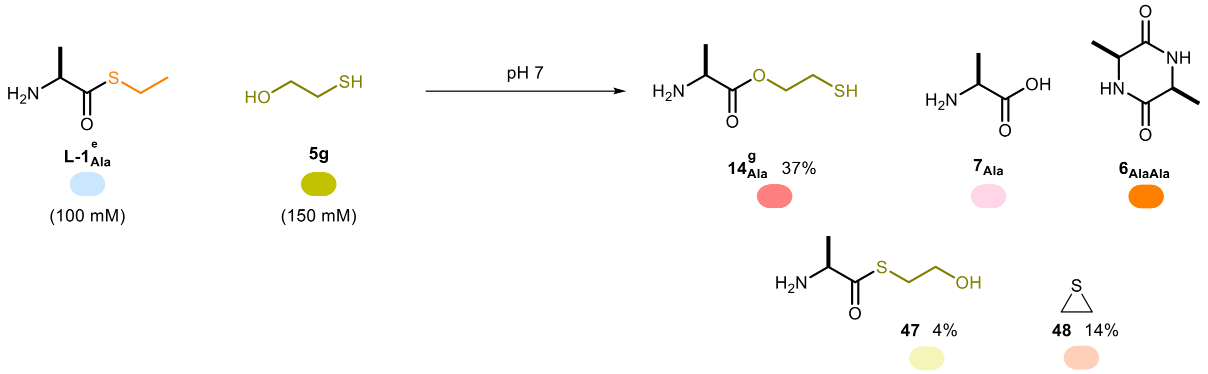

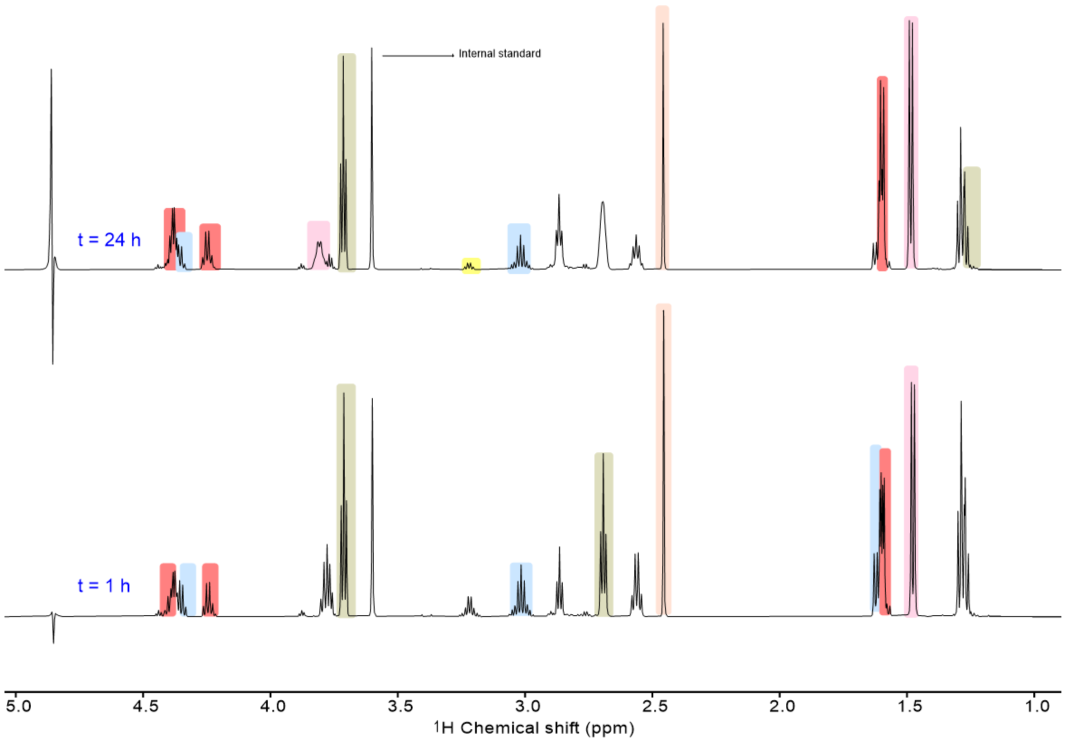

*Supplementary Figure 18. ^{1}H NMR (600 MHz, H_2O/D_2O 9:1, noesygppr1d, 1.0 – 5.0 ppm) spectra to show the reaction of L-alanine thioester (L-$\mathbf{1}^{e}_{Ala}$, 100 mM) with 2-mercaptoethanol (**5g**, 150 mM) at pH 7, using PET (10 mM) as an internal standard. Set up following General Procedure A.*

^{1}H NMR (600 MHz, H_2O/D_2O 9:1) $\mathbf{14}^{g}_{Ala}$(partial assignment): δ_H 4.38 (2H, td, *J* = 6.2, 4.1 Hz, CHCOOC*H*$_2$), 4.25 (1H, q, *J* = 7.2 Hz, α-C*H*COOCH$_2$), 1.60 (3H, d, *J* = 7.2 Hz, CH(C*H*$_3$));

47 (partial assignment): δ_H 3.28 – 3.17 (2H, m, ala-α CHCOSC*H*$_2$).

$\mathbf{1}^{e}_{Ala}$ (partial assignment): δ_H 3.02 (2H, p, *J* = 7.6 Hz, Ala-α CHCOSC*H*$_2$).

$\mathbf{7}_{Ala}$: δ_H 3.81 (1H, m, Ala-α*H*-COOH), 1.48 (3H, d, *J* = 7.2 Hz).

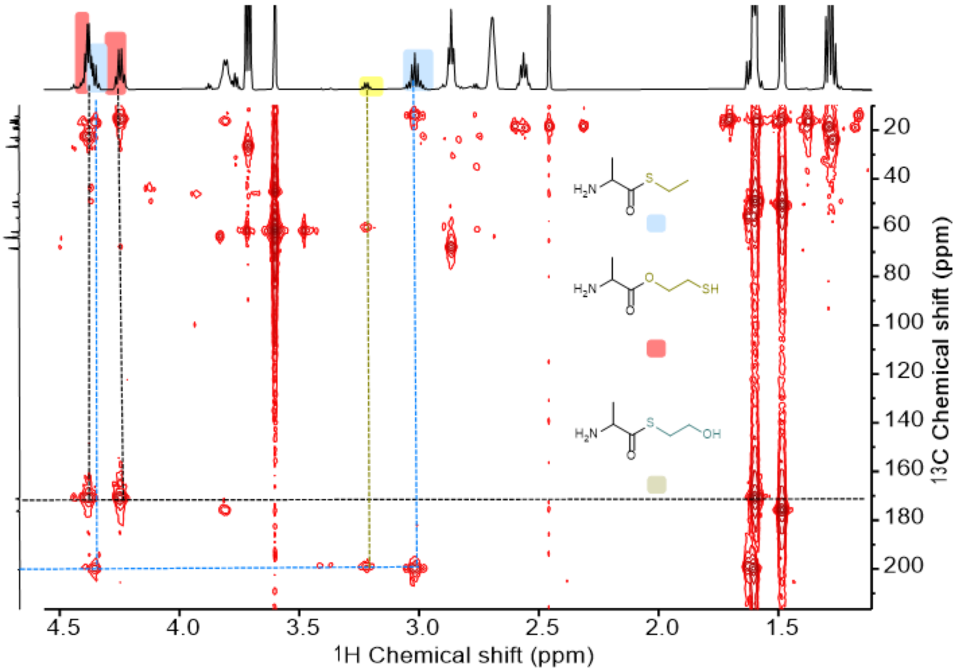

Supplementary Figure 19. *^{1}H–^{13}C HMBC (^{1}H: 600 MHz [1.5 – 4.5 ppm], ^{13}C: 176 MHz [10 – 210 ppm], H_2O/D_2O 9:1) spectrum showing the diagnostic ^{2}JCH and ^{3}JCH, coupling of alanyl α-CH and OCH_2 in* ***14^{g}_{Ala}*** *at 4.25 and 4.38 ppm with one resonance at 170.9 ppm, which is characteristic for aminoacyl ester bond formation in* ***14^{g}_{Ala}****. A second resonance was also observed which is diagnostic ^{2}JCH and ^{3}JCH, coupling of alanyl α-H and -SCH_2 in* ***47*** *at 4.35 (overlapped) and 3.37 with one resonance at 199.3 ppm, which is characteristic of thioester in* ***47****. A third resonance was also observed which is diagnostic ^{2}JCH and ^{3}JCH, coupling of both alanyl α-H and -SCH_2 in* ***1^{e}_{Ala}*** *at 4.35 and 3.00 with one resonance at 199.3 ppm, which is characteristic peak of residual starting material.*

Synthesis of α-aminoacyl ester $\mathbf{14_{Ala}}$ *from alanine nitrile* $\mathbf{8_{Ala}}$ *and 3-mercaptopropanol* **5f** *at different pHs*

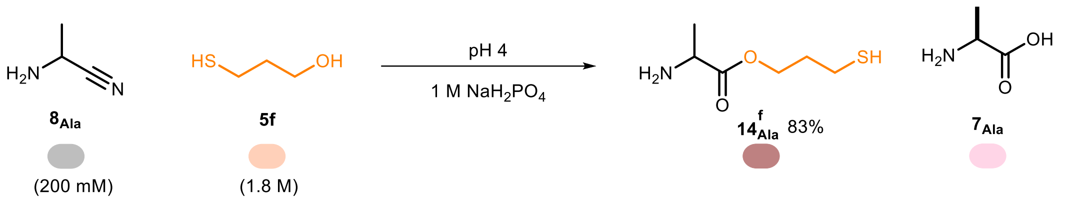

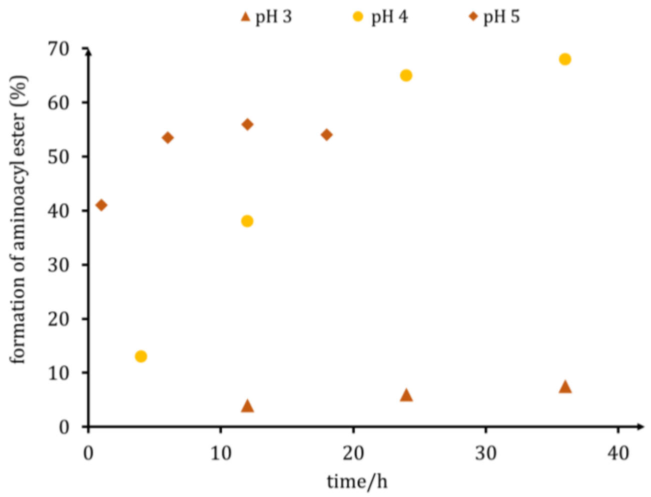

Supplementary Figure 20. Yield of $\mathbf{14^{f}_{Ala}}$ *plotted against time for the reaction of alanine nitrile (*$\mathbf{8_{Ala}}$*, 200 mM) with 3-mercaptopropanol (***5f***, 1.8 M) at the specified pH and room temperature.*

Entry	$\mathbf{8_{Aaa}}$	$\mathbf{7_{Aaa}}$ (%)	$\mathbf{14^{f}_{Aaa}}$ (%)
1	Ala	15	83
2	Pro	19	72
3	Ser	12	87

Supplementary Table 3. The yield of aminoacyl ester $\mathbf{14^{f}_{Ala}}$ *from the reaction of an α-aminonitrile (*$\mathbf{8_{Aaa}}$*, 200 mM), 3-mercaptopropanol (***5f***, 1.8 M) and 1 M* NaH_2PO_4 *at pH 4 and room temperature, following General Procedure A.*

Synthesis of α-aminoacyl ester ***14*** $_{\mathbf{Ala}}$ *from the reaction of alanine nitrile* ***8*** $_{\mathbf{Ala}}$ *with 3-mercaptopropanol* ***5f*** *at pH 4*

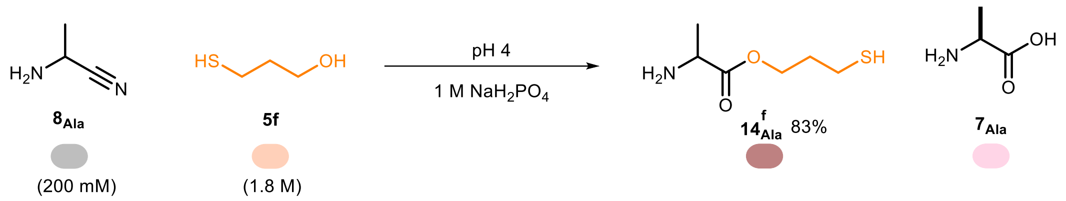

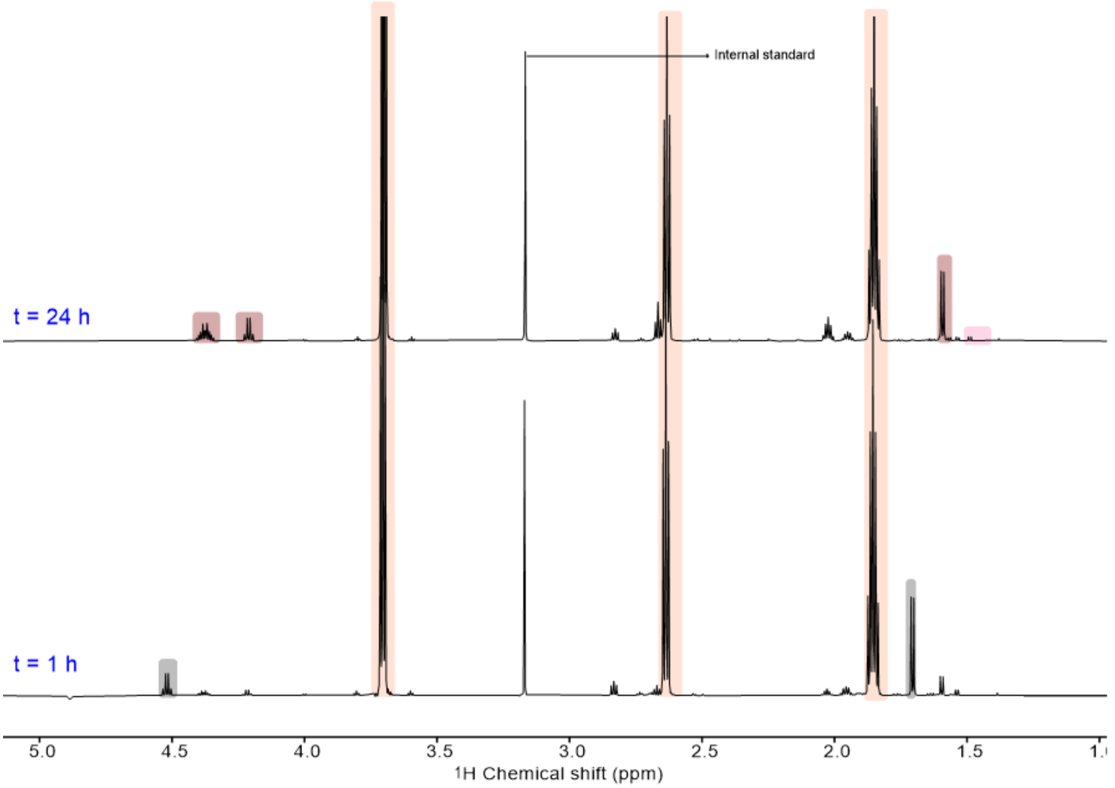

Supplementary Figure 21. *^{1}H NMR (600 MHz, H_2O/D_2O 9:1, noesygppr1d, 1.0–5.0 ppm) spectrum to show the reaction of alanine nitrile (**8** $_{\mathbf{Ala}}$, 200 mM) with 3-mercaptopropanol (**5f**, 1.8 M) in 1 M NaH_2PO_4 buffer, with MSM (100 mM) as an internal standard at pH 4 and room temperature. Set up following General Procedure A.*

^{1}H NMR (600 MHz, H_2O/D_2O 9:1) $\mathbf{14^f_{Ala}}$ (partial assignment) : δ_H 4.38 (2H, qt, *J* = 11.0, 6.1 Hz, Ala-α-CHCOOC*H*$_2$), 4.21 (1H, q, *J* = 7.3 Hz, Ala-α-C*H*COOCH$_2$), 2.67 (2H, t, *J* = 7.0 Hz, Ala-α-CHCOOCH$_2$CH$_2$C*H*$_2$SH), 2.03 (2H, p, *J* = 6.6 Hz, Ala-α-CHCOOCH$_2$C*H*$_2$CH$_2$SH), 1.60 (3H, d, *J* = 7.2 Hz, Ala-α-CH(C*H*$_3$)).

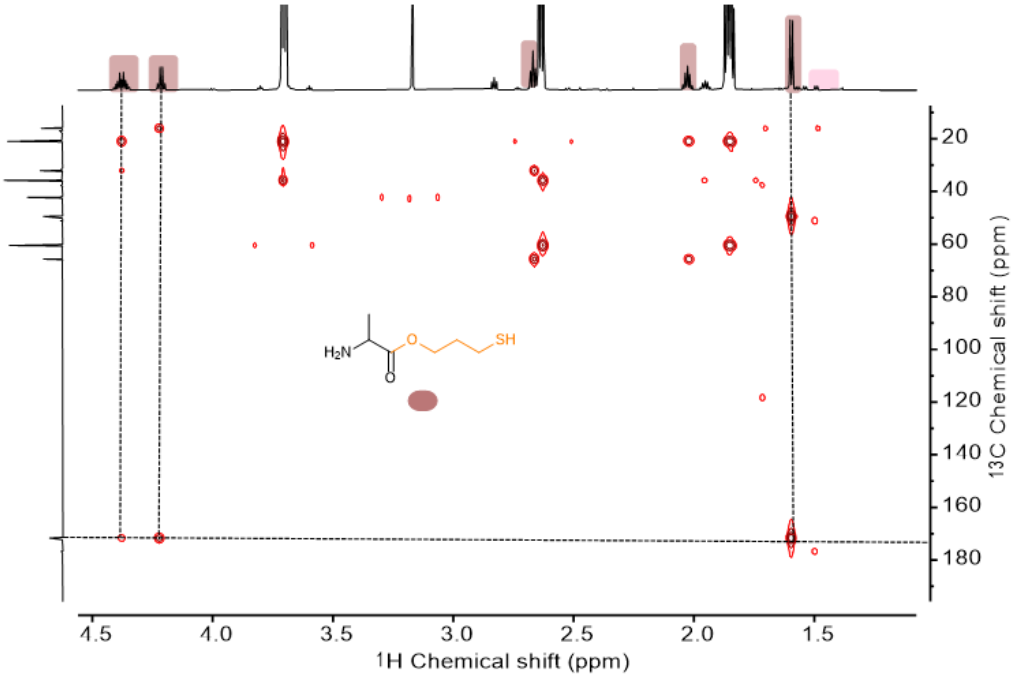

Supplementary Figure 22. ^{1}H–^{13}C *HMBC (*^{1}H*: 600 MHz [1.4 – 4.5 ppm],* ^{13}C*: 176 MHz [20 – 190 ppm],* H_2O/D_2O *9:1) spectrum showing the diagnostic* ^{2}JCH *and* ^{3}JCH *coupling of alanine-a-*$CHCOOCH_2$*, alanine-aH-*$COOCH_2$ *and alanine-a-*$CH(CH_3)COOCH_2$ *in* $\mathbf{14^{f}_{Ala}}$ *at 4.38, 4.21 ppm and 1.60 ppm with a resonance at 171.0 ppm, which is characteristic peak of aminoacyl ester bond formation.*

*Synthesis of α-aminoacyl ester **14**$_{Ser}$ from the reaction of serine nitrile **8**$_{Ser}$ with 3-mercaptopropanol **5f** at pH 4*

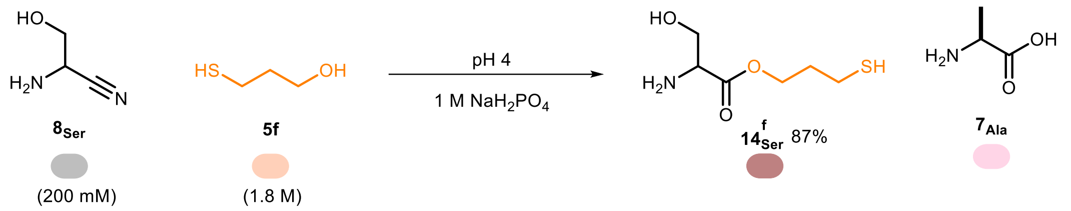

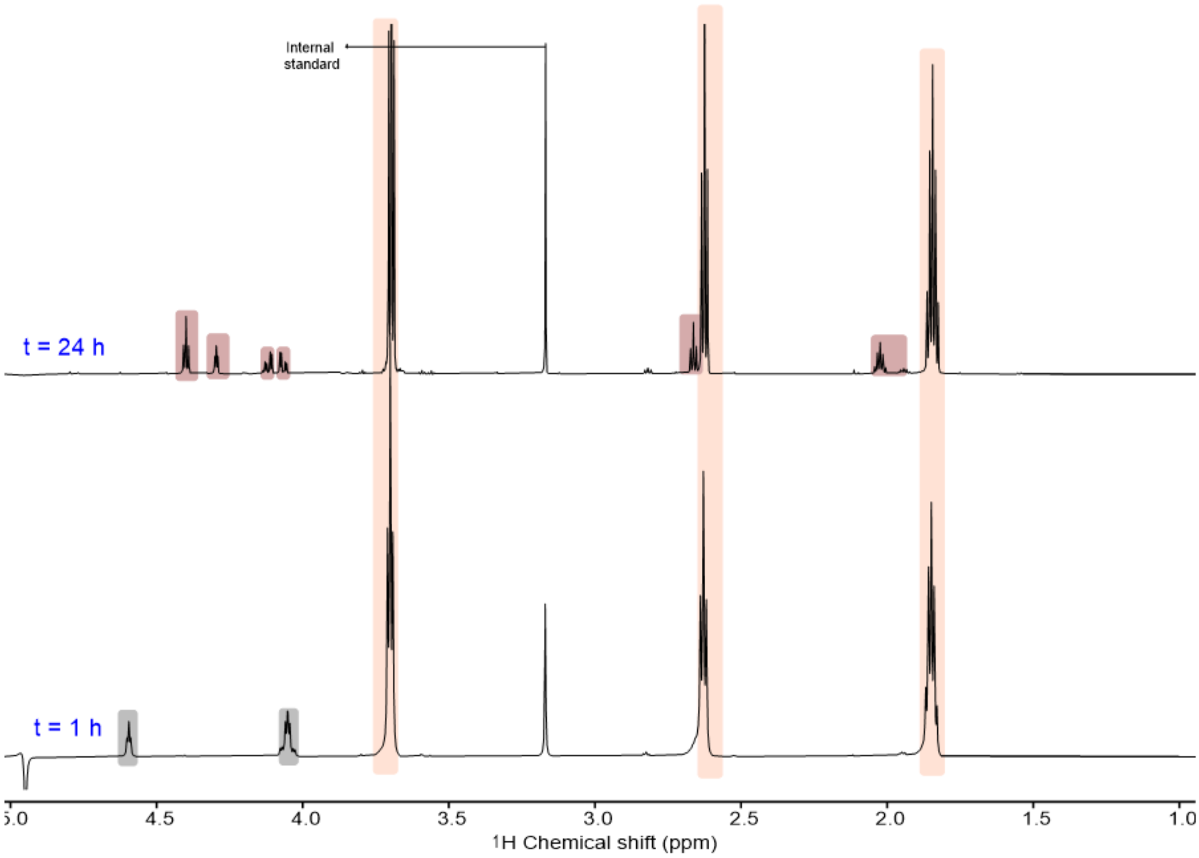

*Supplementary Figure 23. ¹H NMR (600 MHz, H_2O/D_2O 9:1, noesygppr1d, 1.0 – 5.0 ppm) spectrum to show the reaction of serine nitrile (**2**$_{Ser}$, 200 mM) with 3-mercaptopropanol (**3f**, 1.8 M) in 1M NaH_2PO_4 buffer, with MSM (100 mM) as an internal standard at pH 3 and room temperature. Set up following General Procedure A.*

¹H NMR (600 MHz, H_2O/D_2O 9:1) $\mathbf{14}^{f}_{Ser}$ (partial assignment) : δ_H 4.21 (2H, t, Ser-α-CHCOOC*H*$_2$), 4.11 (1H, d, Ser-α-C*H*COOCH$_2$), 3.93 (1H, dd, *J* = 12.5, 4.2 Hz, Ser-α-CHC*H*HOH), 3.88 (1H, dd, *J* = 12.5, 3.5 Hz, Ser-α-CHCH*H*OH), 2.48 (2H, t, *J* = 7.0 Hz, Ser-α-CHCOOCH$_2$CH$_2$C*H*$_2$SH), 2.48 (2H, t, *J* = 7.0 Hz, Ser-α-CHCOOCH$_2$CH$_2$C*H*$_2$SH), 1.88 – 1.80 (m, Ser-α-CHCOOCH$_2$C*H*$_2$CH$_2$SH).

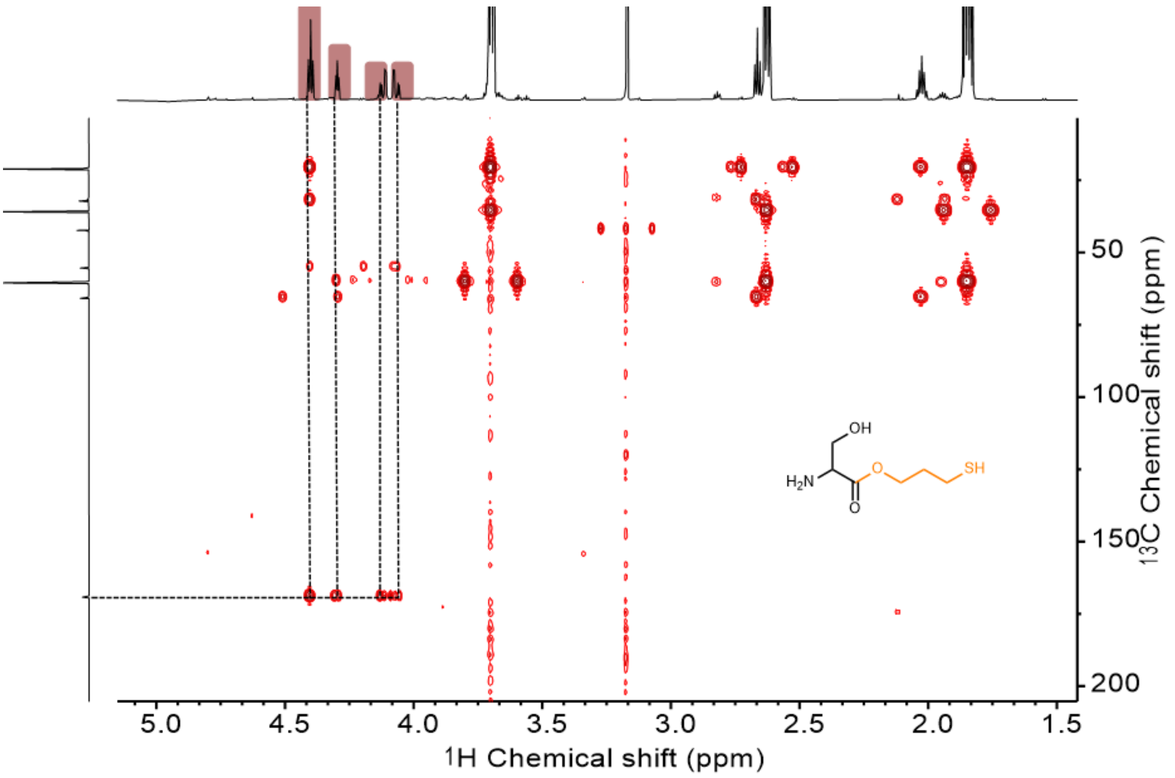

Supplementary Figure 24. ^{1}H–^{13}C *HMBC* (^{1}H: *600 MHz [1.5 – 5.0 ppm],* ^{13}C*: 176 MHz [0 – 200 ppm],* H_2O/D_2O *9:1) spectrum showing the diagnostic* ^{2}JCH *and* ^{3}JCH *coupling of Ser-aCHCOOCH*$_2$*, Ser-aH-COOCH*$_2$ *and Ser-aCHCH*$_2$*COOCH*$_2$ *in* $\mathbf{14^{f}_{Ser}}$ *at 4.40, 4.30 ppm and 4.12, 4.07 ppm with a resonance at 170.8 ppm, which is characteristic of aminoacyl ester bond formation.*

*Synthesis of α-aminoacyl ester **14**$_{Pro}$ from the reaction of proline nitrile **8**$_{Pro}$ with 3-mercaptopropanol **5f** at pH 4*

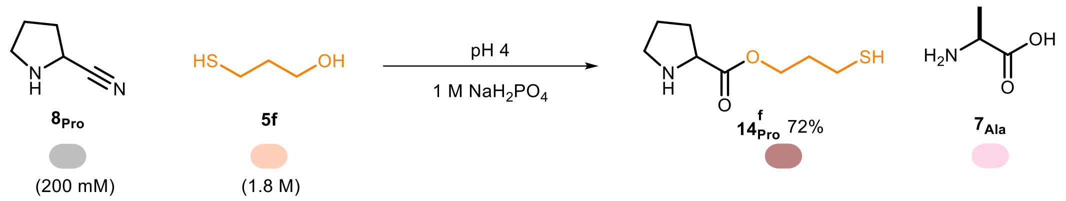

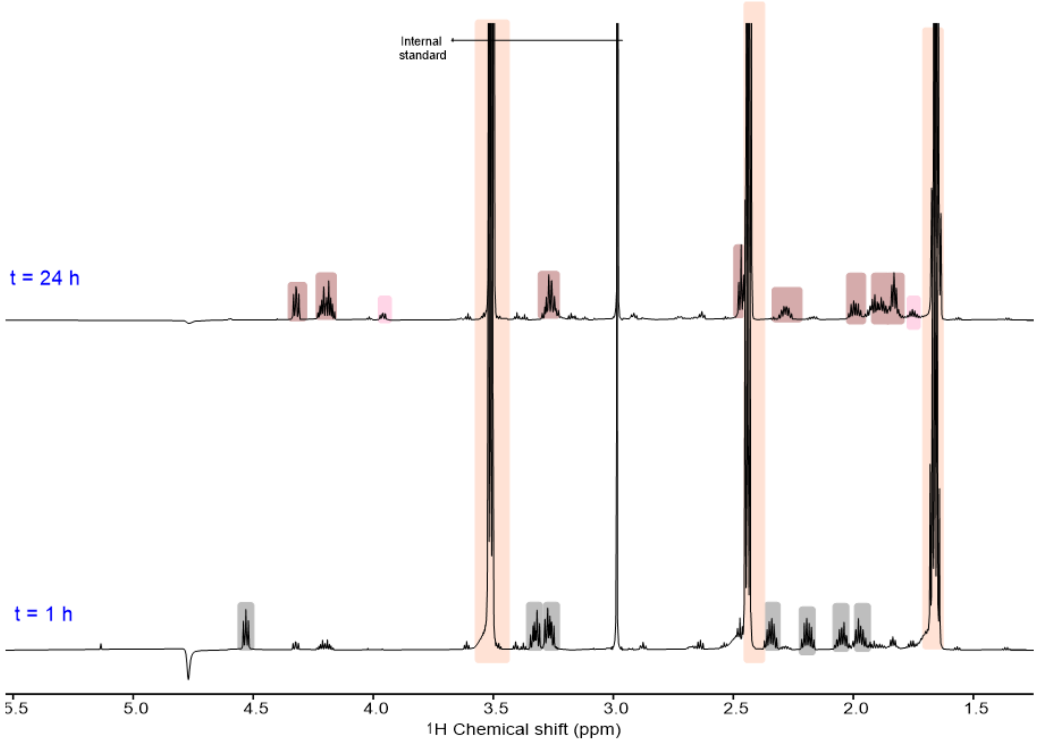

*Supplementary Figure 25. ^{1}H NMR (600 MHz, H_2O/D_2O 9:1, noesygppr1d, 1.5 – 5.5 ppm) spectrum to show the reaction of proline nitrile (**8**$_{Pro}$, 200 mM) with 3-mercaptopropanol (**5f**, 1.8 M) in 1 M NaH_2PO_4 buffer, with MSM (100 mM) as an internal standard at pH 3 and room temperature. Set up following General Procedure A.*

^{1}H NMR (600 MHz, H_2O/D_2O 9:1) **14**$^{\mathbf{f}}_{\mathbf{Pro}}$ (partial assignment) : δ_H 4.51 (1H, dd, J = 8.8, 6.9 Hz, Pro-α-C*H*COOCH$_2$), 4.43 – 4.29 (2H, m, Pro-α-CHCOOC*H*$_2$), 3.53 – 3.38 (2H, m, J = 12.5, 4.2 Hz, Pro-NHC*H*$_2$CH$_2$CH$_2$CHCOO), 2.51 – 2.42 (2H, m, Pro-α-CHCOOCH$_2$CH$_2$C*H*$_2$SH), 2.30 – 2.15 (2H, m, Pro-NHCH$_2$CH$_2$C*H*HCHCOO), 2.12 – 2.00 (2H, m, Pro-NHCH$_2$CH$_2$CH*H*CHCOO.

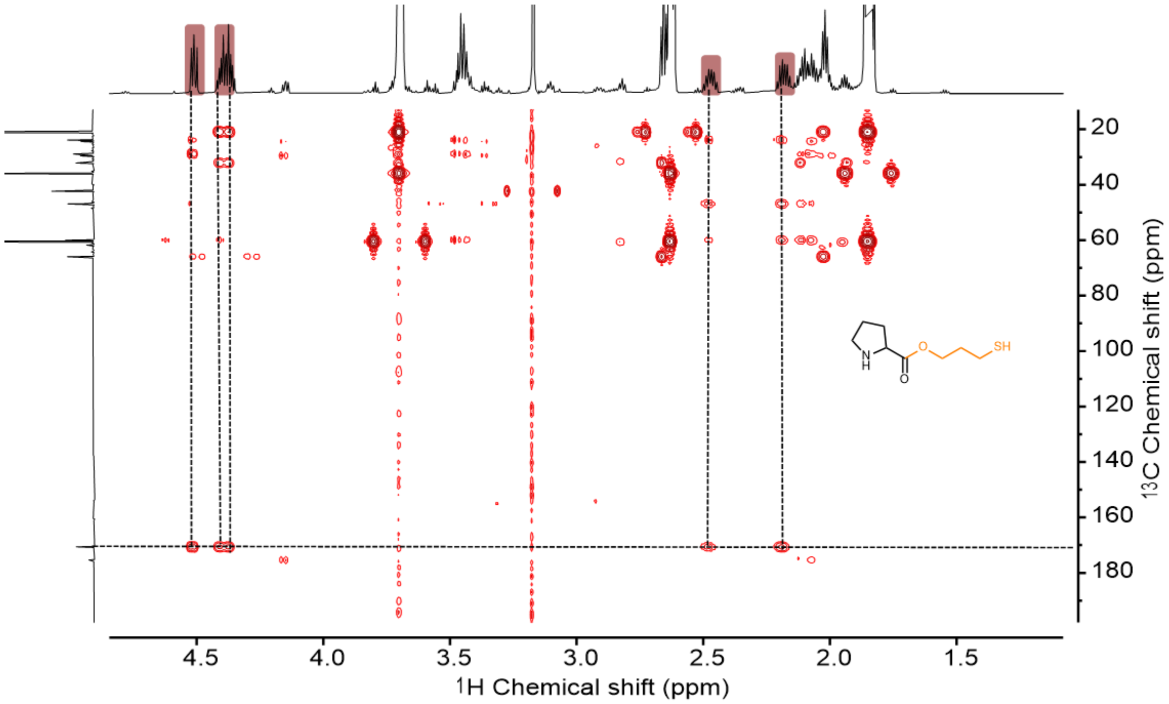

Supplementary Figure 26. ^{1}H–^{13}C *HMBC (*^{1}H*: 600 MHz [2.2-4.5 ppm],* ^{13}C*: 176 MHz [155-200 ppm],* H_2O/D_2O *9:1) spectrum showing the diagnostic* ^{2}JCH *and* ^{3}JCH *coupling of Pro-aCH, Pro-aH-*$COOCH_2$ *and Pro-a*$CH_2CHCOOCH_2$ *in* $\mathbf{14^{f}_{Pro}}$ *at 4.51, 4.38 ppm and 2.47, 2.18 ppm with a resonance at 170.4 ppm, which is characteristic of aminoacyl ester bond formation.*

Synthesis of α-amino thioacids **12_{Aaa}** from α-amino thioesters **1_{Aaa}**

*General Procedure B: Synthesis of α-amino thioacids **12_{Aaa}** from α-amino thioesters **1_{Aaa}***

α-Amino thioester **1^e_{Aaa}** (0.05 mmol) and methylsulfonylmethane (MSM, 0.01 mmol) or pentaerythritol (PET, 0.005 mmol) were dissolved in degassed H_2O/D_2O (98:2, 0.5 mL) and adjusted to pH 5 with NaOH/HCl. Sodium hydrogen sulfide (0.1 – 0.2 mmol) was added to the acidic solution under a flow of argon gas (**CAUTION:** This reaction generates volatile (toxic) H_2S. The reaction was performed with a very high fumehood ventilation flowrate and reaction outgas bubbled through an alkaline bleach quenching solution). Following addition of sulfide, the solution was measured as between pH 8-9. NMR spectra were obtained directly and **1^e_{Aaa}** formation was confirmed NMR spectroscopy and quantified against MSM or PET. No further attempts were made optimise sub-quantitative reaction yields.

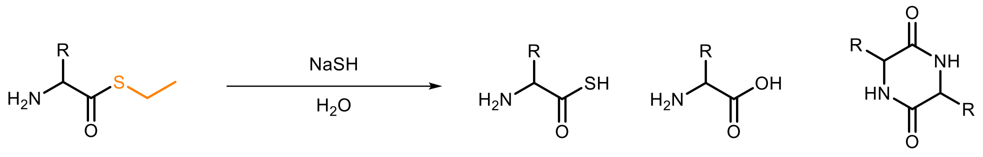

Entry	**1^e_{Aaa}**	Time (minutes)	**12_{Aaa}** (%)	**7_{Aaa}** (%)
1	Gly	10	>95	n.d.
2	L-Ala	30	>95	3
3	L-Pro	40	82	18
4	L-Val	60	>95	3
5	L-Leu	60	88	8
6	L-Lys	20	>95	4
7	L-Phe	30	70	23
8	L-Glu	10	91	6
9	L-Arg	10	90	obs.
10	DL-Ser	10	>95	n.d.

*Supplementary Table 4. Yield (%) for the formation a-amino thioacids **12_{Aaa}** from the reaction of a-amino thioester **1^e_{Aaa}** (100 mM) upon reaction with NaSH (200-400 mM). Set up following General Procedure B. obs. = obscured by signal overlap.*

Synthesis of glycine thioacid ***12**$_{Gly}$* *from glycine thioester* ***1**$_{Gly}$*

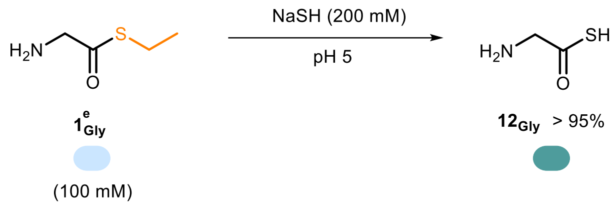

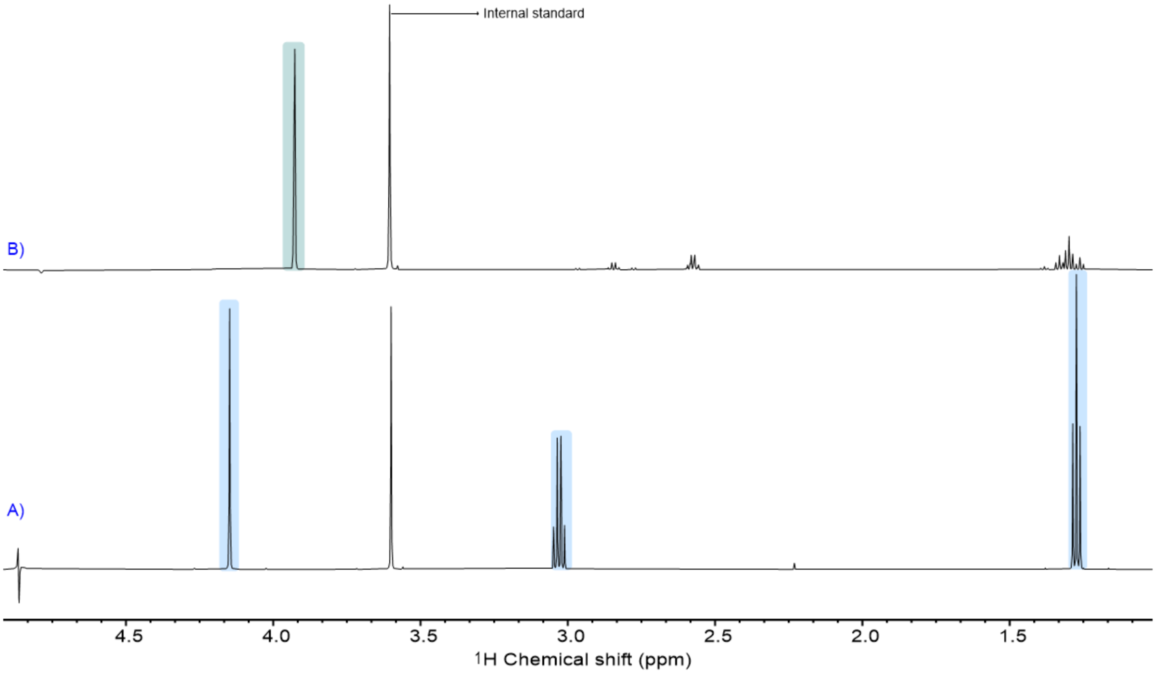

*Supplementary Figure 27. ^{1}H NMR (600 MHz, H_2O/D_2O 9:1, noesygppr1d, 1.0 – 5.0 ppm) spectra to show the reaction of glycine thioester (**1^e_{Gly}**, 100 mM) with NaSH (200 mM) at pH 5, using PET (10 mM) as an internal standard. Set up following General Procedure B A) **1^e_{Gly}**; B) **12_{Gly}** formed from the reaction of **1^e_{Gly}** with NaSH [final pH = 8.3].*

^{1}H NMR (600 MHz, H_2O/D_2O 9:1) **12_{Gly}** (partial assignment): δ_H 3.84 (2H, s, Gly-α-CH_2COSH).

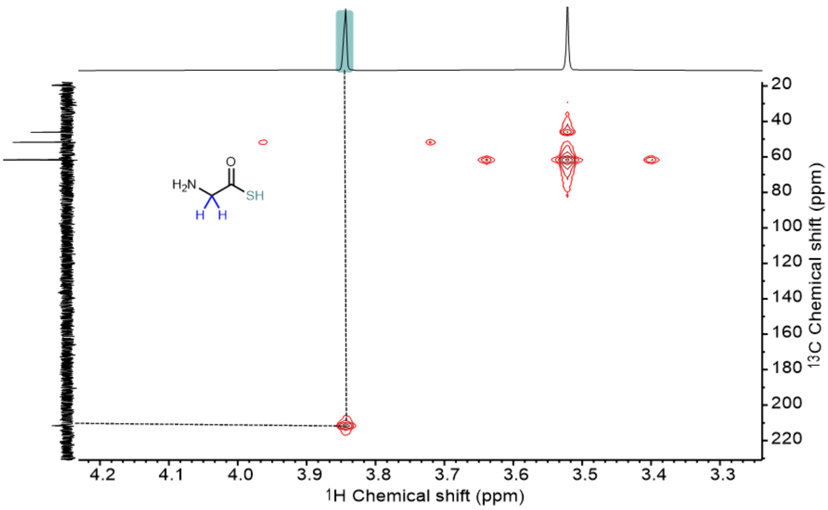

*Supplementary Figure 28. ^{1}H–^{13}C HMBC ([1]H: 600 MHz [3.1 – 4.2 ppm], [13]C: 176 MHz [20 – 225 ppm], H_2O/D_2O 9:1) spectrum showing the diagnostic ^2JCH of glycinyl-aH in **12_{Gly}** at 3.84 ppm with a resonance at 213.3 ppm, which is characteristic of amino thioacid bond formation.*

Synthesis of alanine thioacid ***12*****$_{Ala}$** *from L-alanine thioester* ***1*****$_{Ala}$**

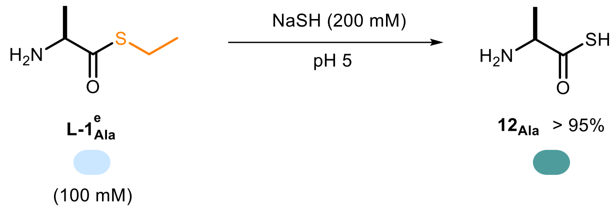

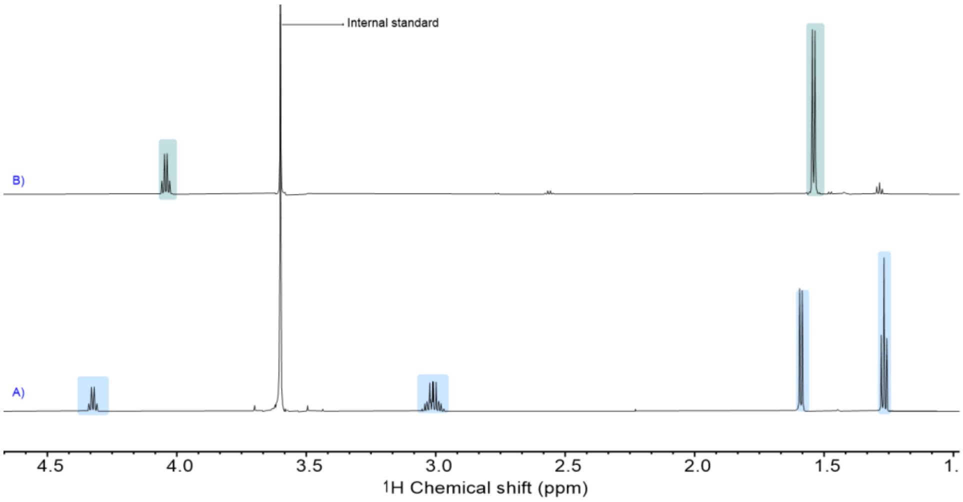

*Supplementary Figure 29. ^{1}H NMR (700 MHz, H_2O/D_2O 9:1, noesygppr1d, 1.0–4.7 ppm) spectrum shows the reaction of L-alanine thioester (**1^e_{Ala}**, 100 mM) with NaSH (200 mM) at pH 5, using PET (10 mM) as an internal standard. Set up following General Procedure B. A) **1^e_{Ala}**; B) **12_{Ala}** formed from the reaction of **1^e_{Ala}** with NaSH [final pH = 8.3].*

^{1}H NMR (700 MHz, H_2O/D_2O 9:1) **12_{Ala}** (partial assignment): δ_H 4.04 (1H, q, J = 7.1 Hz, Ala-α-C*H*COSH), 1.54 (3H, d, J = 7.1 Hz, Ala-α-CHC*H*$_3$).

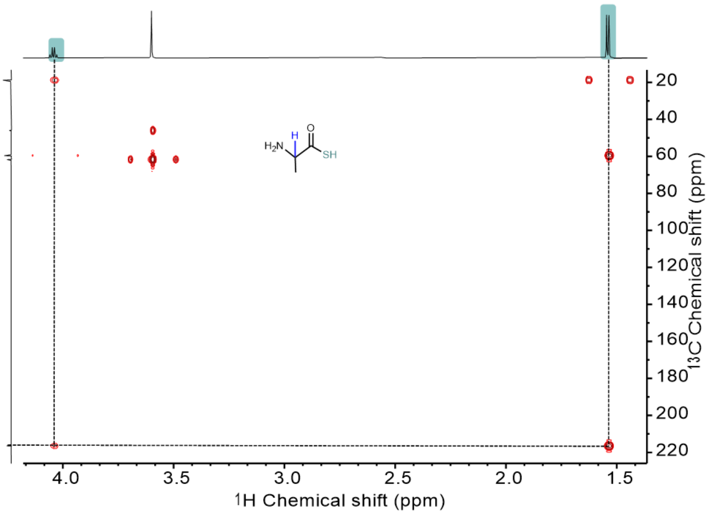

*Supplementary Figure 30. ^{1}H–^{13}C HMBC (^{1}H: 600 MHz [0.8-4.2 ppm], ^{13}C: 176 MHz [15-225 ppm], H_2O/D_2O 9:1) spectrum showing the diagnostic ^2JCH and ^3JCH coupling of alaninyl-aH and -CHCH$_3$ in **12_{Ala}** at 4.04 ppm and 1.53 ppm with a resonance at 216.5 ppm, which is characteristic of amino thioacid formation.*

Synthesis of leucine thioacid $\mathbf{12_{Leu}}$ *from* L-*leucine thioester* $\mathbf{1_{Leu}}$

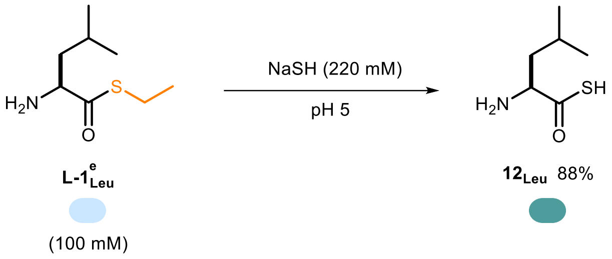

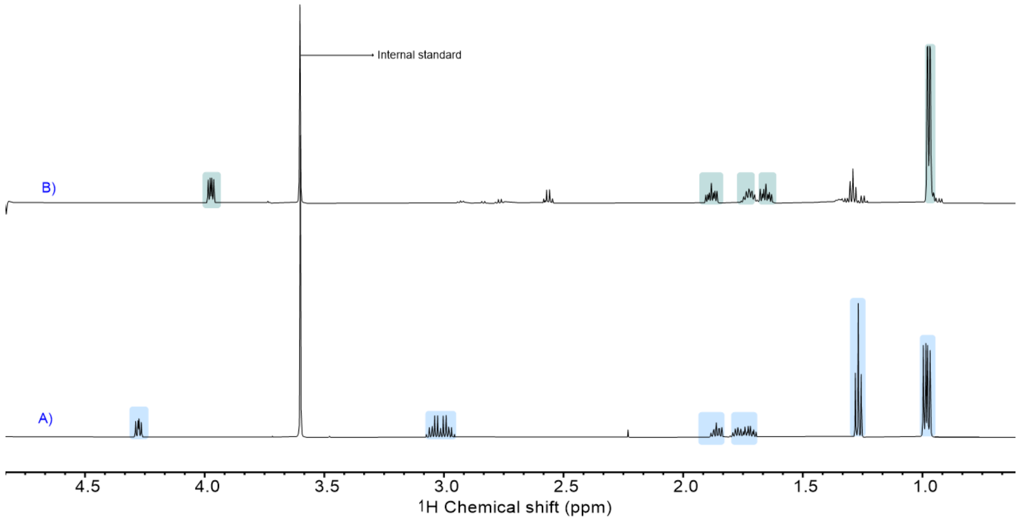

Supplementary Figure 31. [1]*H NMR (600 MHz,* H_2O/D_2O *9:1, noesygppr1d, 0.5 – 4.7 ppm) spectra to show the reaction of* L*-leucine thioester (*L*-*$\mathbf{1}^{e}_{Leu}$*, 100 mM) with NaSH (220 mM) at pH 5, using PET (10 mM) as an internal standard. Set up following General Procedure* B*. A)* $\mathbf{1}^{e}_{Leu}$*; B)* $\mathbf{12_{Leu}}$ *formed from the reaction of* $\mathbf{1}^{e}_{Leu}$ *with NaSH [final pH = 8.0].*

^{1}H NMR (600 MHz, H_2O/D_2O 9:1) $\mathbf{12_{Leu}}$ (partial assignment): δ_H 3.97 (1H, dd, *J* = 9.0, 5.1 Hz, Leu-α-C*H*COSH), 1.88 (1H, ddd, *J* = 14.0, 8.8, 5.1 Hz, Leu-α-CHC*H*HCH$_2$), 1.80 – 1.69 (1H, m, Leu-α-CHC*H*HCH$_2$), 1.65 (1H, ddd, *J* = 14.0, 8.9, 5.0 Hz, Leu-α-CHCH*H*CH$_2$), 0.97 (6H, dd, *J* = 6.5, 1.7 Hz, -CH$_2$(C*H*$_3$)$_2$).

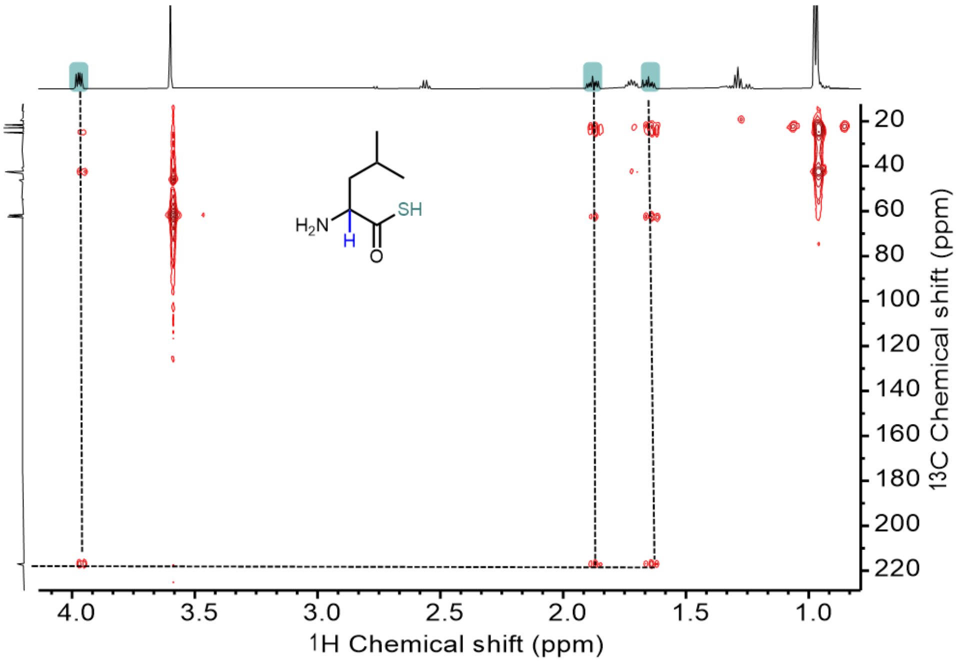

Supplementary Figure 32. ^1H–^{13}C HMBC (^1H: 600 MHz [0.8 – 4.2 ppm], ^{13}C: 176 MHz [15 – 225 ppm], H_2O/D_2O 9:1) spectrum showing the diagnostic ^2JCH and ^3JCH coupling of Leucine-aH-COSH and -COCHCH$_2$ in ***12$_{Leu}$*** *at 3.95 ppm, 1.87 ppm and 1.63 ppm with a resonance at 217.3 ppm, which is characteristic of amino thioacid formation.*

*Synthesis of arginine thioacid **12**$_{Arg}$ from arginine thioester **1**$_{Arg}$*

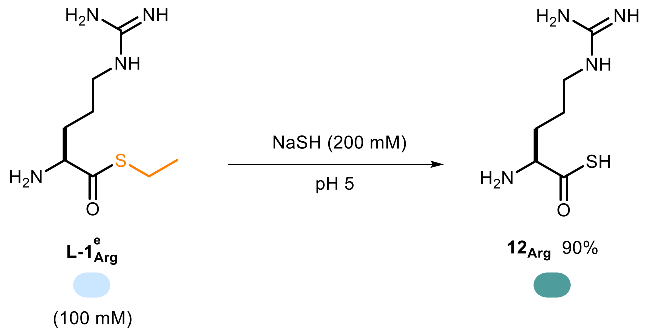

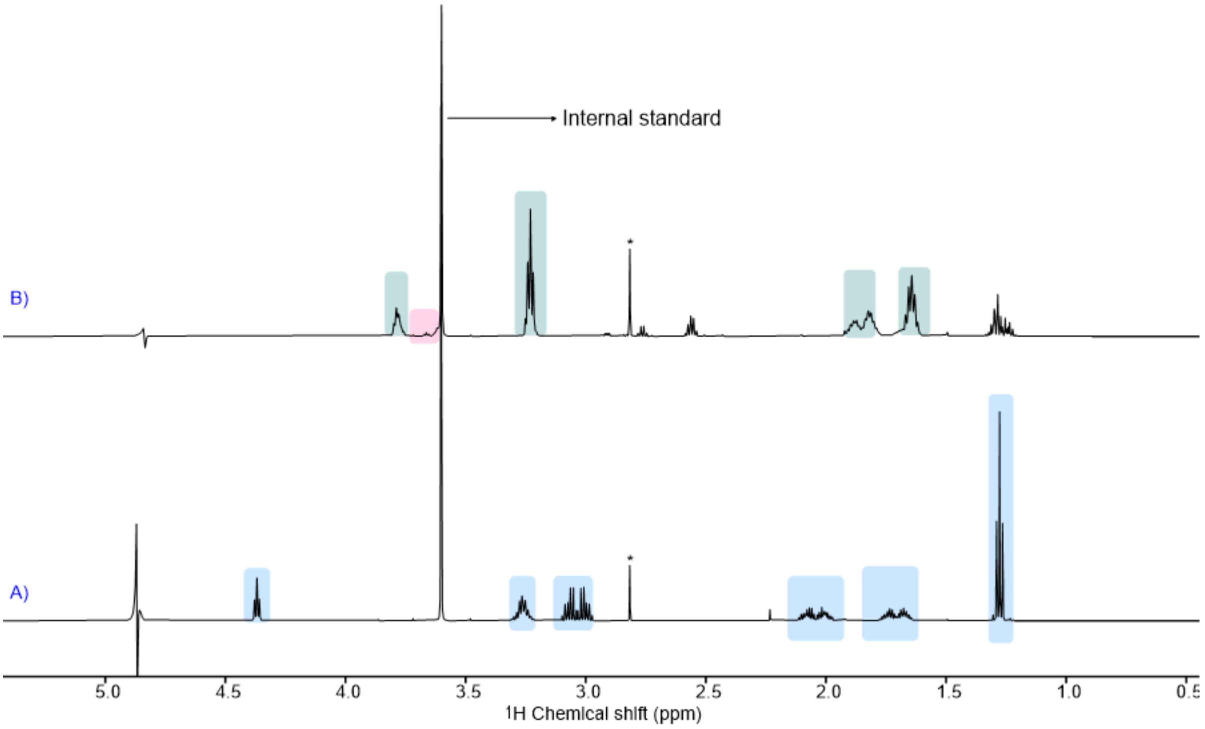

Supplementary Figure 33. ^{1}*H NMR (600 MHz, H_2O/D_2O 9:1, noesygppr1d, 0.5 – 5.0 ppm) spectra to show the reaction of L-arginine thioester (L-**1**$^{e}_{Arg}$, 100 mM) with NaSH (200 mM) at pH 5, using PET (10 mM) as an internal standard. Set up following General Procedure* B. *A) **1**$^{e}_{Arg}$ with PET (10 mM) as an internal standard; B) **12**$_{Arg}$ formed from the reaction of **1**$^{e}_{Arg}$ with NaSH [final pH = 8.0].*Tetramethyl urea from the synthesis of L-**1**$^{e}_{Arg}$.*

^{1}H NMR (600 MHz, H_2O/D_2O 9:1) **12**$_{Arg}$ (partial assignment): δ_H 3.65 (1H, m, Arg-α-C*H*COSH), 3.10 – 3.21 (2H, m, CH_2CH_2NH-guanidine); 1.83 – 1.60 (2H, m, α-$CHCH_2CH_2CH_2$ NH), 1.51 (2H, p, *J* = 6.5 Hz, α-CH $CH_2CH_2CH_2$NH).

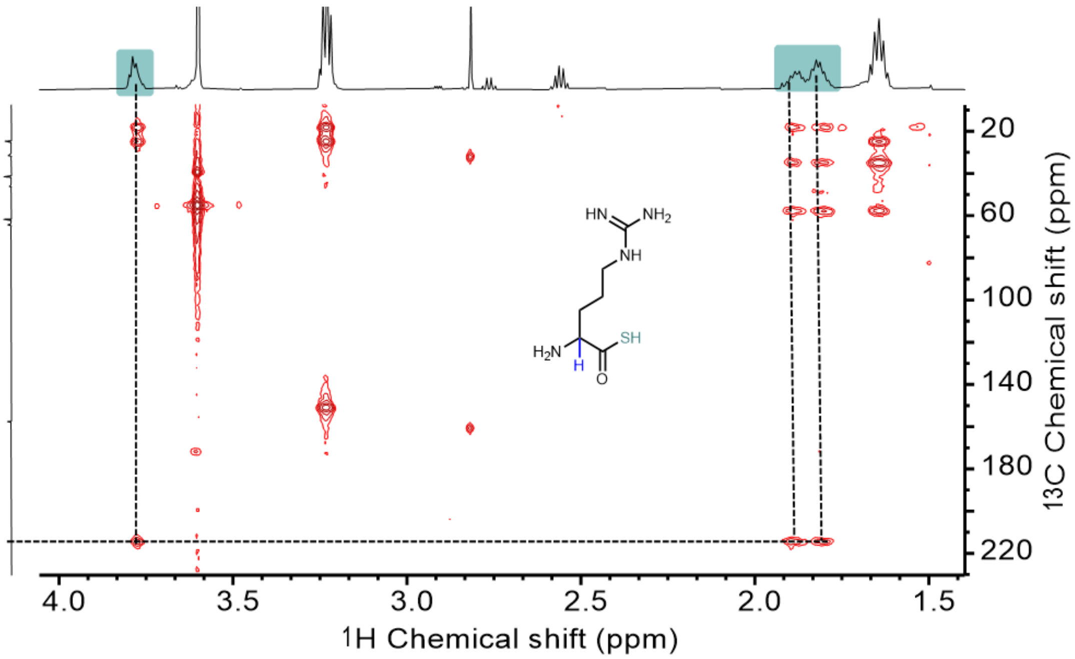

Supplementary Figure 34. *^{1}H–^{13}C HMBC (^{1}H: 600 MHz [1.5 – 4.0 ppm], ^{13}C: 176 MHz [10 – 220 ppm], H_2O/D_2O 9:1) spectrum showing the diagnostic ^{2}JCH and ^{3}JCH coupling of Argininyl-aH and -CH_2 in* ***12_{Arg}*** *at 3.77 ppm, 1.80 ppm and 1.89 ppm with a resonance at 214.4 ppm, which is characteristic of amino thioacid formation.*

*Synthesis of proline thioacid **12_{Pro}** from L-proline thioester **1_{Pro}***

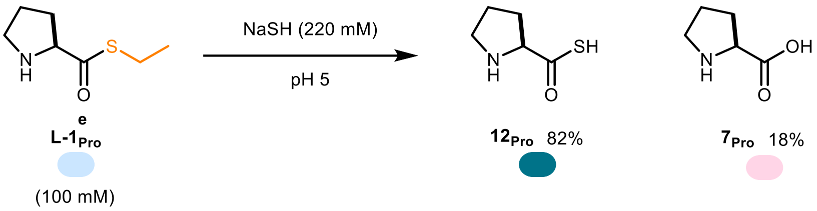

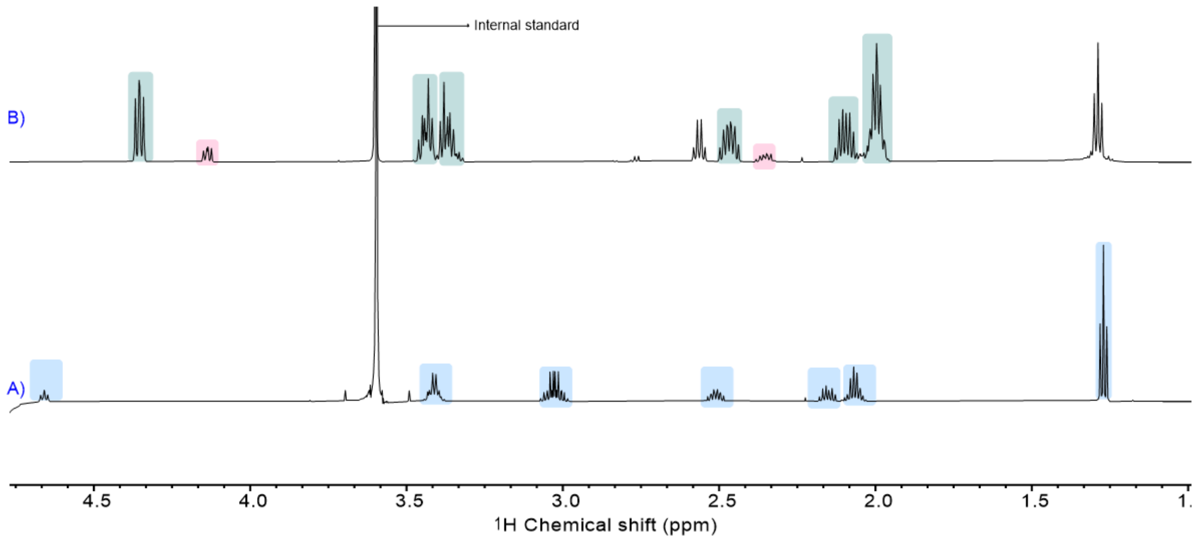

*Supplementary Figure 35. ¹H NMR (600 MHz, H_2O/D_2O 9:1, noesygppr1d, 1.0 – 4.7 ppm) spectra to show the reaction of L-proline thioester) (L-**1^e_{Pro}**, 100 mM) with NaSH (220 mM) at pH 5, using PET (10 mM) as an internal standard. Set up following General Procedure B. A) **1^e_{Pro}**; B) **12_{Pro}** formed from the reaction of **1^e_{Pro}** with NaSH [final pH = 8.2].*

^{1}H NMR (600 MHz, H_2O/D_2O 9:1) **12_{Pro}** (partial assignment) : δ_H 4.35 (1H, dd, *J* = 8.7, 6.9 Hz, Pro-α-C*H*COSH), 3.44 (1H, app. dt, *J* = 11.5, 7.0 Hz, Pro-α-CHNHC*H*H); 3.37 (1H, dt, *J* = 11.5, 7.0 Hz, Pro-α-CHNHCH*H*); 2.47 (1H, app. ddt, *J* = 13.4, 8.7, 6.8 Hz, α-CHCH*H*CH_2CH_2NH), 2.22 – 2.05 (1H, m, α-CHC*H*HCH_2CH_2NH, 1.99 (2H, m, α-CH CH_2C*H_2*CH_2NH).

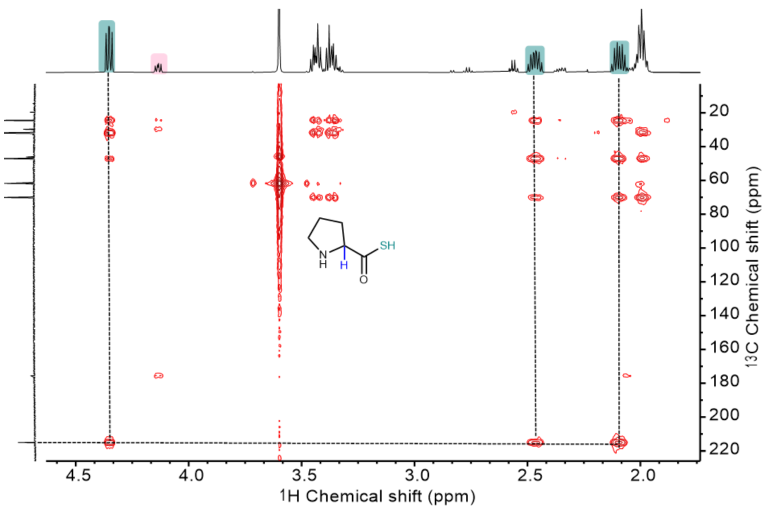

*Supplementary Figure 36. ^{1}H–^{13}C HMBC (^{1}H: 600 MHz [1.5-4.5 ppm], ^{13}C: 176 MHz [20-220 ppm], H_2O/D_2O 9:1) spectrum showing the diagnostic ^{2}JCH and ^{3}JCH coupling of both Proline-aH and $CHCH_2$ in **12_{Pro}** at 4.35 ppm, 2.47 and 2.09 ppm with a resonance at 215.0 ppm, which is characteristic of amino thioacid formation.*

Synthesis of lysine thioacid ***12$_{Lys}$*** *from* L*-lysine thioester* ***1$_{Lys}$***

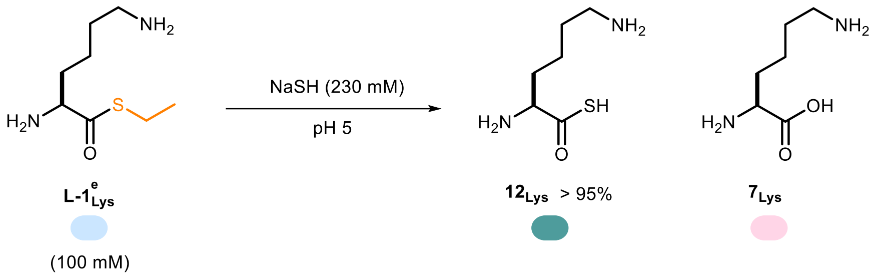

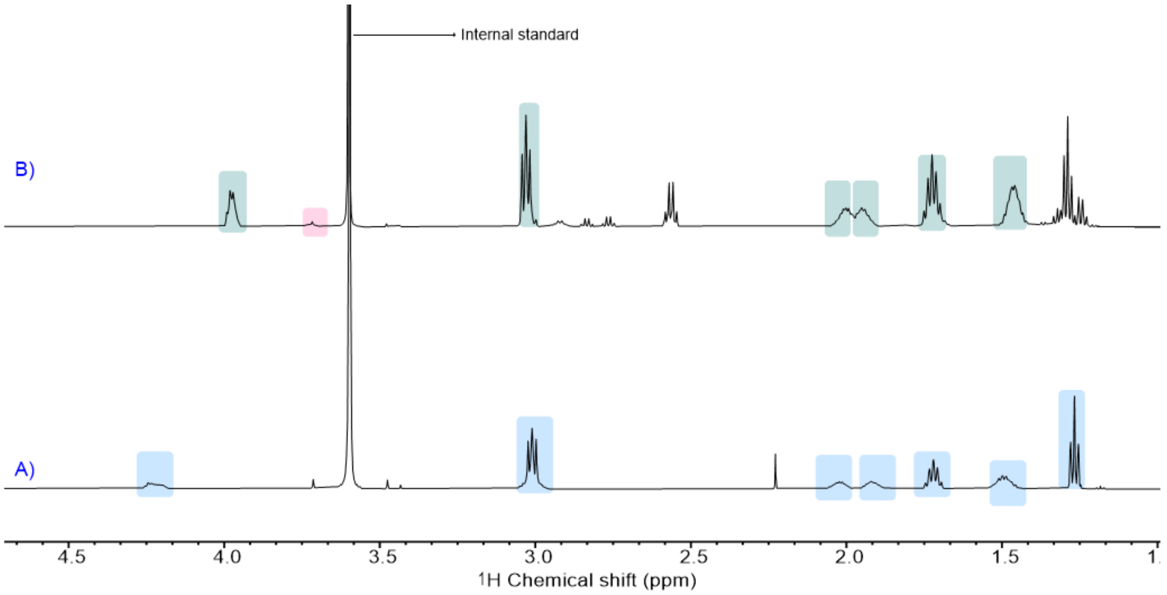

Supplementary Figure 37. *^{1}H NMR (600 MHz, H_2O/D_2O 9:1, noesygppr1d, 1.0–4.6 ppm) spectra to show the reaction of* L*-lysine thioester (*L*-**1^e_{Lys}**, 100 mM) with NaSH (230 mM) at pH 5, using PET (10 mM) as an internal standard. Set up following General Procedure B. A) **1^e_{Lys}**; B) **12$_{Lys}$** formed from the reaction of **1^e_{Lys}** with NaSH [final pH = 8.2].*

^{1}H NMR (600 MHz, H_2O/D_2O 9:1) **12$_{Lys}$** (partial assignment) : δ_H 4.11 – 3.89 (1H, m, Lys-α-C*H*COSH), 3.03 (2H, t, *J* = 7.7 Hz, Lys-ε-C*H*$_2$NH$_2$); 2.07 – 1.89 (2H, m, Lys-α-CHC*H*$_2$CH$_2$), 1.73 (2H, m, α-CHCH$_2$CH$_2$C*H*$_2$CH$_2$NH), 1.60 – 1.39 (2H, m, α-CHCH$_2$C*H*$_2$CH$_2$CH$_2$NH).

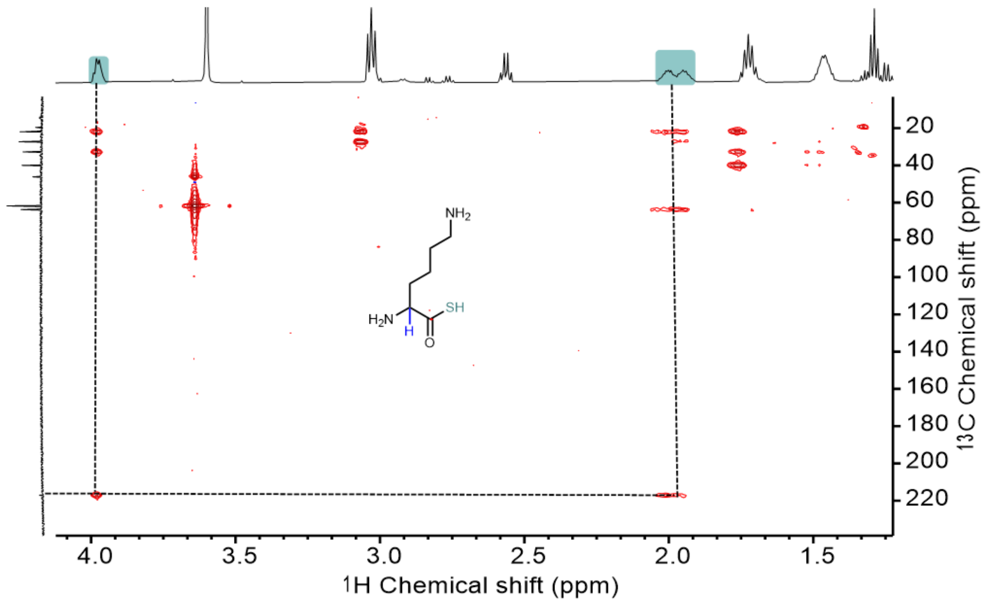

*Supplementary Figure 38. ^{1}H–^{13}C HMBC (^{1}H: 600 MHz [1.2 – 4.2 ppm], ^{13}C: 176 MHz [15 – 225 ppm], H_2O/D_2O 9:1) spectrum showing the diagnostic ^{2}JCH and ^{3}JCH coupling of Lysine-aH and – aCHCH$_2$ in **12$_{Lys}$** at 3.98 ppm and 1.97 ppm with a resonance at 216.9 ppm, which is characteristic of amino thioacid formation.*

Synthesis of valine thioacid ***12****$_{Val}$* *from L-valine thioester* ***1****$_{Val}$*

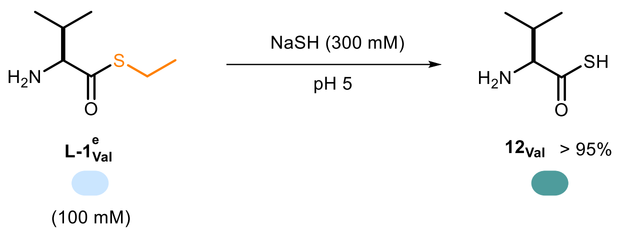

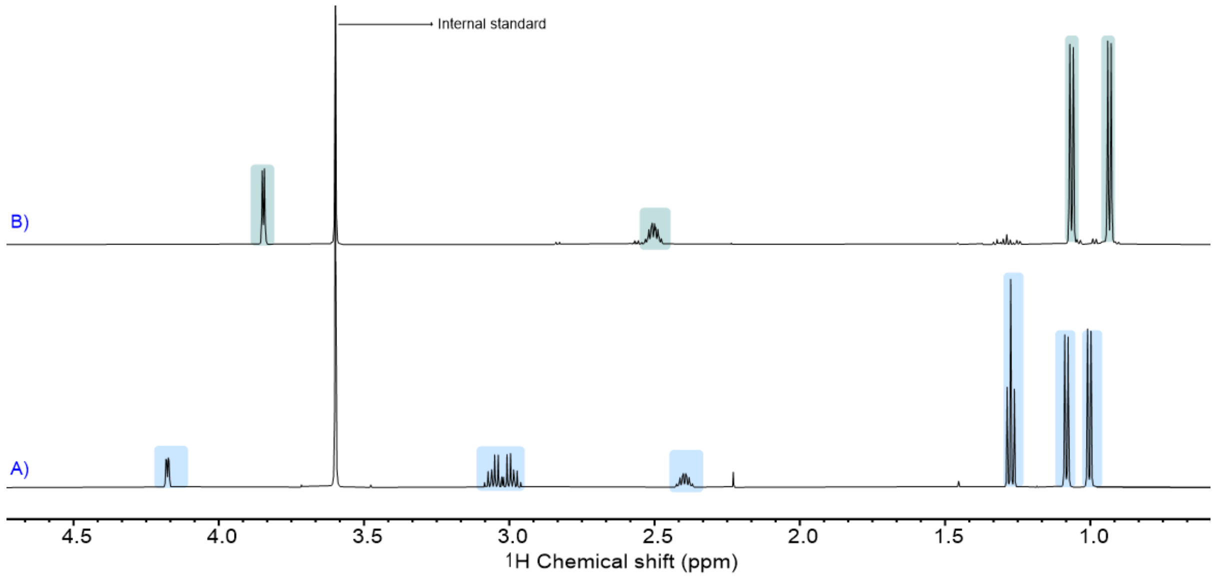

*Supplementary Figure 39. ^{1}H NMR (600 MHz, H_2O/D_2O 9:1, noesygppr1d, 0.5 – 4.7 ppm) spectra to show the reaction of L-valine thioester (L-**1**$^{e}_{Val}$, 100 mM) with NaSH (300 mM) at pH 5, using PET (10 mM) as an internal standard. Set up following General Procedure B. A) **1**$^{e}_{Val}$; B) **12**$_{Val}$ formed from the reaction of **1**$^{e}_{Val}$ with NaSH [final pH = 8.0].*

^{1}H NMR (600 MHz, H_2O/D_2O 9:1) **12**$_{Val}$ (partial assignment): δ_H 3.85 (1H, d, *J* = 4.7 Hz, Val-α-C*H*COSH), 2.51 (1H, m, α-CHC*H*(CH_3)$_2$), 1.07 (3H, d, *J* = 7.0 Hz, α-CHCH C*H*$_3$CH$_3$), 0.94 (3H, d, *J* = 7.0 Hz, α-CHCHCH$_3$C*H*$_3$).

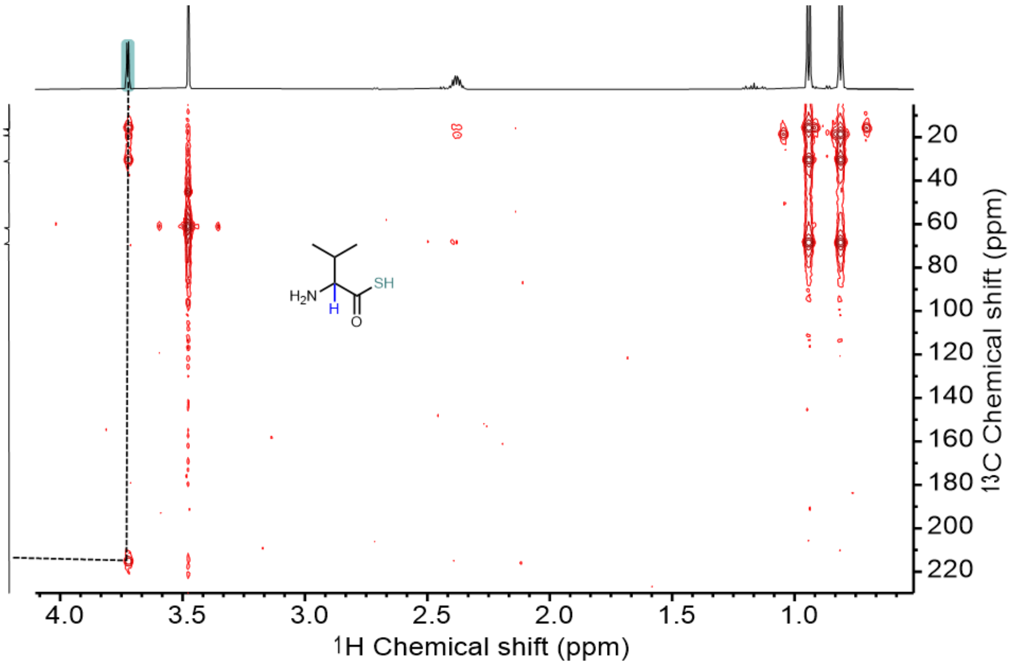

Supplementary Figure 40. ^{1}H–^{13}C HMBC (^{1}H: 600 MHz [0.5 – 4.0 ppm], ^{13}C: 176 MHz [20 – 220 ppm], H_2O/D_2O 9:1) spectrum showing the diagnostic ^{2}JCH coupling of Val-aH in ***12_{Val}*** *at 3.73 ppm with a resonance at 214.1 ppm, which is characteristic of amino thioacid formation.*

Synthesis of phenylalanine thioacid ***12*****$_{Phe}$** *from L-phenylalanine thioester* ***1*****$_{Phe}$**

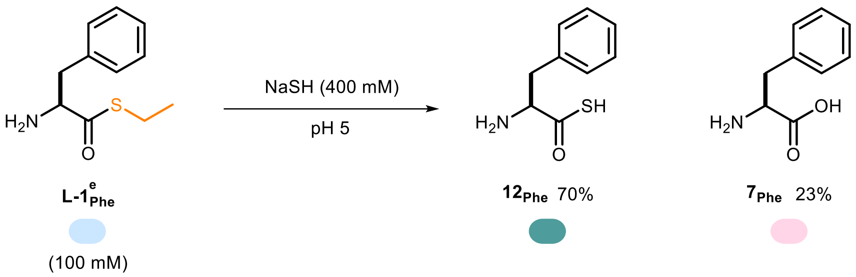

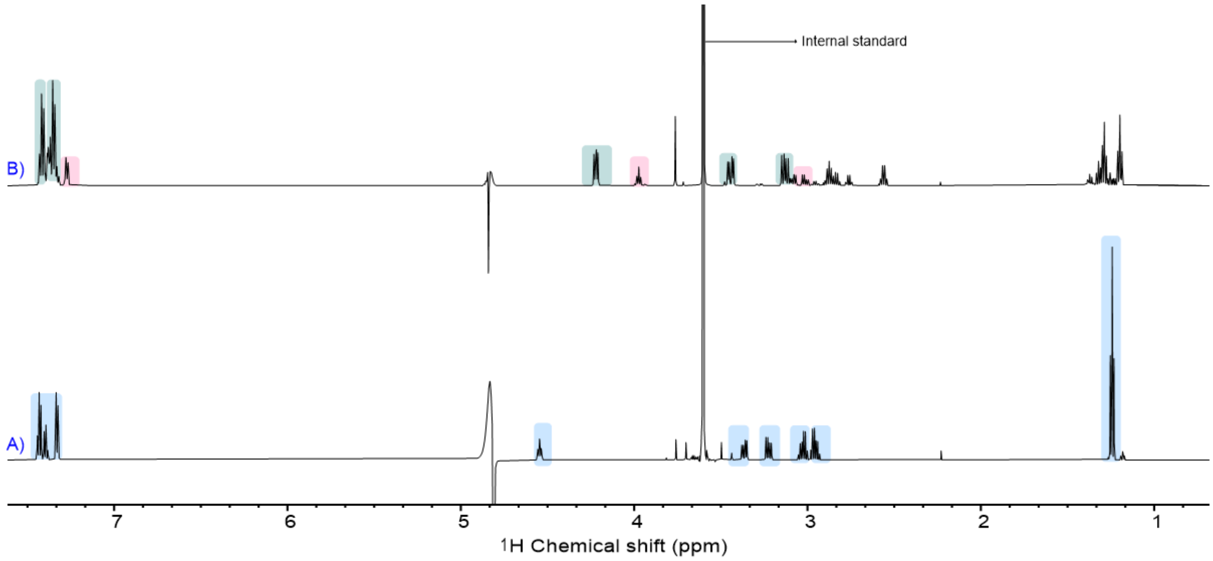

*Supplementary Figure 41. ^{1}H NMR (600 MHz, H_2O/D_2O 9:1, noesygppr1d, 1.0 – 7.8 ppm) spectrum shows the reaction of L-phenylalanine thioester (L-**1^{e}_{Phe}**, 100 mM) with NaSH (400 mM), using PET (10 mM) as an internal standard. Set up following General Procedure B. A) **1^{e}_{Phe}**; B) **12_{Phe}** formed from the reaction of **1^{e}_{Phe}** with NaSH [final pH = 8.0].*

^{1}H NMR (600 MHz, H_2O/D_2O 9:1) **12_{Phe}** (partial assignment): δ_H 7.47 – 7.31 (5H, m, Phe-α-C*H*CH$_2$C$_6$*H*$_5$), 4.22 (1H, ABX, J_{AX} = 8.2, J_{BX} = 5.3 Hz, Phe-α-C*H*COSH), 3.44 (1H, ABX, J_{AX} = 14.4, J_{BX} = 5.3 Hz, α-CHC*H*HC$_6$H$_5$), 3.13 (1H, ABX, J_{AX} = 14.4, J_{BX} = 8.3 Hz, α-CHCH*H*C$_6$H$_5$).

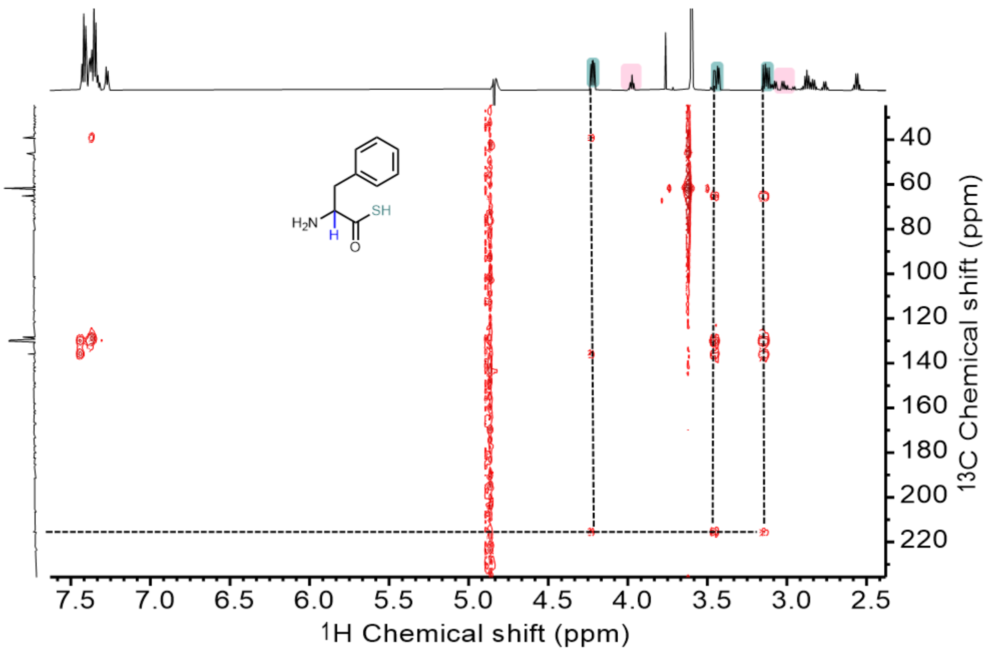

Supplementary Figure 42. ^{1}H–^{13}C *HMBC (*^{1}H*: 600 MHz [2.5 – 7.7 ppm],* ^{13}C*: 176 MHz [30 – 220 ppm],* H_2O/D_2O *9:1) spectrum showing the diagnostic* ^{2}JCH *and* ^{3}JCH *coupling of both phenyl-aH and* $-CH_2C_6H_5$ *in* ***12*** $_{\mathbf{Phe}}$ *at 4.21 ppm, 3.44 ppm and 3.14 ppm with a resonance at 215.5 ppm, which is characteristic of amino thioacid formation.*

*Synthesis of serine thioacid **12**$_{Ser}$ from serine thioester **1**$_{Ser}$*

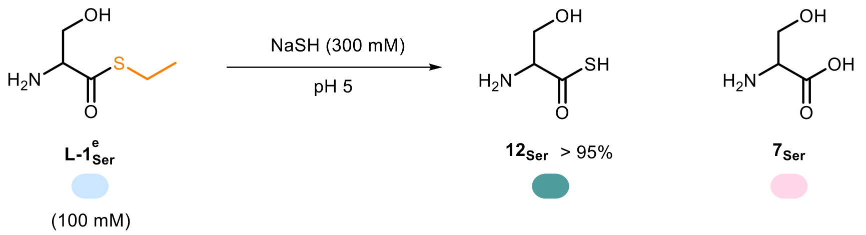

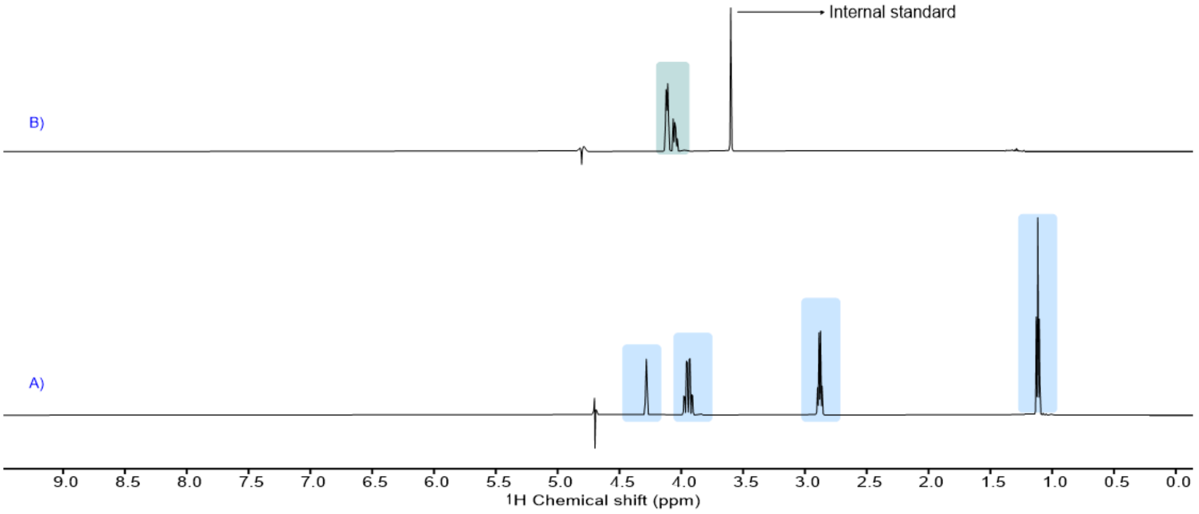

*Supplementary Figure 43: ^{1}H NMR (600 MHz, H_2O/D_2O 9:1, noesygppr1d, 1.0–7.8 ppm) spectrum shows the reaction of serine thioester (**1**$^e_{Ser}$, 100 mM) with NaSH (300 mM), using PET (10 mM) as an internal standard. Set up following General Procedure B. A) **1**$^e_{Ser}$; B) **12**$_{Ser}$ formed from the reaction of **1**$^e_{Ser}$ with NaSH [final pH = 8.0].*

^{1}H NMR (600 MHz, H_2O/D_2O 9:1) (2-amino-3-phenylpropanethioic *S*-acid), **12**$_{ser}$ (partial assignment) : δ_H 4.16 – 4.08 (2H, m, Ser-CHC*H*$_2$OH), 4.09 – 3.96 (1H, m, Ser-α-C*H*CH$_2$OH).

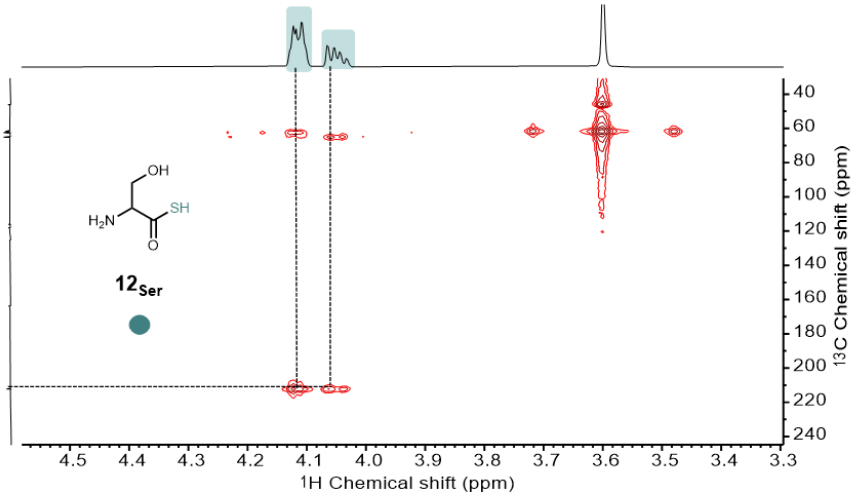

*Supplementary Figure 44: ^{1}H–^{13}C HMBC (^{1}H: 600 MHz [2.5-7.7 ppm], ^{13}C: 176 MHz [30-220 ppm], H_2O/D_2O 9:1) spectrum showing the diagnostic ^{2}JCH and ^{3}JCH coupling of both serinyl-aH and a-H-CH$_2$OH in **12**$_{Ser}$ at 4.05 ppm and 4.11 ppm with a resonance at 211.5 ppm, which is characteristic of amino thioacid formation.*

Thiol exchange to form 1^{n}_{Ala} from alanine thioester 1^{e}_{Ala} at pH 6.5

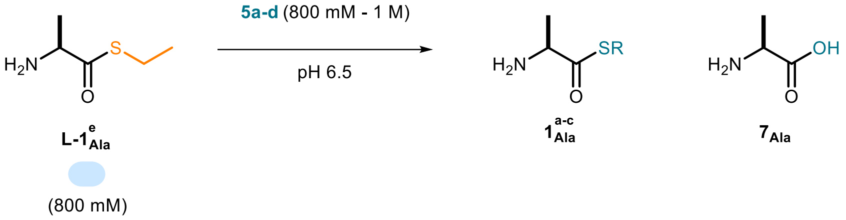

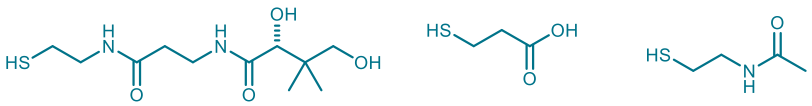

5a **5b** **5d**

L-Alanine thioester (L-$\mathbf{1}^{e}_{Ala}$, 0.4 mmol) and the specified thiol (**5a-d**, 0.4–0.5 mmol) were dissolved in degassed H_2O/D_2O (98:2, 0.5 mL) at pH 6.5. Nitrogen was bubbled through the solution for 15 minutes, and the reaction mixture was then incubated at room temperature. NMR spectra were immediately acquired to confirm the formation of $\mathbf{1}^{a\text{-}d}_{Ala}$. The product yield was quantified based on the methyl group of alanyl L-$\mathbf{1}^{n}_{Ala}$.

Entry	Thiol	pH	$\mathbf{1}^{n}_{Ala}$ (%)	7_{Ala} (%)
1	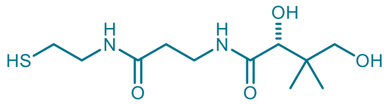 **5a**	6.5	89	9
2	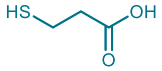 **5b**	6.5	93	6
3	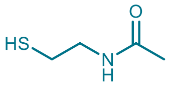 **5d**	6.5	95	5

Supplementary Table 5. Yields (%) of alanine thioester 1^{a-c}_{Ala} and alanine 1_{Ala} from the reaction of L-1^{e}_{Ala} (800 mM) with the specified thiol ($5_{a\text{-}d}$, 800 mM – 1M) after 30 minutes at pH 6.5.

Thioester stability at different pH

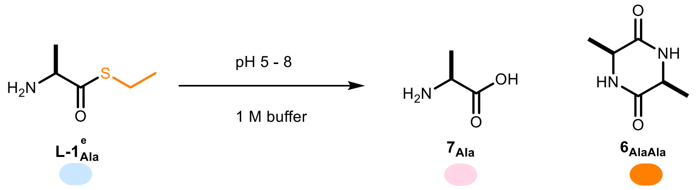

L-Alanine thioester (1^{e}_{Ala}, 100 µmol) was dissolved in a buffered solution of 9:1 H_2O: D_2O (1 M MES or MOPS at pH 5, 6.5 or 8, 0.5 mL). pH was re-adjusted to the desired value (pH 5, 6.5 or 8). NMR spectra were acquired periodically.

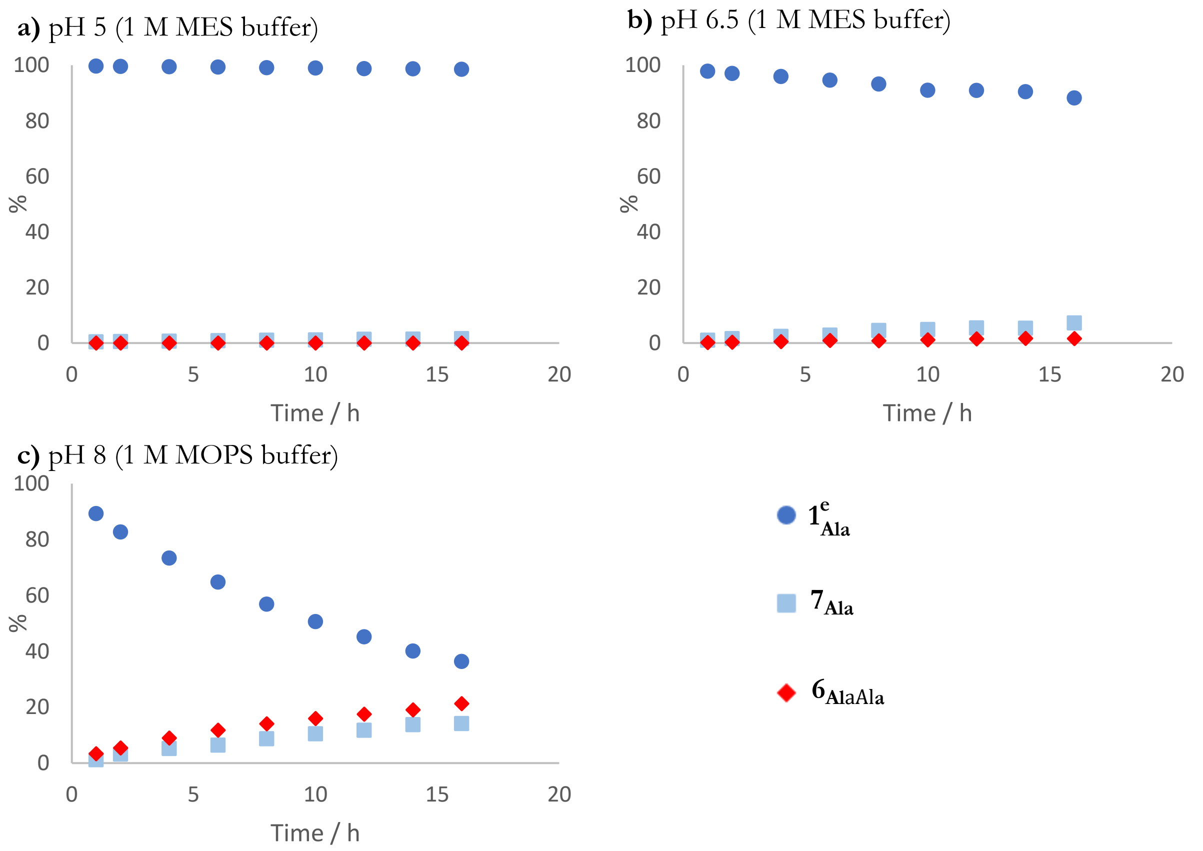

Supplementary Figure 45. ¹H NMR yields (%) of hydrolysis (7_{Ala}) and diketopiperazine 6_{AlaAla} when thioester 1^{e}_{Ala} (200 mM) is incubated in a) MES buffer (1 M, pH 5.0); B) MES buffer (1 M, pH 6.5); C) MOPS buffer (1 M, pH 8.0).

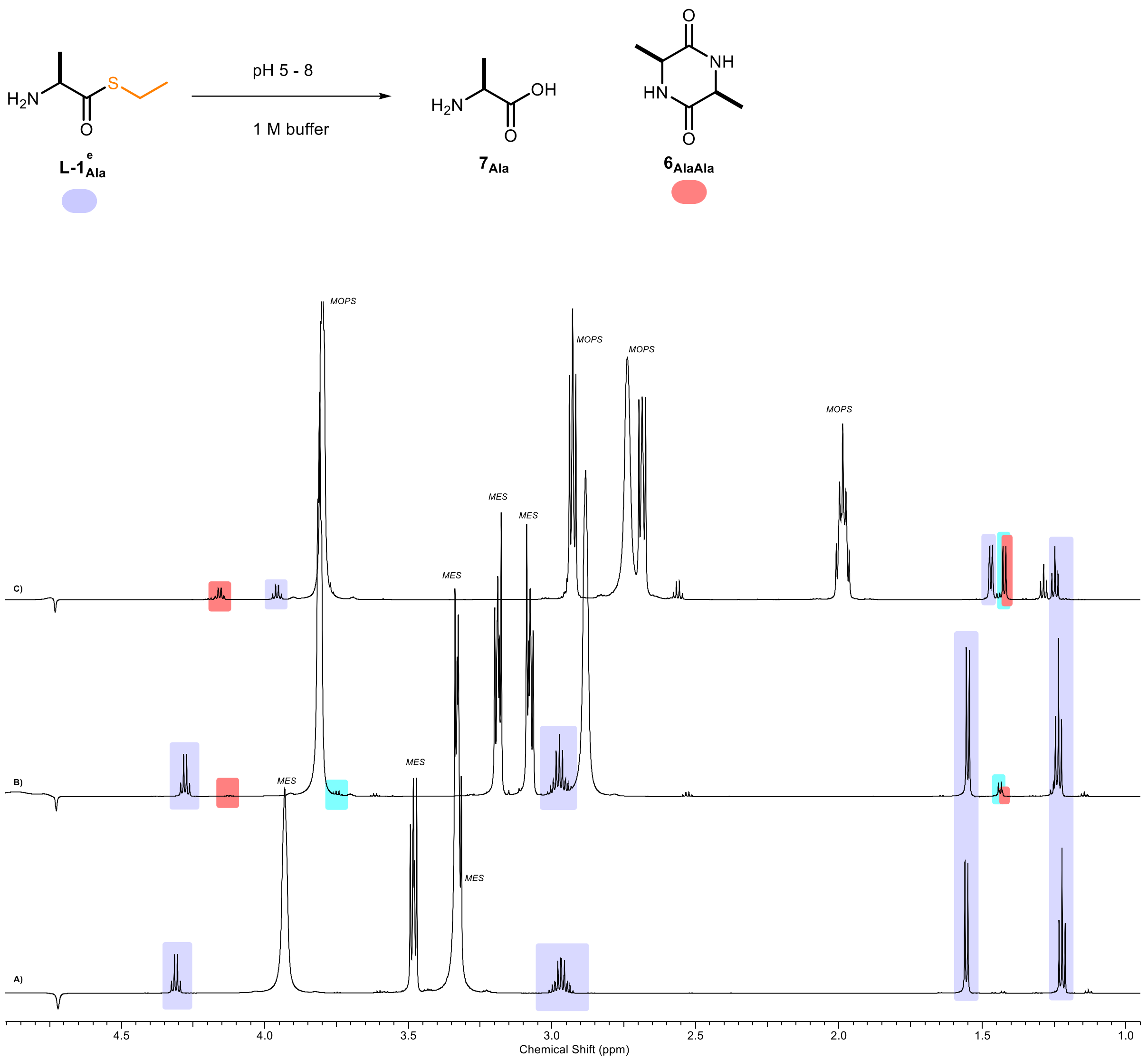

Supplementary Figure 46. ¹H NMR (700 MHz, H_2O/D_2O 9:1, noesygppr1d, 0.5 – 9.0 ppm) spectra to show the reaction of thioester $\mathbf{1^{e}_{Ala}}$ *(200 mM) at pH 5-8 after 16 h.* ***A)*** *pH 5, 1 M MES buffer;* ***B)*** *pH 6.5, 1 M MES buffer;* ***C)*** *pH 8, 1 M MOPS buffer.*

Determination of Thioester **L-1$^{e}_{Aaa}$** pK$_{aH}$

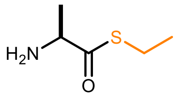

L-1$^{e}_{Ala}$

Thioester **L-1$^{e}_{Aa}$** (50 mM) and MSM (20 mM, internal standard) were dissolved in H_2O/D_2O (9:1). The solution was adjusted to the specified pH using HCl/NaOH (1 – 4 M) and analysed by ^{1}H NMR spectroscopy. ^{1}H NMR spectra were reference to the MSM methyl resonance (δ_H = 3.10 ppm) and the chemical shift of the α-methylene (α-CH) resonance of thioester **L-1$^{e}_{Aaa}$** was measured (Supplementary Figure 47).

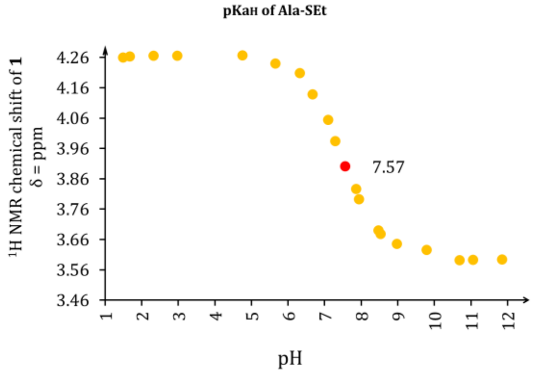

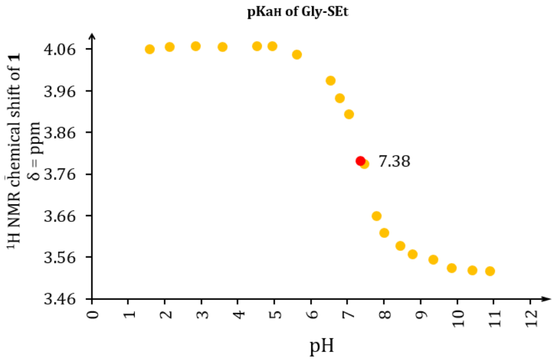

*Supplementary Figure 47: Thioester **L-1$^{e}_{Aa}$** a-methylene ^{1}H NMR resonance chemical shift change (a-CH) observed upon pH titration of thioester **1** (a-CHNH$_3^+$ → a-CHNH$_2$)*

Thioester-mediated aminoacylation of nucleoside and nucleotide monomers

General procedure C – Formation of aminoacyl nucleosides ***17*** *by the reaction of aminoacyl thiols* ***1*** *and nucleosides or nucleotides*

Thioester ($\mathbf{1^{e}_{Aaa}}$, 0.1 mmol) and pentaerythritol (10 mM) were dissolved in degassed H_2O/D_2O (98:2, 0.4 mL) and adjusted to the stated pH with NaOH/HCl. Nucleoside (0.01 mmol) was added, the solution pH was re-adjusted to the stated value, and the volume set to 0.5 mL with degassed H_2O/D_2O (98:2). The resultant solution was incubated at room temperature, and NMR spectra were periodically acquired. The structure of α-aminoacyl ester was confirmed by ^1H–^1H COSY NMR analysis, alongside the diagnostic downfield chemical shifts of 2'/3' protons.

Chemo- and Regioselectivity for aminoacylation of nucleosides upon reaction with thioester $\mathbf{1^{e}_{Ala}}$

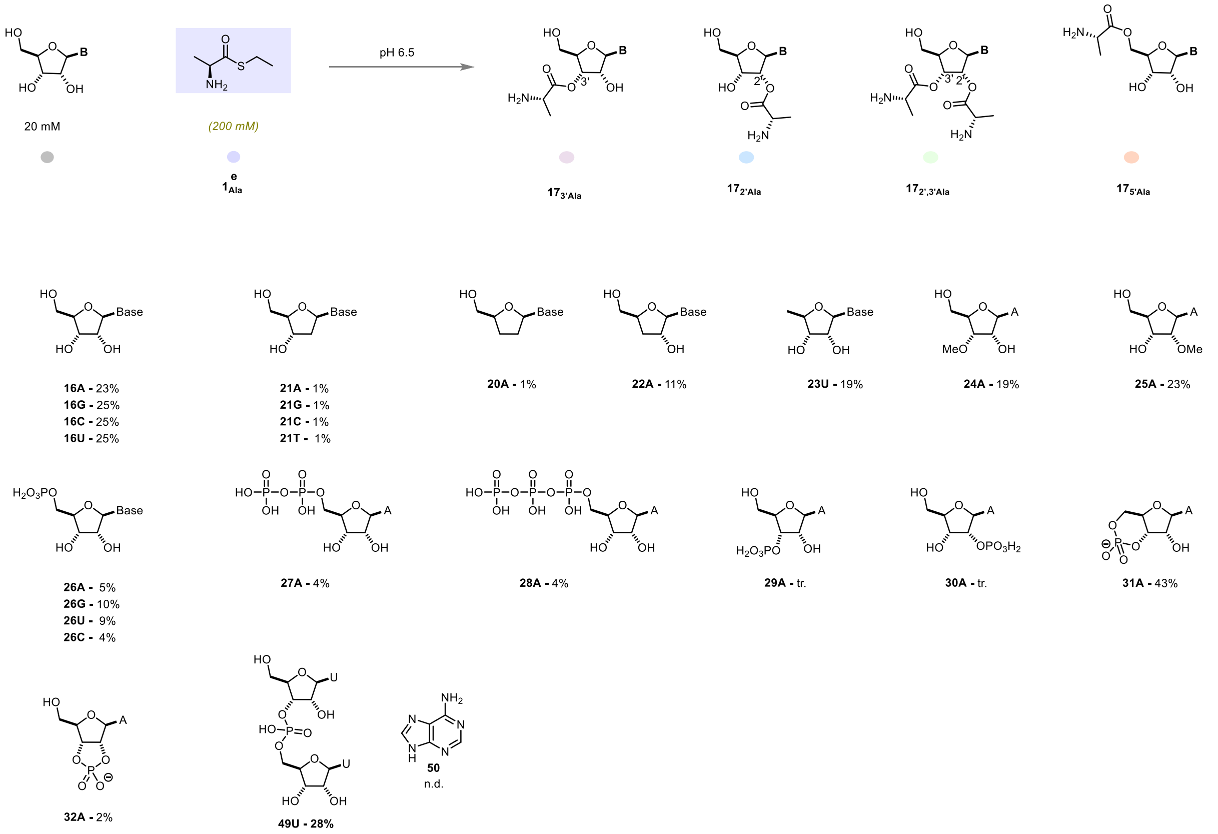

Entry	nucleoside	Time (hours)	**18**$_{3'Aaa}$ (%)	**18**$_{2'Aaa}$ (%)	**18**$_{2',3'Aaa}$ (%)	**18**$_{5'Aaa}$ (%)	**Total** (%)
1	**16A**	24	15	5	2	<1	23
2	**16G**[3]	24	16	5	4	n.d.	25
3	**16C**	24	12	9	3	1	25
4	**16U**	24	14	7	3	2	25
5*	**21A**	24	1	--	--	n.d.	1
6*	**21G**[6]	24	1	--	--	n.d.	1
7*	**21C**	24	1	--	--	n.d.	1
8*	**21T**	24	1	--	--	n.d.	1
9	**22A**	24	--	9	--	1	10
10	**23U**	24	10	7	2	--	19
11	**20A**	24	--	--	--	1	1
12	**24A**	24	--	18	--	1	19
13	**25A**	24	22	--	--	1	23
14	**26A**	24	4	1	--	--	5
15	**26G**	24	7	3	--	--	10
16	**26U**	24	6	3	--	--	9
17	**26C**	24	obs.	obs.	--	--	4
18	**28A**	24	3	1	--	--	4
19	**29A**	24	--	tr.	--	n.d.	tr.
20	**30A**	24	tr.	--	--	n.d.	tr.
21	**31A**	12	43	--	--	--	43
22	**32A**	24	--	--	--	2	2
23	**49A**	24	3	1	--	--	4
24	**50**	24	--	--	--	--	--

*Supplementary Table 6. Total yields for α-aminoacyl ester observed in the reaction of thioester (**1**$^{e}_{Ala}$, 200 mM) and nucleoside (20 mM) with pentaerythritol (10 mM, internal standard) at pH 6.5, after 24 h and room temperature, unless stated otherwise. Set up following General Procedure C. tr. = trace (<0.5%); n.d. = not detected; -- = no hydroxyl moiety. See Supplementary Figures 48-69, 77 for NMR spectra. *All deoxynucleotides (**21**) gave equally low yields for aminoacylation; spectra provided for **21A** (Supplementary Figure 51).*

[3] Due to low solubility of guanosine, the reaction was performed at the following nucleoside concentrations: Guanosine (**16G**, 2 mM); Deoxyguanosine (**21G**, 5 mM).

Aminoacylation of nucleoside ***16C*** *with aminoacyl thiol* $\mathbf{1_{Ala}}$ *at pH 6.5*

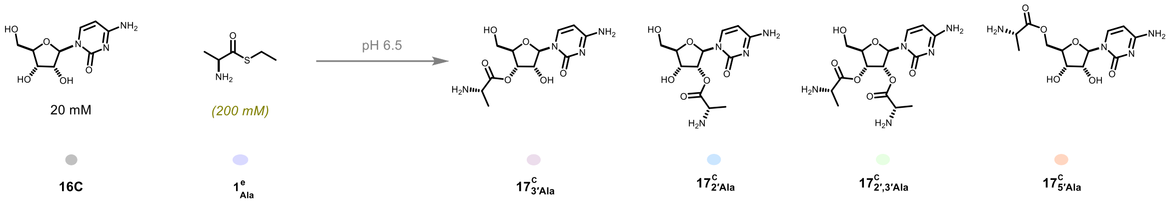

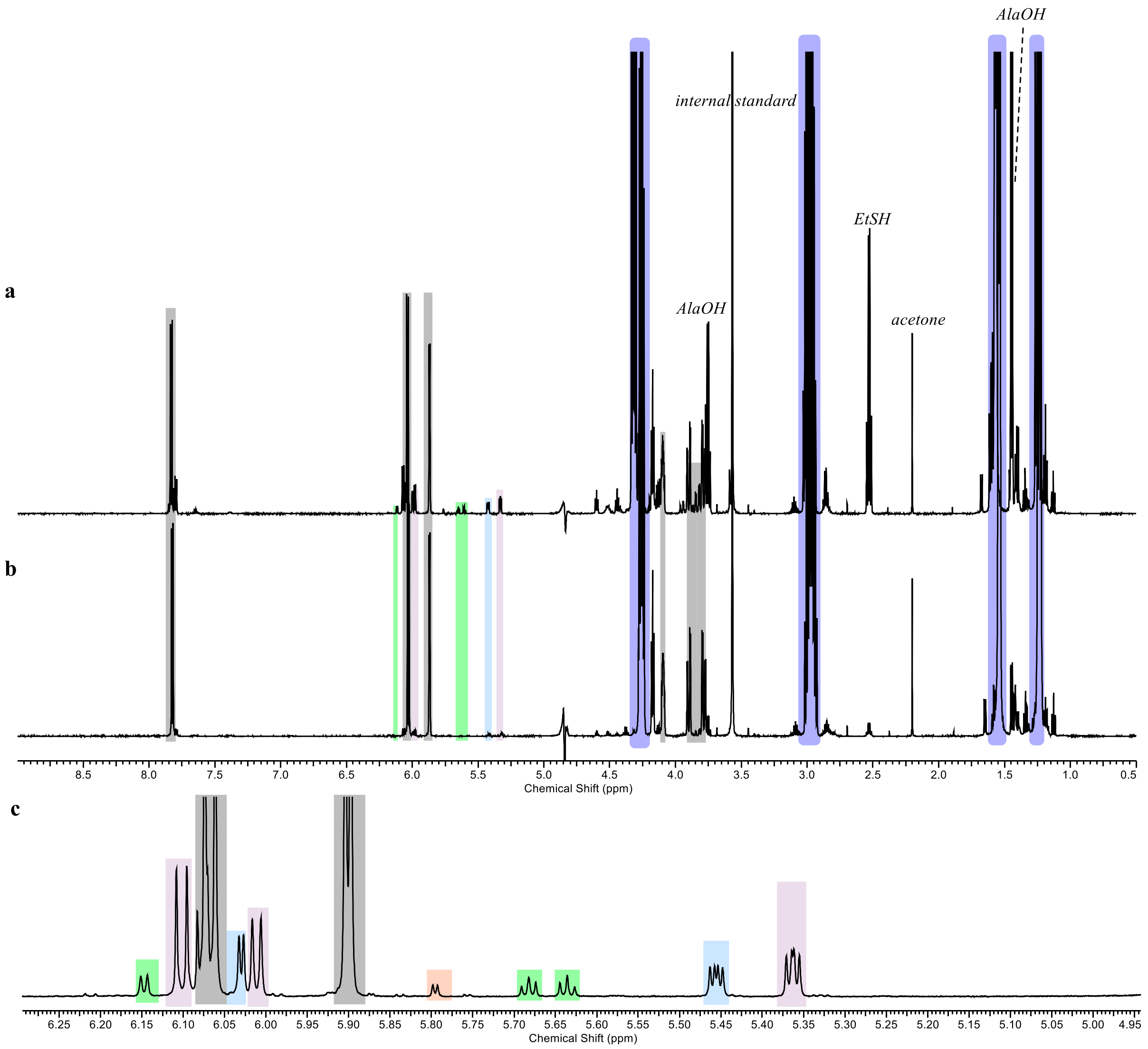

Supplementary Figure 48. ¹*H NMR (600 MHz, H_2O/D_2O 9:1, noesygppr1d, 0.5 – 9.0 ppm) spectra to show the reaction of thioester* $\mathbf{1^{e}_{Ala}}$ *(200 mM) with* ***16C*** *(20 mM) at pH 6.5, using pentaerythritol (10 mM) as an internal standard. Set up following General Procedure C after* ***a)*** *24 h;* ***b)*** *1 h;* ***c)*** *zoom-in of 24-hour timepoint (spectrum* ***a)*** *from 4.9 ppm - 6.3 ppm.*

¹H NMR (600 MHz, H_2O/D_2O 9:1) $\mathbf{17^{C}_{3'Ala}}$ (partial assignment): δ_H 6.01 (1H, d, J = 6.2 Hz, (C1')-H), 5.36 (1H, dd, J = 5.5, 4.0 Hz, (C3')-H);

¹H NMR (600 MHz, H_2O/D_2O 9:1) $\mathbf{17^{C}_{2'Ala}}$ (partial assignment) : δ_H 6.03 (1H, d, J = 3.3 Hz, (C1')-H), 5.46 (1H, dd, J = 5.8, 3.4 Hz, (C2')-H);

^{1}H NMR (600 MHz, H_2O/D_2O 9:1) $\mathbf{17^{C}_{5'Ala}}$ (partial assignment) : δ_H 5.80 (1H, d, *J* = 3.4 Hz, (C1')-H);

^{1}H NMR (600 MHz, H_2O/D_2O 9:1) $\mathbf{17^{C}_{2'3'Ala}}$ (partial assignment) : δ_H 6.15 (1H, d, *J* = 4.7 Hz, (C1')-H), 5.68 (1H, app. t, *J* = 5.3 Hz, (C2')-H), 5.64 (1H, app. t, *J* = 5.2 Hz, (C3')-H).

*Aminoacylation of nucleoside **16G** with aminoacyl thiol **1_{Ala}** at pH 6.5*

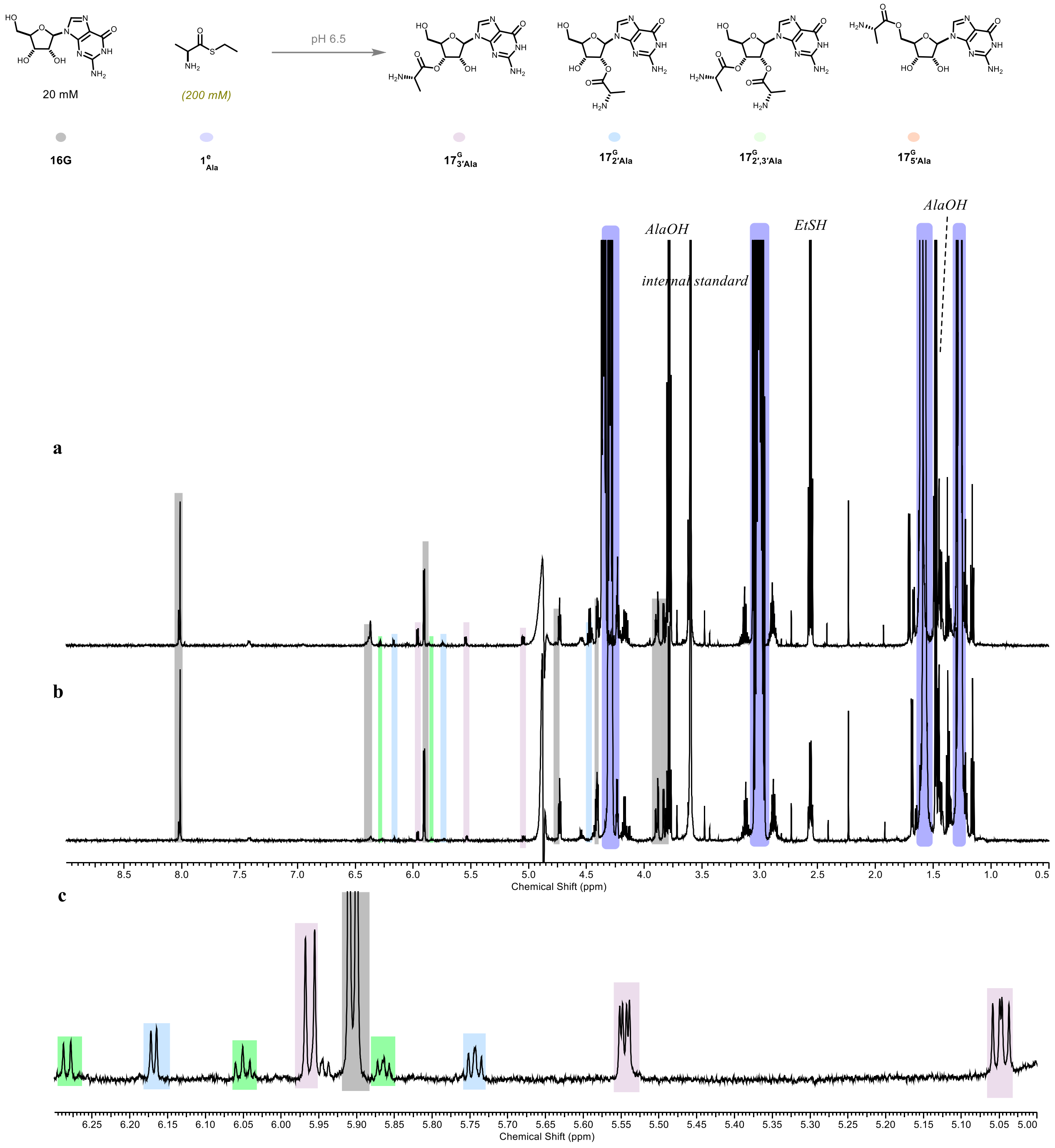

*Supplementary Figure 49. ^{1}H NMR (600 MHz, H_2O/D_2O 9:1, noesygppr1d, 0.5 – 9.0 ppm) spectra to show the reaction of thioester **1^e_{Ala}** (200 mM) with **16G** (20 mM) at pH 6.5, using pentaerythritol (10 mM) as an internal standard. Set up following General Procedure C after: **a)** 24 h; **b)** 1 h; **c)** zoom-in of 24-hour timepoint (spectrum **a)** from 5.0 ppm - 6.3 ppm.*

^{1}H NMR (600 MHz, H_2O/D_2O 9:1) **$17^G_{3'Ala}$** (partial assignment): δ_H 5.97 (1H, d, *J* = 7.3 Hz, (C1')-H), 5.54 (1H, dd, *J* = 5.5, 2.3 Hz, (C3')-H), 5.05 (1H, dd, *J* = 7.3, 5.5 Hz, (C2')-H);

^{1}H NMR (600 MHz, H_2O/D_2O 9:1) **$17^G_{2'Ala}$** (partial assignment): δ_H 6.16 (1H, d, *J* = 4.5 Hz, (C1')-H), 5.74 (1H, app. t, *J* = 5.1 Hz, (C3')-H);

^{1}H NMR (600 MHz, H_2O/D_2O 9:1) $\mathbf{17^{G}_{2'3'Ala}}$ (partial assignment): δ_H 6.28 (1H, d, J = 4.7 Hz, (C1')-H), 6.05 (1H, app. t, J = 5.7 Hz, (C2')-H), 5.86 (1H, app. t, J = 4.2 Hz, (C3')-H).

*Aminoacylation of nucleoside **16A** with aminoacyl thiol **1**$_{Ala}$ at pH 6.5*

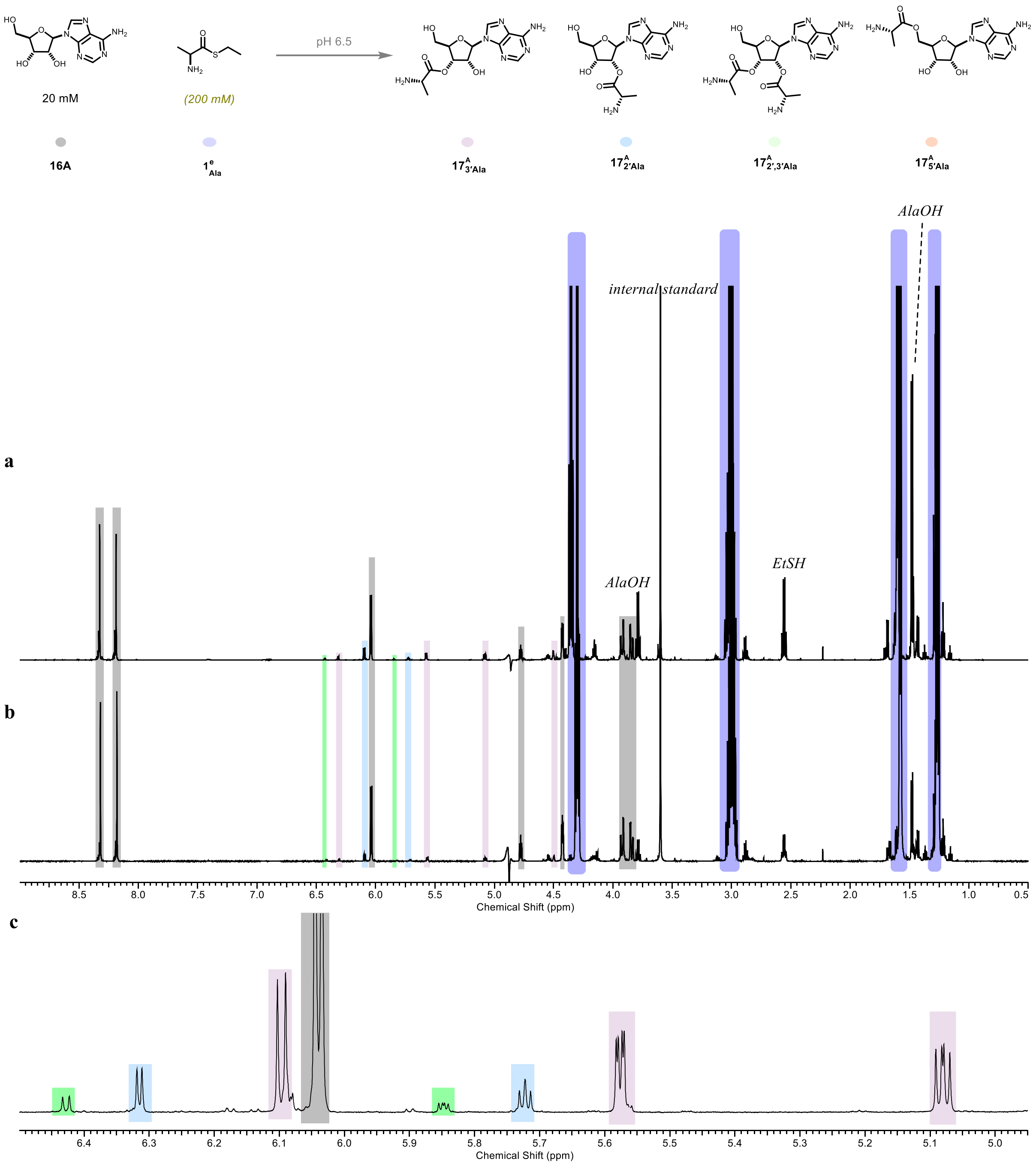

*Supplementary Figure 50. ^{1}H NMR (600 MHz, H_2O/D_2O 9:1, noesygppr1d, 0.5 – 9.0 ppm) spectra to show the reaction of thioester $\mathbf{1^{e}_{Ala}}$ (200 mM) with **16A** (20 mM) at pH 6.5, using pentaerythritol (10 mM) as an internal standard after. Set up following General Procedure C **a)** 24 h; **b)** 1 h; **c)** zoom-in of 24 hour timepoint (spectrum **a)** from 4.9 ppm - 6.5 ppm.*

^{1}H NMR (600 MHz, H_2O/D_2O 9:1) $\mathbf{17^{A}_{3'Ala}}$ (partial assignment) : δ_H 6.10 (1H, d, J = 7.4 Hz, (C1')-H), 5.57 (1H, dd, J = 5.4, 1.9 Hz, (C3')-H), 5.08 (1H, dd, J = 7.4, 5.5 Hz, (C2')-H);

^{1}H NMR (600 MHz, H_2O/D_2O 9:1) $\mathbf{17^{A}_{2'Ala}}$ (partial assignment): δ_H 6.32 (1H, d, *J* = 4.7 Hz, (C1')-H), 5.72 (1H, app. t, *J* = 5.1 Hz, (C2')-H);

^{1}H NMR (600 MHz, H_2O/D_2O 9:1) $\mathbf{17^{A}_{2'3'Ala}}$ (partial assignment): δ_H 6.43 (1H, d, *J* = 6.0 Hz, (C1')-H), 5.85 (1H, dd, *J* = 5.2, 3.3 Hz, (C3')-H).

*Aminoacylation of nucleoside **21A** with aminoacyl thiol **1**$_{Ala}$ at pH 6.5*

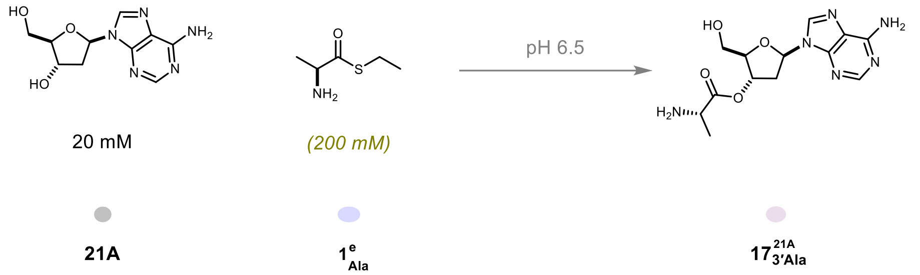

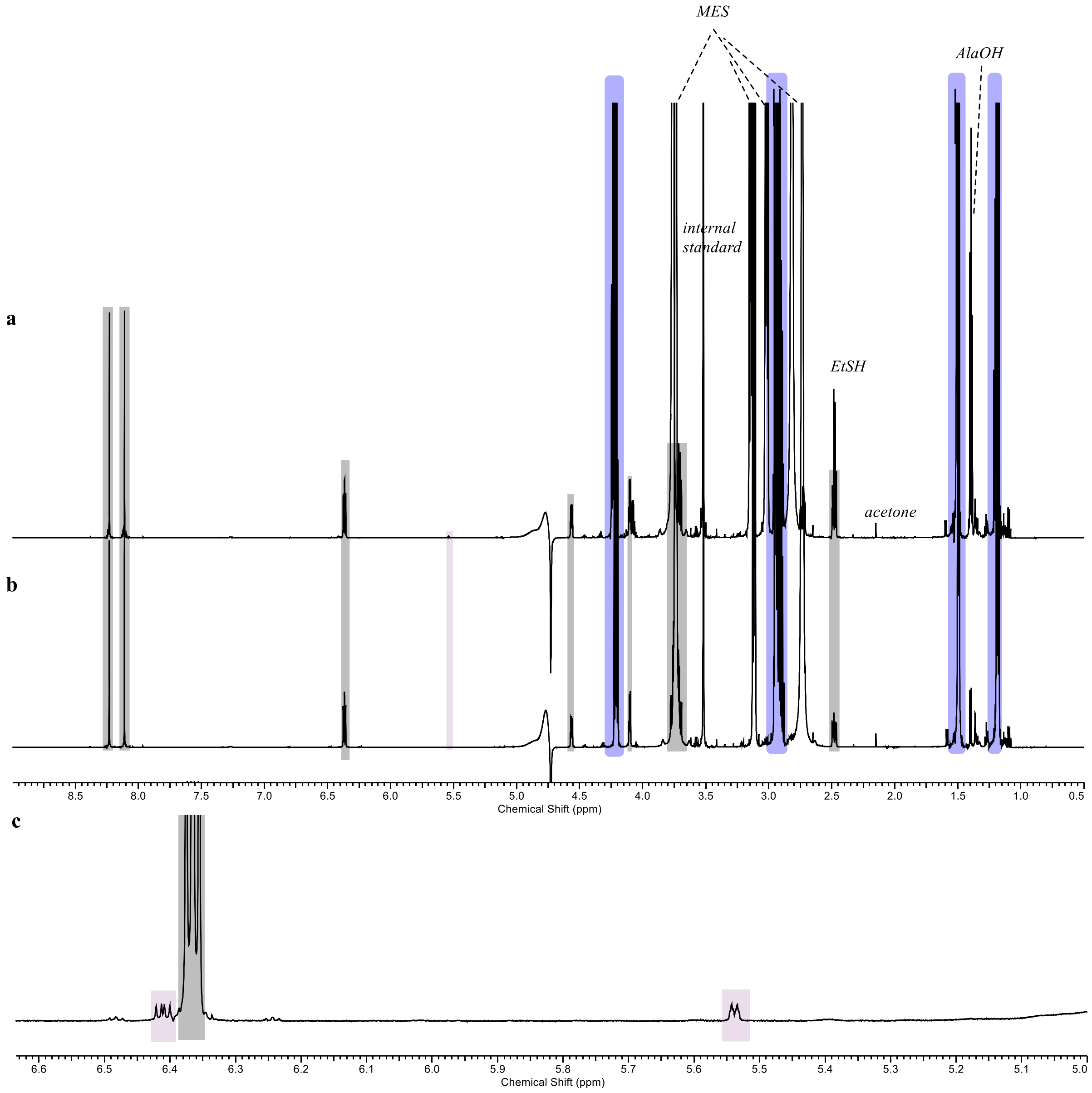

Supplementary Figure 51. ¹H NMR (600 MHz, H$_2$O/D$_2$O 9:1, noesygppr1d, 0.5 – 9.0 ppm) spectra to show the reaction of thioester $\mathbf{1}^{e}_{Ala}$ *(200 mM) with **21A** (20 mM) at pH 6.5, using pentaerythritol (10 mM) as an internal standard and MES buffer. Set up following General Procedure C after: **a**) 24 h; **b**) 1 h; **c)** zoom-in of 24-hour timepoint (spectrum **a**) from 5.0 ppm - 6.6 ppm.*

^{1}H NMR (600 MHz, H_2O/D_2O 9:1) $\mathbf{17^{21A}_{3'Ala}}$ (partial assignment): δ_H 6.49 (1H, dd, J = 8.8, 5.7 Hz, (C1')-H), 5.62 (1H, d, J = 6.1 Hz, (C3')-H).

*Aminoacylation of nucleoside **22A** with aminoacyl thiol $\mathbf{1_{Ala}}$ at pH 6.5*

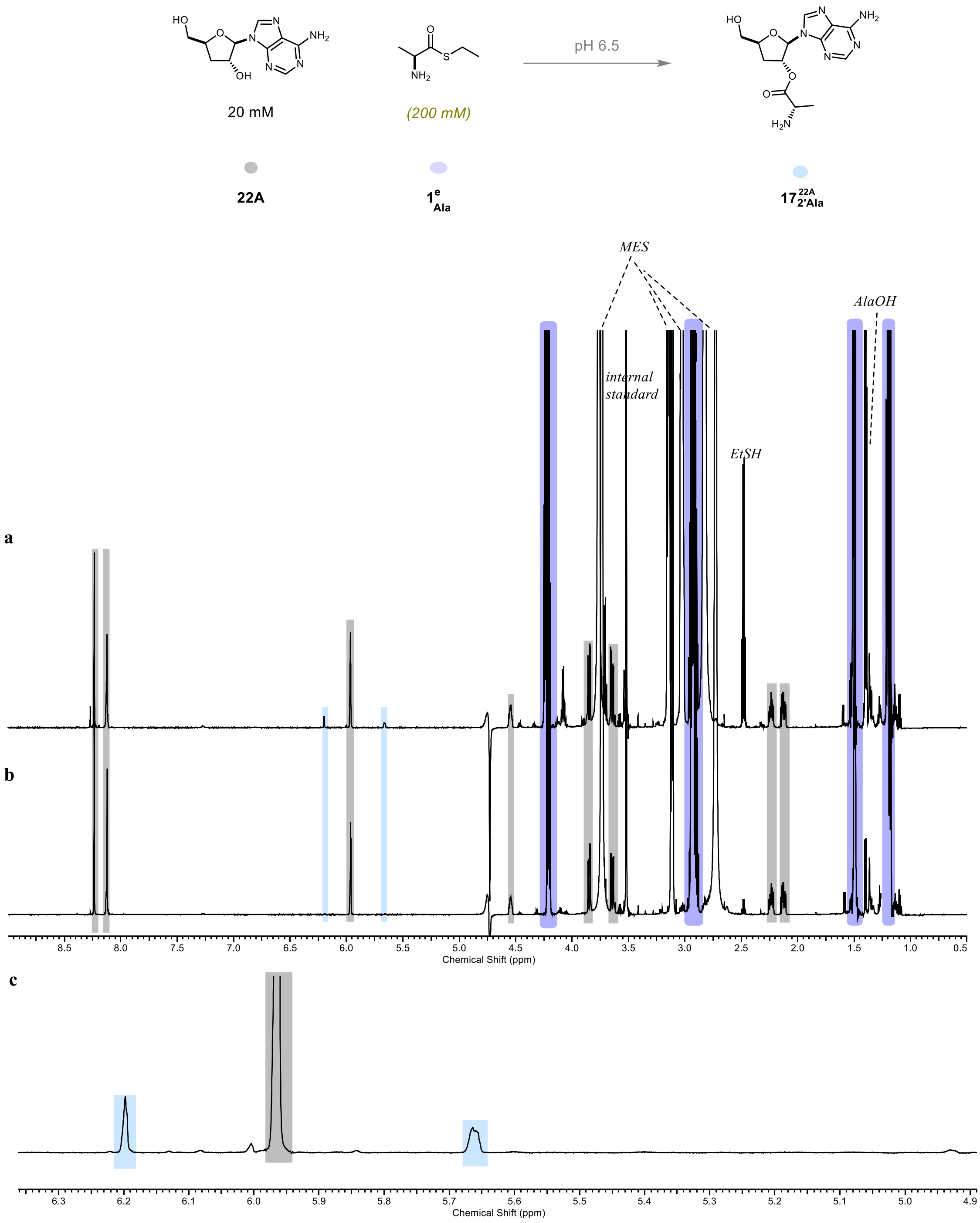

*Supplementary Figure 52. ^{1}H NMR (600 MHz, H_2O/D_2O 9:1, noesygppr1d, 0.5 – 9.0 ppm) spectra to show the reaction of thioester $\mathbf{1^e_{Ala}}$ (200 mM) with **22A** (20 mM) at pH 6.5, using pentaerythritol (10 mM) as an internal standard and MES buffer. Set up following General Procedure C after: **a)** 24 h; **b)** 1 h; **c)** zoom-in of 24-hour timepoint (spectrum **a**) from 4.9 ppm - 6.4 ppm.*

^{1}H NMR (600 MHz, H_2O/D_2O 9:1) $\mathbf{17^{22A}_{2'Ala}}$ (partial assignment) : δ_H 8.27 (s, C2-H), 6.20 (1H, s, (C1')-H), 5.66 (1H, app. s, (C2')-H).

*Aminoacylation of nucleoside **23U** with aminoacyl thiol $\mathbf{1_{Ala}}$ at pH 6.5*

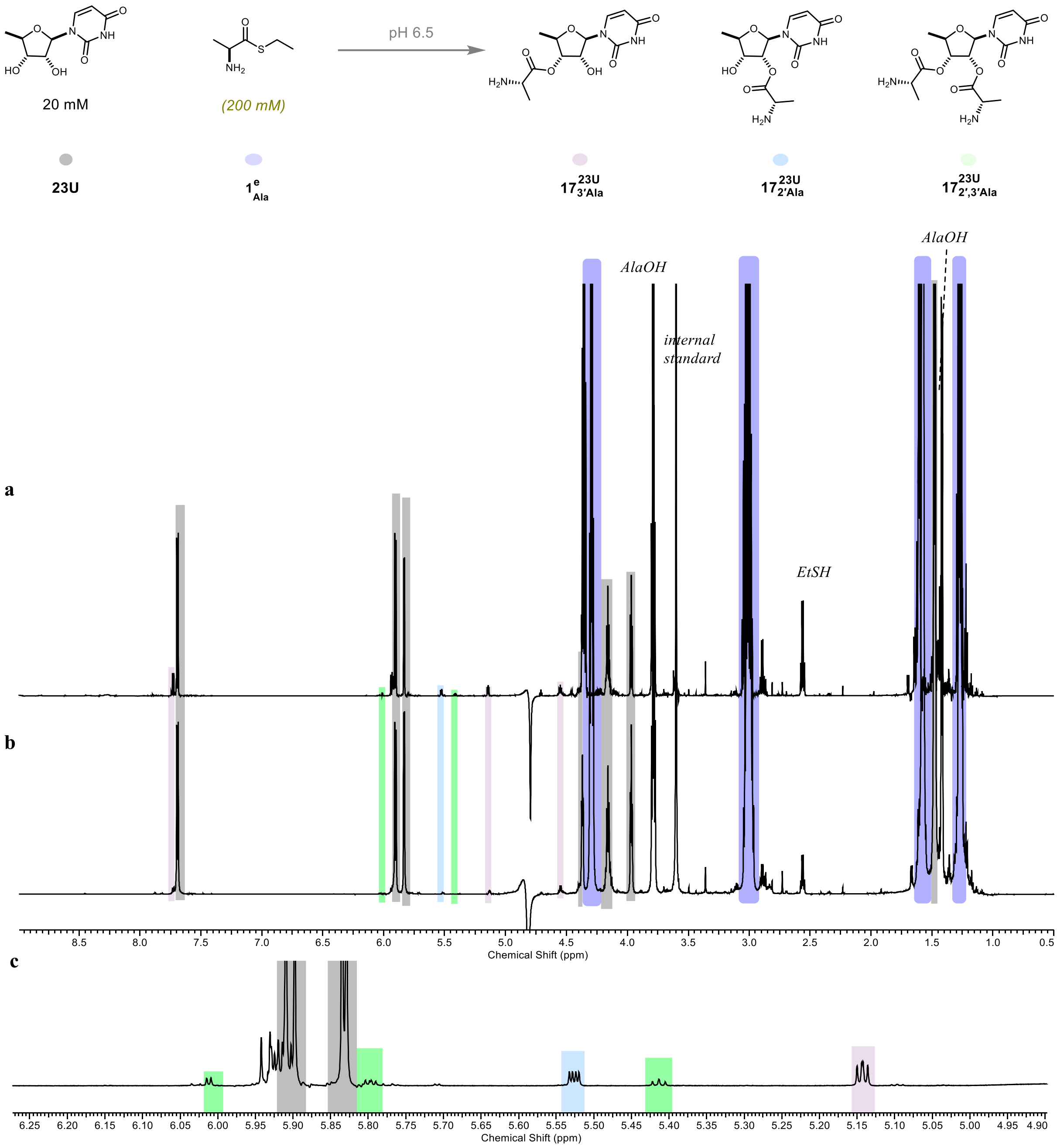

*Supplementary Figure 53. ^{1}H NMR (600 MHz, H_2O/D_2O 9:1, noesygppr1d, 0.5 – 9.0 ppm) spectra to show the reaction of thioester $\mathbf{1^e_{Ala}}$ (200 mM) with **23U** (20 mM) at pH 6.5, using pentaerythritol (10 mM) as an internal standard. Set up following General Procedure C after: **a)** 24 h; **b)** 1 h; **c)** zoom-in of 24-hour timepoint (spectrum **a**) from 4.9 ppm - 6.3 ppm.*

^{1}H NMR (600 MHz, H_2O/D_2O 9:1) $\mathbf{17^{23U}_{2'Ala}}$ (partial assignment): δ_H 5.53 (1H, dd, J = 5.9, 2.8 Hz, (C2')-H),

^{1}H NMR (600 MHz, H_2O/D_2O 9:1) $\mathbf{17^{23U}_{3'Ala}}$ (partial assignment): δ_H 5.14 (1H, dd, J = 5.5, 4.4 Hz, (C3')-H).

^{1}H NMR (600 MHz, H_2O/D_2O 9:1) $\mathbf{17^{23U}_{2'3'Ala}}$ (partial assignment): δ_H 6.01 (1H, d, J = 4.0 Hz, (C1')-H), 5.80 (1H, dd J = 5.6, 4.0 Hz, (C2')-H), 5.41 (1H, app. t, J = 6.1 Hz, (C3')-H).

*Aminoacylation of nucleoside **20A** with aminoacyl thiol **1**$_{Ala}$ at pH 6.5*

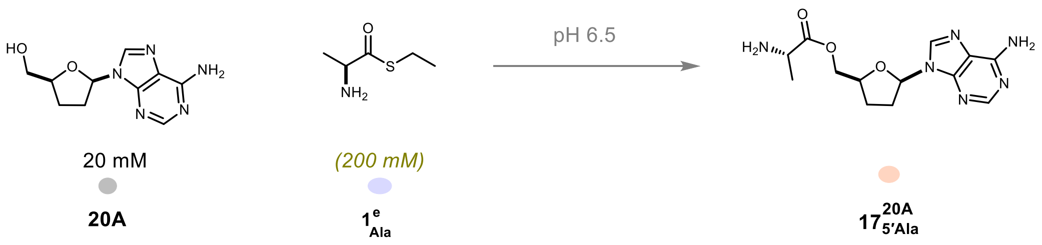

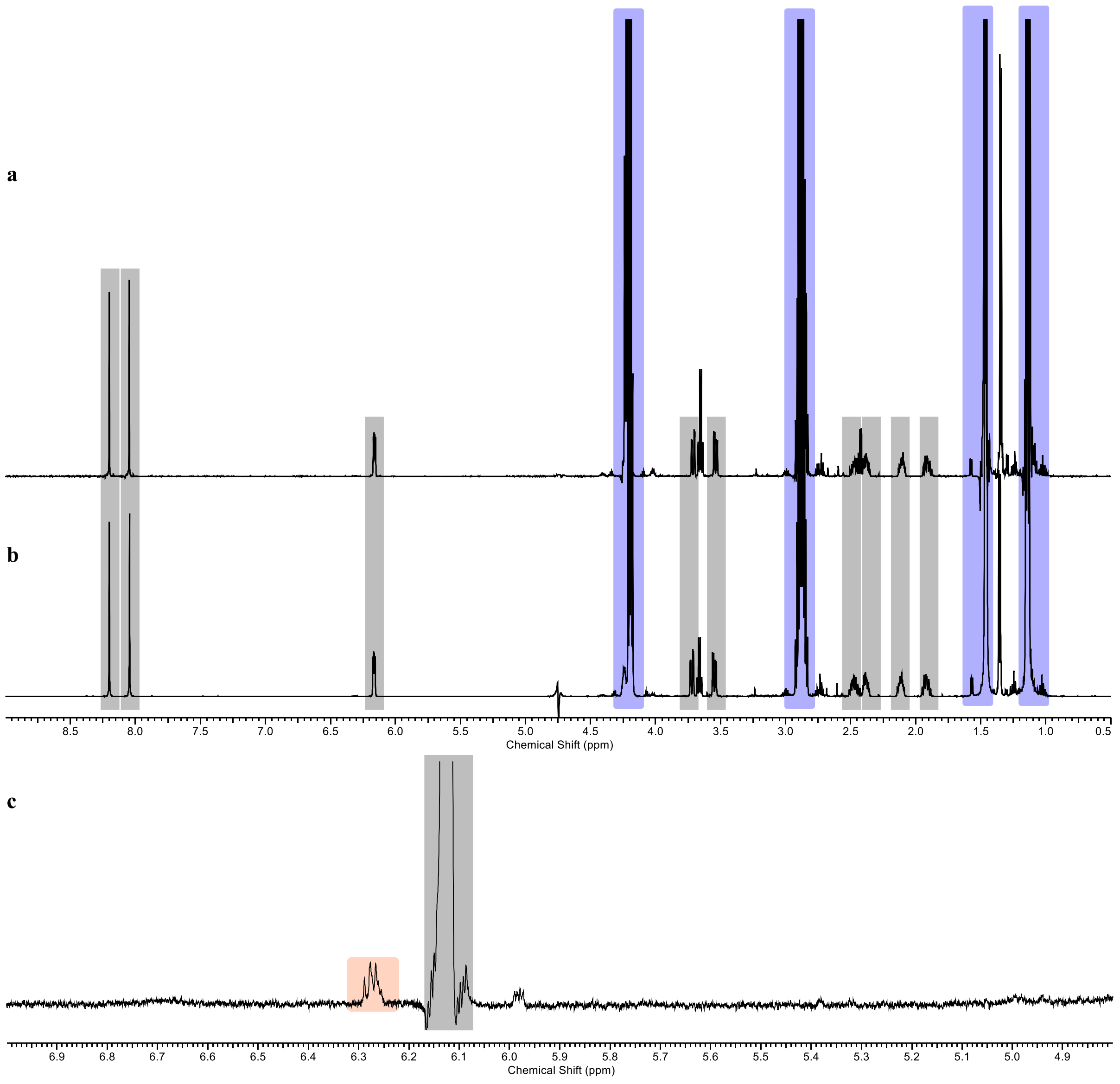

*Supplementary Figure 54. ^{1}H NMR (600 MHz, H_2O/D_2O 9:1, noesygppr1d, 0.5 – 9.0 ppm) spectra to show the reaction of thioester **1^e_{Ala}** (200 mM) with **20A** (20 mM) at pH 6.5, using pentaerythritol (10 mM) as an internal standard. Set up following General Procedure C after: **a)** 24 h; **b)** 1 h; **c)** zoom-in of 24-hour timepoint (spectrum **a**) from 4.9 ppm – 7.0 ppm.*

*Aminoacylation of nucleoside **24A** with aminoacyl thiol **1_{Ala}** at pH 6.5*

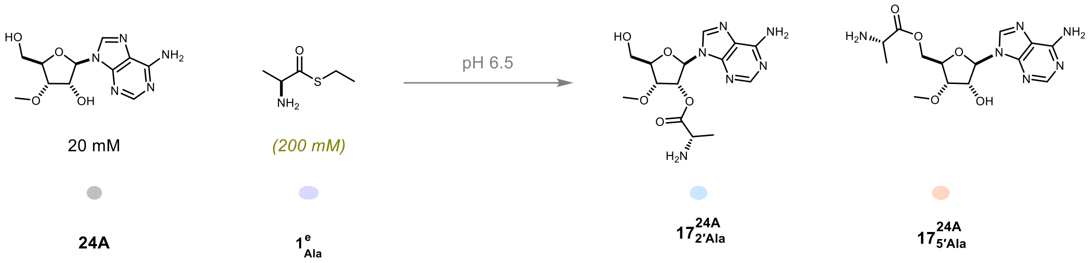

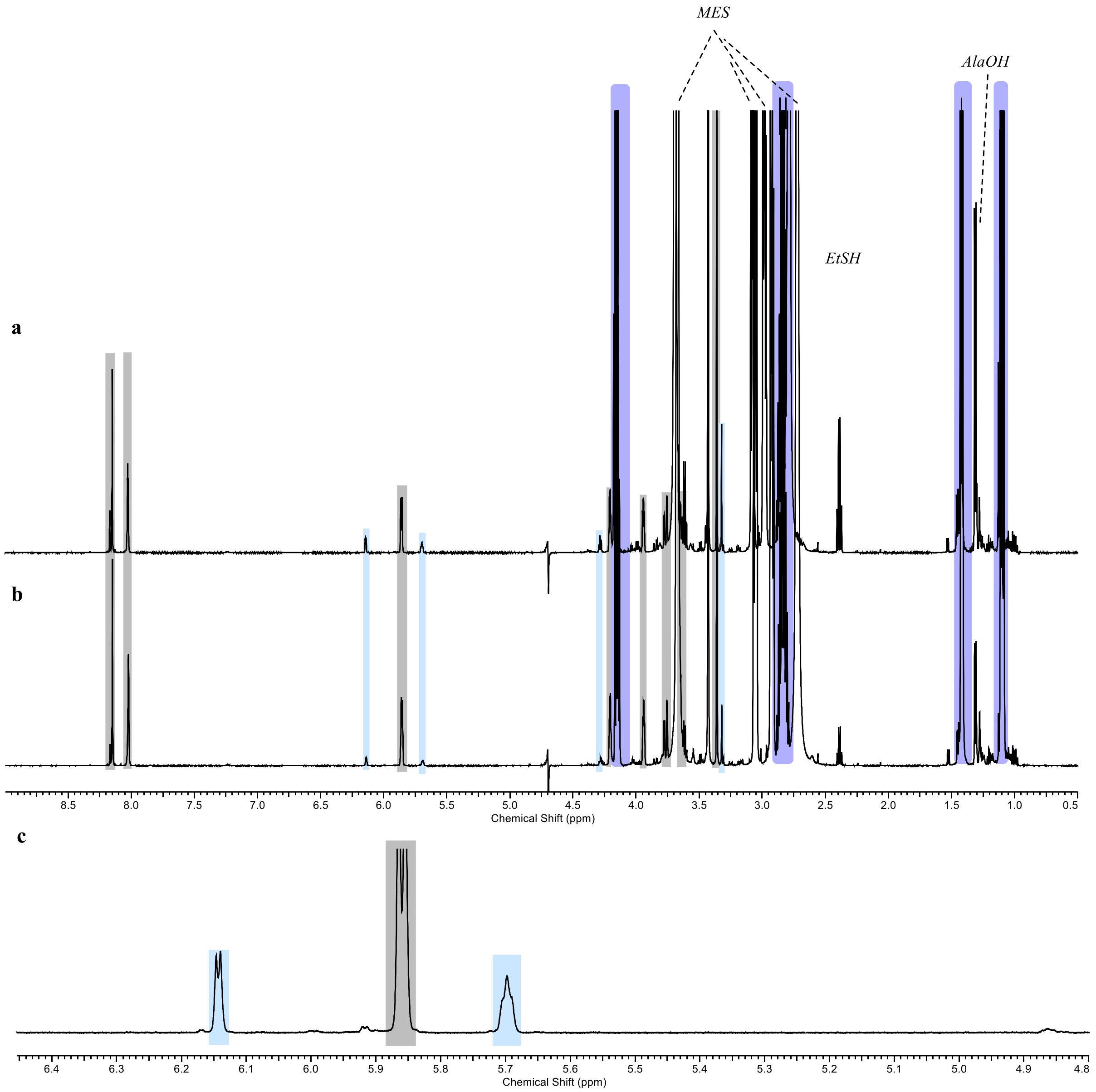

*Supplementary Figure 55. ^{1}H NMR (600 MHz, H_2O/D_2O 9:1, noesygppr1d, 0.5 – 9.0 ppm) spectra to show the reaction of thioester **1^e_{Ala}** (200 mM) with **24A** (20 mM) at pH 6.5, using pentaerythritol (10 mM) as an internal standard and MES buffer. Set up following General Procedure C after: **a)** 24 h; **b)** 1 h; **c)** zoom-in of 24-hour timepoint (spectrum **a**) from 4.8 ppm - 6.4 ppm.*

^{1}H NMR (600 MHz, H_2O/D_2O 9:1) **$17^{24A}_{2'Ala}$** (partial assignment): δ_H 8.17 (s, 1H, (C2)-H), 6.14 (1H, d, *J* = 4.0 Hz, (C1')-H), 5.70 (1H, app. t, *J* = 4.1 Hz, (C2')-H).

*Aminoacylation of nucleoside **25A** with aminoacyl thiol $\mathbf{1_{Ala}}$ at pH 6.5*

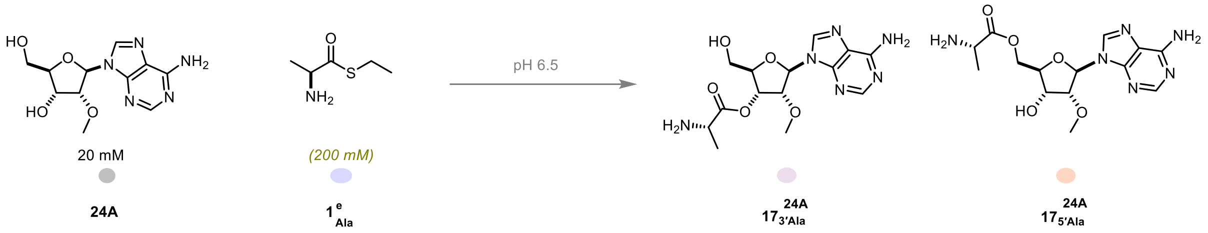

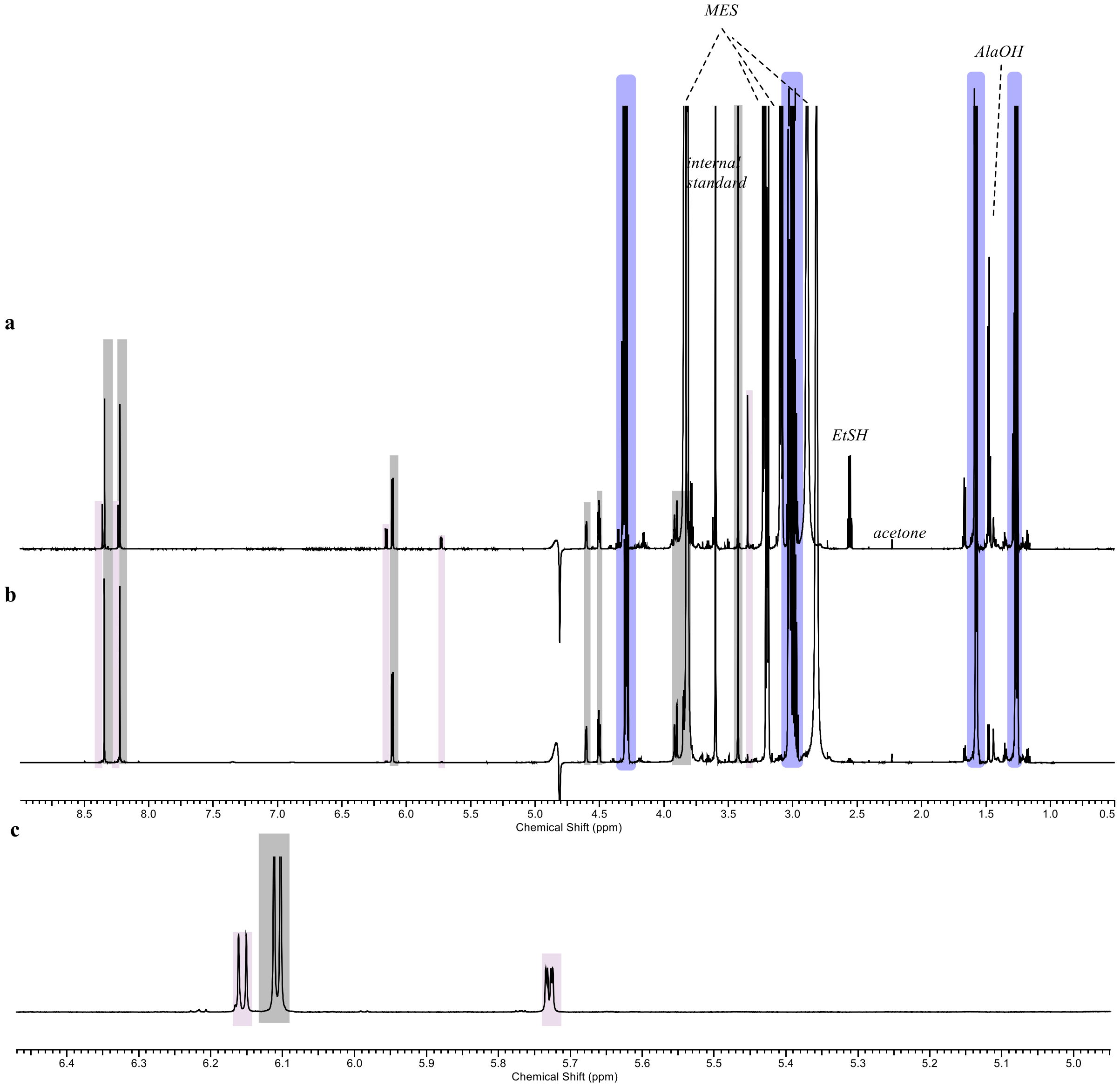

Supplementary Figure 56. ¹H NMR (600 MHz, H_2O/D_2O 9:1, noesygppr1d, 0.5 – 9.0 ppm) spectra to show the reaction of thioester $\mathbf{1^{e}_{Ala}}$ *(200 mM) with **25A** (20 mM) at pH 6.5, using pentaerythritol (10 mM) as an internal standard and MES buffer. Set up following General Procedure C after: **a**) 24 h; **b**) 1 h; **c)** zoom-in of 24 hour timepoint (spectrum **a**) from 4.9 ppm – 6.5 ppm.*

¹H NMR (600 MHz, H_2O/D_2O 9:1) $\mathbf{17^{25A}_{3'Ala}}$ (partial assignment) : δ_H 8.36 (s, 1H, (C2)-H), 8.24 (s, 1H, (C8)-H), 6.15 (1H, d, J = 7.4 Hz, (C1')-H), 5.73 (1H, dd, J = 5.1, 1.7 Hz, (C3')-H).

*Aminoacylation of nucleoside **26U** with aminoacyl thiol $\mathbf{1_{Ala}}$ at pH 6.5*

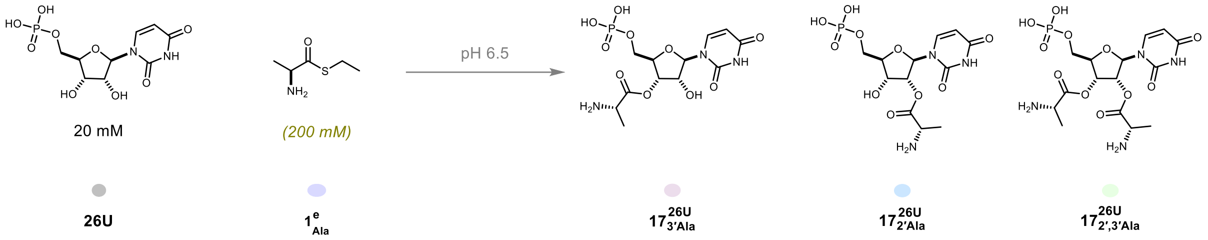

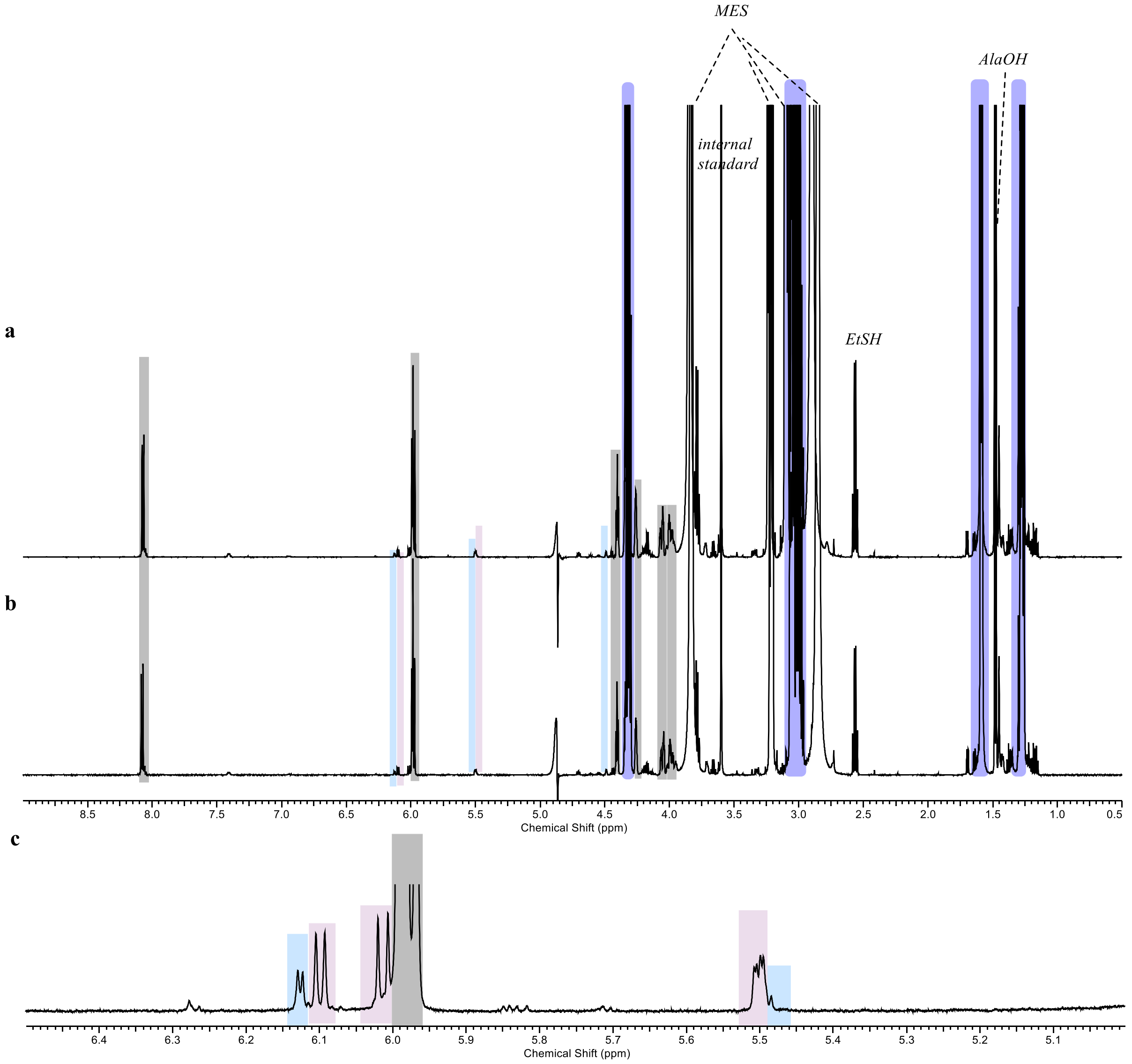

*Supplementary Figure 57. ¹H NMR (600 MHz, H_2O/D_2O 9:1, noesygppr1d, 0.5 – 9.0 ppm) spectra to show the reaction of thioester $\mathbf{1^{e}_{Ala}}$ (200 mM) with **26U** (20 mM) at pH 6.5, using pentaerythritol (10 mM) as an internal standard and MES buffer. Set up following General Procedure C after: **a)** 24 h; **b)** 4 h; **c)** zoom-in of 24 hour timepoint (spectrum **a**) from 5.0 ppm – 6.5 ppm.*

¹H NMR (600 MHz, H_2O/D_2O 9:1) $\mathbf{17^{26U}_{3'Ala}}$ (partial assignment) : δ_H 6.10 (1H, d, *J* = 7.2 Hz, (C1')-H), 6.02 (1H, d, *J* = 8.1 Hz, (C5)-H).

¹H NMR (600 MHz, H_2O/D_2O 9:1) $\mathbf{17^{26U}_{2'Ala}}$ (partial assignment) : δ_H 6.12 (1H, d, *J* = 4.0 Hz, (C1')-H).

*Aminoacylation of nucleoside **26G** with aminoacyl thiol $\mathbf{1_{Ala}}$ at pH 6.5*

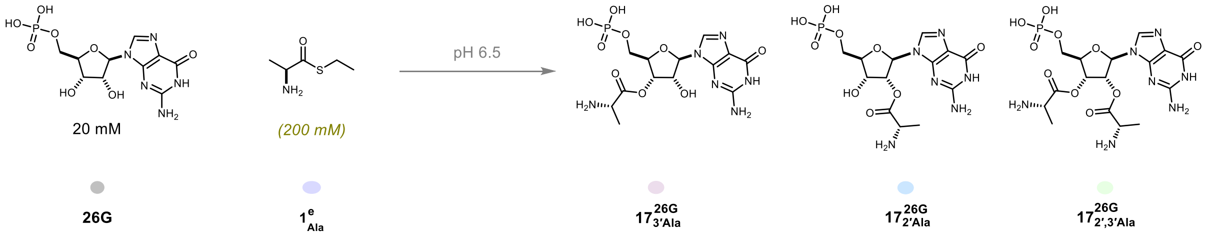

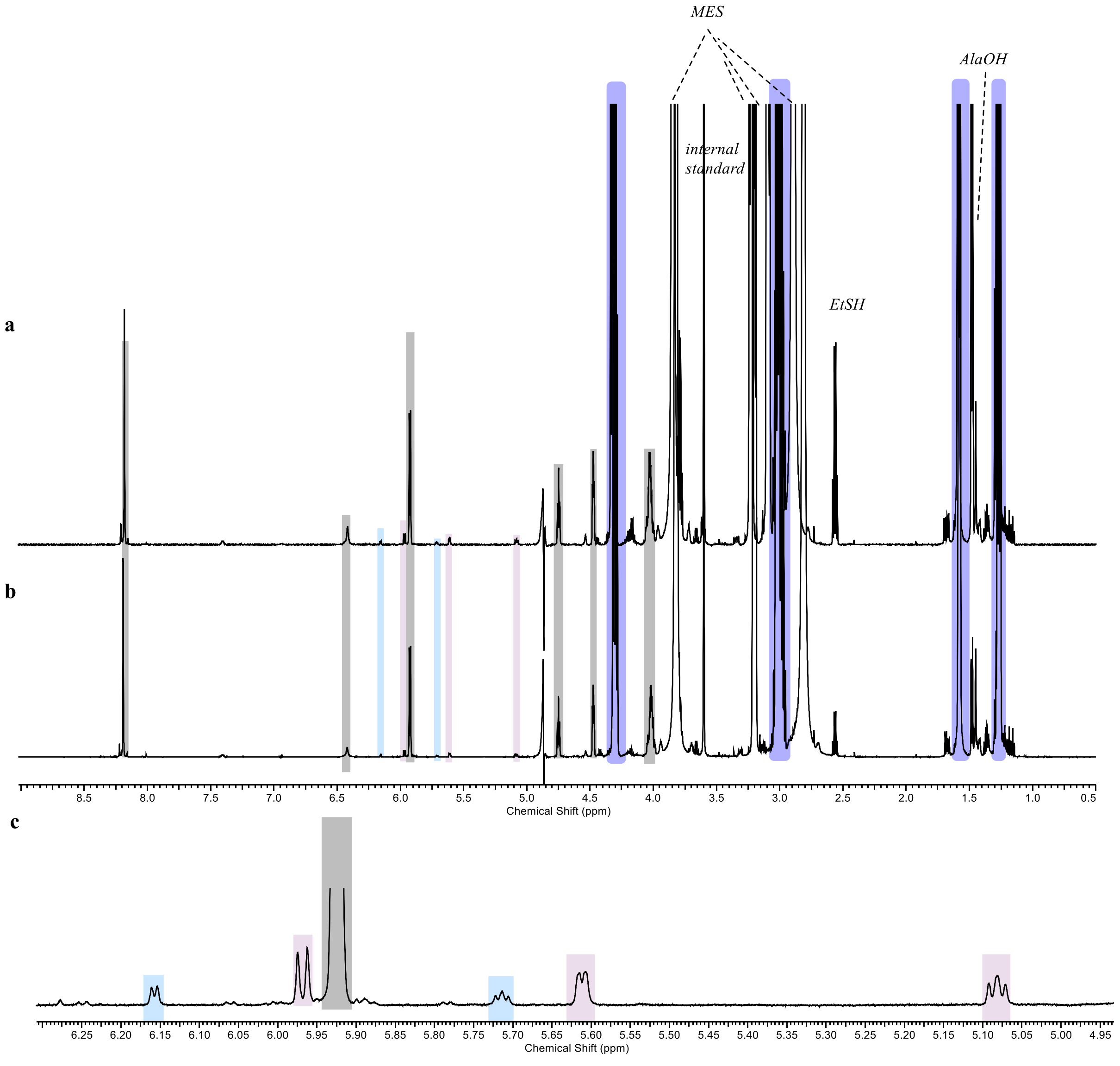

*Supplementary Figure 58. ^{1}H NMR (600 MHz, H_2O/D_2O 9:1, noesygppr1d, 0.5 – 9.0 ppm) spectra to show the reaction of thioester $\mathbf{1^{e}_{Ala}}$ (200 mM) with **26G** (20 mM) at pH 6.5, using pentaerythritol (10 mM) as an internal standard and MES buffer. Set up following General Procedure C after: **a**) 24 h; **b**) 1 h; **c)** zoom-in of 24-hour timepoint (spectrum **a**) from 4.9 ppm – 6.3 ppm.*

^{1}H NMR (600 MHz, H_2O/D_2O 9:1) $\mathbf{17^{26G}_{3'Ala}}$ (partial assignment) : δ_H 8.21 (1H, s, (C8)-H), 5.97 (1H, d, J = 7.3 Hz, (C1')-H), 5.81(1H, app. d, J = 5.4 Hz, (C3')-H), 5.08 (1H, app. t, J = 6.3 Hz, (C2')-H);

^{1}H NMR (600 MHz, H_2O/D_2O 9:1) $\mathbf{17^{26G}_{2'Ala}}$ (partial assignment) : δ_H 8.15 (1H, s, (C8)-H), 6.16 (1H, d, J = 4.1 Hz, (C1')-H), 5.71 (1H, app. t, J = 5.1 Hz, (C2')-H).

*Aminoacylation of nucleoside **26C** with aminoacyl thiol $\mathbf{1_{Ala}}$ at pH 6.5*

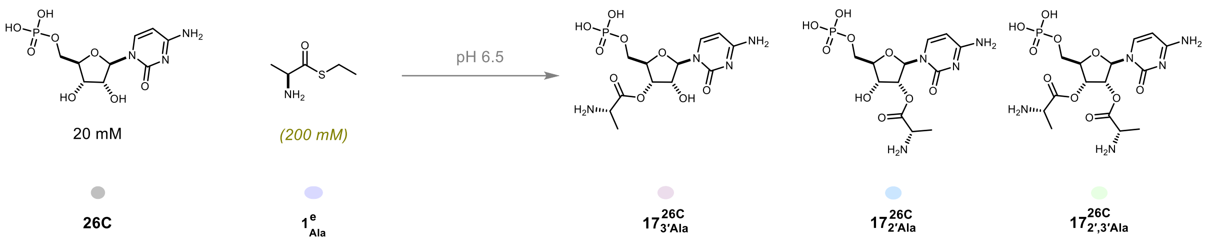

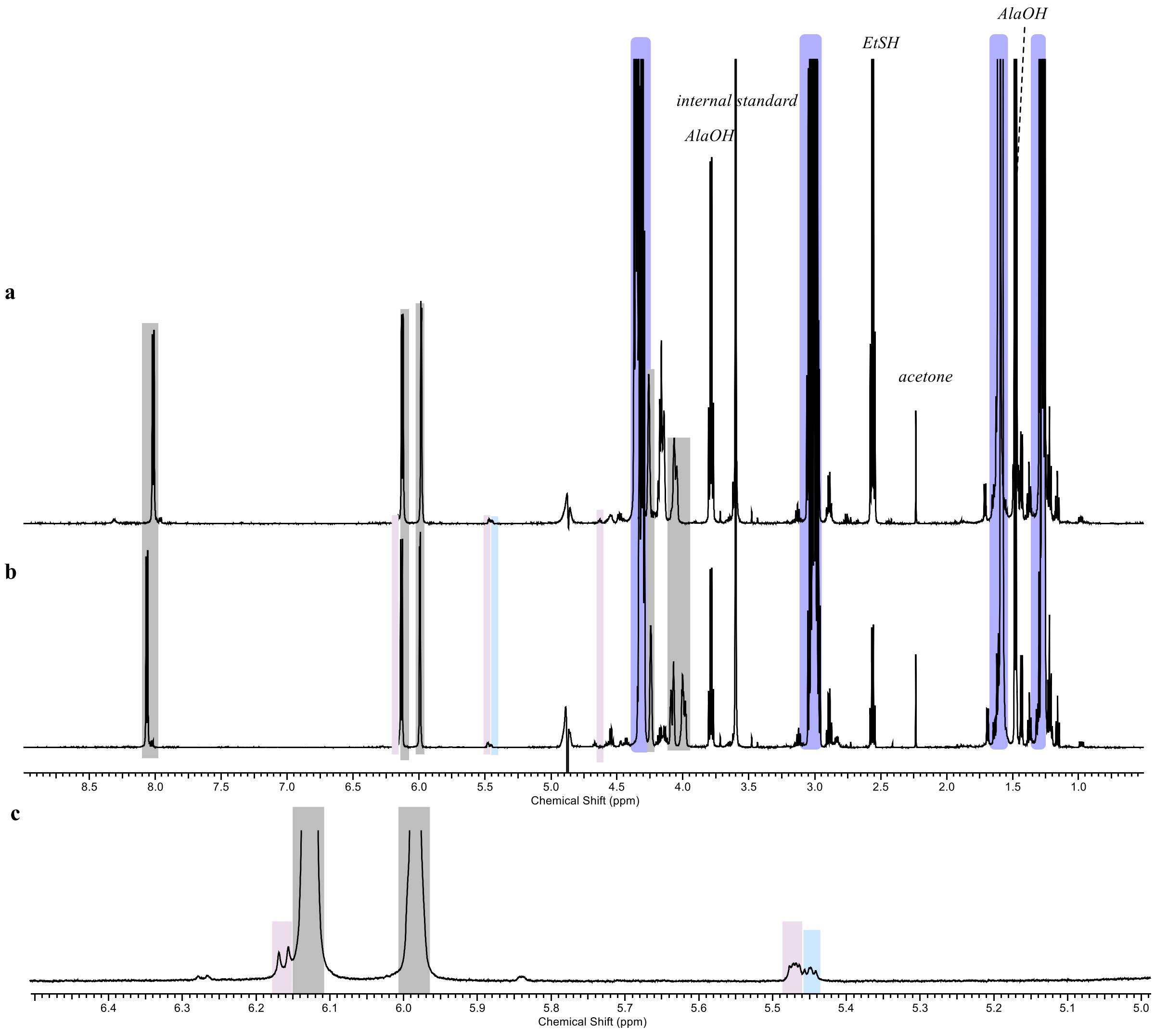

*Supplementary Figure 59. ^{1}H NMR (600 MHz, H_2O/D_2O 9:1, noesygppr1d, 0.5 – 9.0 ppm) spectra to show the reaction of thioester $\mathbf{1^{e}_{Ala}}$ (200 mM) with **26C** (20 mM) at pH 6.5, using pentaerythritol (10 mM) as an internal standard. Set up following General Procedure C after: **a)** 24 h; **b)** 1 h; **c)** zoom-in of 24-hour timepoint (spectrum **a)** from 5.0 ppm – 6.5 ppm.*

4% total aminoacylation was observed. Due to signal overlap the separated acylation regioisomers were not quantified.

*Aminoacylation of nucleoside **26A** with aminoacyl thiol $\mathbf{1_{Ala}}$ at pH 6.5*

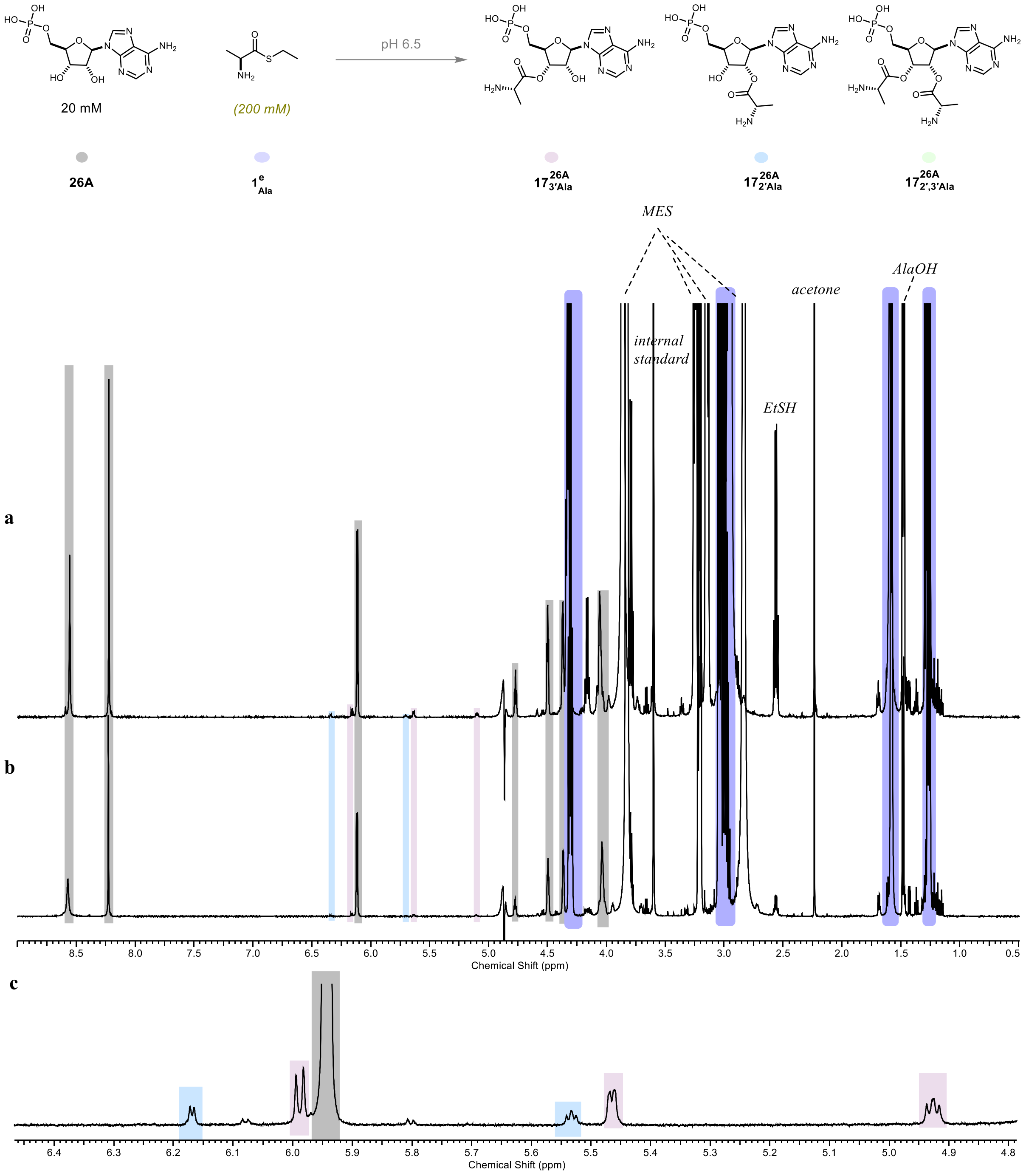

Supplementary Figure 60. ^{1}H NMR (600 MHz, H2O/D2O 9:1, noesygppr1d, 0.5 – 9.0 ppm) spectra to show the reaction of thioester $\mathbf{1^{e}_{Ala}}$ *(200 mM) with **26A** (20 mM) at pH 6.5, using pentaerythritol (10 mM) as an internal standard and MES buffer. Set up following General Procedure C after: **a)** 24 h; **b)** 1 h; **c)** zoom-in of 24-hour timepoint (spectrum **a**) from 4.8 ppm – 6.5 ppm.*

^{1}H NMR (600 MHz, H_2O/D_2O 9:1) $\mathbf{17^{26A}_{3'Ala}}$ (partial assignment) : δ_H 5.99 (1H, d, *J* = 7.3 Hz, (C1')-H), 5.81(1H, app. d, *J* = 5.0 Hz, (C3')-H), 5.08 (1H, app. t, *J* = 6.9 Hz, (C2')-H).

^{1}H NMR (600 MHz, H_2O/D_2O 9:1) $\mathbf{17^{26A}_{2'Ala}}$ (partial assignment) : δ_H 6.16 (1H, d, *J* = 3.9 Hz, (C1')-H), 5.53 (1H, app. t, *J* = 4.1 Hz, (C2')-H).

*Aminoacylation of nucleoside **27A** with aminoacyl thiol $\mathbf{1_{Ala}}$ at pH 6.5*

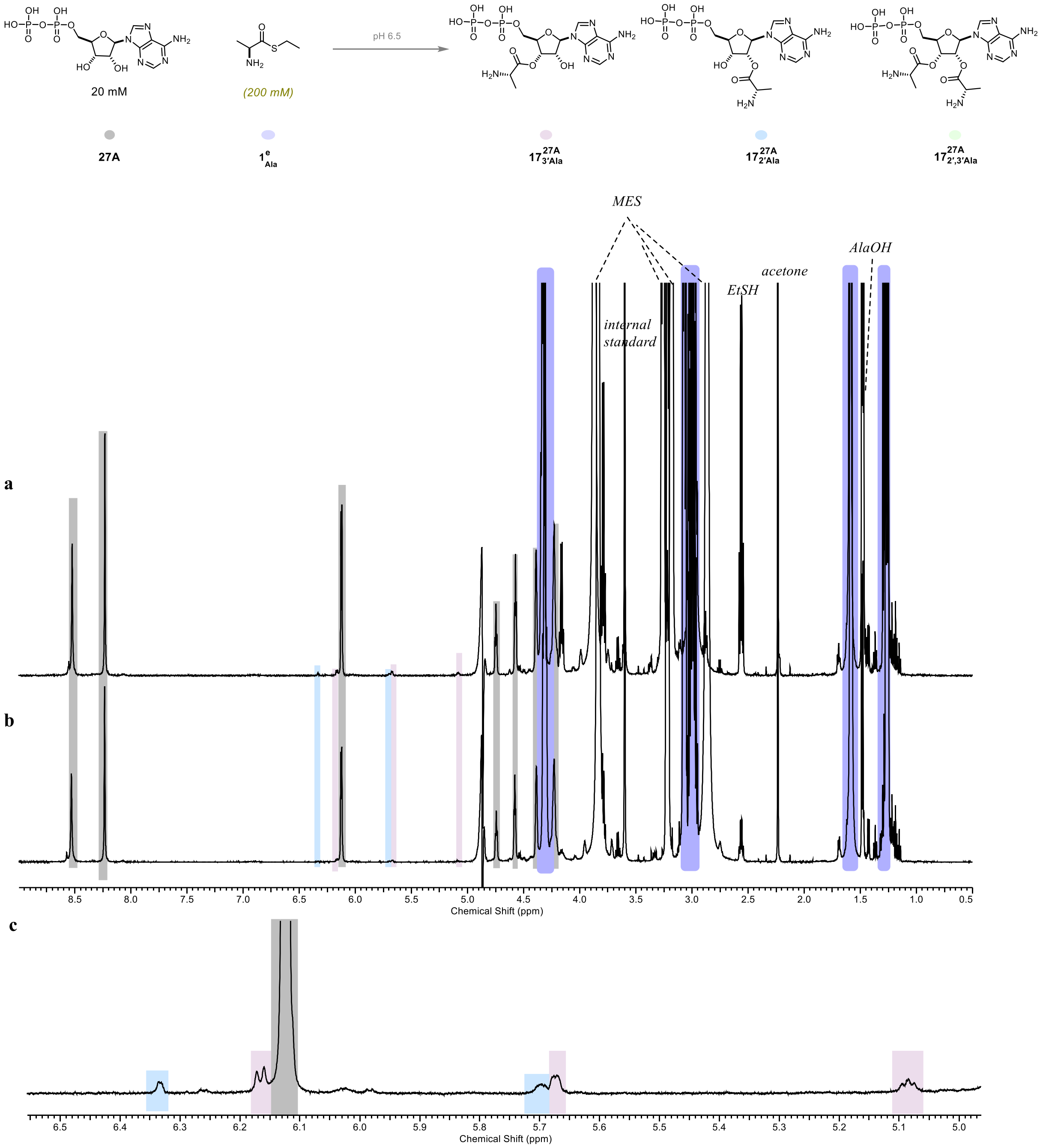

*Supplementary Figure 61. ^{1}H NMR (600 MHz, H_2O/D_2O 9:1, noesygppr1d, 0.5 – 9.0 ppm) spectra to show the reaction of thioester $\mathbf{1^{e}_{Ala}}$ (200 mM) with **27A** (20 mM) at pH 6.5, using pentaerythritol (10 mM) as an internal standard and MES buffer. Set up following General Procedure C after: **a)** 24 h; **b)** 1 h; **c)** zoom-in of 24-hour timepoint (spectrum **a**) from 5.0 ppm – 6.5 ppm.*

^{1}H NMR (600 MHz, H_2O/D_2O 9:1) $\mathbf{17^{27A}_{3'Ala}}$ (partial assignment) : δ_H 6.16 (1H, d, J = 7.3 Hz, (C1')-H), 5.67(1H, app. d, J = 4.7 Hz, (C3')-H), 5.08 (1H, app. t, J = 6.3 Hz, (C2')-H).

^{1}H NMR (600 MHz, H_2O/D_2O 9:1) $\mathbf{17^{27A}_{2'Ala}}$ (partial assignment) : δ_H 6.33 (1H, app. s, (C1')-H), 5.70 (1H, unresolved, (C2')-H).

*Aminoacylation of nucleoside **28A** with aminoacyl thiol $\mathbf{1_{Ala}}$ at pH 6.5*

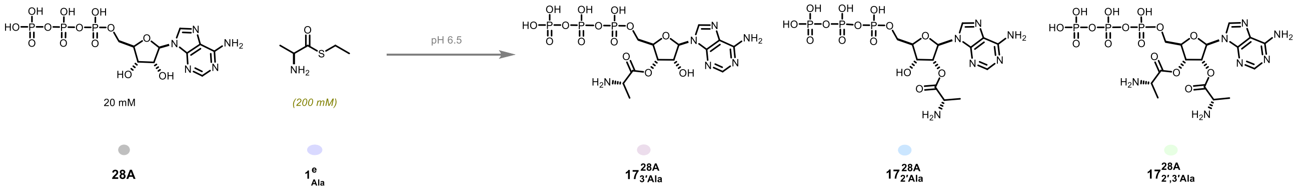

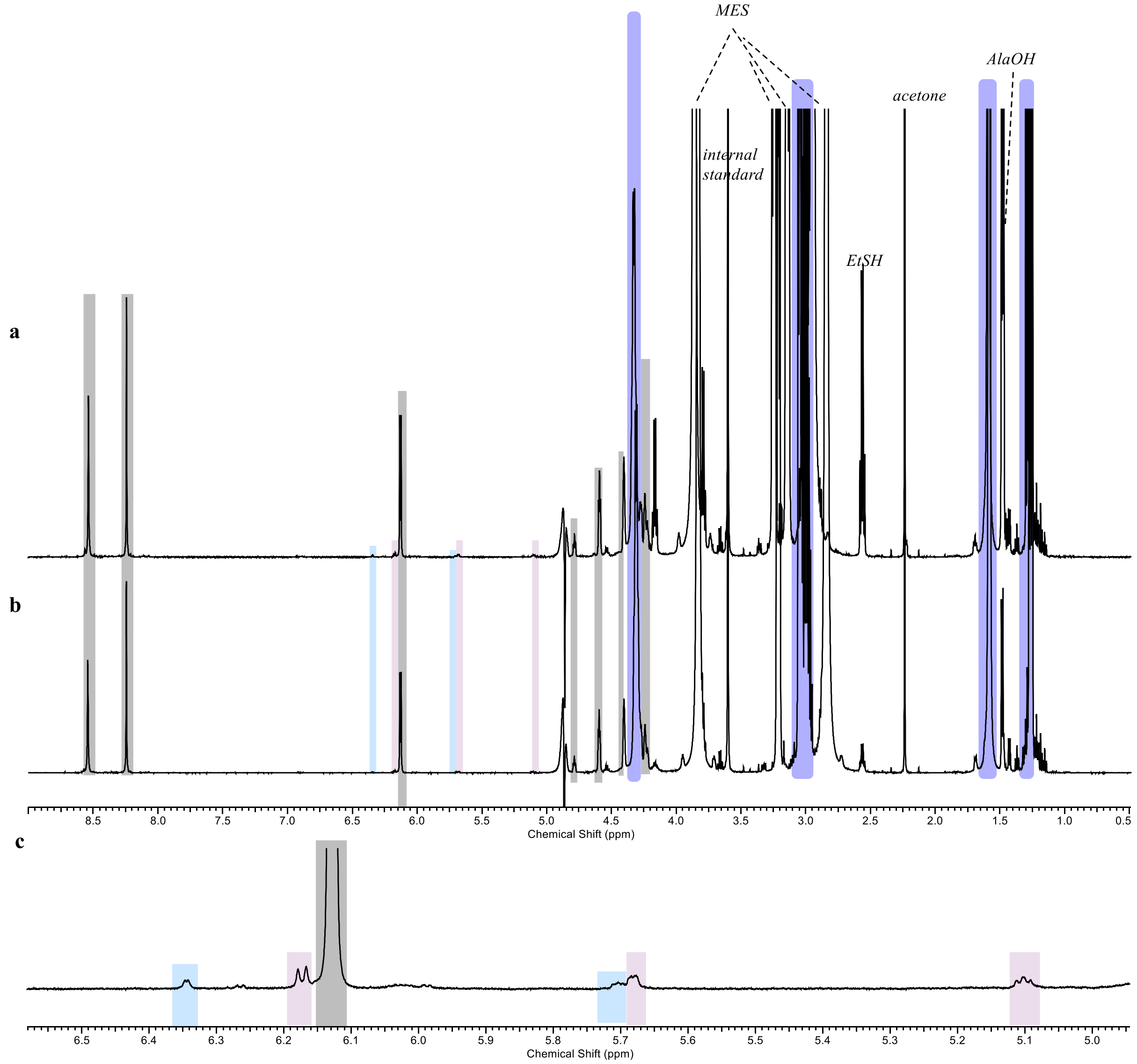

*Supplementary Figure 62. ^{1}H NMR (600 MHz, H_2O/D_2O 9:1, noesygppr1d, 0.5 – 9.0 ppm) spectra to show the reaction of thioester $\mathbf{1^{e}_{Ala}}$ (200 mM) with **28A** (20 mM) at pH 6.5, using pentaerythritol (10 mM) as an internal standard and MES buffer. Set up following General Procedure C after: **a**) 24 h; **b**) 1 h; **c)** zoom-in of 24-hour timepoint (spectrum **a**) from 5.0 ppm – 6.5 ppm.*

^{1}H NMR (600 MHz, H_2O/D_2O 9:1) $\mathbf{17^{28A}_{3'Ala}}$ (partial assignment) : δ_H 6.17 (1H, d, *J* = 7.3 Hz, (C1')-H), 5.68 (1H, app. d, *J* = 5.2 Hz, (C3')-H), 5.10 (1H, app. t, *J* = 6.3 Hz, (C2')-H).

^{1}H NMR (600 MHz, H_2O/D_2O 9:1) $\mathbf{17^{28A}_{2'Ala}}$ (partial assignment) : δ_H 6.33 (1H, d, *J* =3.2 Hz, (C1')-H), 5.70 (1H, unresolved, (C2')-H).

*Aminoacylation of nucleoside **29A** with aminoacyl thiol $\mathbf{1_{Ala}}$ at pH 6.5*

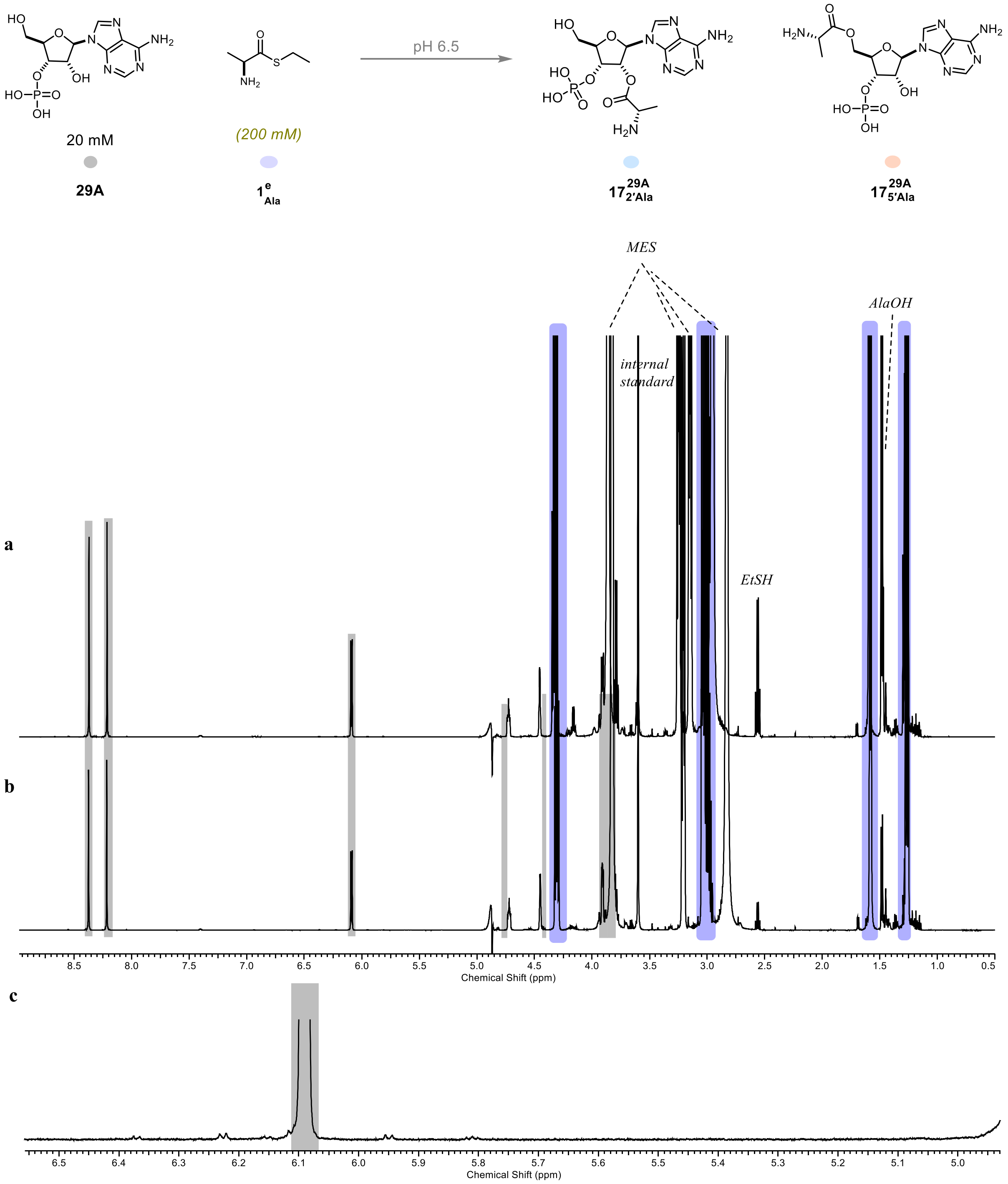

*Supplementary Figure 63. ^{1}H NMR (600 MHz, H_2O/D_2O 9:1, noesygppr1d, 0.5 – 9.0 ppm) spectra to show the reaction of thioester $\mathbf{1^e_{Ala}}$ (200 mM) with **29A** (20 mM) at pH 6.5, using pentaerythritol (10 mM) as an internal standard and MES buffer. Set up following General Procedure C after: **a)** 24 h; **b)** 1 h; **c)** zoom-in of 24-hour timepoint (spectrum **a**) from 5.0 ppm – 6.5 ppm. Diagnostic signals for 2'-aminoacylation are not visible.*

*Aminoacylation of nucleoside **30A** with aminoacyl thiol $\mathbf{1_{Ala}}$ at pH 6.5*

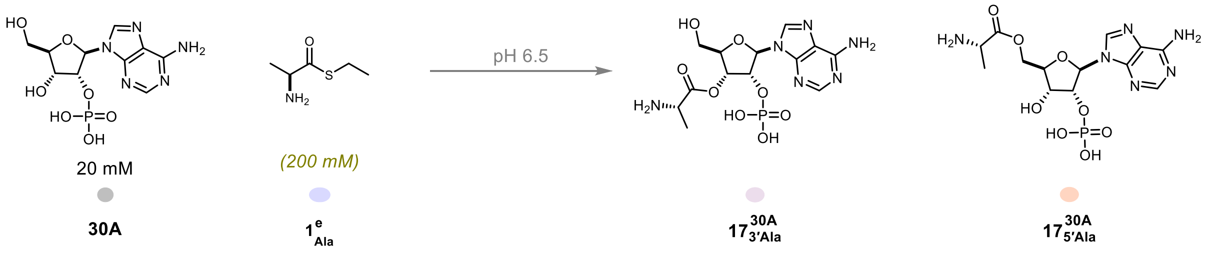

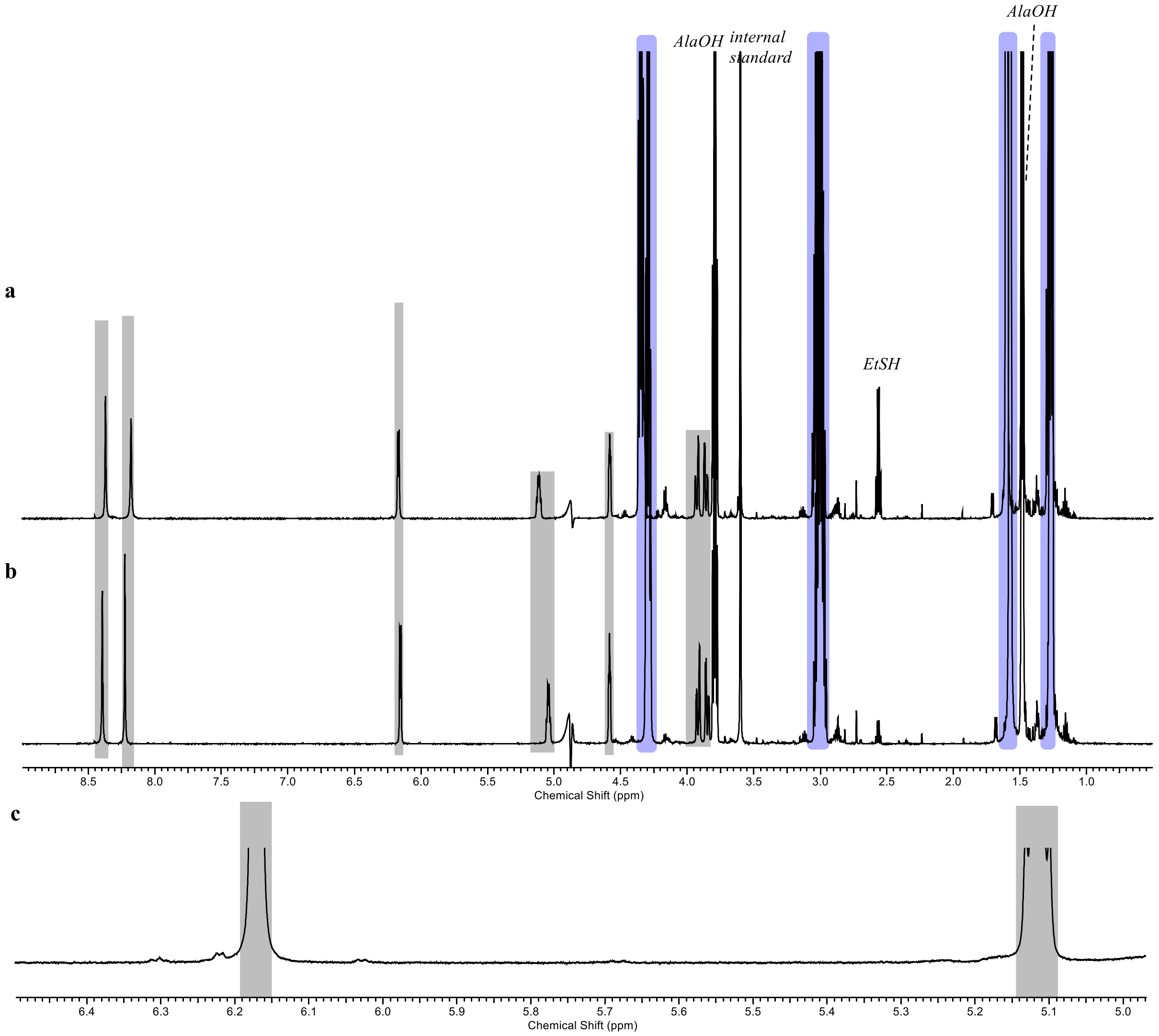

*Supplementary Figure 64. ¹H NMR (600 MHz, H_2O/D_2O 9:1, noesygppr1d, 0.5 – 9.0 ppm) spectra to show the reaction of thioester $\mathbf{1^e_{Ala}}$ (200 mM) with **30A** (20 mM) at pH 6.5, using pentaerythritol (10 mM) as an internal standard. Set up following General Procedure C after: **a)** 24 h; **b)** 1 h; **c)** zoom-in of 24-hour timepoint (spectrum **a)** from 5.0 ppm – 6.5 ppm. Diagnostic signals for 3'-aminoacylation are not visible.*

*Aminoacylation of nucleoside **31A** with aminoacyl thiol **5**$_{\mathbf{Ala}}$ at pH 6.5*

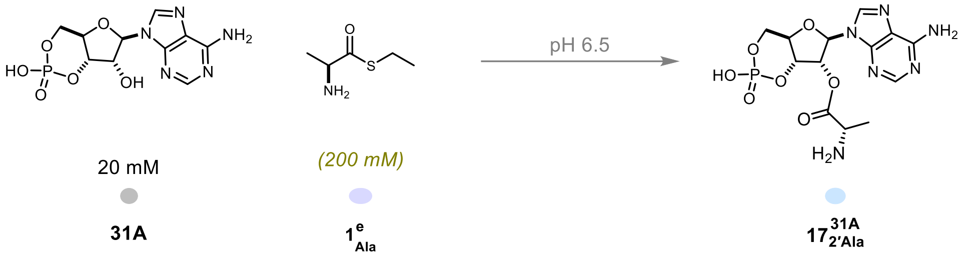

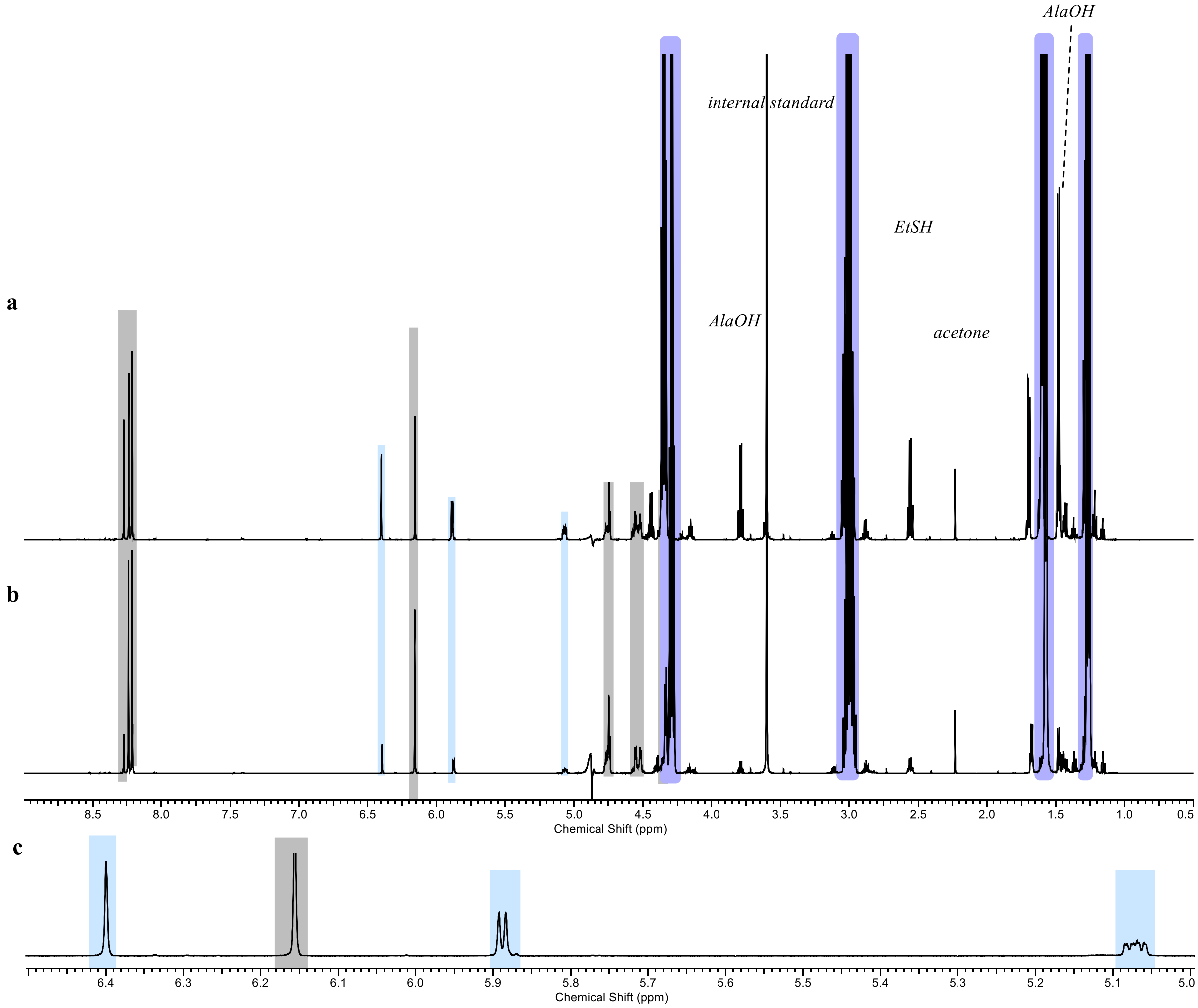

Supplementary Figure 65. ¹H NMR (600 MHz, H₂O/D₂O 9:1, noesygppr1d, 0.5 – 9.0 ppm) spectra to show the reaction of thioester $\mathbf{1^{e}_{Ala}}$ *(200 mM) with **31A** (20 mM) at pH 6.5, using pentaerythritol (10 mM) as an internal standard. Set up following General Procedure C after: **a)** 24 h; **b)** 1 h; **c)** zoom-in of 24-hour timepoint (spectrum **a)** from 5.0 ppm – 6.5 ppm.*

^{1}H NMR (600 MHz, H_2O/D_2O 9:1) $\mathbf{17^{31A}_{2'Ala}}$ (partial assignment) : δ_H 8.27 (1H, s, (C2)-H), 6.40 (1H, s, (C1')-H), 5.89 (1H, d, J = 5.2 Hz, (C2')-H), (1H, ddd, J = 9.2, 5.2, 1.8 Hz, (C3')-H).

*Aminoacylation of nucleoside **32A** with aminoacyl thiol $\mathbf{1_{Ala}}$ at pH 6.5*

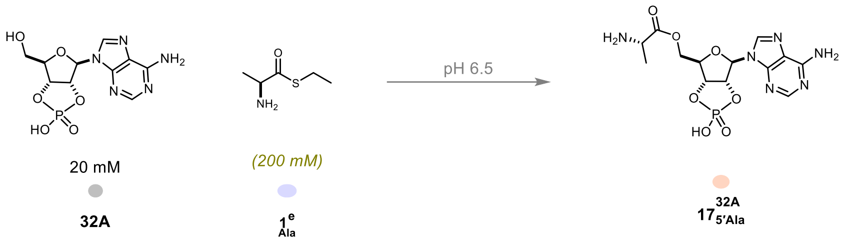

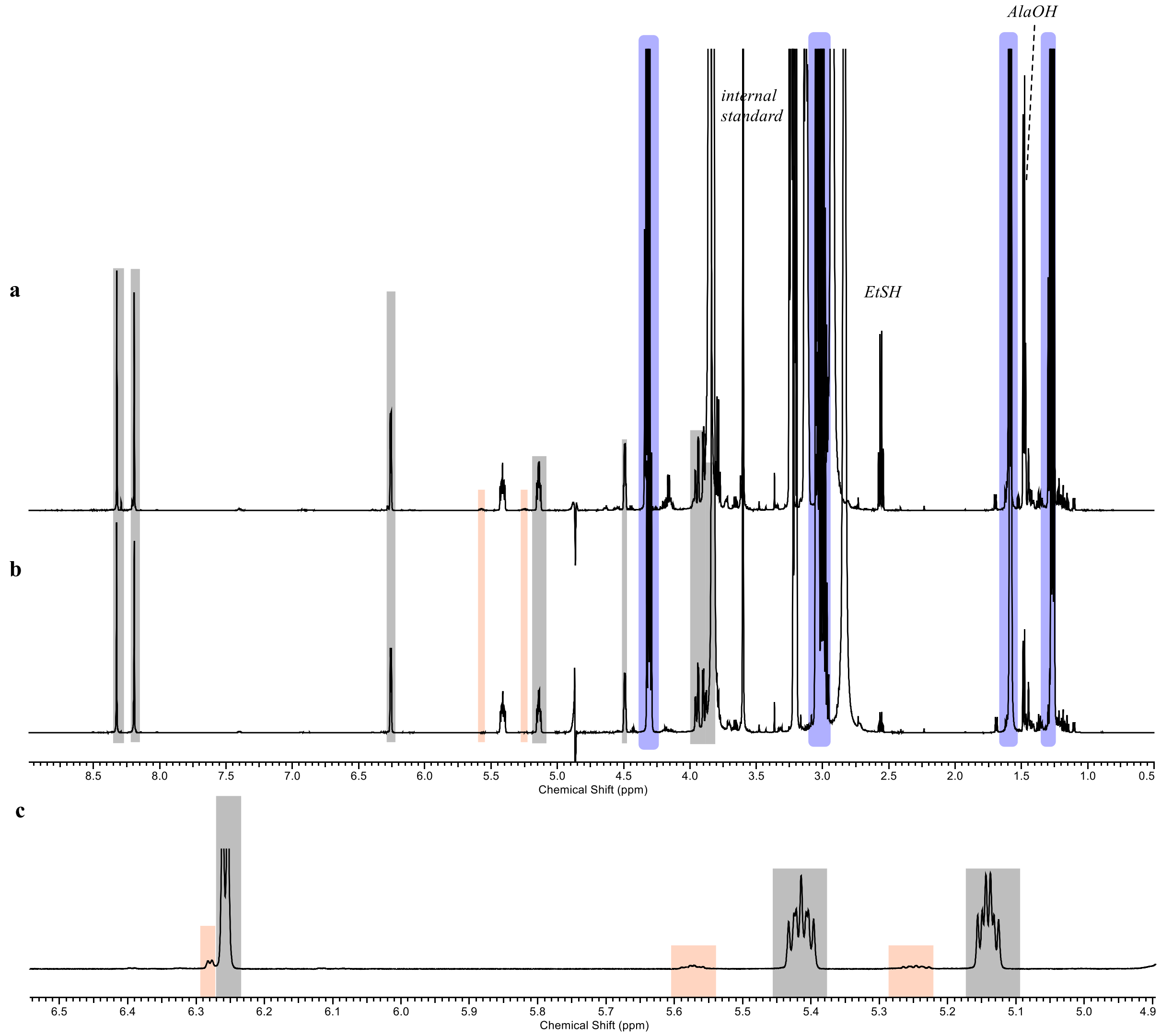

*Supplementary Figure 66. ¹H NMR (600 MHz, H_2O/D_2O 9:1, noesygppr1d, 0.5 – 9.0 ppm) spectra to show the reaction of thioester $\mathbf{1^{e}_{Ala}}$ (200 mM) with **32A** (20 mM) at pH 6.5, using pentaerythritol (10 mM) as an internal standard and MES buffer. Set up following General Procedure C after: **a**) 24 h; **b**) 1 h; **c)** zoom-in of 24-hour timepoint (spectrum **a**) from 4.9 ppm – 6.5 ppm.*

¹H NMR (600 MHz, H_2O/D_2O 9:1) $\mathbf{17^{32A}_{5'Ala}}$ (partial assignment) : δ_H 6.28 (1H, d, *J* = 3.3 Hz, (C1')-H), 5.57 (1H, app. td, *J* = 7.5, 3.3 Hz, (C2')-H), 6.28 (1H, ddd, *J* = 10.6, 6.6 5.3 Hz, (C3')-H).

*Aminoacylation of nucleoside **49U** with aminoacyl thiol $\mathbf{1_{Ala}}$ at pH 6.5*

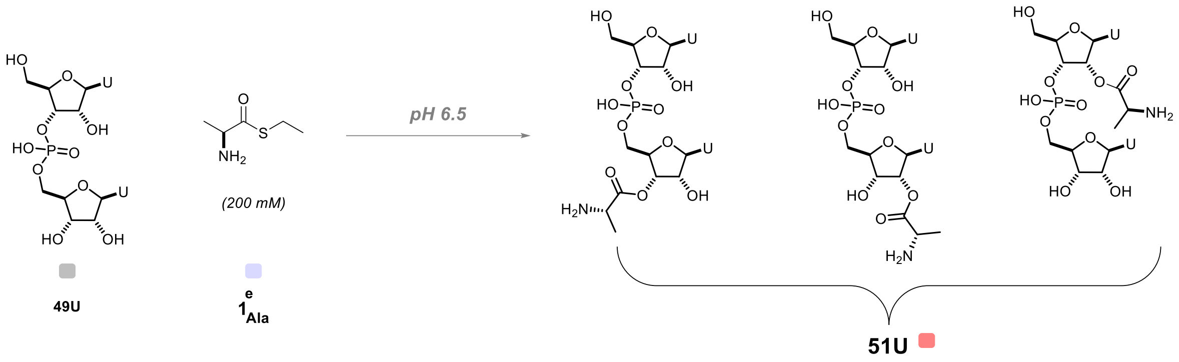

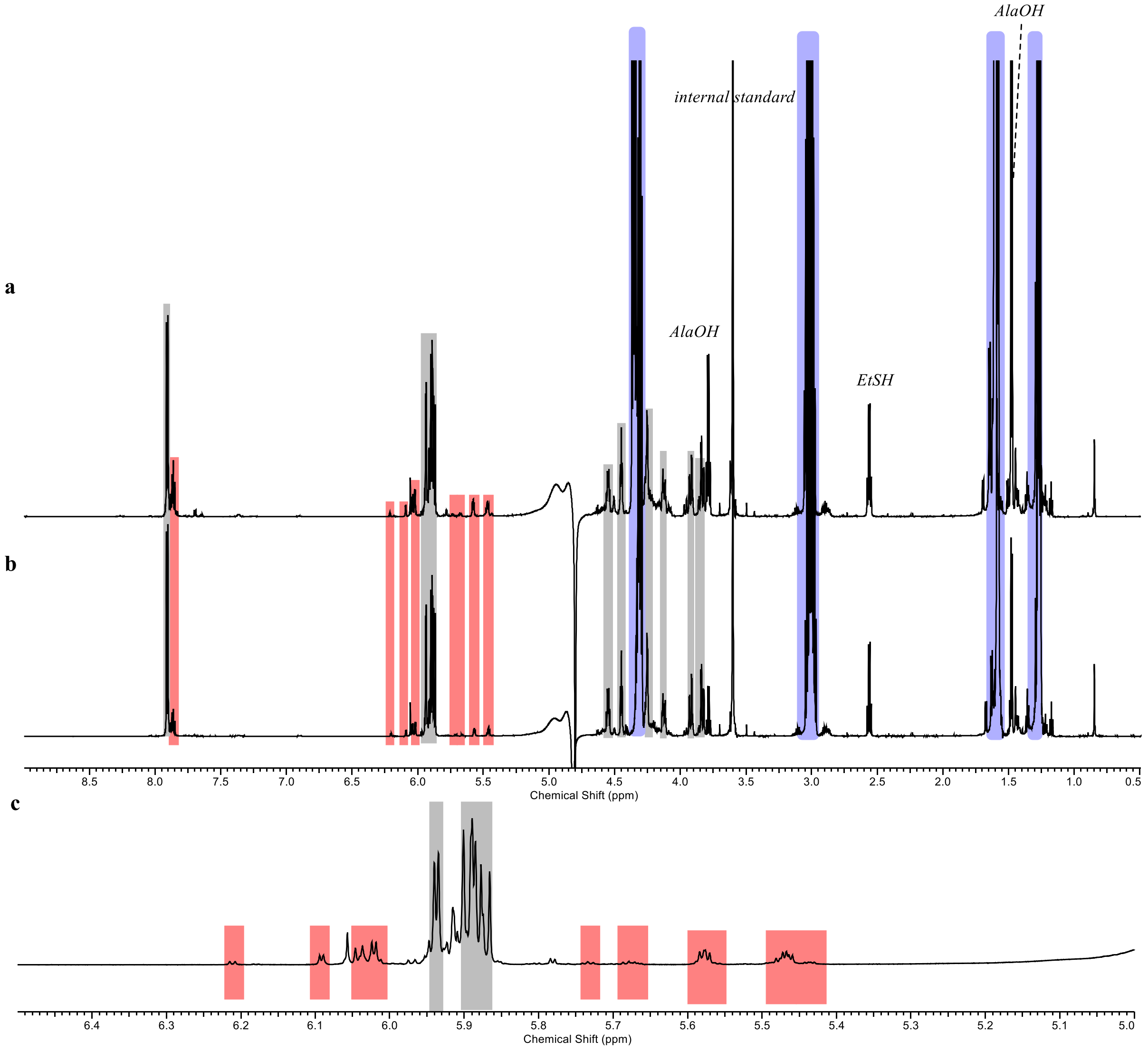

*Supplementary Figure 67. ^{1}H NMR (600 MHz, H_2O/D_2O 9:1, noesygppr1d, 0.5 – 9.0 ppm) spectra to show the reaction of thioester $\mathbf{1^e_{Ala}}$ (200 mM) with **49U** (20 mM) at pH 6.5, using pentaerythritol (10 mM) as an internal standard. Set up following General Procedure C after: **a)** 24 h; **b)** 2 h; **c)** zoom-in of 24-hour timepoint (spectrum **a)**) from 5.0 ppm – 6.5 ppm. Note – due to signal overlap it was not possible to individually quantify the separate aminoacylated regioisomers.*

*Aminoacylation of nucleoside **15A** with aminoacyl thiol **1**$_{Ala}$ at pH 6.5*

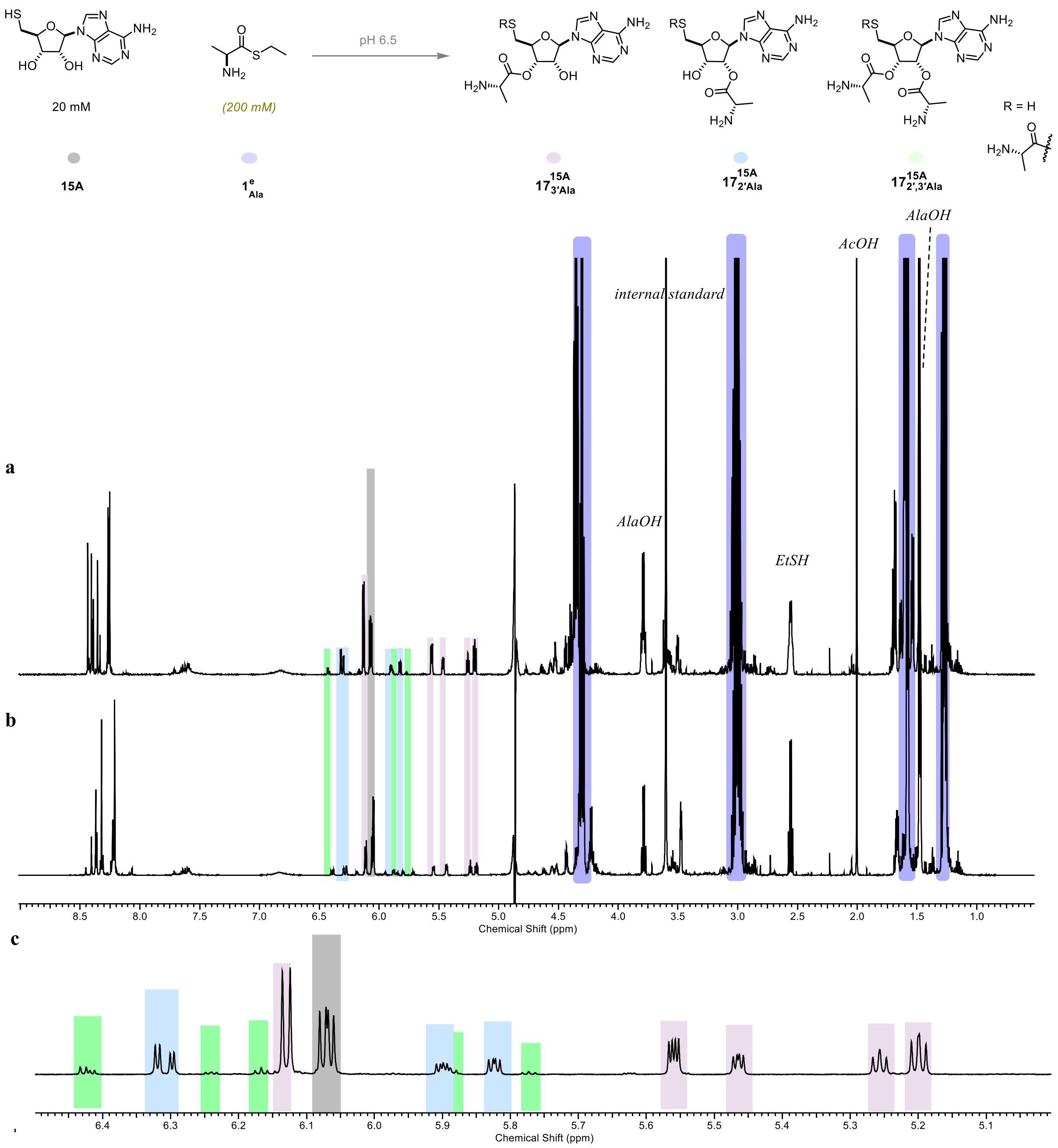

*Supplementary Figure 68. ^{1}H NMR (600 MHz, H_2O/D_2O 9:1, noesygppr1d, 0.5 – 9.0 ppm) spectra to show the reaction of thioester $\mathbf{1^e_{Ala}}$ (100 mM) with **15A** (20 mM) at pH 6.5, using pentaerythritol (10 mM) as an internal standard. Set up following General Procedure C after: **a)** 24 h; **b)** 1 h; **c)** zoom-in of 24-hour timepoint (spectrum **a**) from 5.0 ppm – 6.5 ppm.*

Note - each alcohol aminoacylation product is an equilibrating mixture of 5'-thiol and 5'-aminoacylthio-ester products. It was not possible to unambiguously assign which resonances belong to the thiol and which to the thioester, and so diol aminoacylation products are reported as a mixture of thiol and thioester.

^{1}H NMR (600 MHz, H_2O/D_2O 9:1) $\mathbf{17^{15A}_{3'Ala}}$ (partial assignment) : δ_H 6.13 (1H, d, *J* = 6.9 Hz, (C1')-H), 5.56 (1H, dd, *J* = 5.7, 3.0 Hz, (C3')-H, *major*), 5.46 (1H, dd, *J* = 5.4, 3.3 Hz, (C3')-H, *minor*), 5.26 (1H, app. t, *J* = 6.0 Hz, (C2')-H, *minor*), 5.20 (1H, dd, *J* = 6.9, 5.9 Hz, (C2')-H, *major*).

^{1}H NMR (600 MHz, H_2O/D_2O 9:1) $\mathbf{17^{15A}_{2'Ala}}$ (partial assignment) : δ_H 6.32 (1H, d, *J* = 4.0 Hz, (C1')-H, *major*), 6.29 (1H, d, *J* = 3.4 Hz, (C1')-H, *minor*), 5.90 (1H, dd, *J* = 5.9, 3.4 Hz, (C2')-H, *minor*), 5.82 (1H, dd, *J* = 5.9, 4.0 Hz, (C2')-H, *major*).

^{1}H NMR (600 MHz, H_2O/D_2O 9:1) $\mathbf{17^{15A}_{2'3'Ala}}$ (partial assignment) : δ_H 6.42 (1H, d, *J* = 5.2 Hz, (C1')-H, *major*), 6.41 (1H, d, *J* = 4.0 Hz, (C1')-H, *minor*), 6.24 (1H, dd, *J* = 5.2, 4.4 Hz, (C2')-H, *minor*), 6.17 (1H, app. t, *J* = 5.4 Hz, (C2')-H, *major*), 5.77 (1H, app. t, *J* = 5.7 Hz, (C3')-H, *minor*).

Time (h)	$\mathbf{17^{15A}_{2'Ala}}$ (%)	$\mathbf{17^{15A}_{3'Ala}}$ (%)	$\mathbf{17^{15A}_{2'3'Ala}}$ (%)	Total (%)
24 h	17	34	4	54

Supplementary Table 7. % yields for the reaction of thioester $\mathbf{1^{e}_{Ala}}$ *(100 mM) with* ***15A*** *(20 mM) at pH 6.5 after 24 h.*

Aminoacylation of nucleosides ***16C*** *and* ***15A*** *with aminoacyl thiol* $\mathbf{1_{Ala}}$ *at pH 6.5*

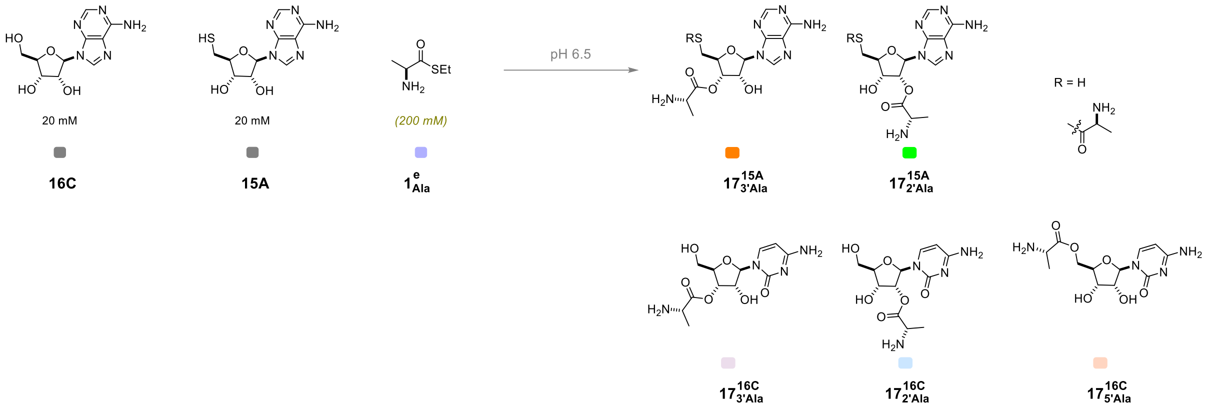

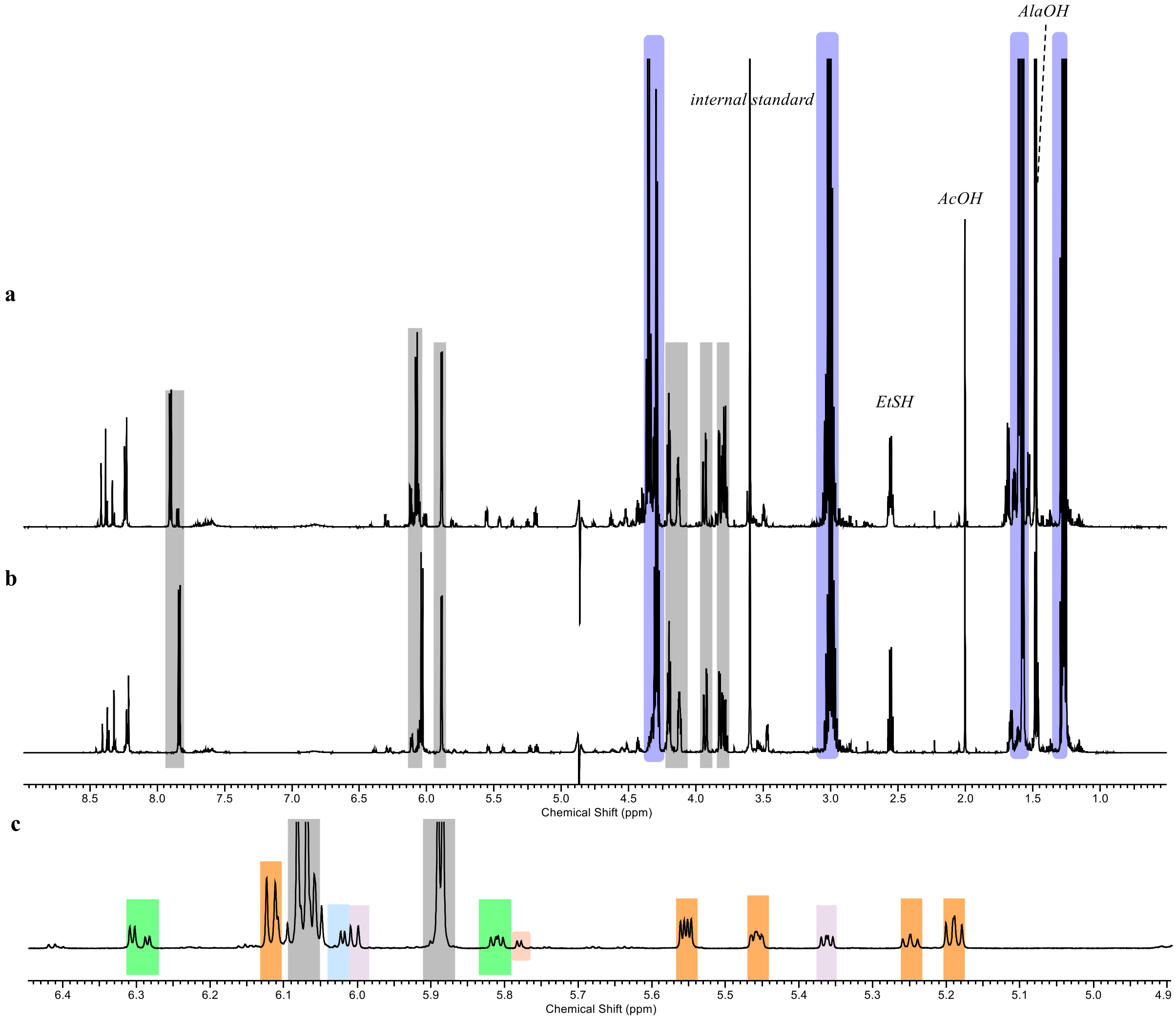

Supplementary Figure 69. ^{1}*H NMR (600 MHz,* H_2O/D_2O *9:1, noesygppr1d, 0.5 – 9.0 ppm) spectra to show the reaction of thioester* $\mathbf{1^{e}_{Ala}}$ *(100 mM) with* ***15A*** *(15 mM) and* ***16C*** *(20 mM) at pH 6.5, using pentaerythritol (10 mM) as an internal standard. Set up following General Procedure C after:* ***a)*** *24 h;* ***b)*** *1 h;* ***c)*** *4.9 ppm – 6.5 ppm region of spectrum A).*

*Aminoacylation of uridine (**16U**) with aminoacyl thiol **1**$_{\mathbf{Ala}}$ at different pHs*

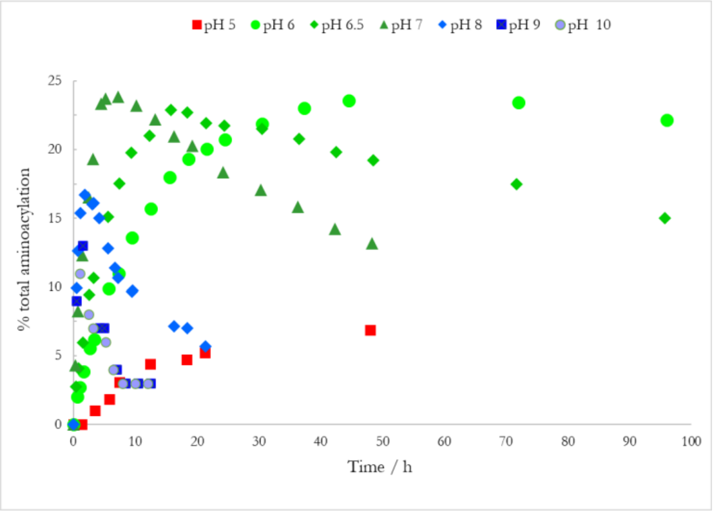

*Supplementary Figure 70. Total aminoacylation (%) of **16U** (20 mM) in the presence of* $\mathbf{1}^{\mathbf{e}}_{\mathbf{Ala}}$ *(200 mM) in 1 M buffer with PET (20 mM) as an internal standard. pH 5, 6, 6.5: 1 M MES buffer. pH 7, 8: 1 M MOPS. pH 9, 10: 1 M Pyrophosphate buffer. Set up following General Procedure C.*

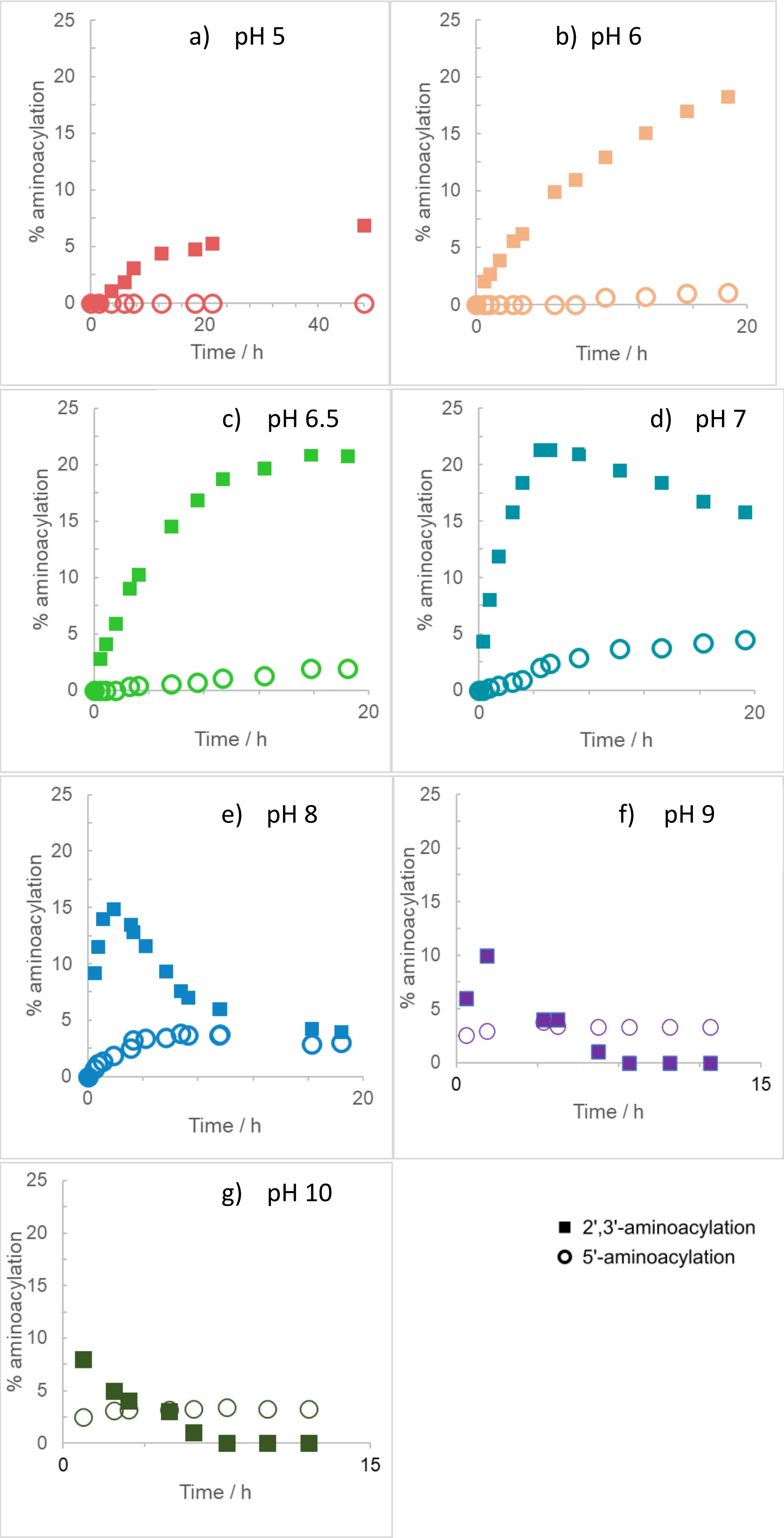

Supplementary Figure 71. ^{1}H NMR yields (%) of aminoacylation on the 2'- or 3'-diol of ***16U*** *(*$\mathbf{17^{U}_{3'Ala}}$*,* $\mathbf{17^{U}_{2'Ala}}$*,* $\mathbf{17^{U}_{2'3'Ala}}$*, ■), and on the 5'-alcohol of* ***16U*** *(*$\mathbf{17^{U}_{5'Ala}}$*,○) from the reaction of* $\mathbf{1^{e}_{Ala}}$ *(200 mM) with* ***16U*** *(20 mM) using PET (15 mM) as an internal standard in A) MES buffer (1 M, pH 5.0); B) MES buffer (1 M, pH 6.0); C) MES buffer (1 M, pH 6.5); D) MOPS buffer (1 M, pH 7.0); E) MOPS buffer (1 M, pH 8.0); F) Pyrophosphate buffer (1 M, pH 9); G) Pyrophosphate buffer (1 M, pH 10). Set up following General Procedure C.*

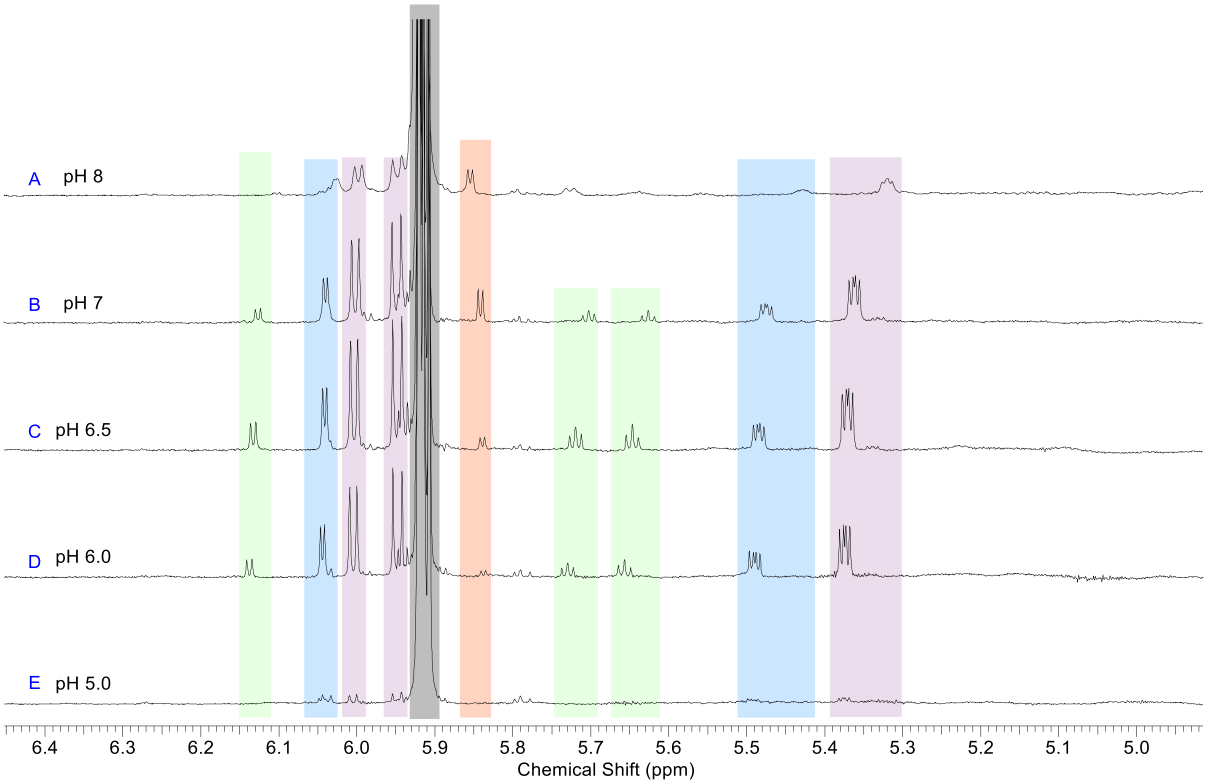

Supplementary Figure 72. ¹H NMR (700 MHz, H_2O/D_2O 98:2, noesygppr1d, 4.9 – 6.4 ppm) spectra to show the buffered reaction of thioester $\mathbf{1}^{e}_{Ala}$ *(200 mM) with uridine (**16U**, 20 mM) using PET (15 mM) as an internal standard after 12 h in A) MOPS buffer (1 M, pH 8); B) MOPS buffer (1 M, pH 7.5); C) MES buffer (1 M, pH 7.0); D) MES buffer (1 M, pH 6.5); E) MES buffer (1 M, pH 5.0). Set up following General Procedure C.*

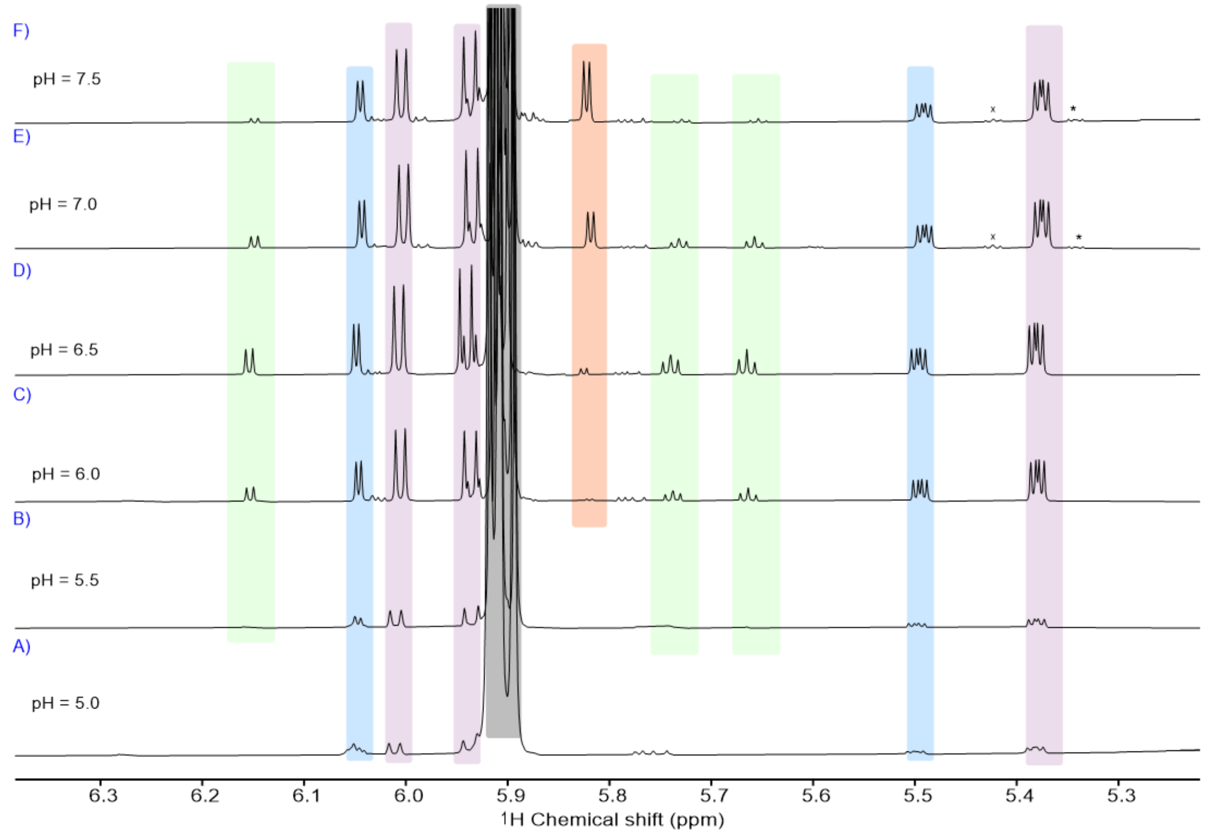

Supplementary Figure 73: ^{1}H NMR (600 MHz, H_2O/D_2O 9:1, noesygppr1d, 5.3 – 6.3 ppm) spectra to show the unbuffered reaction of thioester $\mathbf{1^{e}_{Ala}}$ *(200 mM) with uridine (**16U**, 20 mM) with PET (10 mM) as an internal standard after 24 hours at: A) pH 7.5; B) pH 7.0; C) pH 6.5; D) pH 6.0; E) pH 5.5; F) pH 5.0. Set up following General Procedure C. * = At higher pHs, approximately 1 - 2% racemization was observed. Tentatively assigned X=3',5' -bis amino-acylated product was observed.*

*Diketopiperazine **6**$_{AlaAla}$ formation at different pHs*

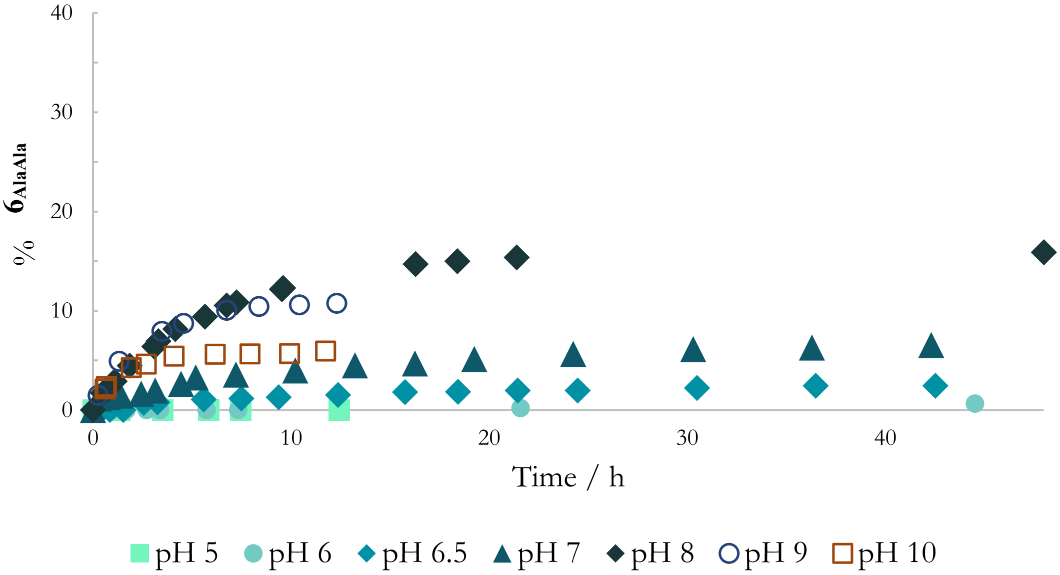

*Supplementary Figure 74. Alanyl diketopiperazine **6**$_{AlaAla}$ formation (%) as determined by ^{1}H NMR spectroscopy plotted against time in the reaction of **16U** (20 mM) with L-**1**$^{e}_{Ala}$ (200 mM) in 1 M buffer with PET (20 mM) as an internal standard. pH 5, 6, 6.5: 1 M MES buffer; pH 7, 8: 1 M MOPS buffer; pH 9, 10: 1M pyrophosphate buffer, L-**1**$^{e}_{Ala}$ (190 mM). Set up following General Procedure C.*

*Aminoacylation of uridine (**16U**) with thioester $\mathbf{1_{Ala}}$ at different nucleoside concentrations at pH 6.5*

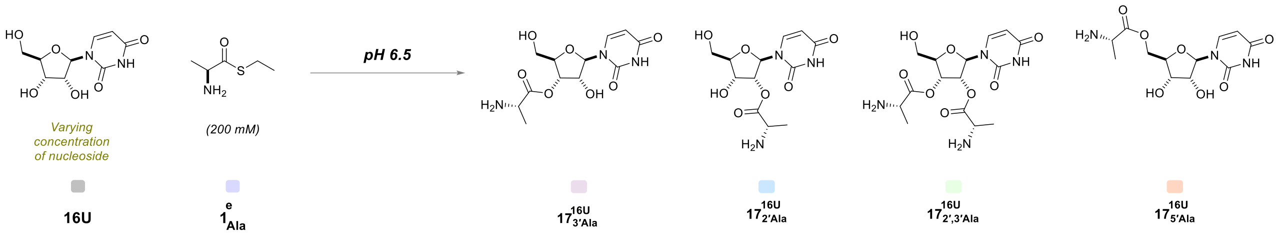

Entry	Time (hours)	**16U** (mM)	$\mathbf{17_{3'Aaa}}$ (%)	$\mathbf{17_{2'Aaa}}$ (%)	$\mathbf{17_{2',3'Aaa}}$ (%)	$\mathbf{17_{5'Aaa}}$ (%)	**Total** (%)
1	18	2	13	6	4	--	23
2	18	20	14	7	3	1	25
3	18	100	11	7	2	2	22

*Supplementary Table 8: Yields of 2'-, 3'-, 2',3'- and 5'-aminoacyl-nucleoside $\mathbf{17_{Ala}}$ after the reaction of specified concentration of uridine (**16U**) with 200 mM alanine thioester $\mathbf{1^{e}_{Ala}}$ at room temperature. Set up following General Procedure C.*

*Aminoacylation of uridine (**16U**) with different concentrations of thioester **1_{Ala}***

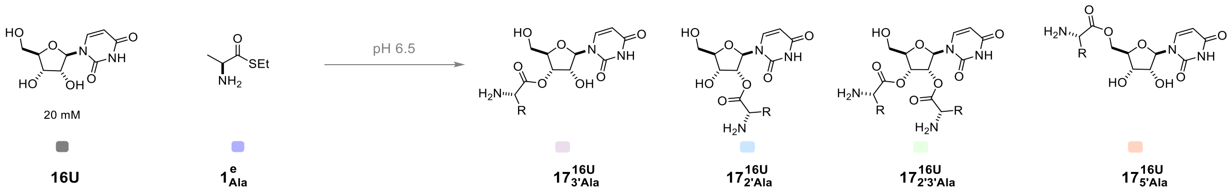

Entry	1^{e}_{Ala} (mM)	$17_{3'Aaa}$ (%)	$17_{2'Aaa}$ (%)	$17_{2',3'Aaa}$ (%)	$17_{5'Aaa}$ (%)	Total (%)
1	10	1	--	--	--	1
2	20	2	1	--	--	3
3	100	8	4	1	--	13
4	200	14	7	4	2	26
5*	400	21	11	6	2	40
6*	600	27	12	9	2	50

*Supplementary Table 9: Yields of 2'-, 3'-, 2',3'- and 5'-aminoacyl-nucleoside **17_{Ala}** after the reaction of specified concentration of alanine thioester **1^{e}_{Ala}** with 20 mM uridine (**16U**) at 6.5 and after 24 hours at room temperature. Set up following General Procedure C. *Racemization (~2%) was observed. X is tentatively assigned as 3',5' and 2',5'-bis amino-acylated product. The numbers for 2',3',5'-aminoacylation are not included in the above table.*

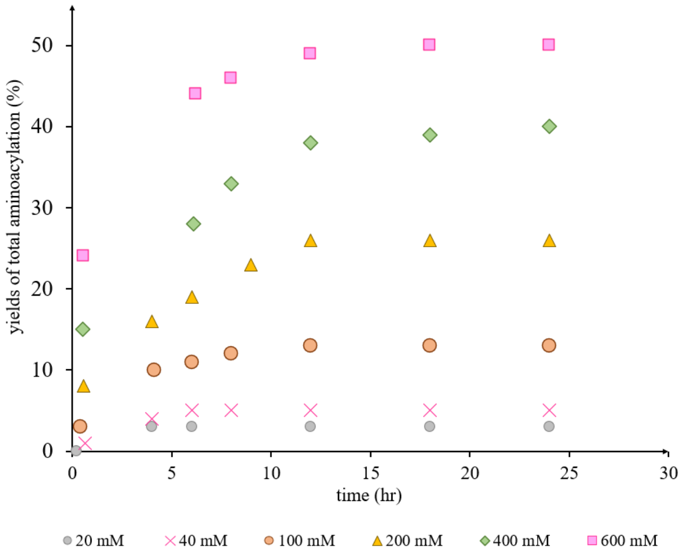

*Supplementary Figure 75: Aminoacylation yields (%) over time for the reaction of uridine (**16U**, 20 mM) with specified concentration of alanine thioester **1^{e}_{Ala}** at pH 6.5 and room temperature.*

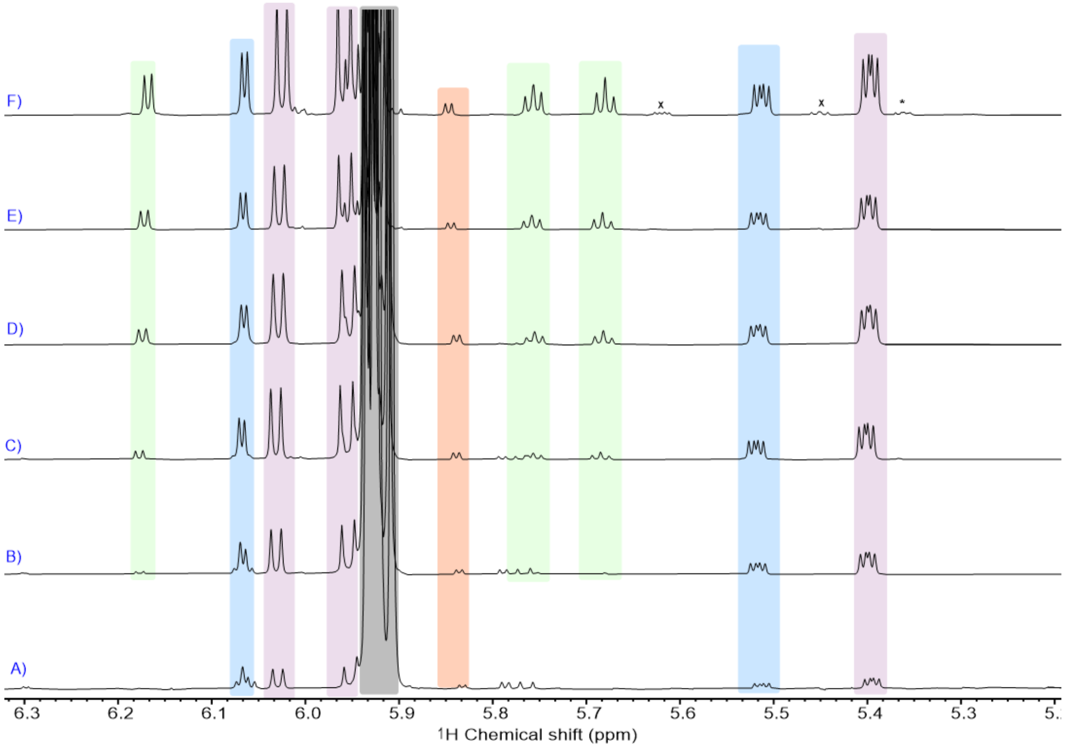

Supplementary Figure 76. ¹H NMR (600 MHz, H_2O/D_2O 9:1, noesygppr1d, 5.2 – 6.3 ppm) spectra to show the different concentration of thioester $\mathbf{1^e_{Ala}}$ *with uridine (**16U**, 20 mM) with PET (10 mM) as an internal standard at pH 6.5 after 24 hours: A) thioester (*$\mathbf{1^e_{Ala}}$*, 20 mM); B) thioester (*$\mathbf{1^e_{Ala}}$*, 40 mM); C) thioester (*$\mathbf{1^e_{Ala}}$*, 100 mM); D) thioester (*$\mathbf{1^e_{Ala}}$*, 200 mM); E) thioester (*$\mathbf{1^e_{Ala}}$*, 400 mM); F) thioester (*$\mathbf{1^e_{Ala}}$*, 600 mM). Set up following General Procedure C. * = At higher concentrations, < 2% racemization was observed. X is tentatively assigned as 3',5' and 2',5'-bis amino-acylated products.*

*Side chain compatibility for aminoacylation of uridine (**16U**) by thioesters **1**$_{Aaa}$*

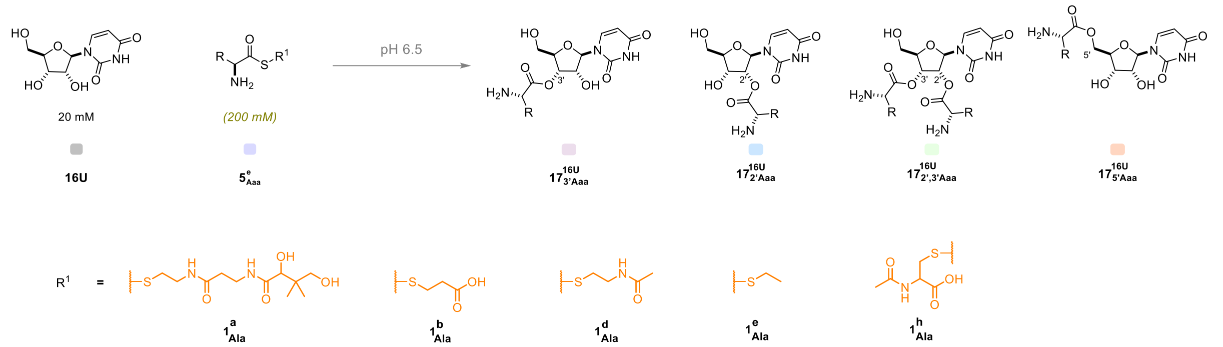

Entry	1_{Aaa} (R) (mM)	1 (R¹) (side chain)	Time (h)	$17_{3'Aaa}$ (%)	$17_{2'Aaa}$ (%)	$17_{2',3'Aaa}$ (%)	$17_{5'Aaa}$ (%)	Total (%)
1	Gly (220)	1^e_{Ala}	24	11	5	3	1	20
2	L-Ala (200)	1^e_{Ala}	24	14	7	3	1	26
3	L-Ala (200)	1^h_{Ala}	24	13	7	--	2	22
4	L-Ala (200)	1^b_{Ala}	24	9	4	1	--	14
5	L-Ala (200)	1^d_{Ala}	24	17	9	3	1	30
6	L-Ala (200)	1^a_{Ala}	24	17	8	3	1	29
7	L-Leu (210)	1^e_{Ala}	24	15	7	3	2	27
8	L-Pro (200)	1^e_{Ala}	24	13	8	2	1	24
9	L-Glu (190)	1^e_{Ala}	24	8	4	1	1	14
10*	L-Gln (185)	1^e_{Ala}	24	8	4	--	1	13
11	L-Val (220)	1^e_{Ala}	24	4	2	--	--	6
12#	L-Phe (150)	1^e_{Ala}	24	6	4	--	--	10
13	L-Lys (220)	1^e_{Ala}	24	14	7	2	1	24
14	L-Arg (220)	1^e_{Ala}	6	40	21	2	2	65
15	L-His (180)	1^e_{Ala}	24	7	4	--	1	12
16	L-Ser (200)	1^e_{Ala}	24	6	3	--	--	9
17	L-Pip (180)	1^e_{Ala}	18	11	6	1	1	19
18	L-Met (210)	1^e_{Ala}	24	11	5	2	--	18

*Supplementary Table 10: Total yields for a-aminoacyl ester observed in the reaction of thioester (1^e_{Aaa}, 180 - 230 mM) and uridine (**16U**, 20 mM) with pentaerythritol (10 mM, internal standard) at pH 6.5 and room temperature, unless stated otherwise. Set up following General Procedure C. # -thioester (1^e_{Phe}, 150 mM) and uridine (**16U**, 20 mM) in MES buffer (400 mM, pH 6.5) using PET (30 mM) as an internal standard.*

*During the reaction of Gln-thioester (1^e_{Gln}, 185 mM) and uridine **16U** (20 mM) in MES buffer (200 mM) with pentaerythritol (20 mM, internal standard) at pH 6.5 and room temperature, cyclisation of Gln-thioester 1^e_{Gln} (10%) to pyroglutamate thioester $1^e_{PyroGln}$ (~10%) was observed (Supplementary Figure 82). $1^e_{PyroGln}$ was not observed to lead to an addition nucleoside acylation product further demonstrating that under these conditions a free α-amine is essential for nucleoside aminoacylation.

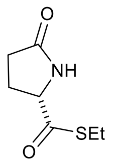

$1^e_{pyroGln}$

*High-resolution mass spectrometry data for aminoacylation of uridine (**17U**)*

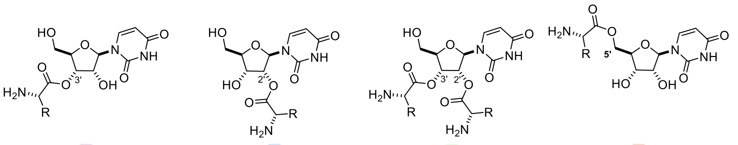

$\mathbf{18_{3'Aaa}}$ $\mathbf{18_{2'Aaa}}$ $\mathbf{18_{2',3'Aaa}}$ $\mathbf{18_{5'Aaa}}$

Entry	$\mathbf{18^{U}_{Aaa}}$	HRMS-ESI for Aminoacylation RNAs		
		Formula	Theoretical	Found
1	Gly	$C_{11}H_{15}N_3O_7$ [M+H]$^+$	302.0983	302.0978
2	(Gly)$_2$	$C_{13}H_{14}N_4O_8$ [M+H]$^+$	359.1197	359.1190
3	Ala	$C_{12}H_{16}N_3O_7$ [M-H]$^-$	314.0994	314.0980
4	(Ala)$_2$	$C_{15}H_{21}N_4O_8$ [M-H]$^-$	385.1348	385.1364
5	Val	$C_{14}H_{22}N_3O_7$ [M+H]$^+$	344.1452	344.1447
6	(Val)$_2$	$C_{19}H_{31}N_4O_8$ [M+H]$^+$	443.2136	443.2130
7	Leu	$C_{15}H_{23}N_3O_7$ [M+H]$^+$	358.1609	358.1611
8	(Leu)$_2$	$C_{21}H_{34}N_4O_8$ [M+H]$^+$	471.2449	471.2445
9	Lys	$C_{15}H_{25}N_4O_7$ [M+H]$^+$	373.1718	373.1703
10	(Lys)$_2$	$C_{21}H_{37}N_6O_8$ [M+H]$^+$	501.2667	501.2647
11	Arg	$C_{15}H_{25}N_4O_7$ [M+Na]$^+$	373.1718	373.1703
12	His	$C_{15}H_{20}N_5O_7$ [M+H]$^+$	382.1357	382.1359
13	(His)$_2$	$C_{21}H_{27}N_8O_8$ [M+H]$^+$	519.1946	519.1950
14	Pro	$C_{14}H_{20}N_3O_7$ [M+H]$^+$	342.1296	342.1285
15	(Pro)$_2$	$C_{19}H_{27}N_4O_8$ [M+H]$^+$	439.1823	439.1811
16	Phe	$C_{18}H_{22}N_3O_7$ [M+H]$^+$	392.1452	392.1433
17	(Phe)$_2$	$C_{27}H_{31}N_4O_8$ [M+H]$^+$	539.2136	539.2115
18	Glu	$C_{14}H_{20}N_3O_9$ [M+H]$^+$	374.1194	374.1186
19	(Glu)$_2$	$C_{19}H_{27}N_4O_{12}$ [M+H]$^+$	503.1620	503.1605

*Supplementary Table 11. HRMS data for aminoacylation of uridine **17U** with aminoacyl-thioester (**5**$_{\mathbf{Aaa}}$) to form **18**$_{\mathbf{3'Aaa}}$/ **18**$_{\mathbf{2'Aaa}}$ (addition of Aaa) or **18**$_{\mathbf{2'3'Aaa}}$ (addition of (Aaa)$_2$).*

Synthesis of aminoacyl-uridine ***17**$^{U}_{Ala}$* *upon incubating uridine (**16U**) with thioester* ***1**$_{Ala}$*

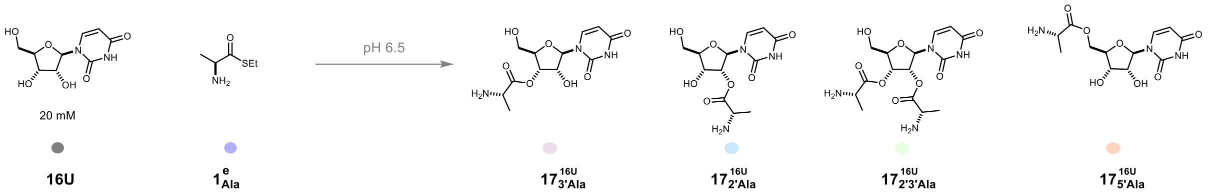

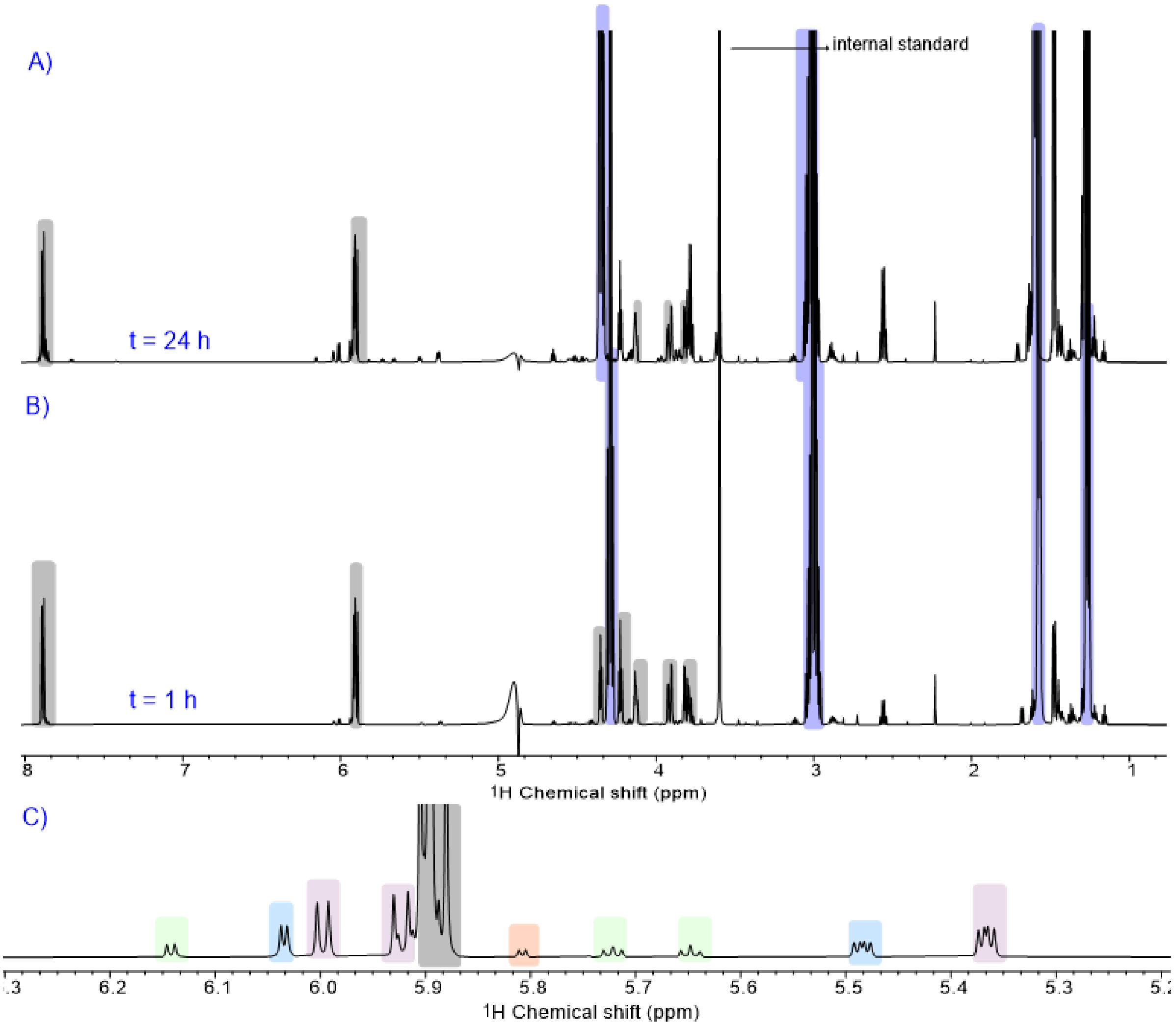

Supplementary Figure 77. ¹H NMR (600 MHz, H₂O/D₂O 9:1, noesygppr1d, 1.0 – 8.0 ppm) spectra to show the reaction of thioester ***1**$^{e}_{Ala}$* *(200 mM) with uridine (**16U**, 20 mM) at pH 6.5, using PET (10 mM) as an internal standard. Set up following General Procedure C, after A) 24 h; B) 1h. C) 5.2 – 6.3 ppm region of spectrum A.*

^{1}H NMR (600 MHz, H_2O/D_2O 9:1), **17**$^{16U}_{3'Ala}$ (partial assignment): δ_H 6.01 (1H, d, *J* = 6.5 Hz, (C1')-H), 5.94 (1H, d, *J* = 8.1 Hz, (C5)-H), 5.38 (1H, dd, *J* = 5.7, 3.5 Hz, (C3')-H).

^{1}H NMR (600 MHz, H_2O/D_2O 9:1), **17**$^{16U}_{2'Ala}$ (partial assignment): δ_H 6.05 (1H, d, *J* = 3.4 Hz, (C1')-H), 5.50 (1H, dd, *J* = 6.0, 3.4 Hz, (C2')-H).

^{1}H NMR (600 MHz, H_2O/D_2O 9:1)), $\mathbf{17^{16U}_{5'Ala}}$ (partial assignment): δ_H 5.82 (1H, d, *J* = 3.8 Hz, (C1')-H).

^{1}H NMR (600 MHz, H_2O/D_2O 9:1, $17^{\mathbf{16U}}_{\mathbf{2',3'Ala}}$ (partial assignment): δ_H 6.16 (1H, d, *J* = 4.7 Hz, (C1')-H), 5.74 (1H, apt. t, *J* = 5.1 Hz, (C2')-H), 5.66 (1H, t, *J* = 5.1, (C3')-H).

Synthesis of aminoacyl-uridine ***17***$^{\boldsymbol{U}}_{\boldsymbol{Gly}}$ *upon incubating uridine (**16U**) with thioester* $\boldsymbol{1_{Gly}}$

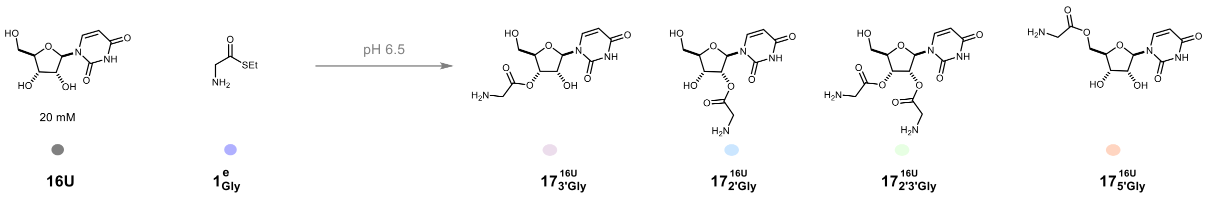

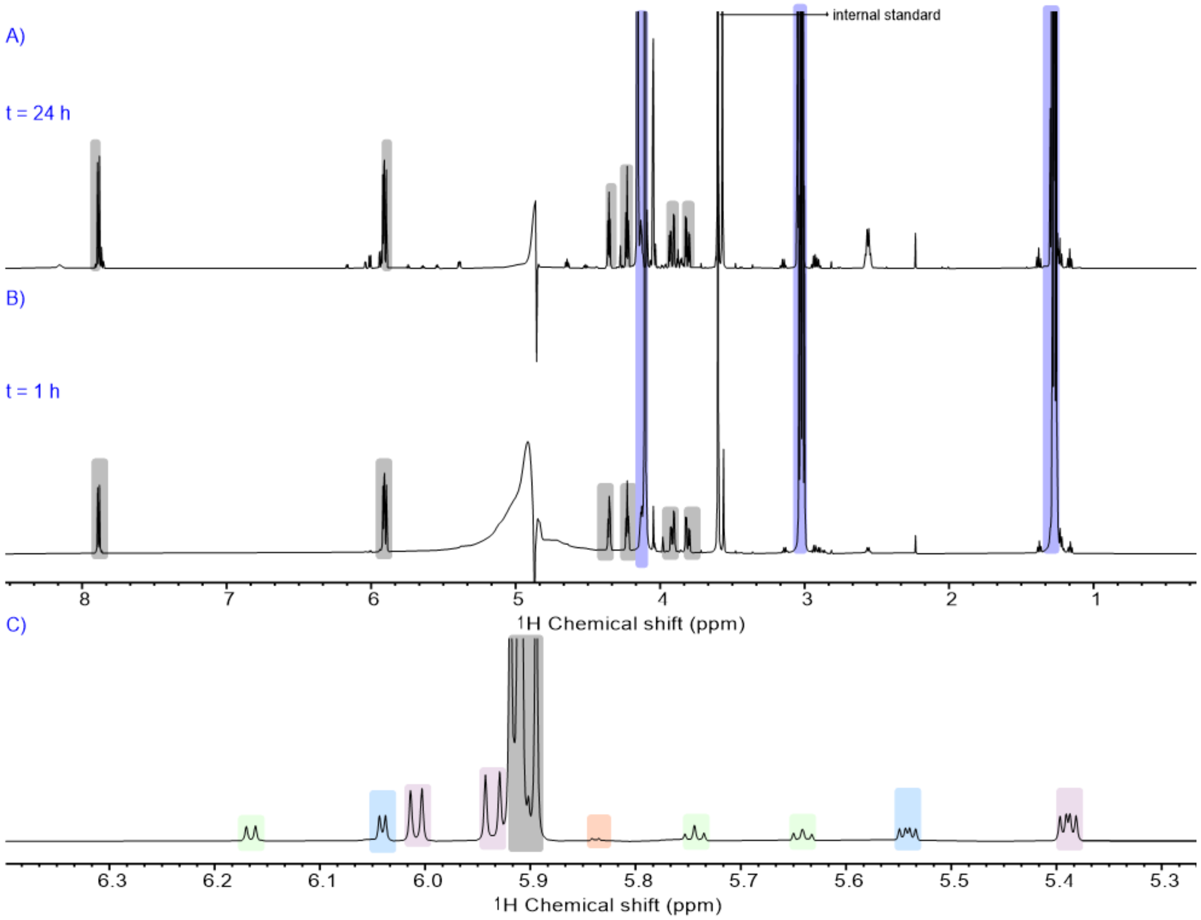

Supplementary Figure 78. ^{1}H NMR (600 MHz, H_2O/D_2O 9:1, noesygppr1d, 1.0 – 8.0 ppm) spectra to show the reaction of thioester $\mathbf{1^{e}_{Gly}}$ *(220 mM) with uridine (**16U**, 20 mM) at pH 6.5, using PET (10 mM) as an internal standard. Set up following General Procedure C, after A) 24 h; B) 1h. C) 5.3 – 6.4 ppm region of spectrum A.*

^{1}H NMR (600 MHz, H_2O/D_2O 9:1), $\mathbf{17^{16U}_{3'Gly}}$ (partial assignment): δ_H 6.01 (1H, d, *J* = 6.5 Hz, (C1')-*H*), 5.94 (1H, d, *J* = 8.1 Hz, (C5)-H), 5.39 (1H, dd, *J* = 5.7, 3.5 Hz, (C3')-H).

^{1}H NMR (600 MHz, H_2O/D_2O 9:1), $\mathbf{17^{16U}_{2'Gly}}$ (partial assignment): δ_H 6.04 (1H, d, *J* = 3.4 Hz, (C1')-H), 5.54 (1H, dd, *J* = 6.0, 3.4 Hz, (C2')-H).

^{1}H NMR (600 MHz, H_2O/D_2O 9:1), $\mathbf{17^{16U}_{5'Gly}}$ (partial assignment): δ_H 5.84 (1H, d, *J* = 3.9 Hz, (C1')-H).

^{1}H NMR (600 MHz, H_2O/D_2O 9:1), $\mathbf{17^{16U}_{2',3'Gly}}$ (partial assignment): δ_H 6.17 (1H, d, *J* = 5.2 Hz, (C1')-H), 5.74 (1H, t, *J* = 5.2 Hz, (C2')-H), 5.64 (1H, t, *J* = 5.2, C3')-H).

Synthesis of aminoacyl-uridine ***17***$^{U}_{Leu}$ *upon incubating uridine (**16U**) with thioester* ***1***$_{Leu}$

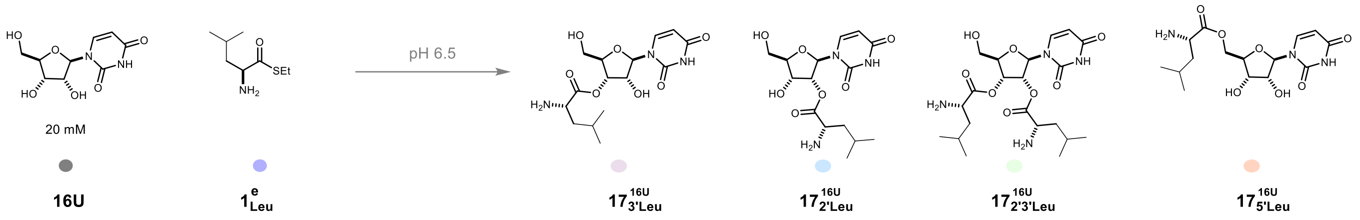

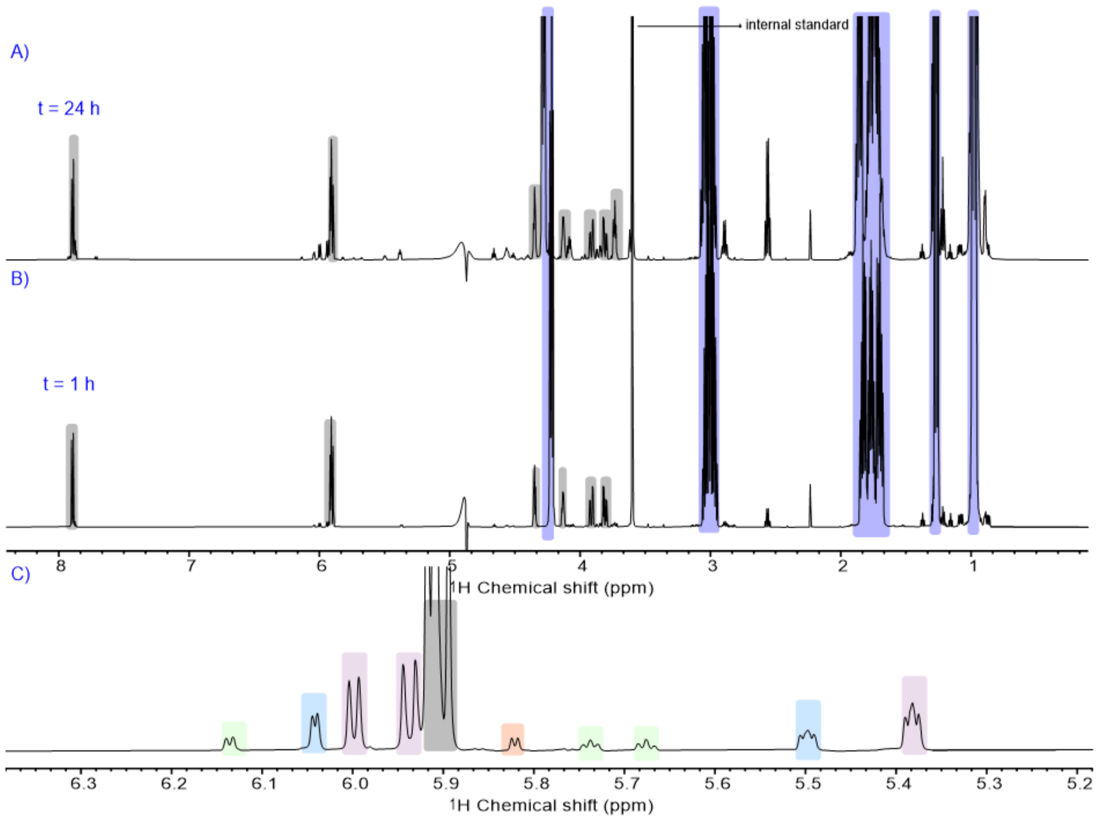

Supplementary Figure 79. ^{1}*H NMR (600 MHz,* H_2O/D_2O *9:1, noesygppr1d, 1.0 – 8.0 ppm) spectra to show the reaction of thioester* 1^{e}_{Leu} *(210 mM) with uridine (**16U**, 20 mM) at pH 6.5, using PET (10 mM) as an internal standard. Set up following General Procedure C, after A) 24 h; B) 1 h. C) 5.2 – 6.3 ppm region of spectrum A.*

^{1}H NMR (600 MHz, H_2O/D_2O 9:1), $\mathbf{17^{16U}_{3'Leu}}$ (partial assignment): δ_H 6.00 (1H, d, *J* = 6.5 Hz, (C1')-*H*), 5.94 (1H, d, *J* = 8.1 Hz, (C5)-H), 5.38 (1H, dd, *J* = 5.7, 3.5 Hz, (C3')-H).

^{1}H NMR (600 MHz, H_2O/D_2O 9:1), $\mathbf{17^{16U}_{2'Leu}}$ (partial assignment): δ_H 6.04 (1H, d, *J* = 3.4 Hz, (C1')-H), 5.50 (1H, dd, *J* = 6.0, 3.4 Hz, (C2')-H).

^{1}H NMR (600 MHz, H_2O/D_2O 9:1), $\mathbf{17^{16U}_{5'Leu}}$ (partial assignment): δ_H 5.82 (1H, d, *J* = 4.0 Hz, (C1')-H).

^{1}H NMR (600 MHz, H_2O/D_2O 9:1), $\mathbf{17^{16U}_{2',3'Gly}}$ (partial assignment): δ_H 6.14 (1H, d, J = 5.2 Hz, (C1')-H), 5.74 (1H, t, J = 5.2 Hz, (C2')-H), 5.68 (1H, t, J = 5.2, (C3')-H).

Synthesis of aminoacyl-uridine $\mathbf{17^{U}_{Pro}}$ *upon incubating uridine (**16U**) with thioester* $\mathbf{1_{Pro}}$

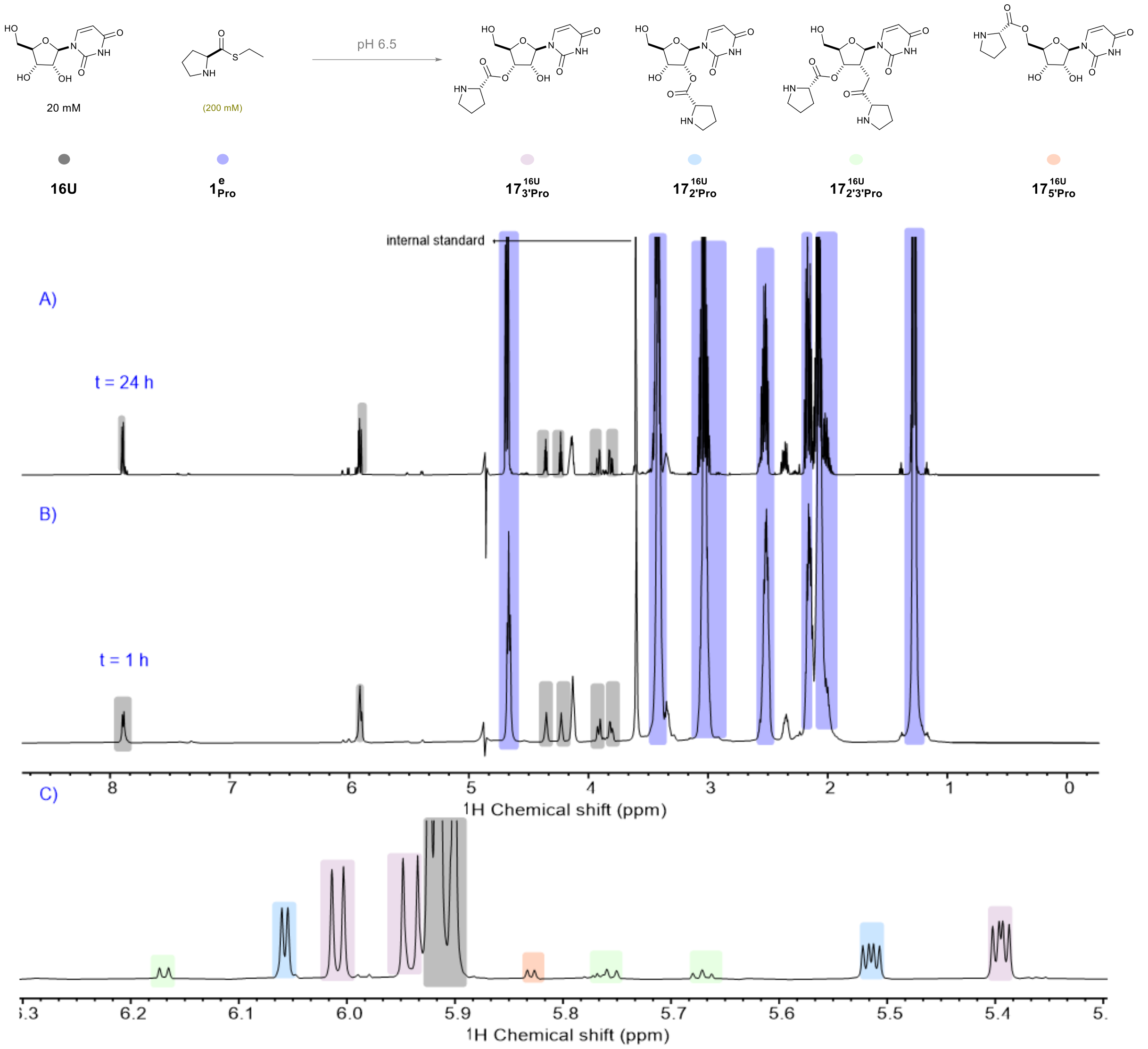

Supplementary Figure 80. ^{1}*H NMR (600 MHz,* H_2O/D_2O *9:1, noesygppr1d, 0.0 – 9.0 ppm) spectra to show the reaction of thioester* $\mathbf{1^{e}_{Pro}}$ *(200 mM) with uridine (**16U**, 20 mM) at pH 6.5, using PET (10 mM) as an internal standard. Set up following General Procedure C, after A) 24 h; B) 1h. C) 5.3 – 6.3 ppm region of spectrum A.*

^{1}H NMR (600 MHz, H_2O/D_2O 9:1), $\mathbf{17^{16U}_{3'Pro}}$ (partial assignment): δ_H 6.00 (1H, d, J = 6.5 Hz, (C1')-H), 5.94 (1H, d, J = 8.1 Hz, (C5)-H), 5.39 (1H, dd, J = 5.7, 3.5 Hz, (C3')-H).

^{1}H NMR (600 MHz, H_2O/D_2O 9:1), $\mathbf{17^{16U}_{2'Pro}}$ (partial assignment): δ_H 6.05 (1H, d, J = 3.4 Hz, (C1')-H), 5.51 (1H, dd, J = 6.0, 3.4 Hz, (C2')-H).

^{1}H NMR (600 MHz, H_2O/D_2O 9:1), $\mathbf{17^{16U}_{5'Pro}}$ (partial assignment): δ_H 5.83 (1H, d, J = 3.9 Hz, (C1')-*H*).

^{1}H NMR (600 MHz, H_2O/D_2O 9:1), $\mathbf{17^{16U}_{3'Pro}}$ (partial assignment): δ_H 6.16 (1H, d, J = 5.0 Hz, (C1')-H), 5.74 (1H, overlapped, (C2')-H), 5.67 (1H, t, J = 5.2 Hz, (C3')-H).

Synthesis of aminoacyl-uridine $\mathbf{17^{U}_{Glu}}$ *upon incubating uridine (**16U**) with thioester* $\mathbf{1_{Glu}}$

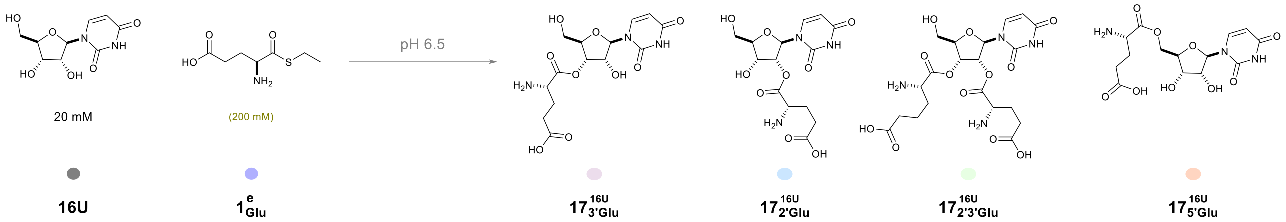

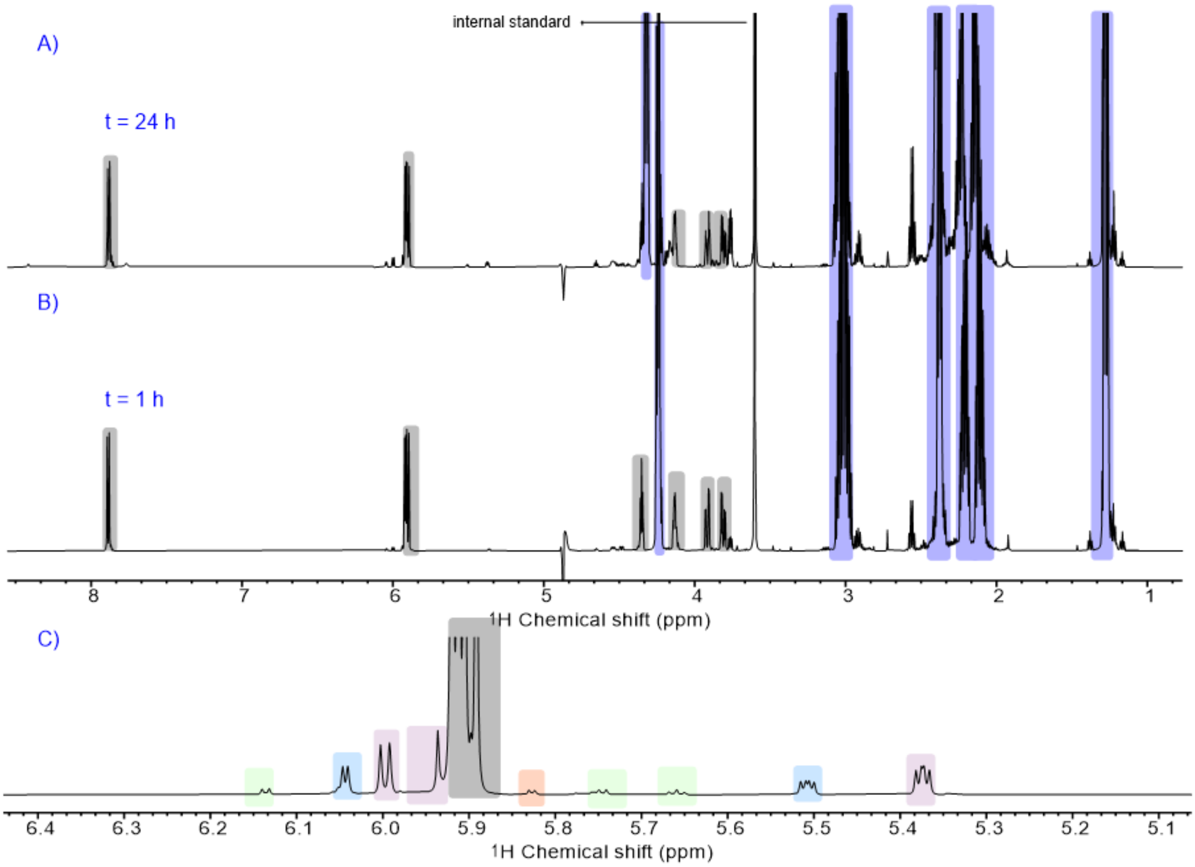

Supplementary Figure 81. [1]*H NMR (600 MHz, H_2O/D_2O 9:1, noesygppr1d, 1.0 – 9.0 ppm) spectra to show the reaction of thioester* $\mathbf{1^{e}_{Glu}}$ *(190 mM) with uridine (**16U**, 20 mM) at pH 6.5, using PET (10 mM) as an internal standard. Set up following General Procedure C, after A) 24 h; B) 1h. C) 5.1 – 6.4 ppm region of spectrum A.*

^{1}H NMR (600 MHz, H_2O/D_2O 9:1), $\mathbf{17^{16U}_{3'Glu}}$ (partial assignment): δ_H 6.00 (1H, d, J = 6.5 Hz, (C1')-H), 5.94 (1H, overlapped, (C5)-H), 5.37 (1H, dd, J = 5.7, 3.7 Hz, (C3')-H).

^{1}H NMR (600 MHz, H_2O/D_2O 9:1), $\mathbf{17^{16U}_{2'Glu}}$ (partial assignment): δ_H 6.04 (1H, d, J = 3.4 Hz, (C1')-H), 5.51 (1H, dd, J = 6.0, 3.4 Hz, (C2')-H).

^{1}H NMR (600 MHz, H_2O/D_2O 9:1), $\mathbf{17^{16U}_{5'Glu}}$ (partial assignment): δ_H 5.83 (1H, d, *J* = 3.9 Hz, (C1')-H).

^{1}H NMR (600 MHz, H_2O/D_2O 9:1), $\mathbf{17^{16U}_{2',3'Leu}}$ (partial assignment): δ_H 6.13 (1H, d, *J* = 4.8 Hz, (C1')-H), 5.77 (1H, overlapped, (C2')-H), 5.66 (1H, t, *J* = 5.2, (C3')-H).

*Synthesis of aminoacyl-uridine $\mathbf{17^{U}_{Gln}}$ upon incubating uridine (**16U**) with thioester $\mathbf{1_{Gln}}$*

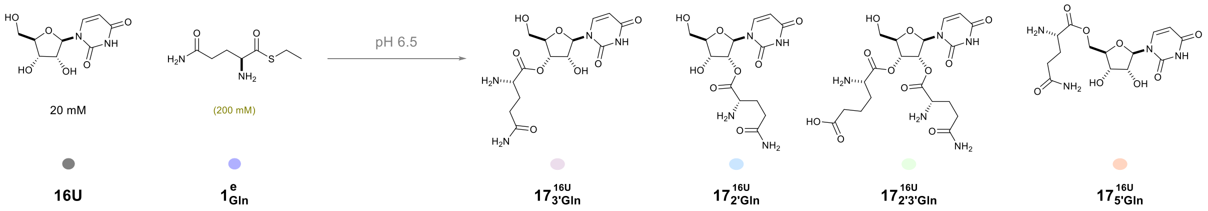

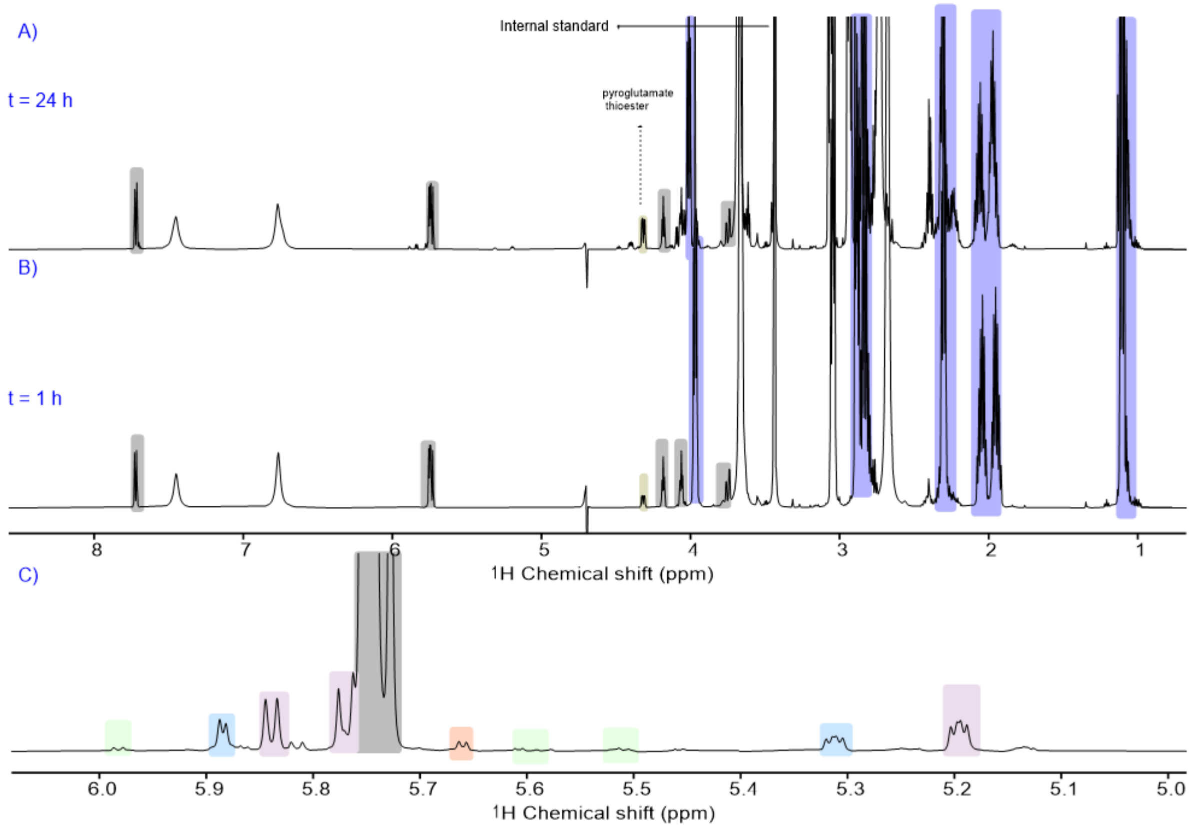

*Supplementary Figure 82. ^{1}H NMR (600 MHz, H_2O/D_2O 9:1, noesygppr1d, 1.0 – 9.0 ppm) spectra to show the reaction of thioester $\mathbf{1^{e}_{Gln}}$ (185 mM) with uridine (**16U**, 20 mM) in MES buffer (200 mM, pH 6.5), using PET (10 mM) as an internal standard. Set up following General Procedure C, after A) 24 h; B) 1h. C) 5.0 – 6.0 ppm region of spectrum A.*

^{1}H NMR (600 MHz, H_2O/D_2O 9:1), $\mathbf{17^{16U}_{3'Gln}}$ (partial assignment): δ_H 6.00 (1H, d, *J* = 6.5 Hz, (C1')-H), 5.93 (1H, d, *J* = 8.1 Hz, (C5)-H), 5.36 (1H, dd, *J* = 5.7, 3.7 Hz, (C3')-H).

^{1}H NMR (600 MHz, H_2O/D_2O 9:1), $\mathbf{17^{16U}_{2'Gln}}$ (partial assignment): δ_H 6.05 (1H, d, J = 3.4 Hz, (C1')-H), 5.47 (1H, dd, J = 6.0, 3.4 Hz, (C2')-H).

^{1}H NMR (600 MHz, H_2O/D_2O 9:1, $\mathbf{17^{16U}_{5'Gln}}$ (partial assignment): δ_H 5.83 (1H, d, J = 4.3 Hz, (C1')-*H*).

^{1}H NMR (600 MHz, H_2O/D_2O 9:1, $\mathbf{1^{e}_{PyroGln}}$ (partial assignment): δ_H 4.48 (1H, dd, J = 9.5, 3.7 Hz, pyro-Gln-α-C*H*).

Synthesis of aminoacyl-uridine $\mathbf{17^{U}_{Met}}$ *upon incubating uridine (**16U**) with thioester* $\mathbf{1_{Met}}$

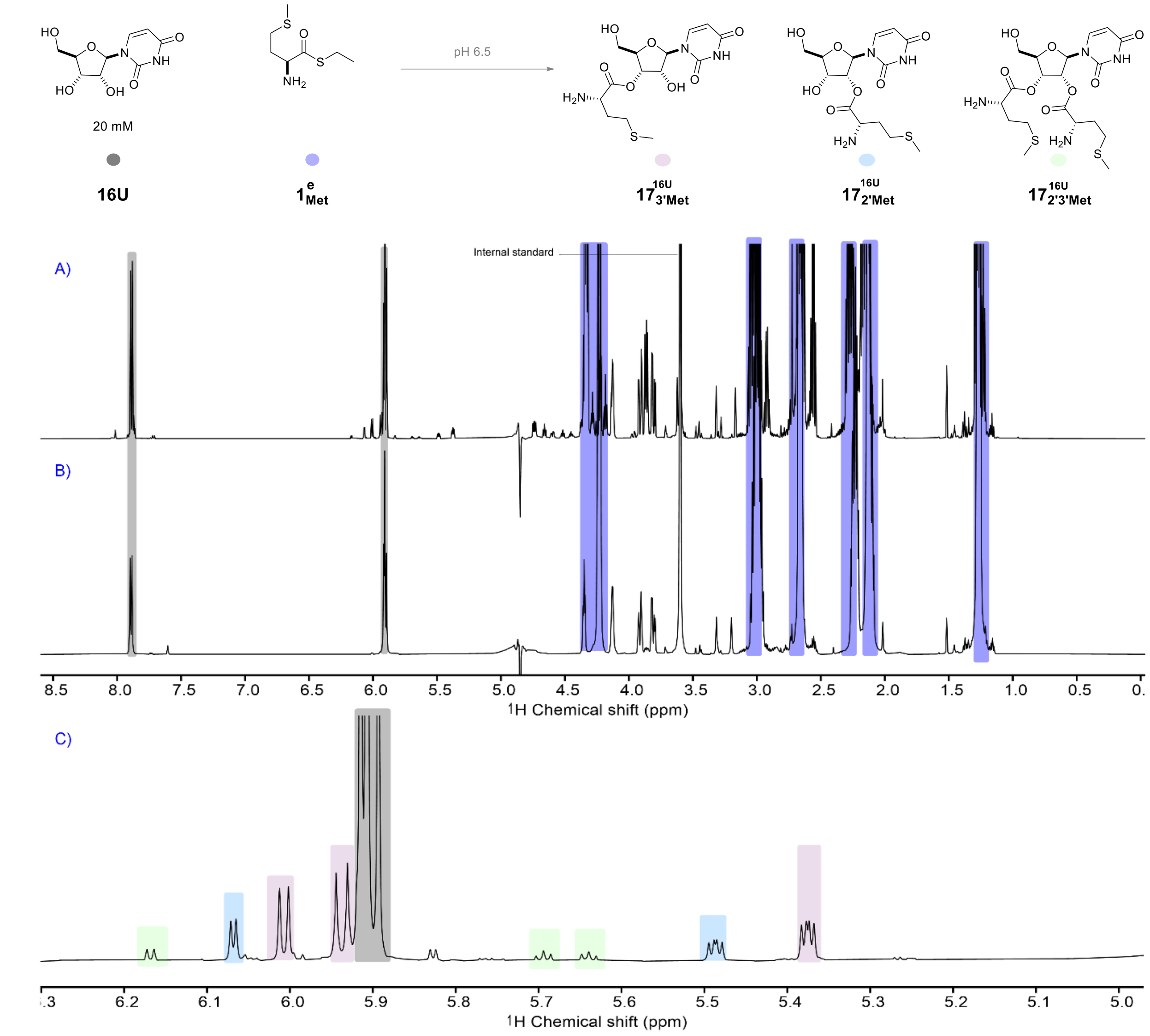

Supplementary Figure 83: ^{1}H NMR (600 MHz, H_2O/D_2O 9:1, noesygppr1d, 0.5 – 8.5 ppm) spectra to show the reaction of methionine thioester $\mathbf{1^{e}_{Met}}$ *(210 mM) with uridine (**16U**, 20 mM) at pH 6.5, using PET (10 mM) as an internal standard. Set up following General Procedure C, after A) 24 h; B) 1 h. C) 5.0 – 6.3 ppm region of spectrum A.*

^{1}H NMR (600 MHz, H_2O/D_2O 9:1), $\mathbf{17^{16U}_{3'Met}}$ (partial assignment): δ_H 6.01 (1H, d, J = 6.5 Hz, (C1')-H), 5.94 (1H, d, J = 8.1 Hz, (C5)-H), 5.38 (1H, dd, J = 5.6, 3.8 Hz, (C3')-H).

^{1}H NMR (600 MHz, H_2O/D_2O 9:1), $\mathbf{17^{16U}_{2'Met}}$ (partial assignment): δ_H 6.07 (1H, d, *J* = 3.4 Hz, (C1')-H), 5.49 (1H, dd, *J* = 5.8, 3.4 Hz, (C2')-H).

^{1}H NMR (600 MHz, H_2O/D_2O 9:1), $\mathbf{17^{16U}_{2',3'Met}}$ (partial assignment): δ_H 6.17 (1H, d, *J* = 5.1 Hz, (C1')-H), 5.70 (1H, apt. t, *J* = 5.2 Hz, (C2')-H), 5.64 (1H, t, *J* = 5.2 Hz, (C3')-H).

*Synthesis of aminoacyl-uridine $\mathbf{17^{U}_{Arg}}$ upon incubating uridine (**16U**) with thioester $\mathbf{1_{Arg}}$*

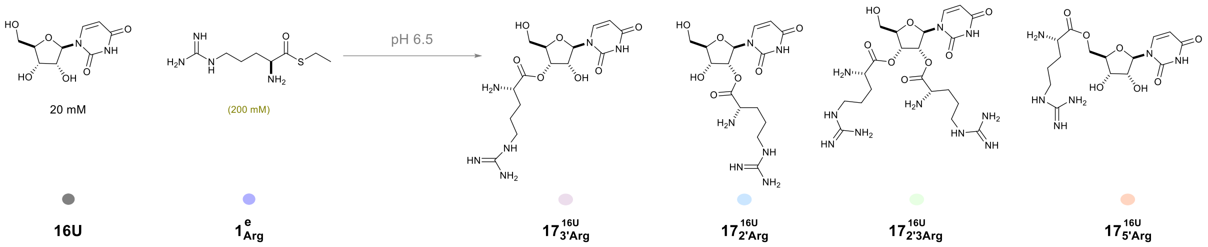

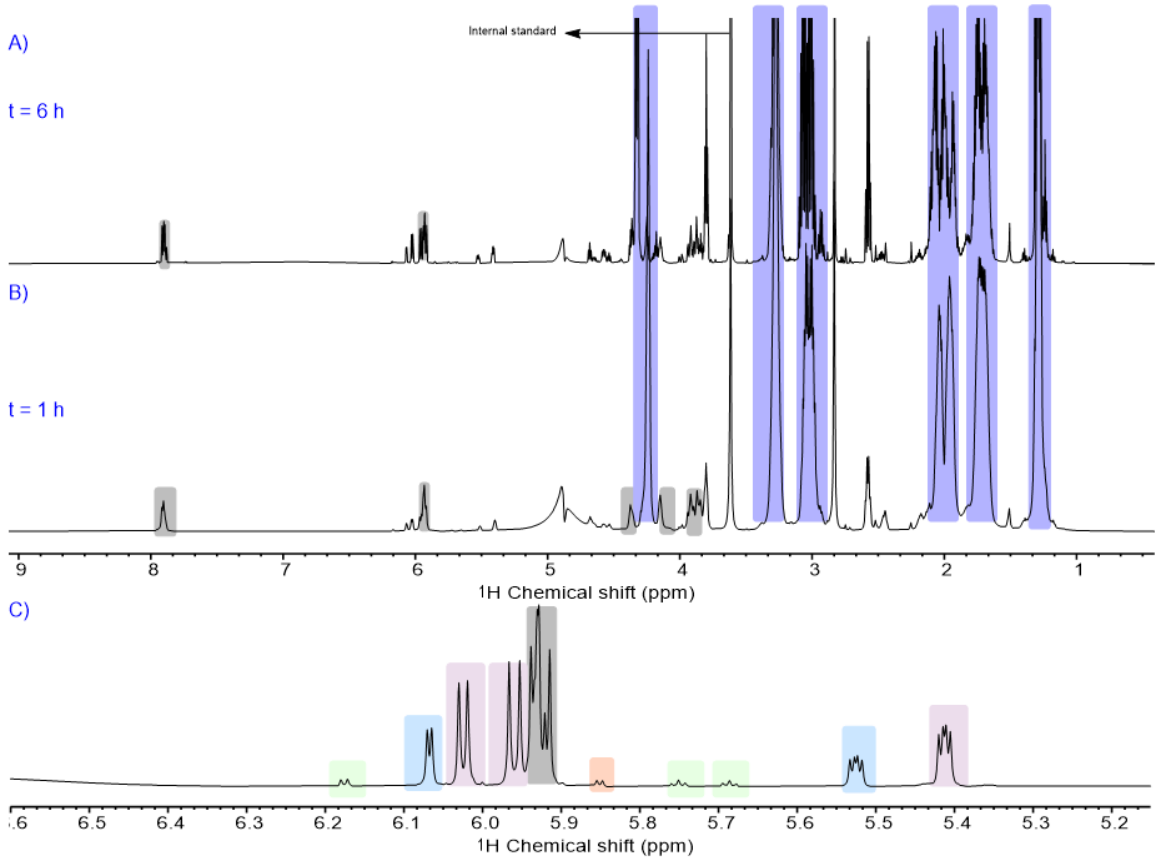

*Supplementary Figure 84. ¹H NMR (600 MHz, H₂O/D₂O 9:1, noesygppr1d, 1.0 – 9.0 ppm) spectra to show the reaction of thioester $\mathbf{1^{e}_{Arg}}$ (220 mM) with uridine (**16U**, 20 mM) at pH 6.5, using PET (10 mM) as an internal standard. Set up following General Procedure C, after A) 24 h; B) 1 h. C) 5.2 – 6.6 ppm region of spectrum A.*

^{1}H NMR (600 MHz, H_2O/D_2O 9:1), $\mathbf{17^{16U}_{3'Arg}}$ (partial assignment): δ_H 6.01 (1H, d, *J* = 6.5 Hz, (C1')-H), 5.95 (1H, d, *J* = 8.1 Hz, (C5)-H), 5.40 (1H, dd, *J* = 5.7, 3.7 Hz, (C3')-H).

^{1}H NMR (600 MHz, H_2O/D_2O 9:1), $\mathbf{17^{16U}_{2'Arg}}$ (partial assignment): δ_H 6.05 (1H, d, *J* = 3.4 Hz, (C1')-H), 5.51 (1H, dd, *J* = 6.0, 3.4 Hz, (C2')-H).

^{1}H NMR (600 MHz, H_2O/D_2O 9:1), $\mathbf{17^{16U}_{5'Arg}}$ (partial assignment): δ_H 5.84 (1H, d, *J* = 4.3 Hz, (C1')-H).

^{1}H NMR (600 MHz, H_2O/D_2O 9:1), $\mathbf{17}^{\mathbf{16U}}_{\mathbf{2',3'Arg}}$ (partial assignment): δ_H 6.16 (1H, d, *J* = 4.8 Hz, (C1')-H), 5.74 (1H, dd, *J* = 4.8, 5.2 Hz, (C2')-H), 5.67 (1H, t, *J* = 5.2 Hz, (C3')-H).

Synthesis of aminoacyl-uridine $\mathbf{17^{U}_{Lys}}$ *upon incubating uridine (**16U**) with thioester* $\mathbf{1_{Lys}}$

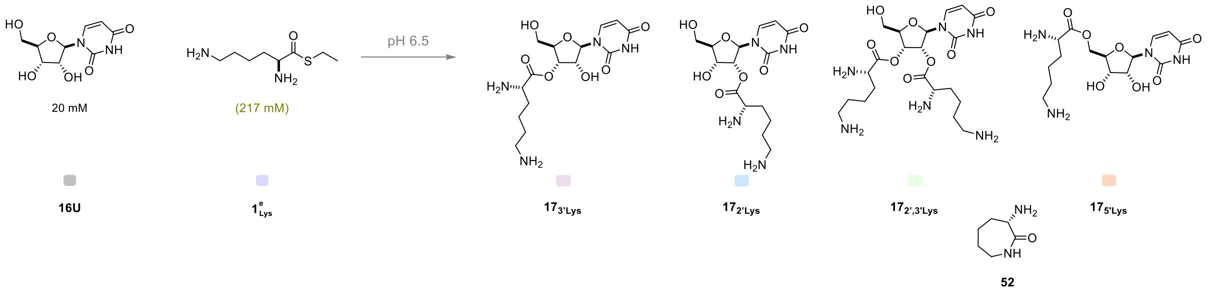

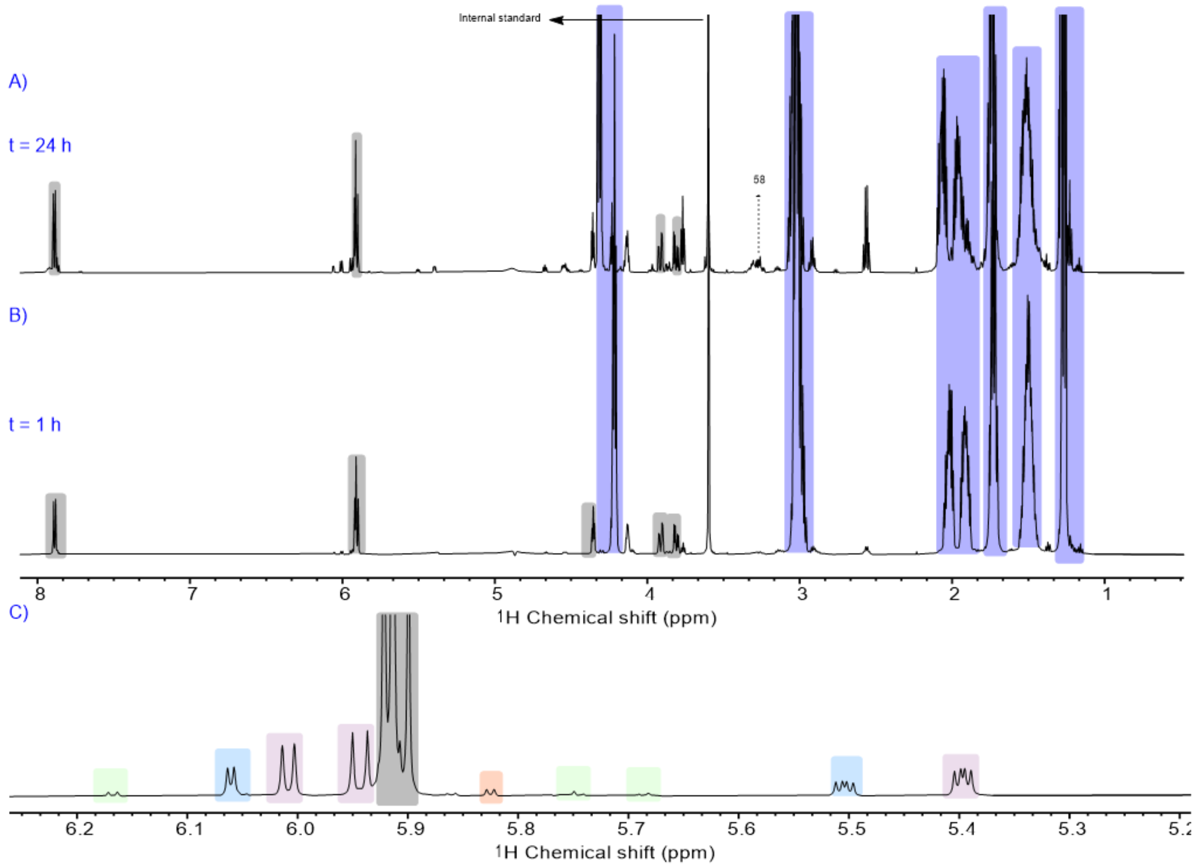

Supplementary Figure 85. ^{1}*H NMR (600 MHz,* H_2O/D_2O *9:1, noesygppr1d, 1.0 – 8.0 ppm) spectra to show the reaction of thioester* $\mathbf{1^{e}_{Lys}}$ *(220 mM) with uridine (**16U**, 20 mM) at pH 6.5, using PET (10 mM) as an internal standard. Set up following General Procedure C, after A) 24 h; B) 1 h. C) 5.2 – 6.3 ppm region of spectrum A.*

^{1}H NMR (600 MHz, H_2O/D_2O 9:1), $\mathbf{17^{16U}_{3'Lys}}$ (partial assignment): δ_H 6.01 (1H, d, *J* = 6.5 Hz, (C1')-H), 5.94 (1H, d, *J* = 8.1 Hz, (C5)-H), 5.40 (1H, dd, *J* = 5.7, 3.7 Hz, (C3')-H).

^{1}H NMR (600 MHz, H_2O/D_2O 9:1), $\mathbf{17^{16U}_{2'Lys}}$ (partial assignment): δ_H 6.06 (1H, d, *J* = 3.4 Hz, (C1')-H), 5.50 (1H, dd, *J* = 6.0, 3.4 Hz, (C2')-H).

^{1}H NMR (600 MHz, H_2O/D_2O 9:1), $\mathbf{17^{16U}_{5'Lys}}$ (partial assignment): δ_H 5.83 (1H, d, *J* = 4.3 Hz, (C1')-H).

^{1}H NMR (600 MHz, H_2O/D_2O 9:1), $\mathbf{17^{16U}_{2',3'Lys}}$ (partial assignment): δ_H 6.17 (1H, d, *J* = 4.8 Hz, (C1')-H), 5.75 (1H, apt. t, *J* = 5.2 Hz, (C2')-H), 5.68 (1H, t, *J* = 5.2 Hz, (C3')-H).

*Synthesis of aminoacyl-uridine $\mathbf{17^{U}_{His}}$ upon incubating uridine (**16U**) with thioester $\mathbf{1_{His}}$*

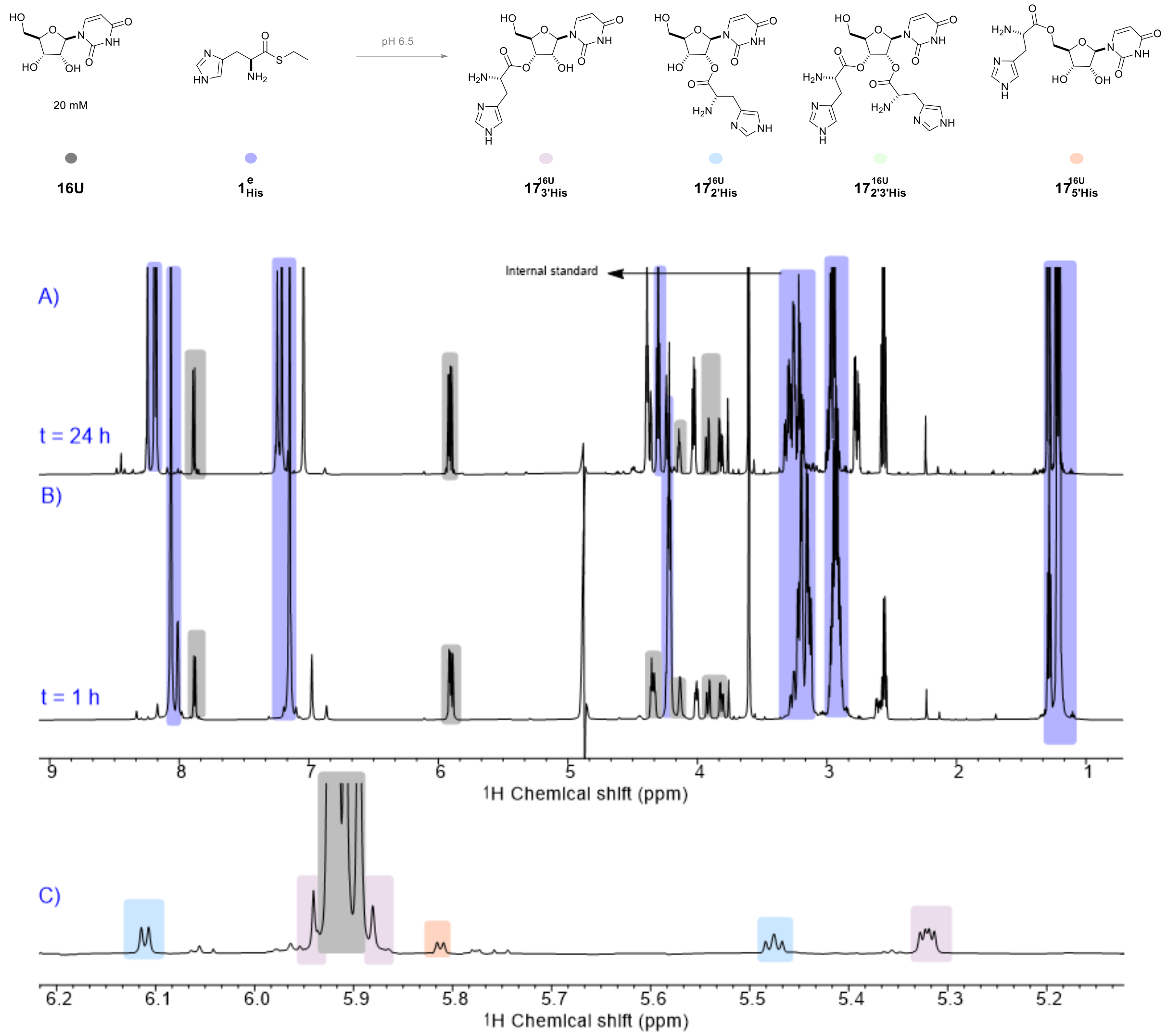

*Supplementary Figure 86. ^{1}H NMR (600 MHz, H$_2$O/D$_2$O 9:1, noesygppr1d, 1.0 – 9.0 ppm) spectra to show the reaction of thioester $\mathbf{1^{e}_{His}}$ (180 mM) with uridine (**16U**, 20 mM) at pH 6.5, using PET (10 mM) as an internal standard. Set up following General Procedure C, after A) 24 h; B) 1 h. C) 5.2 – 6.3 ppm region of spectrum A.*

^{1}H NMR (600 MHz, H$_2$O/D$_2$O 9:1), $\mathbf{17^{16U}_{3'His}}$ (partial assignment): δ$_H$ 5.94 (1H, overlapped, (C1')-H), 5.89 (1H, overlapped, (C5)-H), 5.32 (1H, dd, *J* = 5.7, 3.7 Hz, (C3')-H).

^{1}H NMR (600 MHz, H$_2$O/D$_2$O 9:1), $\mathbf{17^{16U}_{2'His}}$ (partial assignment): δ$_H$ 6.1 (1H, d, *J* = 4.4 Hz, (C1')-H), 5.48 (1H, dd, *J* = 6.0, 4.4 Hz, (C2')-H).

^{1}H NMR (600 MHz, H$_2$O/D$_2$O 9:1), $\mathbf{17^{16U}_{5'His}}$ (partial assignment): δ$_H$ 5.81 (1H, d, *J* = 3.9 Hz, (C1')-H).

*Synthesis of aminoacyl-uridine **17^{U}_{Val}** upon incubating uridine (**16U**) with thioester **1_{Val}***

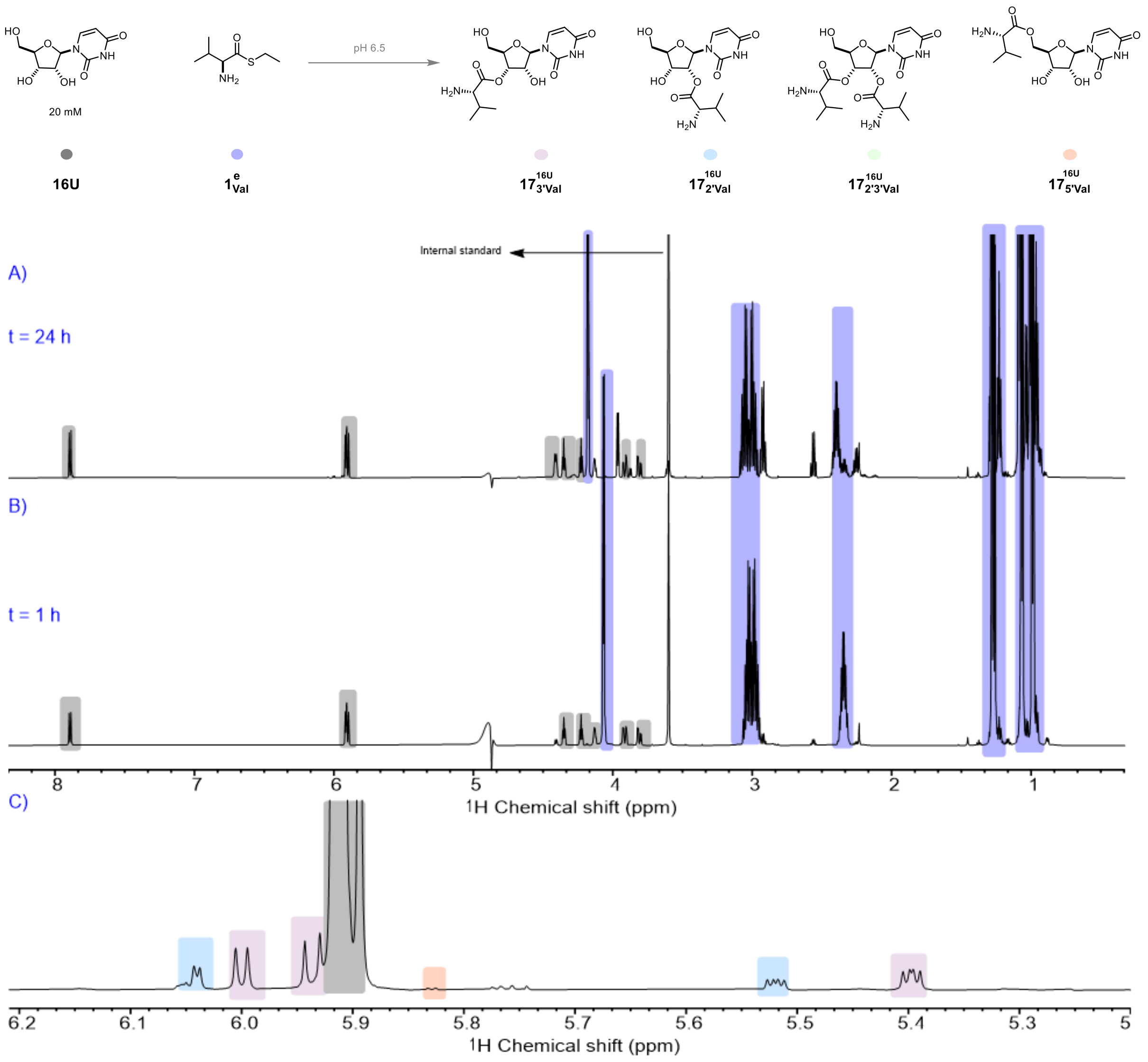

*Supplementary Figure 87. ¹H NMR (600 MHz, H_2O/D_2O 9:1, noesygppr1d, 0.5 – 8.0 ppm) spectra to show the reaction of thioester **1^{e}_{Val}** (220 mM) with uridine (**16U**, 20 mM) at pH 6.5, using PET (10 mM) as an internal standard. Set up following General Procedure C, after A) 24 h; B) 1 h. C) 5.2 – 6.2 ppm region of spectrum A.*

¹H NMR (600 MHz, H_2O/D_2O 9:1), **$17^{16U}_{3'Val}$** (partial assignment): δ_H 6.00 (1H, d, *J* = 6.5 Hz, (C1')-H), 5.94 (1H, d, *J* = 8.1 Hz, (C5)-H), 5.40 (1H, dd, *J* = 5.7, 3.7 Hz, (C3')-H).

¹H NMR (600 MHz, H_2O/D_2O 9:1), **$17^{16U}_{2'Val}$** (partial assignment): δ_H 6.04 (1H, d, *J* = 3.4 Hz, (C1')-H), 5.52 (1H, dd, *J* = 5.8, 3.4 Hz, (C2')-H).

Synthesis of aminoacyl-uridine ***17***$^{U}_{Phe}$ *upon incubating uridine (**16U**) with thioester* ***1***$_{Phe}$

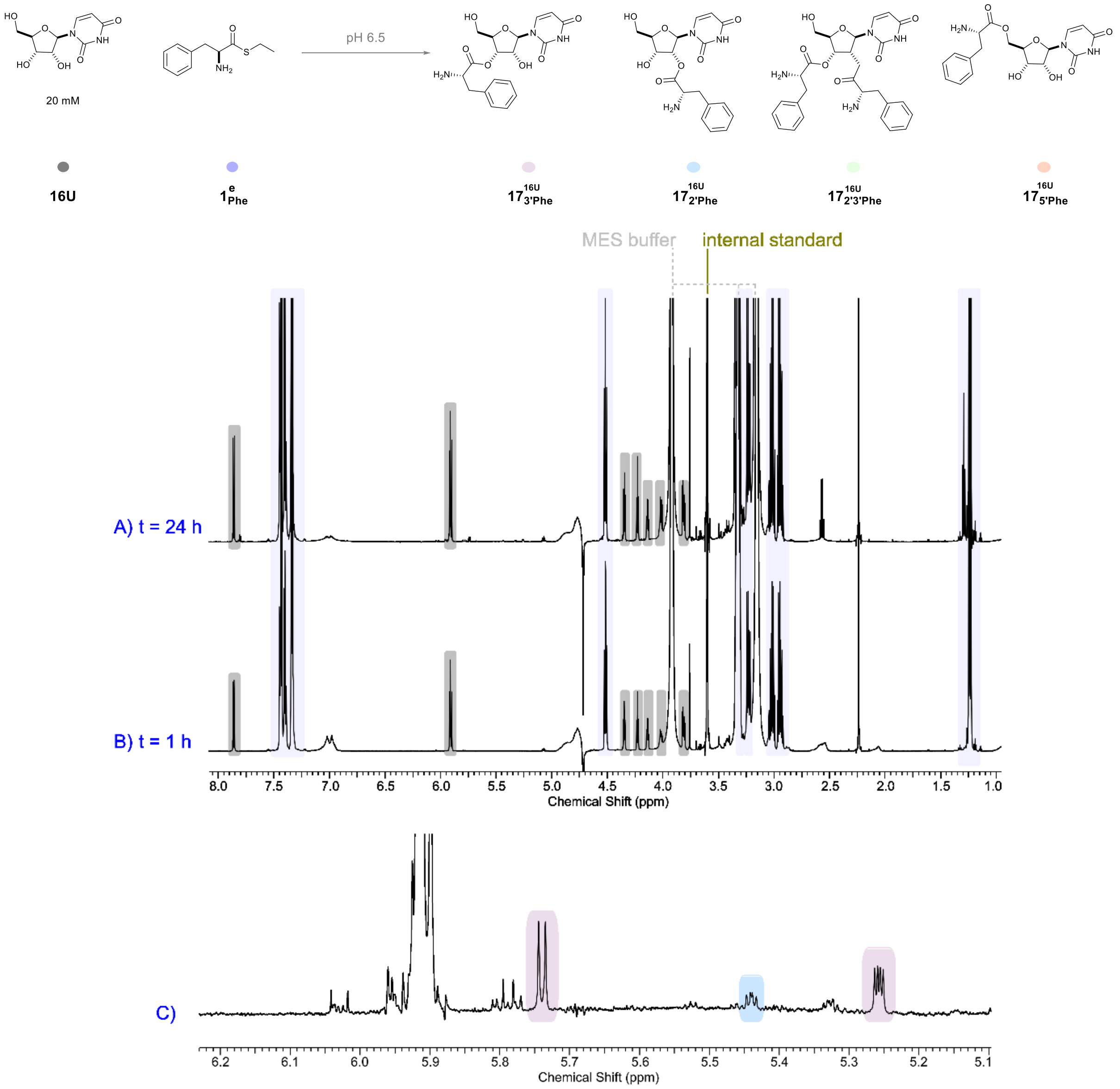

Supplementary Figure 88. ^{1}H NMR (700 MHz, H_2O/D_2O 98:2, noesygppr1d, 1.0 – 8.0 ppm) spectra to show the reaction of thioester $\mathbf{1}^{e}_{Phe}$ *(150 mM) with uridine (**16U**, 20 mM) in MES buffer (400 mM, pH 6.5) using PET (30 mM) as an internal standard. Set up following General Procedure C, after A) 24 h; B) 1 h. C) 5.1 – 6.2 ppm region of spectrum A.*

^{1}H NMR (700 MHz, H_2O/D_2O 98:2), $\mathbf{17^{16U}_{3'Phe}}$ (partial assignment): δ_H 5.74 (1H, d, J = 7.0 Hz, (C1')–H), 5.26 (1H, dd, J = 5.6, 2.9 Hz, (C3')–H).

^{1}H NMR (700 MHz, H_2O/D_2O 98:2), $\mathbf{17^{16U}_{3'Phe}}$ (partial assignment): δ_H 5.44 (1H, dd, J = 5.8, 3.8 Hz, (C2')–H).

*Synthesis of aminoacyl-uridine $\mathbf{17^{U}_{Ser}}$ upon incubating uridine (**16U**) with thioester $\mathbf{1_{Ser}}$*

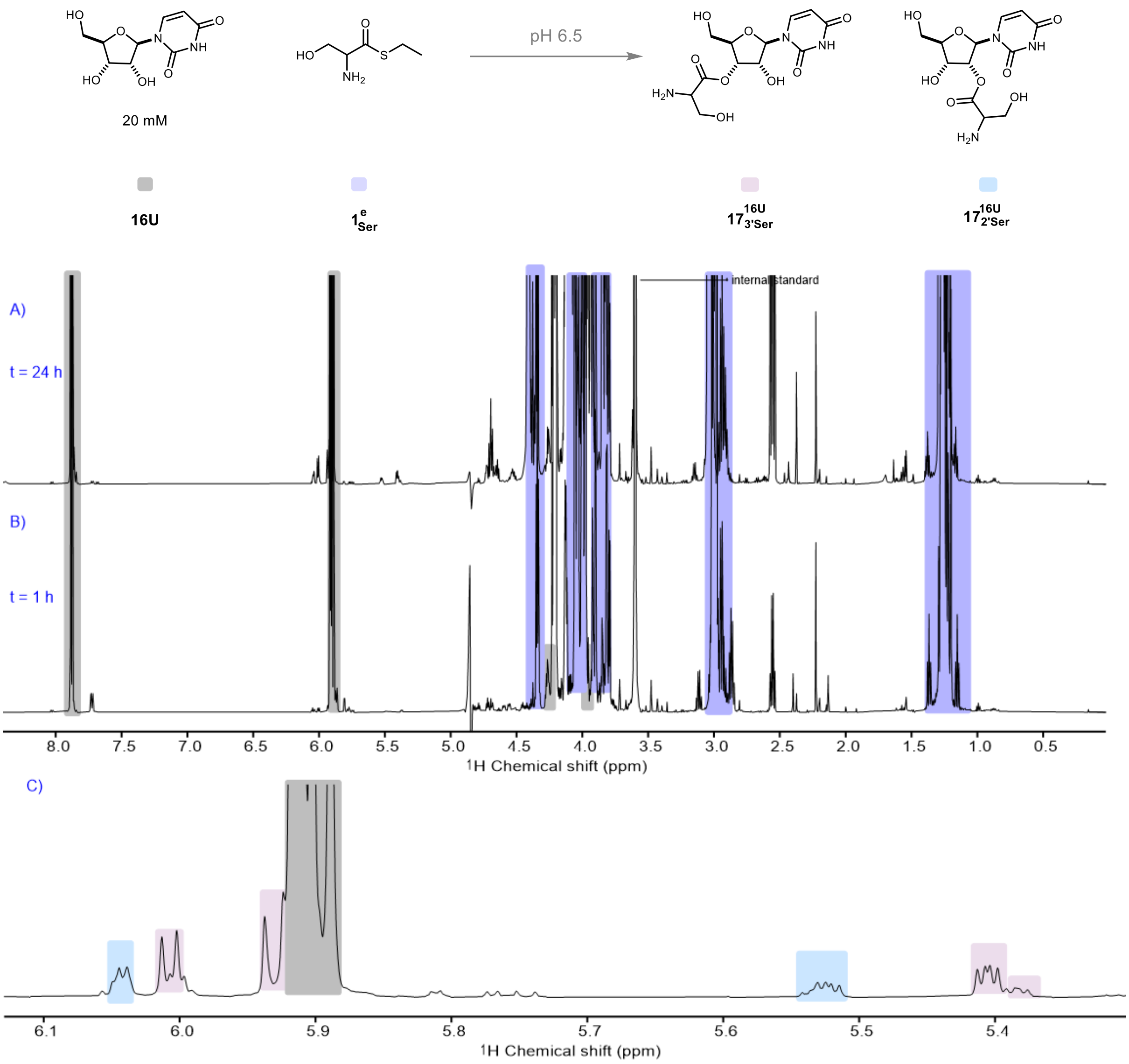

*Supplementary Figure 89. ¹H NMR (600 MHz, H_2O/D_2O 9:1, noesygppr1d, 0.5 – 8.5 ppm) spectra to show the reaction of ethyl aminoacyl thiol L-$\mathbf{1^{e}_{Ser}}$ (230 mM) with uridine (**16U**, 20 mM) at pH 6.5, using PET (10 mM) as an internal standard. Set up following General Procedure C, after A) 24 h; B) 1 h. C) 5.2 – 6.2 ppm region of spectrum A. The ethyl aminoacyl thiol $\mathbf{1^{e}_{Ser}}$ undergoes racemization during thioester synthesis. For additional details, see the N-carboxyanhydride $\mathbf{4_{ser}}$ reaction with thiol in Supplementary Figures **157** and **158**.*

¹H NMR (600 MHz, H_2O/D_2O 9:1), $\mathbf{17^{16U}_{3'Ser}}$ (partial assignment – both diastereomers a + b): δ_H 6.01 (2H, m, (C1')-Ha + (C1')-Hb), 5.94 (2H, overlapped, (C5)-Ha/b), 5.41 (1H, dd, *J* = 5.6, 3.4 Hz, (C3')-Ha), 5.38 (1H, dd, *J* = 5.7, 3.5 Hz, (C3')-Hb).

¹H NMR (600 MHz, H_2O/D_2O 9:1), $\mathbf{17^{16U}_{2'Ser}}$ (partial assignment – both diastereomers a + b): δ_H 6.05 (2H, m, overlapped, (C1')-Ha + (C1')-Hb), 5.54 (2H, m, (C2')-Ha + (C2')-Hb).

*Synthesis of aminoacyl-uridine **17^{U}_{Pip}** upon incubating uridine (**16U**) with thioester **1_{Pip}***

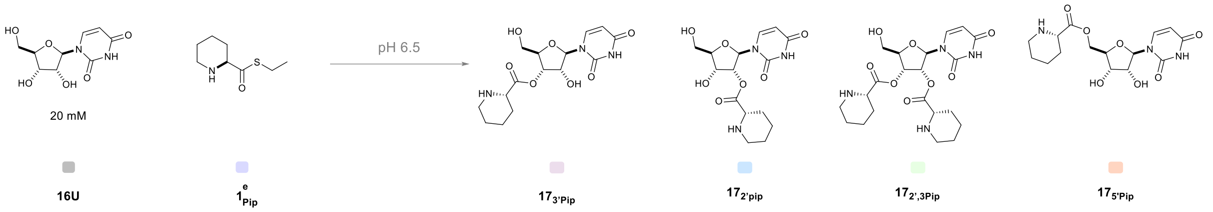

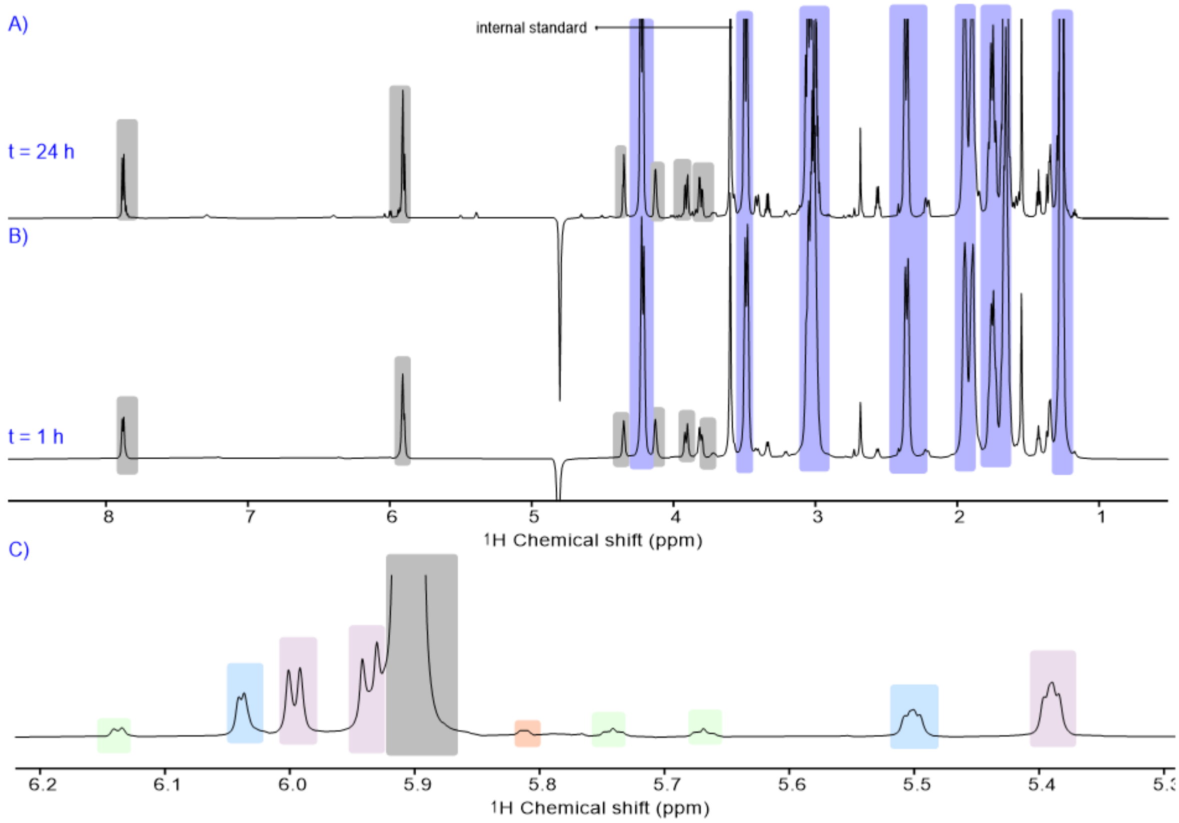

*Supplementary Figure 90. ^{1}H NMR (600 MHz, H_2O/D_2O 9:1, noesygppr1d, 1.0 – 8.0 ppm) spectra to show the reaction of thioester 1^{e}_{Pip} (180 mM) with uridine (**16U**, 20 mM) at pH 6.5, using PET (10 mM) as an internal standard. Set up following General Procedure C, after A) 24 h; B) 1 h. C) 5.3 – 6.2 ppm region of spectrum A.*

^{1}H NMR (600 MHz, H_2O/D_2O 9:1), $\mathbf{17^{16U}_{3'Pip}}$ (partial assignment): δ_H 6.00 (1H, d, *J* = 6.5 Hz, (C1')-H), 5.94 (1H, d, *J* = 8.1 Hz, (C5)-H), 5.39 (1H, dd, *J* = 5.7, 3.7 Hz, (C3')-H).

^{1}H NMR (600 MHz, H_2O/D_2O 9:1), $\mathbf{17^{16U}_{2'Pip}}$ (partial assignment): δ_H 6.04 (1H, d, *J* = 3.4 Hz, (C1')-H), 5.50 (1H, dd, *J* = 5.9, 3.4 Hz, (C2')-H).

^{1}H NMR (600 MHz, H_2O/D_2O 9:1) $\mathbf{17^{16U}_{5'Pip}}$ (partial assignment): δ_H 5.82 (1H, d, *J* = 3.8 Hz, (C1')-H).

^{1}H NMR (600 MHz, H_2O/D_2O 9:1) $\mathbf{7^{16U}_{2',3'Pip}}$ (partial assignment): δ_H 6.14 (1H, d, *J* = 4.7 Hz, (C1')-H), 5.74 (1H, apt. t, *J* = 4.7 Hz, (C2')-H), 5.66 (1H, t, *J* = 5.1, (C3')-H).

Synthesis of aminoacyl-uridine ***17^{U}$_{Ala}$*** *under eutectic conditions upon incubating uridine (****16U****) with thioester* ***1$_{Ala}$***

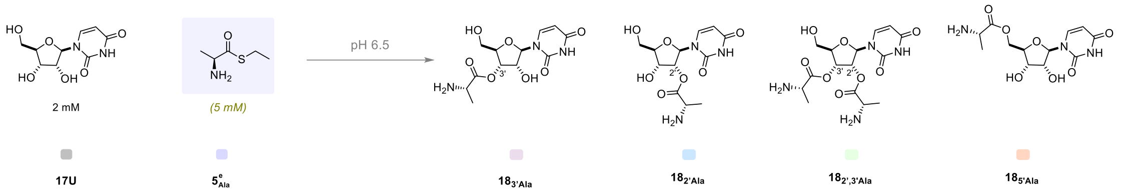

Uridine (**16U**, 2 μmol) and thioester **1$^{e}_{Ala}$** (5–10 μmol) were dissolved in degassed H_2O (0.9 mL). The solution was adjusted to pH 6.5 with 4 M NaOH and the solution volume made up to 1 mL. The sample was flash-frozen at -80 °C and then incubated in a cryogenic bath at -7 °C. After the specified time the reaction was thawed, and NMR spectra were acquired. The observed yields are given in Supplementary Table **12**.

Entry	**L-1$^{e}_{Ala}$** (mM)	Time (days)	Temperature (° C)	**17$_{3'Ala}$** (%)	**17$_{2'Ala}$** (%)	**17$_{2',3'Ala}$** (%)	**17$_{5'Ala}$** (%)	**Total** (%)
1	5	1	-7	14	7	4	2	27
2	5	5	-7	25	13	7	7	53*
3	10	1	-7	20	11	11	4	46
4	10	5	-7	28	14	9	7	58#
5	10	1	r.t.	n.d.	n.d.	n.d.	n.d.	n.d.
6	10	5	r.t.	n.d.	n.d.	n.d.	n.d.	n.d.

*Supplementary Table 12. Total yields of a-aminoacyl ester observed in the reaction of thioester (L-****1$^{e}_{Ala}$****) with uridine (****16U****, 2 mM) at pH 6.5. Reaction at -7 °C were prepared as solutions at room temperature, then flash-frozen at -80 °C and then incubated for the specified time at -7 °C. r.t. = room temperature; n.d = not detected.*

** = In eutectic reaction, <2% racemization was observed after 5 days, and (tentatively assigned) 3',5' and 2',5'-bis amino-acylated products were observed (~8%). These numbers are not included in the table %-yield recorded in the table.*

# = In eutectic reaction after 5 days, ~3% racemization was observed, and (tentatively assigned) 3',5' and 2',5'-bis amino-acylated products were observed (~8%). These numbers are not included in the table %-yield recorded in the table.

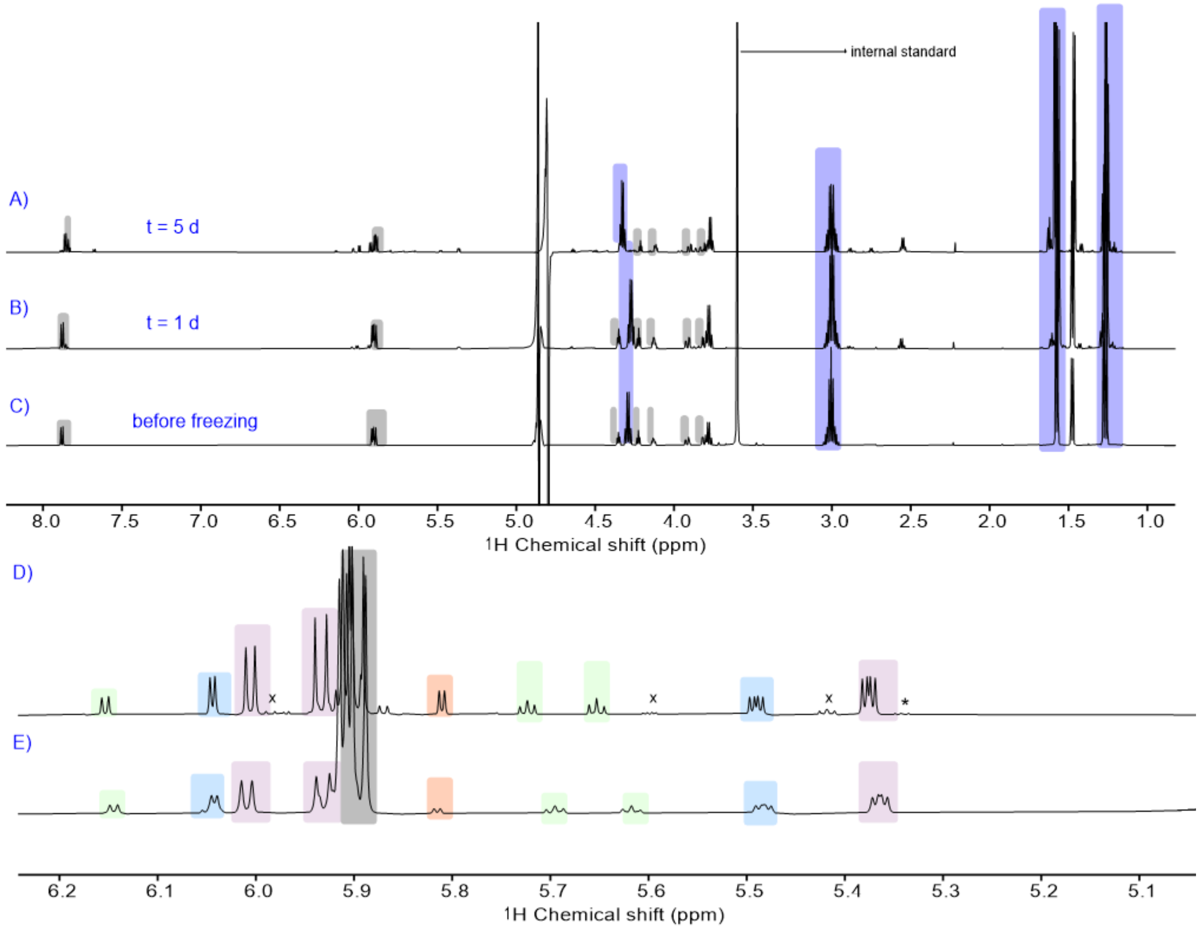

Supplementary Figure 91. *^{1}H NMR (600 MHz, H_2O/D_2O 9:1, noesygppr1d, 1.0 – 8.0 ppm) spectra to show the reaction of thioester* $\mathbf{1^{e}_{Ala}}$ *(5 mM) with uridine (**16U**, 2 mM) at pH 6.5, in eutectic mixture at -7 °C after: A) 5 days; B) 1 day. C) Reaction mixture before forming the eutectic. D) 5.1 – 6.2 ppm region of spectrum A. E) 5.1 – 6.2 ppm region of spectrum B. * = In eutectic reaction after 5 days, <2% racemization was observed. Tentatively assigned X = 3',5' and 2',5' -bis amino-acylated product was observed (~8%).*

Synthesis of aminoacyl-adenosine ***17^{A}$_{Arg}$*** *upon incubating adenosine (****16A****) with thioester* ***1$_{Arg}$***

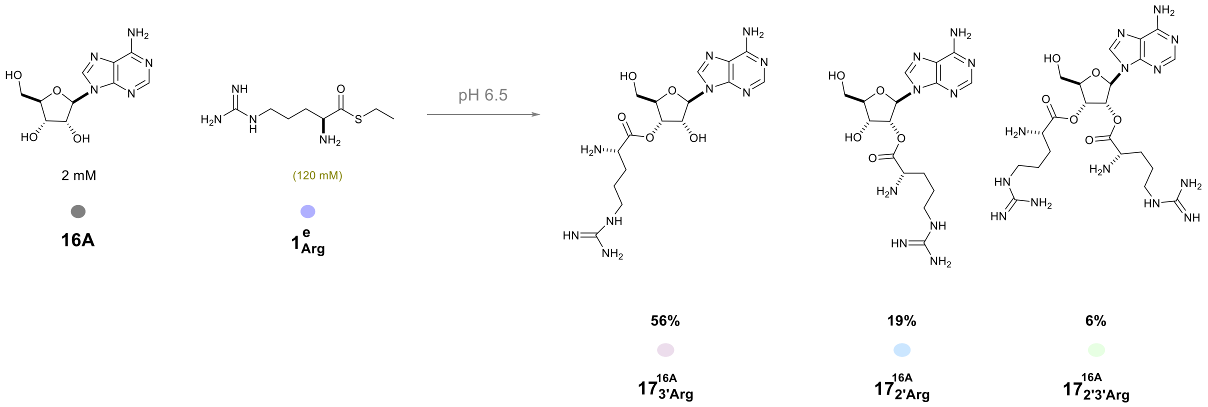

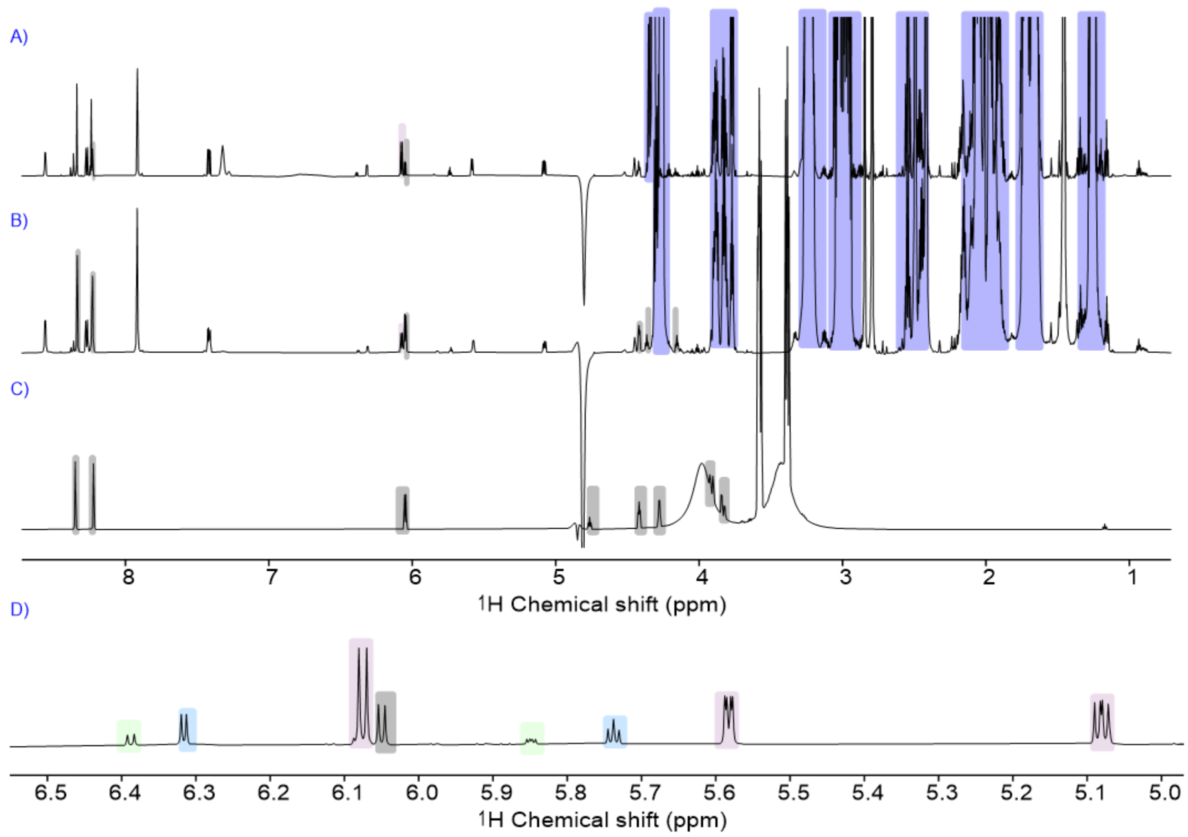

Supplementary Figure 92. ^{1}H NMR (600 MHz, H$_2$O/D$_2$O 9:1, noesygppr1d, 1.0 – 9.0 ppm) spectra to show the reaction of thioester $\mathbf{1^{e}_{Arg}}$ *(120 mM) with adenosine (****16A****, 2 mM) at pH 6.5. Set up following General Procedure C, after A) 6 h; B) 1 h. C)* ***16A*** *in MES buffer (200 mM, pH 6.5) in the absence of* $\mathbf{1^{e}_{Arg}}$*. D) 5.0 – 6.5 ppm region of spectrum A.*

^{1}H NMR (600 MHz, H$_2$O/D$_2$O 9:1), $\mathbf{17^{16A}_{3'Arg}}$ (partial assignment): δ$_H$ 6.07 (1H, d, *J* = 7.3 Hz, (C1')-H), 5.58 (1H, d, *J* = 5.4, 2.0 Hz, (C3')-H), 5.08 (1H, dd, *J* = 7.3, 5.4 Hz, (C2')-H).

^{1}H NMR (600 MHz, H$_2$O/D$_2$O 9:1), $\mathbf{17^{16A}_{2'Arg}}$ (partial assignment): δ$_H$ 6.31 (1H, d, *J* = 4.9 Hz, (C1')-H), 5.73 (1H, apt. t, *J* = 4.9 Hz, (C2')-H).

^{1}H NMR (600 MHz, H_2O/D_2O 9:1), $\mathbf{17}^{\mathbf{16A}}_{\mathbf{2',3'Arg}}$ (partial assignment): δ_H 6.38 (1H, d, *J* = 6.4 Hz, (C1')-H), 5.84 (1H, dd, *J* = 5.2, 3.0 Hz, (C3')-H).

*Synthesis of cyclic guanidine **33***

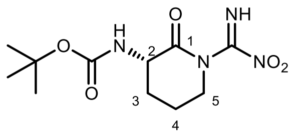

Boc-Arg(NO$_2$)

To **Boc-Arg(NO$_2$)-OH** (2.32 g, 7.27 mmol) in 9:1 EtOAc/DMF (165 mL) was added *N*,*N*'-dicyclohexylcarbodiimide (1.65 g, 8.00 mmol) under an argon atmosphere. The reaction mixture was stirred at room temperature for 16 hours, filtered and the filtrate concentrated *in vacuo*. DMF (5 mL) was added, the reaction mixture filtered again, and the filtrate concentrated to dryness by co-evaporation with toluene. The crude product was purified by column chromatography (eluting with a gradient of Et_2O/EtOAc; 100:0 to 0:100) to afford the title compound as a white foam (830 mg, 2.75 mmol, 38%). **^{1}H NMR** (400 MHz, $CDCl_3$) δ_H 5.17 (1H, br s, NH), 4.61–4.70 (1H, m, (C5)–H), 4.41–4.51 (1H, m, (C2)–H), 3.52–3.59 (1H, m, (C5)–H'), 2.43-2.52 (1H, m (C3)–H), 1.88-2.03 (2H, overlapping m, (C3)–H' + (C4)–H), 1.57-1.67 (1H, m, (C4)–H'), 1.47 (9H, s, (CO)OC(CH_3)$_3$). **^{13}C NMR** (101 MHz, $CDCl_3$) δ_C 177.1 (C1), 158.5 (**C**NNHNO$_2$/(**C**O)OC(CH_3)$_3$), 155.3 (**C**NNHNO$_2$/(**C**O)OC(CH_3)$_3$), 80.6 ((CO)O**C**(CH_3)$_3$), 52.5 (C2), 42.6 (C5), 28.3 ((CO)OC(**C**H$_3$)$_3$), 25.2 (C2), 19.7 (C3). **HRMS-ESI** $[M+H]^-$ calc. for $C_{11}H_{18}N_5O_5^-$ 300.1314; obs. 300.1302. Consistent with literature data.[7]

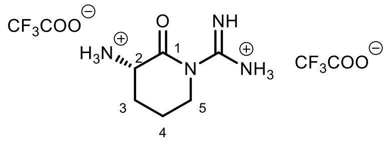

Boc-Arg(NO$_2$) (658 mg, 2.18 mmol) was dissolved in TFA/CH_2Cl_2 (15 mL, 1:1) and stirred at room temperature. After 16 hours, the reaction mixture was concentrated to dryness *in vacuo* and triturated with Et_2O to afford a hydroscopic white solid. The residue was dissolved in MeOH (15 mL). Pd/C (10%, 120 mg, 0.11 mmol) and 37% aqueous HCl (0.2 mL) were added. A balloon of H_2 was attached to the reaction vessel, the reaction mixture was twice evacuated under vacuum and backfilled with H_2. The reaction was then stirred vigorously under the H_2 atmosphere at room temperature. After 16 hours, the reaction mixture was twice evacuated under vacuum and backfilled with H_2 and subsequently heated at 50 °C for 4 hours under a H_2 atmosphere. The reaction mixture was then filtered through a layer of Celite® and concentrated to dryness to afford a yellow oil. Precipitation from ^{i}PrOH/Et_2O afforded cyclic arginine **33** as an extremely hydroscopic white solid (247 mg, 1.08 mmol, 49% over two steps). **^{1}H NMR** (700 MHz, D_2O) δ_H 4.32 (1H, dd, *J* = 7.2, 12.1 Hz, (C2)–H), 3.84-3.87 (1H, m, (C5)–H), 3.78-3.81 (1H, m, (C5)–H'), 2.40-2.44 (1H, m, (C3)–H), 2.11-2.17 (1H, m, (C4)–H), 2.04-2.10 (1H, m, (C4)–H'), 1.92-1.98 (1H, m, (C3)–H'). **^{13}C NMR** (176 MHz, D_2O) δ_C 171.4 (C1), 157.2 (CNHNH$_2$), 51.8 (C2), 47.3 (C5), 23.7 (C3), 19.8 (C4). **HRMS-ESI** $[M+H]^+$ calc. for $C_6H_{13}N_4O^+$ 157.1085; obs. 157.1084. Consistent with literature data.[8]

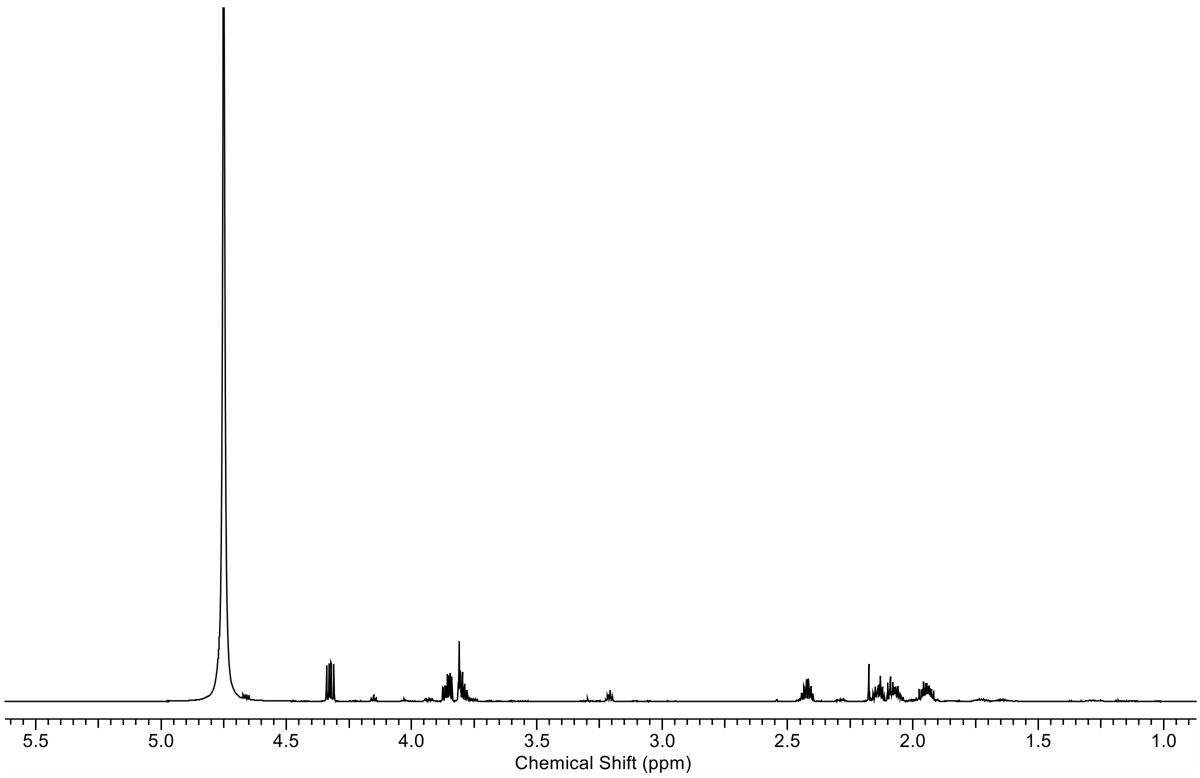

Supplementary Figure 93. ¹H NMR (700 MHz, D_2O, 1.0 – 5.5 ppm) spectrum of cyclic arginine ***33****.*

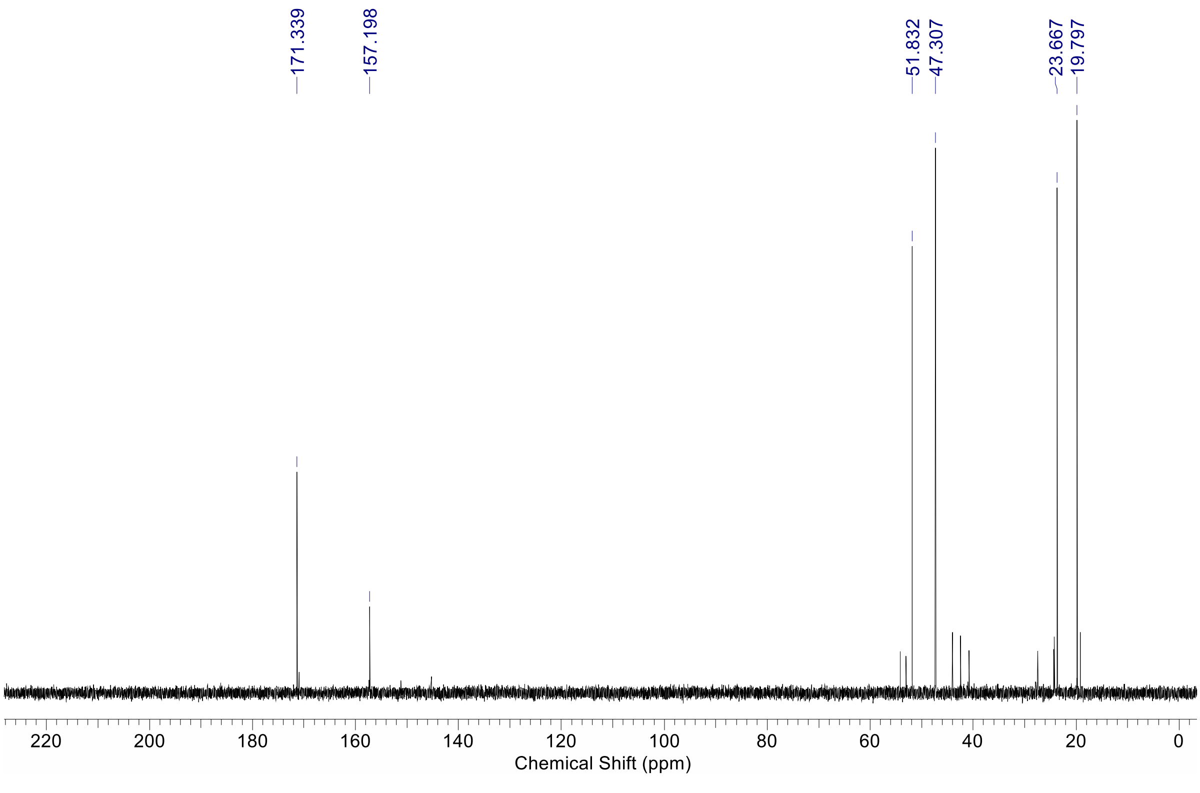

Supplementary Figure 94. ¹³C NMR (176 MHz, D_2O, 0 – 220 ppm) spectrum of cyclic arginine ***33****.*

*Synthesis of aminoacyl-adenosine **17^{A}_{Arg}** upon incubating adenosine (**16A**) with cyclic arginine **33***

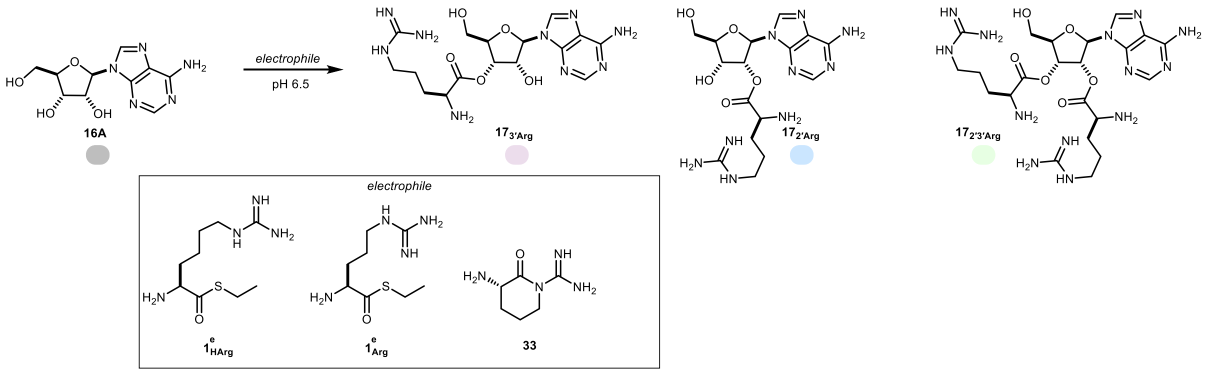

Entry	Electrophile	Time (h)	pH	$17_{3'Aaa}$ (%)	$17_{2'Aaa}$ (%)	$17_{2',3'Aaa}$ (%)	**Total** (%)
**1**	**33**	0.5	6.5	27	12	1	40
**2**	**33**	0.3	6.0	24	9	1	34
**3**	$\mathbf{1^{e}_{Arg}}$	1	6.0	7	2	n.d.	9
**4#**	$\mathbf{1^{e}_{HArg}}$	1	6.5	<1	--	--	<1

*Supplementary Table 13. Yields (%) of aminoacyl-nucleosides **17^{A}_{Arg}** following the reaction of **1^{e}_{Arg}** or cyclic arginine **33** (40 mM) with adenosine **16A** (20 mM) at pH 6.0 and room temperature. # = the reaction of **1^{e}_{HArg}** (30 mM) with adenosine (**16A**, 20 mM) at pH 6.5. No significant rate acceleration was observed for the arginine homolog. n.d. = not detected.*

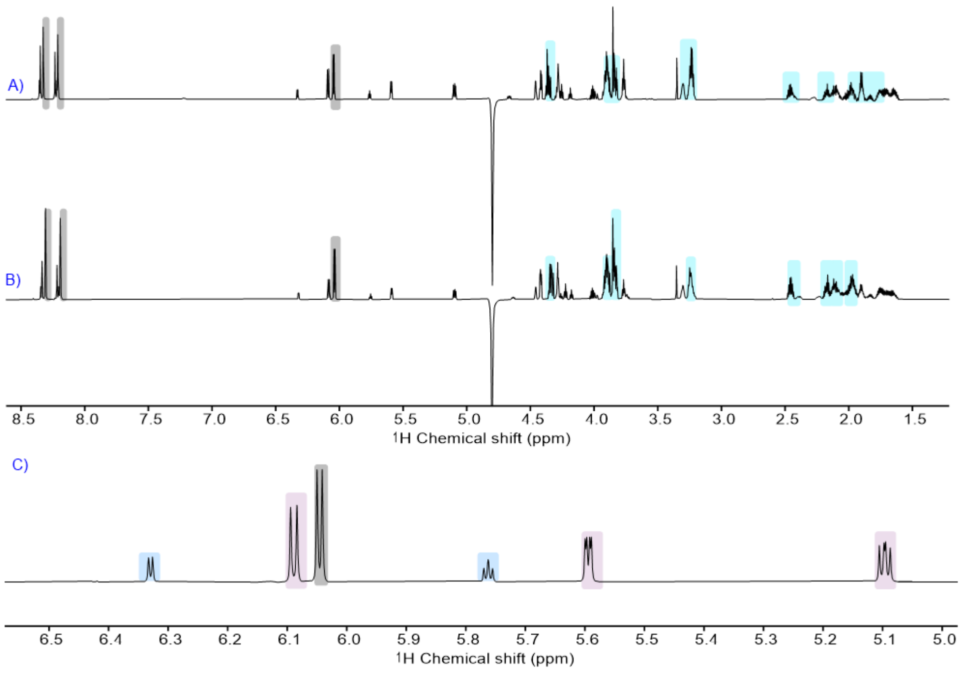

*Supplementary Figure 95. ^{1}H NMR (600 MHz, H_2O/D_2O 9:1, noesygppr1d, 1.5 – 8.5 ppm) spectra to show the reaction of cyclic arginine **33** (40 mM) with adenosine (**16A**, 20 mM) at pH 6.0. Set up following General Procedure C, after A) 4 h; B) 0.5 h. C) 5.0 – 6.5 ppm region of A.*

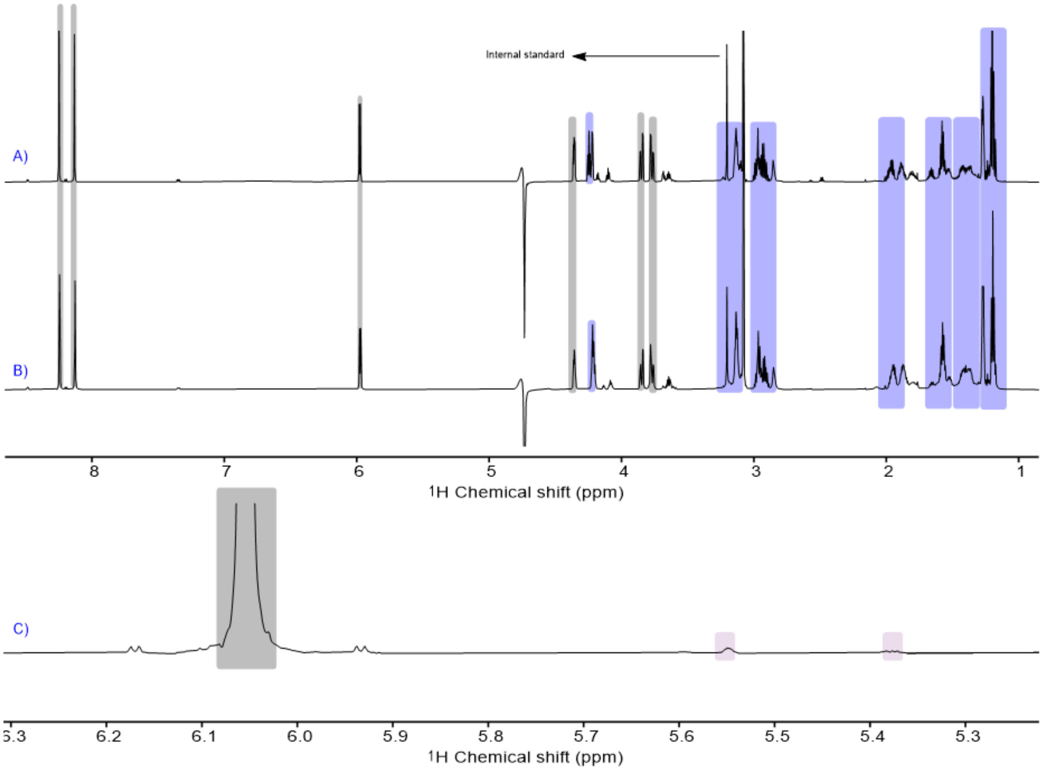

Supplementary Figure 96: ¹H NMR (600 MHz, H_2O/D_2O 9:1, noesygppr1d, 1.0 – 9.0 ppm) spectra to show the reaction of $\mathbf{1^{e}_{HArg}}$ *(30 mM) with adenosine (**16A**, 20 mM) at pH 6.5. Set up following General Procedure C, after A) 16 h; B) 0.5 h. C) 5.3 – 6.3 ppm region of B.*

Synthesis of aminoacyl-adenosine ***17****$^{A}_{Arg}$ *upon incubating adenosine* ***16A*** *with varying amounts of cyclic arginine* ***33***

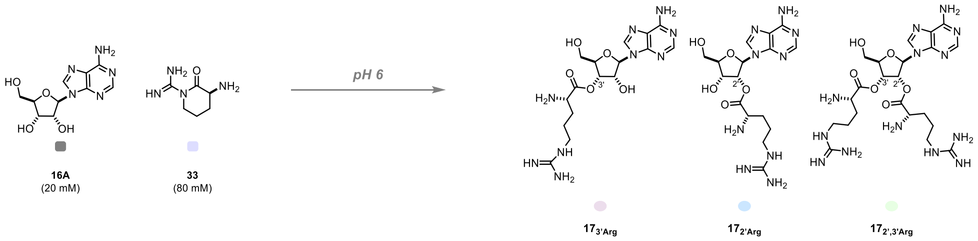

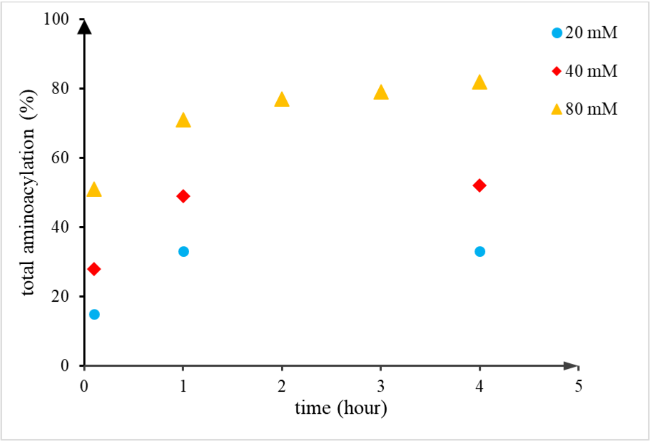

Supplementary Figure 97: Total yields (%) of aminoacyl-nucleosides $\mathbf{17^{A}_{Arg}}$ *from the reaction of cyclic arginine* ***33*** *(varying concentration at 20 mM, 40 mM, and 80 mM) with adenosine* ***16A*** *(20 mM) in 0.5 M MES buffer at pD 6.0 and room temperature.*

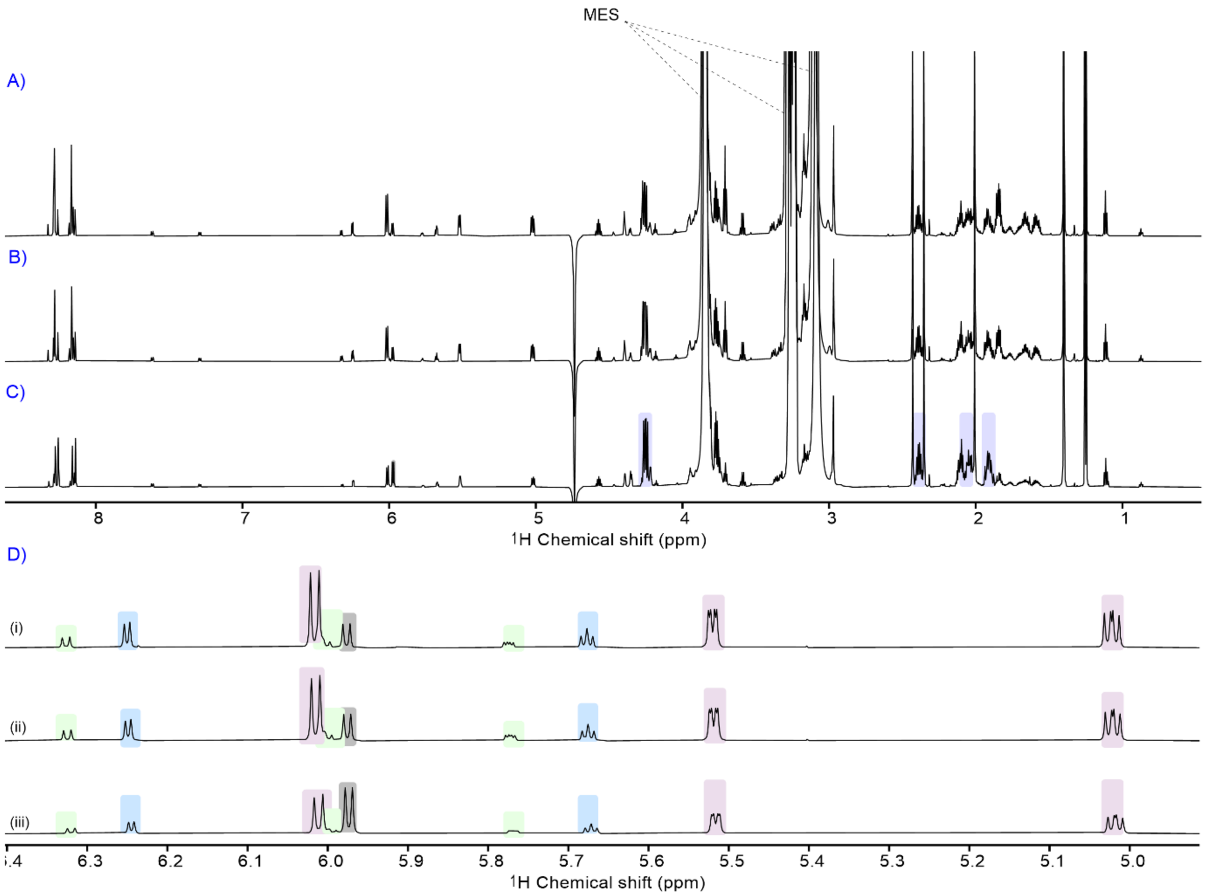

Supplementary Figure 98: ^{1}H NMR (700 MHz, D_2O, noesygppr1d, 1.0 – 9.0 ppm) spectra to show the reaction ***33*** *(80 mM) with adenosine (**16A**, 20 mM) at pD 6.0. Set up following General Procedure C, after A) 3 h; B) 2 h; C) 0.2 h; D) zoom in spectra A → (i), B → (ii), and C → (iii) between 4.8–6.5 ppm, with starting material and product signals highlighted.*

*Coupling of cyclic arginine **33** with 2-mercaptoethanesulfonate **5c** at pH 6.5 and room temperature*

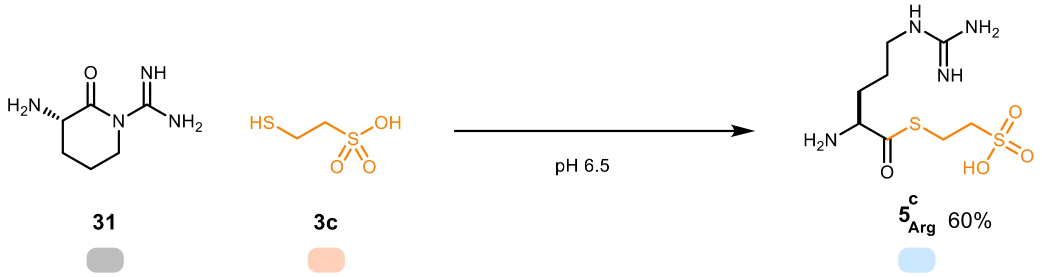

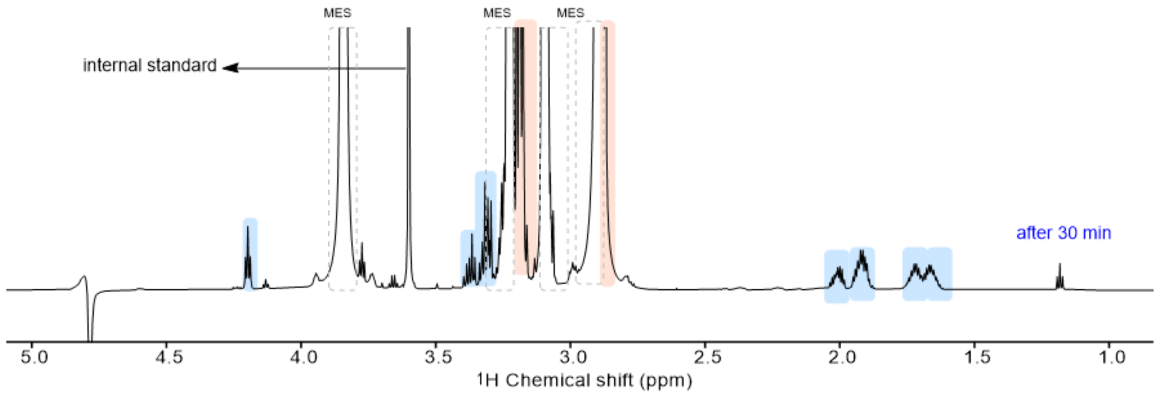

Supplementary Figure 99. ¹*H NMR (600 MHz, H_2O/D_2O 9:1, noesygppr1d, 1.0 – 5.0 ppm) spectrum to show the reaction of cyclic arginine **33** (20 mM) with 2-mercaptoethanesulfonate (**5c**, 100 mM) in MES buffer (200 mM, pH 6.5), with PET (10 mM) as an internal standard after 30 minutes at room temperature.*

¹H NMR (600 MHz, H_2O/D_2O 9:1) **1**$^{c}_{\mathbf{Arg}}$ (partial assignment) : δ_H 4.20 (1H, t, *J* = 6.2 Hz, Arg-α-C*H*COSCH₂), 3.35 – 3.28 (2H, m, COSC*HH*), 1.5 – 2.0 (4H, m, Arg-α-CHC*H₂*C*H₂*CH₂-gaunidinium).

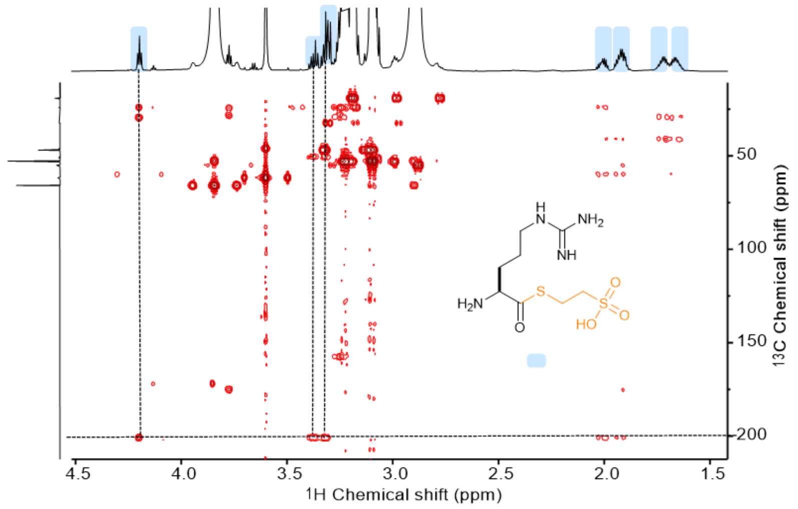

Supplementary Figure 100: ¹*H–*¹³*C HMBC (*¹*H: 600 MHz [2.5-8.0 ppm],* ¹³*C: 176 MHz [20-220 ppm], H_2O/D_2O 9:1) spectrum showing the diagnostic* ²*JCH and* ³*JCH coupling of argininyl-αH and -SCH_2 in **1**$^{c}_{Arg}$ at 4.20 ppm, 3.38 ppm with a resonance at 199.6 ppm, which is characteristic of amino thioester bond formation.*

*Aminoacylation of uridine (**16U**) upon reaction with different thioester **1**$^{n}_{Ala}$*

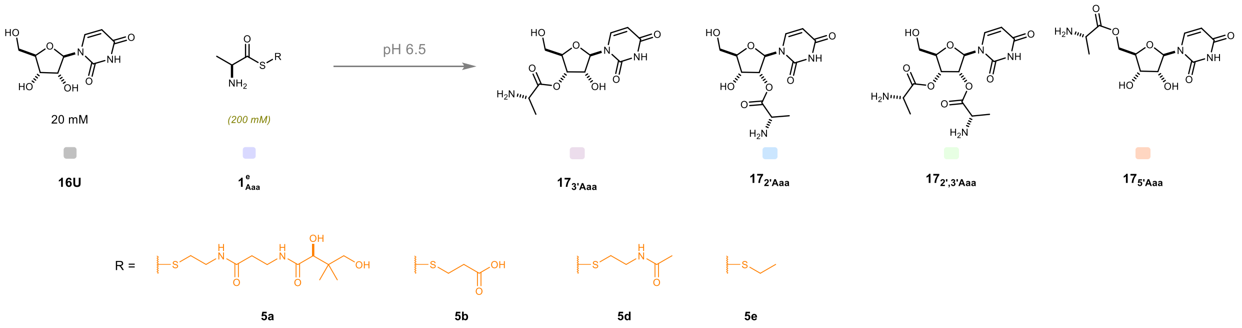

Entry	1^{a-e}_{Ala} (mM)	**17**$_{3'Aaa}$ (%)	**17**$_{2'Aaa}$ (%)	**17**$_{2',3'Aaa}$ (%)	**17**$_{5'Aaa}$ (%)	**Total** (%)
1	1^{d}_{Ala} (200)	16	9	3	1	29
2	1^{a}_{Ala} (200)	16	8	3	1	28
3	1^{b}_{Ala} (200)	10	5	1	--	17
4	1^{e}_{Ala} (200)	14	7	3	1	25
5[a]	1^{e}_{Ala} (200)	9	4	1	1	15

*Supplementary Table 14. Yields of 2'-, 3'-, 2',3'- and 5'-aminoacyl-nucleoside **17**$_{Ala}$ after the reaction of specified concentration of alanine thioester 1^{a-e}_{Ala} with uridine (**16U**, 20 mM) at 6.5 and after 18 hours at room temperature. Set up following General Procedure C. a = Uridine (**16U**, 20 mM) with 1^{e}_{Ala} (200 mM) at pH 6.5 with 1.8 M NaOAc buffer after 18 hours at room temperature.*

*Competition reaction of uridine (**16A**) with thioester **1**$_{Ala}$ and amido thioester **39**$_{Ala}$*

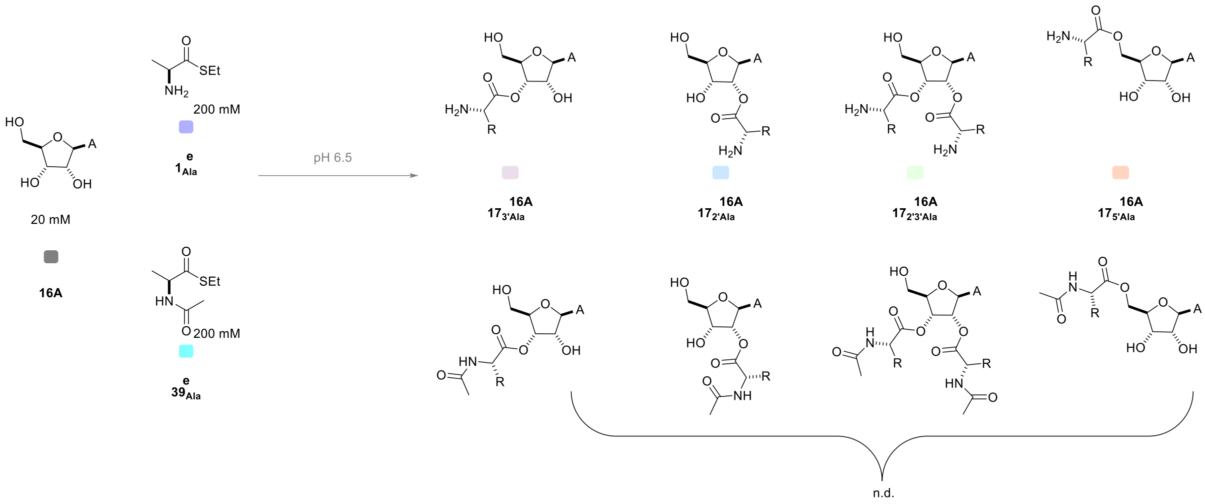

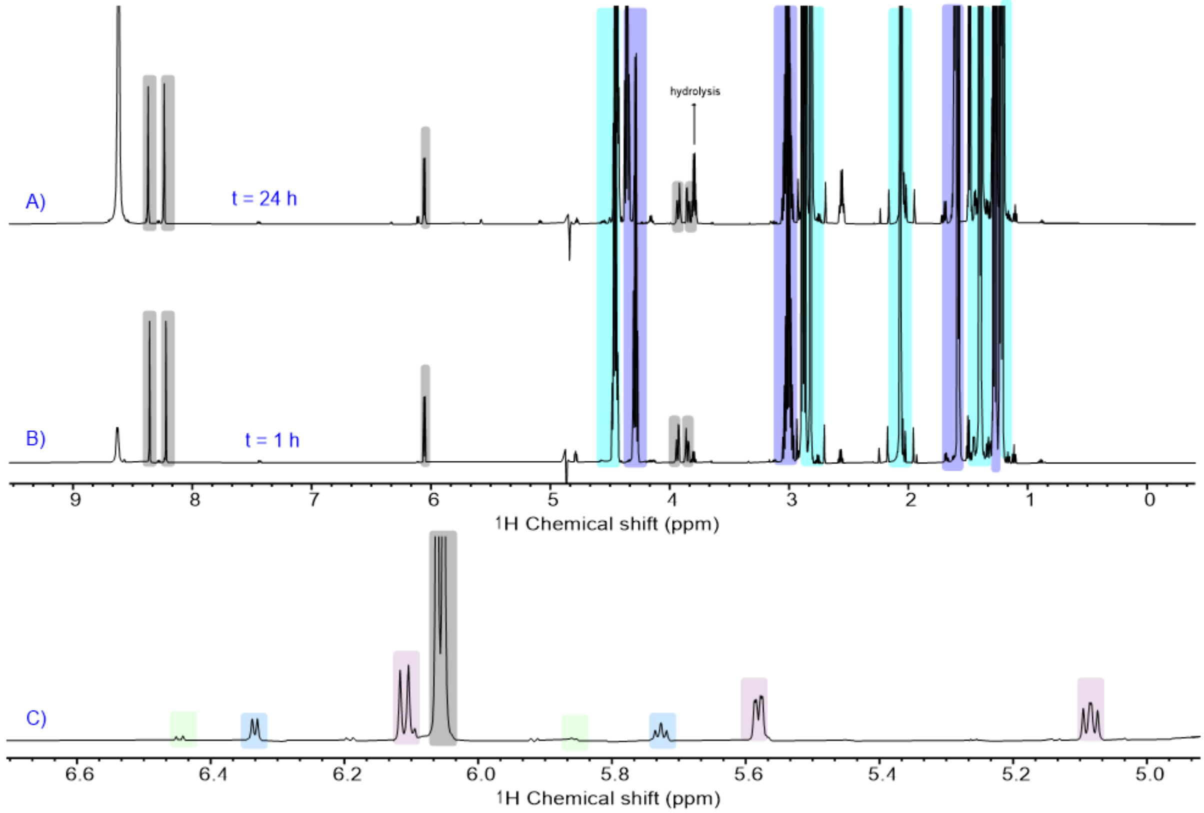

Supplementary Figure 101. ^{1}*H NMR (600 MHz,* H_2O/D_2O *9:1, noesygppr1d, 0 – 9.0 ppm) spectra to show the reaction of thioester* $\mathbf{1^{e}_{Ala}}$ *(H-Ala-SEt, 200 mM) and amido thioester* $\mathbf{39^{e}_{Ala}}$ *(Ac-Ala-SEt, 200 mM) with adenosine (**16A**, 20 mM) at pH 6.5, using PET (10 mM) as an internal standard after A) 24 h; B) 1 h; C) 5.0–6.6 ppm region of spectrum A). Set up following General Procedure C.*

Aminoacylation of RNA oligomers

Purification of RNA oligomers

All oligonucleotides (**ON**) were purchased from *Integrated DNA Technologies Inc.* (IDT) and purified on either a 20 or 30% (19:1) polyacrylamide gel. The bands containing the desired oligomer were excised and the oligomer was eluted with 500 mM NaOAc for 16 – 18 hours. The oligomer was then precipitated from EtOH/H_2O (92:8) at 4 °C, the pellet washed with EtOH/H_2O (92:8), dried at ambient pressure and redissolved in water (1 mL). Oligomer solution concentration was calculated from A_{260} (10 mm path) values determined on a NanoDrop spectrophotometer.

Polyacrylamide gel electrophoresis (PAGE) procedure

Samples were run on a 20% (19:1) denaturing polyacrylamide gel in 1 × EA buffer pre-cooled to 1 °C with an IKA RC 2 lite S002 Recirculating Chiller. The gel was then shaken in 1 × TBE buffer for 5-10 minutes in order to deprotonate FAM-fluorophores to enable fluorescence at 473 nm. The gel was then immediately scanned on a Typhoon FLA 9500 scanner (Mode = Fluorescence, Method = [FAM], Laser = 473 nm, Filter = [LPB] (ch.1), PMT Type = Bialkali, PMT = ch.1, 700V, Stage = Fluorescence Stage), and the bands were quantified using ImageJ software. % yield of aminoacylation was found to be invariant to quantification by ImageJ, ImageQuantTL or by fitting Gaussian curves to extracted intensity plots.

1 × EA buffer = 100 mM NaOAc, 2 mM EDTA, pH 5.2.

1 × TBE buffer = 89 mM Tris, 89 mM borate, 2 mM EDTA, pH 8.3.

20% (19:1) denaturing polyacrylamide gel = 36% w/v urea, 20% (19:1) acrylamide, 10% 10×EA.

General procedure D: Analysis of non-template-directed aminoacylation of oligonucleotides

At each required time point, an aliquot (1−2 μL) was removed from the reaction and a quenching buffer (16 μL) containing 10 mM EDTA, 150 mM sodium acetate and 93% (v/v) formamide was added. Samples were then run according to PAGE procedure (above).

General procedure E: Analysis of template-directed aminoacylation of oligonucleotides

At each required time point, an aliquot (1–2 μL) was removed and a competitor oligomer (30 equiv. with respect to FAM-tagged oligomer in the aliquot) added, resulting in a single-stranded FAM-tagged oligomer. The solution was left for 5–10 mins, and a quenching buffer (16 μL) containing 10 mM EDTA, 150 mM sodium acetate and 93% (v/v) formamide was then added. Samples were then run according to PAGE procedure (above).

Sequence nomenclature

	**Substrates**	
**ON1**	5'-FAM-dUdAdGdGdAdGdAdGdCddC	
**ON2**	5'-FAM-dUdAdGdGdAdGdAdCdCdA	
**ON3**	5'-FAM-dUdAdGdGdAdGdAdCdCrA	
**ON4**	5'-FAM-rUrArGrGrArGrArCrCdA	
**ON5**	5'-FAM-rUrArGrGrArGrArCrCrA	
**ON14**	5'-FAM-rUrArGrGrArGrArGrCrA	
**ON15**	5'-FAM-rUrArGrGrArGrArGrCrU	
**ON16**	5'-FAM-rUrArGrGrArGrArGrCrC	
**ON17**	5'-FAM-rUrArGrGrArGrArGrCrG	
**ON10**	5'-FAM-rUrGrArGrArGrArGrCrCrU	
**ON21**	5'-FAM-rUrCrGrCrUrUrUrCrCdA	
**ON22**	5'-FAM-rUrCrGrCrUrUrUrCrCrA	
**ON5-3'p**	5'-FAM-rUrArGrGrArGrArCrCrA-3'-phos	
	**Templates**	
**ON11**	5'-rArGrGrCrUrCrUrCrUrCrA	
**ON12**	5'-GrGrCrUrCrUrCrUrCrA	
**ON13**	5'-rGrUrGrGrCrUrCrUrCrUrCrA	
**ON2-3-complement, ON19**	5'-dUdGdGdUdCdUdCdCdUdA	
**ON4-5-complement, ON9**	5'-rUrGrGrUrCrUrCrCrUrA	
**ON5-complement**	5'-rUrGrCrUrCrUrCrCrUrA	
**ON6-complement**	5'-rArGrCrUrCrUrCrCrUrA	
**ON7-complement**	5'-rGrGrCrUrCrUrCrCrUrA	
**ON8-complement,**	5'-rCrGrCrUrCrUrCrCrUrA	
**ON11-12-complement**	5'-rUrGrGrArArArGrCrGrA	
**15merRNA, ON7**	5'-rGrCrArGrUrUrGrGrUrCrUrCrCrUrA	
**15merDNA, ON6**	5'-dGdCdAdGdUdUdGdGdUdCdUdCdCdUdA	
**15merDNA-I ON20**	5'-dGdCdAdGdUdGdGdCdUdCdUdCdCdUdA	
**5mer, ON8**	5'-phos-rArCrUrGrC	
**5mer-I, ON23**	5'-phos-rArGrCrGrA	
**5merOH, ON18**	5'-rArCrUrGrC	
**Competitors**	**Sequence**	**For use with**
**CompetitorA**	5'-rUrArGrGrArGrArCrCrArArCrUrGrC	**15merRNA**
**CompetitorB**	5'-dUdAdGdGdAdGdAdCdCdAdAdCdUdGdC	**15merDNA**
**CompetitorC**	5'-rUrArGrGrArGrArCrCrA	**ON4-5-complement**
**CompetitorD**	5'-dUdAdGdGdAdGdAdCdCdA	**ON2-3-complement**
**CompetitorE**	5'-rUrArGrGrArGrArGrCrCrArCrUrGrC	**15merDNA-I**

Supplementary Table 15: Substrate, template and competitor oligonucleotide names and their sequences.

*General Procedure F: Non-template-directed aminoacylation of oligonucleotides by aminoacyl thiols **1***

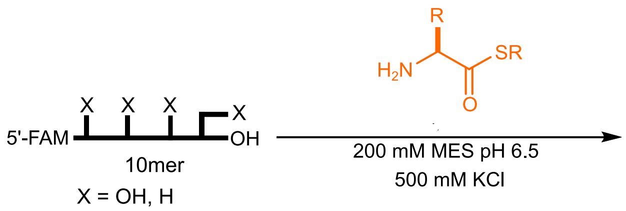

The specified oligonucleotide (**ON#**; 10 µM, 0.5 µL) was added to a solution containing 2.5 M KCl and 1 M MES at pH 6.5 (2 µL). The reaction mixture was diluted with H_2O (2.5 µL) and the specified thioester pre-adjusted to pH 6.5 (400 mM, 5 µL) was added to initiate the reaction. The final reaction mixture (10 µL) contained 0.5 µM RNA substrate, 200 mM MES pH 6.5, 500 mM KCl and 200 mM thioester. The reaction mixture was vortexed, briefly centrifuged and incubated at room temperature. The solution was analysed by General Procedure D.

*General Procedure G: Template-directed aminoacylation of oligonucleotides by aminoacyl thiols **1***

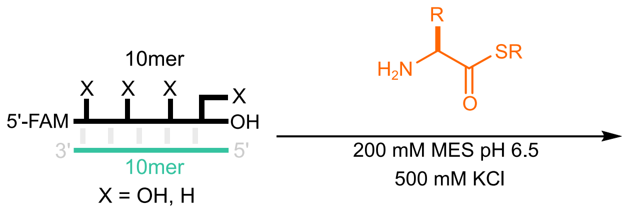

The specified oligonucleotide substrate (**ON#**; 10 µM, 0.5 µL) and its complementary template (10 µM, 0.6 µL) were added to a solution containing 2.5 M KCl and 1 M MES pH 6.5 (2 µL). The reaction mixture was diluted with H_2O (1.90 µL) and the specified thioester pre-adjusted to pH 6.5 (400 mM, 5 µL) added to initiate the reaction. The final reaction mixture (10 µL) contained 0.5 µM RNA substrate, 0.6 µM template, 200 mM MES pH 6.5, 500 mM KCl and 200 mM thioester. The reaction mixture was vortexed, briefly centrifuged and incubated at room temperature. The solution was analysed by General Procedure E.

*General Procedure H: Aminoacylation in a nicked duplex construct with aminoacyl thiols **1***

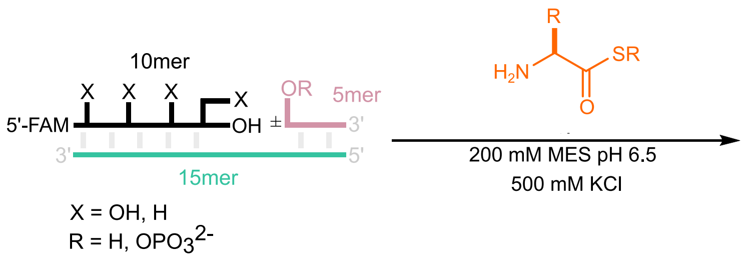

The specified oligonucleotide substrate (**ON#**; 10 µM, 0.5 µL), 15mer template (10 µM, 0.6 µL) and 5mer (10 µM, 0.6 µL) were added to a solution containing 2.5 M KCl and 1 M MES pH 6.5 (2 µL). The specified thioester pre-adjusted to pH 6.5 (400 mM, 5 µL) was added, and the reaction mixture diluted with H_2O to 10 µL. The final reaction mixture (10 µL) contained 0.5 µM RNA substrate, ±0.6 µM 15mer-template, ±0.6 µM 5mer, 200 mM MES pH 6.5, 500 mM KCl and 200 mM thioester. The solution was analysed by General Procedure E.

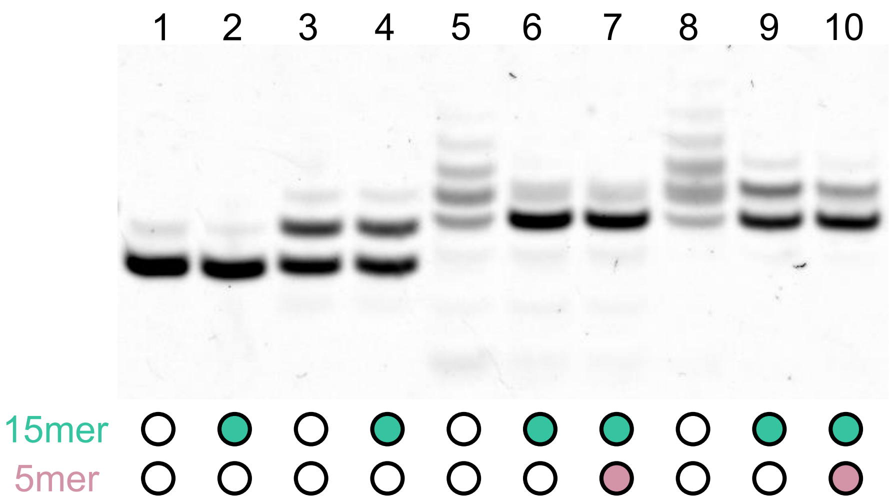

*Supplementary Figure 102. PAGE [expanded from Figure 5 in main text] to show the reaction of L-alanyl (R)-pantetheinyl thioester (L-$\mathbf{1}^{a}_{\mathbf{Ala}}$, 600 mM) in MES buffer (200 mM, pH 6.5) and KCl (500 mM) with a FAM-tagged RNA oligomer **ON2-5** in the presence or absence of the specified 15mer template (0.6 µM) and 5mer (0.6 µM) after 16 h at rt. The solution was analysed by general procedure G (ss)/H (ds). Lanes 1-2: **ON2** (5'-FAM-dUdAdGdGdAdGdAdCdCdA). Lanes 3-4: **ON3** (5'-FAM-dUdAdGdGdAdGdAdCdCrA). Lanes 5-7: **ON4** (5'-FAM-rUrArGrGrArGrArCrCdA). Lanes 8-10: **ON5** (5'-FAM-rUrArGrGrArGrArCrCrA). For the DNA-substrates **ON2** and **ON3**, a DNA template (**15merDNA, ON6**) was used. For the RNA-substrates **ON4** and **ON5**, an RNA template (**15merRNA, ON7**) was used. **5mer, ON8** (5'-phos-ACUGC).*

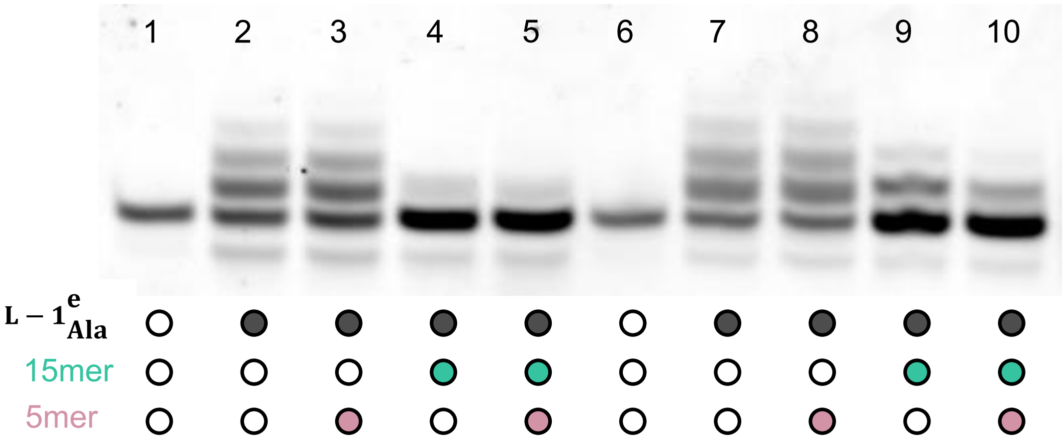

*Supplementary Figure 103. PAGE to show the effect of templating and nick-formation during non-enzymatic aminoacylation. An RNA with a 3'-deoxy-A at the 3'-terminus **ON4** (0.5 µM, lanes 1–5, 5'-FAM-rUrArGrGrArGrArCrCdA) or all-RNA oligomer **ON5** (0.5 µM, lanes 6–10, 5'-FAM-rUrArGrGrArGrArCrCrA) were reacted with L-$\mathbf{1}^{e}_{\mathbf{Ala}}$ (200 mM) in MES buffer (200 mM, pH 6.5) and KCl (500 mM) for 16 h at rt (lanes 2 and 7). The solution was analysed by general procedure G (ssRNA)/H (dsRNA). The addition of a downstream **5merOH, ON18** (0.6 µM, 5'-rArCrUrGrC) did not influence the aminoacylation reaction (lanes 3 and 8), but addition of a 15mer complement **15merRNA, ON7** (0.6 µM, 5'-rGrCrArGrUrUrGrGrUrCrUrCrCrUrA) led to suppression of reactivity for **ON4** whilst good yields of aminoacylation for **ON5** (lanes 4 and 9) were observed. Inclusion of both **5merOH, ON18** and **15merRNA, ON7** led to similar results (lanes 5 and 10).*

**ON4**		**ON5**	
Lane	% aminoacylation	Lane	% aminoacylation
2	61	7	65
3	62	8	65
4	12	9	33
5	9	10	20

*Supplementary Table 16. % conversion of aminoacylation as determined by PAGE in the reaction of the specified FAM-tagged RNA oligomer (0.5 µM) with L-$\mathbf{1}^{e}_{\mathbf{Ala}}$ (200 mM) in MES buffer (200 mM, pH 6.5) and KCl (500 mM) at 23 °C after 1 day in the presence or absence of **15merRNA, ON7** (0.6 µM, 5'-rGrCrArGrUrUrGrGrUrCrUrCrCrUrA) and a downstream **5merOH, ON18** (0.6 µM, 5'-rArCrUrGrC).*

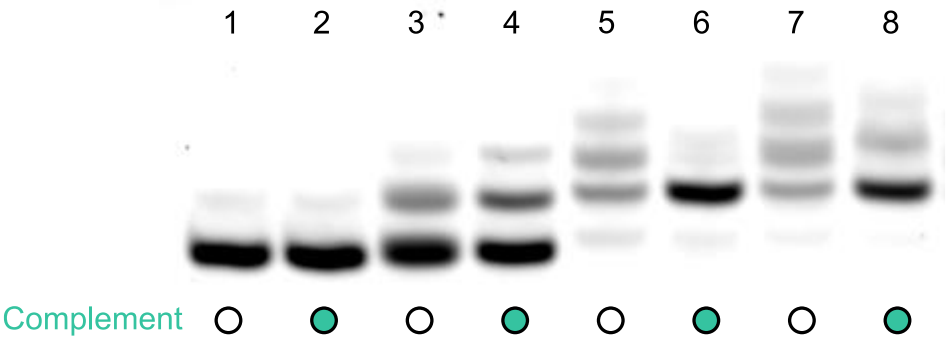

Supplementary Figure 104. PAGE to show the reaction of L-alanyl ethanethiol thioester (L-$\mathbf{1}^{e}_{\mathbf{Ala}}$, 200 mM) in MES buffer (200 mM, pH 6.5) and KCl (500 mM) with a FAM-tagged RNA oligomer in the presence or absence of the specified 10mer template (0.6 µM) after 16 h at rt. The solution was analysed by general procedure G (ss)/H (ds). Lanes 1-2: ***ON2*** *(5'-FAM-dUdAdGdGdAdGdAdCdCdA). Lanes 3-4:* ***ON3*** *(5'-FAM-dUdAdGdGdAdGdAdCdCrA). Lanes 5-6:* ***ON4*** *(5'-FAM-rUrArGrGrArGrArCrCdA). Lanes 7-8:* ***ON5*** *(5'-FAM-rUrArGrGrArGrArCrCrA). For the DNA-substrates* ***ON2*** *and* ***ON3****, a DNA template (****ON2-3-complement, ON19****) was used. For the RNA-substrates* ***ON4*** *and* ***ON5****, an RNA template (****ON4-5-complement, ON9****) was used.*

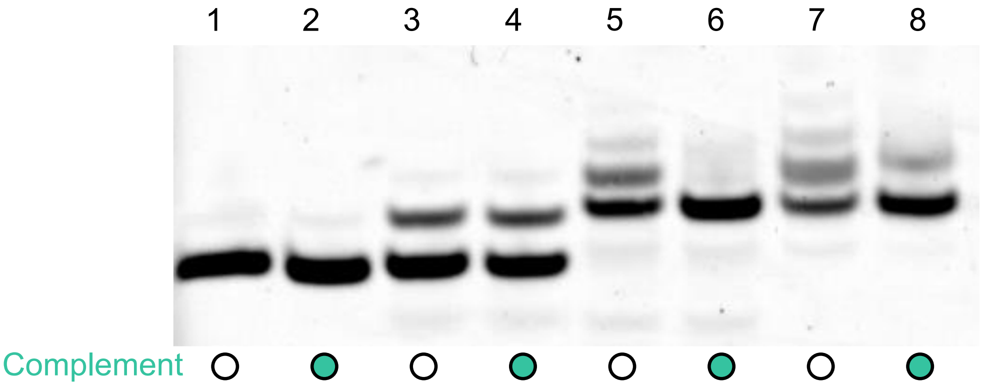

Supplementary Figure 105. PAGE to show the reaction of L-alanyl (R)-pantetheinyl thioester (L-$\mathbf{1}^{a}_{\mathbf{Ala}}$, 200 mM) in MES buffer (200 mM, pH 6.5) and KCl (500 mM) with a FAM-tagged RNA oligomer (0.5 µM) in the presence or absence of the specified 10mer template (0.6 µM) after 16 h at rt. Lanes 1-2: ***ON2*** *(5'-FAM-dUdAdGdGdAdGdAdCdCdA). The solution was analysed by general procedure G (ss)/H (ds). Lanes 3-4:* ***ON3*** *(5'-FAM-dUdAdGdGdAdGdAdCdCrA). Lanes 5-6:* ***ON4*** *(5'-FAM-rUrArGrGrArGrArCrCdA). Lanes 7-8:* ***ON4*** *(5'-FAM-rUrArGrGrArGrArCrCrA). For the DNA-substrates* ***ON2*** *and* ***ON3****, a DNA template (****ON2-3-complement, ON19****) was used. For the RNA-substrates* ***ON4*** *and* ***ON5****, an RNA template (****ON4-5-complement, ON9****) was used.*

		L-$\mathbf{1}^{e}_{Ala}$		D-$\mathbf{1}^{e}_{Ala}$		L-$\mathbf{1}^{a}_{Ala}$		L-$\mathbf{1}^{e}_{Arg}$*	
Substrate	Sequence	-	+	-	+	-	+	-	+
**ON2**	5'FAM-dUdAdGdGdAdGdAdCdCdA	7	6	6	5	4	3	5	3
**ON3**	5'FAM-dUdAdGdGdAdGdAdCdCrA	31	32	49	57	28	31	33	34
**ON4**	5'FAM-rUrArGrGrArGrArCrCA	53	14	95	25	49	8	71	15
**ON5**	5'FAM-rUrArGrGrArGrArCrCrA	55	31	95	49	58	33	70	33

Supplementary Table 17. % conversion of aminoacylation as determined by PAGE in the reactions of the specified FAM-tagged RNA oligomer ***ON2-5*** *(0.5 µM) with the specified thioester (200 mM) in MES buffer (200 mM, pH 6.5) and KCl (500 mM) after 16 h at rt with (+) or without (-) the presence of the specified 10mer template (0.6 µM). *Reaction carried out with 25 mM L-$\mathbf{1}^{e}_{\mathbf{Arg}}$. Ribonucleotides are underlined for clarity.*

		15mer	
Substrate	Sequence	-	+
**ON2**	5'FAM-dUdAdGdGdAdGdAdCdCdA	9	7
**ON3**	5'FAM-dUdAdGdGdAdGdAdCdCrA	45	45
**ON4**	5'FAM-rUrArGrGrArGrArCrCdA	72	18 (17)
**ON5**	5'FAM-rUrArGrGrArGrArCrCrA	80	44 (34)

*Supplementary Table 18. % conversion of aminoacylation as determined by PAGE in the reactions of the specified FAM-tagged RNA oligomer **ON2-5** (0.5 µM) with L-$\mathbf{1}^{a}_{Ala}$ (600 mM) in MES buffer (200 mM, pH 6.5) and KCl (500 mM) after 16 h at rt with (+) or without (-) the presence of the specified 15mer template **15merRNA, ON7/15merDNA, ON6** (0.6 µM). Indicated in brackets is %-aminoacylation for 15mer reaction in the presence of **5mer, ON8** (0.6 µM, 5'phos-rArCrUrGrC). Ribonucleotides are underlined for clarity.*

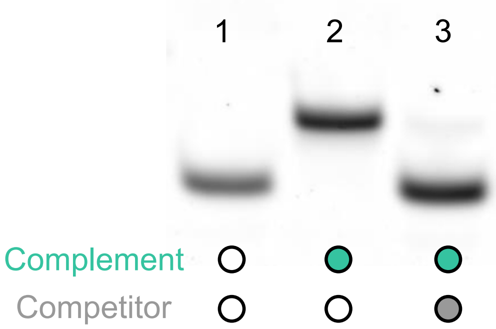

*Supplementary Figure 106. PAGE showing the effect of the addition of a competitor oligomer (**Competitor C**, 30 equiv.) to a **ON5:ON9 (ON4-5-complement)** duplex. The addition of a competitor oligomer enables visualisation of unduplexed FAM-tagged substrates and their unduplexed aminoacylated products on cold and acidic 20% polyacrylamide gels.*

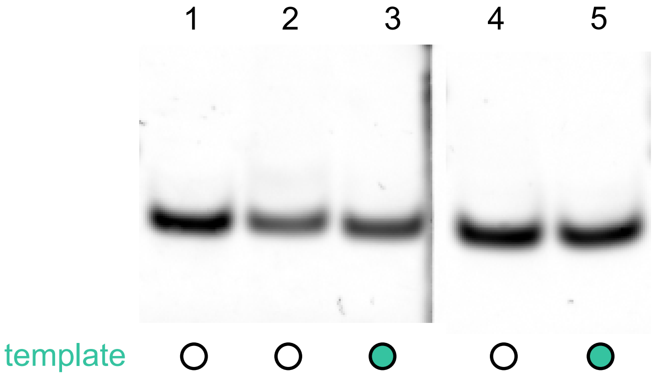

*Supplementary Figure 107. PAGE to show perdeoxy oligonucleotide **ON1** (lane 1, 5'-FAM-dUdAdGdGdAdGdAdGdCddC) and the reaction of **ON1** (0.5 µM) in the presence and absence of template **15merDNA-I, ON20** (0.6 µM) with either L-alanyl ethanethiol thioester (L-$\mathbf{1}^{e}_{Ala}$, 200 mM, lanes 2–3) or L-arginyl ethanethiol thioester (L-$\mathbf{1}^{e}_{Arg}$, 60 mM, lanes 4–5) in MES buffer (200 mM, pH 6.5) and KCl (500 mM) after 16 h at rt. The solution was analysed by general procedure D (ssDNA)/H (dsDNA).*

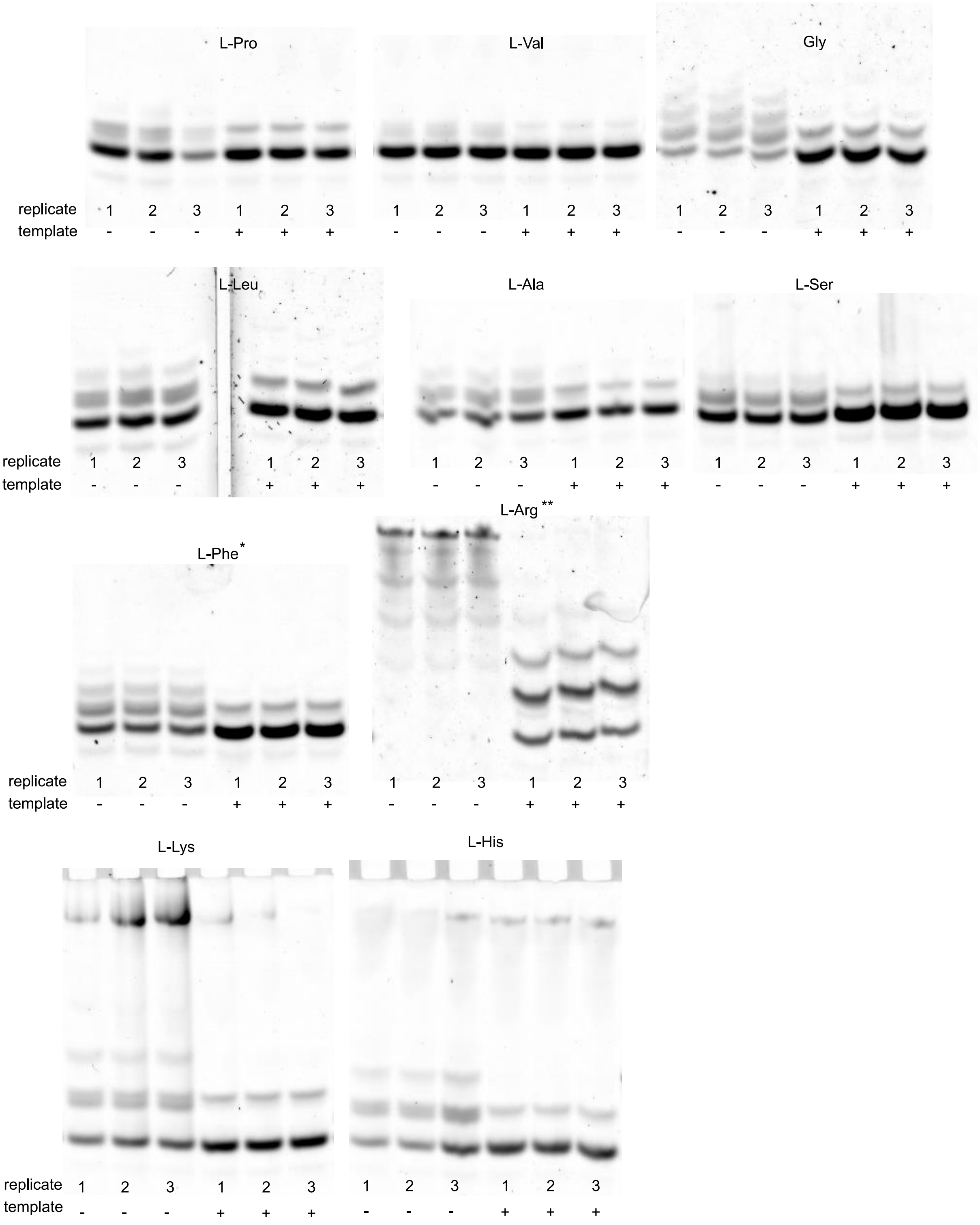

*Supplementary Figure 108. PAGE to show triplicates of the reaction of **ON5** (0.5 μM, 5'-FAM-rUrArGrGrArGrArCrCrA) with the specified $L-\mathbf{1}^{\mathbf{e}}_{\mathbf{Aaa}}$ (200 mM) in the presence (+) or absence (-) of 15mer template **15merRNA, ON7** (0.6 μM, 5'-rGrCrArGrUrUrGrGrUrCrUrCrCrUrA) in MES buffer (400 mM, pH 6.5) and KCl (1 M) after 16 h at rt. The solution was analysed by general procedure D (ssRNA)/H (dsRNA) . *100 mM $L-\mathbf{1}^{\mathbf{e}}_{\mathbf{Phe}}$. **60 mM $L-\mathbf{1}^{\mathbf{e}}_{\mathbf{Arg}}$. Slower running bands observed in the reactions of $L-\mathbf{1}^{\mathbf{e}}_{\mathbf{Lys}}$ and $L-\mathbf{1}^{\mathbf{e}}_{\mathbf{His}}$ were not included in quantification for aminoacylation % and will be the subject of future investigation.*

	Val	Pro	Lys	Ser*	His	Leu	Ala	Ala**	Gly	Phe	Arg†
ssRNA	12	24	34	42	45	45	60	51	62	71	>95
dsRNA	6	12	27	10	15	20	25	21	17	10	69

*Supplementary Table 19. % conversion of aminoacylation as determined by PAGE in the reactions of the RNA oligomer **ON5** (0.5 µM, 5'-FAM-rUrArGrGrArGrArCrCrA) in the presence and absence of **15merRNA, ON7** (0.6 µM, 5'-rGrCrArGrUrUrGrGrUrCrUrCrCrUrA) with the specified thioester L-$\mathbf{1}^{e}_{\mathbf{Aaa}}$(200 mM) in MES buffer (200 mM, pH 6.5) and KCl (500 mM) after 16 h at rt .*DL-$\mathbf{1}^{e}_{\mathbf{Ser}}$. ** L-alanyl (R)-pantetheinyl thioester (L-$\mathbf{1}^{a}_{\mathbf{Ala}}$, 200 mM). †60 mM L-$\mathbf{1}^{e}_{\mathbf{Arg}}$.*

L-$\mathbf{1}^{e}_{\mathbf{Aaa}}$	Val	Pro	Ser	Lys	Leu	Ala	Phe*	His	Gly	Arg†
ssRNA 1	14.5	32.5	45.7	50.3	46	48.5	53.8	55.8	66	100
2	15	34.1	47.8	46	49	46.7	53.9	54.2	69.9	100
3	14.1	37	46.4	46.5	49	49.1	54.5	55.1	67.4	100
mean	15±0.5	35±2.3	36±0.3	48±2.4	48±1.7	48±1.2	54±0.4	55±0.8	68±2.0	100
dsRNA 1	4.8	17.6	12.3	24.5	25.2	25.7	17.1	20.8	28.4	71
2	5.7	18.4	13.1	19.8	24.9	18.9	16.4	17.9	25.8	72.4
3	5.8	17.4	12.8	19.6	29.3	20	15.7	18.5	25	72.1
mean	5±0.6	18±0.5	12±0.4	21±2.8	26±2.5	22±3.7	16±0.7	19±1.5	26±1.8	72±0.7

*Supplementary Table 20. % conversion of aminoacylation as determined by PAGE in the reactions of the RNA oligomer **ON5** (0.5 µM, 5'-FAM-rUrArGrGrArGrArCrCrA) in the presence and absence of **15merRNA, ON7** (0.6 µM, 5'-rGrCrArGrUrUrGrGrUrCrUrCrCrUrA) with the specified thioester L-$\mathbf{1}^{e}_{\mathbf{Aaa}}$(200 mM) in MES buffer (400 mM, pH 6.5) and KCl (1 M) after 16 h at rt. Reactions were repeated in triplicate, individual, mean and standard deviation are reported. *100 mM L-$\mathbf{1}^{e}_{\mathbf{Phe}}$. †60 mM L-$\mathbf{1}^{e}_{\mathbf{Arg}}$.*

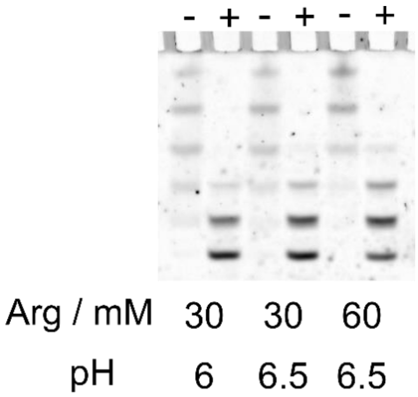

	L-$\mathbf{1}^{e}_{\mathbf{Arg}}$		
	30 mM		60 mM
	pH 6	pH 6.5	pH 6.5
ssRNA	> 95	> 95	> 95
dsRNA	49	56	69

*Supplementary Table 21. % conversion of aminoacylation as determined by PAGE in the reactions of RNA oligomer **ON5** (0.5 µM, 5'-FAM-rUrArGrGrArGrArCrCrA) in the presence (+) and absence (-) of **15merRNA, ON7** (0.6 µM, 5'-rGrCrArGrUrUrGrGrUrCrUrCrCrUrA) with L-arginyl ethanethiol thioester (L-$\mathbf{1}^{e}_{\mathbf{Arg}}$) in MES buffer (200 mM, pH 6.5) and KCl (500 mM) after 16 h at rt. The solution was analysed by general procedure D (ss)/E (ds). Up to +5 aminoacylation is observed before the negative charge of **ON5** is neutralised and the aminoacylated products will not migrate down the gel.*

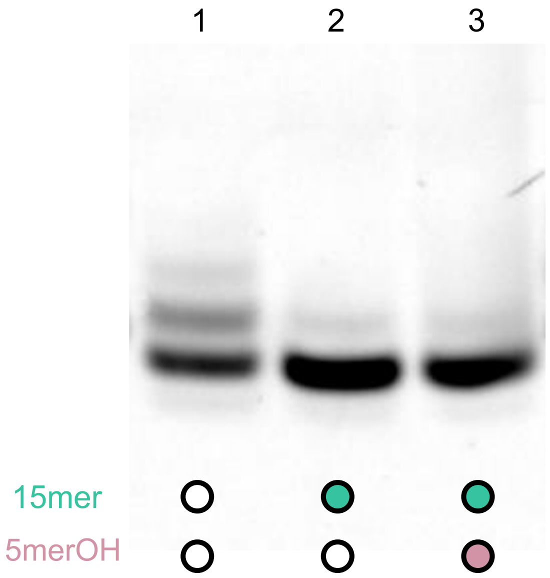

Supplementary Figure 109. PAGE to show the reaction of ***ON5-3'p*** *(0.5 µM, 5'-FAM-rUrArGrGrArGrArCrCrA-3'-p, lanes) with* $L-\mathbf{1}^{e}_{Ala}$ *(200 mM) in the presence or absence of 15mer template* ***15merRNA, ON7*** *(0.6 µM, 5'-rGrCrArGrUrUrGrGrUrCrUrCrCrUrA) and* ***5merOH, ON18*** *(5'-rArCrUrGrC, 0.6 µM, lane 3) in MES buffer (400 mM, pH 6.5) and KCl (1 M) after 16 h at rt. The solution was analysed by general procedure D (ssRNA)/H (dsRNA).*

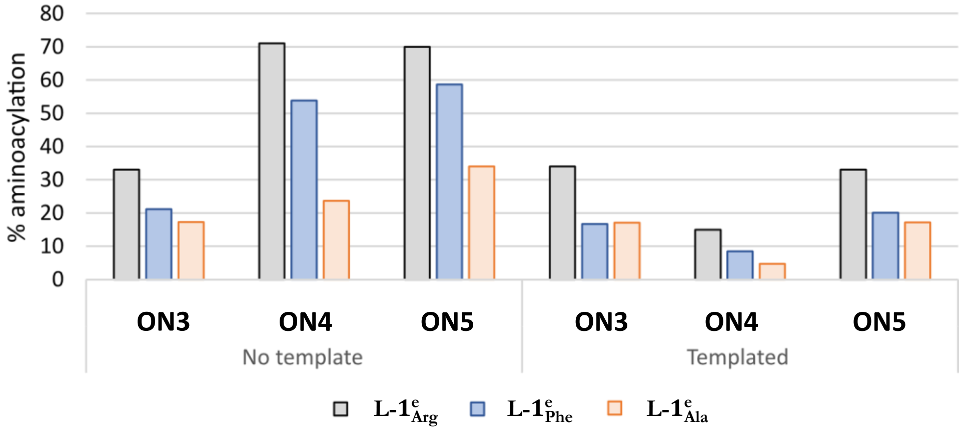

Supplementary Figure 110. % conversions of aminoacylation in the reaction of ***ON3*** *(5'-FAM-dUdAdGdGdAdGdAdCdCrA, 0.5 µM),* ***ON4*** *(5'-FAM-rUrArGrGrArGrArCrCdA, 0.5 µM) or* ***ON5*** *(5'-FAM-rUrArGrGrArGrArCrCrA, 0.5 µM) ± the specified DNA or RNA template (****ON2-3-complement****,* ***ON19*** *or* ***ON4-5-complement****,* ***ON9*** *0.6 µM) with L-phenyl ethanethiol thioester (L-*$\mathbf{1}^{e}_{Phe}$*, 100 mM), L-alanyl ethanethiol thioester (L-*$\mathbf{1}^{e}_{Ala}$*, 100 mM) or L-arginyl ethanethiol thioester (L-*$\mathbf{1}^{e}_{Arg}$*, 25 mM) in MES buffer (200 mM, pH 6.5) and KCl (500 mM) after 16 h at rt. The solution was analysed by general procedure D (no template)/H (templated).*

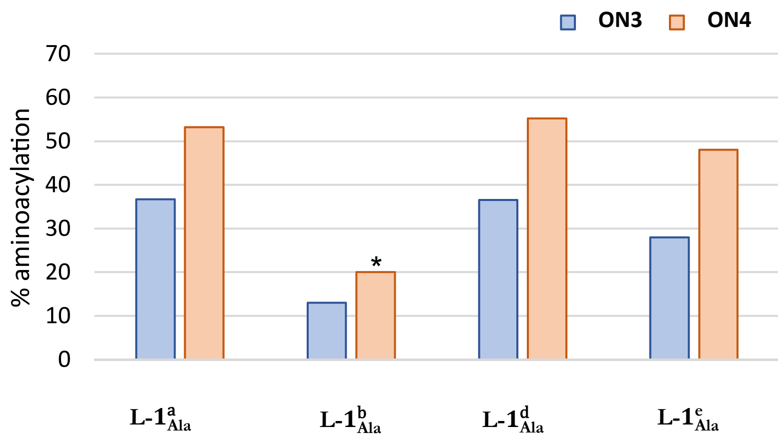

*Supplementary Figure 111. % conversions of aminoacylation in the reaction of **ON3** (5'-FAM-dUdAdGdGdAdGdAdCdCrA, 0.5 µM) or **ON4** (5'-FAM-rUrArGrGrArGrArCrCdA, 0.5 µM) with the specified L-alanyl thioester L-$\mathbf{1}^{n}_{Ala}$ (200 mM; a = (R)-pantetheine, b = 3-mercaptopropionic acid, d = N-Acetyl cysteamine, e = ethanethiol) in MES buffer (200 mM, pH 6.5) and KCl (500 mM) after 16 h at rt. * L-$\mathbf{1}^{b}_{Ala}$ (130 mM). The solution was analysed by general procedure E.*

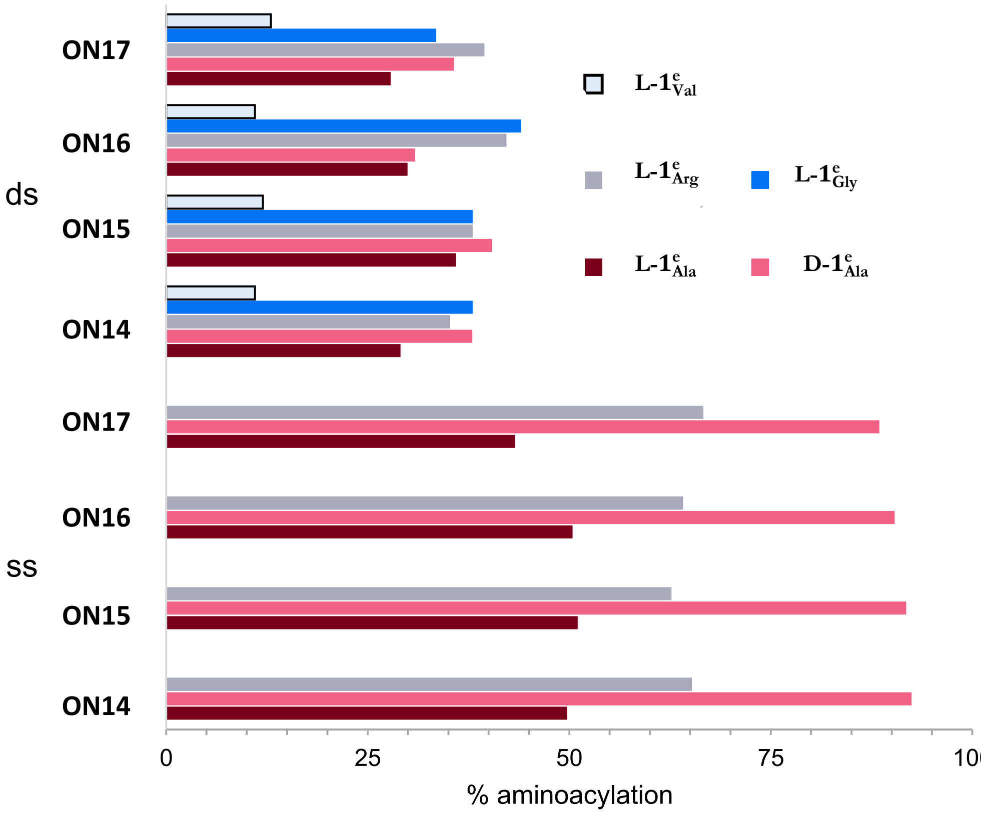

*Supplementary Figure 112. Plot to show % conversion of aminoacylation as determined by PAGE in the reactions of the specified FAM-tagged RNA oligomer **ON14** (5'-FAM-rUrArGrGrArGrArGrCrA,0.5 µM), **ON15** (5'-FAM-rUrArGrGrArGrArGrCrU,0.5 µM), **ON16** (5'-FAM-rUrArGrGrArGrArGrCrC ,0.5 µM), **ON17** (5'-FAM-rUrArGrGrArGrArGrCrG ,0.5 µM) with the specified amino thioester $\mathbf{1}^{e}_{Aaa}$ (200 mM) in MES buffer (200 mM, pH 6.5) and KCl (500 mM) at 23 °C after 16 h in the presence or absence of the specified complementary strand (0.6 µM). The solution was analysed by general procedure D (ss)/E (ds). * 15 mM L-$\mathbf{1}^{e}_{Arg}$.*

	ds					ss		
ON#	L-1^{e}_{Val}	L-1^{e}_{Arg}	1^{e}_{Gly}	L-1^{e}_{Ala}	D-1^{e}_{Ala}	L-1^{e}_{Arg}	L-1^{e}_{Ala}	D-1^{e}_{Ala}
**ON14**	11	35	38	29	38	65	50	92
**ON15**	12	38	38	36	40	63	51	92
**ON16**	11	42	44	30	31	64	50	90
**ON17**	13	39	34	28	36	67	43	88

*Supplementary Table 22. Table to show % conversion of aminoacylation as determined by PAGE in the reactions of the specified FAM-tagged RNA oligomer **ON14** (5'-FAM-rUrArGrGrArGrArGrCrA,0.5 μM), **ON15** (5'-FAM-rUrArGrGrArGrArGrCrU,0.5 μM), **ON16** (5'-FAM-rUrArGrGrArGrArGrCrC ,0.5 μM), **ON17** (5'-FAM-rUrArGrGrArGrArGrCrG ,0.5 μM) with the specified amino thioester $\mathbf{1}^{e}_{Aaa}$ (200 mM) in MES buffer (200 mM, pH 6.5) and KCl (500 mM) at 23 °C after 16 h in the presence or absence of their complementary strand (0.6 μM). * 15 mM L-1^{e}_{Arg}.*

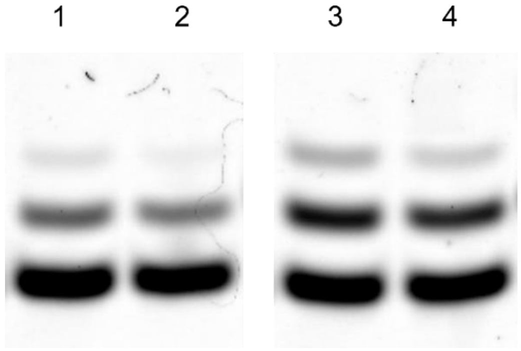

*Supplementary Figure 113. PAGE to show the reaction of **ON3** (0.5 μM, 5'-FAM-dUdAdGdGdAdGdAdCdCrA) with either L-alanyl ethanethiol thioester (L-$\mathbf{1}^{e}_{Ala}$, 200 mM, lanes 1–2) or D-alanyl ethanethiol thioester (D-$\mathbf{1}^{e}_{Ala}$, 200 mM, lanes 3–4) in MES buffer (200 mM, pH 6.5) and KCl (500 mM) after 16 h at rt in the presence (lanes 2, 4) or absence (lanes 1, 3) of complement **ON2-3-complement, ON19** (5'-dUdGdGdUdCdUdCdCdUdA). The solution was analysed by general procedure G (ssDNA)/H (dsDNA).*

Lane	aminoacylation (%)
1	31
2	31
3	45
4	42

*Supplementary Table 23. % conversion of aminoacylation as determined by PAGE in the reactions of oligomer **ON3** (0.5 μM, 5'-FAM-dUdAdGdGdAdGdAdCdCrA) with either L-alanyl ethanethiol thioester (L-$\mathbf{1}^{e}_{Ala}$, 200 mM, lanes 1–2) or D-alanyl ethanethiol thioester (D-$\mathbf{1}^{e}_{Ala}$, 200 mM, lanes 3–4) in MES buffer (200 mM, pH 6.5) and KCl (500 mM) after 16 h at rt in the presence (lanes 2, 4) or absence (lanes 1, 3) of complement **ON2-3-complement, ON19** (5'-dUdGdGdUdCdUdCdCdUdA).*

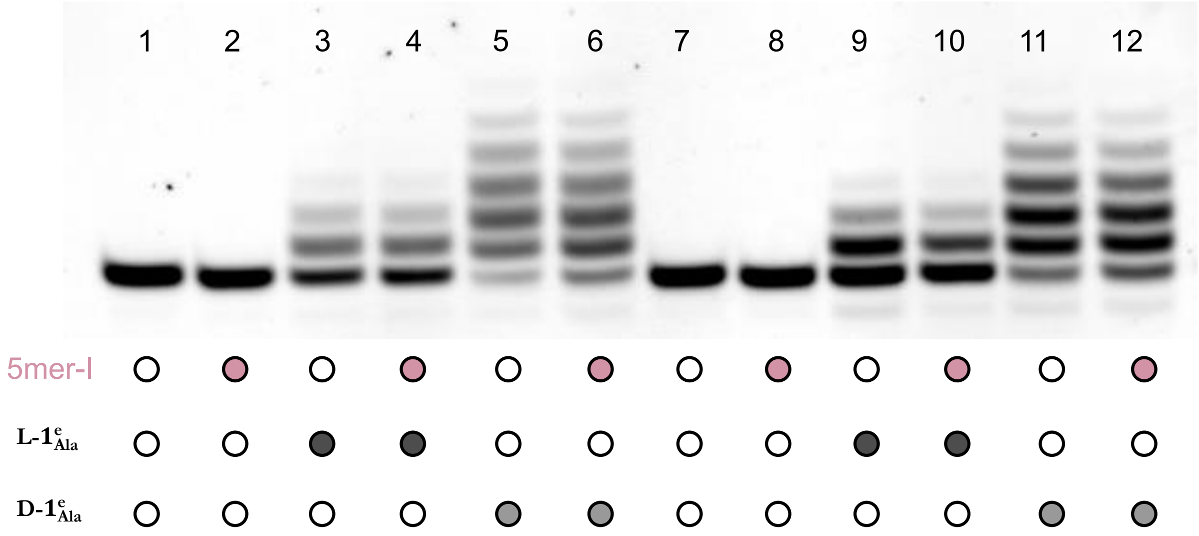

*Supplementary Figure 114. PAGE to show the reaction of either all-RNA oligomer **ON22** (0.5 μM, lanes 1-6, 5'-FAM-rUrCrGrCrUrUrUrCrCrA) or RNA with a 3'-deoxy-C **ON21** (0.5 μM, lanes 7-12, 5'-FAM-rUrCrGrCrUrUrUrCrCdA) with L/D-$\mathbf{1}^{e}_{Ala}$(200 mM) in the presence or absence of **5mer-I, ON23** (5'-phos-rArGrCrGrA) in MES buffer (200 mM, pH 6.5) and KCl (500 mM) after 16 h at rt. The solution was analysed by general procedure G.*

ON22		ON21	
Lane	% aminoacylation	Lane	% aminoacylation
3	56	9	55
4	50	10	59
5	77	11	91
6	81	12	89

Supplementary Table 24. % conversion of aminoacylation as determined by PAGE in the reaction of ***ON21*** *(0.5 μM, 5'-FAM-rUrCrGrCrUrUrUrCrCdA) or* ***ON22*** *(0.5 μM, 5'-FAM-rUrCrGrCrUrUrUrCrCrA) with* L/D-**5^{e}_{Ala}** *((200 mM) in MES buffer (200 mM, pH 6.5) and KCl (500 mM) in the presence or absence of* ***5mer-I, ON23*** *(0.6 μM, 5'-phos-rArGrCrGrA) after 16 h at rt.*

Diastereoselective aminoacylation of nucleosides with thioesters L-$\mathbf{1}_{Ala}$ and/or D-$\mathbf{1}_{Ala}$

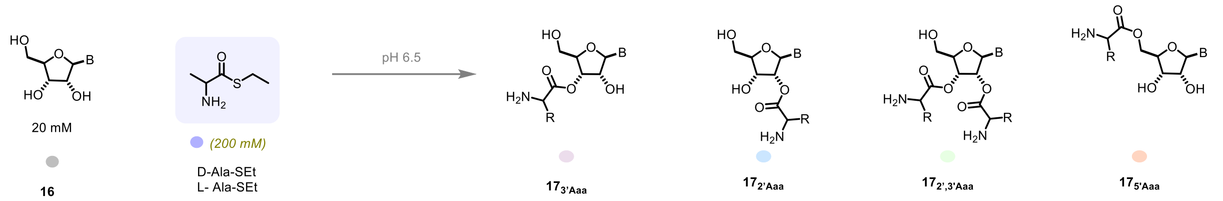

Entry	Nucleophile	Electrophile	$17_{3'Aaa}$ (%)	$17_{2'Aaa}$ (%)	$17_{2',3'Aaa}$ (%)	$17_{5'Aaa}$ (%)	**Total** (%)
1	**16A**	**L-1^{e}_{Ala}**	17	5	2	1	25
2	**16A**	**D-1^{e}_{Ala}**	29	8	4	---	41
3	**16G***	**L-1^{e}_{Ala}**	17	5	3	---	25
4	**16G***	**D-1^{e}_{Ala}**	32	8	8	---	48
5	**16C**	**L-1^{e}_{Ala}**	12	9	3	1	25
6	**16C**	**D-1^{e}_{Ala}**	12	8	3	2	25
7	**16U**	**L-1^{e}_{Ala}**	14	7	3	1	25
8	**16U**	**D-1^{e}_{Ala}**	15	7	5	--	27
9	**16I**	**L-1^{e}_{Ala}**	16	5	3	---	24
10	**16I**	**D-1^{e}_{Ala}**	29	8	6	---	43
11	**21A****	**L-1^{e}_{Ala}**	1	---	---	---	1
12	**21A****	**D-1^{e}_{Ala}**	1	---	---	---	1

*Supplementary Table 25. Yield (%) of aminoacylation in the reaction of the specified nucleoside (20 mM) with either thioester (200 mM) or D-$\mathbf{1}^{e}_{Ala}$ (200 mM) at pH 6.5 after 18 h at room temperature * = **16G** (2 mM) with L-$\mathbf{1}^{e}_{Ala}$ (200 mM) or D-$\mathbf{1}^{e}_{Ala}$ (200 mM) at pH 6.5 after 18 h at room temperature unless otherwise stated; ** = **21A** (2'-deoxyadenosine, 20 mM) with L-$\mathbf{1}^{e}_{Ala}$ (200 mM) or D-$\mathbf{1}^{e}_{Ala}$ (200 mM) at pH 6.5 after 18 h at room temperature. Set up following General Procedure C*

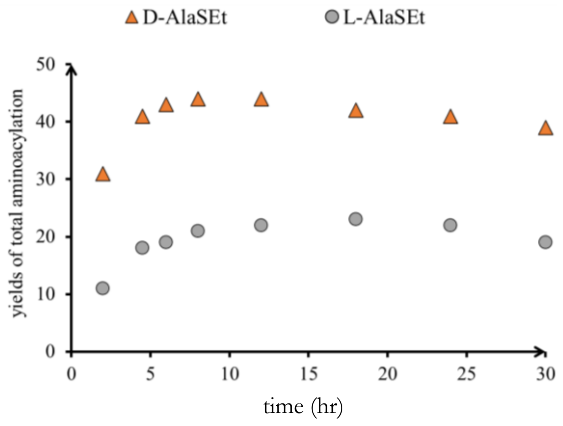

*Supplementary Figure 115. Aminoacylation yield (%), as determined by ^{1}H NMR spectroscopy plotted over time for the reaction of thioester L-$\mathbf{1}^{e}_{Ala}$ (L-AlaSEt, 200 mM) or D-$\mathbf{1}^{e}_{Ala}$ (D-AlaSEt, 200 mM) with adenosine (**16A**, 20 mM) at pH 6.5 and room temperature. hr = hours.*

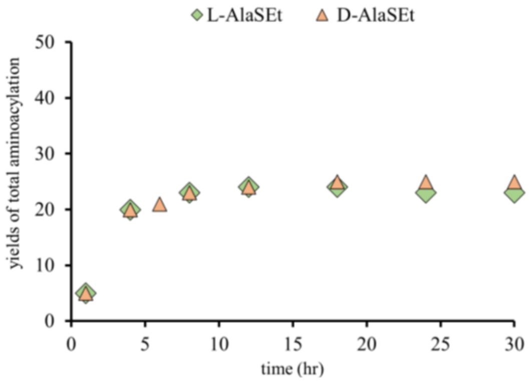

*Supplementary Figure 116. Aminoacylation yield, as determined by ^{1}H NMR spectroscopy plotted over time for the reaction of thioester L-$\mathbf{1}^{e}_{Ala}$ (L-AlaSEt, 200 mM) or D-$\mathbf{1}^{e}_{Ala}$ (D-AlaSEt, 200 mM) with cytidine (**16C**, 20 mM) at pH 6.5 and room temperature. hr = hours.*

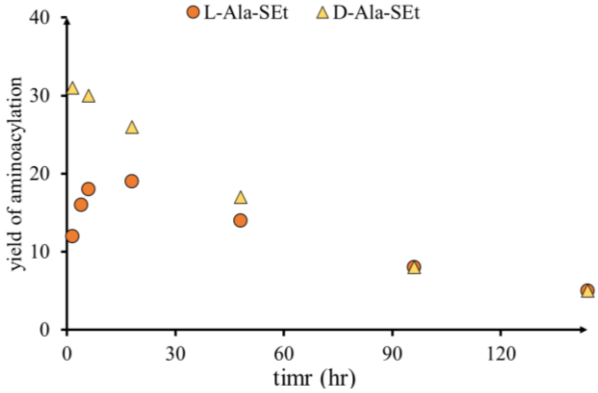

*Supplementary Figure 117. Aminoacylation yield, as determined by ^{1}H NMR spectroscopy plotted over time for the reaction of thioester L-$\mathbf{1}^{e}_{Ala}$ (L-AlaSEt, 200 mM) or D-$\mathbf{1}^{e}_{Ala}$ (D-AlaSEt, 200 mM) with 3',5'-cAMP (**31A,** 20 mM) at pH 6.5 and room temperature. hr = hours.*

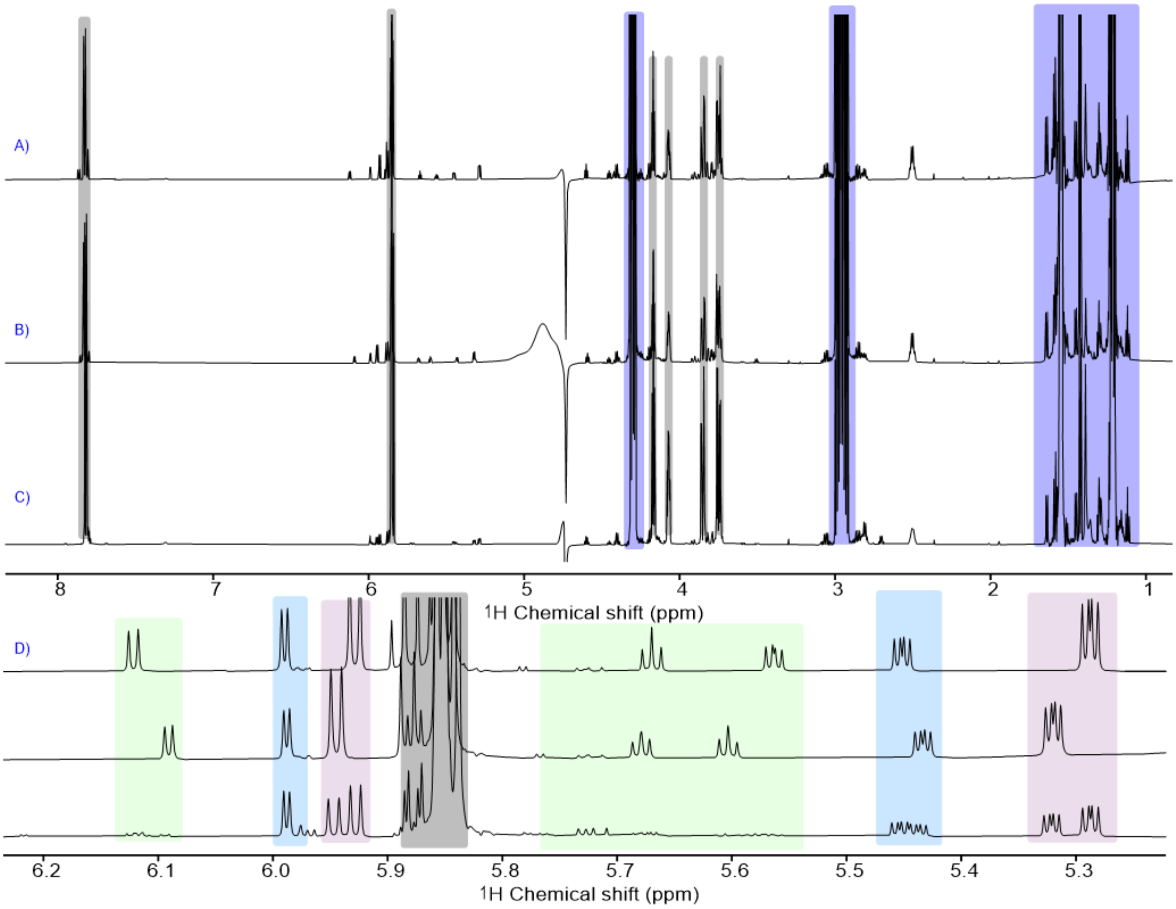

*Supplementary Figure 118: ^{1}H NMR (600 MHz, H_2O/D_2O 9:1, noesygppr1d, 1.0 – 8.0 ppm) spectra to show **A)** the reaction of thioester L-$\mathbf{1^{e}_{Ala}}$ (200 mM) with **16U** (20 mM) at pH 6.5, after 18 h. **B)** Reaction of thioester D-$\mathbf{1^{e}_{Ala}}$ (200 mM) with **16U** (20 mM) at pH 6.5 after 18 h; **C)** Reaction of thioester L-$\mathbf{1^{e}_{Ala}}$ (200 mM) and D-$\mathbf{1^{e}_{Ala}}$ (200 mM) with **16U** (20 mM) at pH 6.5 after 18 h. **D)** Zoom in of spectra **A – C** in the 5.3 – 6.2 ppm region. Set up following General Procedure C.*

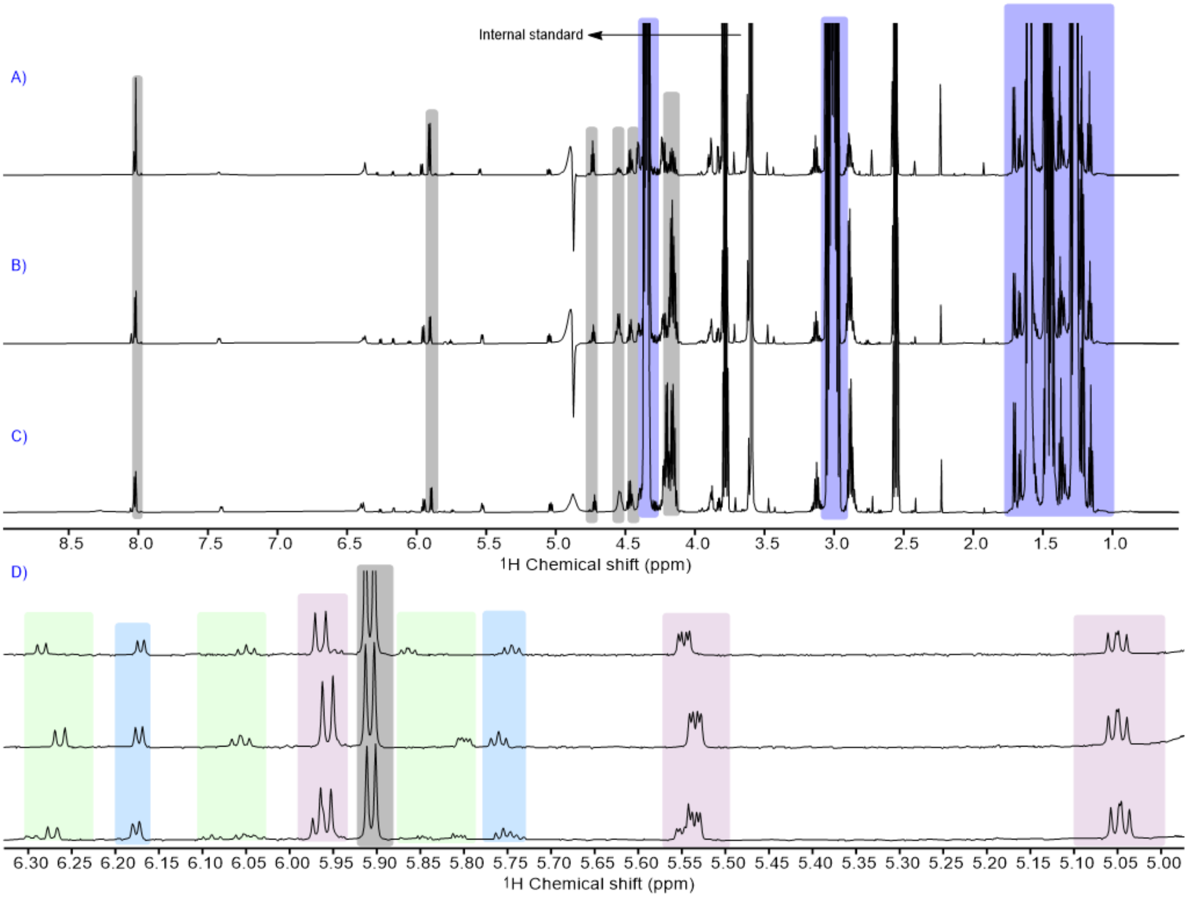

Supplementary Figure 119: ^{1}H NMR (600 MHz, H_2O/D_2O 9:1, noesygppr1d, 0.5 – 9.0 ppm) spectra to show ***A)*** *the reaction of thioester* L-$\boldsymbol{1^{e}_{Ala}}$ *(200 mM) with* ***16G*** *(2 mM) at pH 6.5, using pentaerythritol (10 mM) as an internal standard after 18 h;* ***B)*** *Reaction of thioester* D-$\boldsymbol{1^{e}_{Ala}}$ *(200 mM) with* ***16G*** *(2 mM) at pH 6.5, using pentaerythritol (10 mM) as an internal standard after 18 h;* ***C)*** *Reaction of thioester* L-$\boldsymbol{1^{e}_{Ala}}$ *(200 mM) and* D-$\boldsymbol{1^{e}_{Ala}}$ *(200 mM) with* ***16G*** *(2 mM) at pH 6.5, using pentaerythritol (10 mM) as an internal standard after 18 h. D) Zoom in of spectra* ***A – C*** *in the 5.3 – 6.2 ppm region.*

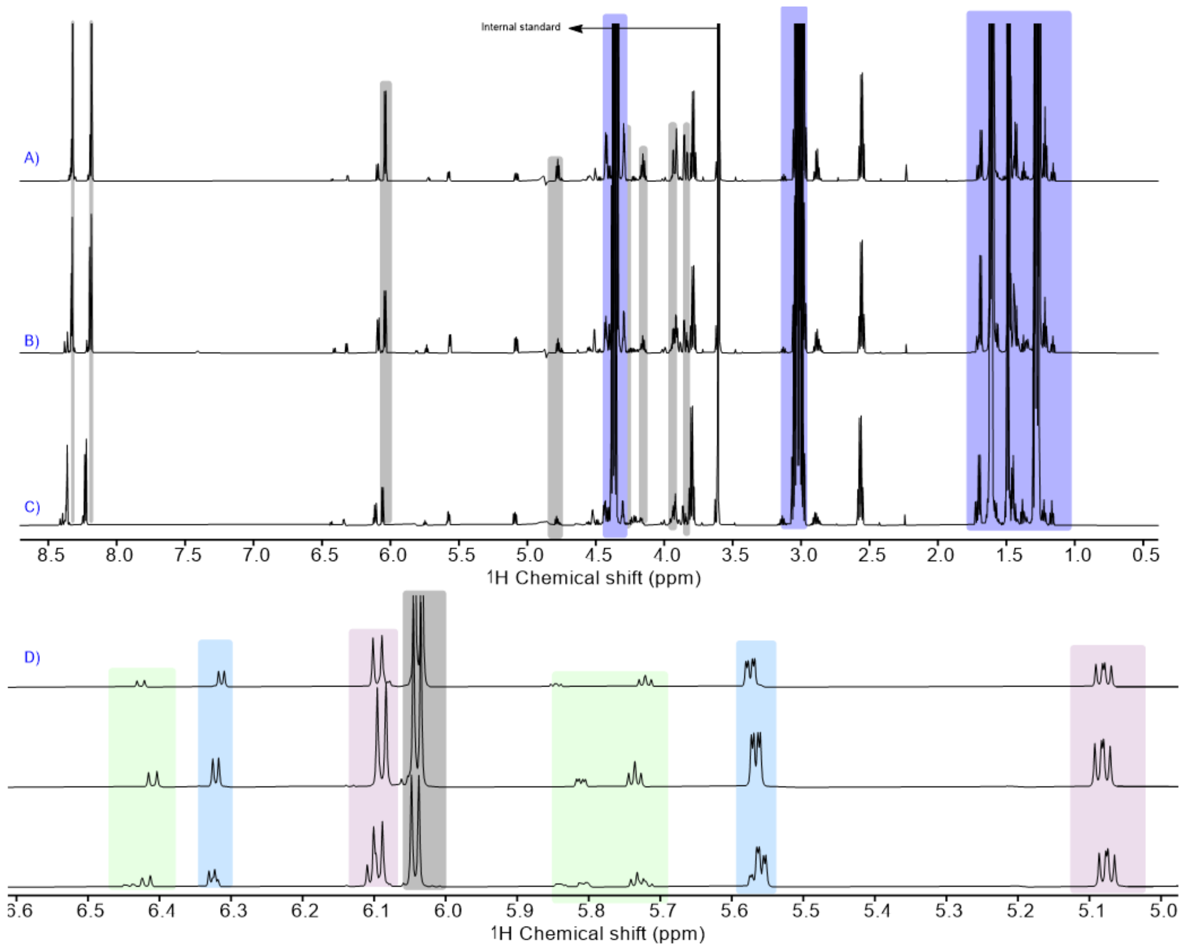

Supplementary Figure 120: ¹H NMR (600 MHz, H_2O/D_2O 9:1, noesygppr1d, 0.5 – 9.0 ppm) spectra to show ***A)*** *the reaction of thioester L-$\mathbf{1^{e}_{Ala}}$ (200 mM) with* ***16A*** *(20 mM) at pH 6.5, using pentaerythritol (10 mM) as an internal standard after 18 hours;* ***B)*** *Reaction of thioester D-$\mathbf{1^{e}_{Ala}}$ (200 mM) with* ***16A*** *(20 mM) at pH 6.5, using pentaerythritol (10 mM) as an internal standard after 18 hour;* ***C)*** *Reaction of thioester L-$\mathbf{1^{e}_{Ala}}$ (200 mM) with* ***16A*** *(20 mM) at pH 6.5, using pentaerythritol (10 mM) as an internal standard after 18 hour; and D-$\mathbf{1^{e}_{Ala}}$ (200 mM) with* ***16A*** *(20 mM) at pH 6.5, using pentaerythritol (10 mM) as an internal standard after 18 hour.* ***D)*** *Zoom in of spectra* ***A – C*** *in the 5.0 – 6.6 ppm region.*

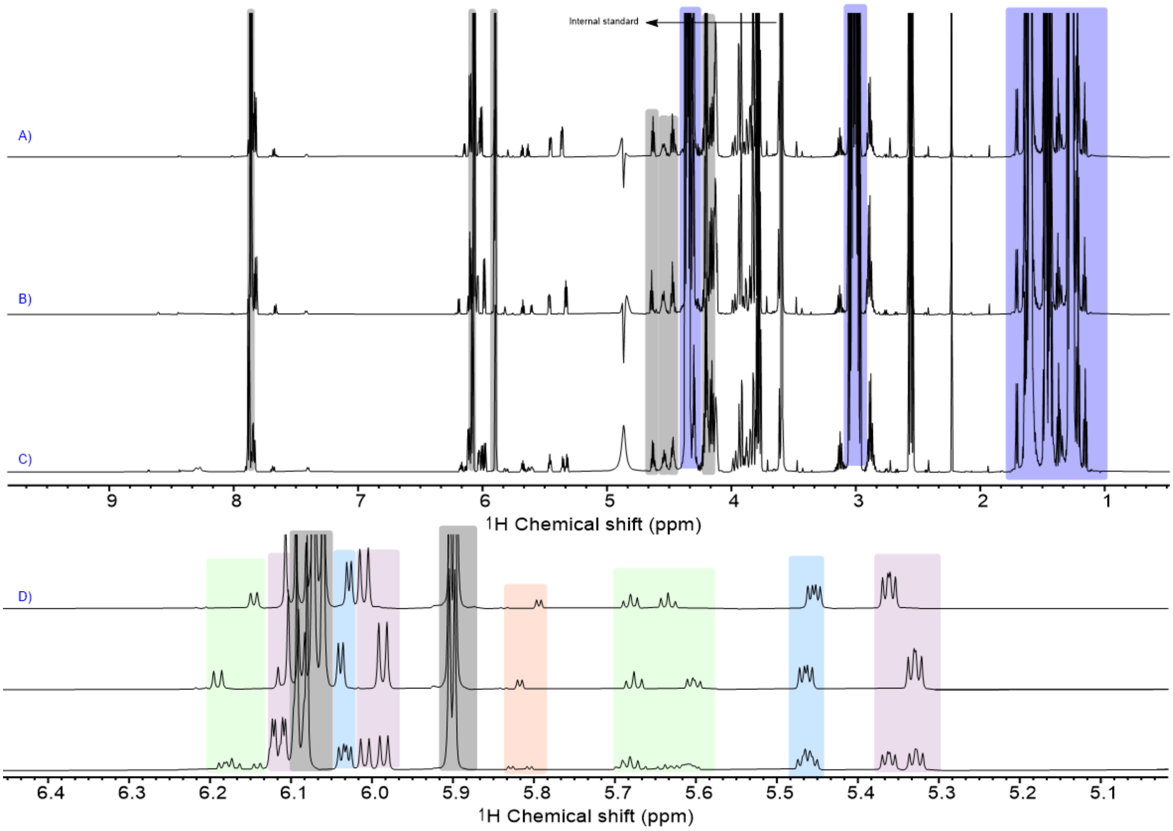

Supplementary Figure 121: ¹H NMR (600 MHz, H_2O/D_2O 9:1, noesygppr1d, 0.5 – 9.0 ppm) spectra to show ***A)*** *the reaction of thioester L-$\mathbf{5^{e}_{Ala}}$ (200 mM) with* ***17C*** *(20 mM) at pH 6.5, using pentaerythritol (10 mM) as an internal standard after 18 h;* ***B)*** *Reaction of thioester* ***D-5^{e}_{Ala}*** *(200 mM) with* ***17C*** *(20 mM) at pH 6.5, using pentaerythritol (10 mM) as an internal standard after 18 h;* ***C)*** *Reaction of thioester L-$\mathbf{5^{e}_{Ala}}$ (200 mM) and* ***D-5^{e}_{Ala}*** *(200 mM) with* ***17C*** *(20 mM) at pH 6.5, using pentaerythritol (10 mM) as an internal standard after 18 h.* ***D)*** *Zoom in of spectra* ***A – C*** *in the 5.1 – 6.4 ppm region.*

ON10 (8.5 µM, 0.59 µL, 5 pmol) and the specified template sequence (**ON11**, **ON12** or **ON13**, 6 pmol) were added to a solution containing 2.5 M KCl and 1 M MES pH 6.5 (2 µL). The specified electrophile was added, and the reaction mixture diluted with H_2O to 10 µL. The final reaction mixture (10 µL) contained 0.5 µM RNA substrate, 0.6 µM template, 200 mM MES pH 6.5, 500 mM KCl and 2–200 mM electrophile. The solution was analysed by general procedure G.

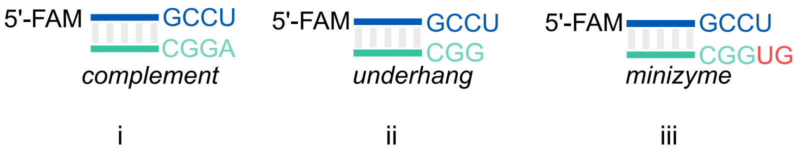

Supplementary Figure 122. Pictorial representation of duplexes formed by ***ON10*** *(5'-FAM-rUrGrArGrArGrArGrCrCrU) with (i) complement:* ***ON11*** *(5'-rArGrGrCrUrCrUrCrUrCrA), (ii) underhang:* ***ON12*** *(5'-GrGrCrUrCrUrCrUrCrA) and (iii) minizyme:* ***ON13*** *(5'-rGrUrGrGrCrUrCrUrCrUrCrA).*

	L-1^{c}_{Ala}		L-1^{c}_{Phe}			L-**Ala-AMP (2$_{Ala}$)**			L-**Phe-AMP (2$_{Phe}$)**		
Template	100 mM	200 mM	1 mM	2 mM	100 mM	1 mM	8 mM	80 mM*	1 mM	10 mM	100 mM*
None	38	50	< 1	< 1	70		< 1	6		19	15
**ON12** **1**	16				30.5			4	< 1	7	10
**2**	17				32.5						
**3**	17				34.3						
**m**	17±1	33	3	< 1	32±2		12	4	< 1	7	10
**ON13** **1**	27.8				62.5						
**2**	26				60.2						
**3**	23.2				54.3						
**m**	26±2	56		10	59±4	4	22	13	15	44	38
**ON11** **1**	18.5				31.5						
**2**	18.3				29.9						
**3**	15.2				26.4						
**m**	17±2	36			29±3		6	< 1		9	< 1

Supplementary Table 26. % conversion of aminoacylation as determined by PAGE in the reactions of RNA oligomer ***ON10*** *(0.5 µM, 5'-FAM-rUrGrArGrArGrArGrCrCrU) with the specified template (0.6 µM) in the presence of the specified electrophile in MES buffer (200 mM, pH 6.5) and KCl (500 mM) after 16 at rt. * 600 mM MES pH 6.5, 1.5 M KCl. Reactions of* ***ON10:ON11, ON10:ON12, ON10:ON13*** *with 100 mM thioester were repeated in triplicate (reported individual value); mean and standard deviation from the mean are reported.*

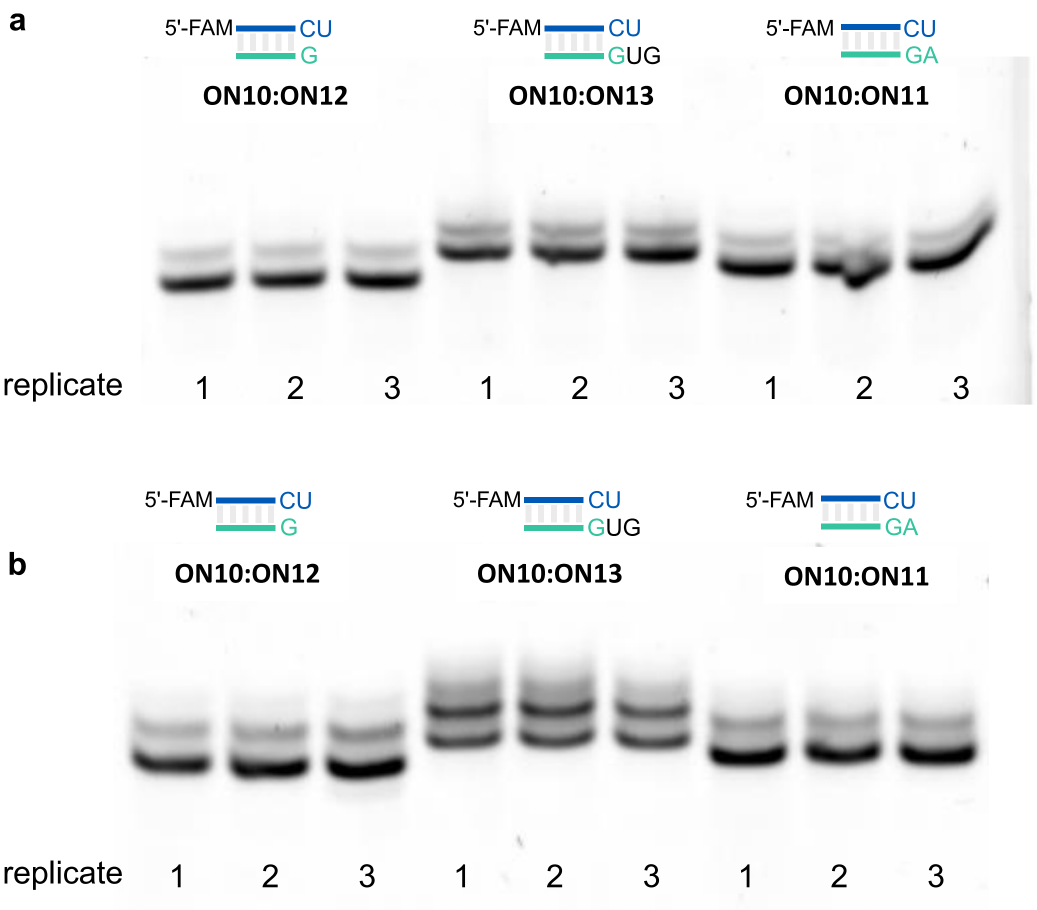

*Supplementary Figure 123. PAGE to show the reaction of underhang **ON10:ON12** (0.5 µM), minizyme **ON10:ON13** (0.5 µM) or complement **ON10:ON11** (0.5 µM) with **a)** alanine thioester L-$\mathbf{1}^{e}_{Ala}$ or **b)** phenylalanine thioester L-$\mathbf{1}^{e}_{Phe}$ in MES buffer (200 mM, pH 6.5) and KCl (500 mM) after 16 h at rt. Each reaction was repeated in triplicate and analysed by General Procedure D.*

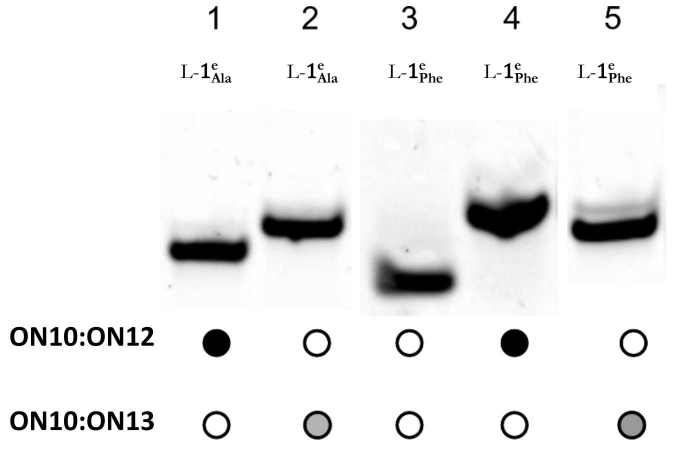

*Supplementary Figure 124. PAGE to show the reaction of underhang **ON10:ON12** (0.5 µM, lanes 1 and 4), minizyme **ON10:ON13** (0.5 µM, lanes 2 and 5) or **ON10** (0.5 µM, lane 3) on its own with either L-$\mathbf{1}^{e}_{Ala}$ (2 mM) or L-$\mathbf{1}^{e}_{Phe}$ (2 mM) in MES buffer (200 mM, pH 6.5) and KCl (500 mM) after 16 h at rt.*

Aminoacylation with other electrophiles

The specified FAM-labelled RNA oligomer (5 pmol) and template (6 pmol) were added to a solution containing 2.5 M KCl and 1 M MES pH 6.5 (2 µL). The reaction mixture was diluted with H_2O to 10 µL and added to a vial containing the specified electrophile. The final reaction mixture (10 µL) contained 0.5 µM RNA substrate, 0.6 µM template, 200 mM MES pH 6.5, 500 mM KCl and 80–200 mM electrophile. The solution was analysed by general procedure G (ssRNA)/H (dsRNA).

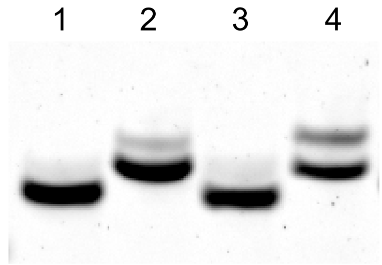

*Supplementary Figure 125. PAGE to show the reaction of **ON10** (0.5 µM, 5'-FAM-rUrGrArGrArGrArGrCrCrU) with L-**Ala-AMP** (80 mM, lanes 1–2) or L-**Phe-AMP** (100 mM, lanes 3–4) in the presence of complement **ON12** (0.6 µM, 5'-GrGrCrUrCrUrCrUrCrA, lanes 1 and 3) or minizyme **ON13** (0.6 µM, 5'-rGrUrGrGrCrUrCrUrCrUrCrA, lanes 2 and 4) in MES buffer (200 mM, pH 6.5) and KCl (500 mM) after 16 h at rt. The solution was analysed by general procedure E.*

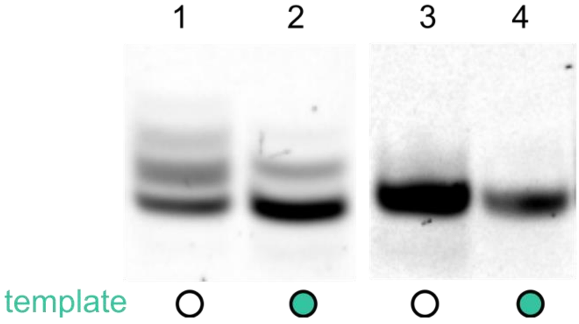

*Supplementary Figure 126. PAGE to show the reaction of **ON5** (0.5 µM, 5'-FAM-rUrArGrGrArGrArCrCrA) in the presence (lanes 2, 4) or absence of template **15merRNA, ON7** (0.6 µM, 5'-rGrCrArGrUrUrGrGrUrCrUrCrCrUrA, lanes 1, 3) with thioester L-$\mathbf{1}^{\mathbf{e}}_{\mathbf{Ala}}$ (200 mM, lanes 1-2) or L-**Ala-AMP** (200 mM, lanes 3-4) in MES buffer (200 mM, pH 6.5) and KCl (500 mM) after 16 h at rt. The solution was analysed by general procedure E. No reactivity was observed for L-**Ala-AMP**. La^{3+}, Zn^{2+} or Mg^{2+} (50 mM) were not found to increase the yield of L-**Ala-AMP** aminoacylation.*

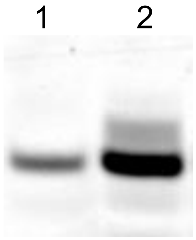

*Supplementary Figure 127. PAGE to show the reaction of **ON5** (0.5 µM, 5'-FAM-rUrArGrGrArGrArCrCrA) with NCA L-$\mathbf{4}_{\mathbf{Ala}}$ (400 mM) in MES buffer (pH 6, 200 mM) and KCl (500 mM) in the absence (lane 1) and presence of 3-mercaptopropionic acid **5b** (200 mM, lane 2). The solution was analysed by general procedure H. Aminoacylation (23%) is observed in the presence of **3b** (lane 2). Significant (~70%) degradation of **ON5** and no aminoacylation was observed in the absence of **3b** (lane 1).*

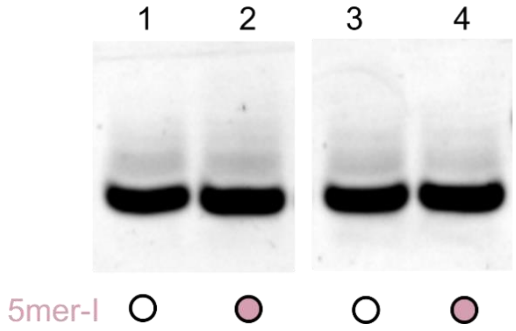

Supplementary Figure 128. PAGE to show the reaction of ***ON22*** *(0.5 µM, 5'-FAM-rUrCrGrCrUrUrUrCrCrA, lanes 1–2) or* ***ON21*** *(0.5 µM, 5'-FAM-rUrCrGrCrUrUrUrCrCdA, lanes 3–4) with L-*$\mathbf{4_{Val}}$ *(140 mM) in MES buffer (200 mM, pH 6.5) and KCl (500 mM) in the presence or absence of template* ***5mer-I, ON23*** *(0.6 µM, 5'-phos-rArGrCrGrA) after 16 h at rt. The solution was analysed by general procedure G. No change in reactivity is observed in the presence or absence of* ***5mer-I, ON23****.*

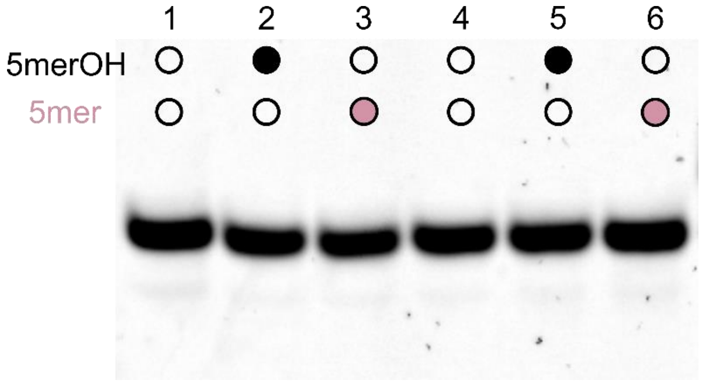

Supplementary Figure 129. PAGE to show the attempted aminoacylation of RNA in a nicked duplex using NCAs. ***ON5*** *(0.5 µM, 5'-FAM-rUrArGrGrArGrArCrCrA) was incubated in the presence of* ***15merRNA, ON7*** *(0.6 µM, 5'-rGrCrArGrUrUrGrGrUrCrUrCrCrUrA) and* ***5merOH, ON18*** *(0.6 µM, 5'-rArCrUrGrC) or* ***5mer, ON8*** *(0.6 µM, 5'-phos-rArCrUrGrC) in MES buffer (400 mM, pH 6.5). D/L-*$\mathbf{33_{Ala}}$ *(200 mM) was added and the reaction mixture analysed by general procedure H after 16 h at rt. Lanes 1-3: L-*$\mathbf{4_{Ala}}$*. Lanes 4-6: D-*$\mathbf{4_{Ala}}$*. No reaction was observed.*

	Metal additive			
**ON#**	No metal	La^{3+}	Zn^{2+}	Mg^{2+}
**ON3**	61	n.d.	n.d.	27
**ON4**	35	7	n.d.	22

Supplementary Table 27. % conversion of aminoacylation as determined by PAGE in the reactions of ***ON3*** *(0.5 µM, 5'-FAM-dUdAdGdGdAdGdAdCdCrA) or* ***ON4*** *(0.5 µM, 5'-FAM-rUrArGrGrArGrArCrCdA) in the presence of the specified metal (50 mM) and L-*$\mathbf{1^{e}_{Ala}}$ *(200 mM) in MES buffer (200 mM, pH 6.5) and KCl (500 mM) after 16 h at rt. The solution was analysed by general procedure D. n.d. = not detected.*

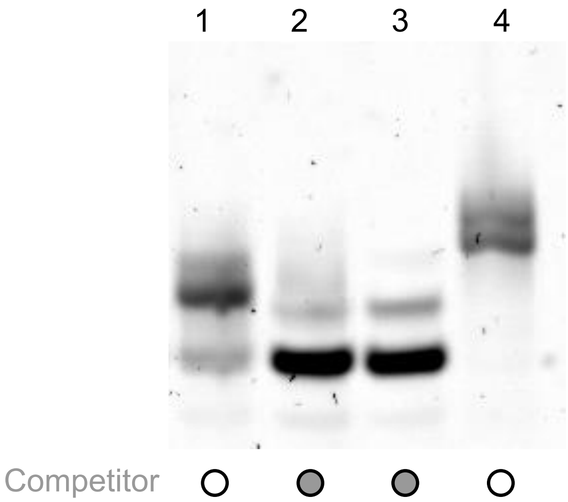

Supplementary Figure 130. PAGE to show the reaction of template ***ON4-5-complement, ON9*** *(0.6 μM, lanes 1-2, 5'-rUrGrGrUrCrUrCrCrUrA) or* ***15merRNA, ON7*** *(0.6 μM, lanes 3-4, 5'-rGrCrArGrUrUrGrGrUrCrUrCrCrUrA) with L-$\mathbf{1}^{c}_{\mathbf{Ala}}$ (200 mM) in MES buffer (200 mM, pH 6.5) and KCl (500 mM) at rt. After 16 h, FAM-tagged oligomer* ***ON5*** *(0.5 μM, 5'-FAM-rUrArGrGrArGrArCrCrA) was added and the reaction mixture left for a further 16 h. Both aminoacylation (25%) and duplex formation (lane 1 and lane 4) are still observed, which can be removed by the addition of a competitor oligomer (lane 2:* ***CompetitorC****; lane 3:* ***CompetitorA****), suggesting that aminoacylation of an RNA strand does not significantly inhibit duplex formation.*

MALDI-MS oligomer aminoacylation data

Reaction mixtures containing **ON5** (0.5 μM), thioester L-$\mathbf{1}^{\mathbf{e}}_{\mathbf{Aaa}}$(200 mM), MES buffer (200 mM, pH 6.5) and KCl (500 mM) were washed with 100 mM pH 4.4 triethylammonium acetate followed by water on a ZipTip® with a C4 resin. The desalted sample was eluted into 9:1 3-hydroxypicolinic acid (50 mg/mL in 1:1 MeCN/water)/dibasic ammonium citrate in (50 mg/mL in water) and 1−2 μL was spotted directly onto a stainless-steel sample plate. The samples were left to dry and analysed on a Shimadzu MALDI-8030 MALDI-TOF instrument (tuning = linear negative; power = 100, pulsed extract = 3500, ion gate blanking = 500).

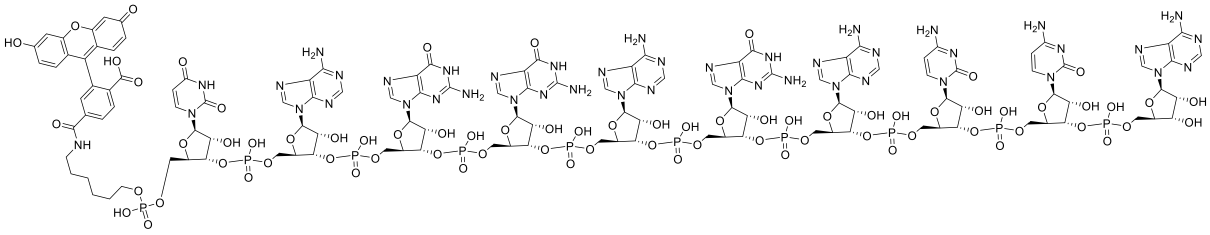

Chemical Formula: $C_{124}H_{144}N_{44}O_{74}P_{10}$
Exact Mass: 3742.62

*Supplementary Figure 131. Structure, chemical formula and exact mass of **ON5** (5'-FAM-rUrArGrGrArGrArCrCrA).*

	+1 aminoacylation			+2 aminoacylation		
Aaa	Formula	Calculated	Observed	Formula	Calculated	Observed
Ala	$C_{127}H_{149}N_{45}O_{75}P_{10}$	3813.7	3814.2	$C_{130}H_{154}N_{46}O_{76}P_{10}$	3884.7	3885.6
Leu	$C_{130}H_{155}N_{45}O_{75}P_{10}$	3855.7	3854.9			
Gly	$C_{126}H_{147}N_{45}O_{75}P_{10}$	3799.6	3799.4	$C_{128}H_{150}N_{46}O_{76}P_{10}$	3856.7	3856.2
Phe	$C_{133}H_{153}N_{45}O_{75}P_{10}$	3889.7	3889.6	$C_{142}H_{162}N_{46}O_{76}P_{10}$	4036.8	4034.8
Pro	$C_{129}H_{151}N_{45}O_{75}P_{10}$	3839.7	3839.4			
Val	$C_{129}H_{153}N_{45}O_{75}P_{10}$	3841.7	3840.6			
Lys	$C_{130}H_{156}N_{46}O_{75}P_{10}$	3870.7	3870.3	$C_{136}H_{168}N_{48}O_{76}P_{10}$	3998.8	3997.9
Ser#	$C_{127}H_{149}N_{45}O_{76}P_{10}$	3829.7	3829.7	$C_{130}H_{154}N_{46}O_{78}P_{10}$	3916.7	3917.5
Glu*	$C_{129}H_{151}N_{45}O_{77}P_{10}$	3871.7	3872.1			

*Supplementary Table 28. MALDI-MS data for the +1 and +2 aminoacylated products following the reaction of **ON5** (0.5 μM, 5'-FAM-rUrArGrGrArGrArCrCrA) with the specified thioester L-$\mathbf{1}^{\mathbf{e}}_{\mathbf{Aaa}}$ (200 mM) in MES buffer (200 mM, pH 6.5) and KCl (500 mM) following purification by ZipTip® with C4 resin. # = DL-Ser *Aminoacylated reaction products of L-$\mathbf{1}^{\mathbf{e}}_{\mathbf{Glu}}$ with **ON5** could not be observed by PAGE due to the addition of Glu to **ON5** not imparting a net change in charge, but Glu-aminoacylated products in the presence and absence of **15merRNA, ON7** (0.6 μM, 5'-rGrCrArGrUrUrGrGrUrCrUrCrCrUrA) could be observed by MALDI-MS.*

	+1*			+2*	
Formula	Calculated	Observed	Formula	Calculated	Observed
$C_{130}H_{156}N_{48}O_{75}P_{10}$	3898.7	3898.4	$C_{136}H_{168}N_{52}O_{76}P_{10}$	4054.8	4054.0
	+3			+4	
Formula	Calculated	Observed	Formula	Calculated	Observed
$C_{142}H_{180}N_{56}O_{77}P_{10}$	4210.9	4207.6	$C_{148}H_{192}N_{60}O_{78}P_{10}$	4367.0	4366.7
	+5			+6	
Formula	Calculated	Observed	Formula	Calculated	Observed
$C_{154}H_{204}N_{64}O_{79}P_{10}$	4523.1	4523.1	$C_{160}H_{216}N_{68}O_{80}P_{10}$	4679.2	4677.8
	+7				
Formula	Calculated	Observed			
$C_{166}H_{228}N_{72}O_{81}P_{10}$	4835.3	4832.6			

*Supplementary Table 29. MALDI-MS data for the aminoacylated species observed in the reaction of **ON5** (0.5 μM, 5'-FAM-rUrArGrGrArGrArCrCrA) with thioester L-$\mathbf{1}^{\mathbf{e}}_{\mathbf{Arg}}$ (60 mM) in MES buffer (200 mM, pH 6.5) and KCl (500 mM) following purification by ZipTip® with C4 resin. *Observed in the presence of template **15merRNA, ON7** (0.6 μM, 5'-rGrCrArGrUrUrGrGrUrCrUrCrCrUrA).*

Formation of thioesters **1** from *N*-carboxyanhydrides **4**

*General procedure I for the formation of thioesters **1** in the reaction of thiols **5** with N-carboxyanhydrides $\mathbf{4_{aa}}$*

Thiol (**5**), 2-(*N*-morpholino)ethanesulfonic acid buffer (MES, 200 mM), and pentaerythritol (PET, 10 mM) were dissolved in degassed H_2O/D_2O (98:2), and the solution was adjusted to the desired pH with NaOH/HCl. This solution was added to freshly prepared α-Amino acid-*N*-carboxyanhydride $\mathbf{4_{Aaa}}$ and the solution was vortexed followed by sonication. The solution was then incubated at room temperature, and NMR spectra were acquired. The structure of α-aminoacyl-thioester $\mathbf{1^{n}_{Aaa}}$ was confirmed by ^{1}H–^{13}C HMBC NMR analysis and by sample spiking with pure synthetic standards where available.

*Formation of thioester $\mathbf{1_{Ala}}$ upon incubation thiols **5** with $\mathbf{4_{Ala}}$ at pH 5.0*

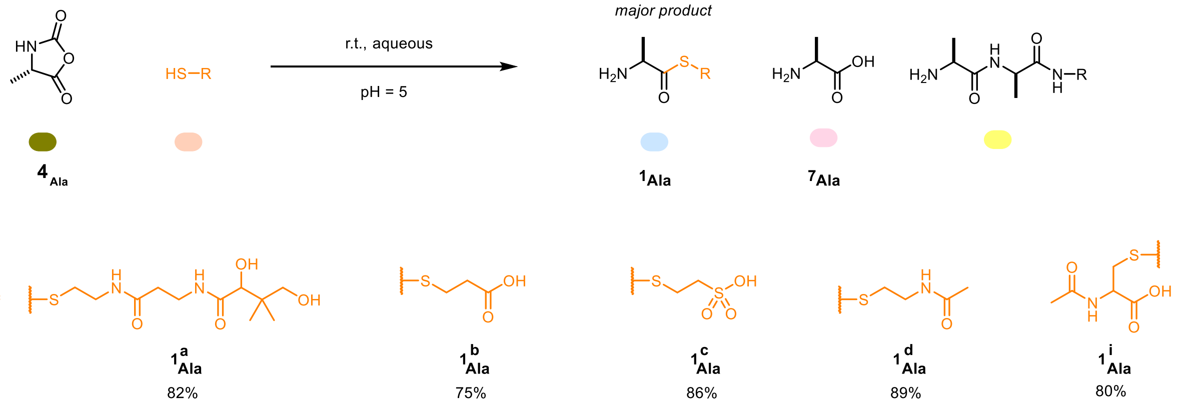

*Supplementary Figure 132: Yields for formation of aminoacyl-thioester $\mathbf{1^{n}_{Ala}}$ from the reaction of α-amino acid-N-carboxyanhydride $\mathbf{4_{Aaa}}$ (20 mM) and thiols **5** (100 mM) with MES buffer (200 mM) with PET (10 mM) as internal standard at pH 5 and room temperature. Set up following General Procedure I.*

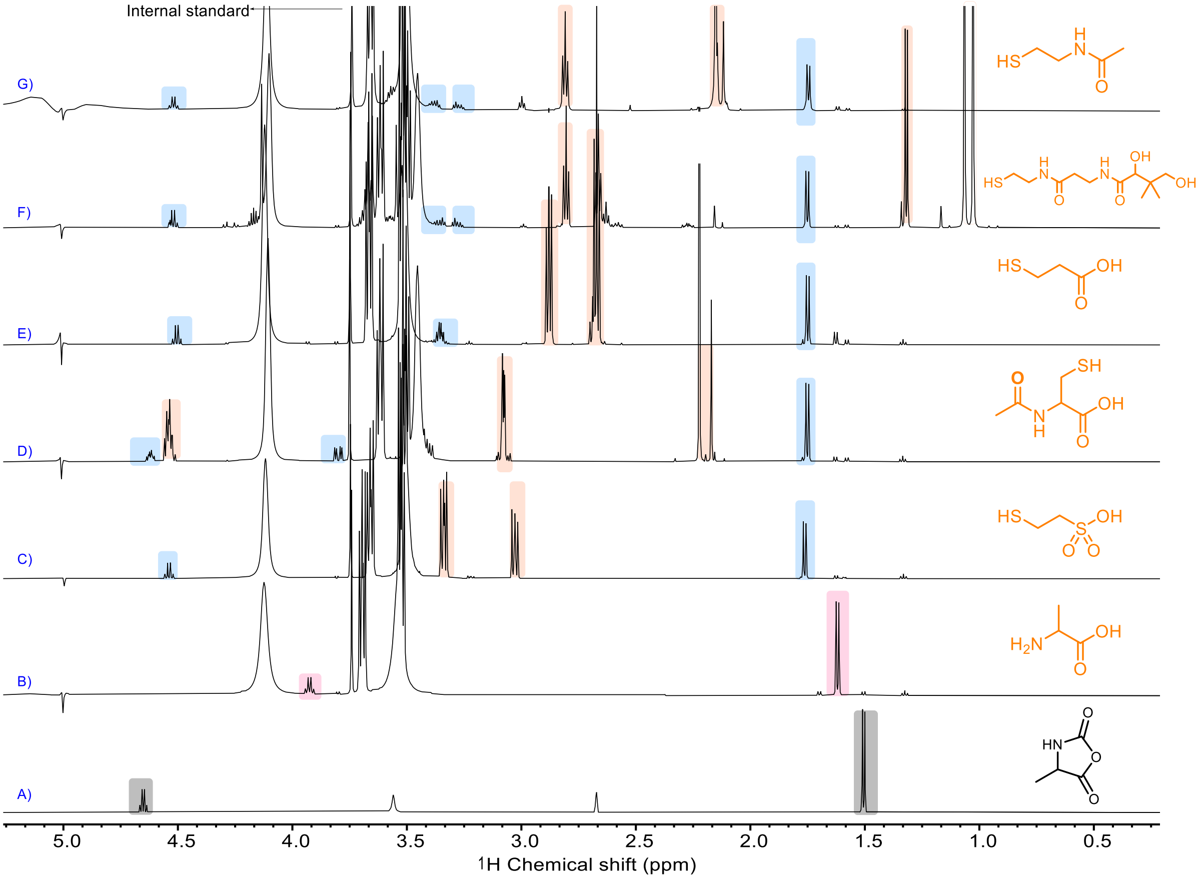

Supplementary Figure 133: ¹H NMR (600 MHz, H_2O/D_2O 9:1 or DMSO-d_6 noesygppr1d, 0.5 – 5.0 ppm) spectra to show: ***A)*** *α-amino acid-N-carboxyanhydride* ***4***$_{\mathbf{Ala}}$ *in DMSO;*[4] ***B)*** ***4***$_{\mathbf{Ala}}$ *in water at pH 6.5 with MES buffer (200 mM) and pentaerythritol as internal standard after 10 minutes;* ***C)*** *the reaction between* ***5c*** *(100 mM) and* ***4***$_{\mathbf{Ala}}$ *(20 mM) with MES buffer (200 mM) at pH 5 and room temperature with PET as internal standard after 10 minutes;* ***D)*** *the reaction between* ***5i*** *(100 mM) and* ***4***$_{\mathbf{Ala}}$ *(20 mM) with MES buffer (200 mM) at pH 5 and room temperature with PET as internal standard after 10 minutes;* ***E)*** *the reaction between* ***5b*** *(100 mM) and* ***4***$_{\mathbf{Ala}}$ *(20 mM) with MES buffer (200 mM) at pH 5 and room temperature with PET*[5] *as internal standard;* ***F)*** *the reaction between* ***5a*** *(100 mM) and* ***4***$_{\mathbf{Ala}}$ *(20 mM) with MES buffer (200 mM) at pH 5 and room temperature with PET as internal standard after 10 minutes;* ***G)*** *the reaction between* ***5d*** *(100 mM) and* ***4***$_{\mathbf{Ala}}$ *(20 mM) with MES buffer (200 mM) at pH 5 and room temperature with PET as internal standard after 10 minutes. Set up following General Procedure I.*

[4] Due to the instability of NCA **4**$_{\mathbf{Ala}}$ in water, the spectrum for the pure starting material is included in DMSO-d_6 for reference. Note that chemical shifts are not directly comparable between spectrum **A** and spectra **B-G** due to the different NMR solvents.

[5] Pentaerythritol (PET) has been used as an internal standard for NMR quantification. PET contains four primary alcohols, and although we did not expect it to aminoacylate due to the high pK_a, we tested this reactivity by incubating PET (10 mM) with amino thioester **1**$_{\mathbf{Ala}}$ and with NCA **4**$_{\mathbf{Ala}}$ but observed no aminoacylation of the alcohol in either case.

Formation of thioester $\mathbf{1^c_{Aaa}}$ *from* $\mathbf{4_{Ala}}$ *with 2-mercaptoethane sulfonate (**5c**) at different pHs and room temperature*

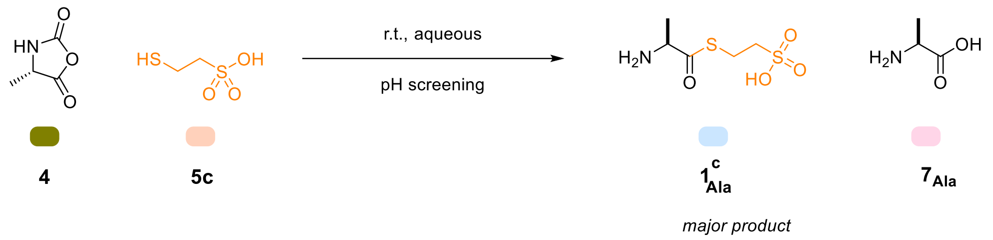

Entry	pH	$\mathbf{1^c_{Ala}}$ (%)	$\mathbf{7_{Ala}}$ (%)
1	3	29	70
2	4	70	27
3	5	81	2
4	6	63	6

Supplementary Table 30. Yield (%) of aminoacyl thiol $\mathbf{1^c_{Ala}}$ *formed from the reaction of* $\mathbf{4_{Ala}}$ *(20 mM) and **5c** (80 mM) in MES buffer (200 mM) at room temperature and the specified pH. Set up following General Procedure I.*

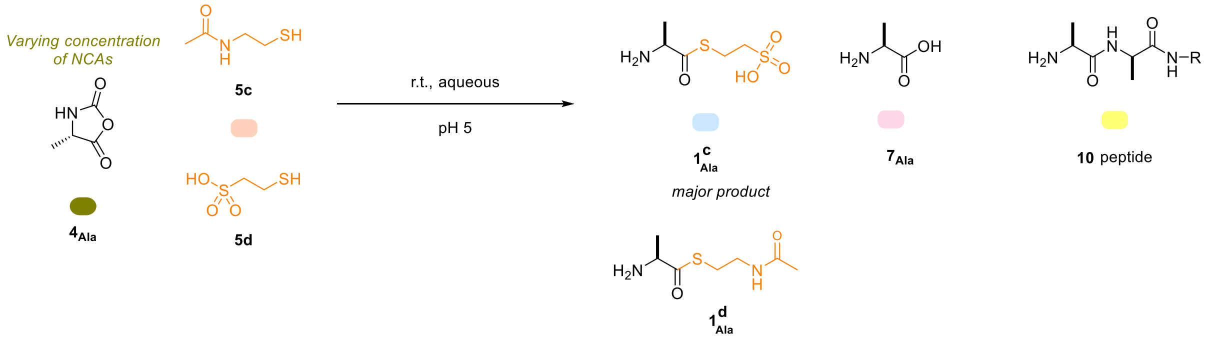

Entry	Thiol	$\mathbf{1_{Ala}}$ (%)	$\mathbf{7_{Ala}}$ (%)	$\mathbf{10_{AlaAla}}$ (%)
1	**5d**	60	4	16
2	**5c**	71	5	13

Supplementary Table 31. Yield (%) of aminoacyl thiol $\mathbf{1_{Ala}}$ *formed from the reaction of* $\mathbf{4_{Ala}}$ *(600 mM) with **5** (1 M), and MES buffer (200 mM) at pH 5 and room temperature. Set up following General Procedure I.*

*Formation of thioester $\mathbf{1^c}_{\mathbf{Aaa}}$ from $\mathbf{4_{Leu}}$ with 2-mercaptoethane sulfonate (**5c**) at different concentrations and room temperature*

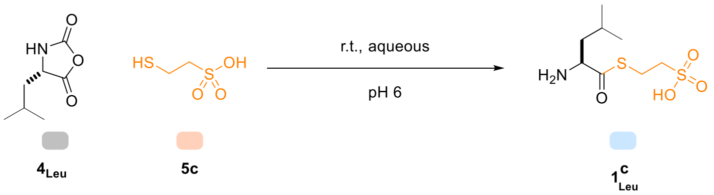

Entry	$\mathbf{4_{Leu}}$ (mM)	**5c** (mM)	$\mathbf{1^c_{Leu}}$ (%)
1	1	1	54
2	1	2	73
3	1	5	88
4	1	10	>95

*Supplementary Table 32: Yield (%) of aminoacyl thiol $\mathbf{1^c_{Leu}}$ formed from the reaction of various dilution concentration of both starting substrate $\mathbf{4_{Leu}}$ and **5c** in Pi buffer (50 mM) at pH 6 and room temperature. Set up following General Procedure I.*

*Thioester **1**^{c}**$_{Aaa}$** formation from the reaction of NCA **4$_{Aaa}$** with 2-mercaptoethanesulfonate **5c***

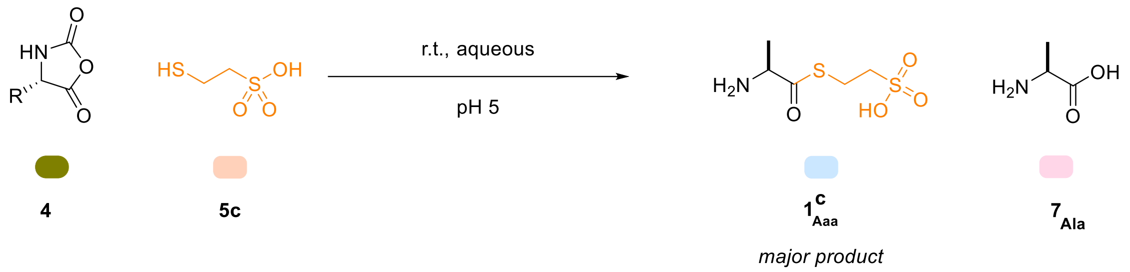

Entry	**4$_{Aaa}$** (%)	**1$^{c}_{Aaa}$** (%)
1	Gly	>95
2	L-Ala	86
3	L-Leu	88
4	L-Phe	>95
5	L-Val	>95
6#	L-Asp	49
7#	L-Glu	82
8	L-Glx	84
9	L-Ser	81

*Supplementary Table 33: Yields (%) of aminoacyl thiol **1**$^{c}_{Aaa}$ formation, from the reaction of NCA **4$_{Aaa}$** (20 mM) with 2-mercaptoethanesulfonate (**5c**, 100 mM) in MES buffer (200 mM) at pH 5 and room temperature. Set up following General Procedure I. Glx = glutamic acid-γ-nitrile. # = MES buffer (200 mM, pH 6).*

*Formation of aminoacyl thiol $\mathbf{1_{Gly}}$ from the reaction of NCA $\mathbf{4_{Gly}}$ with 2-mercaptoethanesulfonate **5c** at pH 5*

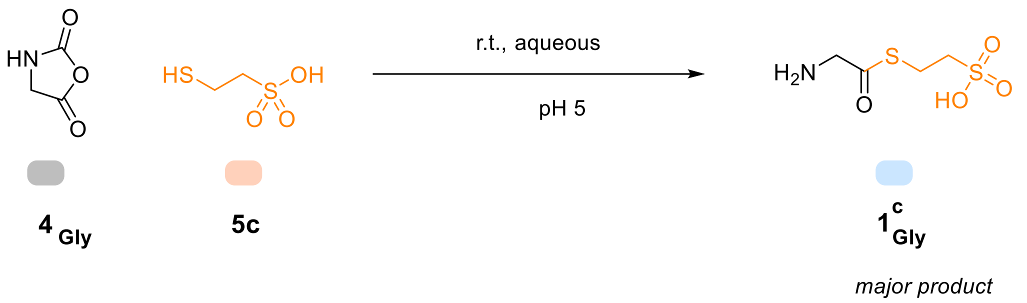

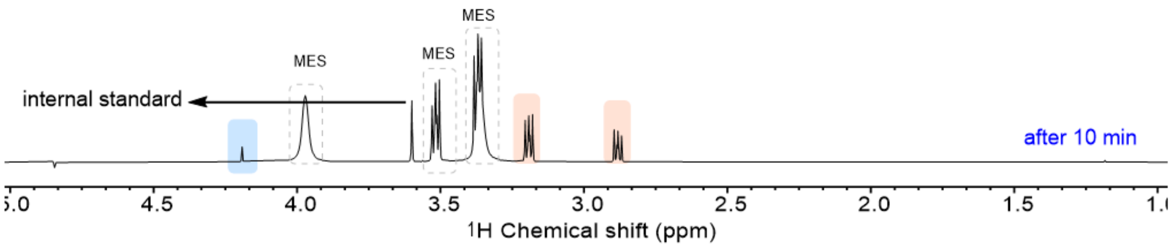

*Supplementary Figure 134: ^{1}H NMR (600 MHz, H_2O/D_2O 9:1, noesygppr1d, 1.0 – 5.0 ppm) spectrum to show the reaction of $\mathbf{4_{Gly}}$ (20 mM) with 2-mercaptoethanesulfonate (**5c**, 100 mM) in MES buffer (200 mM), with PET (10 mM) as an internal standard, at pH 5 and room temperature after 10 min. Set up following General Procedure I.*

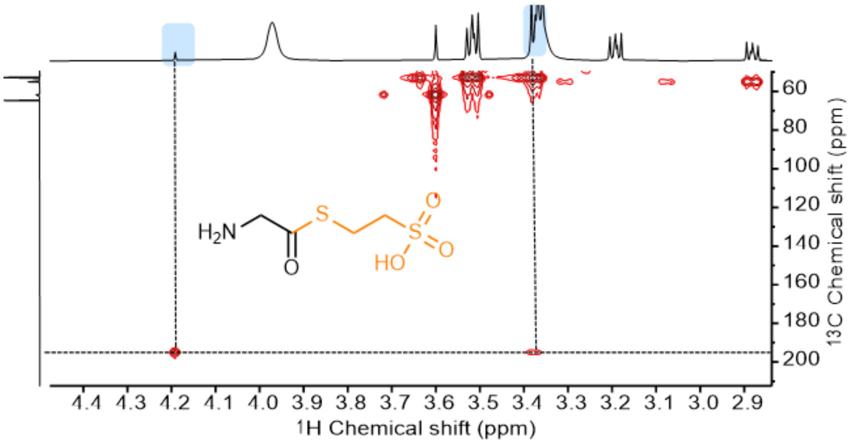

Supplementary Figure 135: ^{1}H–^{13}C HMBC (^{1}H: 600 MHz [2.8 – 4.5 ppm], ^{13}C: 176 MHz [50 – 205 ppm], H_2O/D_2O 9:1) spectrum showing the diagnostic ^{2}JCH and ^{3}JCH coupling of both glycyl α–H and -SCH_2 in $\mathbf{1^c_{Gly}}$ at 4.18 ppm and 3.36 ppm with a resonance at 195.6 ppm, which is characteristic of thioester bond formation.

Formation of aminoacyl thiol ***1***$_{Ala}$ *from the reaction of NCA* ***4***$_{Ala}$ *with 2-mercaptoethanesulfonate* ***5c*** *at pH 5*

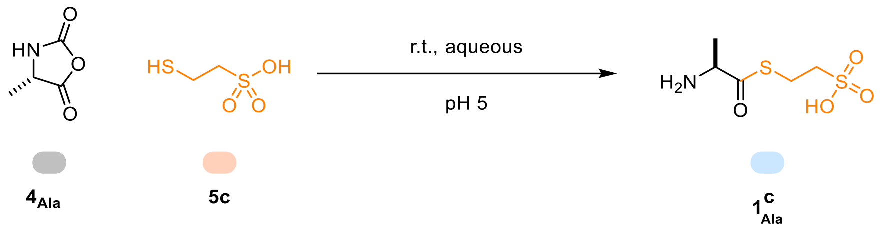

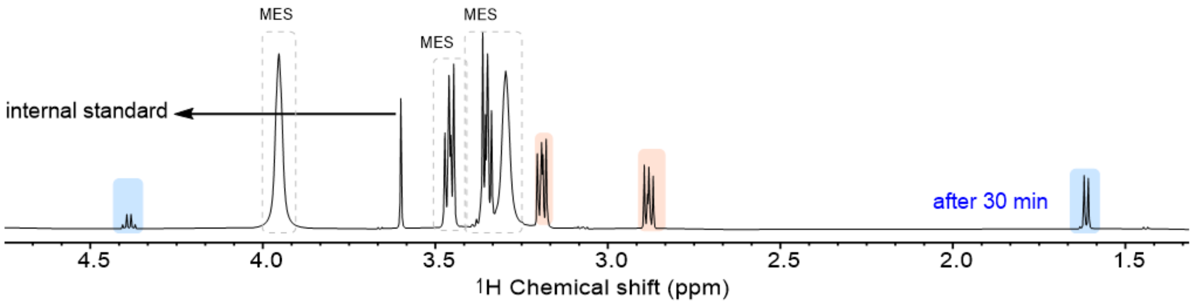

Supplementary Figure 136: ^{1}H NMR (600 MHz, H_2O/D_2O 9:1, noesygppr1d, 1.0 – 5.0 ppm) spectrum to show the reaction of ***4***$_{Ala}$ *(20 mM) with 2-mercaptoethanesulfonate (****5c****, 100 mM) in MES buffer (200 mM), with PET (10 mM) as an internal standard at pH 5 after 30 min at room temperature. Set up following General Procedure I.*

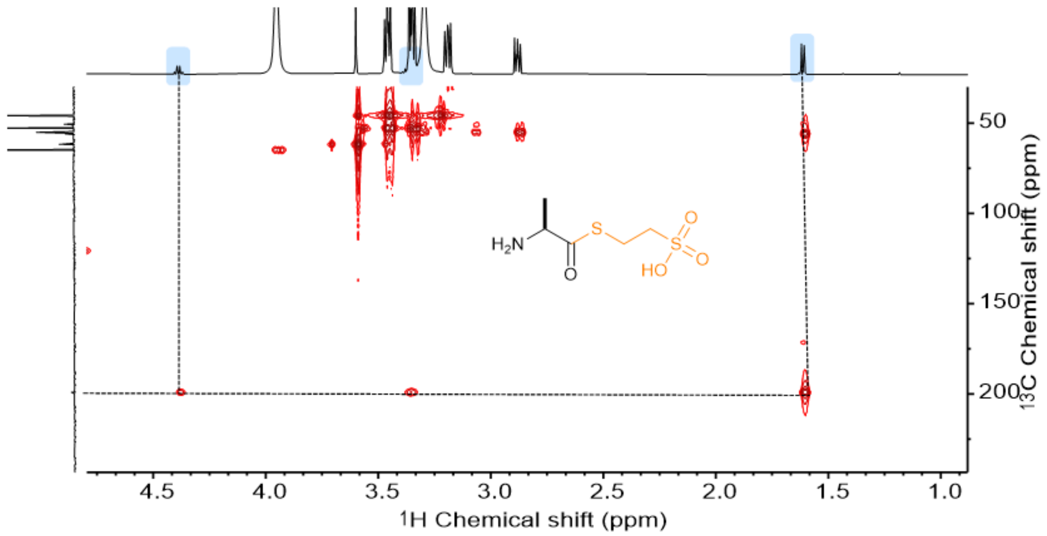

Supplementary Figure 137: ^{1}H–^{13}C HMBC ([1]H: 600 MHz [1.0 – 5.0 ppm], [13]C: 176 MHz [50 – 220 ppm], H_2O/D_2O 9:1) spectrum showing the diagnostic ^2JCH and ^3JCH coupling of Alanyl-αH and -SCH_2 in ***1***$^c_{Ala}$ *at 4.37 ppm and 3.35 ppm with a resonance at 199.4 ppm, which is characteristic of thioester bond formation.*

*Formation of aminoacyl thiol **1**$_{Val}$ from the reaction of NCA **4**$_{Val}$ with 2-mercaptoethanesulfonate **5c** at pH 5*

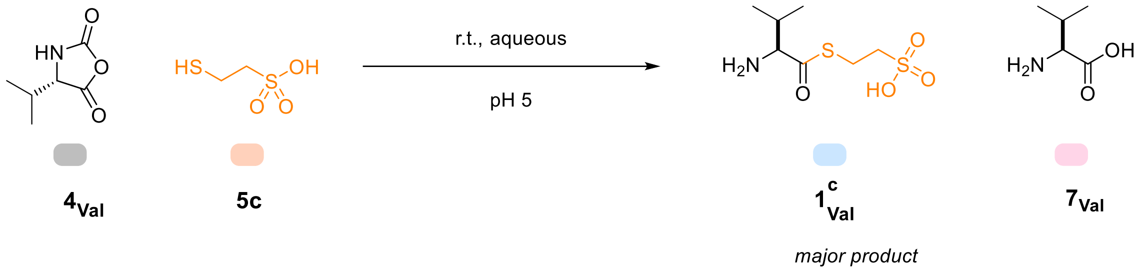

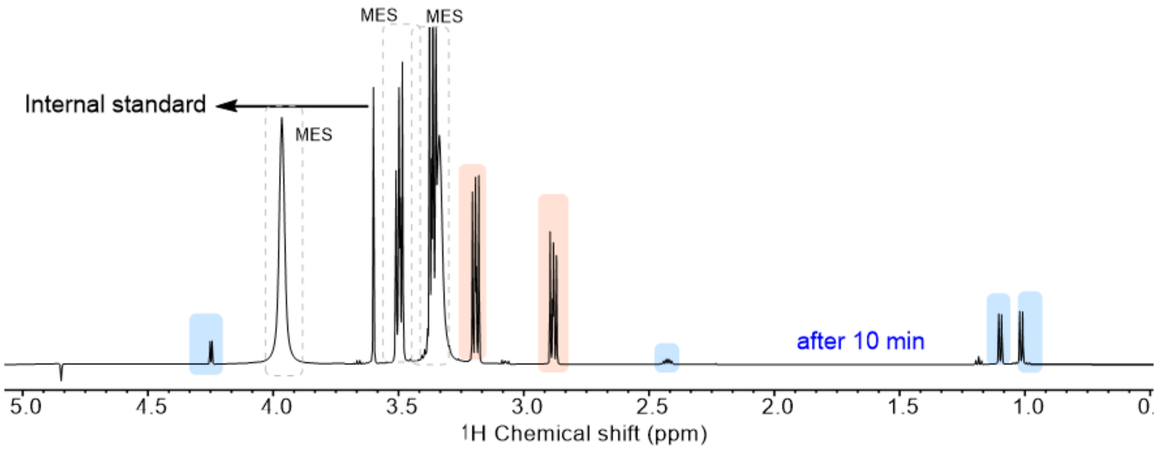

*Supplementary Figure 138: ^{1}H NMR (600 MHz, H_2O/D_2O 9:1, noesygppr1d, 0 – 5.0 ppm) spectrum to show the reaction of **4**$_{Val}$ (20 mM) with 2-mercaptoethanesulfonate (**5c**, 100 mM) in MES buffer (200 mM), with PET (10 mM) as an internal standard at pH 5 and room temperature after 10 min. Set up following General Procedure E.*

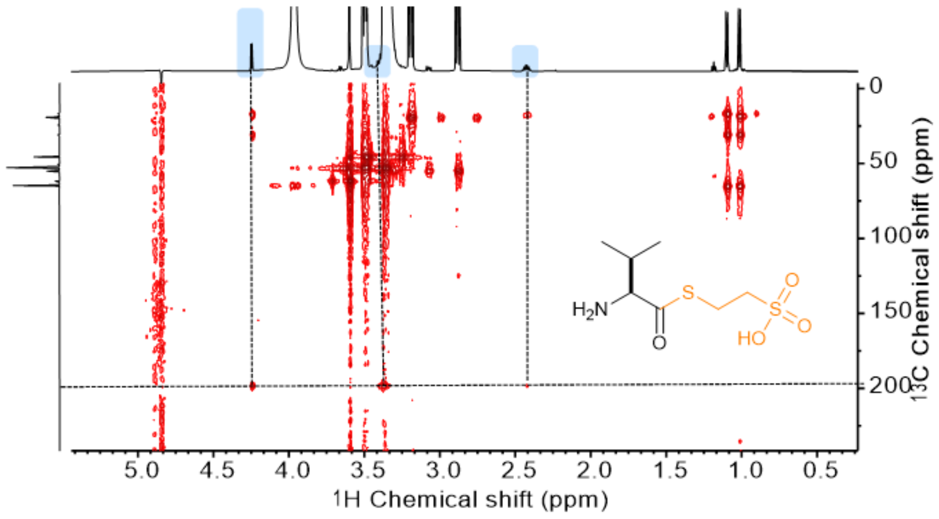

*Supplementary Figure 139: ^{1}H–^{13}C HMBC (^{1}H: 600 MHz [0.5 – 5.0 ppm], ^{13}C: 176 MHz [0 – 200 ppm], H_2O/D_2O 9:1) spectrum showing the diagnostic ^{2}JCH and ^{3}JCH coupling of valinyl-αH and -SCH$_2$ in **1**$^C_{Val}$ at 4.26 ppm, 3.36 ppm with a resonance at 198.8 ppm, which is characteristic of thioester bond formation.*

Formation of aminoacyl thiol 1_{Leu} from the reaction of NCA 4_{Leu} with 2-mercaptoethanesulfonate ***5c*** *at pH 5*

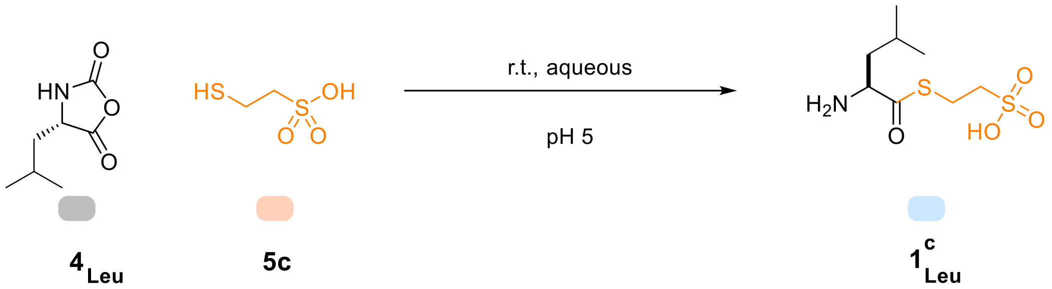

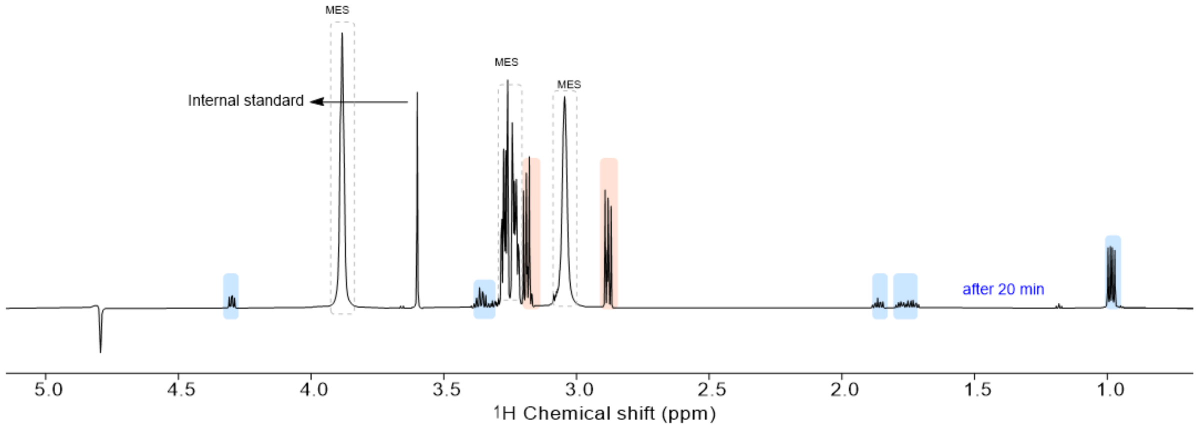

Supplementary Figure 140: ¹H NMR (600 MHz, H_2O/D_2O 9:1, noesygppr1d, 0 – 5.0 ppm) spectrum to show the reaction of $\mathbf{4_{Leu}}$ *(20 mM) with 2-mercaptoethanesulfonate (**5c**, 100 mM) in MES buffer (200 mM), with PET (10 mM) as an internal standard at pH 5 and room temperature after 20 min. Set up following General Procedure I.*

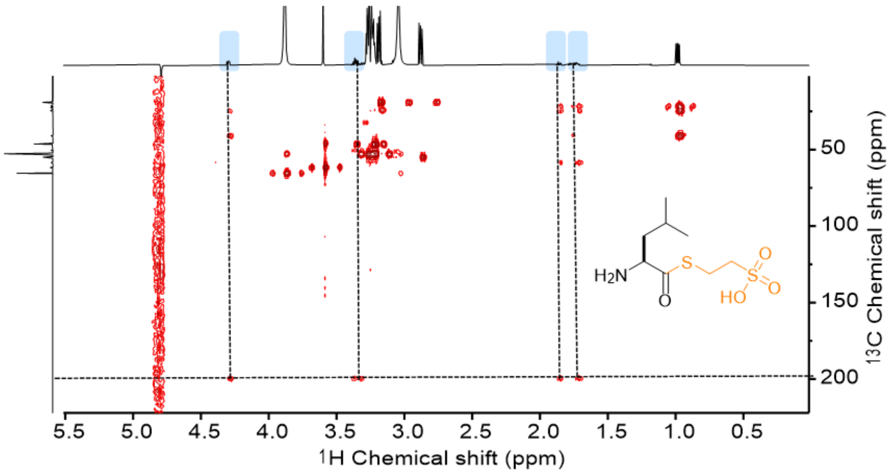

Supplementary Figure 141: ¹H–¹³C HMBC (¹H: 600 MHz [0.5 – 5.5 ppm], ¹³C: 176 MHz [163 – 207 ppm], H_2O/D_2O 9:1) spectrum showing the diagnostic ^2JCH and ^3JCH coupling of leucinyl-αH and -SCH_2 in $\mathbf{1^C_{Leu}}$ *at 4.28 ppm and 3.34 ppm with a resonance at 199.5 ppm, which is characteristic of thioester bond formation.*

*Formation of aminoacyl thiol **1**$_{Ser}$ from the reaction of NCA **4**$_{ser}$ with 2-mercaptoethanesulfonate **5c** at pH 5*

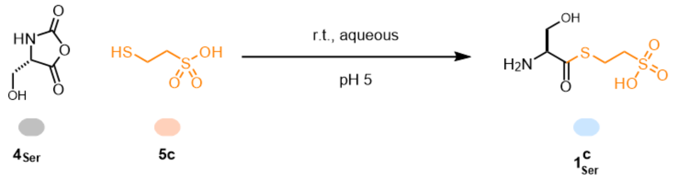

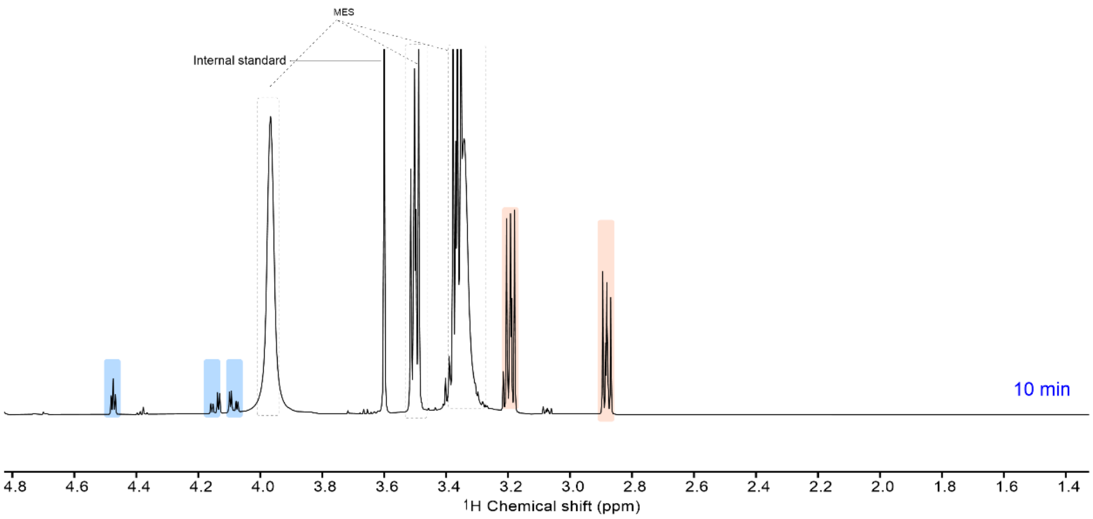

*Supplementary Figure 142: ^{1}H NMR (600 MHz, H_2O/D_2O 9:1, noesygppr1d, 1.5 – 7.5 ppm) spectrum to show the reaction of **4**$_{Ser}$ (20 mM) with 2-mercaptoethanesulfonate (**5c**, 100 mM) in MES buffer (200 mM), with PET (10 mM) as an internal standard at pH 5 and room temperature after 10 min. Set up following General Procedure I.*

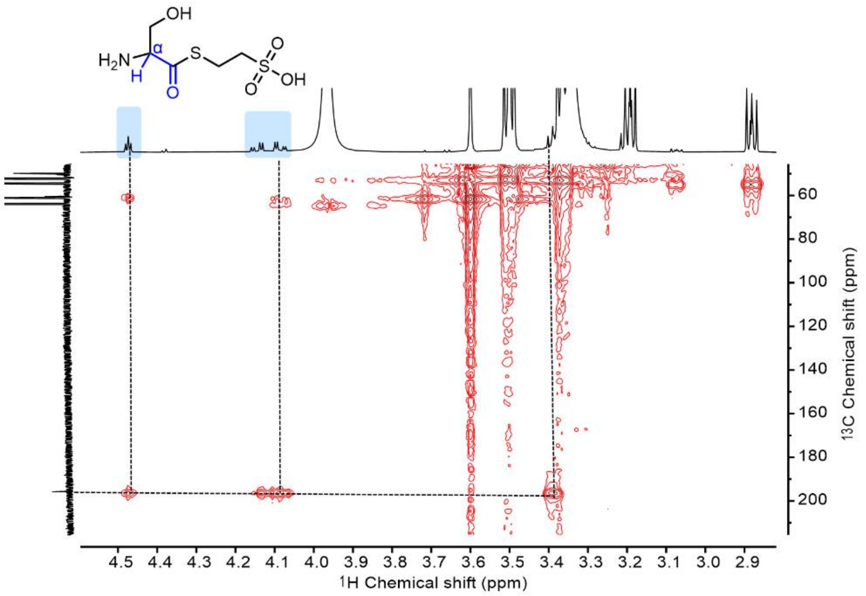

*Supplementary Figure 143: ^{1}H–^{13}C HMBC (^{1}H: 600 MHz [0.5 – 5.0 ppm], ^{13}C: 176 MHz [60 – 200 ppm], H_2O/D_2O 9:1) spectrum showing the diagnostic ^{2}JCH and ^{3}JCH coupling of serniyl-αH and -SCH_2 in **1**$^{C}_{Ser}$ at 4.47 ppm, 3.39 ppm with a resonance at 196.3 ppm, which is characteristic of thioester bond formation.*

Formation of aminoacyl thiol $\mathbf{1_{Phe}}$ from the reaction of NCA $\mathbf{4_{Phe}}$ with 2-mercaptoethanesulfonate ***5c*** *at pH 5*

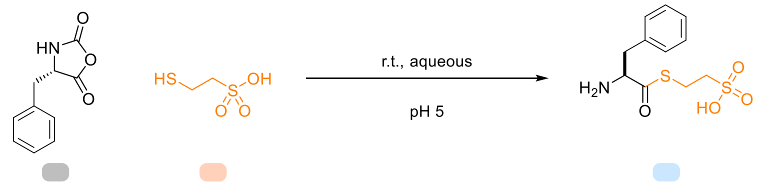

$\mathbf{4_{Phe}}$ **5c** $\mathbf{5^{c}_{Phe}}$

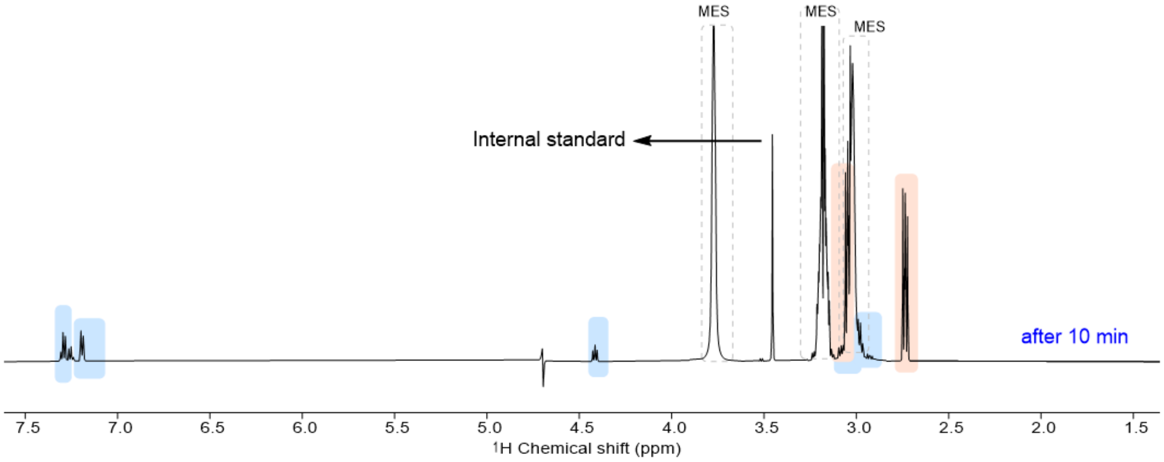

Supplementary Figure 144: ^{1}H NMR (600 MHz, H_2O/D_2O 9:1, noesygppr1d, 1.5 – 7.5 ppm) spectrum to show the reaction of $\mathbf{4_{Phe}}$ *(20 mM) with 2-mercaptoethanesulfonate (**5c**, 100 mM) in MES buffer (200 mM), with PET (10 mM) as an internal standard at pH 5 and room temperature after 10 min. Set up following General Procedure I.*

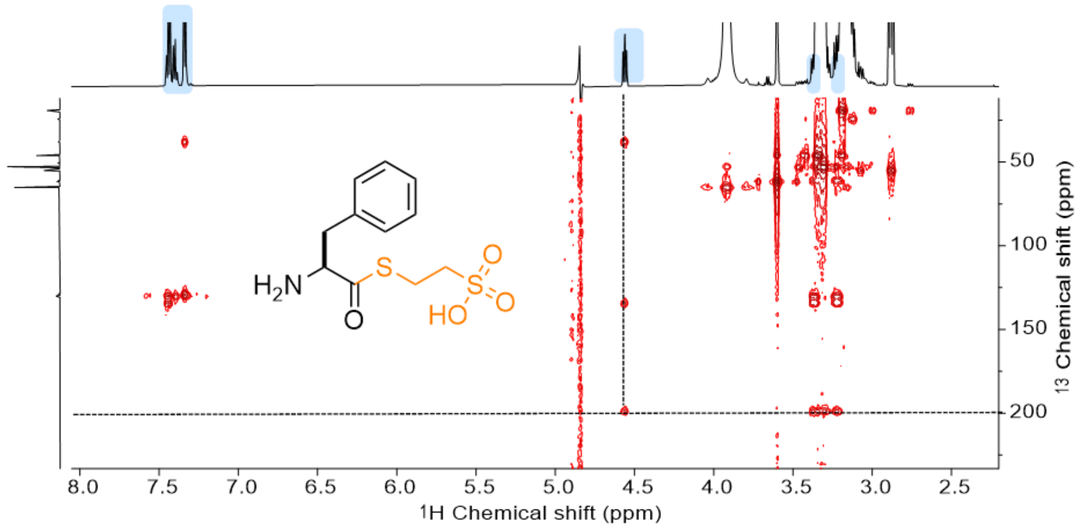

Supplementary Figure 145: ^{1}H–^{13}C HMBC (^{1}H: 600 MHz [2.0-8.0 ppm], ^{13}C: 176 MHz [50-200 ppm], H_2O/D_2O 9:1) spectrum showing the diagnostic $^{2}J_{CH}$ and $^{3}J_{CH}$ coupling of phenylalaninyl-αH and -SCH_2 in $\mathbf{1^{c}_{Phe}}$ *at 4.56 ppm, 3.36 ppm with a resonance at 199.5 ppm, which is characteristic of thioester bond formation.*

Formation of aminoacyl thiol $\mathbf{1_{Glu}}$ *from the reaction of NCA* $\mathbf{4_{Glu}}$ *with 2-mercaptoethanesulfonate* ***5c*** *at pH 5*

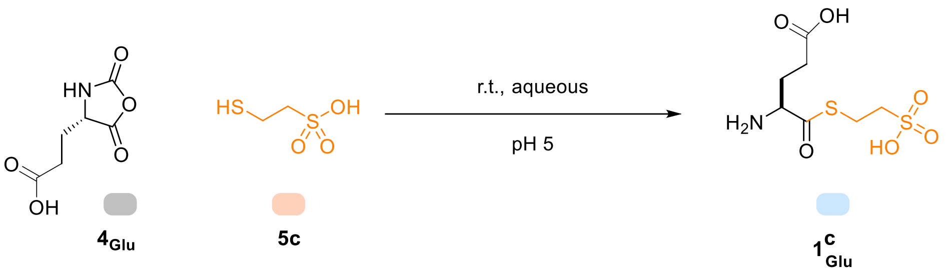

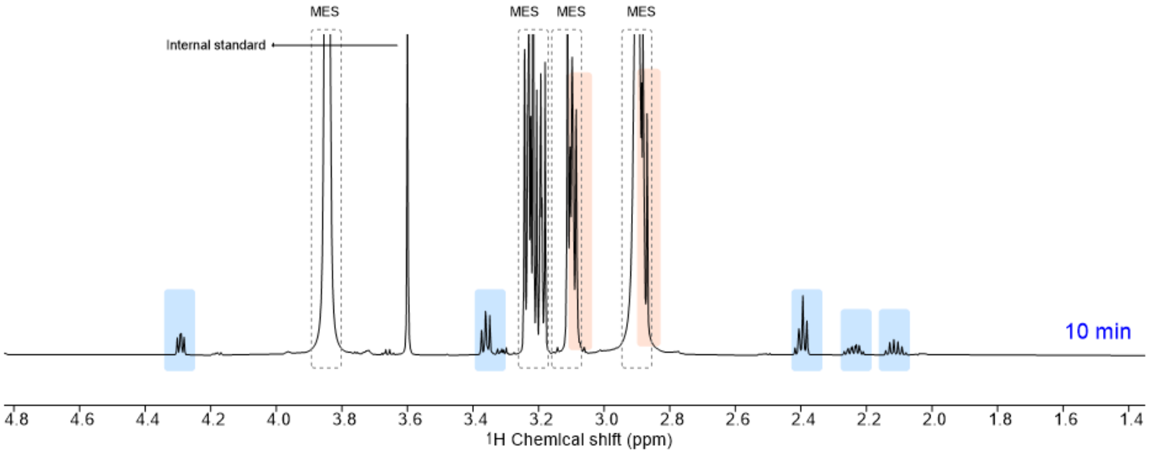

Supplementary Figure 146: ¹H NMR (600 MHz, H_2O/D_2O 9:1, noesygppr1d, 1.4 – 5.0 ppm) spectra to show the reaction of $\mathbf{4_{Glu}}$ *(20 mM) with 2-mercaptoethanesulfonate (**5c**, 100 mM) and MES buffer (200 mM), with PET (10 mM) as an internal standard at pH 6 after 10 min and room temperature. Set up following General Procedure I.*

¹H NMR (600 MHz, H_2O/D_2O 9:1) $\mathbf{1^{c}_{Glu}}$ (partial assignment) : δ_H 4.29 (1H, dd, *J* = 7.4, 5.2 Hz, Glu-α-C*H*COSCH₂), 3.36 (2H, d, *J* = 8.1, 6.8 Hz, COSC*H*H), 2.39 (2H, t, *J* = 7.4 Hz, Glu-α-CHCH₂C*H₂*COOH); 2.24 (1H app. dtd, *J* = 14.8, 7.4, 5.2 Hz, 1H, Glu-α-CHC*H*HCHCOOH), 2.11 (1H, app. dq, *J* = 14.8, 7.4 Hz, 1H, Glu-α-CHCH*H*CHCOOH).

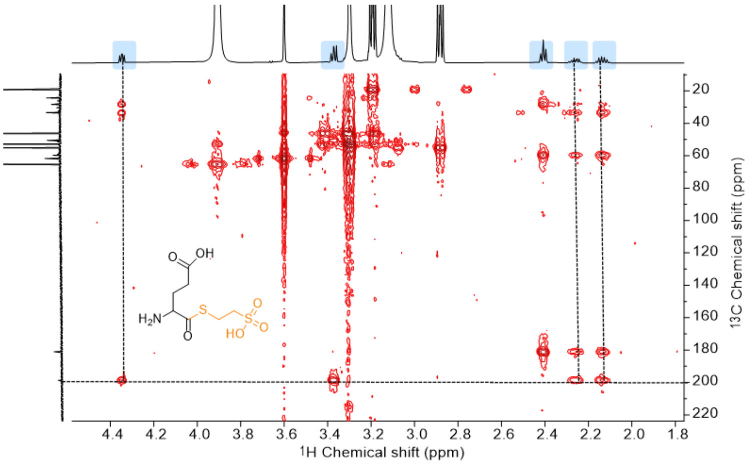

Supplementary Figure 147: ¹H–¹³C HMBC (¹H: 600 MHz [1.8-5.0 ppm], ¹³C: 176 MHz [20-220 ppm], H_2O/D_2O 9:1) spectrum showing the diagnostic ²JCH and ³JCH coupling of glutamic acid-αH and -SCH₂ in $\mathbf{1^{c}_{Glu}}$ *at 4.34 ppm, 3.37 ppm with a resonance at 199.5 ppm, which is characteristic of thioester bond formation.*

Formation of aminoacyl thiol $\mathbf{1_{Asp}}$ from the reaction of NCA $\mathbf{4_{Asp}}$ with 2-mercaptoethanesulfonate ***5c*** *at pH 5*

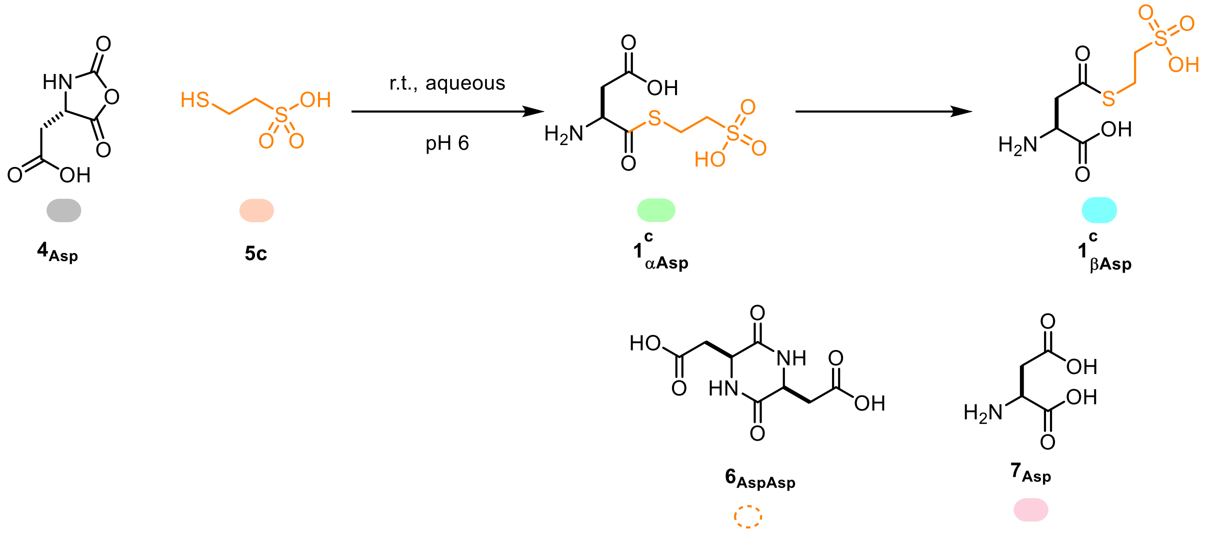

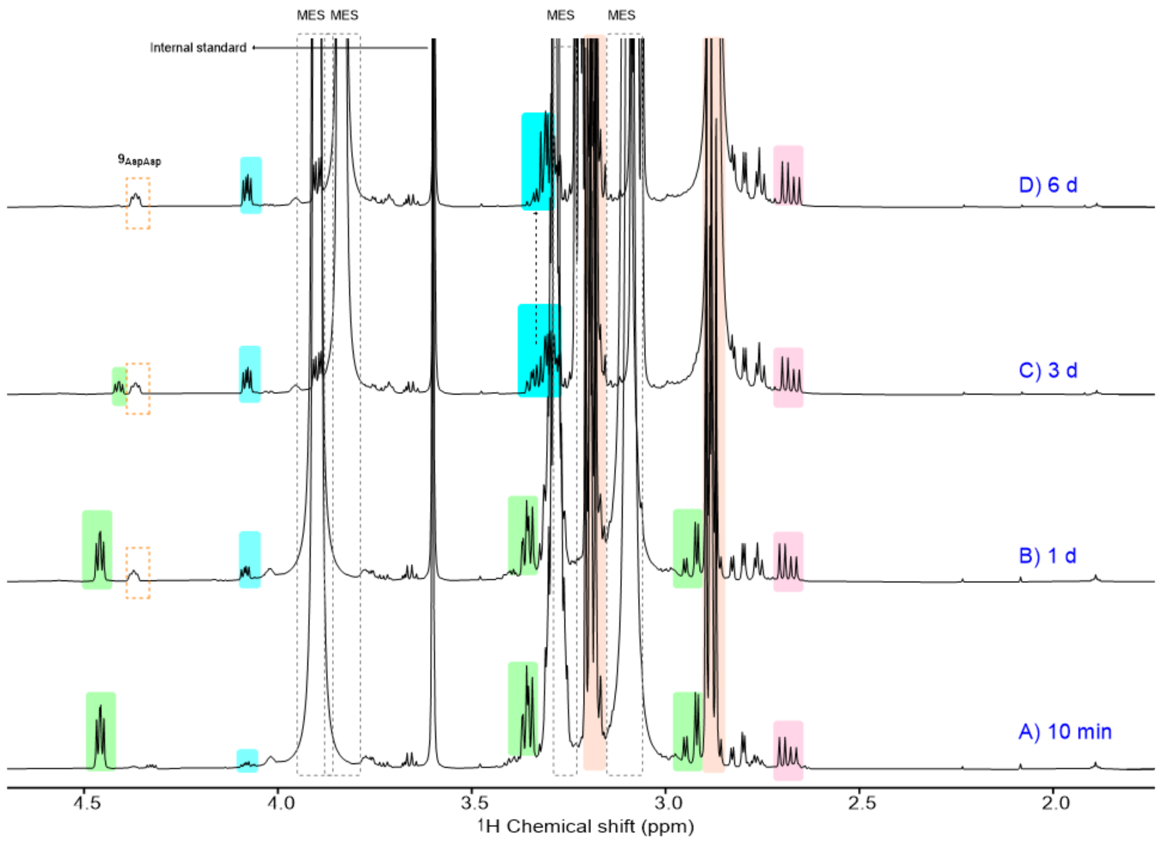

*Supplementary Figure 148: ^{1}H NMR (600 MHz, H_2O/D_2O 9:1, noesygppr1d, 1.4 – 5.0 ppm) spectra to show the reaction of a-aspartic acid-N-carboxyanhydride (**4_{Asp}**, 20 mM) with 2-mercaptoethanesulfonate (**5c**, 100 mM) in MES buffer (200 mM), with PET (10 mM) as an internal standard at pH 6. Set up following General Procedure I after: **A)** 10 min **B)** 1 day **C)** 3 days **D)** 6 days. The α–H resonance of **$1^{c}_{\beta Asp}$** is significantly upfield of the α–H resonance observed in **$1^{c}_{\alpha Asp}$**, this upfield shift is characteristic of an acid moiety, rather than a thioester moiety, at C(1).*

^{1}H NMR (600 MHz, H_2O/D_2O 9:1) $\mathbf{1^{c}_{\alpha Asp}}$ (partial assignment) : δ_H 4.46 (1H, dd, J = 6.6, 4.7 Hz, Asp-α-C*H*$COSCH_2$).

^{1}H NMR (600 MHz, H_2O/D_2O 9:1) $\mathbf{1}^{c}_{\beta Asp}$ (partial assignment) : δ_H 4.08 (1H, dd, *J* = 7.5, 4.4 Hz, Asp-β-*CH*CHHCOSCH$_2$).

DKP, $\mathbf{6}_{AspAsp}$: δ_H 4.37 (1H, t, *J* = 6.6, 4.7 Hz, Asp-α-C*H*CONH).

$\mathbf{7}_{Asp}$: δ_H 2.68 (1H, dd, *J* = 17.5, 8.7 Hz, Asp-α-CHCH*H*COOH).

Entry	Time (h)	$\mathbf{1}^{c}_{\alpha Asp}$ (%)	$\mathbf{1}^{c}_{\beta Asp}$ (%)	$\mathbf{7}_{Asp}$ (%)	$\mathbf{6}_{AspAsp}$ (%)
1	0.1	48	<1	49	<2
2	6	<1	30	60	9

*Supplementary Table 34. Yields (%) for the reaction of a-aspartic acid-N-carboxyanhydride (**4**$_{Asp}$, 20 mM) with 2-mercaptoethanesulfonate (**5c**, 100 mM) in MES buffer (200 mM) with PET (10 mM) as an internal standard at pH 6.*

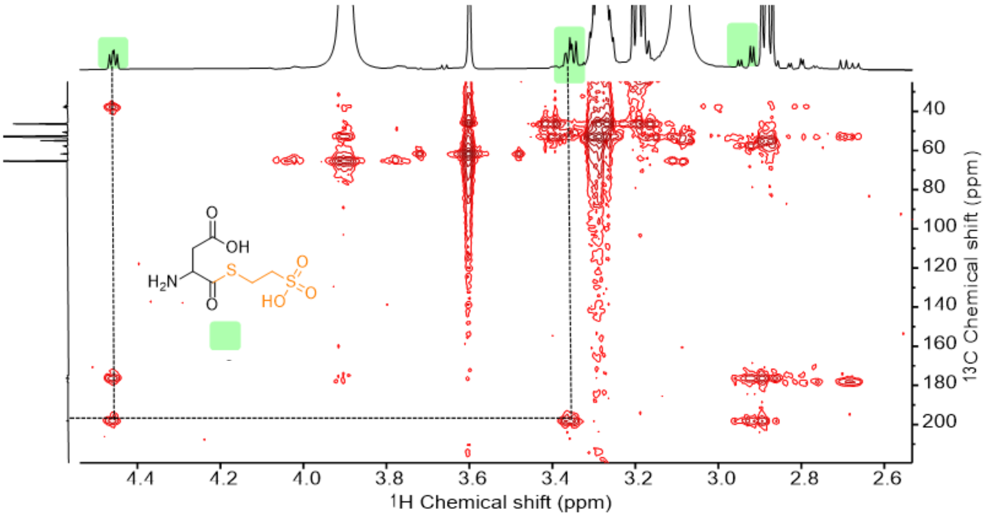

*Supplementary Figure 149: ^{1}H–^{13}C HMBC (^{1}H: 600 MHz [2.6 – 4.5 ppm], ^{13}C: 176 MHz [20 – 220 ppm], H_2O/D_2O 9:1) spectrum showing the diagnostic ^{2}JCH and ^{3}JCH coupling of asparaginyl-aH and -SCH$_2$ in $\mathbf{1}^{c}_{\alpha Asp}$ at 4.46 ppm, 3.37 ppm with a resonance at 198.5 ppm, which is characteristic of thioester bond formation. NMR spectra were acquired after 10 min, showing the formation of $\mathbf{1}^{c}_{\alpha Asp}$ from the reaction of $\mathbf{4}_{Asp}$ with **5c** at pH 6.*

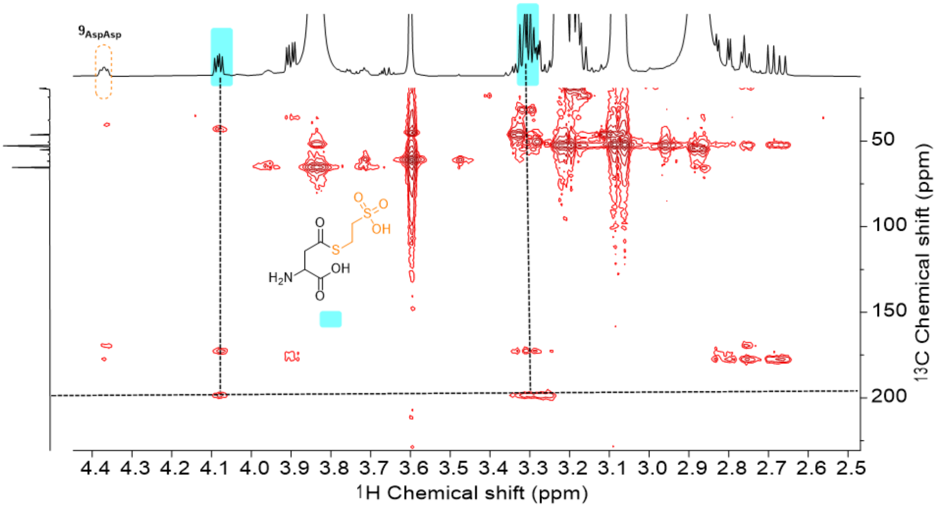

Supplementary Figure 150: ^{1}H–^{13}C HMBC (^{1}H: 600 MHz [2.5 – 4.4 ppm], ^{13}C: 176 MHz [20 – 220 ppm], H_2O/D_2O 9:1) spectrum showing the diagnostic ^{2}JCH and ^{3}JCH coupling of Asp-aH- and -SCH_2 in ***$1'_{\beta Asp}$*** *at 4.08 ppm, 3.30 ppm with a resonance at 198.0 ppm, which is characteristic of thioester bond formation. NMR spectrum acquired after 6 days showing the formation of* ***$1'_{\beta Asp}$*** *from of the reaction of* ***4_{Asp}*** *with thiol* ***5c*** *at pH 6.*

Formation of aminoacyl thiol ***1_{Glx}*** *from the reaction of NCA* ***4_{Glx}*** *with 2-mercaptoethanesulfonate* ***5c*** *at pH 5*

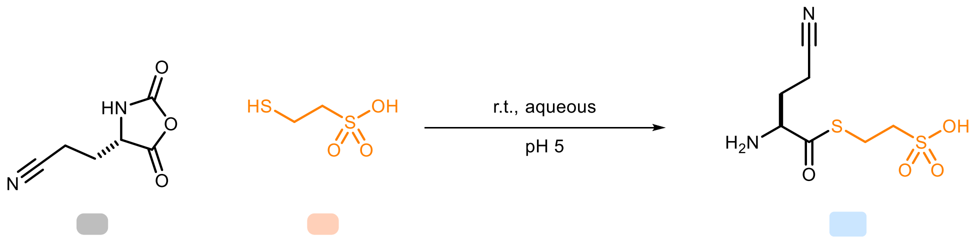

4_{Glx} **5c** **1_{Glx}**

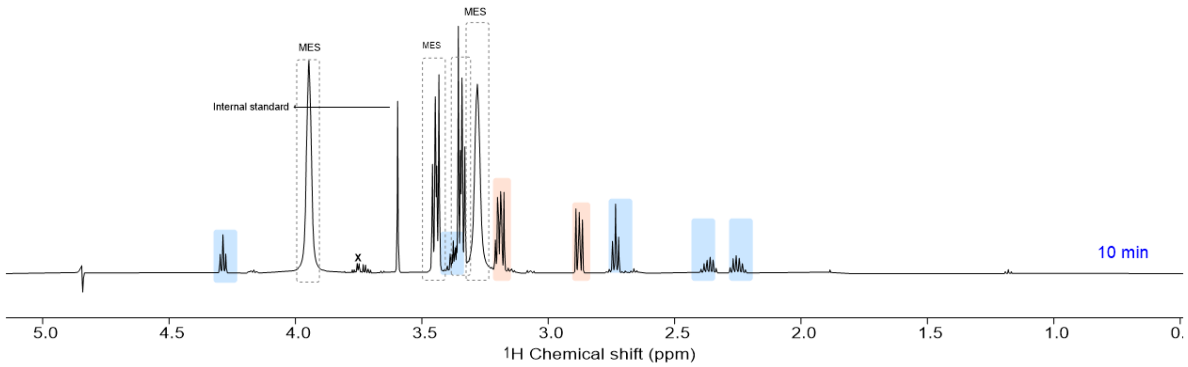

Supplementary Figure 151: ^{1}H NMR (600 MHz, H_2O/D_2O 9:1, noesygppr1d, 1.4–5.0 ppm) spectra to show the reaction of ***4_{Glx}*** *(20 mM) with 2-mercaptoethanesulfonate (**5c**, 100 mM) and MES buffer (200 mM) with PET (10 mM) as an internal standard at pH 5 and room temperature after 10 min. Set up following General Procedure E.*

^{1}H NMR (600 MHz, H_2O/D_2O 9:1) **1^c_{Glx}** (partial assignment) : δ_H 4.29 (1H, t, *J* = 6.6 Hz, Glx-α-C*H*CH_2CH_2CN), 2.73 (2H, t, *J* = 7.4 Hz, Glx-α-CHCH_2C*H*$_2$CN); 2.32 (1H, dq, *J* = 14.5, 7.3 Hz, 1H, Glx-α-CHCH*H*CH_2CN), 2.25 (1H, dq, *J* = 14.5, 7.3 Hz, 1H, Glu-α-CHC*H*HCH$_2$COOH).

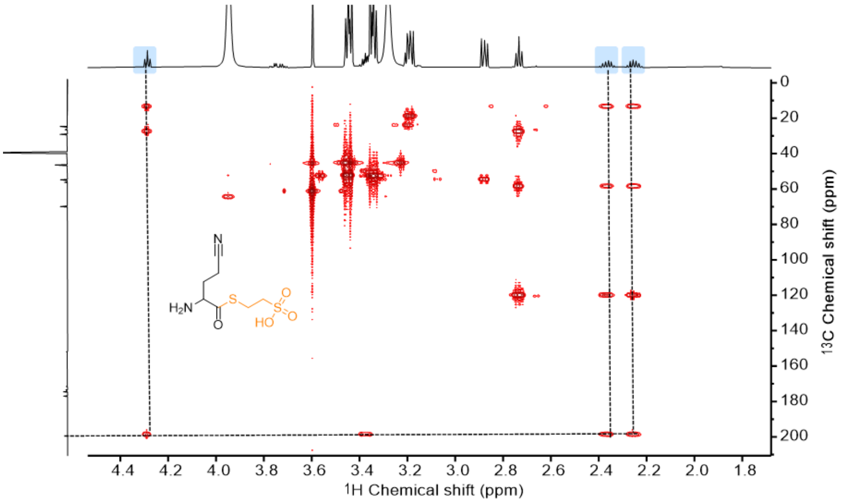

*Supplementary Figure 152: ^{1}H–^{13}C HMBC (^{1}H: 600 MHz [1.8 – 4.4 ppm], ^{13}C: 176 MHz [0 – 220 ppm], H_2O/D_2O 9:1) spectrum showing the diagnostic ^{2}JCH and ^{3}JCH coupling of αH and -SCH$_2$ in **1^c_{Glx}** at 4.29 ppm, 3.37 ppm with a resonance at 199.5 ppm, which is characteristic of thioester bond formation.*

Thiol-mediated aminoacylation of nucleosides **16** from *N*-carboxyanhydrides **4**

*General Procedure J for thiol catalysed aminoacylation of adenosine (**16A**) from NCA **4** and 2-mercaptoethanesulfonate (**5c**)*

Adenosine (**16A**, 2.67 mg, 0.01 mmol), 2-(*N*-morpholino)ethanesulfonic acid (MES, 0.10 mmol), 2-mercaptoethanesulfonate (**3c**, 0.50 mmol), and pentaerythritol (10 mM) were dissolved in degassed H_2O/D_2O (98:2, 0.4 mL), adjusted to pH 5 with NaOH/HCl, and the volume was set to 0.5 mL with degassed H_2O/D_2O (98:2). α-Amino acid-N-carboxyanhydride ($\mathbf{4_{Aaa}}$, 0.30 mmol) was added to the solution in three portions. After each addition the solution was re-adjusted to pH 5 with NaOH/HCl, then vortexed and sonicated for 10 seconds. The solution was then adjusted to pH 6.5 and incubated at room temperature. NMR spectra were acquired periodically. The structure of the α-aminoacyl ester $\mathbf{17^{A}_{Aaa}}$ was confirmed by ^{1}H NMR analysis.

*Formation of aminoacyl-adenosine $\mathbf{17^{A}_{Aaa}}$ from the reaction of adenosine (**16A**) with NCA $\mathbf{4_{Aaa}}$ in the presence of thiol **5c***

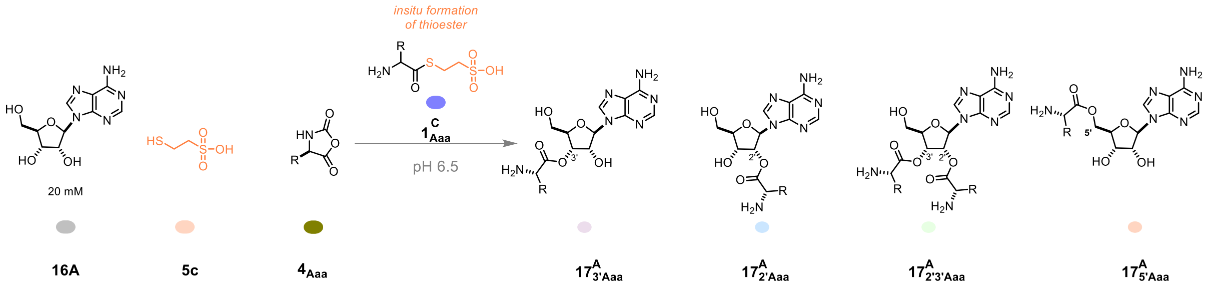

Entry	4_{Aaa}	Time (h)	$17_{3'Aaa}$ (%)	$17_{2'Aaa}$ (%)	Total (%)
1	Gly	24	16	5	21
2	L-Ala	24	25	9	35
3	L-Leu	24	16	6	22
4	L-Asp	18	10	2	12[a]
5	L-Glu	24	9	2	11
6	L-Glx	6	17	5	22
7	L-Val	24	4	1	5
8	L-Phe	24	4	1	5
9	L-Ser	18	11	2	13

*Supplementary Table 35: Total yields for a-aminoacyl ester $\mathbf{17^{A}_{Aaa}}$ in the reaction of $\mathbf{4_{Aaa}}$ (600 mM), 2-mercaptoethanesulfonate (**5c**, 1 M) with adenosine (**16A**, 20 mM) and MES buffer (200 mM) with pentaerythritol (10 mM) as an internal standard at pH 6.5, room temperature. Set up following General Procedure J.*

[a] Initially aminoacylation is highly α-selective. Over several days this changes to become predominantly β-selective (see Supplementary Table 35). At 18 hours ~1% β-Asp aminoacylation was observed.

*Thiol **5c**-catalysed formation of **17^{A}_{Ala}** from the reaction of adenosine (**16A**) with NCA **4_{Ala}***

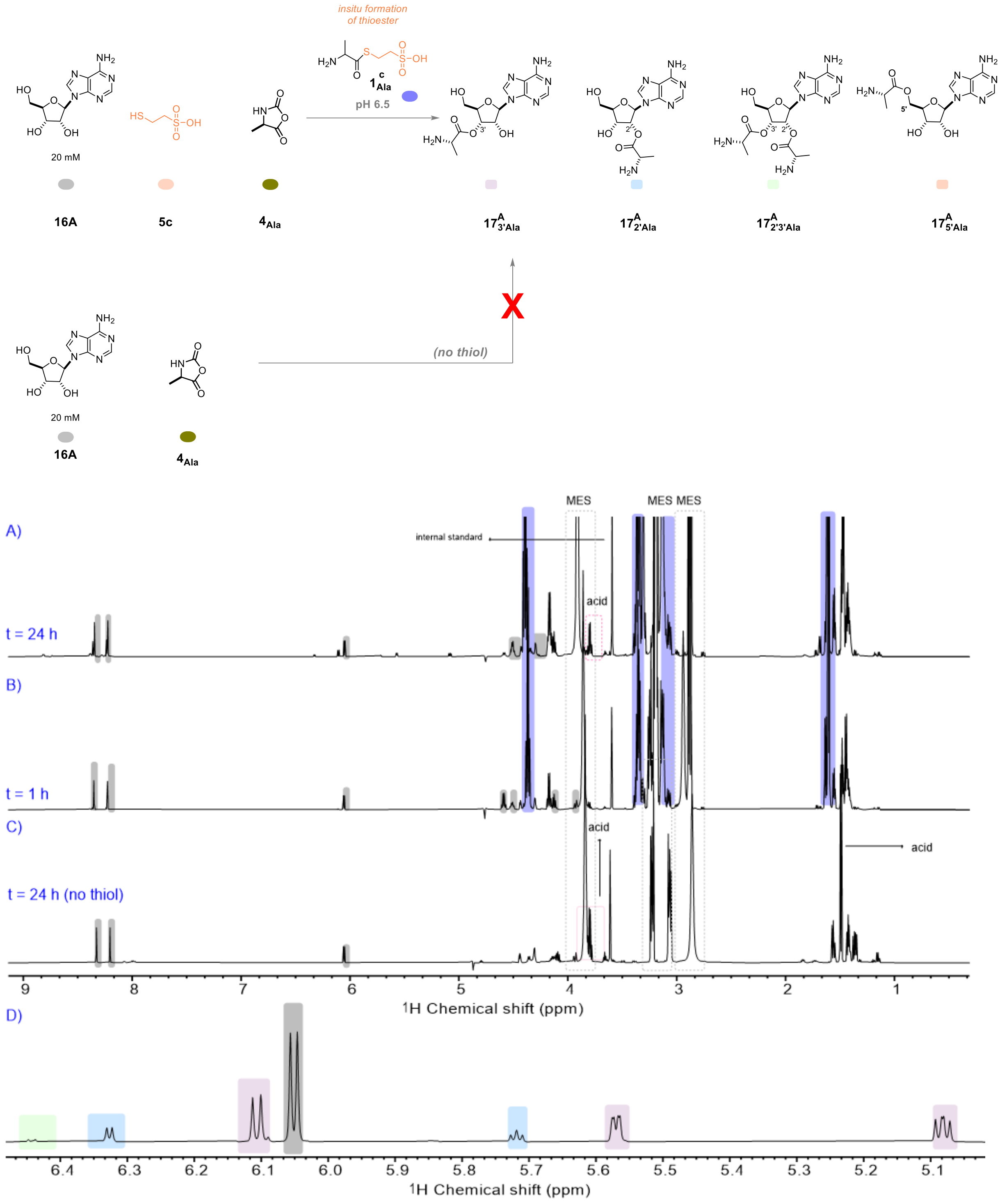

*Supplementary Figure 153: ^{1}H NMR (600 MHz, H_2O/D_2O 9:1, noesygppr1d, 1.0 – 9.0 ppm) spectra to show the reaction of **4_{Ala}** (600 mM) with **5c** (1 M), **16A** (20 mM) and MES buffer (200 mM) at pH 6.5 , using pentaerythritol (10 mM) as an internal standard. Set up following General Procedure J. after: **A)** 24 h; **B)** 1 h; **C)** reaction as in (**A**) but without thiol **3c**; **D)** zoom-in of 24 hour (spectrum **A**) from 5.1 – 6.5 ppm.*

^{1}H NMR (600 MHz, H_2O/D_2O 9:1) **$17^{A}_{3'Ala}$** (partial assignment): δ_H 6.11 (1H, d, *J* = 7.4 Hz, (C1')-H), 5.58 (1H, dd, *J* = 5.4, 1.9 Hz, (C3')-H), 5.09 (1H, dd, *J* = 7.4, 5.5 Hz, (C2')-H).

^{1}H NMR (600 MHz, H_2O/D_2O 9:1) $\mathbf{17^{A}_{2'Ala}}$ (partial assignment): δ_H 6.33 (1H, d, J = 4.7 Hz, (C1')-H), 5.73 (1H, apt. t, J = 4.7 Hz, (C2')-H).

^{1}H NMR (600 MHz, H_2O/D_2O 9:1) $\mathbf{17^{A}_{2'3'Ala}}$ (partial assignment): δ_H 6.45 (1H, d, J = 6.2 Hz, (C1')-H).

*Thiol **5c**-catalysed formation of **17**$^A_{Gly}$ from the reaction of adenosine (**16A**) with NCA **4**$_{Gly}$*

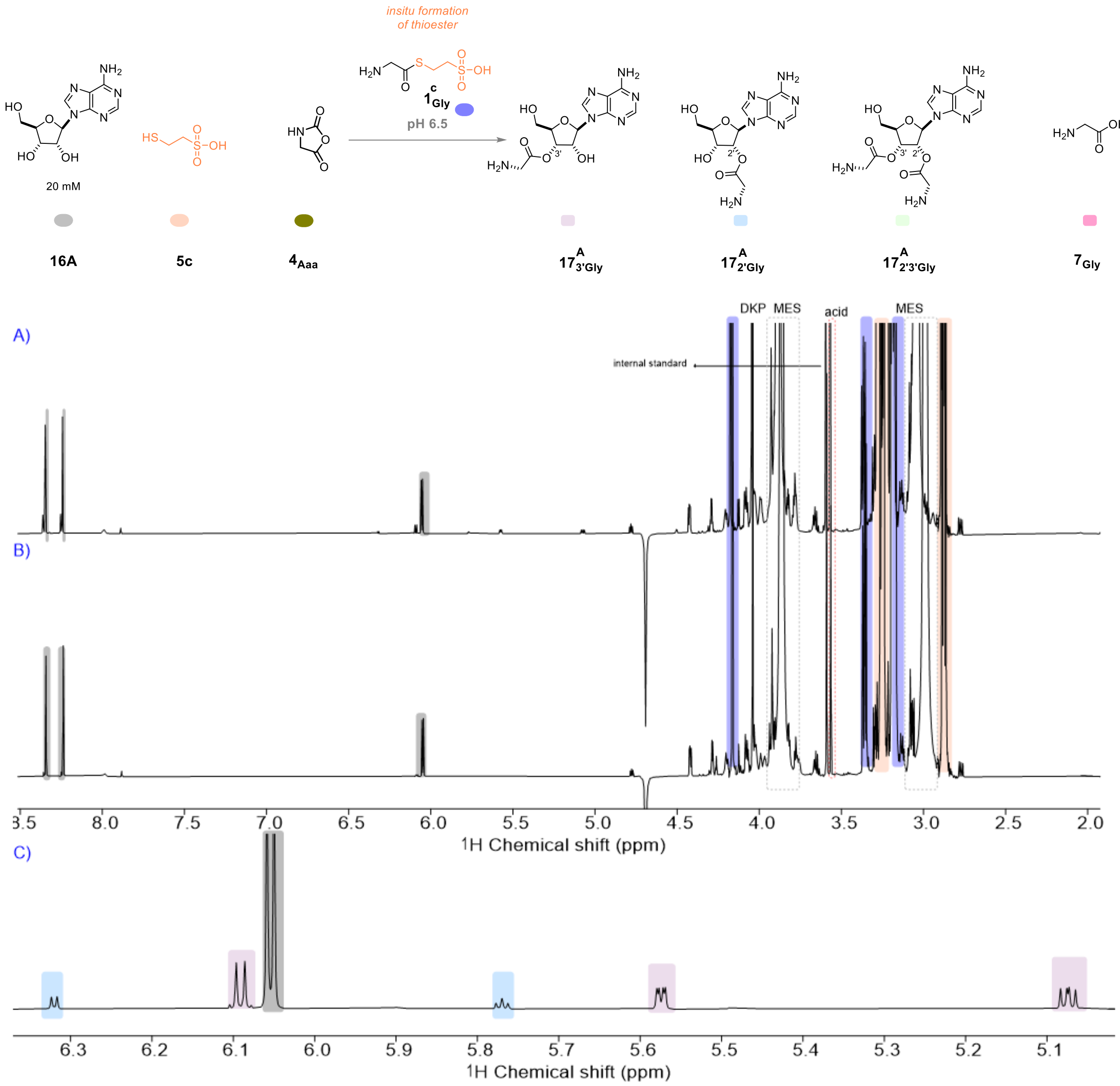

*Supplementary Figure 154: ^{1}H NMR (600 MHz, H_2O/D_2O 9:1, noesygppr1d, 2.0 – 8.5 ppm) spectra to show the reaction of **33**$_{Gly}$ (600 mM) with **5c** (1 M), **16A** (20 mM) and MES buffer (200 mM) at pH 6.5, using pentaerythritol (10 mM) as an internal standard. Set up following General Procedure J after: **A)** 24 h; **B)** 1 h; **C)** zoom-in of 24 hour (spectrum **A**) from 5.1 ppm – 6.5 ppm.*

^{1}H NMR (600 MHz, H_2O/D_2O 9:1) $\mathbf{17^{A}_{3'Gly}}$ (partial assignment): δ_H 6.09 (1H, d, J = 7.4 Hz, (C1')-H), 5.58 (1H, dd, J = 5.5, 2.1 Hz, (C3')-H), 5.08 (1H, dd, J = 7.4, 5.5 Hz, (C2')-H);

^{1}H NMR (600 MHz, H_2O/D_2O 9:1) $\mathbf{17^{A}_{2'Gly}}$ (partial assignment): δ_H 6.32 (1H, d, J = 4.7 Hz, (C1')-H), 5.78 (1H, t, J = 5.2 Hz, (C2')-H);

*Thiol **5c**-catalysed formation of **17^{A}$_{Leu}$** from the reaction of adenosine (**16A**) with NCA **4$_{Leu}$***

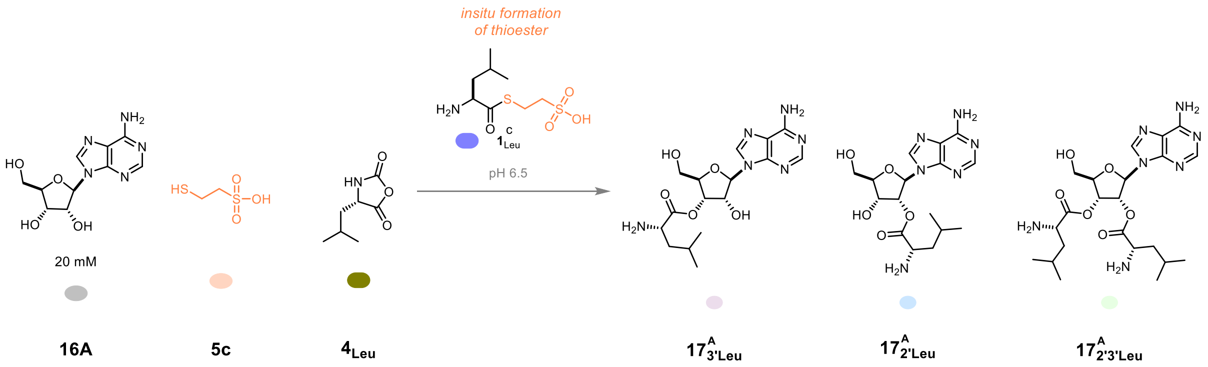

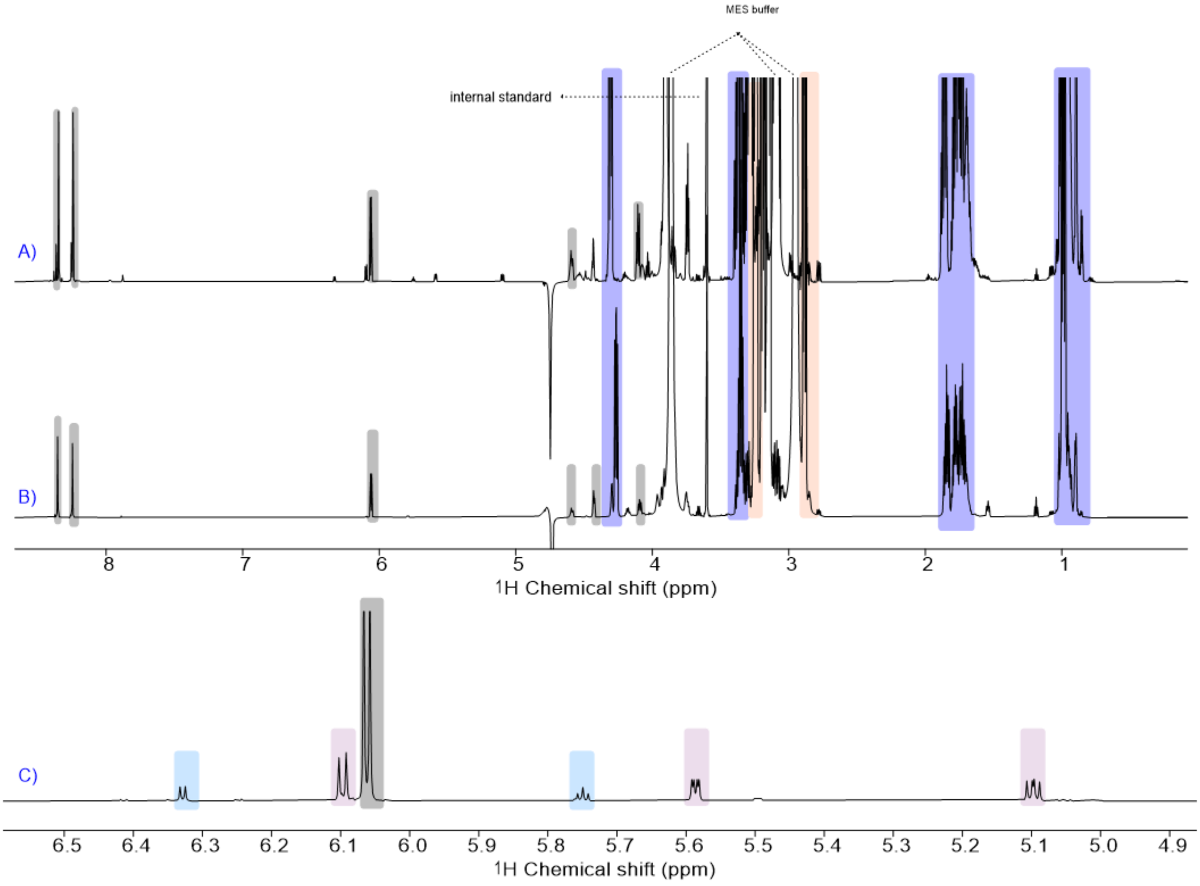

*Supplementary Figure 155: ^{1}H NMR (600 MHz, H_2O/D_2O 9:1, noesygppr1d, 2.0 – 8.5 ppm) spectra to show the reaction of **4$_{Leu}$** (600 mM) with **5c** (1 M), **16A** (20 mM) and MES buffer (200 mM) at pH 6.5, using pentaerythritol (10 mM) as an internal standard. Set up following General Procedure J, after: **A**) 24 h; **B**) 1 h; **C)** zoom-in of 24 hours(spectrum **A**) from 4.9 ppm – 6.5 ppm.*

^{1}H NMR (600 MHz, H_2O/D_2O 9:1) $\mathbf{17^{A}_{3'Leu}}$ (partial assignment): δ_H 6.10 (1H, d, J = 7.4 Hz, (C1')-H), 5.59 (1H, dd, J = 5.4, 2.1 Hz, (C3')-H), 5.08 (1H, dd, J = 7.4, 5.4 Hz, (C2')-H);

^{1}H NMR (600 MHz, H_2O/D_2O 9:1) $\mathbf{17^{A}_{2'Leu}}$ (partial assignment): δ_H 6.33 (1H, d, J = 4.7 Hz, (C1')-H), 5.75 (1H, t, J = 5.4 Hz, (C2')-H);

*Thiol **5c**-catalysed formation of **17^{A}$_{Val}$** from the reaction of adenosine (**16A**) with NCA **4$_{Val}$***

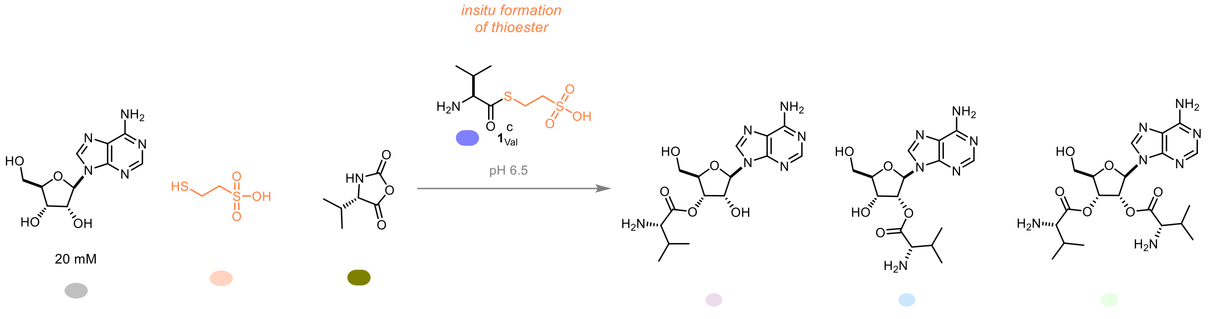

16A **5c** **4$_{Val}$** **$17^{A}_{3'Val}$** **$17^{A}_{2'Val}$** **$17^{A}_{2'3'Val}$**

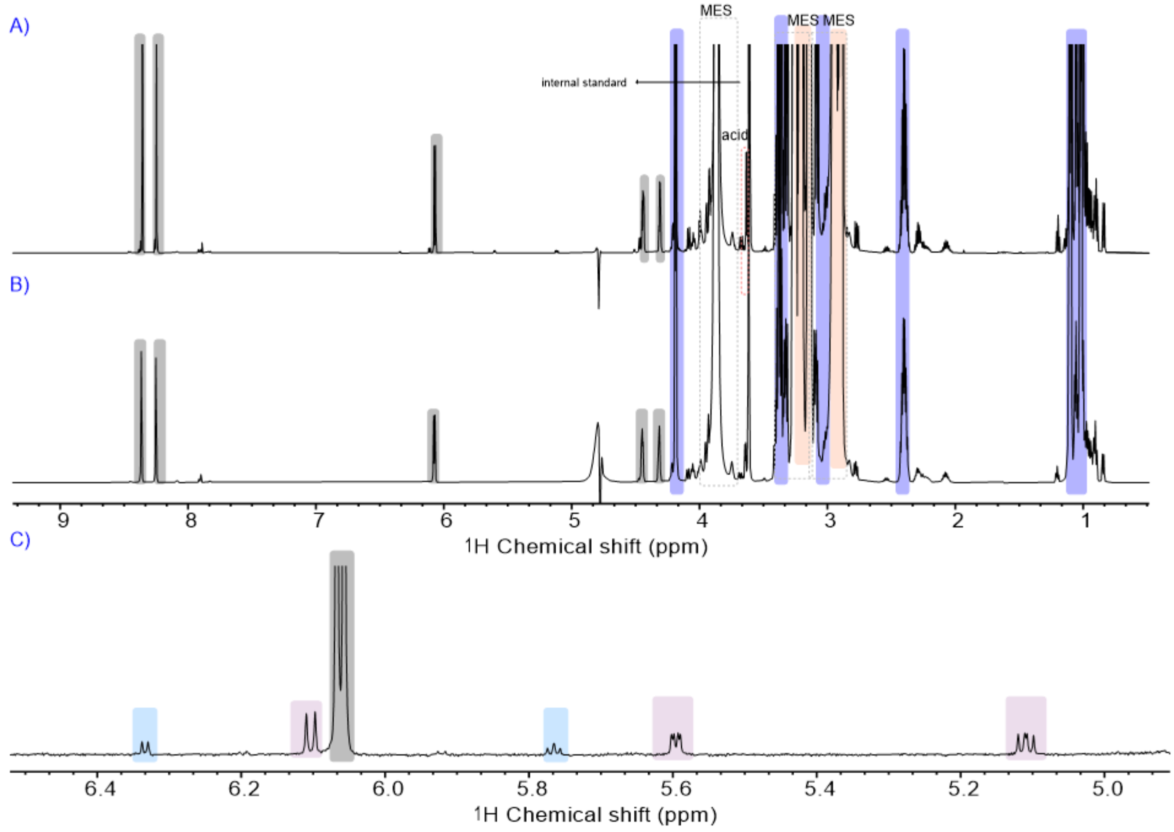

*Supplementary Figure 156: ^{1}H NMR (600 MHz, H_2O/D_2O 9:1, noesygppr1d, 1.0 – 9.0 ppm) spectra to show the reaction of **4$_{Val}$** (600 mM) with **5c** (1 M), **16A** (20 mM) and MES buffer (200 mM) at pH 6.5, using pentaerythritol (10 mM) as an internal standard. Set up following General Procedure J after: **A)** 24 h; **B)** 1 h; **C)** inset zoom-in of 24 hour (spectrum **A**) from 5.0 ppm – 6.4 ppm.*

^{1}H NMR (600 MHz, H_2O/D_2O 9:1) $\mathbf{17^{A}_{3'Val}}$ (partial assignment): δ_H 6.10 (1H, d, J = 7.3 Hz, (C1')-H), 5.60 (1H, dd, J = 5.4, 2.1 Hz, (C3')-H), 5.11 (1H, dd, J = 7.3, 5.4 Hz, (C2')-H);

^{1}H NMR (600 MHz, H_2O/D_2O 9:1) $\mathbf{17^{A}_{2'Val}}$ (partial assignment): δ_H 6.33 (1H, d, *J* = 4.9 Hz, (C1')-H), 5.78 (1H, t, *J* = 5.2 Hz, (C2')-H).

*Thiol **5c**-catalysed formation of **17^{A}_{Ser}** from the reaction of adenosine (**16A**) with NCA **4_{Ser}***

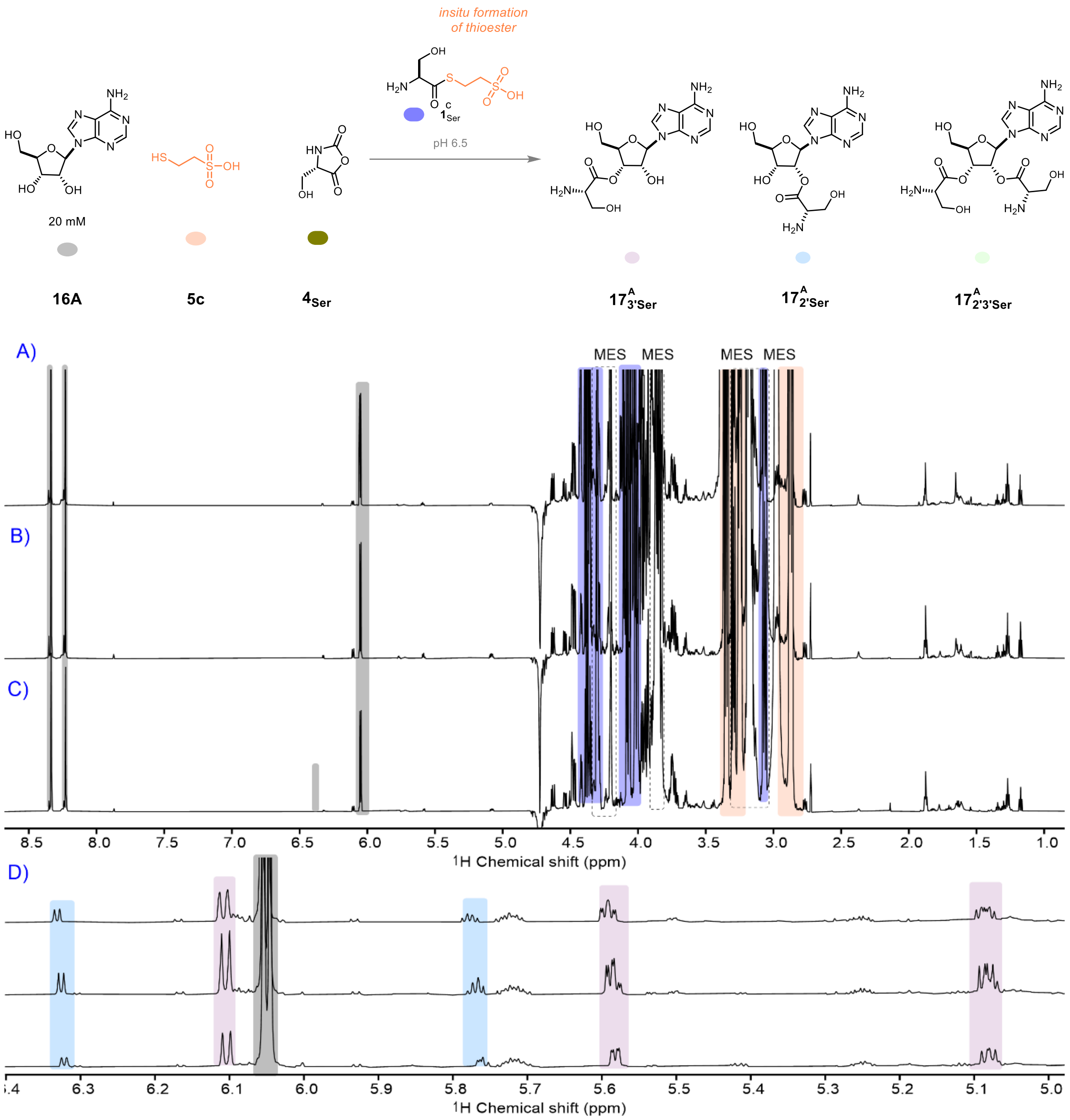

*Supplementary Figure 157: ^{1}H NMR (600 MHz, H_2O/D_2O 9:1, noesygppr1d, 1.0 – 9.0 ppm) spectra to show the reaction of **4_{Ser}** (600 mM) with **5c** (1 M), **16A** (20 mM) and MES buffer (200 mM) at pH 6.5. Set up following General Procedure J after: **A)** 48 h; **B)** 18 h; **C)** 1 h; **D)** inset zoom-in of spectrum **A** to **C** from 5.0 ppm – 6.4 ppm. It was observed that, in the presence of MES buffer (200 mM), racemization occurred over several days. A reaction was also performed without buffer, and no racemization was observed as shown in see Supplementary Figure 158.*

.^{1}H NMR (600 MHz, H_2O/D_2O 9:1) $\mathbf{17^{A}_{3'Ser}}$ (partial assignment – both diastereomers a + b): δ_H 6.11 (2H, d, *J* = 7.5 Hz, (C1')-Ha + (C1')-Hb), 5.59 (2H, m, overlapped, (C3')-Ha + (C3')-Hb), 5.11 (2H, dd, *J* = 7.5, 5.4 Hz, (C2')-Ha + (C2')-Hb);

^{1}H NMR (600 MHz, H_2O/D_2O 9:1) $\mathbf{17^{A}_{2'Ser}}$ (partial assignment – both diastereomers a + b): δ_H 6.33 (2H, d, *J* = 4.9 Hz, (C1')-Ha + (C1')-Hb), 5.77 (2H, m, overlapped, (C2')-Ha + (C2')-Hb).

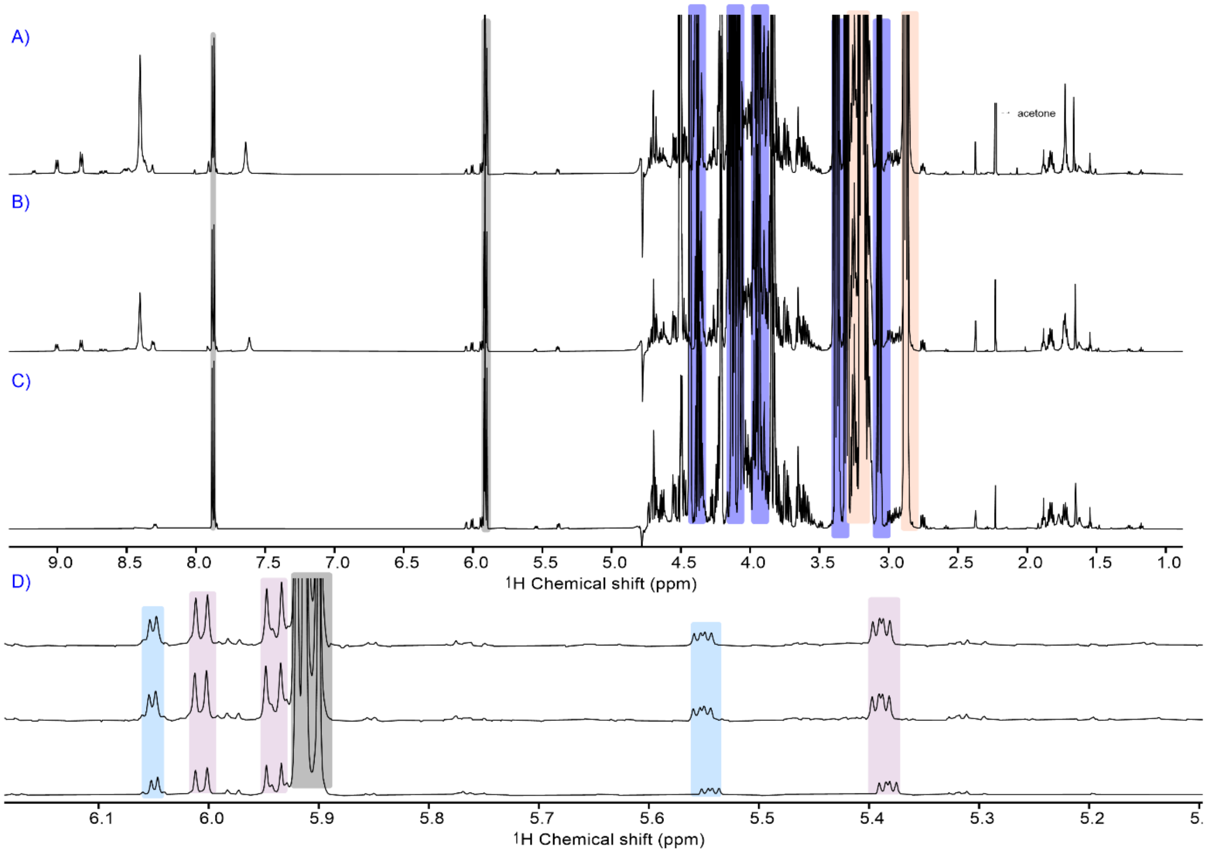

Supplementary Figure 158: ^{1}H NMR (600 MHz, H_2O/D_2O 9:1, noesygppr1d, 1.0 – 9.0 ppm) spectra to show the reaction of $\mathbf{4_{Ser}}$ *(600 mM) with* ***5c*** *(1 M),* ***16U*** *(20 mM) at pH 6.5. Set up following General Procedure J (without addition of buffer) after:* ***A****) 48 h;* ***B****) 24 h;* ***C****) 1 h;* ***D****) inset zoom-in of spectrum* ***A*** *to* ***C*** *from 5.0 ppm – 6.4 ppm.*

*Thiol **5c**-catalysed formation of **17^{A}_{Phe}** from the reaction of adenosine (**16A**) with NCA **4_{Phe}***

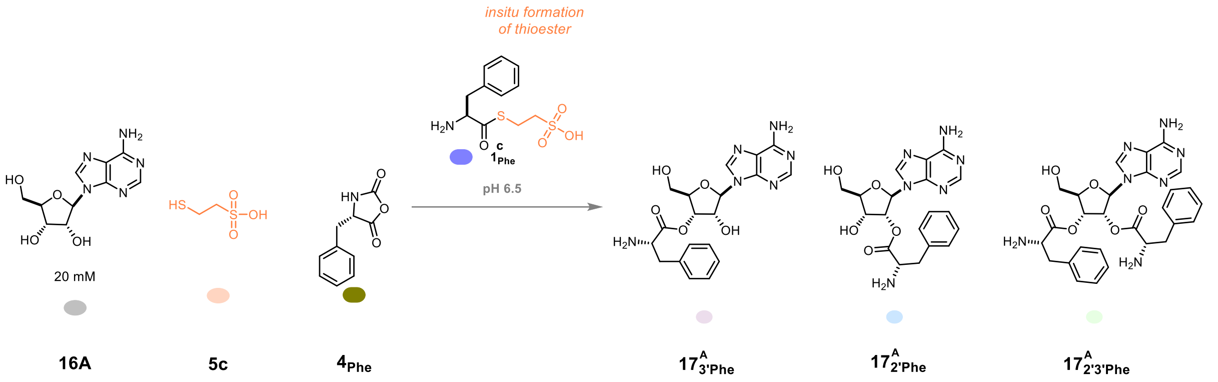

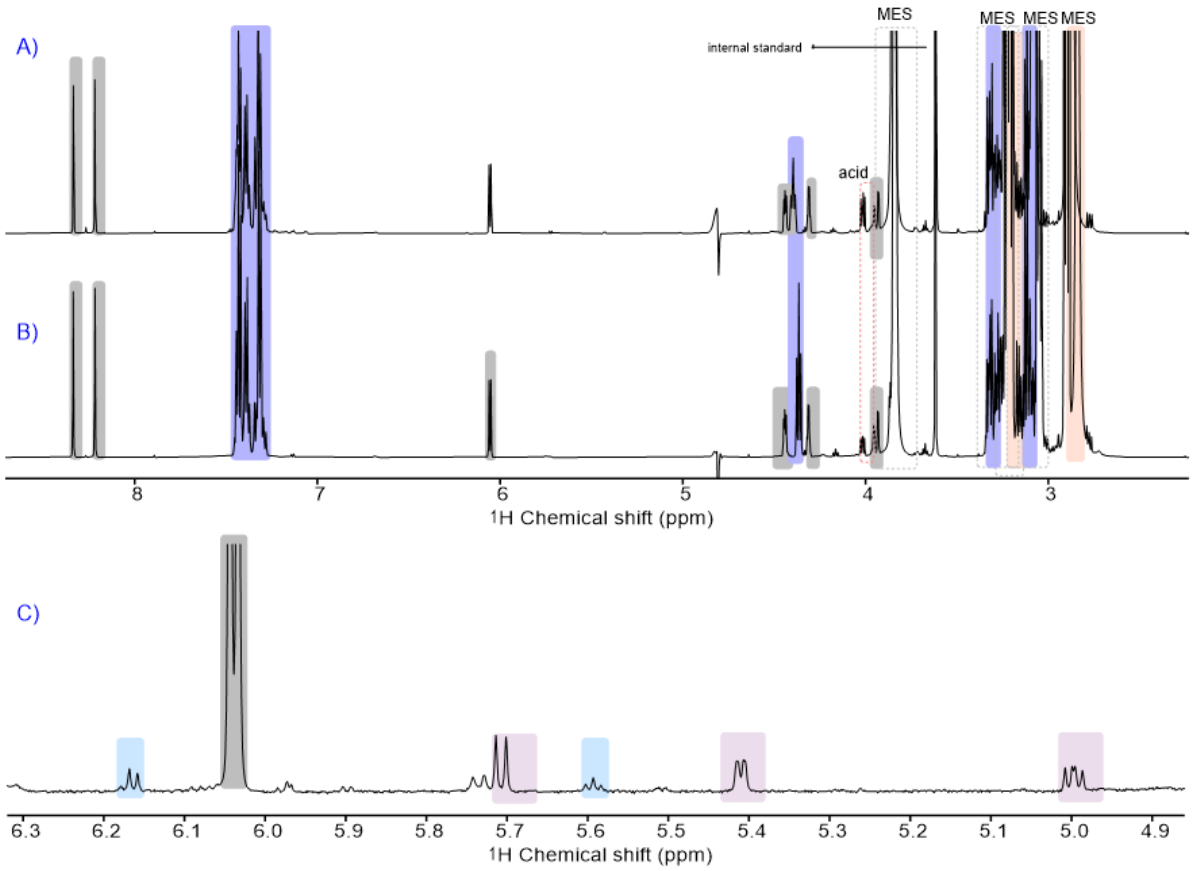

*Supplementary Figure 159: ¹H NMR (600 MHz, H_2O/D_2O 9:1, noesygppr1d, 1.0 – 9.0 ppm) spectra to show the reaction of **4_{Phe}** (600 mM) with **5c** (1 M), **16A** (20 mM) and MES buffer (200 mM) at pH 6.5, using pentaerythritol (10 mM) as an internal standard. Set up following General Procedure J, after: **A)** 24 h; **B)** 1 h; **C)** zoom-in of 24 hour (spectrum **A**) from 4.9 ppm – 6.3 ppm.*

¹H NMR (600 MHz, H_2O/D_2O 9:1) **$17^{A}_{3'Phe}$** (partial assignment): δ_H 5.72 (1H, d, *J* = 7.6 Hz, (C1')-H), 5.43 (1H, d, *J* = 5.4 Hz, (C3')-H), 5.01 (1H, dd, *J* = 7.6, 5.4 Hz, (C2')-H).

¹H NMR (600 MHz, H_2O/D_2O 9:1) **$17^{A}_{2'Phe}$** (partial assignment): δ_H 6.18 (1H, d, *J* = 5.7 Hz, (C1')-H), 5.61 (1H, t, *J* = 5.7 Hz, (C2')-H).

*Thiol **5c**-catalysed formation of **17^{A}_{Glu}** from the reaction of adenosine (**16A**) with NCA **4_{Glu}***

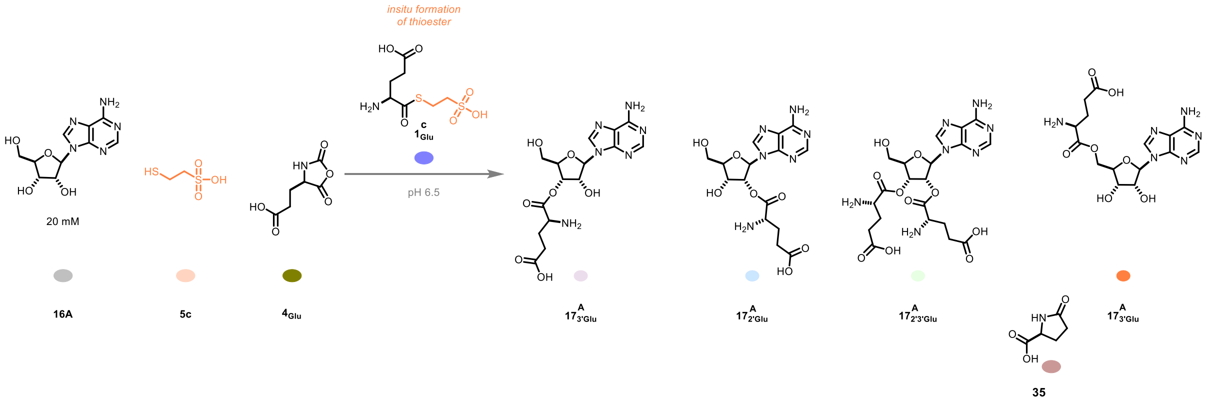

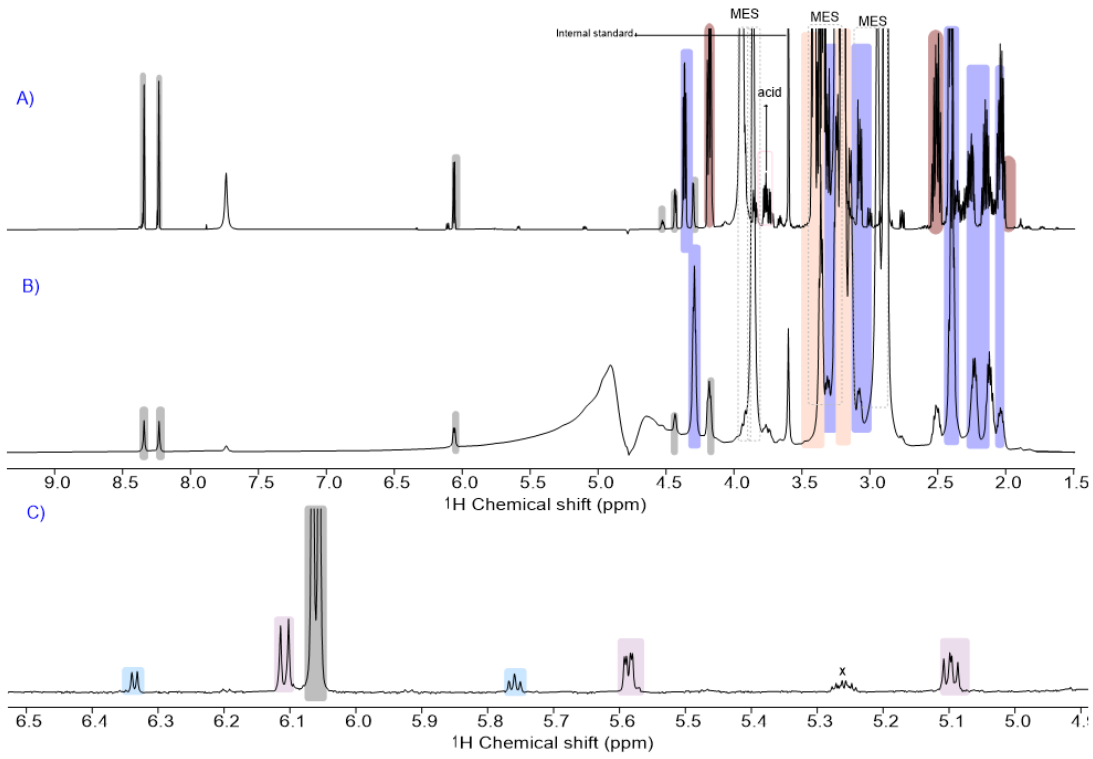

*Supplementary Figure 160: ¹H NMR (600 MHz, H_2O/D_2O 9:1, noesygppr1d, 1.5 – 9.0 ppm) spectra to show the reaction of **4_{Glu}** (600 mM) with **5c** (1 M), **16A** (20 mM) and MES buffer (200 mM) at pH 6.5, using pentaerythritol (10 mM) as an internal standard. Set up following General Procedure J, after: **A**) 24 h; **B**) 1 h; **C)** inset zoom-in of 24 hours (spectrum **A**) from 4.9 ppm – 6.4 ppm. X = unknown by-products derived from NCAs. These are not related to nucleosides and are observed in control reactions of NCAs without nucleoside (see Supplementary Figure 163).*

¹H NMR (600 MHz, H_2O/D_2O 9:1) **$17^{A}_{3'Glu}$** (partial assignment): δ_H 6.11 (1H, d, *J* = 7.4 Hz, (C1')-H), 5.59 (1H, dd, *J* = 5.4, 2.1 Hz, (C3')-H), 5.10 (1H, dd, *J* = 7.4, 5.4 Hz, (C2')-H);

¹H NMR (600 MHz, H_2O/D_2O 9:1) **$17^{A}_{2'Glu}$** (partial assignment): δ_H 6.34 (1H, d, *J* = 4.7 Hz, (C1')-H), 5.76 (1H, t, *J* = 4.7 Hz, (C2')-H).

¹H NMR (600 MHz, H_2O/D_2O 9:1) **35** (partial assignment): δ_H 4.18 (1H, ddt, J = 8.9, 5.9, 1.3 Hz, Glu-α-C*H*COOH).

*Thiol **5c**-catalysed formation of **17$^{A}_{Glx}$** from the reaction of adenosine (**16A**) with NCA **4$_{Glx}$***

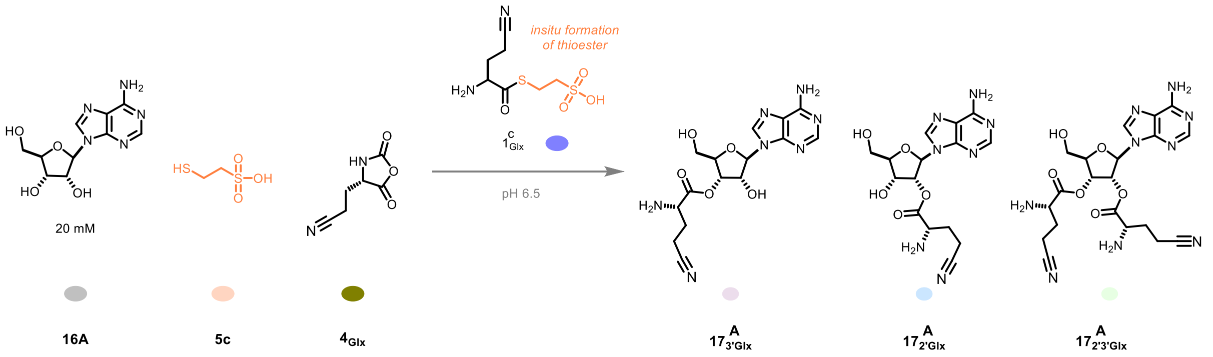

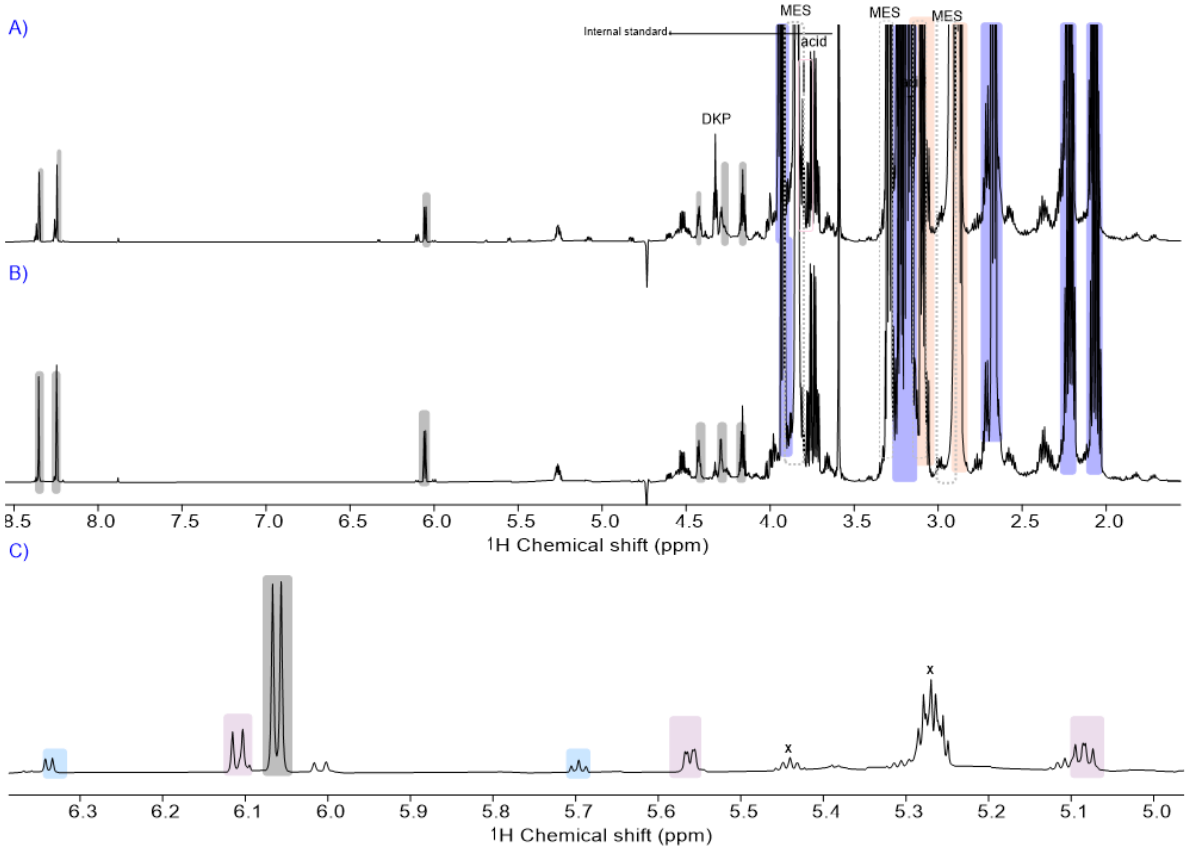

*Supplementary Figure 161: ^{1}H NMR (600 MHz, H_2O/D_2O 9:1, noesygppr1d, 1.5 – 8.5 ppm) spectra to show the reaction of **4$_{Glx}$** (600 mM) with **5c** (1 M), **16A** (20 mM) and MES buffer (200 mM) at pH 6.5, using pentaerythritol (10 mM) as an internal standard. Set up following General Procedure J, after: **A)** 6 h; **B)** 1 h; **C)** inset zoom-in of spectrum **A** from 4.9 ppm - 6.4 ppm. X = unknown by-products derived from NCAs. These are not related to nucleosides and are observed in control reactions of NCAs without nucleoside (see Supplementary Figure 163).*

^{1}H NMR (600 MHz, H_2O/D_2O 9:1) **$17^{A}_{3'Glx}$** (partial assignment): δ_H 6.11 (1H, d, *J* = 7.4 Hz, (C1')-H), 5.59 (1H, dd, *J* = 5.4, 1.9 Hz, (C3')-H), 5.10 (1H, dd, *J* = 7.4, 5.4 Hz, (C2')-H).

^{1}H NMR (600 MHz, H_2O/D_2O 9:1) **$17^{A}_{2'Glx}$** (partial assignment): δ_H 6.34 (1H, d, *J* = 4.7 Hz, (C1')-H), 5.73 (1H, apt t., *J* = 4.7 Hz, (C2')-H).

*Thiol **5c**-catalysed formation of $\mathbf{17^{A}_{Asp}}$ from the reaction of adenosine (**16A**) with NCA $\mathbf{4_{Asp}}$*

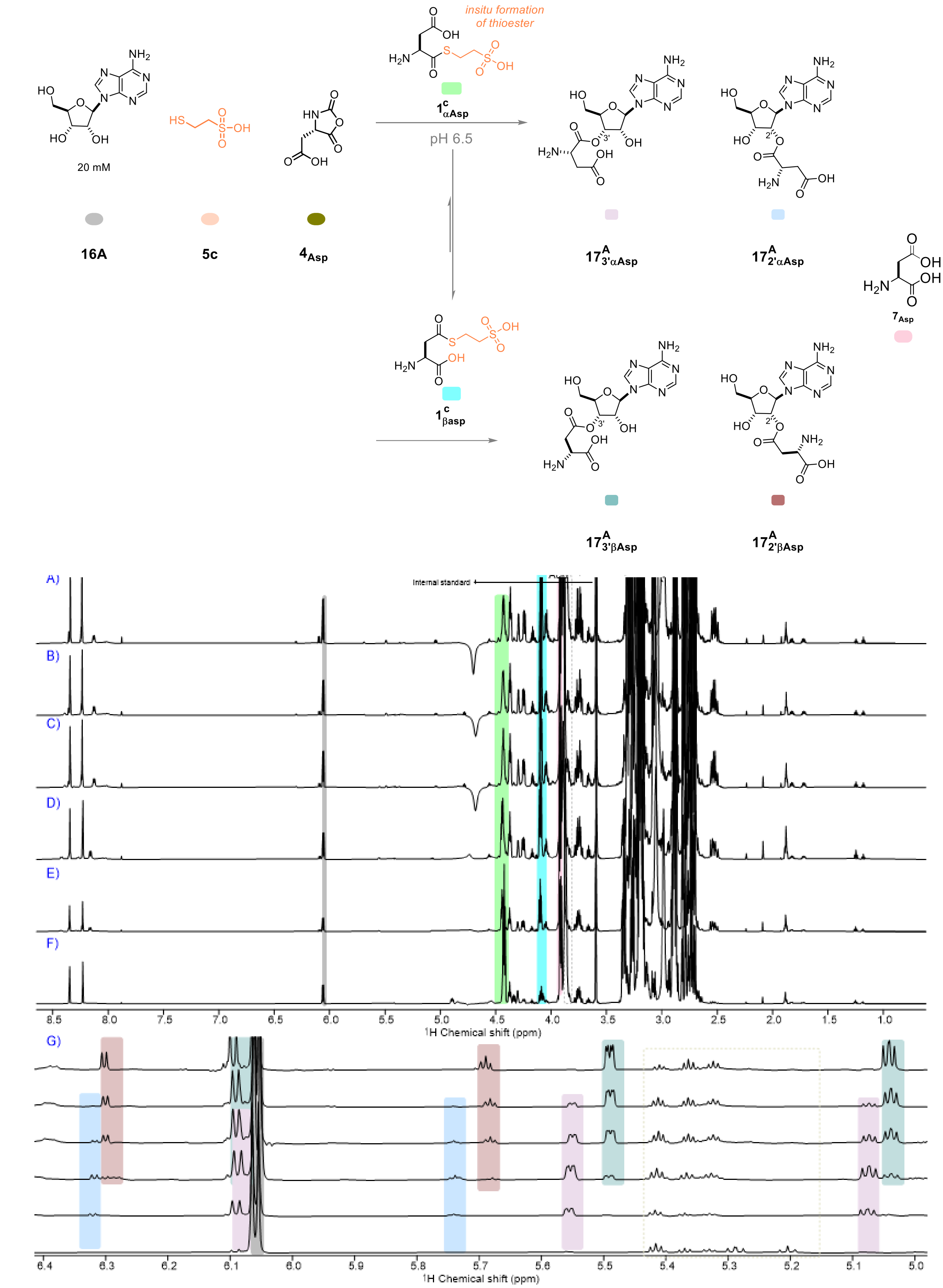

*Supplementary Figure 162: ¹H NMR (600 MHz, H_2O/D_2O 9:1, noesygppr1d, 1.0 – 8.5 ppm) spectra to show the reaction of aspartic N-carboxyanhydride $\mathbf{4_{Asp}}$ (600 mM) with **5c** (1 M) and **16A** (20 mM) in MES buffer (200 mM) at pH 6.5 and room temperature, with pentaerythritol*

(10 mM) as an internal standard. Set up following General Procedure J, after: ***A)*** *5 days* ***B)*** *3 days;* ***C)*** *2.2 days;* ***D)*** *1 day;* ***E)*** *18 hours; and* ***F)*** *1 hour.* ***G)*** *Spectra A-F from 5.0 – 6.4 ppm. NMR resonances 5.15 – 5.45 ppm are unknown by-products derived from NCAs. These are not related to nucleosides and are observed in control reactions of NCAs without nucleoside (see Supplementary Figure 163).*

Entry	Time (days)	$17^{A}_{\alpha Asp}$ (%)	$17^{A}_{\beta Asp}$ (%)	Selectivity ($17^{A}_{\alpha Asp}$:$17^{A}_{\beta Asp}$)
1	0.1	2	--	--
2	0.75	11	<1	>11:1
3	1	10	2	5:1
4	2.2	5	8	1:1.6
5	3	2	12	1:6
6	4	<1	15	1:>15
7	5	<1	17	1:>17

Supplementary Table 36: Yield and α/β-selectivity of $\mathbf{17^{A}_{Asp}}$ *from the reaction of aspartic N-carboxyanhydride* $\mathbf{4_{Asp}}$ *(600 mM) with* ***5c*** *(1 M) and* ***16A*** *(20 mM) in MES buffer (200 mM) at pH 6.5 and room temperature.*

^{1}H NMR (600 MHz, H_2O/D_2O 9:1) $\mathbf{17^{A}_{3'\alpha Asp}}$ (partial assignment) : δ_H 6.09 (1H, d, J = 7.4 Hz, (C1')-H), 5.55 (1H, dd, J = 5.4, 2.1 Hz, (C3')-H), 5.07 (1H, dd, J = 7.4, 5.4 Hz, (C2')-H).

^{1}H NMR (600 MHz, H_2O/D_2O 9:1) $\mathbf{17^{A}_{2'\alpha Asp}}$ (partial assignment) : δ_H 6.32 (1H, d, J = 5.1 Hz, (C1')-H), 5.74 (1H, t, J = 5.1 Hz, (C2')-H).

^{1}H NMR (600 MHz, H_2O/D_2O 9:1) $\mathbf{17^{A}_{3'\beta Asp}}$ (partial assignment) : δ_H 6.09 (1H, d, J = 7.2 Hz, (C1')-H), 5.49 (1H, dd, J = 5.5, 2.3 Hz, (C3')-H), 5.04 (1H, dd, J = 7.2, 5.5 Hz, (C2')-H);

^{1}H NMR (600 MHz, H_2O/D_2O 9:1) $\mathbf{17^{A}_{2'\beta Asp}}$ (partial assignment) : δ_H 6.30 (1H, d, J = 5.1 Hz, (C1')-H), 5.68 (1H, t, J = 5.1 Hz, (C2')-H).

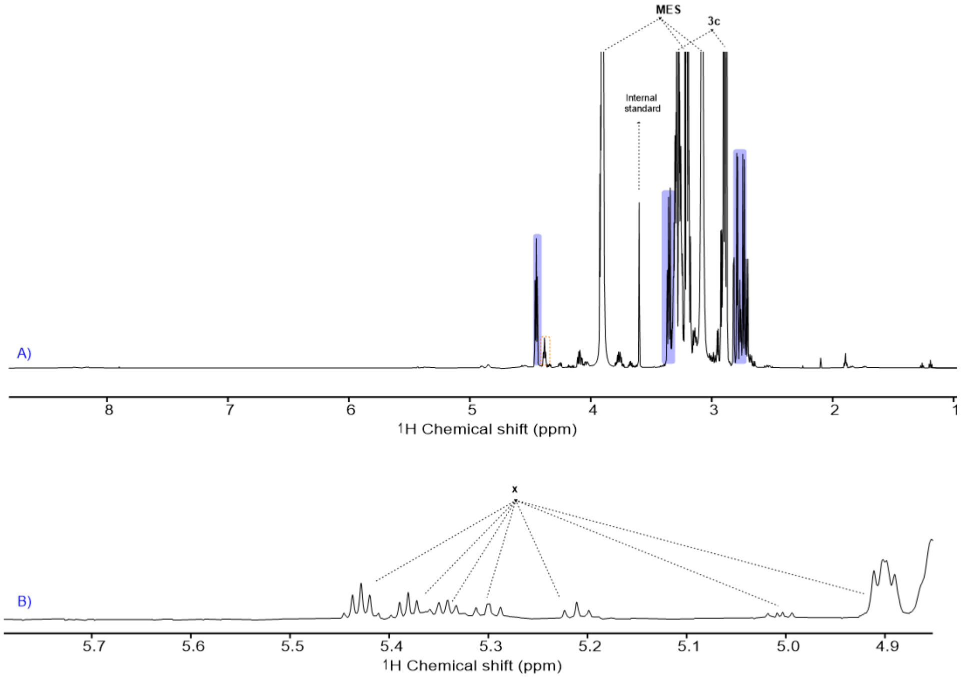

*Supplementary Figure 163: ^{1}H NMR (600 MHz, H_2O/D_2O 9:1, noesygppr1d, 1.0 – 8.5 ppm) spectra to show the reaction of aspartic N-carboxyanhydride **33_{Asp}** (600 mM) with **3c** (1 M) in MES buffer (400 mM) at pH 6.5 and room temperature (without nucleoside), with pentaerythritol (10 mM) as an internal standard. Set up following General Procedure E. **A)** after 1 h, and **B)** spectrum **A** from 4.9 ppm – 5.7 ppm. X = by-products from the reaction of NCAs.*

*Thiol **3c**-catalysed formation of **18**$^{A}_{Glu}$ from the reaction of adenosine (**17A**) with aspartic anhydride **35**$_{Asp}$*

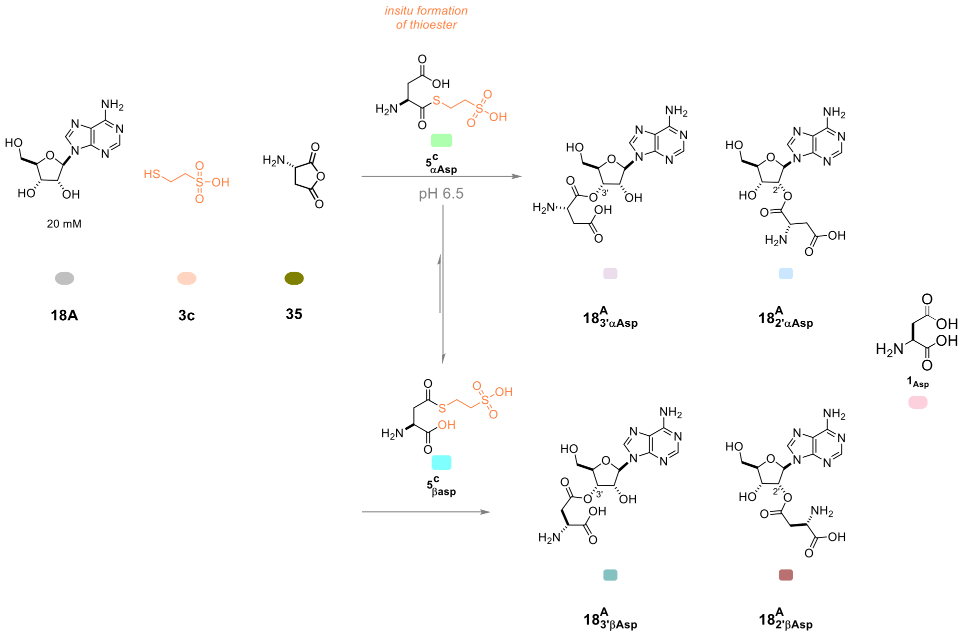

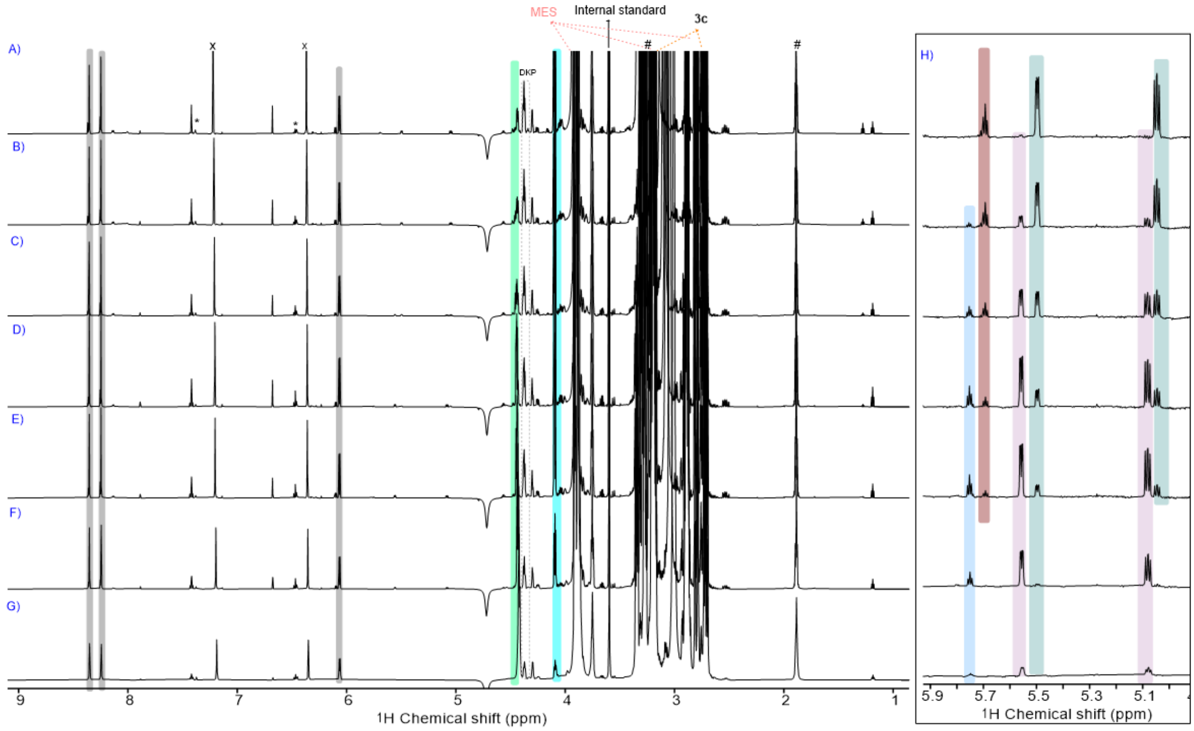

*Supplementary Figure 164: ^{1}H NMR (600 MHz, H_2O/D_2O 9:1, noesygppr1d, 1.0 – 9.0 ppm) spectra to show the reaction of aspartate anhydride **34**$_{Asp}$ (600 mM) with **5c** (1 M) and **16A** (20 mM) in MES buffer (500 mM), with pentaerythritol (10 mM) as an internal standard at pH 6.5 and room temperature. Set up following General Procedure J, after: **A)** 5 days; **B)** 4 days; **C)** 3 days; **D)** 2 days; **E)** 1day; **F)** 12 hours; and **G)** 1 hour. Inset **(H)** shows each adjacent spectrum from 5.0 – 5.9 ppm. Aspartic anhydride **34**$_{Asp}$ was synthesised from **7**$_{Asp}$ following a reported protocol.[9] **34**$_{Asp}$ was recrystallised once from THF and then used without further purification due to its instability. **34**$_{Asp}$ contains H-phosphonate and THF from synthesis. X = H-phosphonate; # = THF.*

Acidic ethanolysis of aspartic anhydride **34**$_{Asp}$ is highly α-selective (>95%),[10,11] this selectivity parallels the kinetic reactivity observed upon reaction of aspartic anhydride **34**$_{Asp}$ and thiol **5c** at pH 6.5.

Entry	Time (days)	$17^{A}_{\alpha Asp}$ (%)	$17^{A}_{\beta Asp}$ (%)	Selectivity ($17^{A}_{\alpha Asp}$:$17^{A}_{\beta Asp}$)
1	0.1	3	--	--
2	0.5	>12	<1	>12:1
4	1	11	4	2.75:1
5	2	6	6	1:1
6	3	2	12	1:6
7	4	1	13	1:13
8	5	<1	17	1:>17

Supplementary Table 37: Yield and α/β-selectivity of $\mathbf{17^{A}_{Asp}}$ *from the reaction of aspartate anhydride* $\mathbf{34_{Asp}}$ *(600 mM) with* ***5c*** *(1 M),* ***16A*** *(20 mM) in MES buffer (500 mM) at pH 6.5 and room temperature.*

*Formation of aminoacyl thiol **1_{Asp}** from the reaction of aspartic anhydride **34_{Asp}** with 2-mercaptoethanesulfonate **5c** at pH 6.5*

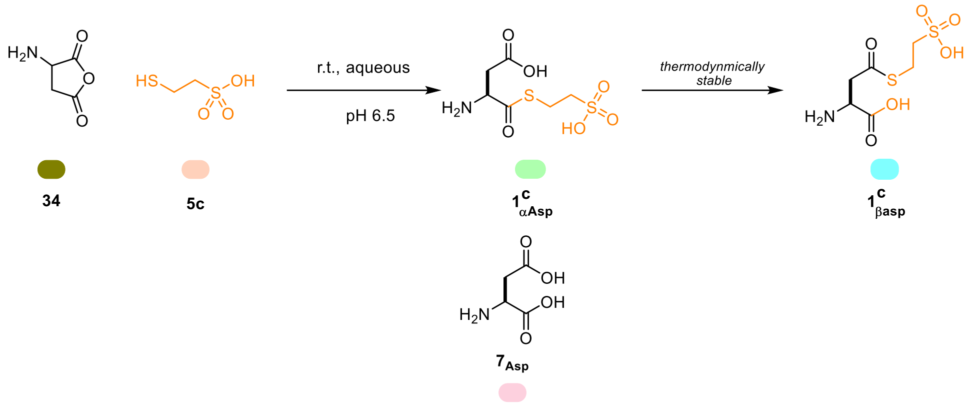

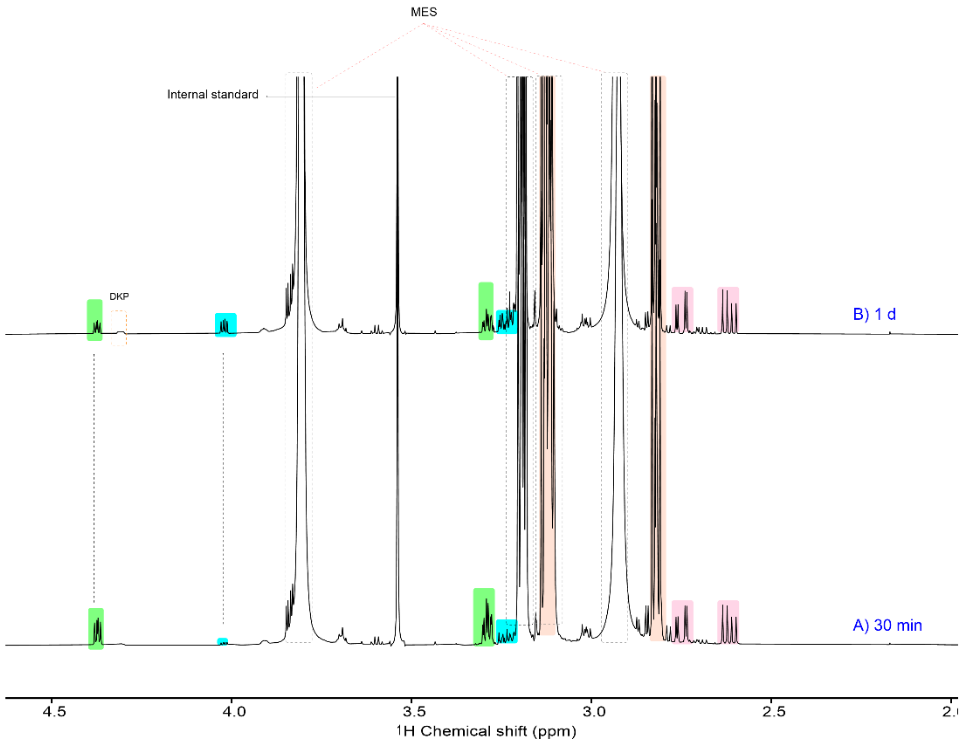

*Supplementary Figure 165: ^{1}H NMR (700 MHz, H_2O/D_2O 9:1, noesygppr1d, 1.4–5.0 ppm) spectra to show the reaction of aspartic anhydride (**34_{Asp}**, 20 mM) with 2-mercaptoethanesulfonate (**5c**, 100 mM) in 200 mM MES buffer, with PET (10 mM) as an internal standard at pH 6.5. NMR trace bottom to top: **A**) after 30 min formation of **$1^c_{\alpha Asp}$** from of the reaction of **34_{Asp}** with **5c** at pH 6.5 B) After 1 day **$1^c_{\alpha Asp}$** converting to **$1^c_{\beta Asp}$** (the ratio of a:β thioester 1:1.2).*

^{1}H NMR (600 MHz, H_2O/D_2O 9:1) **$1^c_{\alpha Asp}$** (partial assignment) : δ_H 4.43 (1H, dd, *J* = 6.6, 4.7 Hz, Asp-α-C*H*COSCH$_2$).

^{1}H NMR (600 MHz, H_2O/D_2O 9:1) **$1^c_{\beta Asp}$** (partial assignment) : δ_H 4.08 (1H, dd, *J* = 7.5, 4.4 Hz, Asp-β-*CH*CHHCOSCH$_2$).

DKP, **6_{AspAsp}**: δ_H 4.37 (1H, dd, *J* = 6.6, 4.7 Hz, Asp-α-C*H*CONH).

7_{Asp}: δ_H 2.68 (1H, dd, *J* = 17.5, 8.8 Hz, Asp-α-CHCH*H*COOH).

Entry	Time (h)	$\mathbf{1}^{c}_{\alpha Asp}$ (%)	$\mathbf{1}^{c}_{\beta Asp}$ (%)	$\mathbf{7}_{Asp}$ (%)	$\mathbf{6}_{AspAsp}$ (%)
1	0.5	45	3.2	41	<2
2	24	24	29	43	6

*Supplementary Table 38: Yields (%) for the reaction of aspartic anhydride (**34_{Asp}**, 20 mM), 20 mM) with 2-mercaptoethanesulfonate (**5c**, 100 mM) in MES buffer (200 mM) with PET (10 mM) as an internal standard at pH 6.5.*

*Formation of thioester **1_{Ala}** upon incubation of aminoacyl adenylate **2_{Ala}** with 3-mercaptopropionic acid **5b***

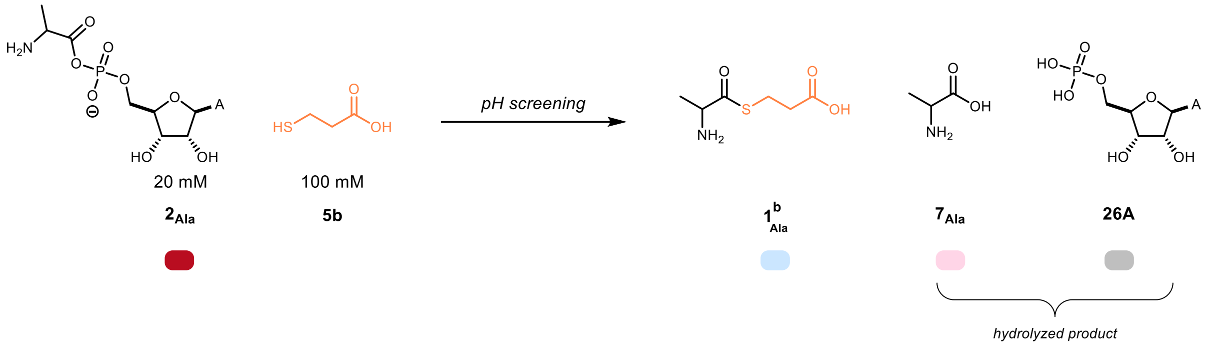

Entry	Buffer	pH	1^{b}_{Ala} (%)
1	Citric acid	2	4
2	Acetic acid	3	10
3	Acetic acid	4	23
4	Acetic acid	5	36
5	MES	6	42

*Supplementary Table 39. Yield (%) of thioester **1^{b}_{Ala}** formed from the reaction of aminoacyl adenylate **2_{Ala}** (20 mM) with 3-mercaptopropionic acid (**5b**, 100 mM) in the specified buffer (200 mM). Set up following General Procedure I.*

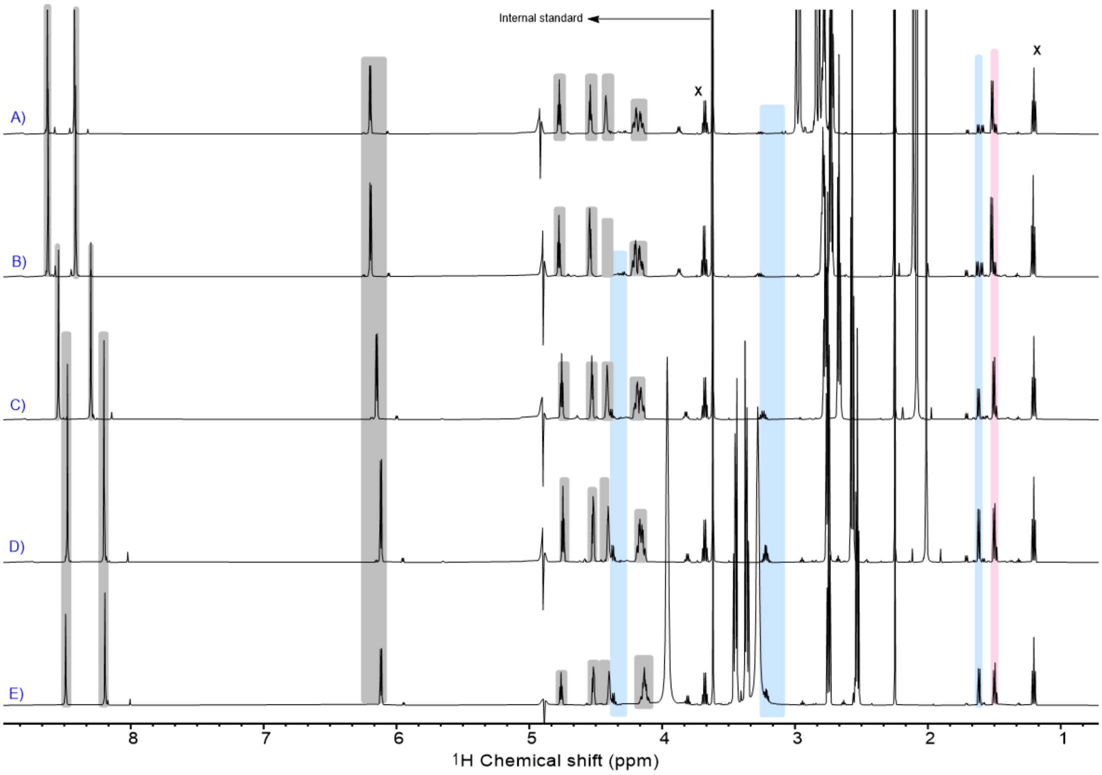

*Supplementary Figure 166. ^{1}H NMR (600 MHz, H_2O/D_2O 9:1, noesygppr1d, 1.0 – 8.5 ppm) spectra to show the reaction of aminoacyl adenylate **2_{Ala}** (20 mM) with 3-mercaptopropionic acid (**5b**, 100 mM), at the specified pH, with PET (10 mM) as an internal standard, after 12 hours: **A**) pH 2, citric acid (200 mM); **B**) pH 3, citric acid (200 mM); **C**) pH 4, acetic acid (200 mM); **D**) pH 5, acetic acid (200 mM); **E**) pH 6, MES (200 mM). **2_{Ala}** was prepared following the protocol of Berg.[12] **2_{Ala}** was precipitated from ethanol and used without further purification. X = ethanol.*

Preparation of synthetic Aminoacyl thioester **$L\text{-}1^{n}_{Aaa}$**

General Procedure for synthetic amino aminoacyl thioester

All glassware was flame-dried under vacuum and cooled under argon three times prior to the commencement of the reaction. Boc-L-amino acid (10 mmol, 1 eq.) was dissolved in anhydrous MeCN (20 mL) under an argon atmosphere. HATU (10 mmol, 1 eq.) was added, followed by the addition of DIPEA (23 mmol, 2.3 eq.). Ethanethiol (30 mmol, 3 eq.) was then carefully added to the reaction mixture, which was stirred for 4−16 hours at room temperature. After 4 or 16 hours, ethanethiol was removed under a flow of argon, and quenched through a bleach solution (sodium hypochlorite: H_2O, 1:1, 50 mL) for 20 minutes. The reaction mixture was then concentrated under reduced pressure, and the resulting crude product was directly purified by column chromatography (eluting with a petroleum ether and ethyl acetate gradient of 100:0 → 0:100).

The Boc-protected L-aminoacyl thioester (8 mmol) was dissolved in 2M HCl in diethyl ether (20 mL) under an argon atmosphere and stirred at room temperature for 16 hours (until white precipitates formed). The precipitate was filtered and washed with a mixture of diethyl ether and petroleum ether (1:1, 10 mL:10 mL). The resulting white solid was further dried overnight under high vacuum, and the quality was checked by ^{1}H and ^{13}C NMR. The data were consistent with reported literature [3], and all yields are listed in the table below.

The following protected aminoacyl thioesters — Boc-Gln(Trt)-SEt, Boc-Arg(Pbf)-SEt, Boc-Ser(tBu)-SEt, Boc-Glu(tBu)-SEt (1 mmol)—were treated with freshly opened TFA (5 mL) under an argon atmosphere and stirred for 30 minutes. The reaction mixture was concentrated under reduced pressure, followed by evaporation with toluene (10 mL). The resulting oily product was dried overnight under high vacuum, and the product was further confirmed by NMR and Mass analysis.

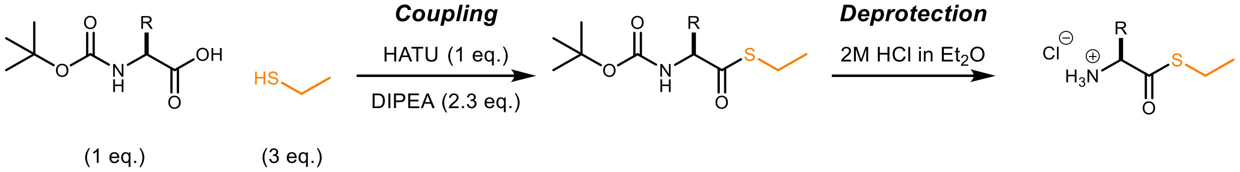

Entry	Starting material 'R'	Thioester ($L\text{-}1^{e}_{Aaa}$) (%)	HRMS-ESI for thioester $L\text{-}1^{e}_{Aaa}$ Formula	Theoretical	Found
1	Boc-Gly-OH	74	$C_4H_{10}NOS$ [M+H]$^+$	120.0478	120.0475
2	Boc-L-Ala-OH	77	$C_5H_{12}NOS$ [M+H]$^+$	134.0634	134.0637
3	Boc-L-Val-OH	63	$C_8H_{18}NOS$ [M+H]+	176.1104	176.1108
4	Boc-L-Phe-OH	85	$C_{11}H_{16}NOS$ [M+H]$^+$	210.0947	210.0947
5	(Boc)-L-Lys(boc)-OH	55	$C_8H_{19}N_2OS$ [M+H]$^+$	191.1213	191.1212
6	Boc-L-Leu-OH	67	$C_8H_{18}NOS$ [M+H]$^+$	176.1104	176.1104
7	Boc-L-His(boc)-OH	59	$C_8H_{18}N_3OS$ [M+H]$^+$	200.0852	200.0852
8	Boc-L-Pro-OH	65	$C_7H_{14}NOS$ [M+H]$^+$	160.0791	160.0790
9	Boc-L-Glu(tbu)-OH	60	$C_7H_{14}NO_3S$ [M+H]$^+$	192.0689	192.0688
10	Boc-L-Gln(Trt)-OH	42	$C_7H_{15}N_2O_2S$ [M+H]$^+$	191.0849	191.0848
11	Boc-L-Arg(Pbf)-OH	35	$C_8H_{19}N_4OS$ [M+H]$^+$	219.1274	219.1270
12	Boc-L-Ser(tbu)-OH	39	$C_5H_{12}NO_2S$ [M+H]$^+$	150.0583	150.0581

Supplementary Table 40: Yield (%) of aminoacyl thioester over two steps and HRMS data for the aminoacyl thioester $\mathbf{1^{e}_{Aa}}$. *The data is consistent with reported literature.*[3]

Valine thioester L-$\mathbf{1^{e}_{Val}}$

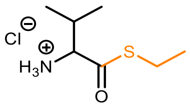

^{1}H NMR (700 MHz, D_2O) δ_H 4.20 (d, J = 4.8 Hz, 1H, α-C*H*CH(CH_3)$_2$), 3.02 – 2.89 (m, 2H, -SC*H*$_2$$CH_3$), 2.40 (m, 1H, β-CHC*H*(CH_3)$_2$), 1.27 (t, J = 7.4 Hz, 3H, -SCH_2C*H*$_3$), 1.03 (d, J = 7.0 Hz, 3H, β-CHCHC*H*$_3$$CH_3$), 0.95 (d, J = 7.0 Hz, 3H, -CHCHCH_3C*H*$_3$). **^{13}C NMR** (176 MHz, D_2O) δ_C 199.9, 64.9, 30.7, 24.3, 18.3, 16.6, 14.1. The data is consistent with reported in literature.[3]

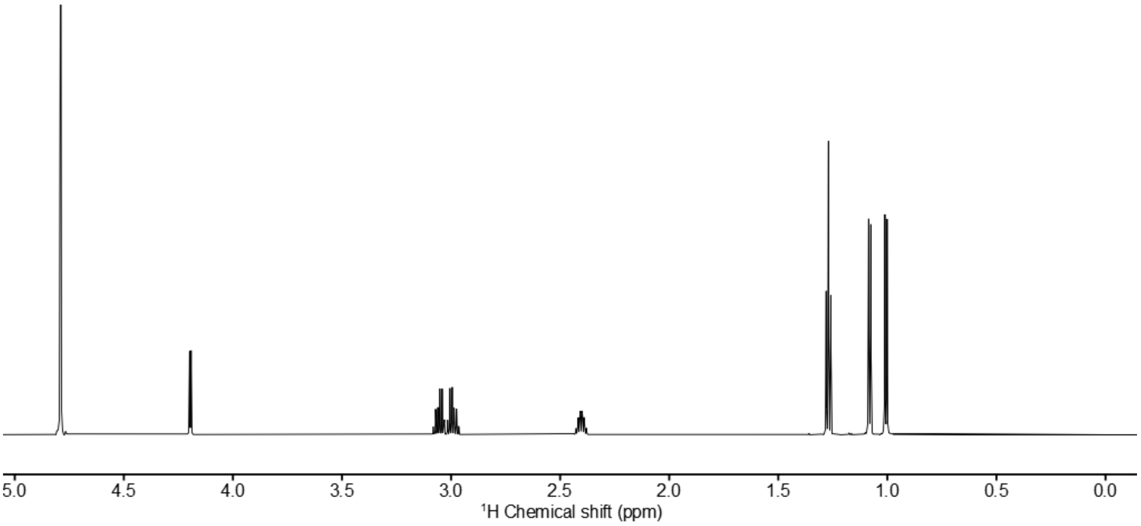

Supplementary Figure 167: ^{1}H NMR (700 MHz, D_2O, 1.0 – 5.5 ppm) spectrum of Valine thioester L-1^{e}_{Val}.

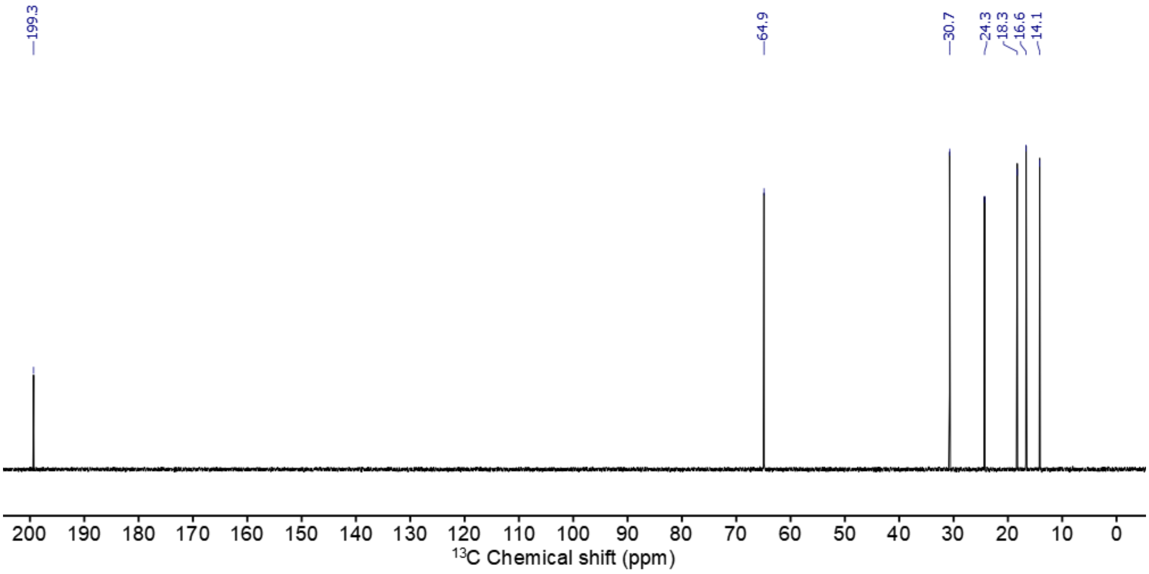

Supplementary Figure 168: ^{13}C NMR (176 MHz, D_2O, 0 – 200 ppm) spectrum of Valine thioester L-1^{e}_{Val}.

Leucine thioester L-$\mathbf{1^{e}_{Leu}}$

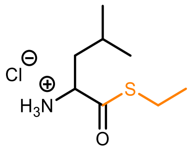

^{1}H NMR (700 MHz, D_2O)δ_H 4.30 (dd, *J* = 8.2, 6.1 Hz, 1H, α-C*H*$CH_2CH(CH_3)_2$), 3.22 – 2.91 (m, 2H, -SC$H_2$$CH_3$), 1.92 – 1.83 (m, 1H, α-CHC*H*HCH$(CH_3)_2$), 1.82 – 1.87 (m, 1H, α-CHCH_2C*H*$(CH_3)_2$), 1.77 – 1.70 (m, 1H, α-CHCH*H*CH$(CH_3)_2$), 1.27 (t, *J* = 7.4 Hz, 3H, -SCH_2CH_3), 1.00 (d, *J* = 6.4 Hz, 3H, -CHC$H_3$$CH_3$), 0.98 (d, *J* = 6.4 Hz, 3H, -CHCH_3CH_3). **^{13}C NMR** (176 MHz, D_2O). δ_C 199.0, 57.2, 39.7, 23.6, 23.3, 21.3, 20.5, 13.1. The data is consistent with reported in literature.[3]

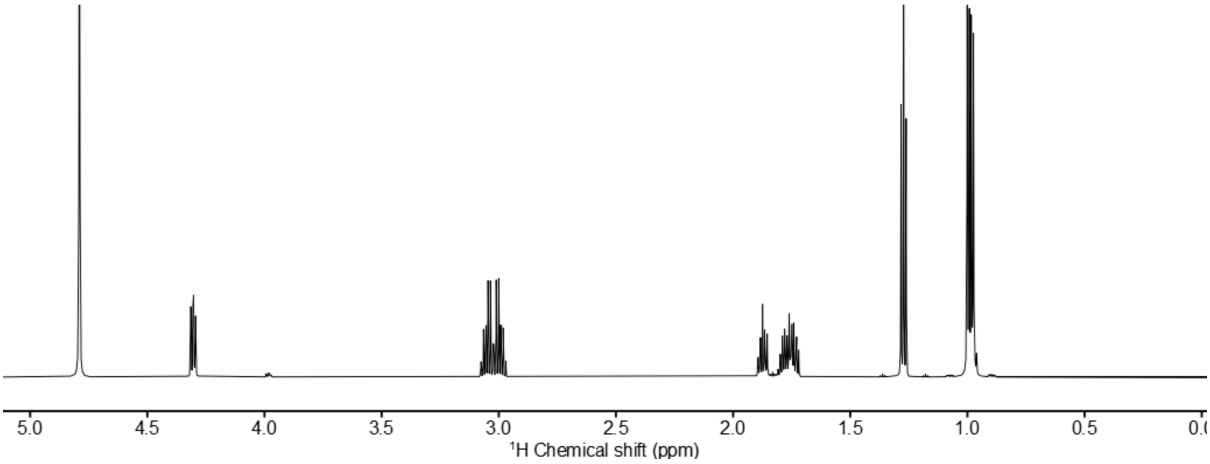

Supplementary Figure 169: ^{1}H NMR (700 MHz, D_2O, 0 – 5.0 ppm) spectrum of Leucine thioester L-1^{e}_{Leu}.

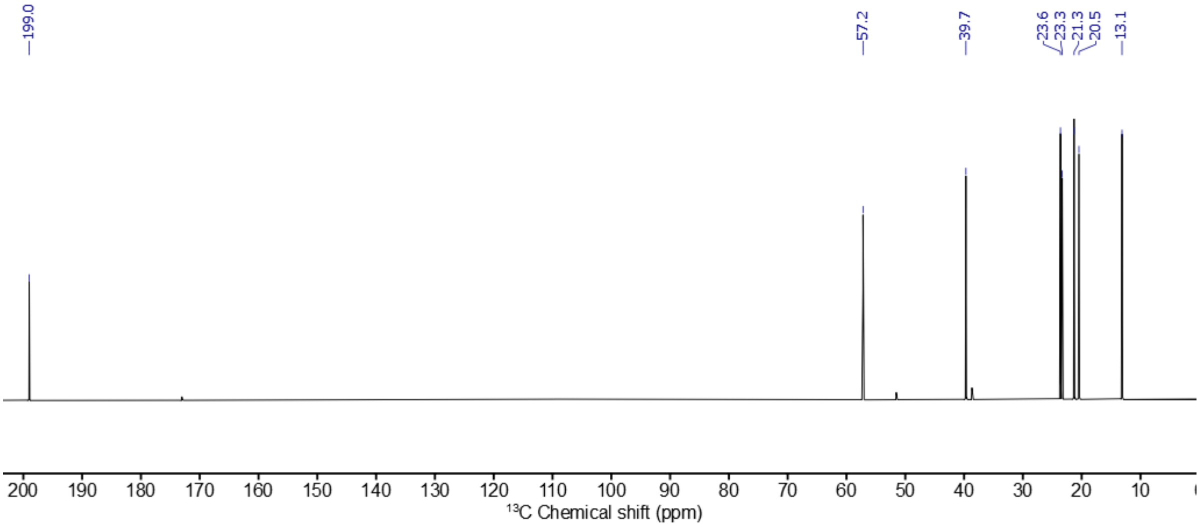

Supplementary Figure 170: ^{13}C NMR (176 MHz, D_2O, 0 – 200 ppm) spectrum of Leucine thioester L-1^{e}_{Leu}.

Glutamic thioester L-$\mathbf{1}^{\mathbf{e}}_{\mathbf{Glu}}$

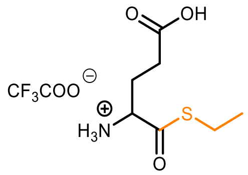

¹H NMR (700 MHz, D_2O)δ_H 4.36 (t, J = 6.5 Hz, 1H, α-CHCH$_2$), 3.12 – 2.93 (m, 2H, -SCH_2CH$_3$), 2.61 (td, J = 7.2, 1.2 Hz, 2H, α-CHCH$_2$CH_2COOH), 2.29 (dtd, J = 14.7, 7.3, 6.4 Hz, 1H, α-CHCHHCH$_2$COOH), 2.20 (ddd, J = 14.8, 13.9, 7.1 Hz, 1H, α-CHCHHCH$_2$COOH), 1.26 (t, J = 7.4 Hz, 3H, -SCH$_2$CH_3). **¹³C NMR** (176 MHz, D_2O)δ_C 198.8, 176.6, 58.7, 29.6, 26.5, 24.4, 13.9.

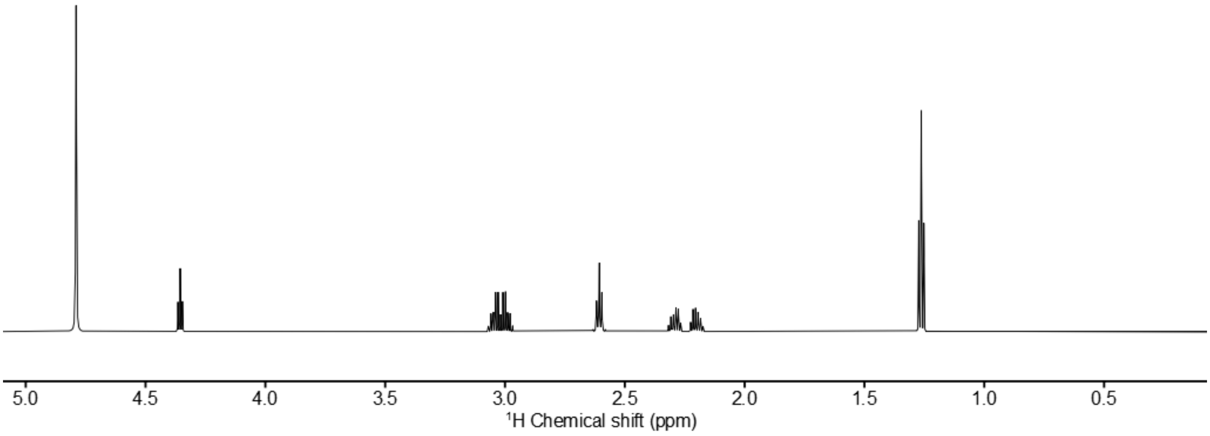

Supplementary Figure 171: ¹H NMR (700 MHz, D_2O, 0 – 5.5 ppm) spectrum of Glutamic thioester L-$\mathbf{1}^{e}_{Glu}$.

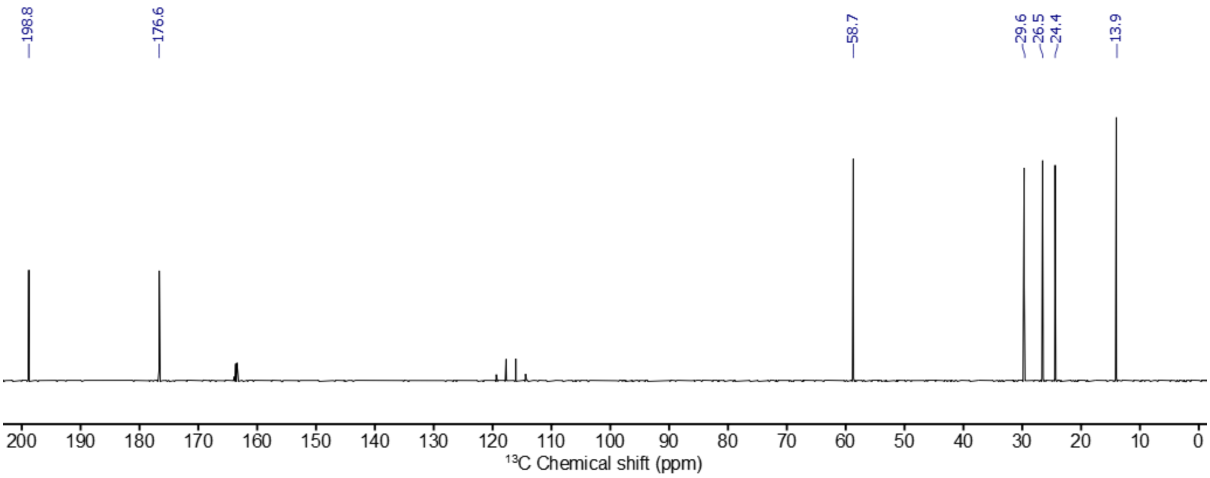

Supplementary Figure 172: ¹³C NMR (176 MHz, D_2O, 0 – 200 ppm) spectrum of Glutamic thioester L-$\mathbf{1}^{e}_{Glu}$.

Glutamine thioester L-1^{e}_{Gln}

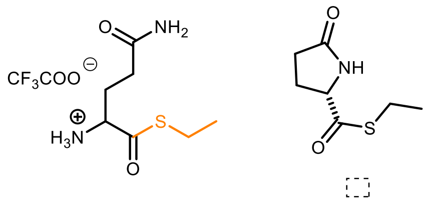

^{1}H NMR (600 MHz, H_2O/D_2O)δ_H 4.19 (t, *J* = 6.4 Hz, 1H, α-C*H*CH_2), 2.94 – 2.81 (m, 2H, -SC*H*$_2$$CH_3$), 2.43 – 2.31 (m, 2H, α-C*H*CH_2C*H*$_2$$CONH_2$), 2.19 – 2.10 (m, 1H, α-CHC*H*HC$H_2$$CONH_2$), 2.10 – 2.01 (m, 1H, α-CHCH*H*C$H_2$$CONH_2$), 1.12 (t, *J* = 7.4 Hz, 3H, -SCH_2C*H*$_3$). **^{13}C NMR** (176 MHz, H_2O/D_2O)δ_C 198.8, 177.4, 58.9, 30.6, 27.1, 24.5, 14.1.

A side product was observed during the deprotection of Boc-Gln(Trt)-thioester.

Reported as Pyroglutamate L-$1^{e}_{PyroGln}$: **^{1}H NMR** (600 MHz, H_2O/D_2O)δ_H 4.32 (dd, *J* = 9.5, 3.7 Hz, 1H, α-C*H*CH_2), 2.77 (q, *J* = 7.4 Hz, 2H, -SC*H*$_2$$CH_3$), 2.48 – 2.38 (m, 2H, α-C*H*CH_2C*H*$_2$$CONH_2$), 2.29 – 2.19 (m, 1H, α-CHCH*H*C$H_2$$CONH_2$), 2.01 – 1.93 (m, 1H, α-CHC*H*HC$H_2$$CONH_2$), 1.08 (t, *J* = 7.4 Hz, 3H, -SCH_2C*H*$_3$).

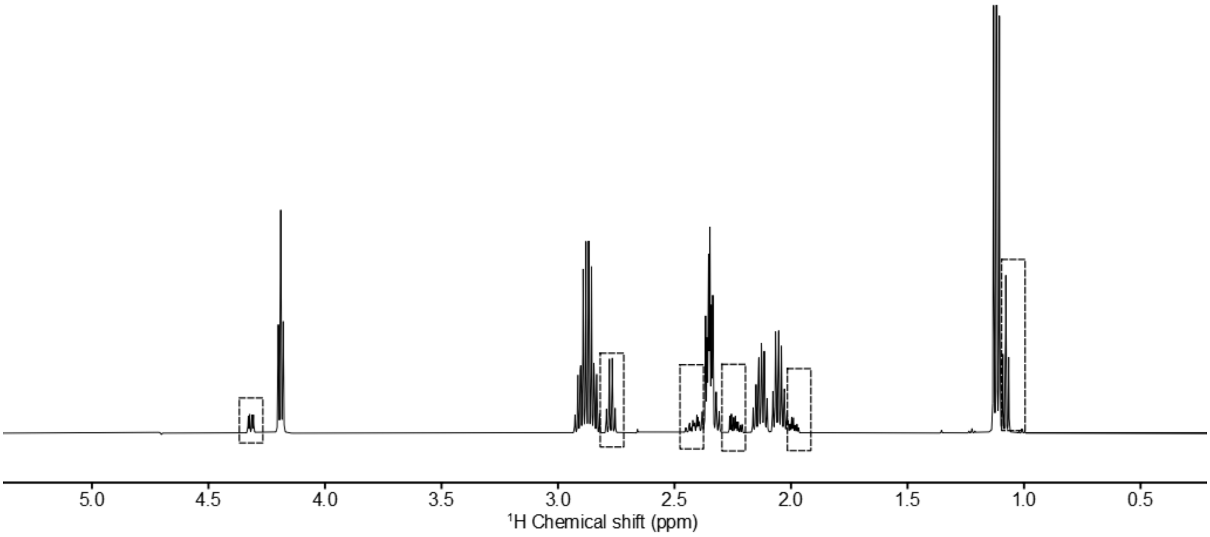

Supplementary Figure 173: ^{1}H NMR (700 MHz, D_2O, 0 – 5.5 ppm) spectrum of Glutamine thioester L-1^{e}_{Gln}

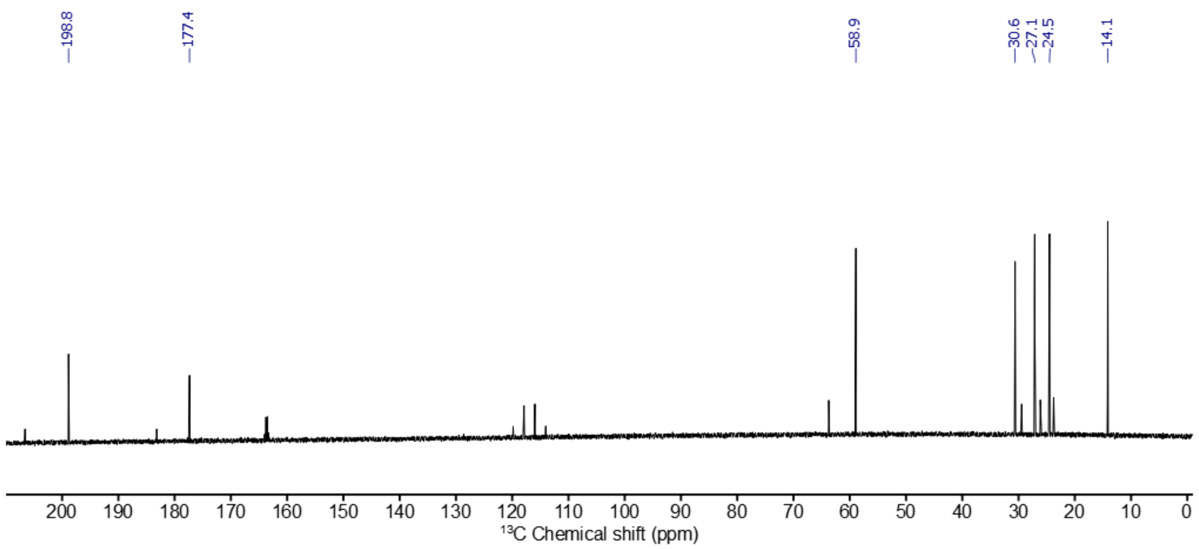

Supplementary Figure 174: ^{13}C NMR (176 MHz, D_2O, 0 – 200 ppm) spectrum of Glutamine thioester L-$\mathbf{1}^{e}_{Gln}$.

Lysine thioester L-$\mathbf{1}^{e}_{Lys}$

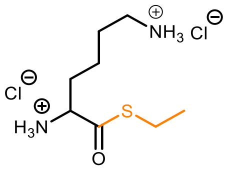

^{1}H NMR (700 MHz, D_2O)δ_H 4.29 (t, J = 6.2 Hz, 1H, α-CHCH$_2$), 3.11 – 2.95 (overlapped, 4H, ε-CH_2NH$_2$, -SCH_2CH$_3$), 2.11 – 2.00 (m, 1H, β-CHCHH), 1.99 – 1.91 (m, 1H, β-CHCHH), 1.77 – 1.68 (m, 2H, δ-CHCH$_2$CH_2), 1.59 – 1.39 (m, 2H, γ-CHCH$_2$CH$_2$CH_2CH$_2$NH$_2$), 1.27 (t, J = 7.4 Hz, 3H, -SCH$_2$CH_3). **^{13}C NMR** (176 MHz, H_2O/D_2O)δ_C 199.7, 59.4, 39.5, 31.2, 26.9, 24.3, 21.8, 14.1.

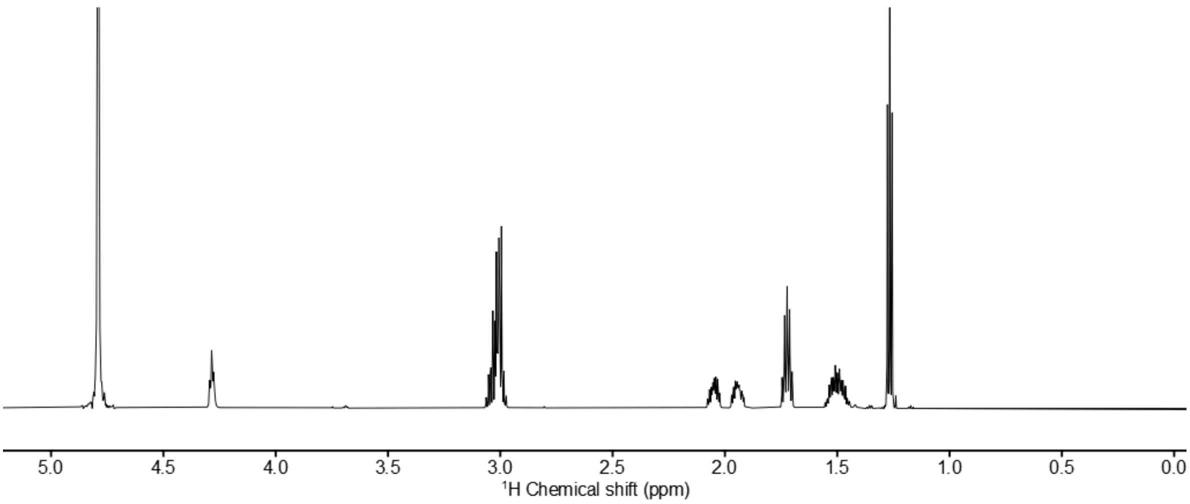

Supplementary Figure 175: ^{1}H NMR (700 MHz, D_2O, 0 – 5.0 ppm) spectrum of Lysine thioester L-$\mathbf{1}^{e}_{Lys}$.

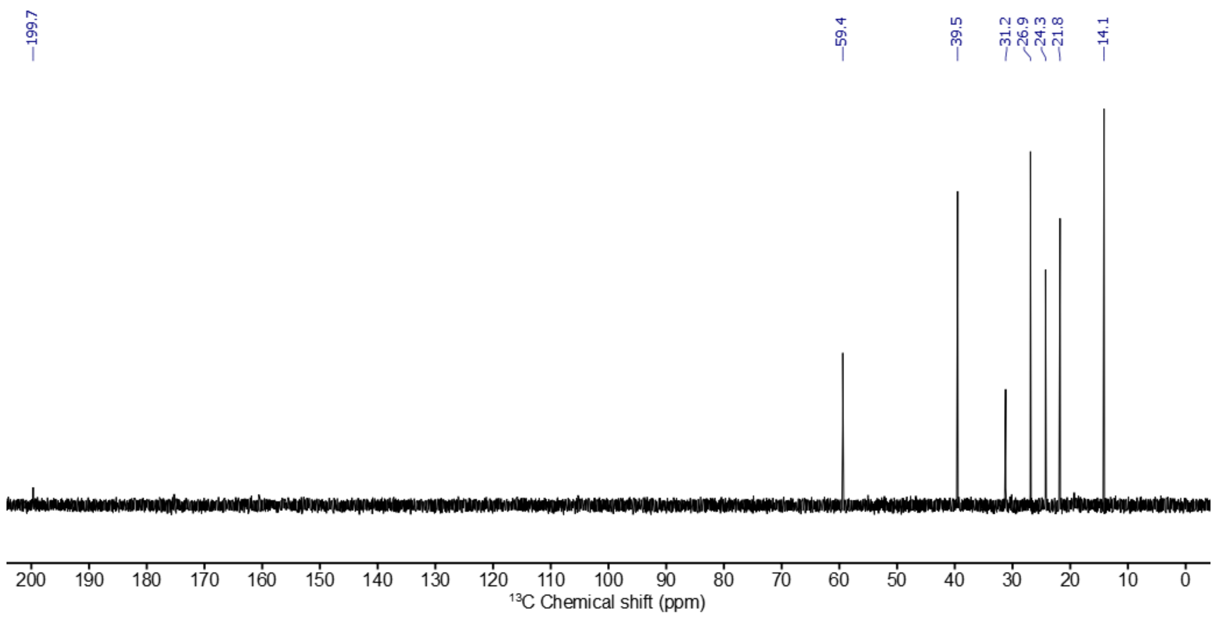

Supplementary Figure 176: ¹³C NMR (176 MHz, D_2O, 0 – 200 ppm) spectrum of Lysine thioester L-$\mathbf{1}^{e}_{Lys}$.

Arginine thioester L-$\mathbf{1}^{\mathbf{e}}_{\mathbf{Arg}}$

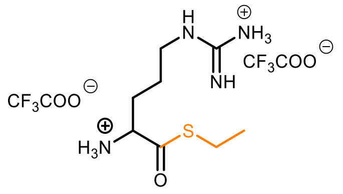

¹H NMR (700 MHz, D_2O)δ_H: 4.36 (t, J = 6.2 Hz, 1H, α-C*H*CH$_2$), 3.36 – 3.17 (m, 2H, δ-C*H*$_2$NH), 3.12 – 2.90 (m, 2H, -SC*H*$_2$CH$_3$), 2.14 – 2.04 (m, 1H, β-CHC*H*H), 2.04 – 1.94 (m, 1H, β-CHCH*H*), 1.79 – 1.71 (m, 1H, γ-C*H*HCH$_2$NH), 1.71 – 1.61 (m, 1H, γ-CH*H*CH$_2$NH), 1.27 (t, J = 7.4 Hz, 3H, -SCH$_2$C*H*$_3$). **¹³C NMR** (176 MHz, H_2O/D_2O)δ_C: 199.1, 157.3, 59.2, 40.7, 28.6, 24.4, 23.9, 14.2.

Note: A side product, tetramethylurea from HATU (labelled as 'X' in the NMR and carried over from the first step), was observed. The side chain of was protected with Pbf-OH, which was removed with TFA but remained as a counter-ion. Specifically, in the case of the Arg-thioester **L-$\mathbf{1}^{\mathbf{e}}_{\mathbf{Arg}}$**, after treatment with TFA, the product was directly placed on a Q-Sepharose strong ion exchange column (12 g) to remove Pbf-OH. Arg-SEt was then eluted with water, directly in the injection peak as a pure, charged arginine thioester **L-$\mathbf{1}^{\mathbf{e}}_{\mathbf{Arg}}$** (the product was used without further purification).

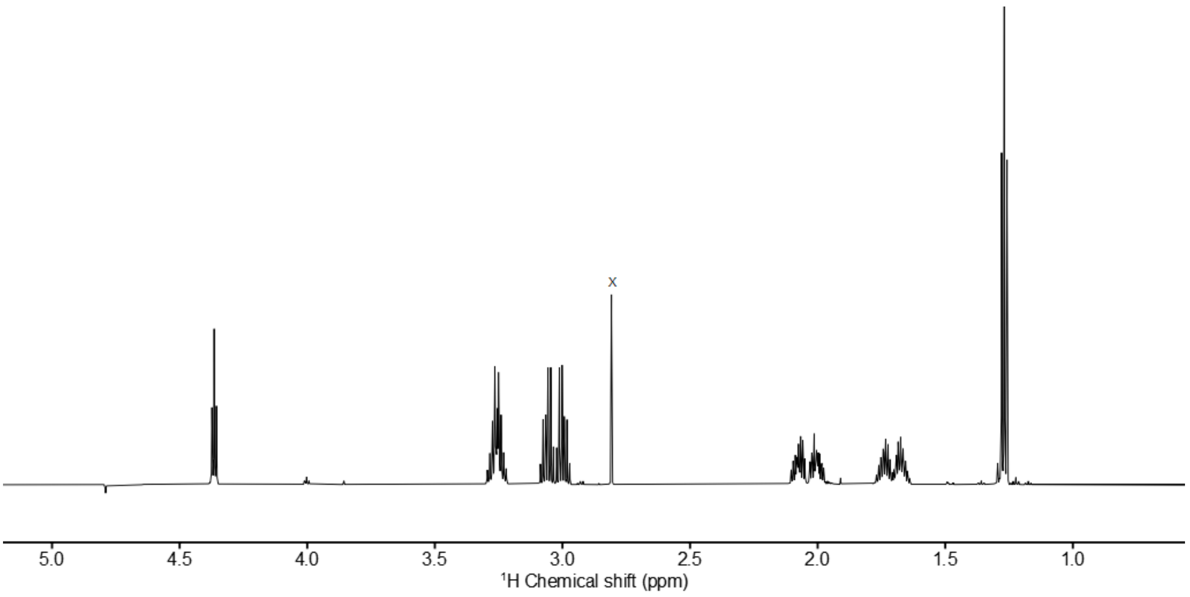

Supplementary Figure 177: ^{1}H NMR (700 MHz, D_2O, 0 – 5.0 ppm) spectrum of Arginine thioester L-$\boldsymbol{1^{e}_{Arg}}$.

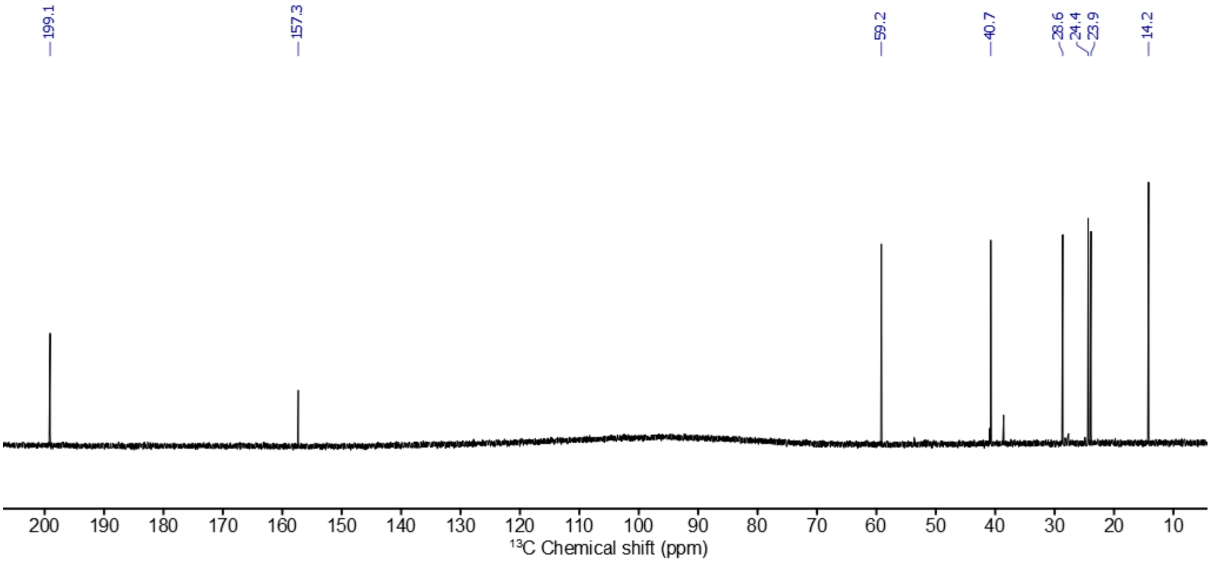

Supplementary Figure 178: ^{13}C NMR (176 MHz, D_2O, 0 – 200 ppm) spectrum of Arginine thioester L-$\boldsymbol{1^{e}_{Arg}}$.

Serine thioester L-$\mathbf{1^{e}_{Ser}}$

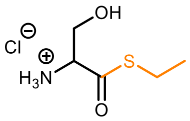

^{1}H NMR (700 MHz, D_2O)δ_H: 4.44 (t, J = 4.0 Hz, 1H, α-C*H*CH$_2$OH), 4.10 (qd, J = 12.8, 4.0 Hz, 2H, α-CHC*H*$_2$OH), 3.03 (q, J = 7.4 Hz, 2H, -SC*H*$_2$CH$_3$), 1.27 (t, J = 7.4 Hz, 3H, -SCH$_2$C*H*$_3$). δ_C 199.0, 57.2, 39.7, 23.6, 23.3, 21.3, 20.5, 13.1. **^{13}C NMR** (176 MHz, D_2O)δ_C: 197.1, 61.3, 60.9, 24.3, 14.1.

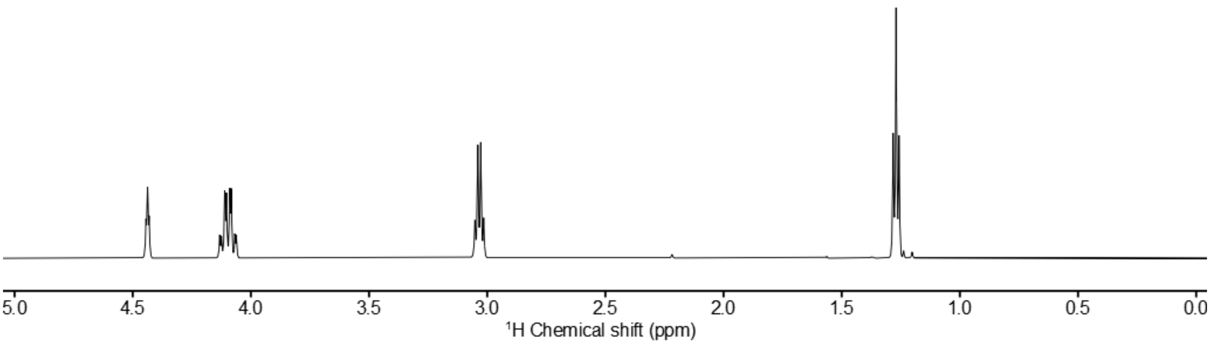

Supplementary Figure 179: ^{1}H NMR (700 MHz, D_2O, 1.0 – 5.5 ppm) spectrum of Serine thioester L-$\mathbf{1}^{e}_{Ser}$.

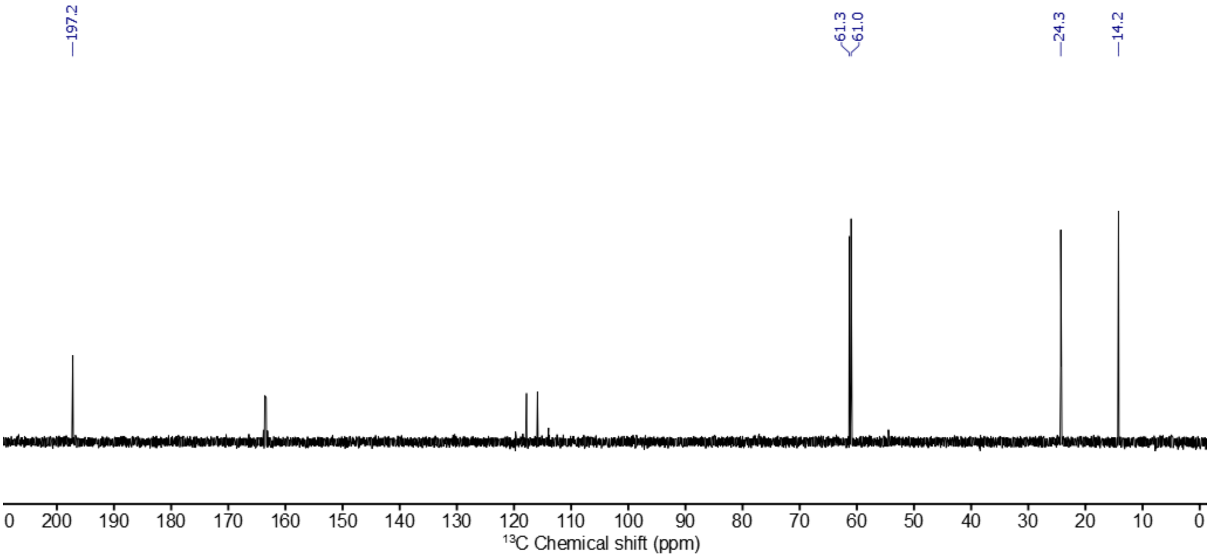

Supplementary Figure 180: ^{13}C NMR (176 MHz, D_2O, 0 – 200 ppm) spectrum of Serine thioester L-$\mathbf{1}^{e}_{Ser}$.

Histidine thioester L-$\mathbf{1}^{e}_{His}$

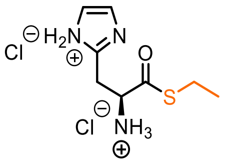

^{1}H NMR (700 MHz, D_2O)δ_H: 8.75 (d, *J* = 1.4 Hz, 1H, imidazole -NC*H*NH), 7.49 (d, *J* = 1.3 Hz, 1H, imidazole -NHC*H*),), 4.63 (t, *J* = 7.2 Hz, 1H, α-C*H*CH$_2$), 3.55 – 3.42 (m, 2H, α-CHC*H*$_2$), 3.10 – 2.91 (m, 2H, -SC*H*$_2$CH$_3$), 1.22 (t, *J* = 7.4 Hz, 3H, -SCH$_2$C*H*$_3$). **^{13}C NMR** (176 MHz, D_2O)δ_C: 197.5, 134.9, 126.2, 119.3, 58.3, 27.0, 24.6, 13.9.

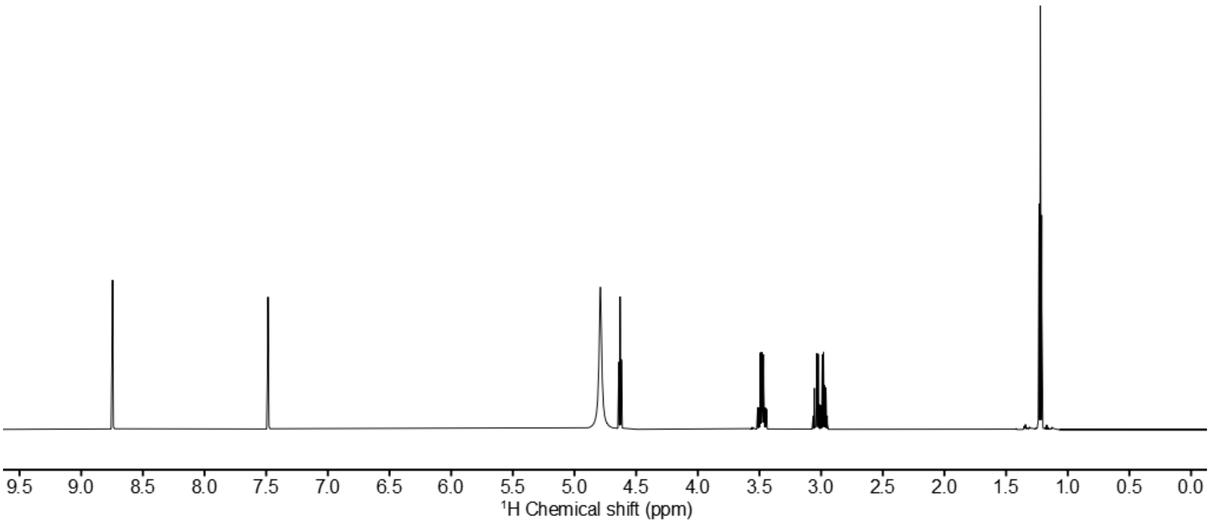

Supplementary Figure 181: ^{1}H NMR (700 MHz, D_2O, 0 – 5.5 ppm) spectrum of Histidine thioester L-$\mathbf{1^{e}_{His}}$.

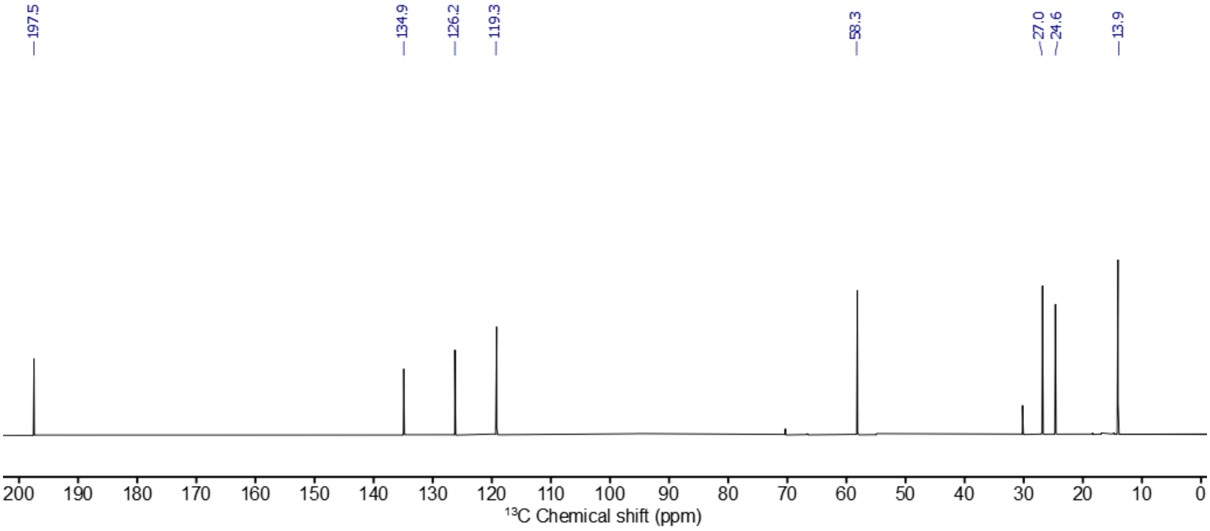

Supplementary Figure 182: ^{13}C NMR (176 MHz, D_2O, 0 – 200 ppm) spectrum of Histidine thioester L-$\mathbf{1^{e}_{His}}$.

Supplementary Discussion

Observed pathways of aminonitrile reaction with thiols

Under neutral and alkaline conditions thiols (**5**) catalyse aminonitrile (**8**) hydration and dimerization.[13,14] Dimerisation forms amidine **54** that cyclises to amino-imidazole (**54**) (Supplementary Figure 181, Path A). For example, at pH 7 – 9, the reaction of $\mathbf{8_{Ala}}$ and thiol **5b** was observed to yield amide $\mathbf{10_{Ala}}$ and imidazole $\mathbf{55_{AlaAla}}$ (Supplementary Table 41, 42 & Supplementary Figure 185). Amino-imidazole (**55**), especially those derived from $\mathbf{8_{Gly}}$, bear a close structure relationship to purines, intermediates in the de novo pathway for purine biosynthesis, and proposed prebiotic purine precursors,[15,16,17,18,19] but further investigation of **55** is beyond the scope of this work. However, at pH 5, alanine thioester $\mathbf{1_{Ala}}$ was formed in 20% yield, alongside amino-imidazole $\mathbf{55_{AlaAla}}$ (Supplementary Table 42), and, at pH 3, selective formation of thioester **1** was observed, with $\mathbf{1_{Ala}}$ formed in 48% yield as the major product (Supplementary Figure 183, Path B), alongside alanine amide $\mathbf{10_{Ala}}$ (8%) (Supplementary Table 2, Entry 3 & Supplementary Figure 183, Path C). Aminonitriles (**8**) were also observed to yield α,β-keto-aldehyde **58** (Supplementary Figure 181, Path D), which are observed in ^{1}H NMR spectra as thioaminal (or thiohemiacetal) **57** and then hydrate **58**•H_2O with characteristic 5 – 6 p.p.m. (singlet) ^{1}H NMR resonances. For most aminonitriles (**8**) studied this α, β-keto-aldehyde pathway (Path D) was a very minor pathway (<7%). For example, $\mathbf{8_{Ala}}$ yielded only 1% $\mathbf{58_{Ala}}$•H_2O (Supplementary Figure 185). The structure of $\mathbf{58_{Ala}}$ was confirmed by sample spiking (with commercial methylglyoxal $\mathbf{58_{Ala}}$) and augmentation of the 5.57 p.p.m. singlet resonance. However, for phenylalanine nitrile $\mathbf{8_{Phe}}$ and valine nitrile $\mathbf{8_{Val}}$ (see Supplementary Figure 204, 208), the keto-aldehyde was a major pathway that limited the yield of these thioester **1** (from their respective aminonitrile **8**). The phenyl-α,β-keto-aldehyde $\mathbf{58_{Phe}}$, likely due to its stabilised (conjugated) enol-tautomer, undergoes further reactions, resulting in accumulation of a dense, yellow oil. This material was a mixture of products; analysis of crude NMR spectra (in DMSO-d_6) indicated this oil was not amide, acid or thioester material (based on ^{1}H, ^{13}C and HMBC-spectra). These water-insoluble by-products have not been purified or further characterised. α,β-Dicarbonyls are important intermediates in biosynthesis (e.g. Krebs-cycle, triose glycolysis) and have been widely exploited in prebiotic chemistry,[20,21,22] however further investigation of this pathway is beyond the scope of this work. The reactions of valine nitrile $\mathbf{8_{Val}}$ and phenylalanine nitrile $\mathbf{8_{Phe}}$ have not been further optimised for thioester synthesis. However, both Val-thioester $\mathbf{1_{Val}}$ and Phe-thioester $\mathbf{1_{Phe}}$ were synthesised in near-quantitative (>95%) yield from their respective NCAs $\mathbf{4_{Val}}$ and $\mathbf{4_{Phe}}$ upon reaction with coenzyme M (**5c**) in water (see main text, and extended data figure 3, Entry 10 & 11).

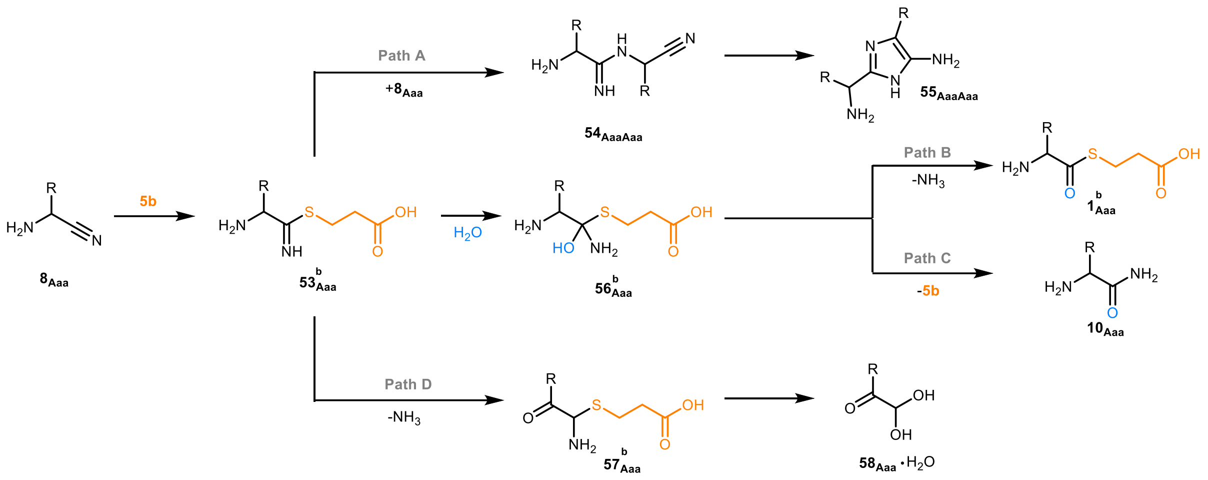

*Supplementary Figure 183: Proposed reaction pathways of aminonitriles **8** with thiols **5** depicted for 3-mercaptopropionic acid (**5b**). Path A: Thiol-catalysed **2**-dimerisation to amidine **54** followed by cyclisation to imidazole **55**. Path B: Hydrolysis of thioimidate **53** with loss of ammonia via tetrahedral intermediate **56** to yield amino thioester **1**; Path C: Hydrolysis of thioimidate **53** with loss of **5b** to yield amino amide **10**; Path D: Hydride-shift or tautomerisation of thioimidate **53** to thiohemiaminal **57** followed by hydrolysis to* α, β*-keto-aldehyde **58** (shown, as observed, as hydrate **58**.*H_2O*).*

Formation of aminoacyl thiol $\mathbf{1}_{\mathbf{Aaa}}$

*General Procedure K – Formation of aminoacyl thiols **1** by the reaction of thiols **5** and aminonitriles **8***

α-Aminonitrile ($\mathbf{8}_{\mathbf{Aaa}}$, 100 μmol), thiol (**5**, 900 μmol), and methylsulfonylmethane (MSM, 50 μmol) or pentaerythritol (PET, 10 μmol), with or without sodium dihydrogen phosphate (500 μmol), were dissolved in degassed H_2O/D_2O (98:2, 0.3 mL). The solution was adjusted to the desired pH with NaOH/HCl, and the final volume set to 0.5 mL with degassed H_2O/D_2O (98:2). The solution was incubated at room temperature, and NMR spectra were periodically acquired until complete consumption of $\mathbf{8}_{\mathbf{Aaa}}$ was observed. The structure of α-aminoacyl-thioester $\mathbf{1}^{n}_{\mathbf{Aaa}}$ was confirmed by ^{1}H–^{13}C HMBC NMR analysis and spiking with pure synthetic standards where available. The reactions were quantified using MSM or PET as an internal standard.

*Reaction of thiol **5** with alanine nitrile $\mathbf{5}_{\mathbf{Ala}}$*

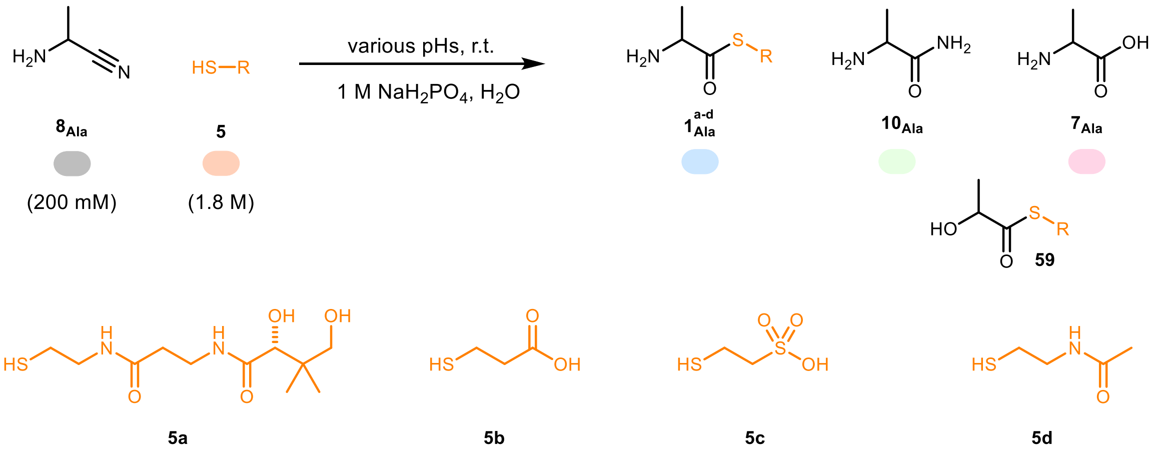

*Supplementary Figure 184. Formation of aminoacyl thiol $\mathbf{1}^{a\text{-}d}_{Ala}$ by reaction of alanine nitrile ($\mathbf{8}_{\mathbf{Ala}}$, 200 mM) and specified thiols (**5a–d**, 1.8 M) in 1 M NaH_2PO_4 buffer at pH 3 and room temperature.*

Entry	Thiol	Time (days)	pH	Buffer	Temp.	$\mathbf{7}_{\mathbf{Ala}}$ (%)	$\mathbf{10}_{\mathbf{Ala}}$ (%)	$\mathbf{1}_{\mathbf{Ala}}$ (%)	$\mathbf{59}_{\mathbf{Ala}}$ (%)
1	**5b**	1	7	-	r.t.	obs.	obs.	**n.d.**	-
2	**5b**	1	5	-	r.t.	3	9	**20**	-
3	**5b**	2	4		r.t.	5	11	**42**	-
3	**5b**	5	3	-	r.t.	11	8	**48**	-
4	**5b**	30	7	PB	-7 °C[a]	8	9	**48**	-
5	**5b**	5	3	PB	r.t.	24	14	**56**	4
6	**5c**	2	3	PB	r.t.	7	17	**52**	1
7	**5d**	2	3	PB	r.t.	6	13	**41**	-
8	**5a**	5	3	PB	r.t.	12	obs.	**39**	-

*Supplementary Table 41. Yield of aminoacyl-thioester $\mathbf{1}^{a\text{-}d}_{Ala}$ formed in the reaction of alanine nitrile ($\mathbf{8}_{\mathbf{Ala}}$, 200 mM) and thiols **5a-d** (1.8 M) at room temperature according to General Procedure K. r.t. = room temperature; n.d. = not detected; obs. = signal obscured by resonance overlap. PB = phosphate buffer. a = eutectic phase reaction, the solution was flash frozen at -80 °C and then incubated at -7 °C (see Supplementary Figure 195).*

Reaction of alanine nitrile ***8**$_{Ala}$* *with 3-mercaptopropanoic acid* ***5b*** *at specified pHs and room temperature*

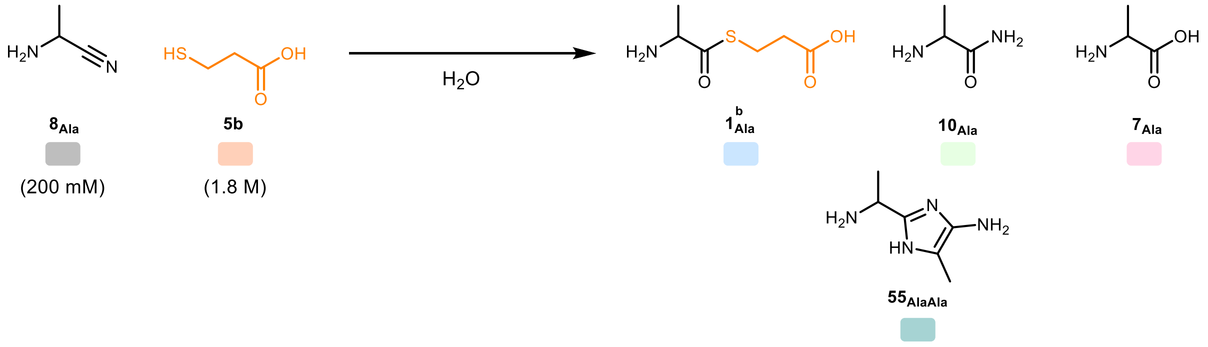

Entry	pH	1^b_{Ala} (%)	10_{Ala} (%)	7_{Ala} (%)	55_{AlaAla} (%)
1	2	28	3	3	1
2	3	48	8	11	6
3	4	33	14	14	24
4	5	13	11	9	33
5	7	n.d.	obs.	obs.	35
6	9	n.d.	obs.	obs.	40

Supplementary Table 42. The yields of aminoacyl thiol $\boldsymbol{1^b_{Ala}}$ *after the reaction of alanine nitrile (**8**$_{Ala}$, 200 mM) with 3-mercaptopropionic acid (**5b**, 1.8 M) at specified pH after 120 h according to General Procedure K. n.d. = not detected.; obs. = signal obscured by resonance overlap.*

Entry	pH	days	1^b_{Ala} (%)	10_{Ala} (%)	7_{Ala} (%)	55_{AlaAla} (%)
1	4	1	30	7	2	16
2	4	2	42	10	5	20
3	4	3	35	13	8	21
4	4	5	33	14	14	24
5	5	1	20	9	3	32
6	5	2	18	9	5	34
7	5	3	15	9	7	37
8	5	5	11	8	10	35

Supplementary Table 43: The yields of aminoacyl thiol $\boldsymbol{1^b_{Ala}}$ *after the reaction of alanine nitrile (**8**$_{Ala}$, 200 mM) with 3-mercaptopropionic acid (**5b**, 1.8 M) at specified pHs over a week according to General Procedure K. n.d. = not detected.; obs. = signal obscured by resonance overlap.*

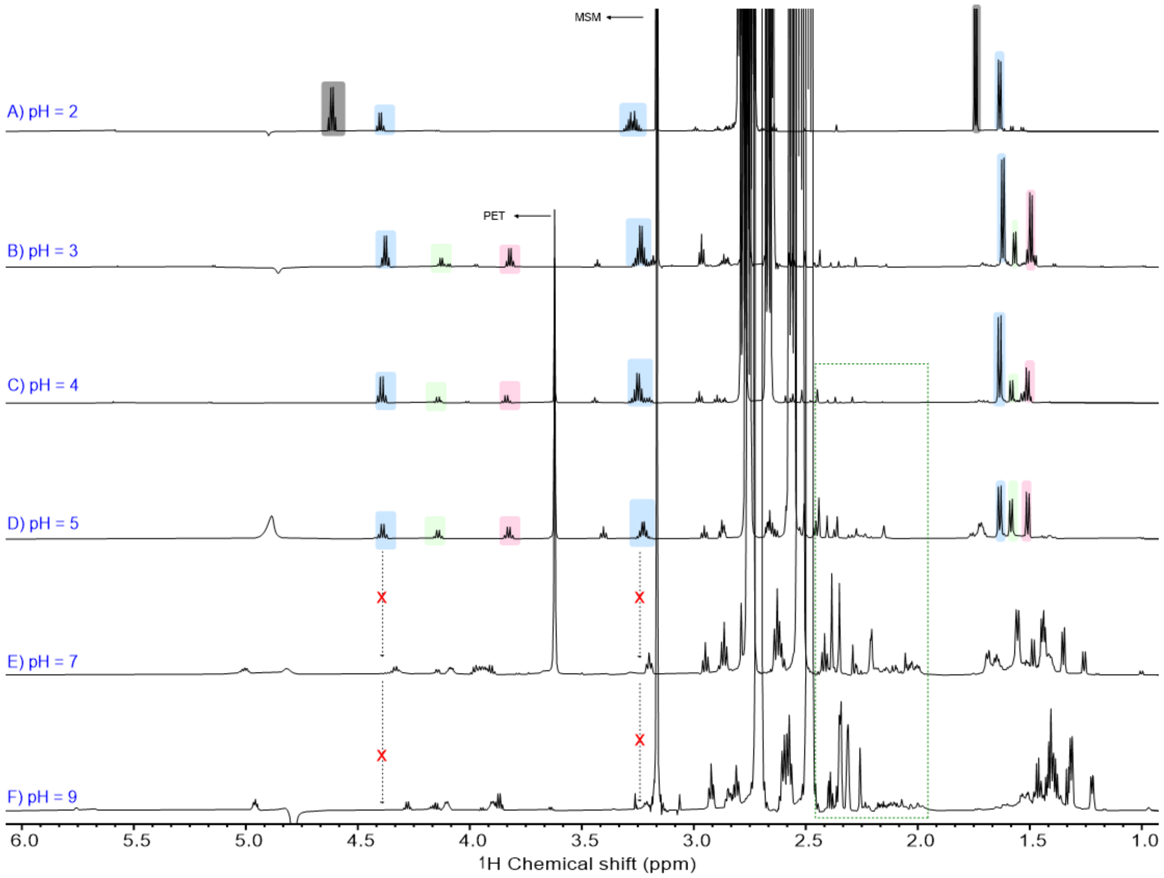

Supplementary Figure 185. ^{1}H NMR (600 MHz, H_2O/D_2O 9:1, noesygppr1d, 1.0 – 6.0 ppm) spectra showing the formation of thioester $\mathbf{1^b_{Ala}}$ *from the reaction of alanine nitrile (*$\mathbf{8_{Ala}}$*, 200 mM) with 3-mercaptopropanoic acid (**5b**, 1.8 M) at room temperature and the specified pH according to General Procedure K: A) at pH 2 after 120 hours, B) at pH 3 after 120 hours, C) at pH 4 after 120 hours, D) at pH 5 after 120 hours, E) at pH 7 after 24 hours, and F) at pH 9 after 24 hours. The characteristic resonances for thioester* $\mathbf{1^b_{Ala}}$ *formation (see for example Supplementary Figure 5), were not observed at pH 7 and pH 9 in* ^{1}H*,* ^{13}C *or* ^{1}H*-*^{13}C *HMBC NMR spectra. The green highlighted box (2.00 – 2.50 ppm) contains aminoimidazole* $\mathbf{55_{AlaAla}}$ *and its derivative species.*

Reaction of alanine nitrile $\mathbf{8}_{Ala}$ with specified stoichiometry of 3-mercaptopropanoic acid ***5b***

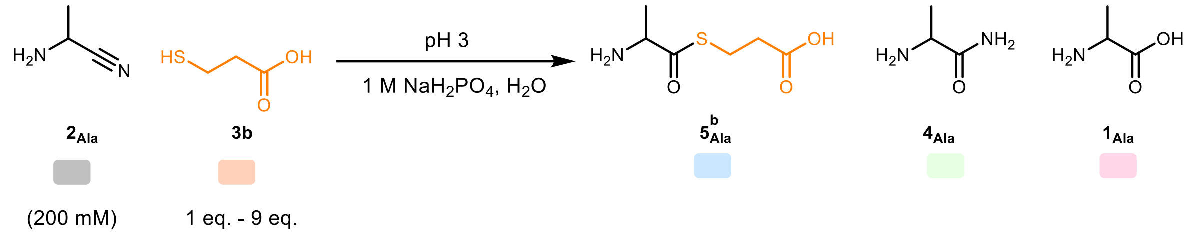

Entry	5b (equiv.)	1^{b}_{Ala} (%)	10_{Ala} (%)	7_{Ala} (%)
1	1	12	4	2
2	2	23	3	6
3	5	32	9	6
4	7	47	9	7
5	9	56	17	13

Supplementary Table 44. Products of the reaction of alanine nitrile ($\mathbf{2}_{Ala}$*, 200 mM) with specified stoichiometry of 3-mercaptopropionic acid (***5b***) at pH 3 after 120 hours. Set up following General Procedure K.*

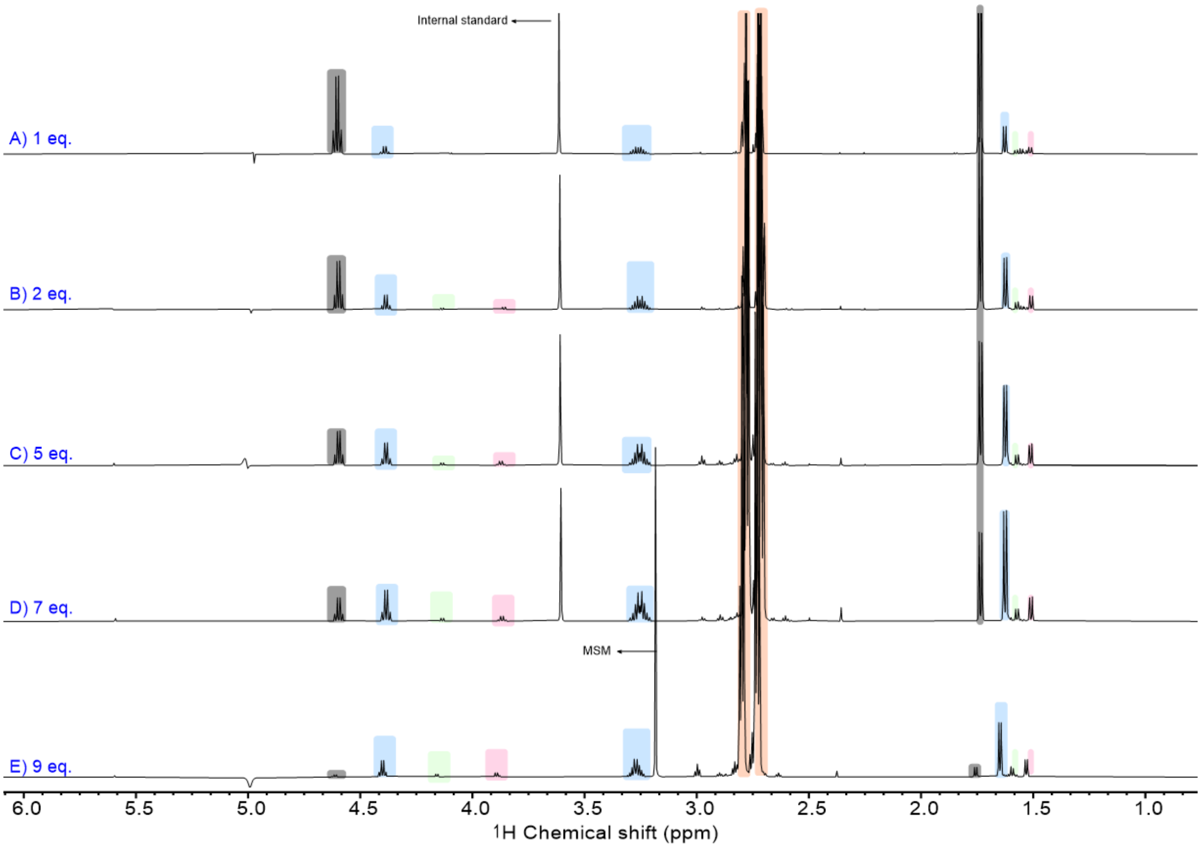

Supplementary Figure 186. The ¹H NMR (600 MHz, H_2O/D_2O 9:1, noesygppr1d, 1.0 – 6.0 ppm) spectra to show the formation of thioester $\mathbf{1}^{b}_{Ala}$ *upon the reaction of alanine nitrile (*$\mathbf{8}_{Ala}$*, 200 mM) with specified stoichiometry of 3-mercaptopropanoic acid (***5b***) at pH 3 and room temperature after 120 hours, set up following General Procedure K. A)* $\mathbf{8}_{Ala}$ *(200 mM) with* ***5b*** *(200 mM) and pentaerythritol (10 mM) as an internal standard. B)* $\mathbf{8}_{Ala}$ *(200 mM) with* ***5b*** *(400 mM) and pentaerythritol (10 mM) as an internal standard. C)* $\mathbf{8}_{Ala}$ *(200 mM) with* ***5b*** *(1 M) and pentaerythritol (10 mM) as an internal standard. D)* $\mathbf{8}_{Ala}$ *(200 mM) with* ***5b*** *(1.8 M) and pentaerythritol (10 mM) as an internal standard. E)* $\mathbf{8}_{Ala}$ *(200 mM) with* ***5b*** *(1.8 M) and MSM (100 mM) as an internal standard.*

Entry	5b	days	1^{b}_{Ala} (%)	10_{Ala} (%)	7_{Ala} (%)
1	1	1	3	1	--
2	1	2	4	1	--
3	1	3	6	1	2
4	1	5	9	2	2
5	1	7	12	2	4
6	2	1	6	1	1
7	2	2	10	1	1
8	2	3	15	2	1
10	2	5	21	6	3
11	2	7	28	11	4
12	5	1	9	1	1
13	5	2	18	2	1
14	5	3	24	4	3
15	5	4	31	4	7
16	5	5	36	4	9
17	5	7	43	6	15
18	7	1	13	1	1
19	7	2	24	3	2
20	7	3	32	4	3
21	7	4	41	5	7
22	7	5	45	6	9
23	7	7	52	7	15
24	9	1	27	5	3
25	9	2	45	8	6
26	9	3	51	11	9
27	9	4	53	12	14
28	9	5	56	15	19
29	9	7	54	14	25

Supplementary Table 45: The yields of aminoacyl thiol $\mathbf{1^{b}_{Ala}}$ *after the reaction of alanine nitrile (*$\mathbf{8_{Ala}}$*, 200 mM) with different concentrations of 3-mercaptopropionic acid (**5b**, 1.8 M) over a week, according to General Procedure K. n.d. = not detected.; obs. = signal obscured by resonance overlap.*

Reaction of alanine nitrile $\mathbf{8_{Ala}}$ *with 3-mercaptopropanoic acid* ***5b***

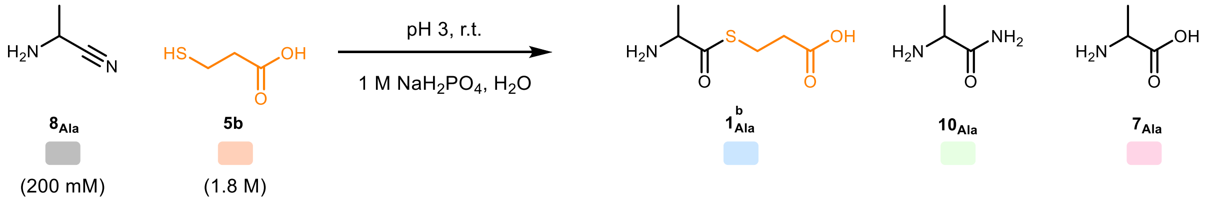

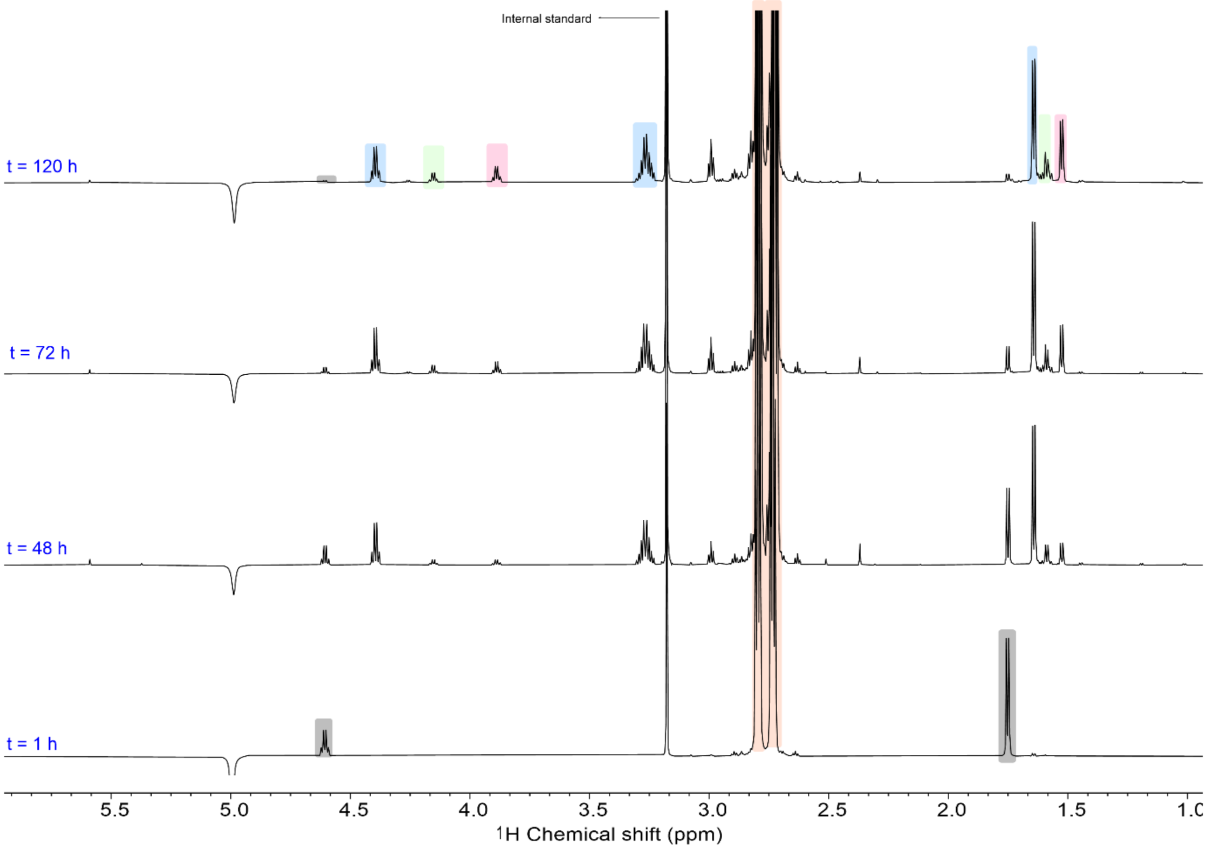

Supplementary Figure 187. [1]*H NMR (600 MHz,* H_2O/D_2O *9:1, noesygppr1d, 1.0 – 6.0 ppm) spectra to show the reaction of alanine nitrile (*$\mathbf{8_{Ala}}$*, 200 mM) with 3-mercaptopropanoic acid (**5b**, 1.8 M) in 1 M* NaH_2PO_4 *buffer, with MSM (100 mM) as an internal standard at pH 3 and room temperature. Set up following General Procedure A. α,β-keto-aldehyde hydrate* $\mathbf{58_{Ala}}$*•*H_2O *= 5.57 p.p.m. singlet resonance (~1% yield).* $\mathbf{7_{Ala}}$ *and* $\mathbf{10_{Ala}}$ *were confirmed by spiking with authentic standards.*

^{1}H NMR (600 MHz, H_2O/D_2O 9:1) (partial assignment) $\mathbf{1^b_{Ala}}$: δ_H 4.39 (1H, q, *J* = 7.2 Hz, Ala-α-C*H*(CH3)), 3.34 – 3.21 (m, 2H, COSCH_2), 1.64 (d, *J* = 7.2 Hz, CH(CH_3)).

$\mathbf{10_{Ala}}$: δ_H 4.14 (1H, q, *J* = 7.1 Hz, Ala-α*H*-CONH$_2$), 1.58 (3H, d, *J* = 7.1 Hz CH(CH_3)).

$\mathbf{7_{Ala}}$: δ_H 3.88 (1H, q, *J* = 7.2 Hz, Ala-α*H*-COOH), 1.52 (3H, d, *J* = 7.2 Hz CH(CH_3)).

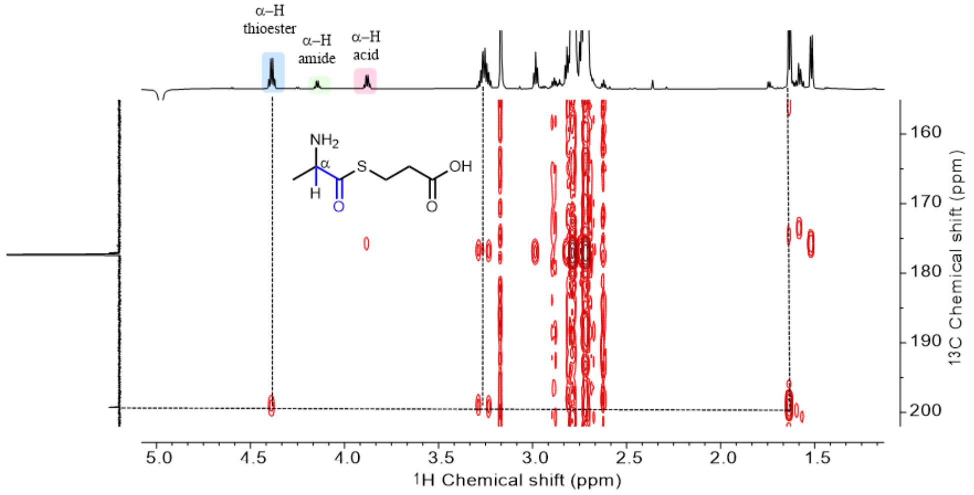

Supplementary Figure 188. ^{1}H–^{13}C *HMBC (*^{1}H*: 600 MHz [1.0 – 5.0 ppm],* ^{13}C*: 176 MHz [160 – 202 ppm],* H_2O/D_2O *9:1) spectrum showing the diagnostic* ^{2}JCH *and* ^{3}JCH *coupling of both Alanyl-aH and -*SCH_2 *in* $\mathbf{5^{b}_{Ala}}$ *at 4.38 ppm and 3.25 ppm with a resonance at 199.3 ppm, which is characteristic of thioester bond formation.*

Reaction of alanine nitrile $\boldsymbol{8_{Ala}}$ *with 2-mercaptoethanesulfonate* ***5c***

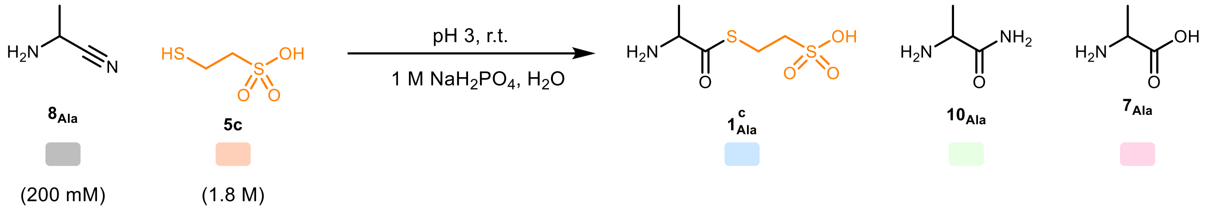

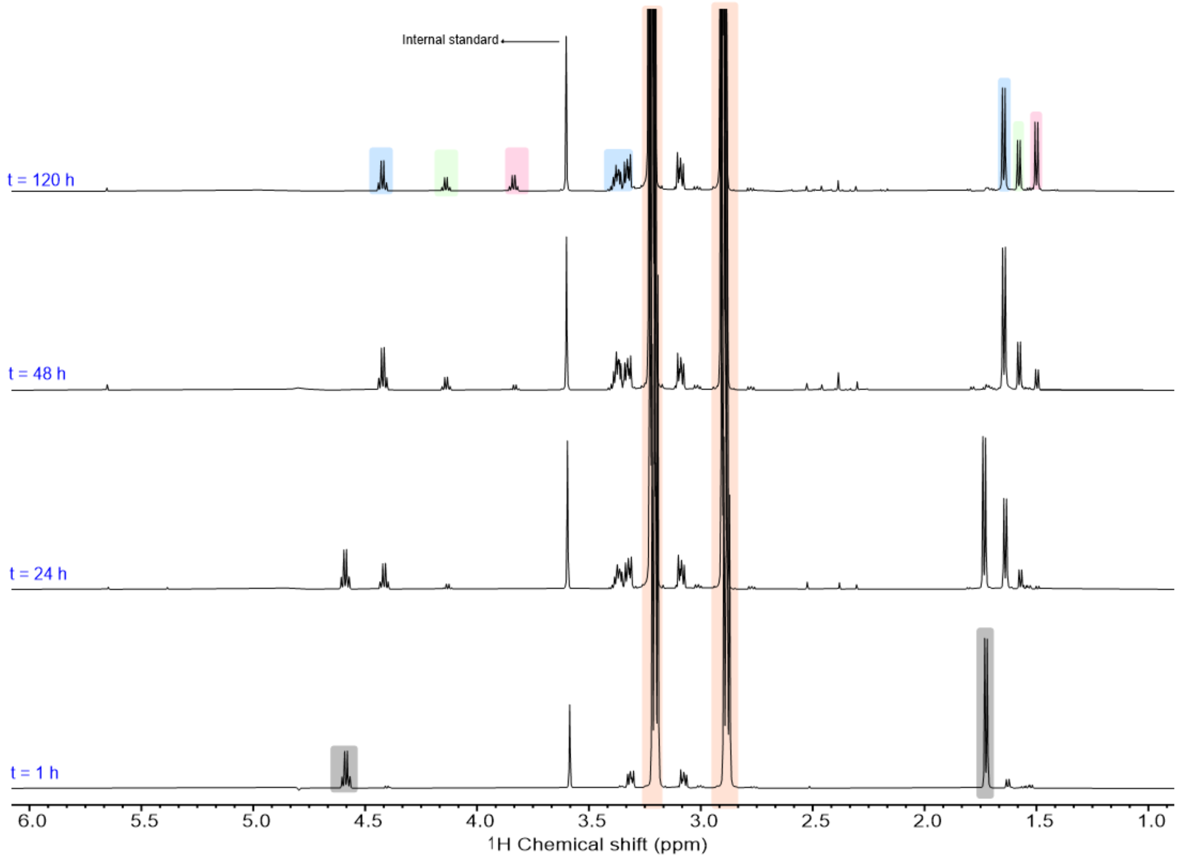

Supplementary Figure 189. *^{1}H NMR (600 MHz, H_2O/D_2O 9:1, noesygppr1d, 1.0 – 6.0 ppm) spectra to show the reaction of alanine nitrile (*$\boldsymbol{8_{Ala}}$*, 200 mM) with 2-mercaptoethanesulfonate (**5c**, 1.8 M) in 1 M NaH_2PO_4 buffer, with pentaerythritol (10 mM) as an internal standard at pH 3 and room temperature. Set up following General Procedure A.*

^{1}H NMR (600 MHz, H_2O/D_2O 9:1) (partial assignment) $\boldsymbol{1^{c}_{Ala}}$: δ_H 4.42 (1H, q, J = 7.2 Hz, Ala-αH-$COSCH_2$), 3.46 – 3.29 (2H, m, $COSCH_2$), 1.64 (3H, d, J = 7.2 Hz, $CH(CH_3)$).

$\boldsymbol{10_{Ala}}$: δ_H 4.14 (1H, q, J = 7.1 Hz, Ala-αH-$CONH_2$), 1.58 (3H, d, J = 7.1 Hz $CH(CH_3)$).

$\boldsymbol{7_{Ala}}$: δ_H 3.83 (1H, q, J = 7.2 Hz, Ala-αH-COOH), 1.50 (3H, d, J = 7.2 Hz, $CH(CH_3)$).

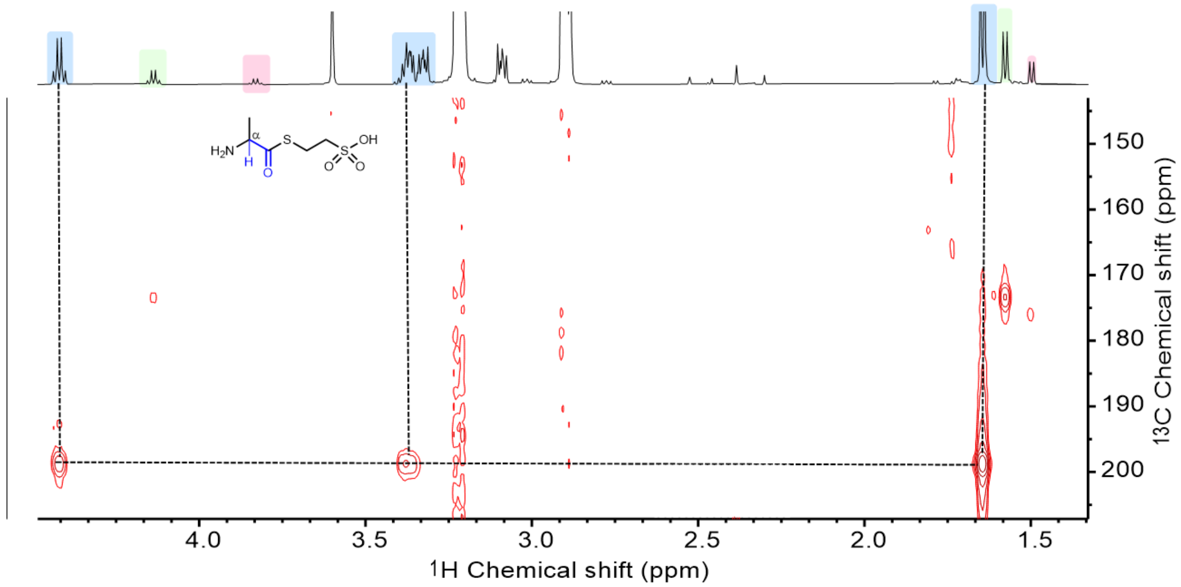

Supplementary Figure 190. ^1H–^{13}C HMBC (^1H: 600 MHz [1.3 – 4.5 ppm], ^{13}C: 176 MHz [150 – 209 ppm], H_2O/D_2O 9:1) spectrum showing the diagnostic ^2JCH and ^3JCH of Alanyl-aH and -SCH_2 in $\mathbf{1^c_{Ala}}$ *at 4.42 ppm and 3.37 ppm with a resonance at 198.5 ppm, which is characteristic of thioester bond formation.*

Reaction of alanine nitrile ***8**$_{Ala}$* *with N-acetylcysteamine* ***5d***

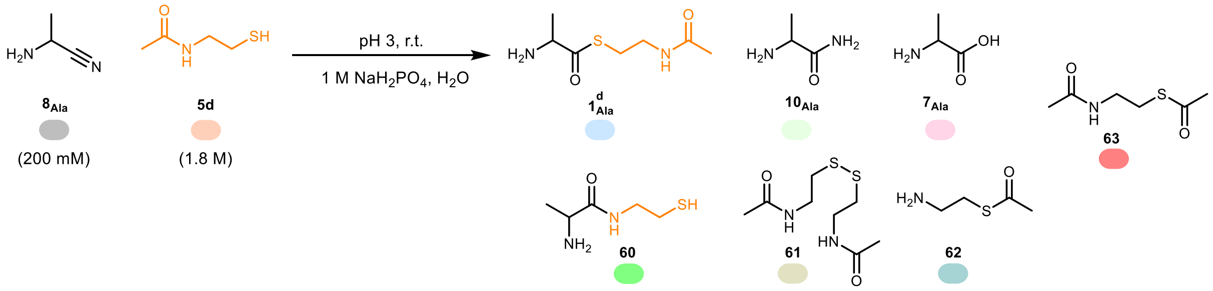

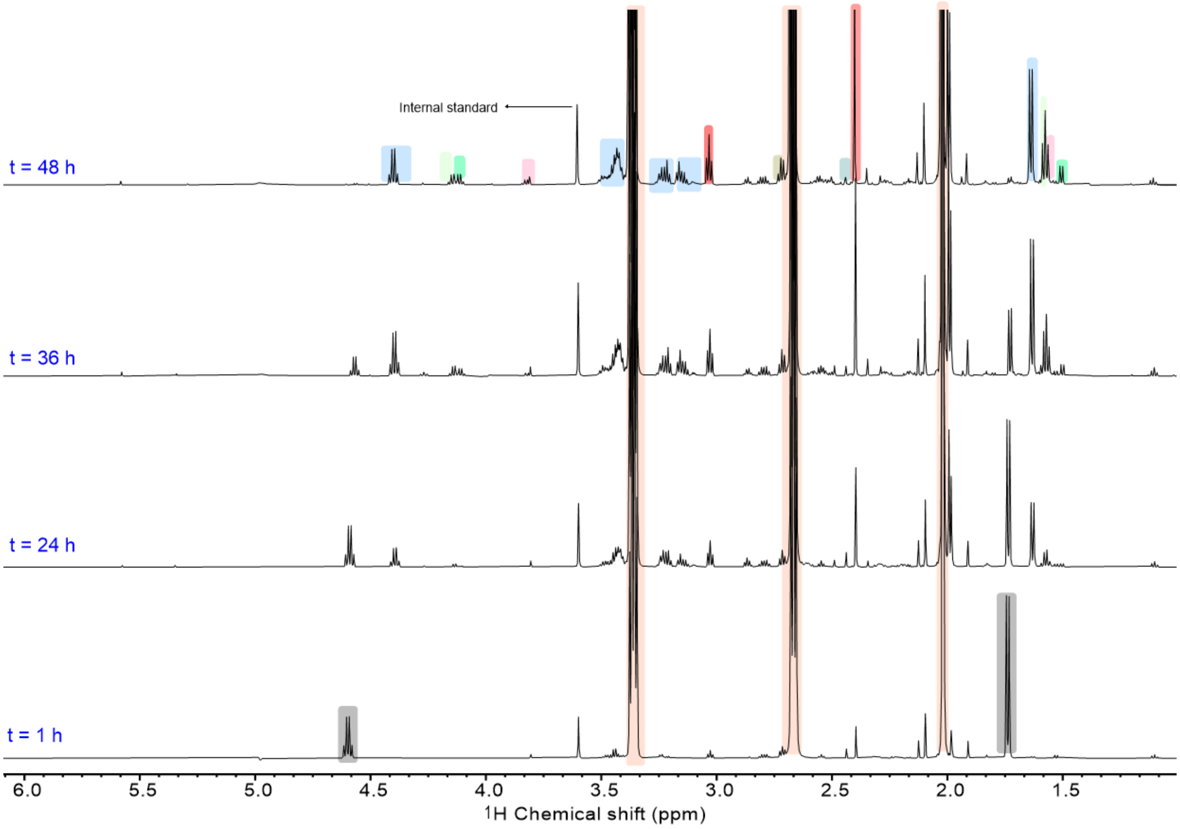

*Supplementary Figure 191. ¹H NMR (600 MHz, H_2O/D_2O 9:1, noesygppr1d, 1.0 – 6.0 ppm) spectra to show the reaction of alanine nitrile (**8**$_{Ala}$, 200 mM) with N-acetylcysteamine (**5d**, 1.8 M) in 1 M NaH_2PO_4 buffer, with pentaerythritol (10 mM) as an internal standard at pH 3 and room temperature. Set up following General Procedure K.*

^{1}H NMR (600 MHz, H_2O/D_2O 9:1) (partial assignment) $\mathbf{1^d_{Ala}}$: δ_H 4.40 (1H, q, *J* = 7.2 Hz, Ala-α*H*-COSCH$_2$), 3.49–3.39 (2H, m, COSCH$_2$C*H*$_2$NH), 3.22 (1H, dt, *J* = 14.1, 6.2 Hz, COSC*H*HCH$_2$), 3.15 (1H, dt, *J* = 14.1, 6.2 Hz, COSCH*H*CH$_2$), 1.61 (3H, d, *J* = 7.2 Hz, CH(C*H*$_3$)).

10$_{Ala}$: δ_H 4.15 (1H, q, *J* = 7.2 Hz, Ala-α*H*-CONH$_2$).

60: δ_H 4.12 (1H, q, *J* = 7.2 Hz, ala-α*H*-CONH).

7$_{Ala}$: δ_H 3.83 (1H, q, *J* = 7.2 Hz, Ala-α*H*-COOH).

63: δ 3.03 (2H, t, *J* = 6.5 Hz, C*H*$_2$COS), 2.40 (3H, s, CH$_2$COS(C*H*$_3$)).

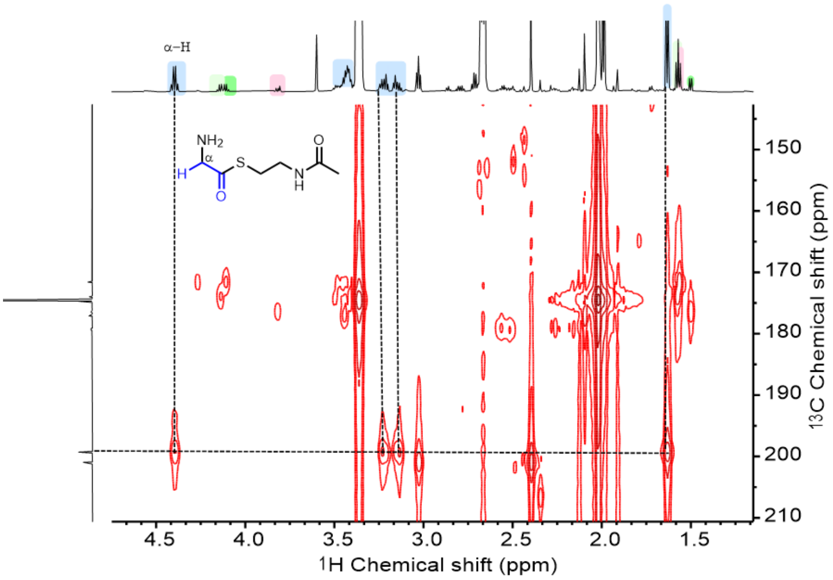

Supplementary Figure 192. ^{1}H*–*^{13}C *HMBC (*^{1}H*: 600 MHz [1.0 – 4.5 ppm],* ^{13}C*: 176 MHz [150 – 210 ppm],* H_2O/D_2O *9:1) spectrum showing the diagnostic* ^{2}JCH *and* ^{3}JCH *coupling of Alanyl-aH and -SCH in* $\mathbf{1^{d}_{Ala}}$ *at 4.40 ppm and 3.15 ppm with a resonance at 199.3 ppm, which is characteristic of thioester bond formation.*

*Reaction of alanine nitrile **8_{Ala}** with pantetheine **5a***

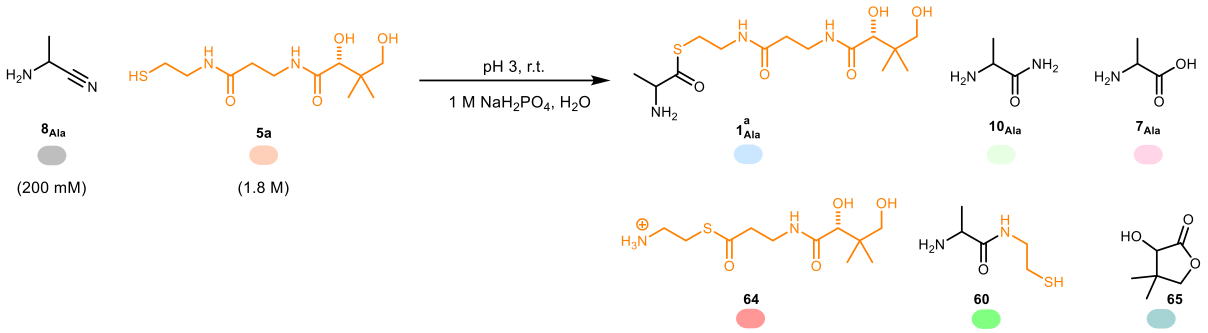

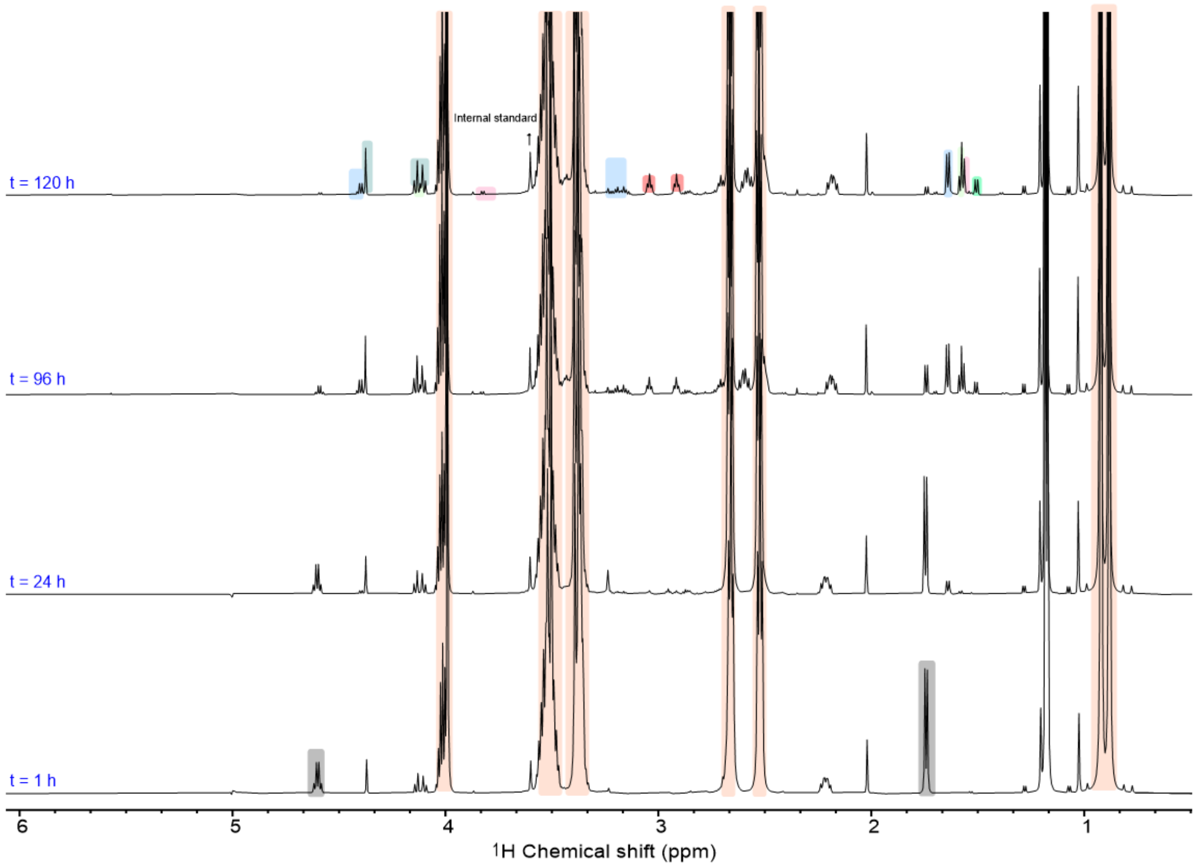

Supplementary Figure 193. [1]*H NMR (600 MHz, H_2O/D_2O 9:1, noesygppr1d, 1.0 – 6.0 ppm) spectra to show the reaction of alanine nitrile (**8_{Ala}**, 200 mM) with pantetheine (**5a**, 1.8 M) in 1 M NaH_2PO_4 buffer, with pentaerythritol (10 mM) as an internal standard at pH 3 and room temperature. Set up following General Procedure K.*

^{1}H NMR (600 MHz, H_2O/D_2O 9:1) **1^a_{Ala}** (partial assignment) : δ_H 4.40 (1H, q, J = 7.2 Hz, Ala-α*H*-COSCH$_2$), 3.29–3.13 (1H, dt, J = 14.1, 6.2 Hz, COSC*H*HCH$_2$), 1.64 (3H, d, J = 7.2 Hz, CH(C*H*$_3$)).

7_{Ala}: δ_H 3.82 (1H, q, J = 7.2 Hz, Ala-α*H*-COOH).

65: δ_H 4.37 (1H, s, -C*H*(OH)CO), 4.14 (1H, AB, J = 8.5 Hz, -CH(OH)C*H*H), 4.10 (1H, AB, J = 8.5 Hz, -CH(OH)C*H*H).

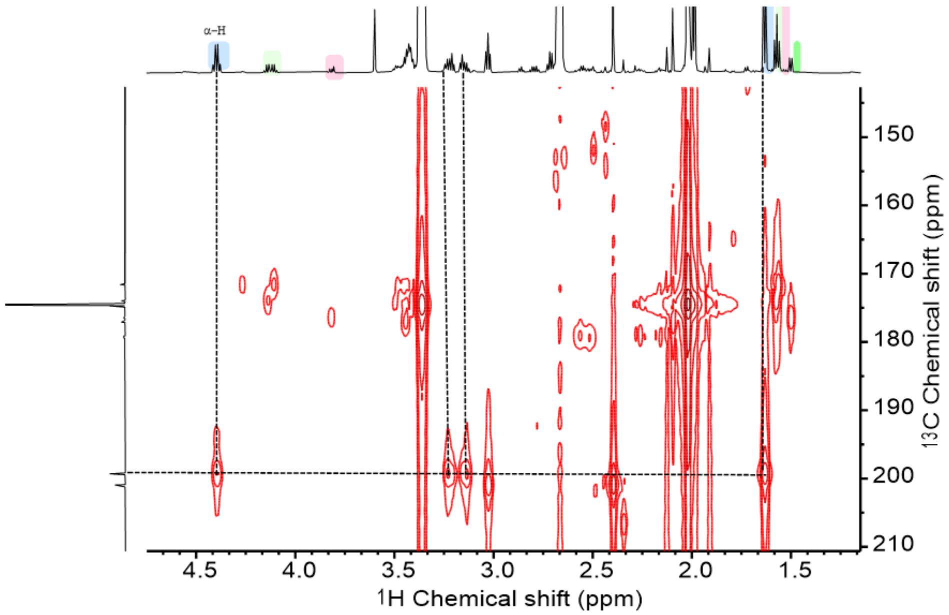

Supplementary Figure 194. ^{1}H–^{13}C *HMBC* (^{1}H*: 600 MHz [1.0 – 4.5 ppm],* ^{13}C*: 176 MHz [150 – 210 ppm],* H_2O/D_2O *9:1) spectrum showing the diagnostic* ^{2}JCH *and* ^{3}JCH *coupling of both Alanyl-aH and -SCH in* $\mathbf{1}^{a}_{Ala}$ *at 4.40 ppm and 3.19 ppm with a resonance at 199.0 ppm, which is characteristic of thioester bond formation.*

Reaction of alanine nitrile ***8**$_{Ala}$* *with 3-mercaptopropanoic acid* ***5b*** *at pH 7 and -7 °C*

3-Mercaptopropionic acid **5b** (17.4 µL, 200 µmol), NaH_2PO_4 (120 mg, 1 mmol) and pentaerythritol (55 µL of a 50 mM solution, 2.75 µmol) were dissolved in degassed H_2O (15 mL). The solution was adjusted to pH 7.0 with 4 M NaOH. 2-Amino propionitrile hydrochloride **8**$_{\mathbf{Ala}}$ (2.1 mg, 20 µmol)[6] was added and the solution was re-adjusted to pH 7.0 with 4 M NaOH. The solution volume was made up to 20 mL, and the solution was aliquoted into 1 mL screw-cap Eppendorf tubes. The samples were flash-frozen at -80 °C, then incubated in a cryogenic bath at -7 °C. Aliquots were removed periodically and quenched by addition of 5,5-dithio-bis-(2-nitrobenzoic acid) (4 mg, 10 µmol, to oxidise **5b**) and D_2O (50 µL). NMR spectra were then acquired and quantified against pentaerythritol as internal standard. After 30 days no **8**$_{\mathbf{Ala}}$ was visible in the solution.

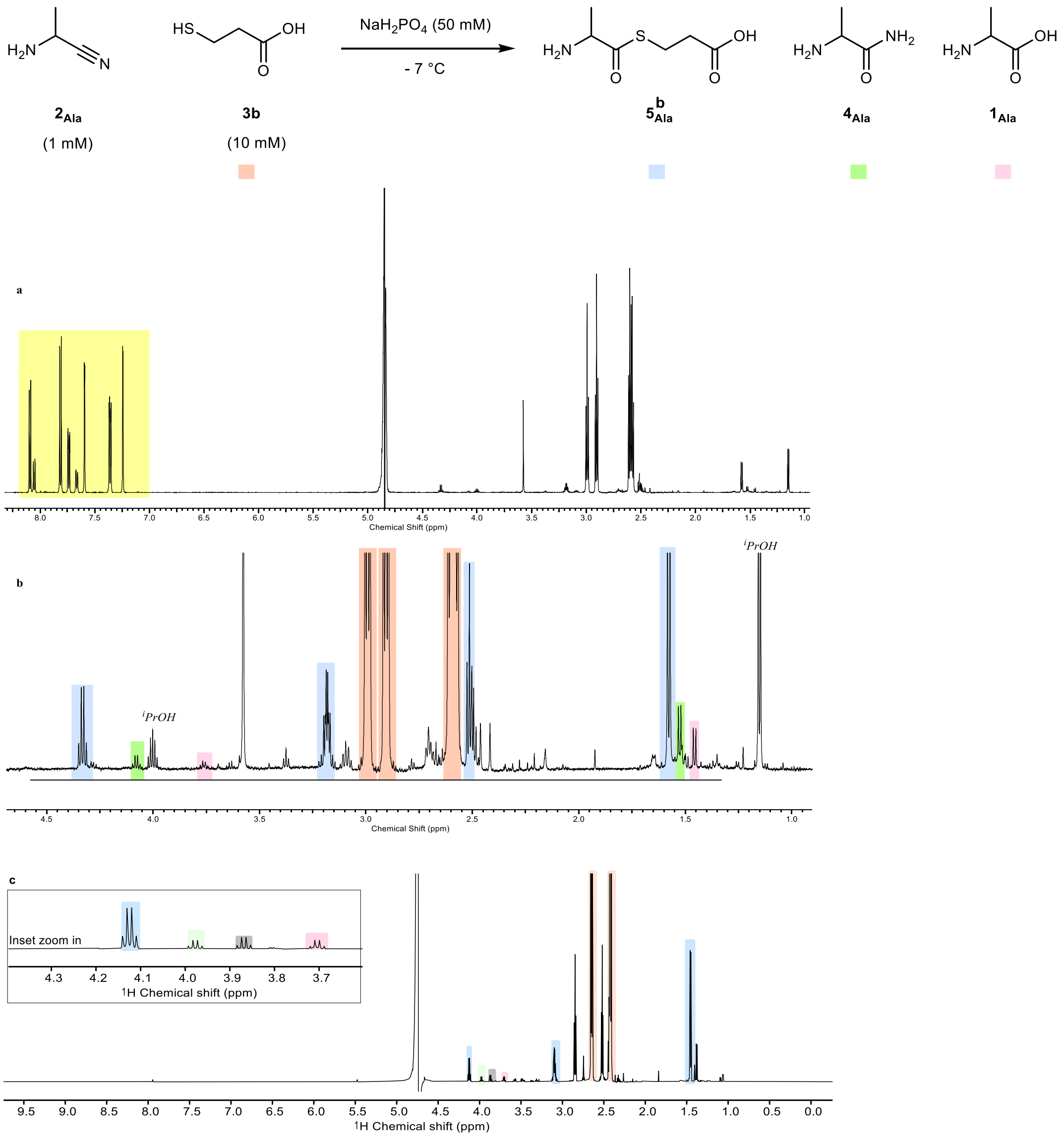

Supplementary Figure 195. *¹H NMR (700 MHz, H_2O/D_2O 19:1, noesygppr1d) spectrum to show the formation of thioester* $\mathbf{1}^{b}_{Ala}$ *from the reaction of* **8**$_{Ala}$ *(1 mM) with 3-mercaptopropanoic acid (**5b**, 10 mM) after 30 days at -7 °C (spectrum acquired after thawing and quenching with 5,5-dithio-bis-(2-nitrobenzoic acid)).* ***a)*** *1.0 – 8.5 ppm;* ***b)*** *0.9 – 4.6 ppm. Pentaerythritol = 3.57 (8H, s, $(CH_2)_4$).* ***c)*** *spectrum to show the formation of thioester* $\mathbf{1}^{b}_{Ala}$ *from the reaction of* **8**$_{Ala}$ *(1 mM) with 3-mercaptopropanoic acid (**5b**, 10 mM) after 35 days at -7 °C.*

[6] **8**$_{\mathbf{Ala}}$ contained 7% w/w isopropanol when purified by recrystallisation from isopropanol. This can be avoided, if required, by recrystallisation of **8**$_{\mathbf{Ala}}$ from EtOH.

Due to the inherently slow nature of these eutectic reactions, and the similarity of the result to room temperature reactions at pH 3-4, these conditions were not optimized further.

Side chain compatibility for the formation of thioesters $\mathbf{1^b_{Aaa}}$ *from the reaction of α-aminonitrile* $\mathbf{8_{Aaa}}$ *with 3-mercaptopropanoic acid* **5b**

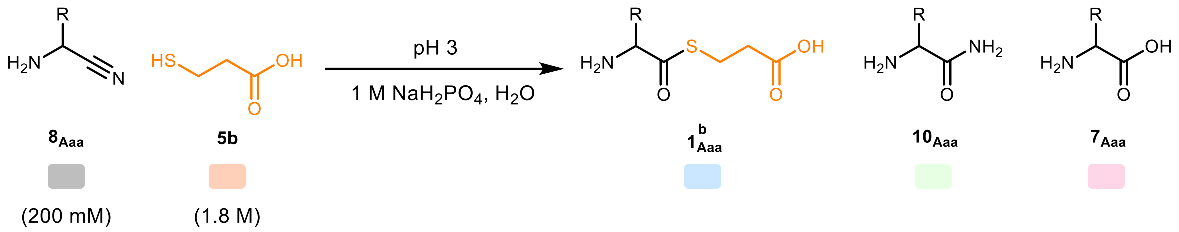

**Entry**	$\mathbf{1_{Aaa}}$	Time (days)	**5b** (equiv.)	$\mathbf{1^b_{Aaa}}$ (%)	$\mathbf{7_{Aaa}}$ (%)	$\mathbf{10_{Aaa}}$ (%)	**HRMS-ESI for thioester 5** Formula	**Theoretical**	**Found**
1	Gly	5	9	46	25	8	$C_5H_{10}NO_3S$ $[M+H]^+$	164.0376	164.0377
2	Ala	7	9	56	17	13	$C_6H_{12}NO_3S$ $[M+H]^+$	178.0532	178.0536
3	Pro	3	9	64	3	23	$C_8H_{14}NO_3S$ $[M+H]^+$	204.0689	204.0690
4	Ser	3	9	40	10	15	$C_6H_{12}NO_4S$ $[M+H]^+$	220.1002	220.1000
5	Leu	18	9	53	20	16	$C_9H_{18}NO_3S$ $[M+H]^+$	194.0482	194.0485
6	Phe	10	9	21	n.d.	n.d.	$C_{11}H_{16}NO_3S$ $[M+H]^+$	254.3235	254.3240
7	Val	7	9	14	n.d.	7	$C_8H_{16}NO_3S$ [M+H]+	206.0845	206.0849
8	Arg	9	9	41	13	6	$C_9H_{19}N_4O_3S$ $[M+H]^+$	263.1172	263.1160
9	Pip	5	9	58	19	11	$C_9H_{16}O_3NS$ $[M+H]^+$	218.0845	218.0846
10	Met	7	9	38	12	7	$C_8H_{16}O_3NS_2$ $[M+H]^+$	233.3395	233.3391

Supplementary Table 46. Yields and ESI-HRMS data of aminoacyl thiol $\mathbf{1^b_{Aaa}}$*, from the reaction of amino nitrile* $\mathbf{8_{Aaa}}$ *(200 mM) with 3-mercaptopropanoic acid* **5b** *(1.8 M) in 1M* NaH_2PO_4 *at pH 3 and room temperature. Set up following General Procedure K.*

*Reaction of glycine nitrile **8**$_{Gly}$ with 3-mercaptopropanoic acid **5b***

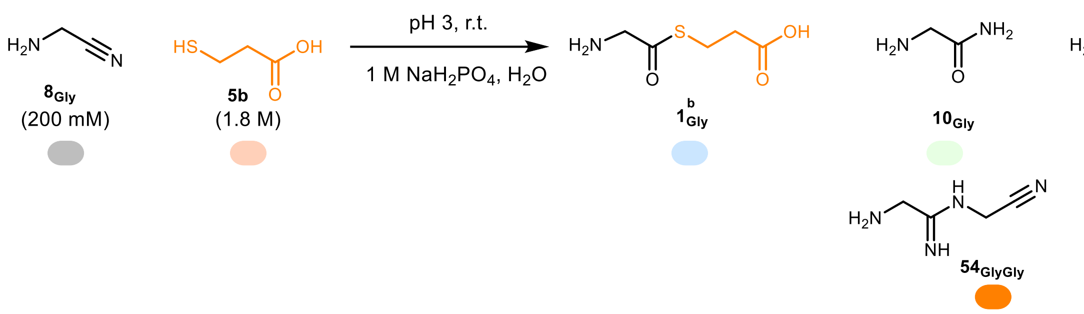

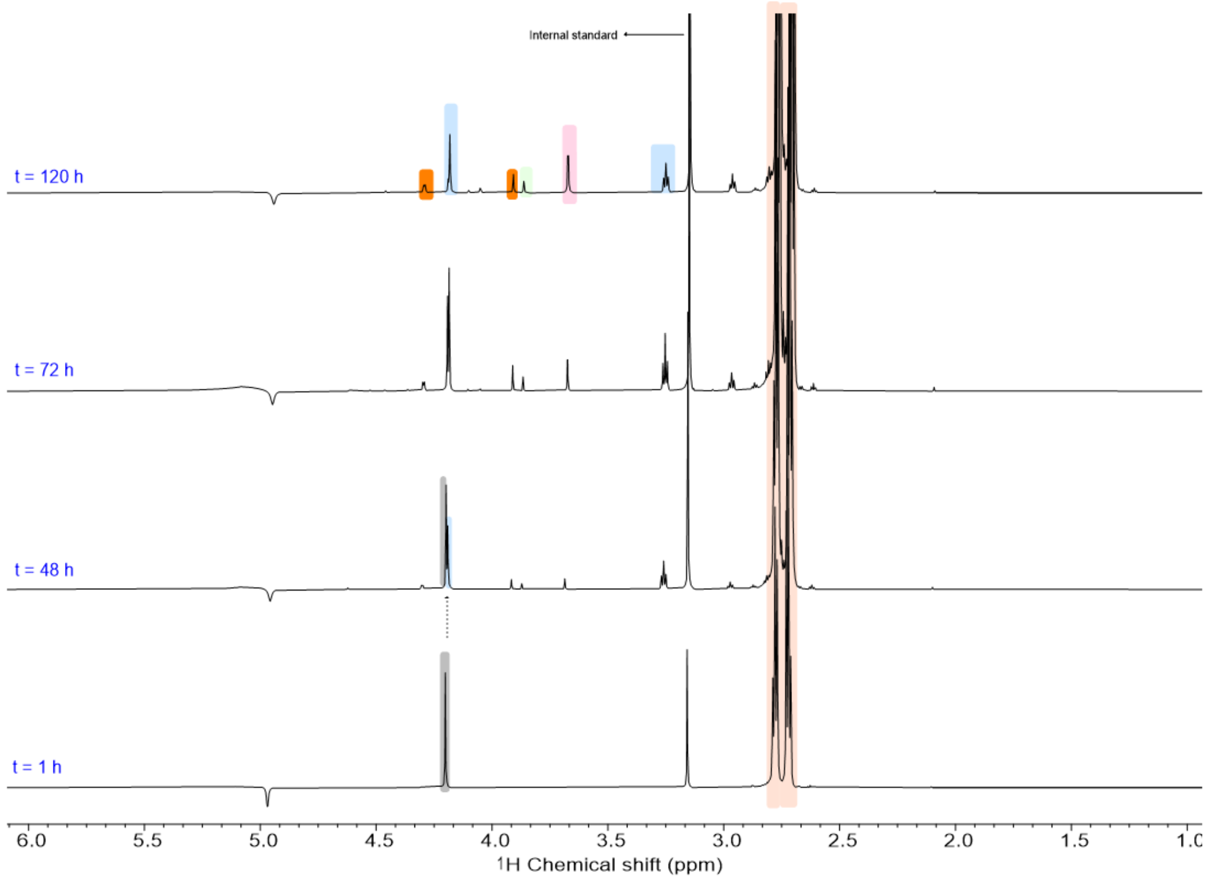

Supplementary Figure 196. [1]*H NMR (600 MHz, H_2O/D_2O 9:1, noesygppr1d, 1.0 – 6.0 ppm) spectra to show the reaction of glycine nitrile (**8**$_{Gly}$, 200 mM) with 3-mercaptopropanoic acid (**5b**, 1.8 M) in 1 M NaH_2PO_4 buffer, with MSM (100 mM) as an internal standard at pH 3 and room temperature. Set up following General Procedure K. α,β-Dialdehyde hydrate **42**$_{Gly}$•H_2O = singlet resonance was not observed.*

^{1}H NMR (600 MHz, H_2O/D_2O 9:1) **1**$^{b}_{Gly}$ (partial assignment): δ_H 4.21 (2H, s, Gly-α-C*H*COSCH$_2$), 3.27 (2H, t, *J* = 6.8 Hz, COSC*H*$_2$).

10$_{Gly}$: δ_H 3.89 (2H, s, Gly-α-C*H*CONH$_2$).

7$_{Gly}$: δ_H 3.70 (2H, s, Gly-α-C*H*COOH).

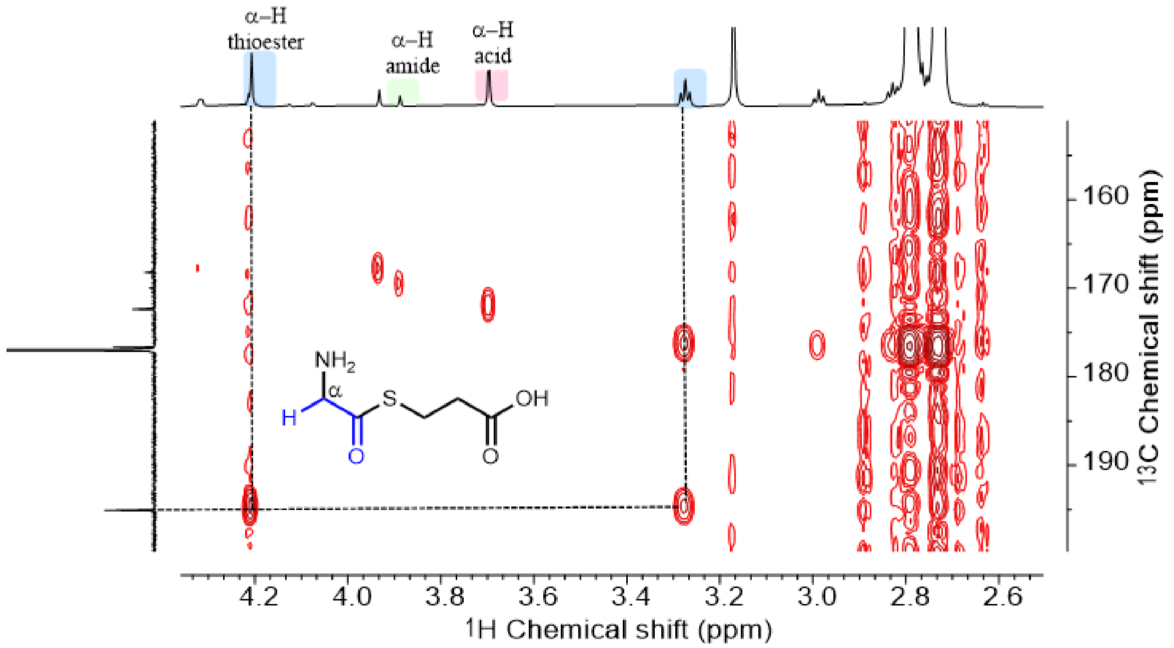

Supplementary Figure 197. ^{1}H–^{13}C HMBC (^{1}H: 600 MHz [2.2 – 4.5 ppm], ^{13}C: 176 MHz [155 – 200 ppm], H_2O/D_2O 9:1) spectrum showing the diagnostic ^{2}JCH and ^{3}JCH coupling of both glycyl-aH and -SCH_2 in $\mathbf{1^{b}_{Gly}}$ *at 4.00 ppm and 3.27 ppm with a resonance at 195.1 ppm, which is characteristic of thioester bond formation.*

Reaction of proline nitrile ***8*** $_{\mathbf{Pro}}$ *with 3-mercaptopropanoic acid* ***5b***

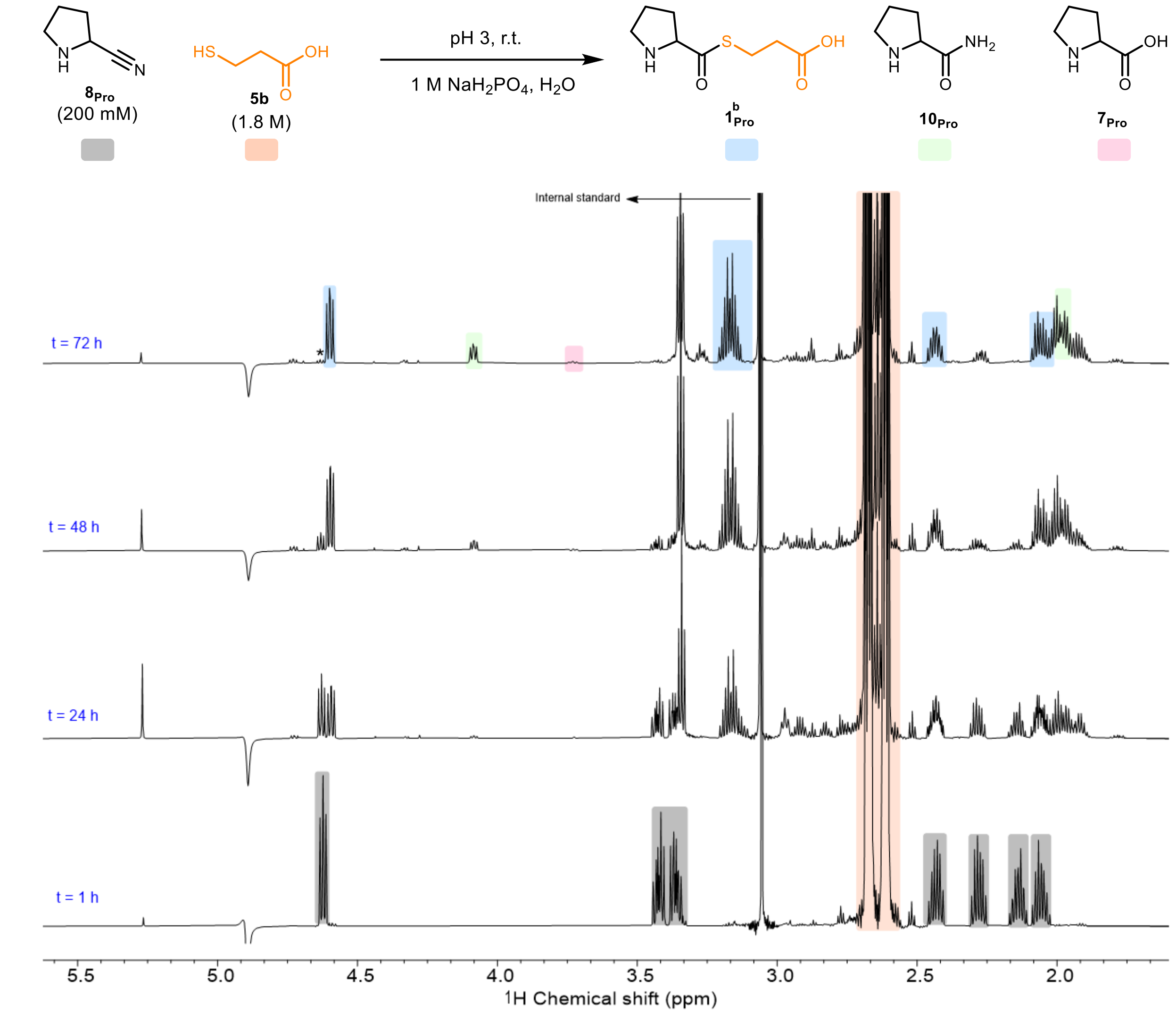

Supplementary Figure 198. ^{1}*H NMR (600 MHz,* H_2O/D_2O *9:1, noesygppr1d, 1.0 – 6.0 ppm) spectra to show the reaction of proline nitrile (**8*** $_{\mathbf{Pro}}$*, 200 mM) with 3-mercaptopropanoic acid (**5b**, 1.8 M) in 1 M* NaH_2PO_4 *buffer, with MSM (100 mM) as an internal standard at pH 3 and room temperature. Set up following General Procedure K.* α,β*-keto-aldehyde hydrate **58*** $_{\mathbf{Pro}}$*•*H_2O *= 5.24 p.p.m. singlet resonance (~2% yield). * Tentatively assigned as 2-(3-mercaptopropionic acid) pyrrolidine, formed via addition of **5b** to the imine product of the retro-Strecker reaction of proline nitrile **8*** $_{\mathbf{Pro}}$ *(~3% yield).*

^{1}H NMR (600 MHz, H_2O/D_2O 9:1) $\mathbf{1^{b}_{Pro}}$ (partial assignment) : δ_H 4.58 (1H, dd, *J* = 8.6, 7.0 Hz, Pro-α-C*H*COSCH$_2$), 3.22 – 3.11 (2H, m, COSC*H*$_2$), 2.42 (1H, dddd, *J* = 13.1, 8.5, 7.2, 6.2 Hz, C*H*HCHCOSC*H*$_2$), 2.08 – 2.01 (1H, m, CH*H*CHCOSC*H*$_2$).

$\mathbf{10_{Pro}}$: δ_H 4.07 (1H, dd, *J* = 8.8, 6.4 Hz, Pro-α-C*H*CONH$_2$).

$\mathbf{7_{Pro}}$: δ_H 3.69 (1H, dd, *J* = 8.8, 7.0 Hz, Pro-α-C*H*COOH).

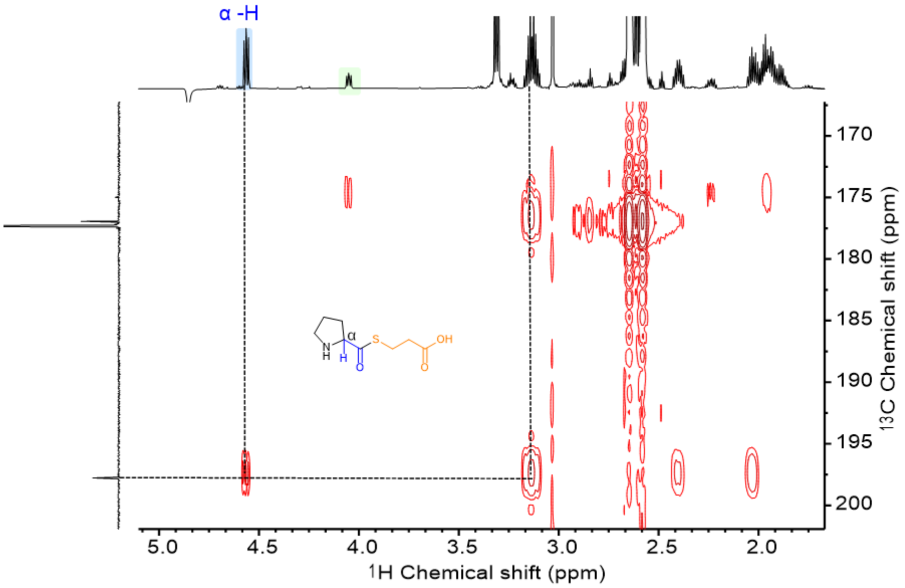

Supplementary Figure 199. *^1H–^{13}C HMBC (^1H: 600 MHz [1.5 – 5.0 ppm], ^{13}C: 176 MHz [169 – 202 ppm], H_2O/D_2O 9:1) spectrum showing the diagnostic ^2JCH and ^3JCH coupling of both Pro-aH and -SCH_2 in $\mathbf{1^{b}_{Pro}}$ at 4.58 ppm and 3.14 ppm with a resonance at 197.7 ppm, which is characteristic of thioester bond formation.*

Reaction of leucine nitrile $\mathbf{8_{Leu}}$ with 3-mercaptopropanoic acid ***5b***

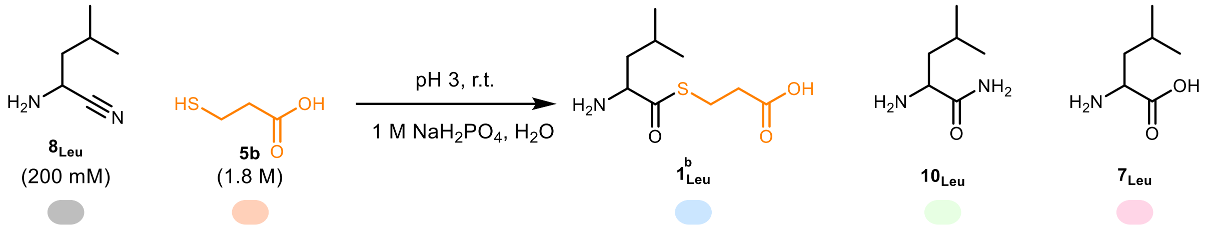

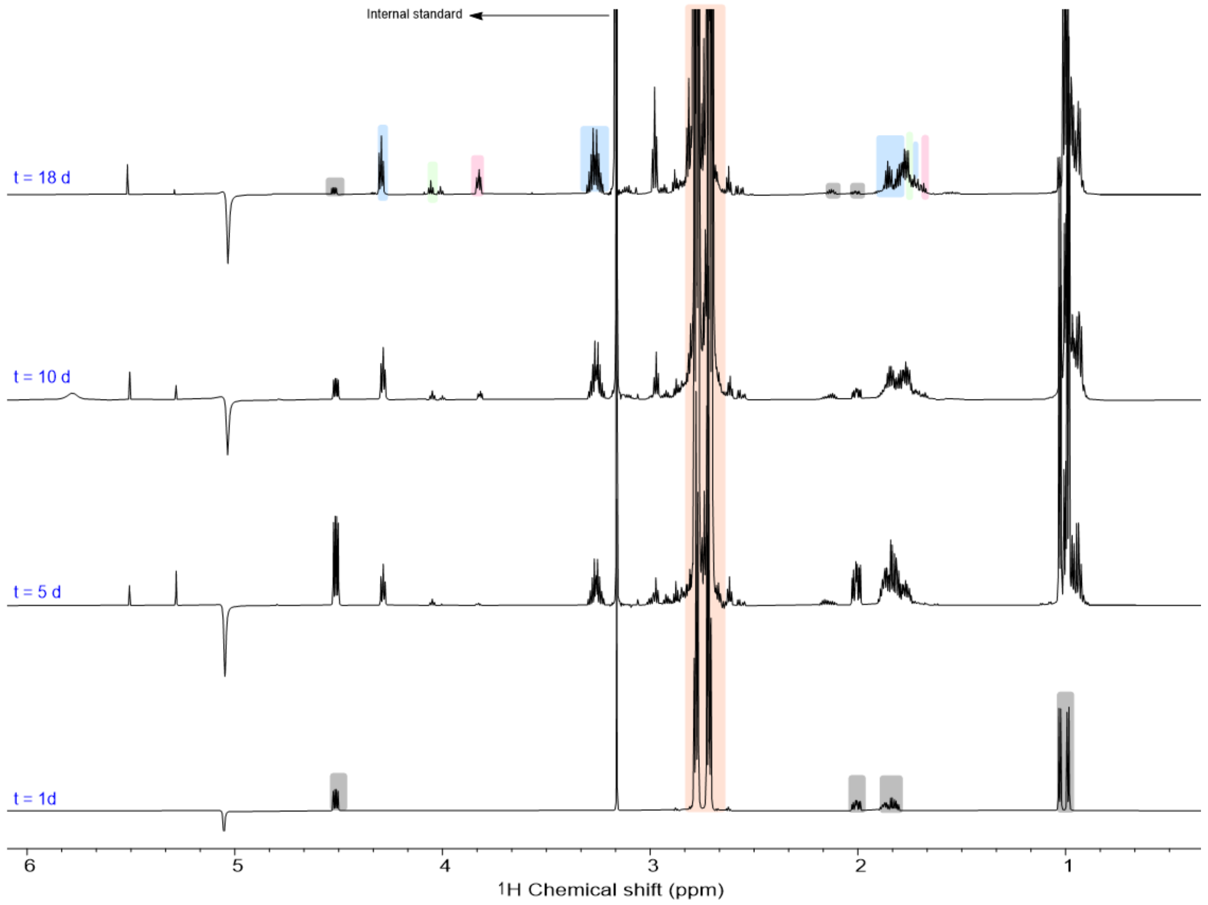

Supplementary Figure 200. ^{1}*H NMR (600 MHz,* H_2O/D_2O *9:1, noesygppr1d, 0.3 – 6.0 ppm) spectra to show the reaction of leucine nitrile (*$\mathbf{8_{Leu}}$*, 200 mM) with 3-mercaptopropanoic acid (**5b**, 1.8 M) in 1 M* NaH_2PO_4 *buffer, with MSM (100 mM) as an internal standard at pH 3 and room temperature. Set up following General Procedure K.* α,β*-keto-aldehyde hydrate* $\mathbf{58_{Luc}}$*•*H_2O *= 5.51 p.p.m. singlet resonance and its precursor* $\mathbf{57_{Leu}}$ *= 5.28 p.p.m. singlet resonance (combined yield 7%).*

^{1}H NMR (600 MHz, H_2O/D_2O 9:1) $\mathbf{1^b_{Leu}}$ (partial assignment) : δ_H 4.29 (1H, dd, *J* = 7.8, 6.2 Hz, Leu-α-C*H*COSCH$_2$), 3.41 – 3.20 (2H, m, COSC*H*$_2$), 1.95 – 1.65 (2H, m, C*H*$_2$(CH$_3$)), 1.19 – 0.85 (6H, m, C*H*$_2$(CH$_3$)).

$\mathbf{10_{Leu}}$: δ_H 4.05 (1H, t, *J* = 7.2 Hz, Leu-α-C*H*CONH$_2$).

$\mathbf{7_{Leu}}$: δ_H 3.82 (1H, m, Leu-α-C*H*COOH).

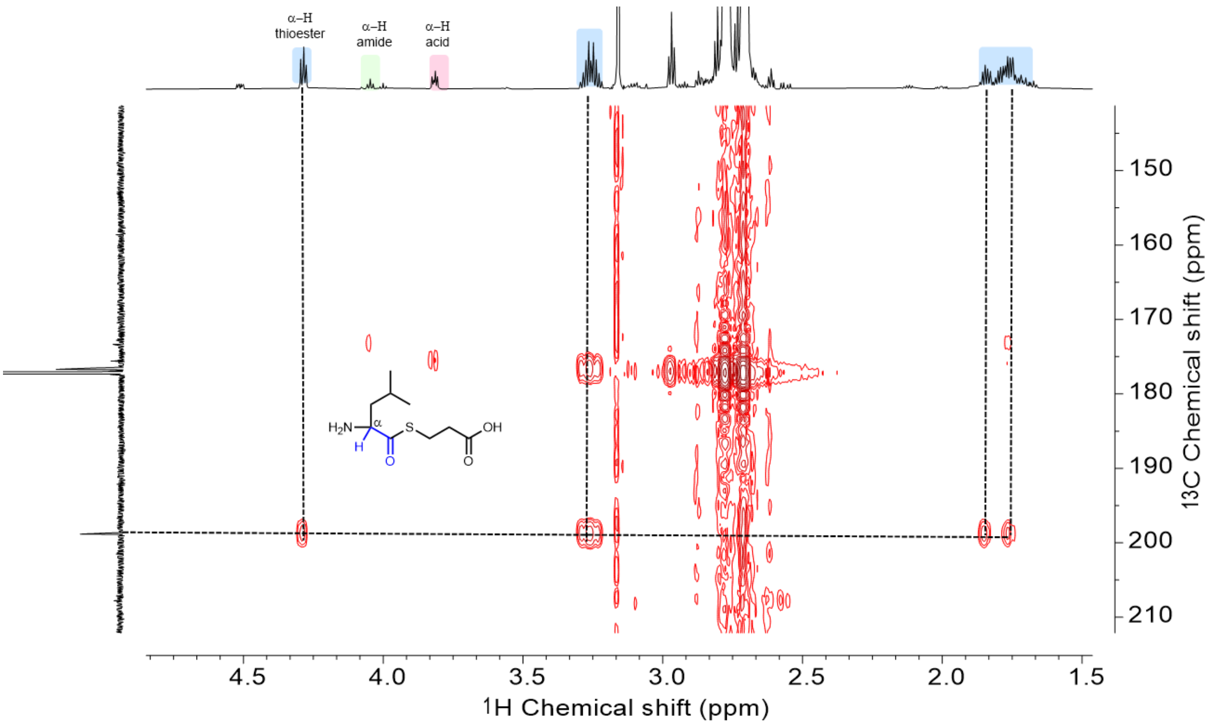

Supplementary Figure 201. *^1H–^{13}C HMBC (^1H: 600 MHz [1.3-4.5 ppm], ^{13}C: 176 MHz [163 – 207 ppm], H_2O/D_2O 9:1) spectrum showing the diagnostic ^2JCH and ^3JCH coupling of both Leucyl-aH and -SCH_2 in $\mathbf{1^{b}_{Leu}}$ at 4.29 ppm and 3.26 ppm with a resonance at 198.8 ppm, which is characteristic of thioester bond formation.*

*Reaction of methionine nitrile **8_{Met}** with 3-mercaptopropanoic acid **5b***

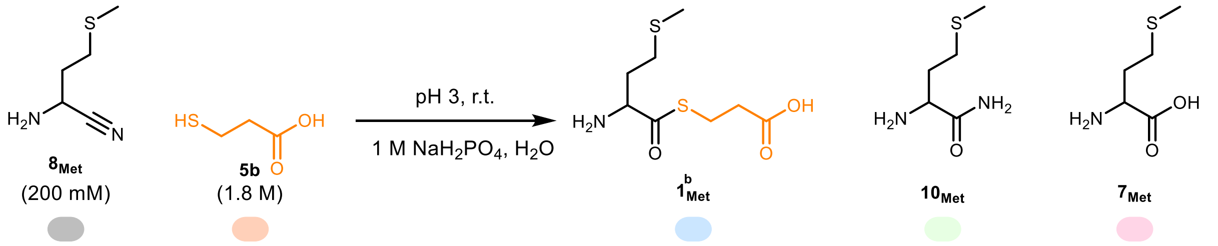

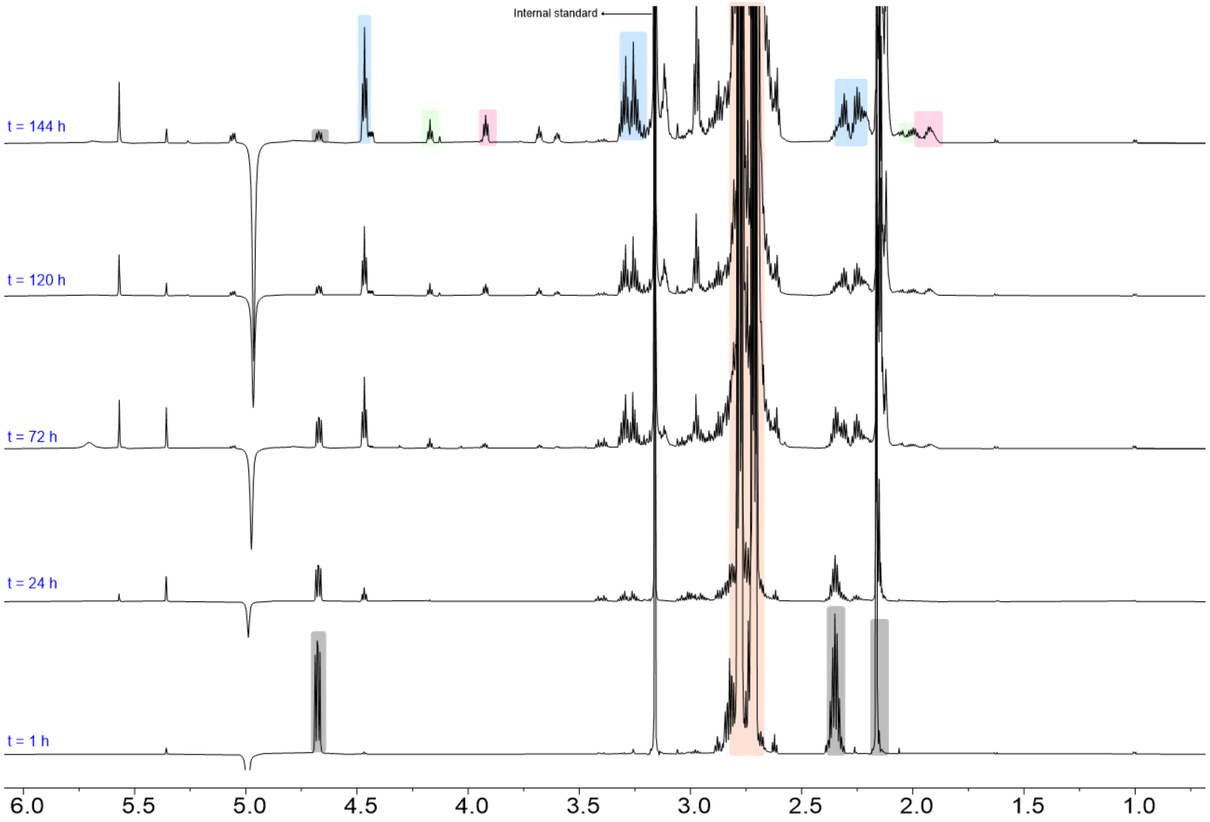

*Supplementary Figure 202: ¹H NMR (600 MHz, H_2O/D_2O 9:1, noesygppr1d, 0.9 – 6.0 ppm) spectra to show the reaction of leucine nitrile (**8_{Met}**, 200 mM) with 3-mercaptopropanoic acid (**5b**, 1.8 M) in 1 M NaH_2PO_4 buffer, with MSM (100 mM) as an internal standard at pH 3 and room temperature. Set up following General Procedure K. a, β-keto-aldehyde hydrate **58_{Met}**•H_2O = 5.57 p.p.m. singlet resonance and its precursor **47_{Met}** = 5.35 p.p.m. singlet resonance (combined yield 11%).*

¹H NMR (600 MHz, H_2O/D_2O 9:1) **1^b_{Met}** (partial assignment) : δ_H 4.47 (1H, t, *J* = 6.3 Hz, Met-α-C*H*COSCH$_2$), 3.35 – 3.22 (2H, m, COSC*H*$_2$), 2.38 – 2.19 (2H, m, C*H*$_2$SCH$_3$), 2.15 (3H, s, CH$_2$SC*H*$_3$).

10_{Met}: δ_H 4.17 (1H, t, *J* = 6.6 Hz, Met-α-C*H*CONH$_2$).

7_{Met}: δ_H 3.92 (1H, t, *J* = 7.2 Hz, Met-α-C*H*COOH).

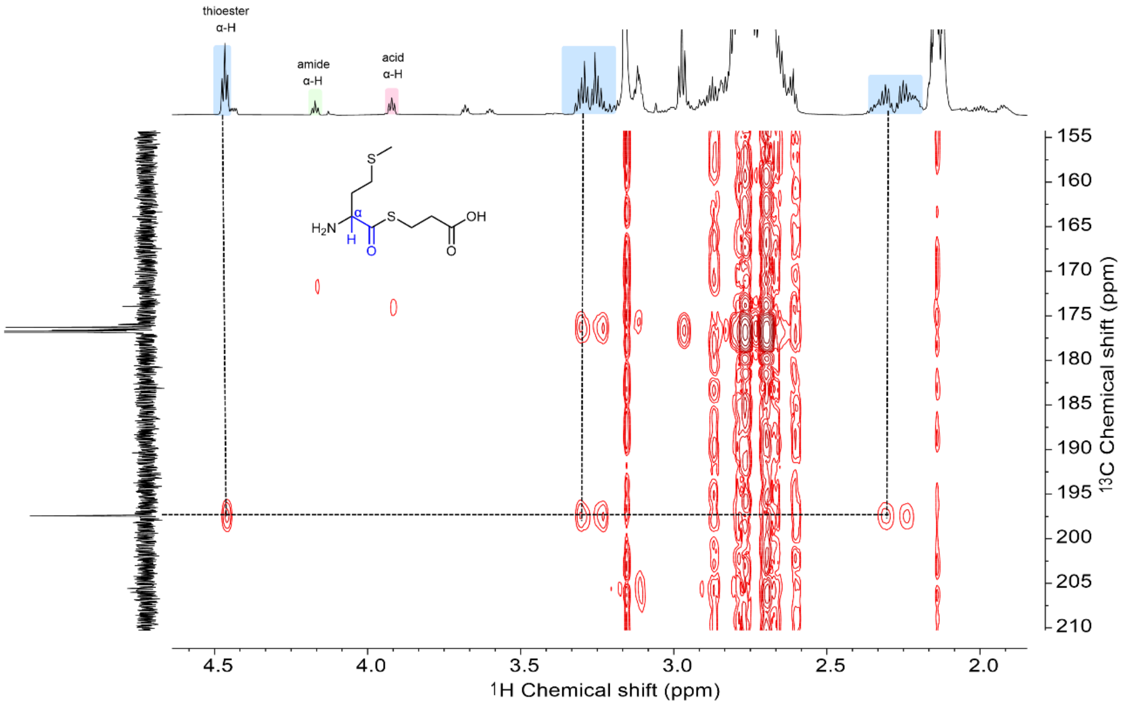

Supplementary Figure 203: ^1H–^{13}C HMBC (^1H: 600 MHz [1.3-4.5 ppm], ^{13}C: 176 MHz [163 – 207 ppm], H_2O/D_2O 9:1) spectrum showing the diagnostic ^2JCH and ^3JCH coupling of both methionine-aH and -SCH_2 in $\mathbf{1^b_{Met}}$ at 4.46 ppm and 3.29 ppm with a resonance at 197.6 ppm, which is characteristic of thioester bond formation.

*Reaction of phenylalanine nitrile **8**$_{Phe}$ with 3-mercaptopropanoic acid **5b***

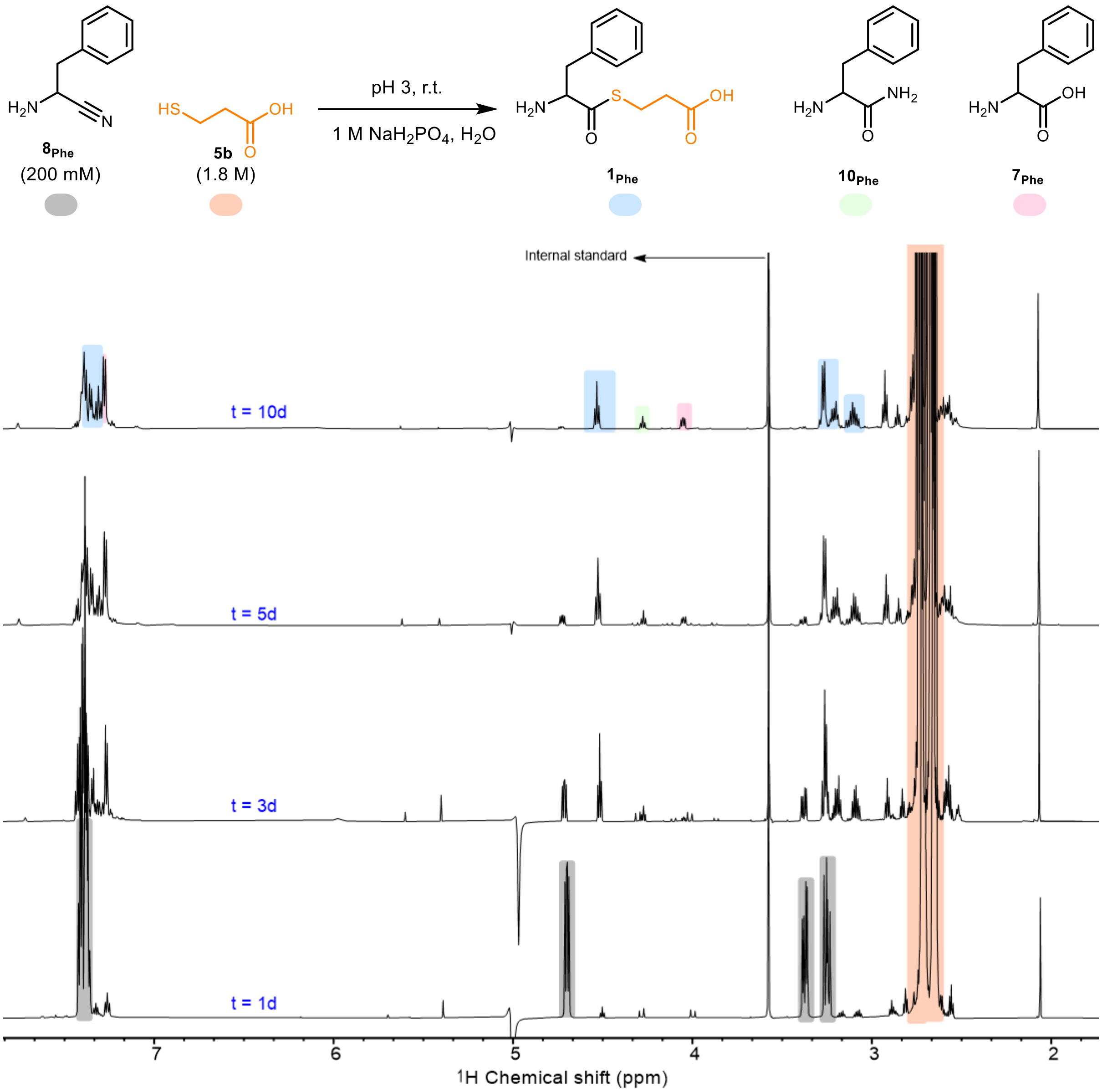

*Supplementary Figure 204. ^{1}H NMR (600 MHz, H_2O/D_2O 9:1, noesygppr1d, 1.0 – 7.4 ppm) spectra to show the reaction of phenylalanine nitrile (**8**$_{Phe}$, 200 mM) with 3-mercaptopropanoic acid (**5b**, 1.8 M) in 1 M NaH_2PO_4 buffer, with PET (10 mM) and NaOAc (10 mM) as an internal standard at pH 3 and room temperature.[7] Set up following General Procedure K.*

^{1}H NMR (600 MHz, H_2O/D_2O 9:1) $\mathbf{1}^{b}_{\mathbf{Phe}}$ (partial assignment) : δ_H 4.56 (1H, t, *J* = 7.0 Hz, Phe-α-C*H*COSCH$_2$), 3.29 (2H, d, *J* = 7.0 Hz, α-CHC*HH*-Ph), 3.27 – 3.19 (1H, m, COSC*H*H); 3.09 – 3.15 (1H, m, 1H, m, COSCH*H*).

10$_{Phe}$: δ_H 4.30 (1H, t, *J* = 7.1 Hz, Phe-α-C*H*CONH$_2$).

7$_{Phe}$: δ_H 4.08 (1H, dd, *J* = 7.8, 5.3 Hz, Phe-α-C*H*COOH).

---

[7] During the reaction Phe-resonance intensity was observed to decrease with respect to the internal standard. This was attributed to the formation of a dense yellow oil that separated from the aqueous phase. After 10 days, the reaction mixture was centrifuged, and the yellow oil dissolved in d$_6$-dimethyl sulfoxide for NMR analysis (not shown), which indicated that this oil was likely a mixture of products derived from α,β-keto-aldehyde (see Supplementary Discussion). α,β-keto-aldehyde hydrate **58**$_{Phe}$•H_2O = 5.64 p.p.m. singlet resonance and its precursor **57**$_{Phe}$ = 5.42 p.p.m. singlet resonance. Further purification, analysis and characterisation of the oil and optimization of the reaction conditions has not been undertaken.

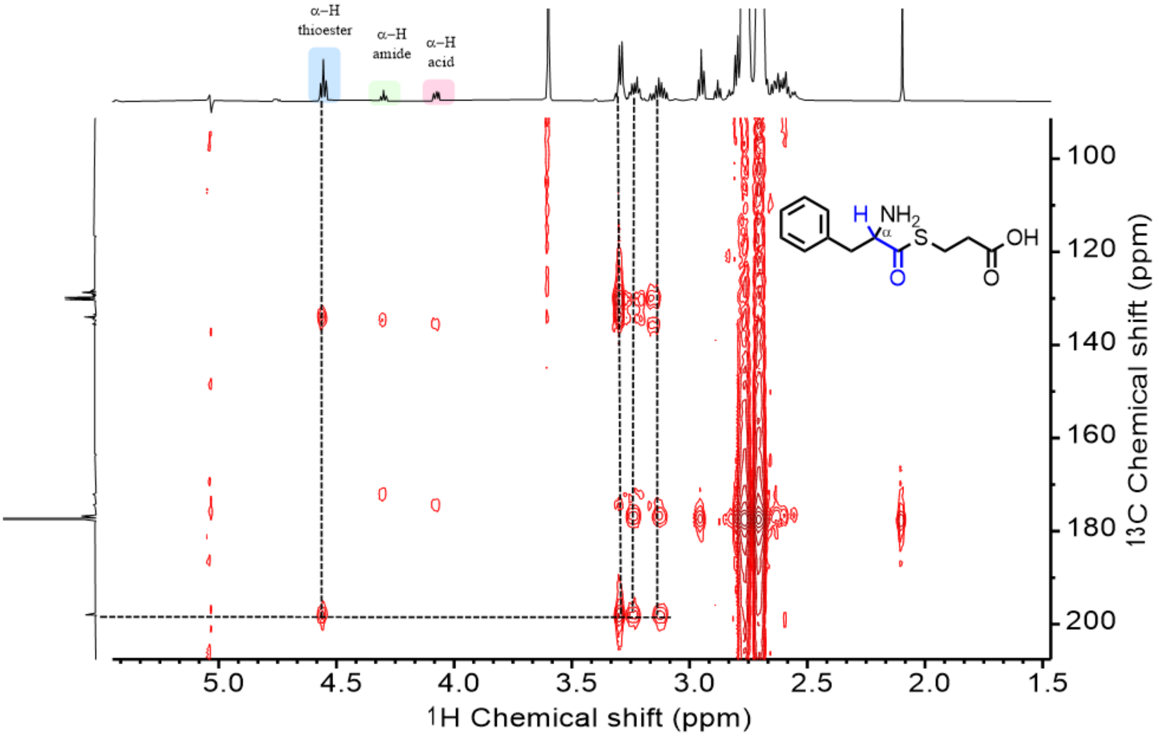

Supplementary Figure 205. ^{1}H–^{13}C HMBC (^{1}H: 600 MHz [1.5 – 4.5 ppm], ^{13}C: 176 MHz [100 – 200 ppm], H_2O/D_2O 9:1) spectrum showing the diagnostic ^{2}JCH and ^{3}JCH coupling of phenylalanine-aH, aCHCH$_2$ and d -SCH$_2$ in ***1^{b}_{Phe}*** *at 4.56 ppm, 3.12 ppm and 3.15 ppm with a resonance at 198.1 ppm, which is characteristic of thioester bond formation.*

Reaction of serine nitrile $\mathbf{8_{Ser}}$ with 3-mercaptopropanoic acid ***5b***

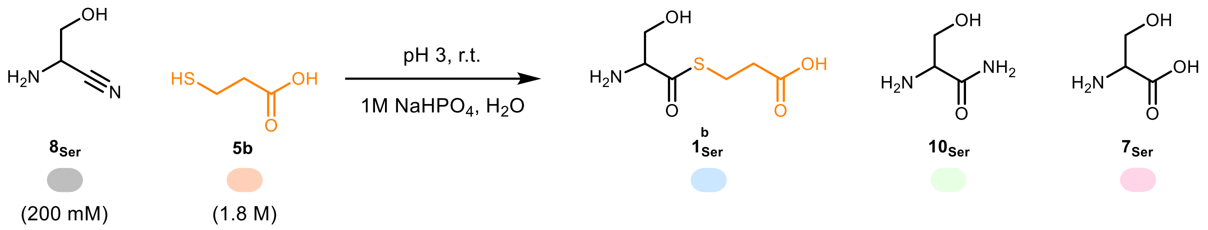

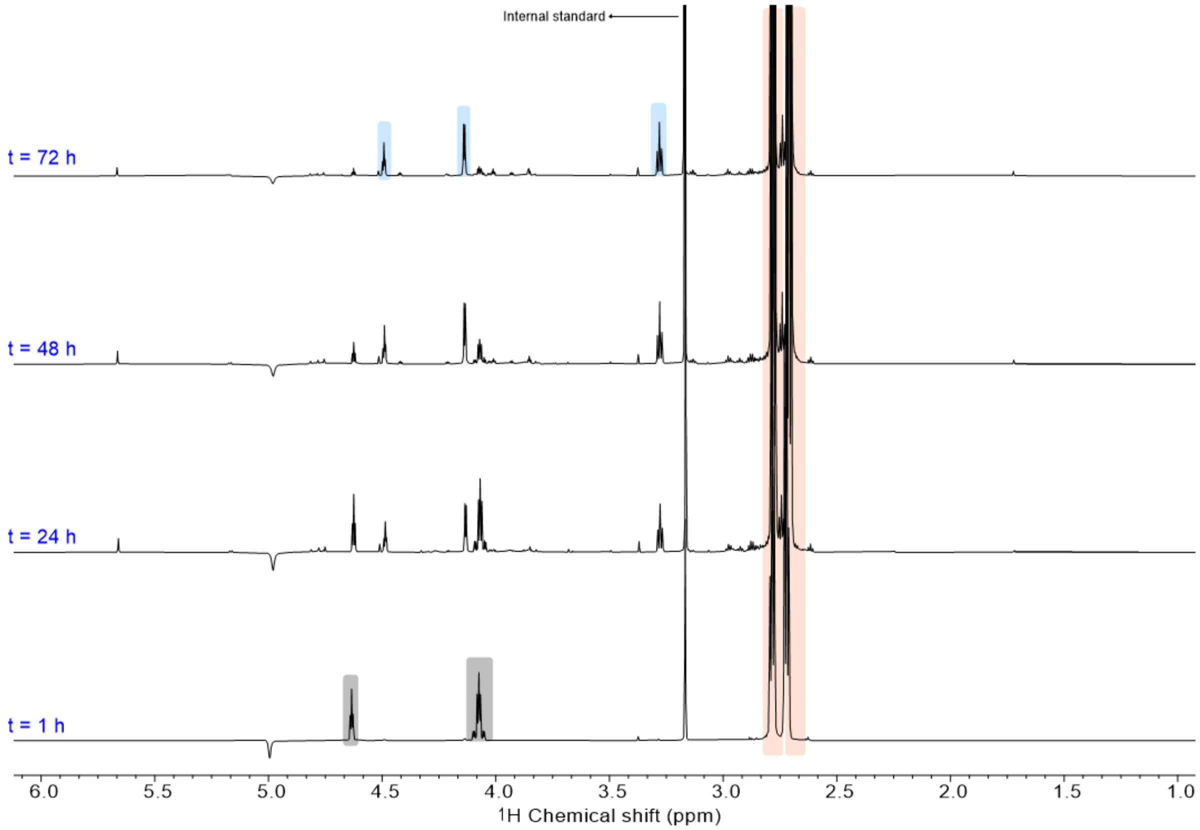

*Supplementary Figure 206. ^{1}H NMR (600 MHz, H_2O/D_2O 9:1, noesygppr1d, 1.0 – 6.0 ppm) spectra to show the reaction of serine nitrile ($\mathbf{8_{Ser}}$, 200 mM) with 3-mercaptopropanoic acid (**5b**, 1.8 M) in 1 M NaH_2PO_4 buffer, with MSM (100 mM) as an internal standard at pH 3 and room temperature. Set up following General Procedure K. α,β-keto-aldehyde hydrate $\mathbf{58_{Ser}}$•H_2O = 5.68 p.p.m. singlet resonance (4% yield).*

^{1}H NMR (600 MHz, H_2O/D_2O 9:1) $\mathbf{1^{b}_{Ser}}$ (partial assignment): δ_H 4.49 (1H, t, *J* = 4.1 Hz, Ser-α-C*H*COSCH$_2$), 4.14 (2H, d, *J* = 4.2 Hz, CHC*H*$_2$OH), 3.28 (2H, t, *J* = 6.9 Hz CHCOSC*H*$_2$).

$\mathbf{10_{Ser}}$: δ_H 4.01 (1H, t, *J* = 4.4 Hz, Ser-α-C*H*CONH$_2$).

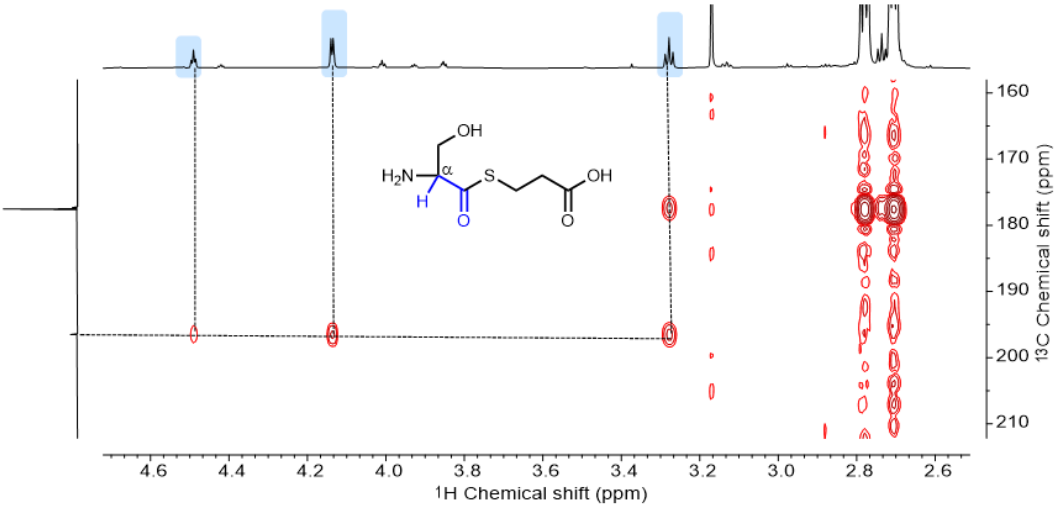

Supplementary Figure 207. ^{1}H–^{13}C HMBC (^{1}H: 600 MHz [2.5 – 4.7 ppm], ^{13}C: 176 MHz [160 – 210 ppm], H_2O/D_2O 9:1) spectrum showing the diagnostic ^{2}JCH, and ^{3}JCH coupling of both Ser-aH, and -SCH_2 in $\mathbf{1^{b}_{Ser}}$ *at 4.49 and 3.28 ppm with a resonance at 196.6 ppm, which is characteristic of thioester bond formation.*

Reaction of valine nitrile $\mathbf{8_{Val}}$ *with 3-mercaptopropanoic acid* ***5b***

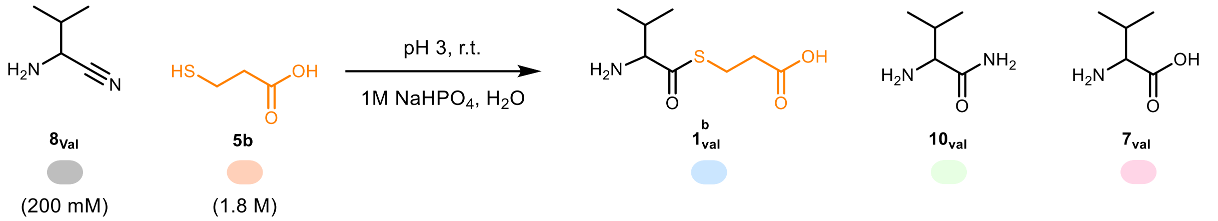

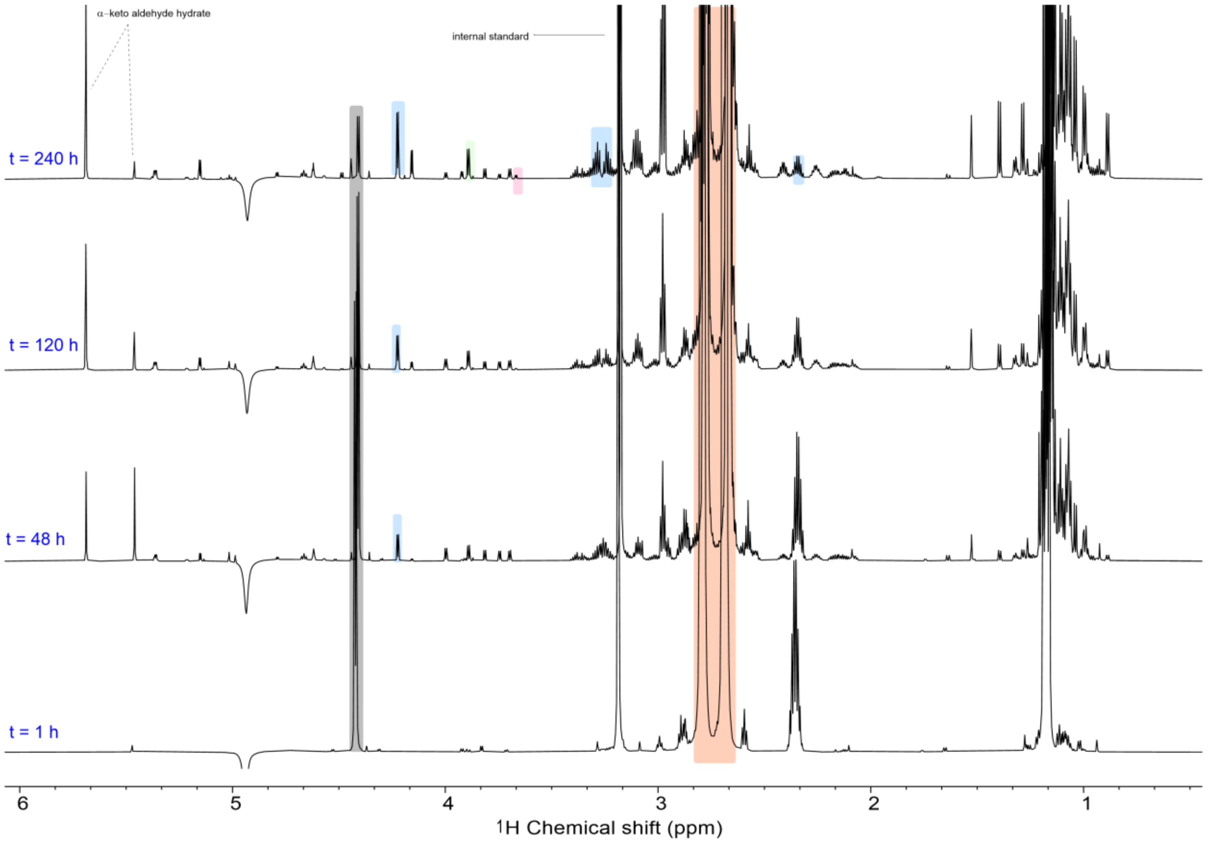

Supplementary Figure 208. ^{1}*H NMR (600 MHz,* H_2O/D_2O *9:1, noesygppr1d, 1.0 – 6.0 ppm) spectra to show the reaction of valine nitrile (*$\mathbf{8_{Val}}$*, 200 mM) with 3-mercaptopropanoic acid (**5b**, 1.8 M) in 1 M* NaH_2PO_4 *buffer, with PET (10 mM) and NaOAc (10 mM) as an internal standard at pH 3 and room temperature. Set up following General Procedure K.* α,β*-Keto-aldehyde hydrate* $\mathbf{58_{Val}}$*•*H_2O *= 5.51 p.p.m. singlet resonance and its precursor* $\mathbf{57_{Val}}$ *= 5.29 p.p.m. singlet resonance (Combined yield 25%). In addition to di-carbonyl **58**, four small cyanohydrin resonances are observed (see supplementary Figure 207;* ^{1}*H 3.5 – 4.0;* ^{13}*C 115.0 – 125.0). These are tentatively assigned to be the diastereomeric cyanohydrins of di-carbonyl **58**.*

^{1}H NMR (600 MHz, H_2O/D_2O 9:1) $\mathbf{1^b_{Val}}$ (partial assignment) : δ_H 4.22 (1H, d, J = 4.6 Hz, Val-α-C*H*COSCH$_2$), 3.30 – 3.20 (2H, m, COSC*H*$_2$).

$\mathbf{10_{val}}$ (partial assignment) : δ_H 3.88 (1H, d, J = 4.6 Hz, Val-α- C*H*CONH$_2$).

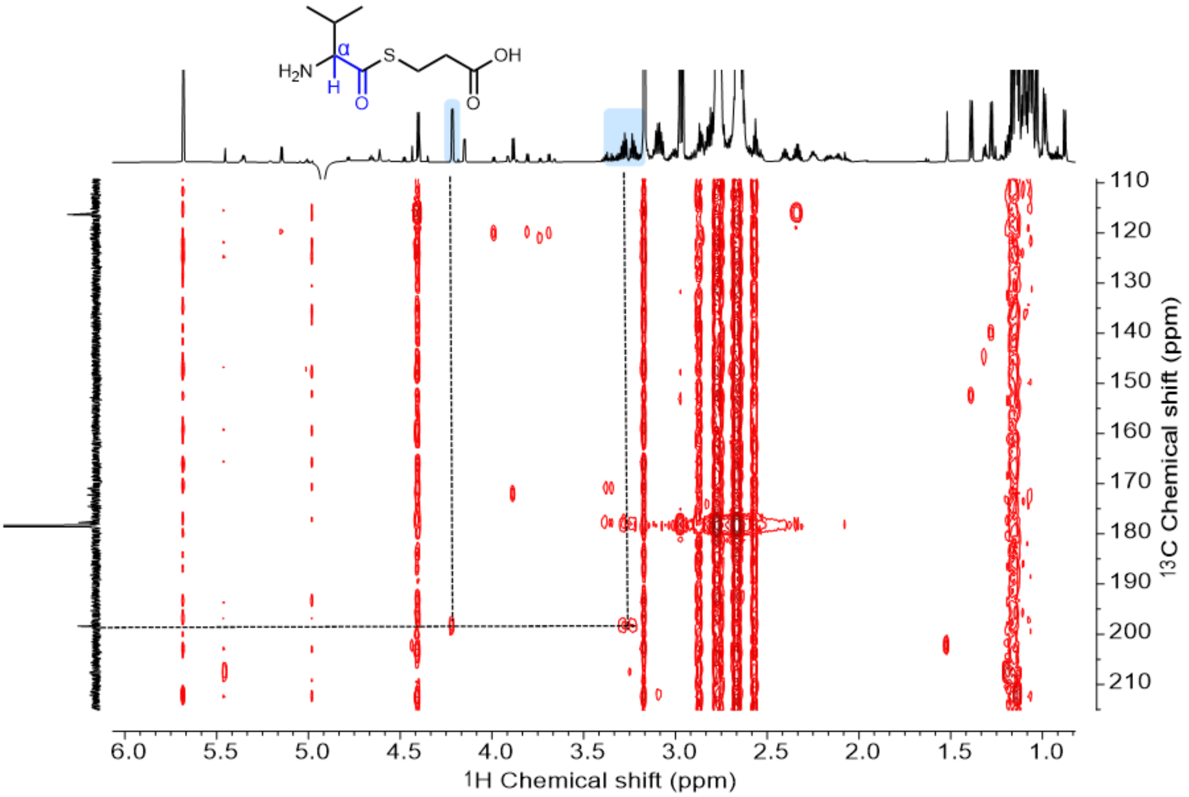

Supplementary Figure 209. ^{1}H–^{13}C HMBC (^{1}H: 600 MHz [1.5-4.5 ppm], ^{13}C: 176 MHz [160-200 ppm], H_2O/D_2O 9:1) spectrum showing the diagnostic ^{2}JCH and ^{3}JCH coupling of valine-aH and -SCH_2 in ***1^{b}_{Val}*** *at 4.22 ppm and 3.25 ppm with a resonance at 198.6 ppm, which is characteristic of thioester bond formation.*

(9H-Fluoren-9-yl)methyl 3,4-dihydropyridine-1(2H)-carboxylate

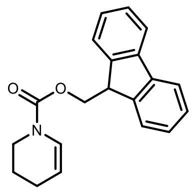

To (9H-fluoren-9-yl)methyl (5-hydroxypentyl)carbamate (4.29 g, 13.0 mmol) was added (2,2,6,6-tetramethylpiperidin-1-yl)oxyl (20.6 mg, 0.13 mmol) in CH_2Cl_2 (130 mL) and potassium bromide (155 mg, 1.30 mmol) in H_2O (35 mL) at 0 °C. 1 M aq. NaOCl pre-adjusted to pH 9.2 with $NaHCO_3$ (0.5 M) was added dropwise. The reaction mixture was stirred for 1 hour before $Na_2S_2O_3$ (3.3 g, 20.9 mmol) and $NaHCO_3$ (520 mg, 6.19 mmol) in H_2O (100 mL) were added. The organic layer was separated, washed with H_2O (3 × 80 mL), dried over anhydrous $MgSO_4$, filtered and concentrated *in vacuo* to afford the title compound (3.97 g, 0.13 mmol, quantitative) as a colourless oil that crystallised on standing. (9H-fluoren-9-yl) methyl 3,4-dihydropyridine-1(2H)-carboxylate was used without further purification. **^{1}H NMR** (500 MHz, $CDCl_3$) δ_H 7.79 (d, *J* = 7.6 Hz, 2H, $(C_6H_4)_2$), 7.60 (d, J = 7.6 Hz, $(C_6H_4)_2$), 7.42 (t, *J* = 7.4 Hz, 2H, $(C_6H_4)_2$), 7.34 (t, *J* = 7.4 Hz, 2H, $(C_6H_4)_2$), 6.82–6.90 (m, 1H, (C1)–H), 4.93–5.02 (m, 1H, (C2)–H), 4.44–4.47 (m, 2H, OC**H**$_2$CH$(C_6H_4)_2$), 4.29 (t, *J* = 7.1 Hz, 1H, OCH$_2$C**H**$(C_6H_4)_2$), 3.63 – 3.68 (m, 2H, (C5)–H_2), 2.08 (br s, 2H, (C3)–H_2), 1.83 – 1.90 (m, 2H, (C4)–H_2). **^{13}C NMR** (101 MHz, $CDCl_3$) δ_C 153.7, 153.3, 144.0, 141.5, 127.9, 127.2, 125.4, 125.2, 125.1, 124.8, 120.2, 107.1, 107.0, 68.0, 67.8, 47.3, 42.5, 42.4, 21.8, 21.7, 21.6, 21.4. **HRMS-ESI** $[M+H]^+$ calc. for $C_{20}H_{20}NO_2^+$ 306.1489; obs. 306.1486. Consistent with literature data.[23]

ε-*N*-(Fmoc)-Lysine nitrile

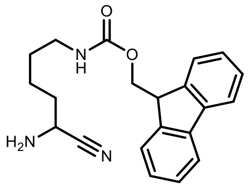

ε-N-(Fmoc)-lysine nitrile

(9H-Fluoren-9-yl)methyl 3,4-dihydropyridine-1(2H)-carboxylate (1.36 g, 4.45 mmol) and $NaHSO_3$ (1.39 g, 13.4 mmol) were dissolved in MeOH/H_2O (2:1, 100 mL). NH_4Cl (4.05 g, 75.7 mmol) and NaCN (920 mg, 18.8 mmol) were added, the solution adjusted to pH ~9.5 with 1 M NaOH and the reaction mixture was heated at 60 °C for 8 hours. The reaction mixture was concentrated *in-vacuo* and extracted with Et_2O (3 × 50 mL). The combined organic extracts were concentrated to dryness *in-vacuo* and purified by column chromatography (gradient, 40:60 petroleum ether/EtOAc) to afford ε-N-(Fmoc)-lysine nitrile (440 mg, 1.26 mmol, 28%) as a white powder. **^{1}H NMR** (700 MHz, $CDCl_3$) δ_H 7.78 (2H, d, *J* = 7.5 Hz, $(C_6H_4)_2$), 7.60 (2H, d, *J* = 7.5 Hz, $(C_6H_4)_2$), 7.41 (2H, t, *J* = 7.4 Hz, $(C_6H_4)_2$), 7.32 (2H, t, *J* = 7.4 Hz, $(C_6H_4)_2$), 4.87 (1H, s, NH), 4.42 (2H, d, *J* = 6.8 Hz, OC**H**$_2$CH$(C_6H_4)_2$), 4.22 (1H, t, *J* = 6.8 Hz, OCH$_2$C**H**$(C_6H_4)_2$), 3.68 (1H, t, *J* = 7.0 Hz, (C2)–H), 3.21 (2H, m, (C6)–H_2), 1.30–1.80 (6H, overlapping m, (C3)–H_2 + (C4)–H_2 + (C5)–H_2). **^{13}C NMR** (176 MHz, $CDCl_3$) δ_C 156.4 (**C**OOCH$_2$CH$(C_6H_4)_2$), 143.9 ($(C_6H_4)_2$), 141.3 ($(C_6H_4)_2$), 127.6 ($(C_6H_4)_2$), 127.0 ($(C_6H_4)_2$), 125.0 ($(C_6H_4)_2$), 121.9 (C1), 119.9 ($(C_6H_4)_2$), 66.5 (O**C**H$_2$CH$(C_6H_4)_2$), 47.2 (OCH$_2$**C**H$(C_6H_4)_2$), 43.1 (C2/C6), 40.5 (C2/C6), 34.7 (C3), 29.3 (C5), 22.5 (C4). **HRMS-ESI** $[M+H]^+$ calc. for $C_{21}H_{24}N_3O_2^+$ 350.1863; obs. 350.1858. **IR** (solid, cm^{-1}): 3339, 1710, 1686, 1534. **R$_f$** = 0.25 in 100% EtOAc. **m.p.** 94 °C.

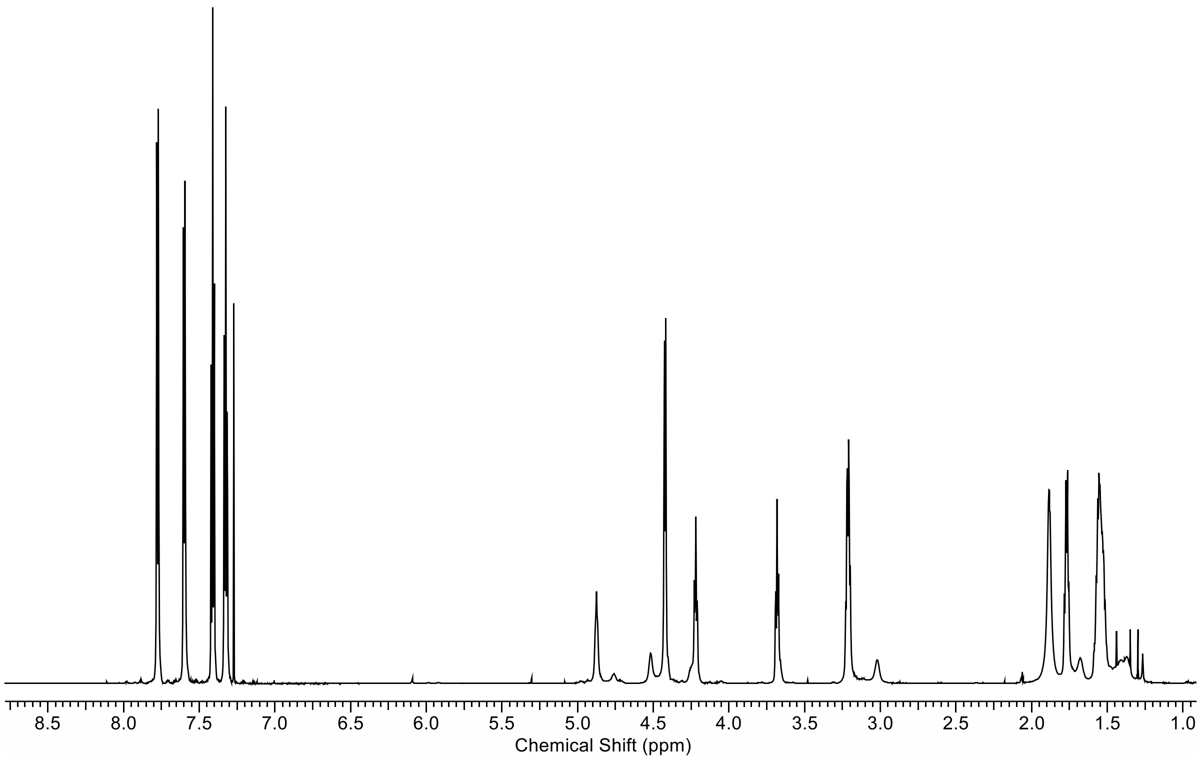

Supplementary Figure 210. ^{1}H NMR (700 MHz, $CDCl_3$, 1.0 – 9.0 ppm) spectrum of ***ε-N-(Fmoc)-lysine nitrile***.

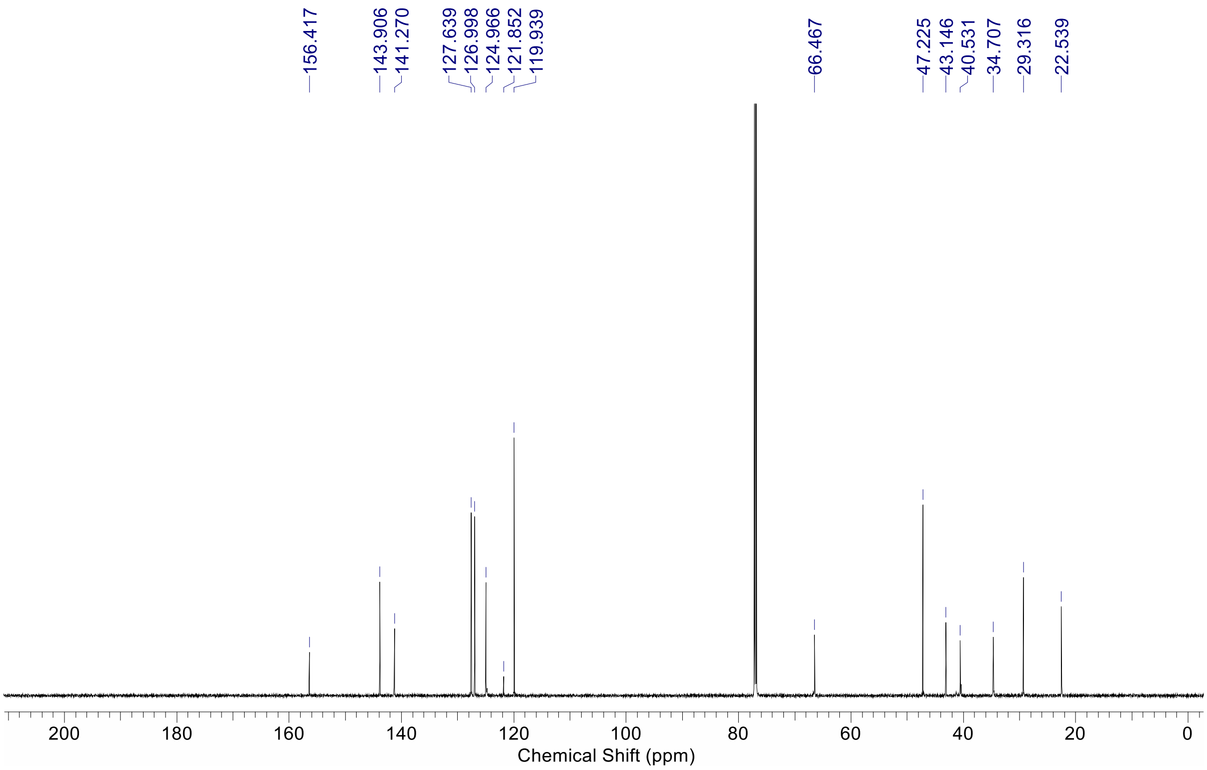

Supplementary Figure 211. ^{13}C NMR (176 MHz, $CDCl_3$, 0 – 210 ppm) spectrum of ***ε-N-(Fmoc)-lysine nitrile***.

Lysine nitrile (8_{Lys})

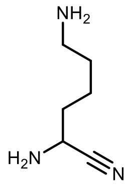

2_{Lys}

To **ε-*N*-(Fmoc)-lysine nitrile** (120 mg, 0.34 mmol) in CH_2Cl_2 (5.0 mL) was added *n*-octylamine (1.00 mL, 6.30 mmol). The reaction mixture was stirred at room temperature and monitored by TLC (ninhydrin stain). After 2 hours, volatiles were removed *in vacuo* and the reaction mixture redissolved in Et_2O/H_2O (40 mL, 1:1). The aqueous layer was extracted with Et_2O (5 × 10 mL). Basification of the aqueous layer to pH ~ 10 was followed by further extraction with Et_2O (3 × 10 mL). The residual Et_2O was then removed *in vacuo* to afford lysine nitrile (**2_{Lys}**) (0.16 mmol, 48%) which was adjusted to pH 2 with 4 M HCl and stored as a stock solution for further use. **^{1}H NMR** (700 MHz, D_2O) δ_H 4.51 (1H, dd, *J* = 6.9, 6.15 Hz, (C2)–H), 2.99 (2H, t, *J* = 7.7 Hz, (C6)–H_2), 1.99–2.03 (m, 2H, (C3)–H_2), 1.70–1.75 (2H, m, (C5)–H_2), 1.52–1.64 (2H, m, (C4)–H_2). **^{13}C NMR** (126 MHz, D_2O) δ_C 123.3 (C1), 43.1 (C2/C6), 40.0 (C2/C6), 34.1 (C3), 27.5 (C5), 22.6 (C4). **HRMS-ESI** $[M+H]^+$ calc. for $C_6H_{14}N_3O^+$ 128.1182; obs. 128.1183. Consistent with literature data.[24]

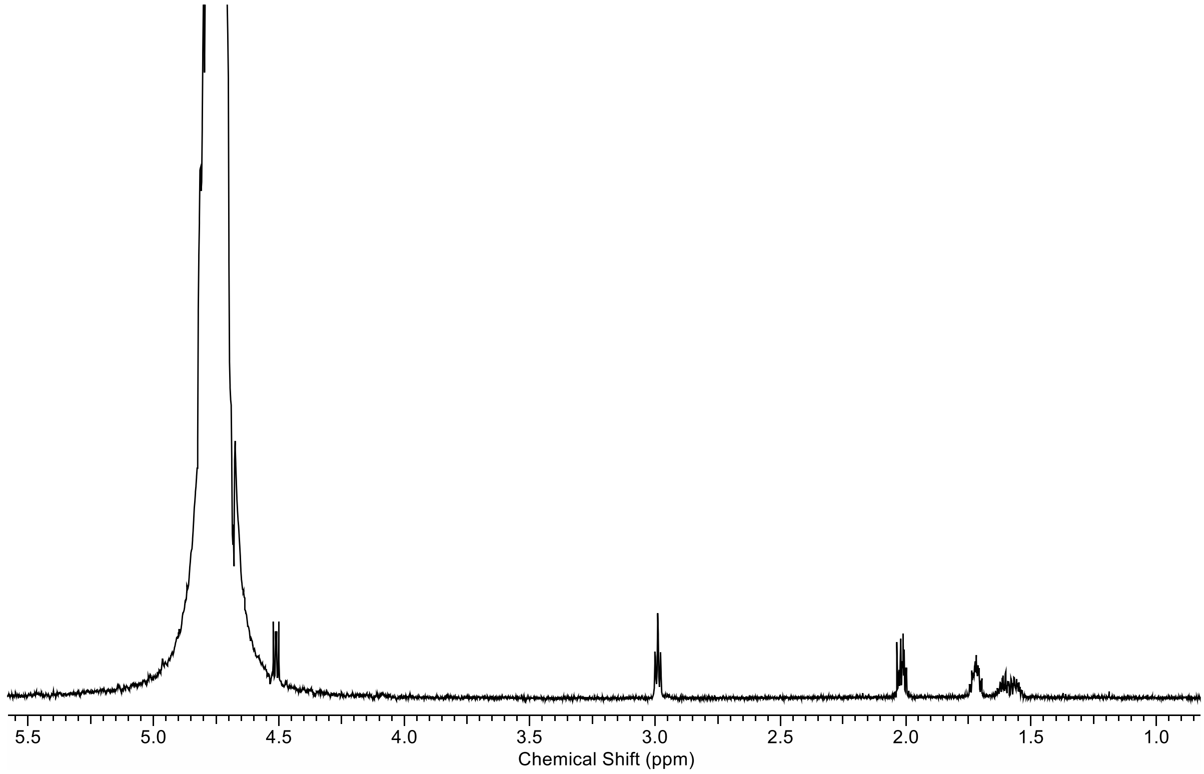

*Supplementary Figure 212. ^{1}H NMR (700 MHz, D_2O, 1.0 – 5.5 ppm) spectrum of **8_{Lys}** at pD 2.*

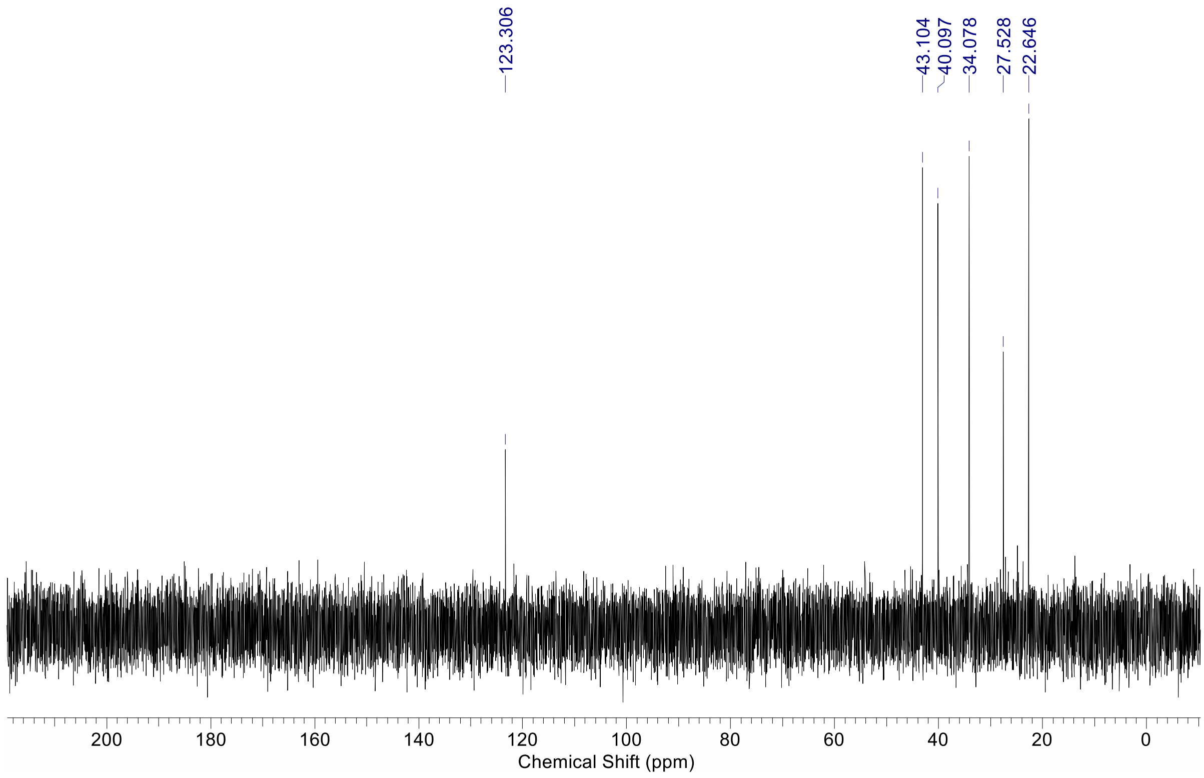

*Supplementary Figure 213. ¹³C NMR (126 MHz, D_2O, 0 – 220 ppm) spectrum of **8_{Lys}**.*

Reaction of lysine nitrile ***8*** $_{Lys}$ *with 2-mercaptopropanoic acid* ***5b***

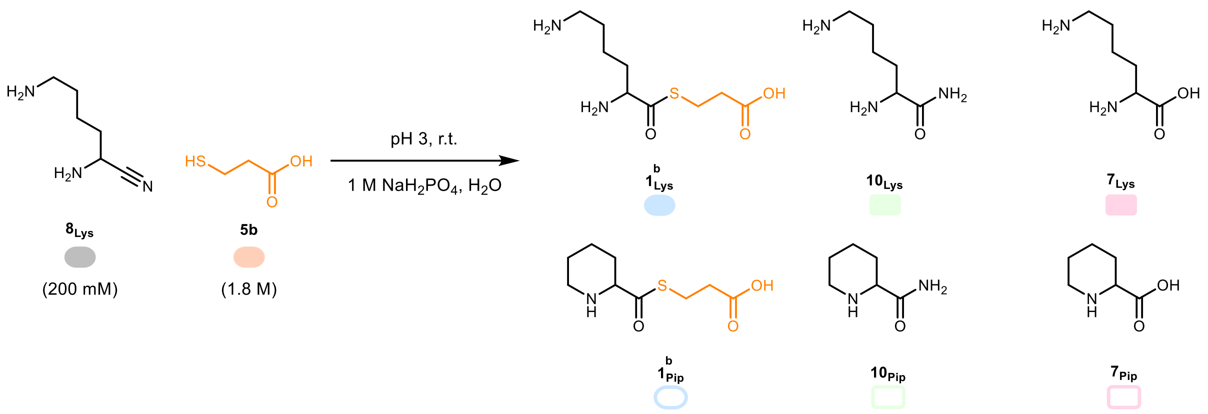

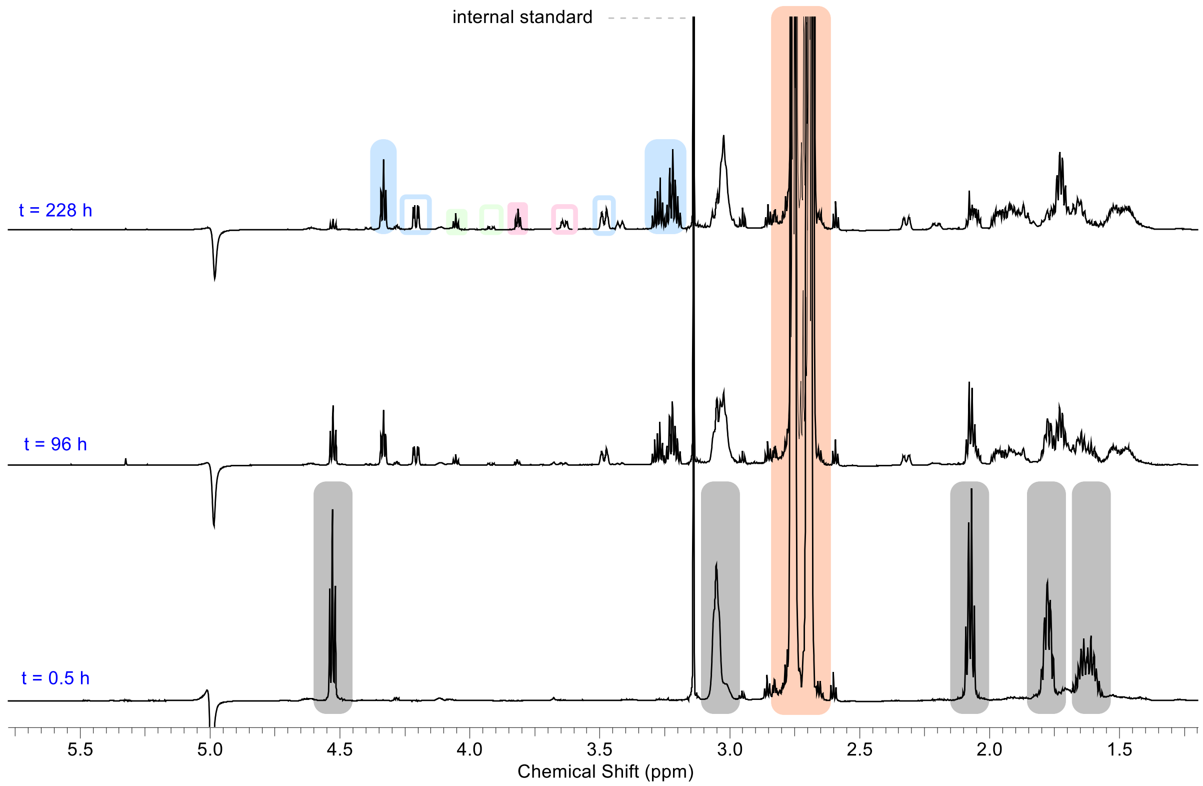

Supplementary Figure 214. *^{1}H NMR (700 MHz, H_2O/D_2O 98:2, noesygppr1d, 1.5 – 5.5 ppm) spectra to show the reaction of* ***8*** $_{Lys}$ *(200 mM) with 3-mercaptopropanoic acid (**5b**, 1.8 M) in 1 M NaH_2PO_4 buffer at pH 3 and room temperature with MSM (28 mM) as an internal standard. Set up following General Procedure K.* ***7*** $_{Lys}$ *and* ***10*** $_{Lys}$ *were confirmed by spiking with authentic standards.*

1^{b}_{Lys}: **^{1}H NMR** (700 MHz, 98:2 H_2O/D_2O, partial assignment) δ_H 4.33 (1H, t, J = 6.2 Hz, Lys-(C2)–H), 3.25–3.30 (1H, m, Lys-SC***H***$_2$CH$_2$CO$_2$H), 3.19–3.24 (1H, m, Lys-SC***H'***$_2$CH$_2$CO$_2$H). **^{13}C NMR** (176 MHz, 98:2 H_2O/D_2O, partial assignment) δ_C 198.4 (Lys-C1), 59.4 (Lys-C2).

1^{b}_{Pip}: **^{1}H NMR** (700 MHz, 98:2 H_2O/D_2O, partial assignment) δ_H 4.21 (1H, dd, J = 11.8, 3.4 Hz, (C2)–H), 2.32 (1H, dd, J = 14.3, 2.6 Hz, (C3)–H). **^{13}C NMR** (176 MHz, 98:2 H_2O/D_2O, partial assignment) δ_C 198.3 (C1), 63.7 (C2).

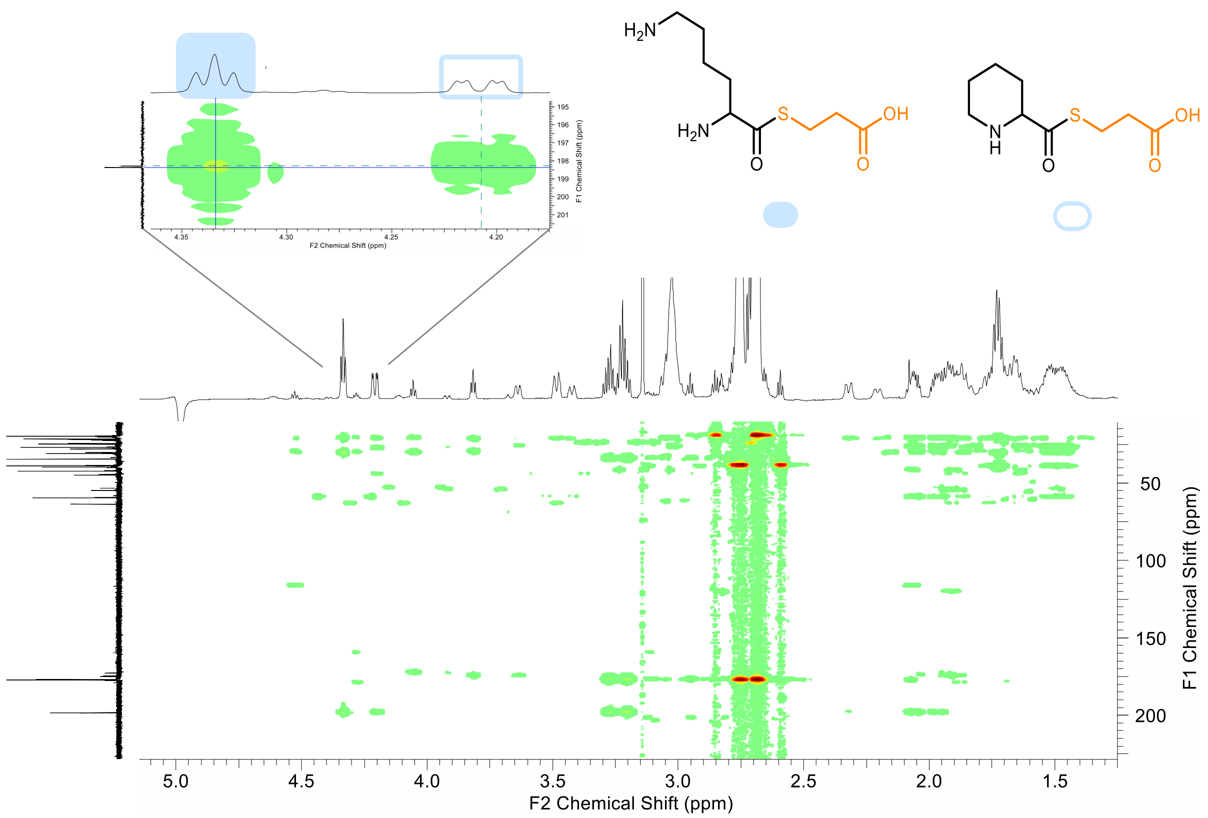

Supplementary Figure 215. ^{1}H– ^{13}C HMBC (^{1}H: 700 MHz [1.5 – 5.0 ppm], ^{13}C: 176 MHz [130-230 ppm], H_2O/D_2O 95:5) spectrum for the reaction of $\mathbf{8_{Lys}}$ (200 mM) with 3-mercaptopropionic acid (**5b**, 1.8 M) in 1 M NaH_2PO_4 buffer at pH 3 with MSM (28 mM) as an internal standard, showing the diagnostic coupling of the thioester signal in $\mathbf{1^{b}_{Lys}}$ at 198 ppm with a signal at 4.33 ppm (Lys-aH, $^{2}J_{CH}$) and the diagnostic coupling of the thioester signal in $\mathbf{1^{b}_{Pip}}$ at 198 ppm with a signal at 4.21 ppm (aH, $^{2}J_{CH}$).

Reaction of pipecolic nitrile $\mathbf{8_{Pip}}$ with 3-mercaptopropanoic acid ***5b***

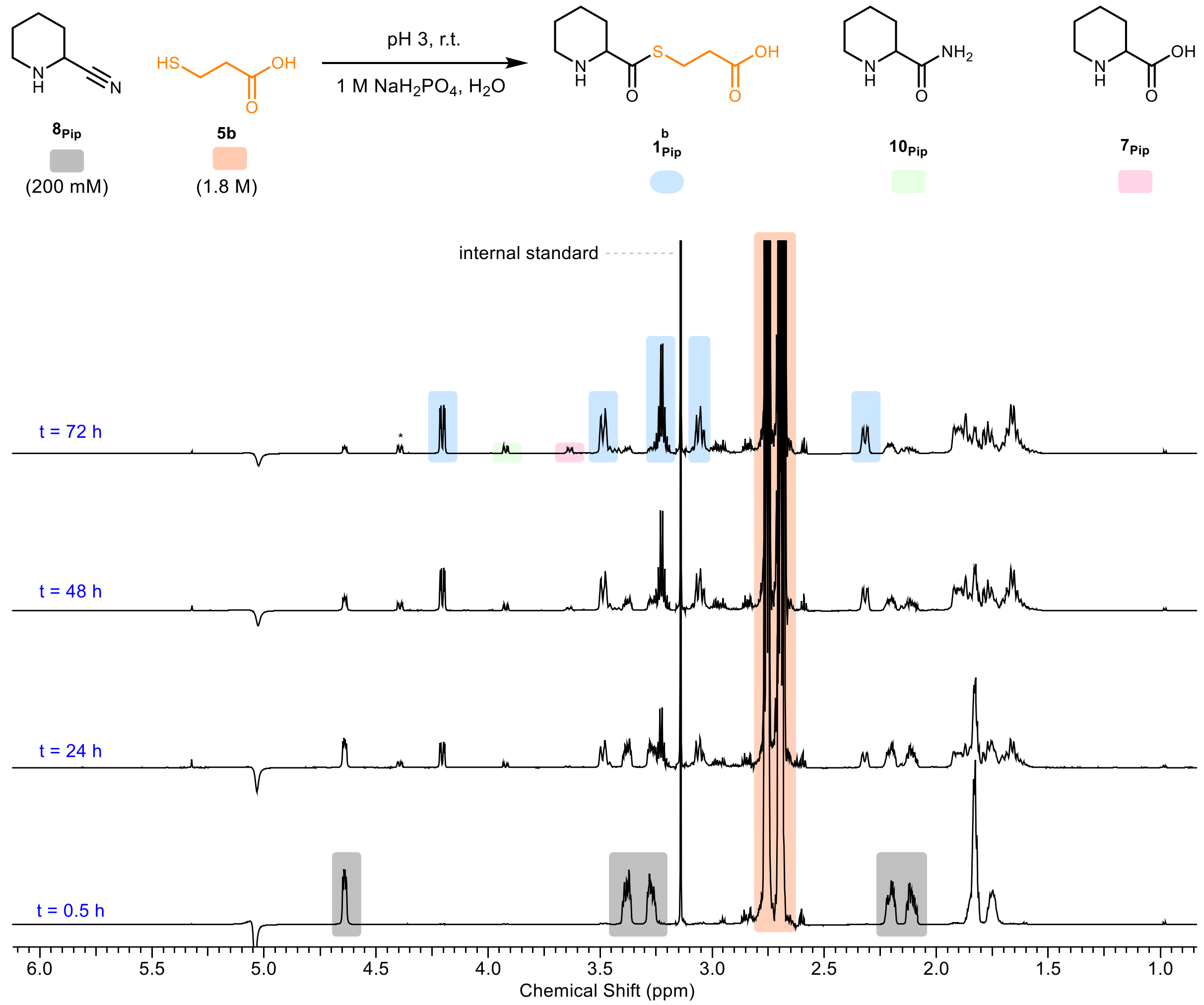

Supplementary Figure 216. ^{1}H NMR (700 MHz, H_2O/D_2O 98:2, noesygppr1d, 1.0 – 6.0 ppm) spectra to show the reaction of $\mathbf{8_{Pip}}$ *(200 mM) with 3-mercaptopropanoic acid (**5b**, 1.8 M) in 1 M NaH_2PO_4 buffer at pH 3 and room temperature with MSM (36mM) as an internal standard. Set up following General Procedure K. * Tentatively assigned as 2-(3-mercaptopropionic acid)piperidine, formed via addition of **5b** to the imine product of the retro-Strecker reaction of piperidine 2-carbonitrile.*

$\mathbf{1^{b}_{Pip}}$: **^{1}H NMR** (700 MHz, 98:2 H_2O/D_2O, partial assignment) δ_H 4.21 (dd, *J* = 11.8, 3.4 Hz, 1H, (C2)–H), 3.47–3.50 (m, 1H, (C5)–H), 3.23 (2H, CHCOSC*H*$_2$), 3.03–3.08 (m, 1H, (C5)–H'), 2.32 (dd, *J* = 14.3, 2.6 Hz, 1H, (C3)–H).

^{13}C NMR (176 MHz, 98:2 H_2O/D_2O, partial assignment) δ_C 198.3 (C1), 63.7 (C2).

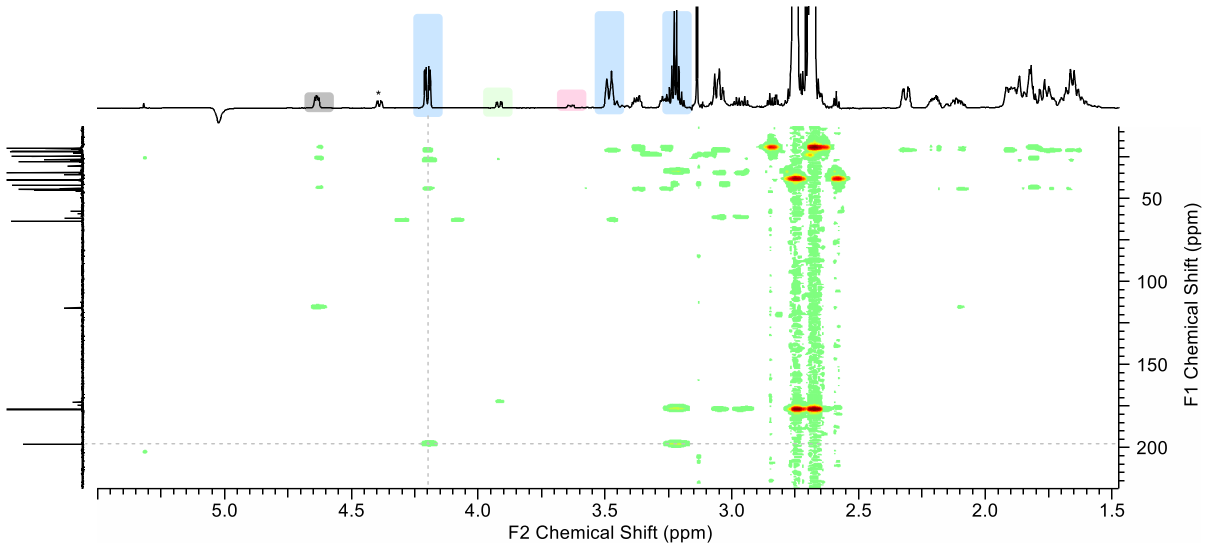

Supplementary Figure 217. ^{1}H– ^{13}C *HMBC (*^{1}*H: 700 MHz [1.5 – 5.5 ppm],* ^{13}*C: 176 MHz [10-220 ppm],* H_2O/D_2O *98:2) spectrum for the reaction of piperidine 2-carbonitrile (**8_{Pip}**, 200 mM) with 3-mercaptopropionic acid (**5b**, 1.8 M) in 1 M* NaH_2PO_4 *buffer at pH 3 with MSM (28 mM) as an internal standard, showing the diagnostic coupling of the thioester signal in **1^b_{Pip}** at 198 ppm with a signal at 4.21 ppm (Pip-aH,* $^2J_{CH}$*).*

*Reaction of arginine nitrile **8**$_{Arg}$ with 3-mercaptopropanoic acid **5b***

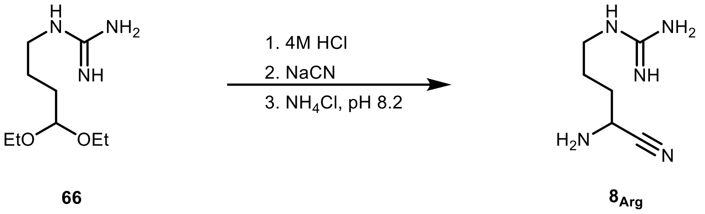

Arginine nitrile (**8**$_{Arg}$) was prepared as a solution from 4-guanidinobutyraldehyde diethylacetal (**66**), which was obtained following the procedure of Patel et al.[25] **66** (465 mg, 1.94 mmol) was dissolved in 4 M HCl (2 mL) and the reaction mixture stirred for 20 min. An iron (II) sulfate trap and bubbler were affixed, and NaCN (95.1 mg, 1.94 mmol) dissolved in water (1 mL) was added dropwise. The reaction mixture was adjusted to pH 6 and NH_4Cl (615 mg, 11.5 mmol) was added. The solution was then adjusted to pH 8.2 and the reaction mixture stirred for 3 days at room temperature. The reaction mixture was concentrated to remove ethanol and dissolved in degassed water to obtain an arginine nitrile (**8**$_{Arg}$) solution (165 mM) which was used immediately, without further purification, for thioester synthesis.

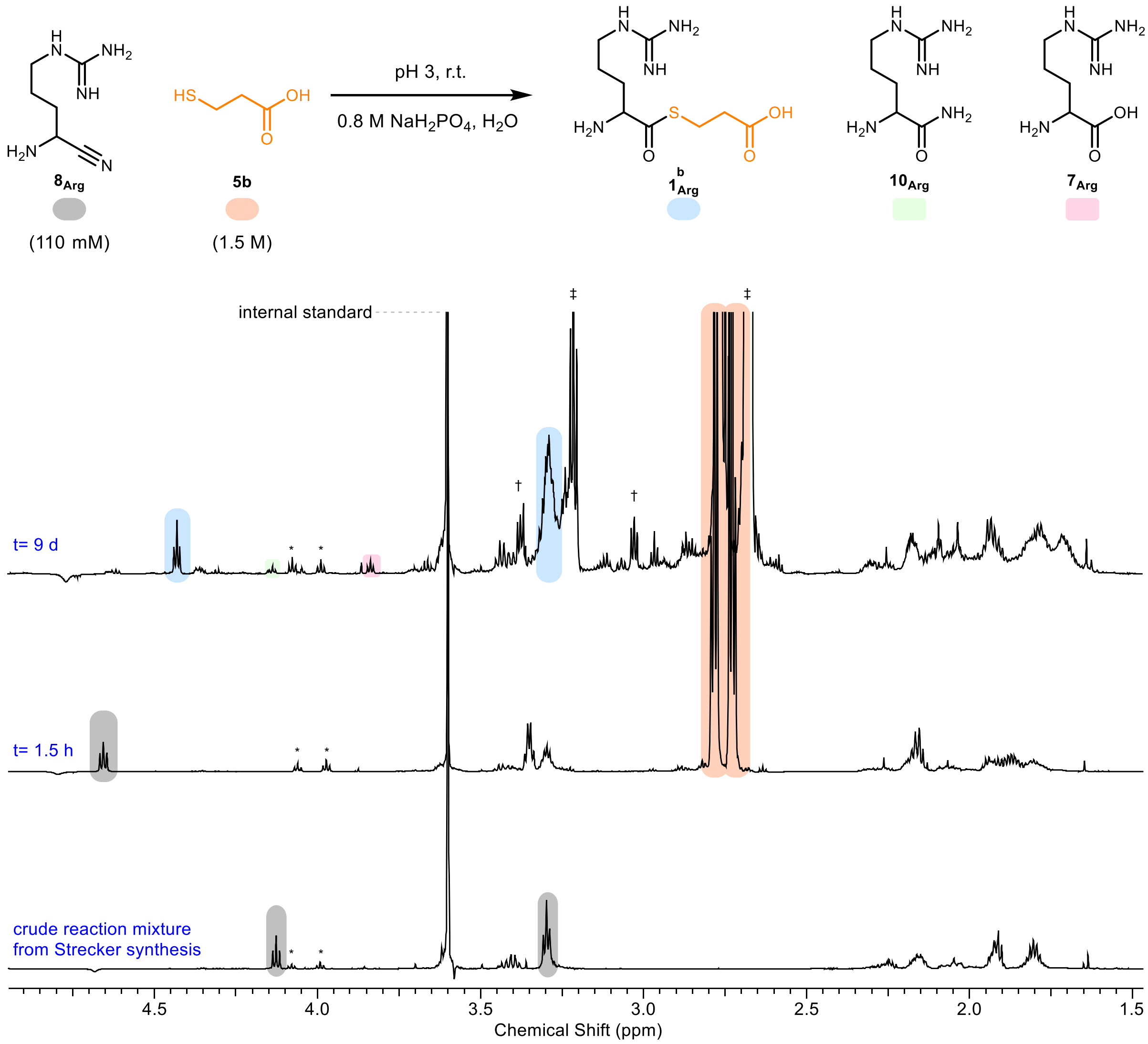

*Supplementary Figure 218. ¹H NMR (700 MHz, H_2O/D_2O 98:2, noesygppr1d, 1.5 – 5.0 ppm) spectra to show the reaction of arginine nitrile (**8**$_{Arg}$, 110 mM), present in a crude mixture from its Strecker synthesis, with 3-mercaptopropanoic acid (**5b**, 1.5 M) in 0.8 M NaH_2PO_4 buffer at pH 3 and room temperature with PET (36 mM) as an internal standard. Set up following General Procedure K. * Tentatively assigned as 1,1'-(azanediylbis(4-cyanobutane-4,1-diyl))diguanidine (N#CC(NC(C#N)CCCNC(N)=N)CCCNC(N)=N), a bis-nitrile Strecker by-product, formed*

*during the Strecker synthesis of arginine nitrile (**8**$_{Arg}$). † = S-formyl and ‡ = S-formimidyl derivatives of 3-mercaptopropionic acid **5b**, respectively – these are formed from the reaction of **5b** with HCN present in the crude Strecker reaction mixture.*

1$^{b}_{Arg}$: **^{1}H NMR** (700 MHz, 98:2 H_2O/D_2O, partial assignment) δ_H 4.43 (1H, t, J = 6.2 Hz, (C2)–H). **^{13}C NMR** (176 MHz, 98:2 H_2O/D_2O, partial assignment) δ_C 198.5 (C1).

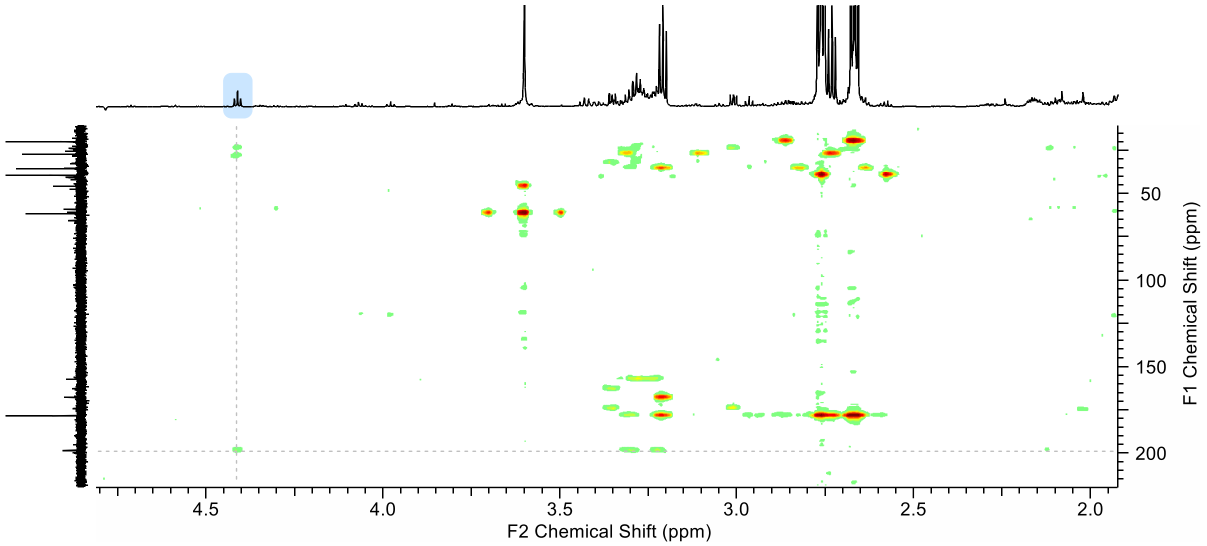

*Supplementary Figure 219. ^{1}H– ^{13}C HMBC (^{1}H: 700 MHz [2.0 – 5.0 ppm], ^{13}C: 176 MHz [10-220 ppm], H_2O/D_2O 98:2) spectrum for the reaction of arginine nitrile (**8**$_{Arg}$, 110 mM) with 3-mercaptopropionic acid (**5b**, 1.5 M) in 0.8 M NaH_2PO_4 buffer at pH 3 with PET (36 mM) as an internal standard, showing the diagnostic coupling of the thioester signal in **1**$^{b}_{Arg}$ at 199.0 ppm with a signal at 4.43 ppm (Arg-aH, $^2J_{CH}$).*

*Competition reaction of alanine nitrile **8**$_{Ala}$ and N-acetylated alanine nitrile **38**$_{Ala}$ with 3-mercaptopropanoic acid **5b***

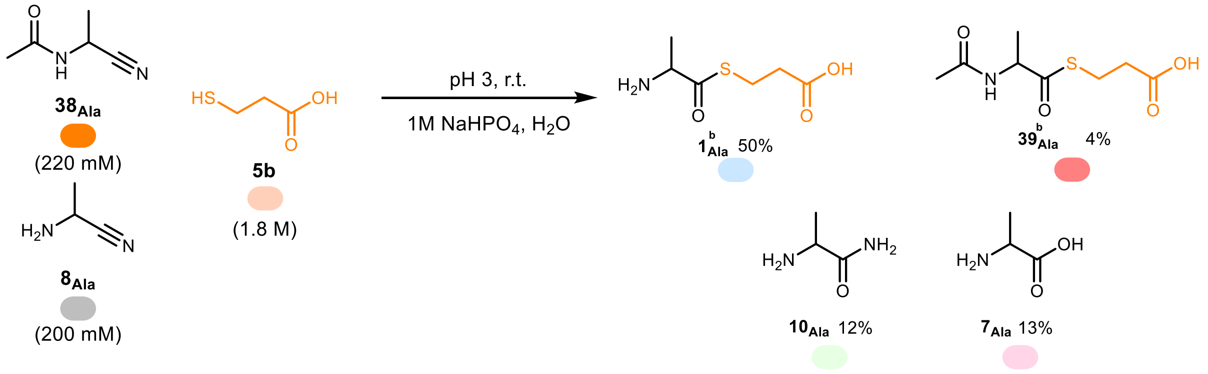

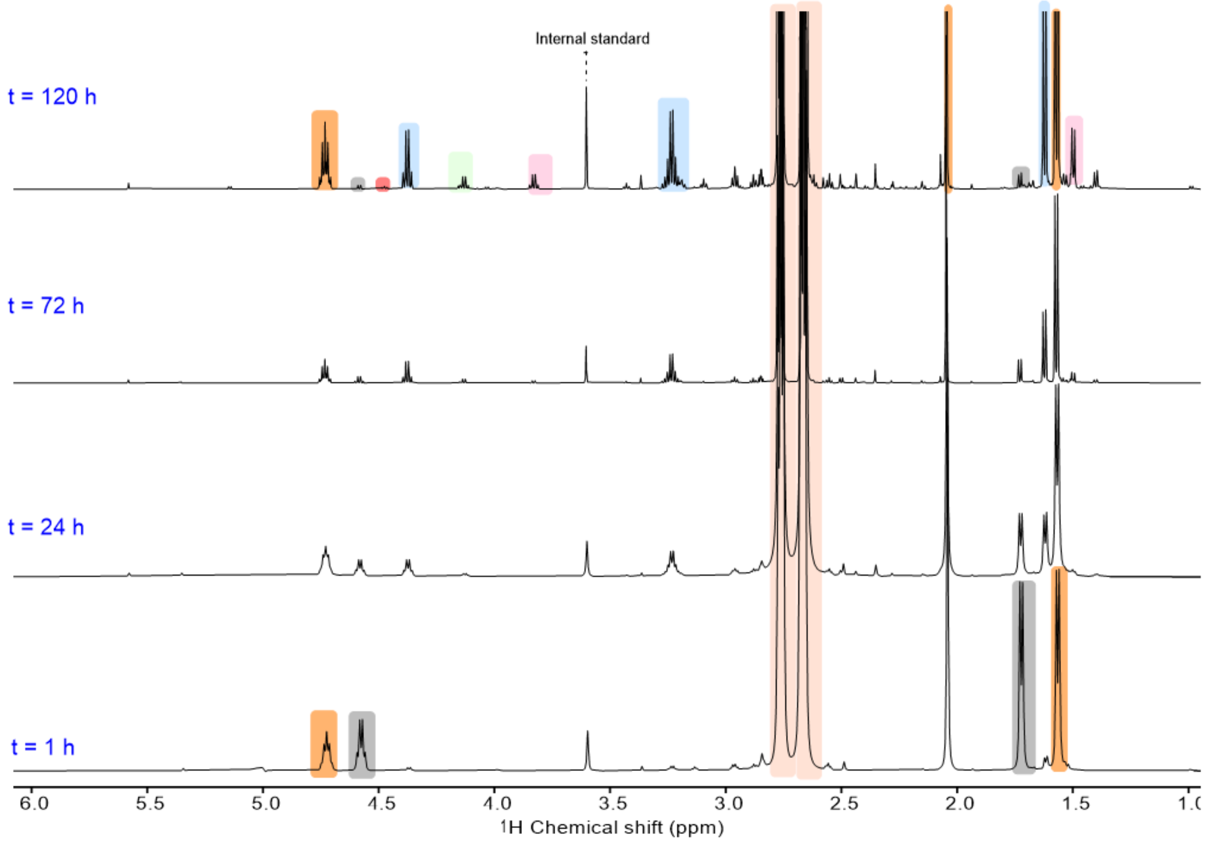

*Supplementary Figure 220. ^{1}H NMR (600 MHz, H_2O/D_2O 9:1, noesygppr1d, 1.0 – 6.0 ppm) spectra to show the competition reaction of alanine nitrile (**8**$_{Ala}$, 200 mM) and N-acetylated alanine nitrile (**38**$_{Ala}$, 220 mM) with 3-mercaptopropanoic acid (**5b**, 1.8 M) in 1 M NaH_2PO_4 buffer, with MSM (100 mM) as an internal standard at pH 3 and room temperature. Set up following General Procedure K, with **38**$_{Ala}$ added alongside **8**$_{Ala}$.*

^{1}H NMR (600 MHz, H_2O/D_2O 9:1) **1**$^{b}_{Ala}$ (partial assignment): δ_H 4.37 (1H, q, *J* = 7.2 Hz, Ala-α-C*H*(CH_3)), 3.34 – 3.21 (2H, m, COSC*H*$_2$), 1.62 (3H, d, *J* = 7.2 Hz, CH(C*H*$_3$)).

39$^{b}_{Ala}$ (partial assignment): δ_H 4.47 (1H, td, *J* = 7.13, 2.3 Hz, Ala-α-C*H*(CH3)).

10$_{Ala}$: δ_H 4.13 (1H, q, *J* = 7.1 Hz, Ala-α*H*-$CONH_2$).

7$_{Ala}$: δ_H 3.83 (1H, q, *J* = 7.2 Hz, Ala-α*H*-COOH).

Competition reaction of alanine nitrile ***8*** $_{\mathbf{Ala}}$ *and lactonitrile* ***36*** *with 3-mercaptopropanoic acid* ***5b***

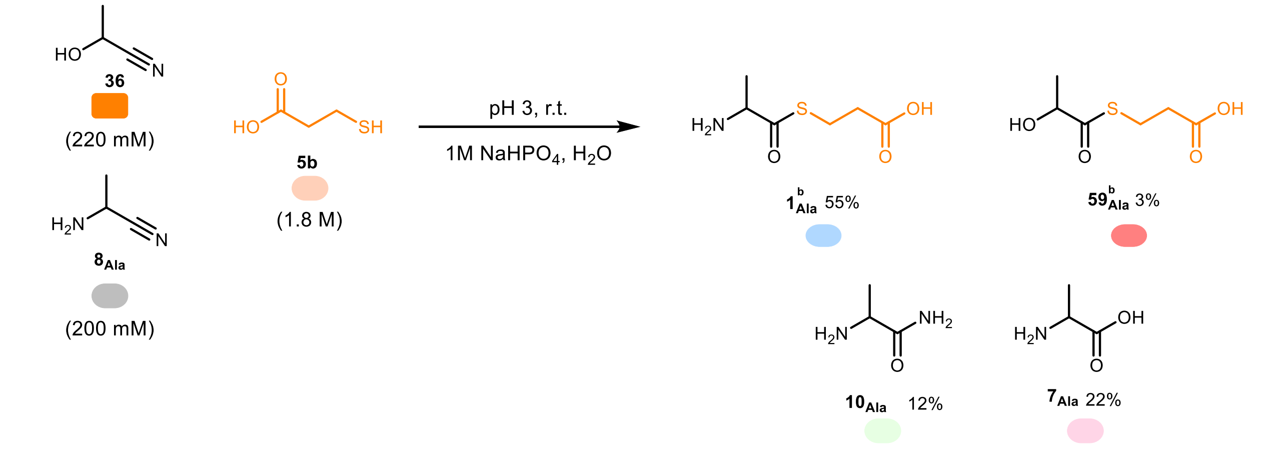

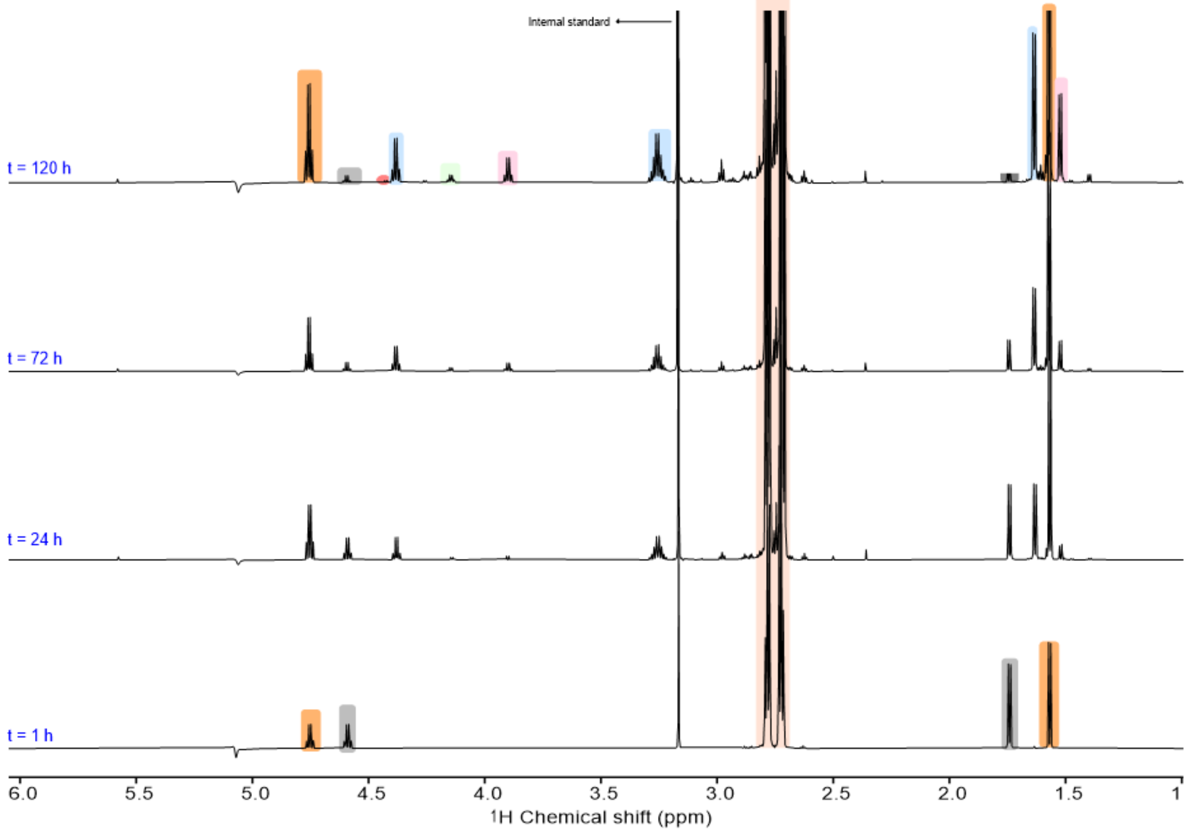

Supplementary Figure 221. ^{1}*H NMR (600 MHz,* H_2O/D_2O *9:1, noesygppr1d, 1.0 – 6.0 ppm) spectra to show the reaction of alanine nitrile (****8*** $_{\mathbf{Ala}}$*, 200 mM) and lactonitrile (****36****, 220 mM) with 3-mercaptopropanoic acid (****5b****, 1.8 M) in 1 M* NaH_2PO_4 *buffer, with MSM (100 mM) as an internal standard at pH 3 and room temperature. Set up following General Procedure K with* ***36*** *added alongside* ***8*** $_{\mathbf{Ala}}$*.*

^{1}H NMR (600 MHz, H_2O/D_2O 9:1) (partial assignment) $\mathbf{1^b_{Ala}}$: δ_H 4.38 (1H, q, J = 7.2 Hz, Ala-α-C*H*(CH_3)), 3.34 – 3.21 (m, 2H, COSC*H*$_2$), 1.64 (d, J = 7.2 Hz, CH(C*H*$_3$));

(partial assignment) $\mathbf{59^b_{Ala}}$: δ_H 4.43 (1H, q, J = 6.9 Hz, Ala-α-C*H*(CH_3)), 3.31 (2H, t, J = 6.9 Hz, COSC*H*$_2$);

$\mathbf{10_{Ala}}$: δ_H 4.15 (1H, q, J = 7.1 Hz, Ala-α*H*-$CONH_2$);

$\mathbf{7_{Ala}}$: δ_H 3.90 (1H, q, J = 7.2 Hz, Ala-α*H*-COOH).

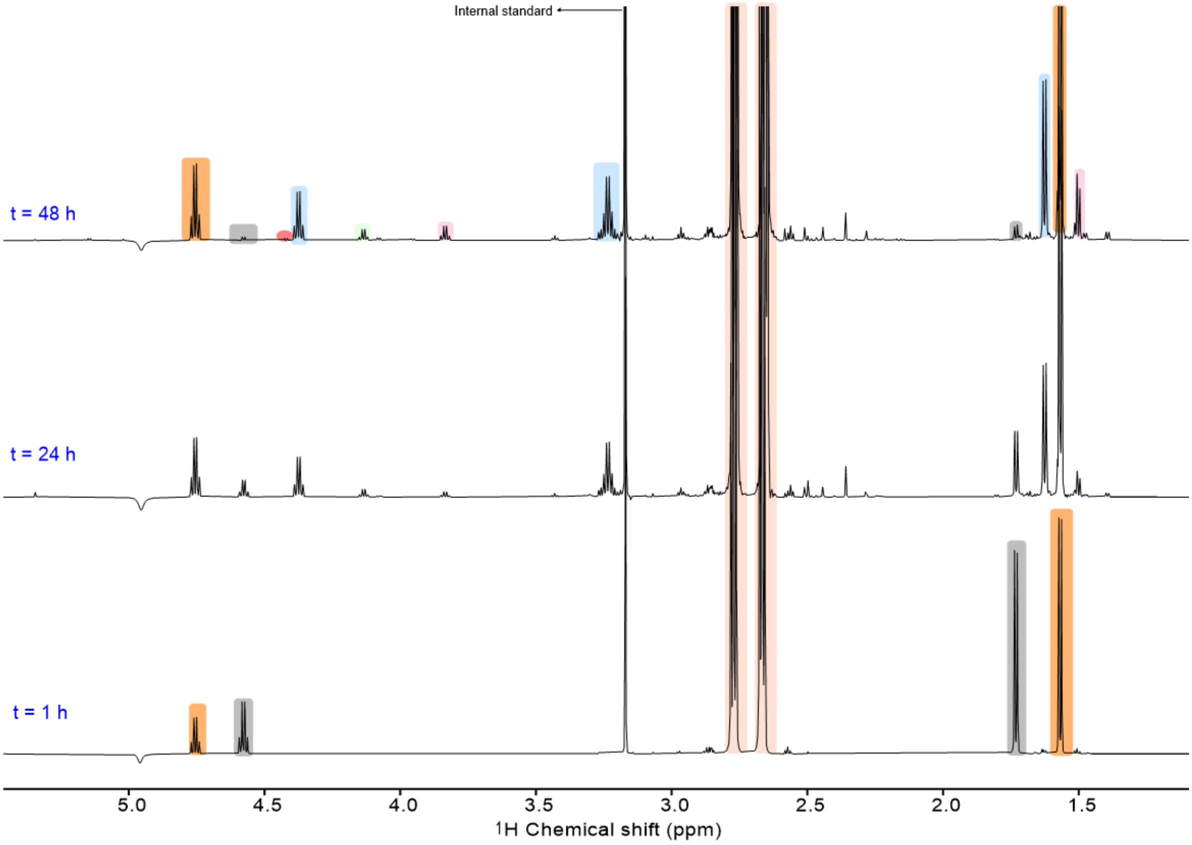

*Supplementary Figure 222. ¹H NMR (600 MHz, H_2O/D_2O 9:1, noesygppr1d, 1.5–5.0 ppm) spectra to show the reaction of alanine nitrile (**8_{Ala}**, 200 mM) and lactonitrile (**36**, 220 mM) with 3-mercaptopropanoic acid (**5b**, 1.8 M) in 1 M NaH_2PO_4 buffer, with MSM (100 mM) as an internal standard at pH 4 and room temperature. Set up following General Procedure K with 3**6** added alongside **8_{Ala}**.*

*Competition reaction of α-alanine nitrile **8_{Ala}** and β-alanine nitrile **37** with 3-mercaptopropanoic acid **5b***

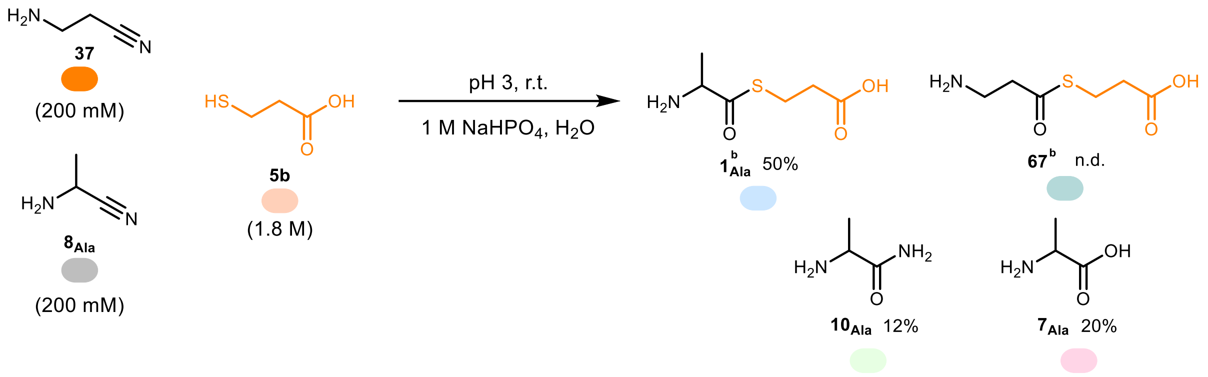

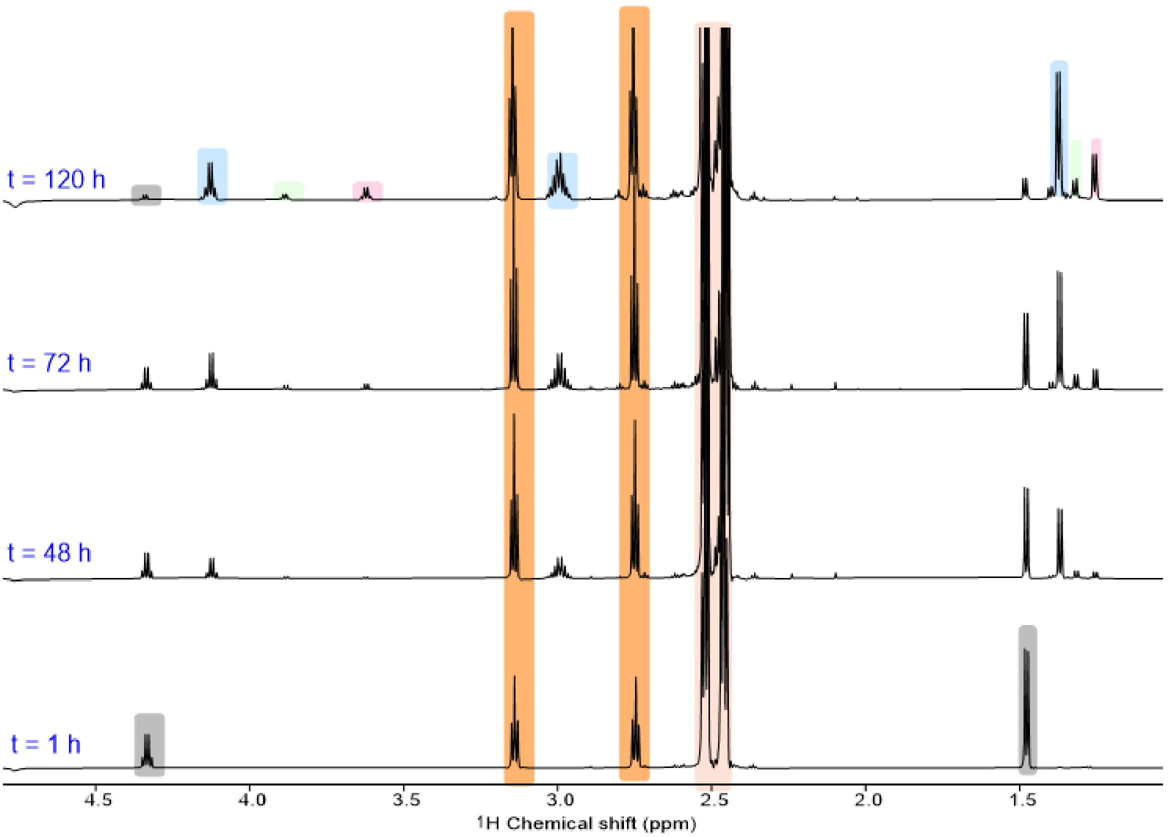

*Supplementary Figure 223. ^{1}H NMR (600 MHz, H_2O/D_2O 9:1, noesygppr1d, 1.5 – 4.5 ppm) spectra to show the competition reaction of α-alanine nitrile (**8_{Ala}**, 200 mM) and β-alanine nitrile (**37**, 200 mM) with 3-mercaptopropanoic acid (**5b**, 1.8 M) in 1 M $NaHPO_4$ buffer, at pH 3 and room temperature. Set up following General Procedure K with **37** added alongside **8_{Ala}**.*

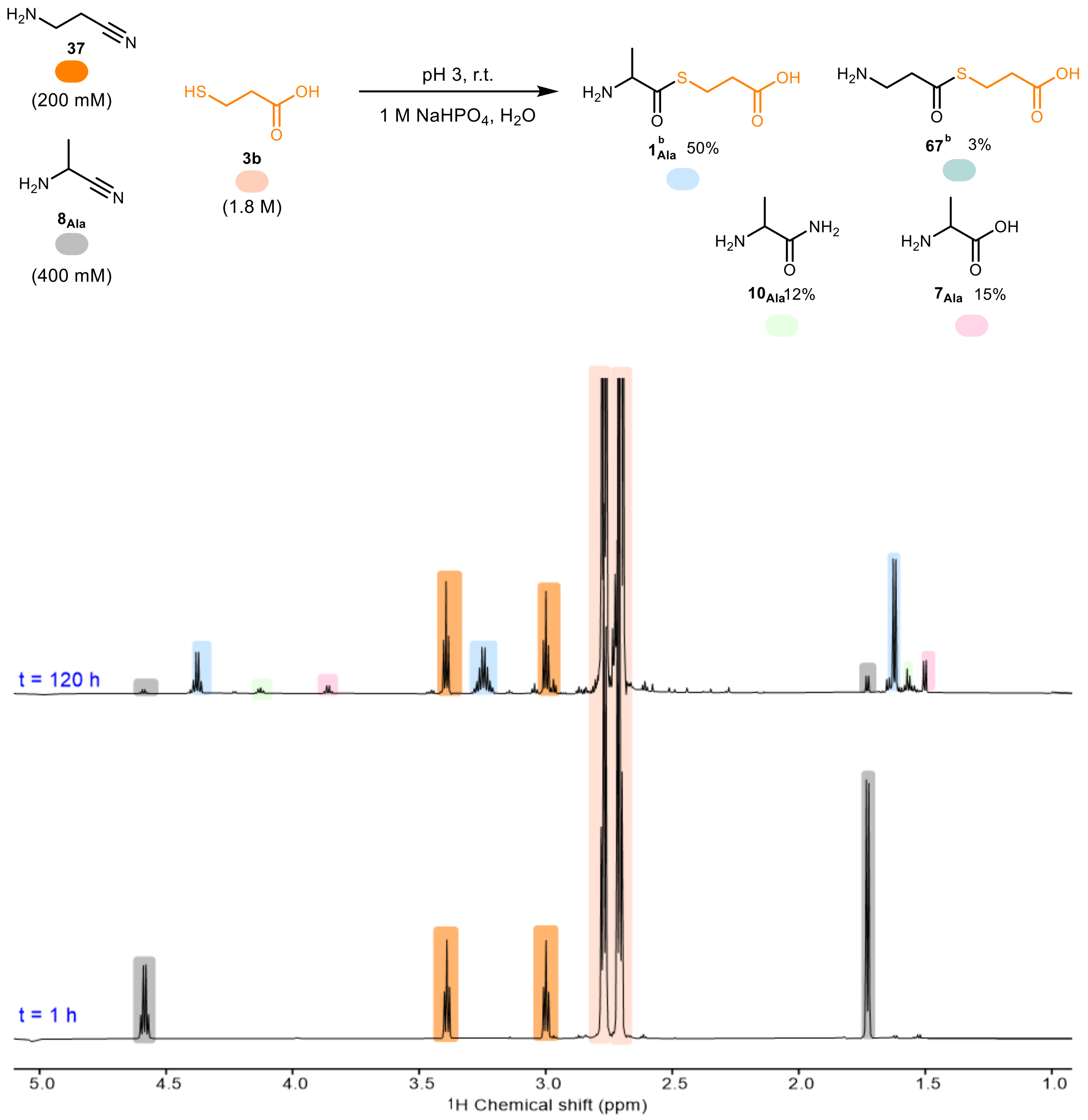

Supplementary Figure 224. [1]*H NMR (600 MHz, H_2O/D_2O 9:1, noesygppr1d, 1.0 – 5.0 ppm) spectrum to show the reaction of alanine nitrile (**8_{Ala}**, 400 mM) and β-alanine nitrile (**37**, 200 mM) with 3-mercaptopropanoic acid (**5b**, 1.8 M) in 1 M $NaHPO_4$ buffer, at pH 3 and room temperature. Set up following General Procedure K with **37** added alongside **8_{Ala}**.*

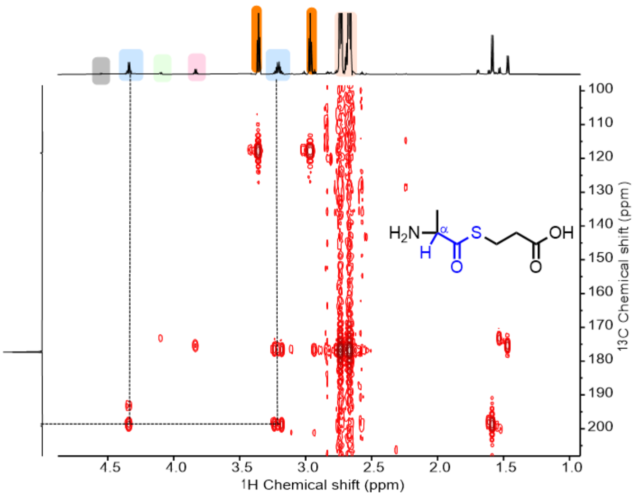

Supplementary Figure 225. *^{1}H–^{13}C HMBC ([1]H: 600 MHz [1.0 – 5.0 ppm], [13]C: 176 MHz [100 – 200 ppm], H_2O/D_2O 9:1) spectrum showing the diagnostic [2]JCH coupling of both Alanyl-aH and -SCH_2 in* $\mathbf{1^{b}_{Ala}}$ *at 4.32 and 3.20 ppm with one resonance at 198.9 ppm, which is characteristic of thioester bond formation.*

Reaction of L-alanine nitrile $\mathbf{8}_{Ala}$ *with 3-mercaptopropanoic acid* **5b** *at pD 4*

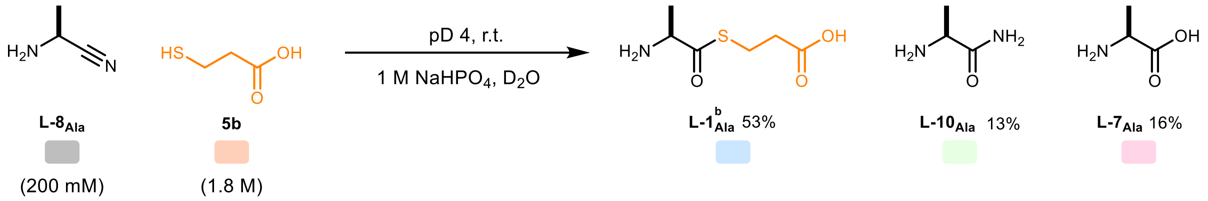

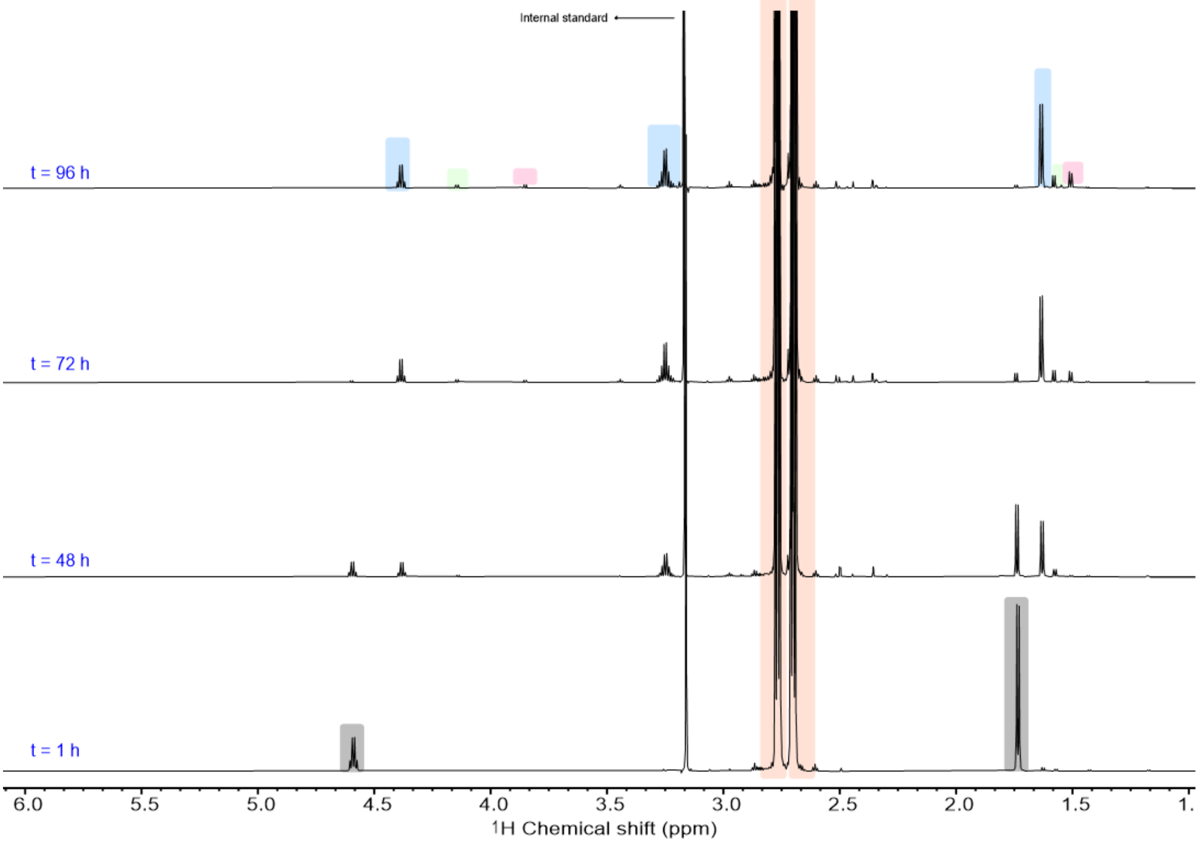

Supplementary Figure 226. *^{1}H NMR (600 MHz, D_2O, noesygppr1d, 1.0–6.0 ppm) spectra to show the reaction of L-alanine nitrile (L-$\mathbf{8}_{Ala}$, 200 mM) with 3-mercaptopropanoic acid (**5b**, 1.8 M) in 1 M NaH_2PO_4 buffer, with MSM (100 mM) as an internal standard at pD 4 and room temperature. Set up following General Procedure K.*

Upon incubation of L-alanine nitrile (L-$\mathbf{2}_{Ala}$, 200 mM) with 3-mercaptopropanoic acid (**3b**, 1.8 M) in 1 M NaD_2PO_4 (deuterium oxide) buffer at pD 4, no deuteration of L-$\mathbf{8}_{Ala}$, L-$\mathbf{1}^{b}_{Ala}$, L-$\mathbf{7}_{Ala}$ or L-$\mathbf{10}_{Ala}$ was observed after 96 hours (Supplementary Figure 226). To further check the enantiomeric purity of alanine thioester $\mathbf{1}^{b}_{Ala}$, the reaction mixture (0.5 mL) was diluted with water (H_2O) to 5 mL total volume and then extracted with ethyl acetate (2 × 5 mL). The resultant aqueous solution was then lyophilised. The white lyophilisate obtained contained L-$\mathbf{1}^{b}_{Ala}$, L-$\mathbf{10}_{Ala}$ and L-$\mathbf{7}_{Ala}$. The lyophilisate was dissolved in water (1 mL). An aliquot (0.1 mL) of the resultant solution was then added to a solution of:

(A) L-cysteine (L-$\mathbf{7}_{Cys}$, 30 mM), 4-mercaptophenylacetic acid (60 mM), TCEP (30 mM) and NaH_2PO_4 (200 mM) dissolved in degassed H_2O/D_2O (98:2, 1 mL); or

(B) DL-cysteine (DL-$\mathbf{7}_{Cys}$, 30 mM), 4-mercaptophenylacetic acid (60 mM), TCEP (30 mM) and NaH_2PO_4 (200 mM) dissolved in degassed H_2O/D_2O (98:2, 1 mL).

Each solution (A & B) was adjusted to pH 7.0 with NaOH/HCl and monitored by NMR spectroscopy. L-Alanine nitrile $\mathbf{8}_{\mathbf{Ala}}$ chirality was observed to be retained (>85:15, L:D) after conversion to thioester L-$\mathbf{1}^{\mathbf{b}}_{\mathbf{Ala}}$ and then to diastereomeric alanyl-cysteine ($\mathbf{13}_{\mathbf{AlaCys}}$) upon reaction with L-cysteine (L-$\mathbf{7}_{\mathbf{Cys}}$). We have not attempted to further optimise the retention of stereochemistry.

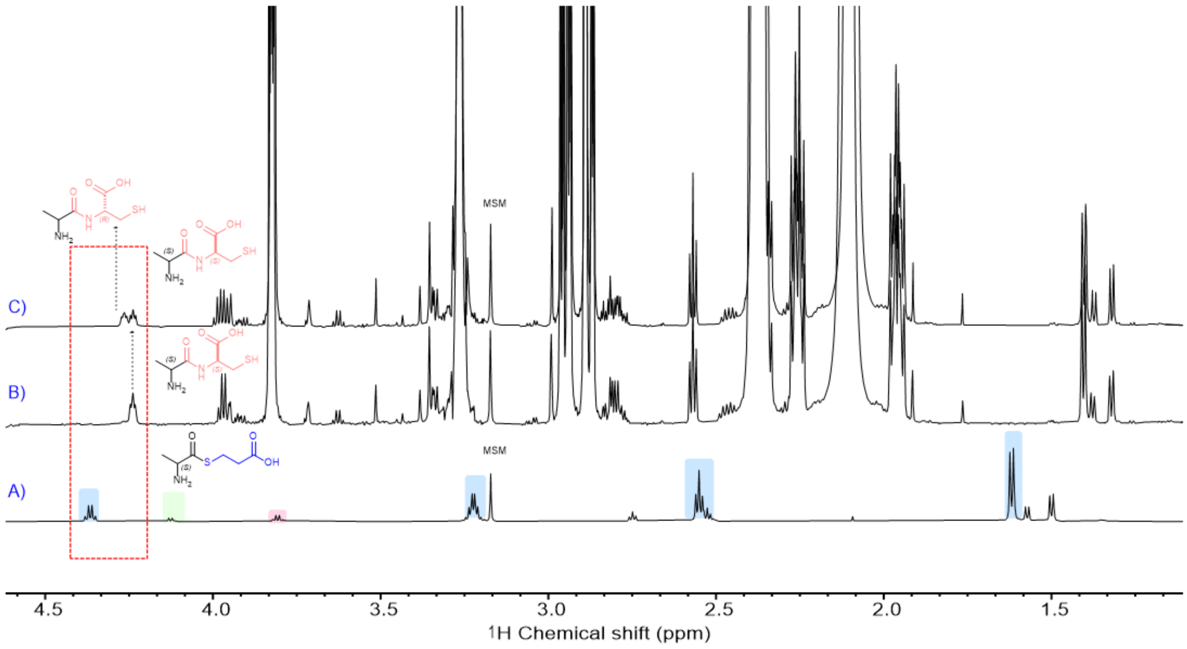

*Supplementary Figure 227. ^{1}H NMR (700 MHz, H_2O/D_2O 9:1, noesygppr1d, 1.0–4.5 ppm) spectra to show: A) L-$\mathbf{1}^{b}_{Ala}$, synthesised from the reaction of L-alanine nitrile (L-$\mathbf{2}_{Ala}$, 200 mM) with 3-mercaptopropanoic acid (**5b**, 1.8 M) in 1 M NaH_2PO_4 buffer at pD 4 following lyophilization; B) L-$\mathbf{1}^{b}_{Ala}$ (1 mM) (from spectrum A) reacted with L-cysteine (30 mM), 4-mercaptophenylacetic acid (60 mM), and TCEP (30 mM) with NaH_2PO_4 (200 mM); C) L-$\mathbf{1}^{b}_{Ala}$ (1 mM) (from spectrum A) reacted with DL-cysteine (30 mM), 4-mercaptophenylacetic acid (60 mM), and TCEP (30 mM) with NaH_2PO_4 (200 mM).*

The enantiomeric purity of L-alanine nitrile L-$\mathbf{8}_{\mathbf{Ala}}$ was quantified by derivatisation with (R)-(+)-α-methoxy-α-(trifluoromethyl)phenylacetyl chloride (Mosher's acid chloride) according to the following procedure. To a solution of L-alanine nitrile L-$\mathbf{8}_{\mathbf{Ala}}$ (0.10 mmol) or DL-alanine nitrile DL-$\mathbf{8}_{\mathbf{Ala}}$ (0.10 mmol) and triethylamine (24.5 mg, 0.24 mmol, 33.5 μL) in DMSO (1 mL) was added (R)-(+)-α-methoxy-α-(trifluoromethyl)phenylacetyl chloride (126 mg, 0.50 mmol, 93.6 μL). The solution was stirred at room temperature for 10 minutes, and ^{19}F NMR data were acquired. The enantiomeric ratio of L-$\mathbf{2}_{\mathbf{Ala}}$ was found to be >99:1.

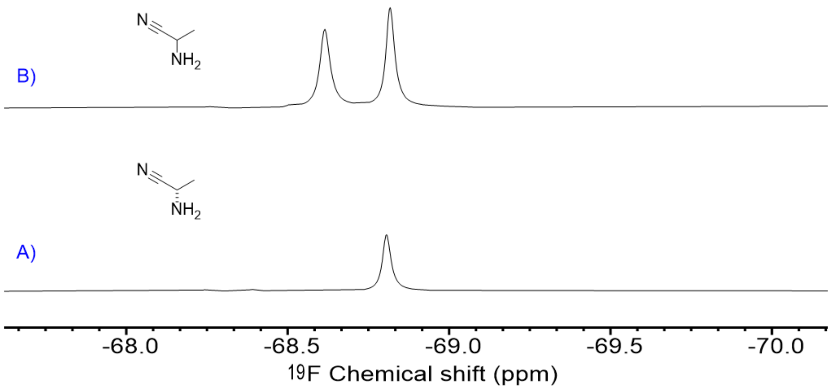

Supplementary Figure 228. *^{19}F (^{1}H-decoupled) NMR (376 MHz, H_2O/D_2O 9:1, noesygppr1d, -69.0 – -70.0 ppm) spectra to show the reaction of A) L-alanine nitrile (L-**8_{Ala}**, 100 mM) with (R)-(+)-a-methoxy-a-(trifluoromethyl)phenylacetyl chloride) in DMSO at room temperature; B) DL-alanine nitrile (DL-**8_{Ala}**, 100 mM) with (R)-(+)-a-methoxy-a-(trifluoromethyl)phenylacetyl chloride) in DMSO at room temperature.*

Formation of peptidyl RNA **40** from aminoacyl RNA **17**

*General Procedure L – Formation of peptidyl RNA **40** by the reaction of α-aminoacyl RNA **17** with α-amidothioacid **11***

α-Aminoacyl thiol (**1^{e}_{Aaa}**, 100 μmol) was dissolved in degassed H_2O/D_2O (98:2, 0.4 mL) with or without MES buffer (1 M), and the solution was carefully adjusted to pH 6 or 6.5 using 1−4 M NaOH or HCl as required. Nucleoside (10 μmol) was then added, the solution was re-adjusted to the specified pH value, and the final volume of the solution was adjusted to 0.5 mL with degassed H_2O/D_2O (98:2). The resulting solution was incubated at room temperature, and NMR spectra were periodically acquired. The structure of α-aminoacyl RNA (**17_{Aaa})** was confirmed by NMR analysis. After 18 or 24 hours, $K_3Fe(CN)_6$ (3 equiv. relative to **11**), and MES buffer (1 M, 500 μmol, pH 6.5) were added (if MES was not already present), followed by addition of α-amidothioacid (**11**, 150 μmol). After 10 min, the reaction mixture was centrifuge and supernatant solution was incubated at room temperature, and NMR spectra were acquired. The structure of peptidyl-RNA (**40**) was confirmed by ^1H NMR analysis and high-resolution mass spectrometry. The conversion (or yield) of each reaction was quantified by the consumption of the α-aminoacyl RNA **17**.

*General Procedure M – Formation of peptidyl RNA **40** by the reaction of α-aminoacyl RNA **17** with α-amidothioacid **11***

Cyclic arginine **33** (40 μmol) and MES buffer (500 mM) were dissolved in degassed D_2O (0.4 mL) and the solution was carefully adjusted to pD 6 or 6.5 using 1−4 M NaOH or HCl as required. The specified nucleoside (10 μmol) was then added, the solution was adjusted to the specified value, and the final volume of the solution was adjusted to 0.5 mL with degassed D_2O. The resulting solution was incubated at room temperature, and NMR spectra were periodically acquired. The structure of α-aminoacyl RNA (**17**) was confirmed by NMR analysis. After 2 or 3 hours, $K_3Fe(CN)_6$ (3 equiv. relative to **11**) was added, followed by α-amidothioacid (**11**, 50 μmol). Upon addition of **11**, the solution decreased to pD 5.0−4.5. After 10 min, the reaction mixture was centrifuge and NMR spectra of the supernatant were acquired. The structure of peptidyl-RNA (**40**) was confirmed by NMR analysis and high-resolution mass spectrometry.

Peptidyl RNA $\mathbf{40^n}_{Aaa}$ amidosynthesis

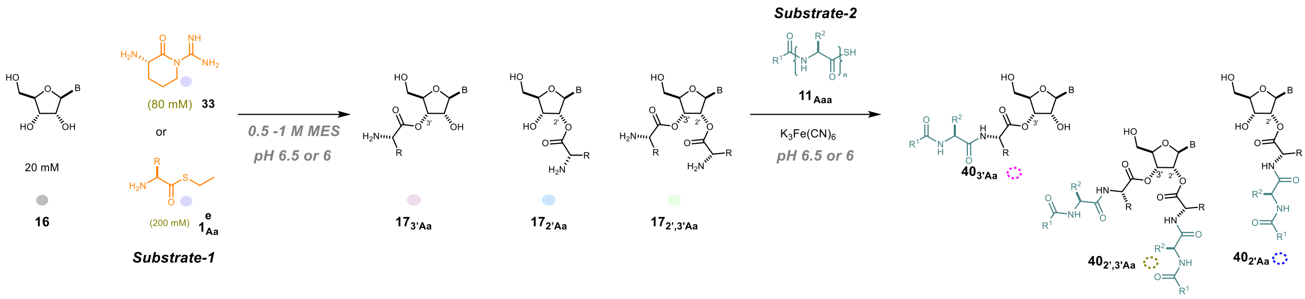

Entry	B	Substrate-1 L-1^{e}_{Ala} or 33	Substrate-2 Ac-AA-SH (mM)	pH/pD	MES present in first step	Reaction time (h) before oxidant	amount of oxidant added $K_3Fe(CN)_6$ (mM)	Peptide-RNA 40_{Aaa} (%)
**1**	**A**	Gly	Gly (300)	6.0	No	24	900	>99
**2**	**A**	Ala	Gly (300)	6.5	Yes	24	900	90
**3**	**A**	Leu	Gly (350)	6.5	Yes	24	1050	95
**4**	**A**	Lys	Gly (300)	6.0	No	24	900	93†
**5**	**A**	Glu	Gly (300)	6.0	No	24	900	92
**6**	**A**	Ser	Gly (300)	6.0	No	24	900	91
**7**	**A**	Gly	Val (300)#	6.0	Yes	18	900	89
**8**	**A**	**33**	Gly (105)	6.0/6.5	Yes	3/2*	315	93/98*
**9**	**A**	**33**	GlyGly (110)	6.0	Yes	3	330	92
**10**	**A**	**33**	GlyGlyGly (200)	6.0/6.5	Yes	3/2*	600	92/96*
**11**	**A**	**33**	AlaAla (150)	6.0	Yes	3	450	89
**12**	**A**	**33**	AlaPro (200)	6.0	Yes	3	600	82
**13**	**A**	**33**	MetGly (120)	6.0	Yes	3	360	77
**14**	**A**	**33**	Val (300)#	6.0	Yes	3	900	88
**15**	**A**	**33**	Phe (300)#	6.0	Yes	3	900	85
**16**	**A**	**33**	Met (300)#	6.0	Yes	3	900	87
**17**	**G$**	**33**	Gly (150)	6.0	Yes	3	450	92
**18**	**C**	**33**	Gly (200)	6.0/6.5	Yes	3/2*	600	96/97*
**19**	**U**	**33**	Gly (200)	6.0/6.5	Yes	3/2*	600	97/96*
**20**	**31**	**33**	Gly (150)	6.5	Yes	1	450	92
**21**	**31**	**33**	GlyGly (110)	6.5	Yes	3	330	93
**22**	**31**	**33**	GlyGlyGly (100)	6.5	Yes	3	300	95

*Supplementary Table 47: Total yields for peptidyl-RNA, $\mathbf{40_{Aaa}}$ were observed upon the reaction of **Substrate 1**, α-aminoacyl thiol $\mathbf{1^{e}_{Aaa}}$ or **33**, following General Procedure L or M respectively, with nucleoside (**16**) in MES buffer at pD 6 or 6.5 room temperature. After specified time reaction mixture was further treated with an oxidant [$K_3Fe(CN)_6$] followed by addition of **Substrate 2** (α-amidothioacid, Ac-AA-SH) until complete consumption of $\mathbf{17_{Aaa}}$ wasn't observed, unless and otherwise stated.*

See Supplementary figures 255–261 for further details for Entries 1–7 which were set up following General Procedure L.

†: Mixture of α-bisamidoacylated RNA and α,ε-bisamidoacylated RNA products, denoted as $\mathbf{40^{A}_{\alpha-LysGlyAc}}$ and $\mathbf{67^{A}_{\alpha,\varepsilon-Lys(GlyAc)2}}$ with a ratio of (ε-NH2/ε-NHGlyAc = 7:1). For more details, see supplementary figure 260–261 and supplementary table 49.

*# = To the reaction mixture was added $K_3Fe(CN)_6$ (300 mM), and then **11** (20 mM) was added in fifteen portions and the reaction mixture was sonicated and vortexed for 30 seconds after each addition, and then NMR spectra were acquired, and the solution was readjusted to pH 6 with 1.5 M MES buffer after five, ten and fifteen additions of **11**. See Supplementary figure 238–240, 260.*

*Entry 8–22 were set up following General Procedure M: Cyclic arginine (**33**, 80 mM) was incubated with nucleoside (**16** or **31**, 20 mM) in MES buffer (500 mM, pD 6.0). After specified time the reaction mixture was further treated with $K_3Fe(CN)_6$ (300 mM), followed by addition to the a-amidothioacid **11** (100 mM) until near complete consumption of $\mathbf{17_{Aaa}}$ or $\mathbf{41_{Aaa}}$ was observed. See Supplementary figure 229–240 for further details.*

** = The reaction at pD 6.5 for more details see Supplementary figure 241–248.*

$ = Initial nucleoside 2 mM.

High-resolution mass spectrometry data for peptidyl-RNA ***40*** $^{n}_{\mathbf{Aaa}}$

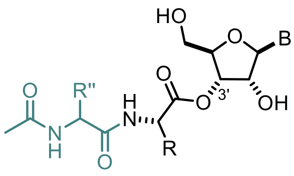

40 $_{3'\mathbf{Aaa}}$

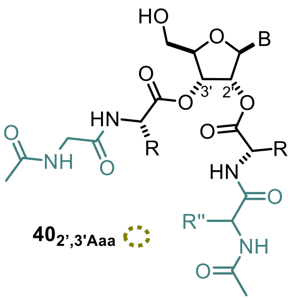

40 $_{2',3'\mathbf{Aaa}}$

Entry	Ac-Aaa-SH	Degree of aminoacylation*	*B*	HRMS-ESI for Aminoacylation RNAs 40^{n}_{Aaa}		
				**Product Formula**	**Theoretical mass**	**Observed mass**
1	Ac-GlyArg-SH	Mono	A	$C_{20}H_{31}N_{10}O_{7}$ [M+H]$^{+}$	523.2372	523.2386
2	Ac-GlyArg-SH	Bis	A	$C_{30}H_{48}N_{15}O_{10}$ [M+H]$^{+}$	777.3703	777.3624
3	Ac-GlyGlyArg-SH	Mono	A	$C_{22}H_{34}N_{11}O_{8}$ [M+H]$^{+}$	580.2586	580.2574
4	Ac-GlyGlyArg-SH	Bis	A	$C_{34}H_{54}N_{17}O_{12}$ [M-H]$^{-}$	892.4132	892.4121
5	Ac-GlyGlyGlyArg-SH	Mono	A	$C_{24}H_{37}N_{12}O_{9}$ [M+H]$^{+}$	637.2801	637.2798
6	Ac-GlyGlyGlyArg-SH	Bis	A	$C_{38}H_{60}N_{19}O_{14}$ [M+H]$^{+}$	1006.4562	1006.4559
7	Ac-AlaAlaArg-SH	Mono	A	$C_{24}H_{38}N_{11}O_{8}$ [M+H]$^{+}$	608.2899	608.2884
8	Ac-AlaAlaArg-SH	Bis	A	$C_{38}H_{62}N_{17}O_{12}$ [M+H]$^{+}$	948.4758	948.4750
9	Ac-MetGlyArg	Mono	A	$C_{25}H_{40}N_{11}O_{8}S$ [M+H]$^{+}$	654.2776	654.2761
10	Ac-MetGlyArg	Bis	A	$C_{40}H_{66}N_{17}O_{12}S_{2}$[M+H]$^{+}$	1040.4513	1040.4510
11	Ac-GlyProArg	Mono	A	$C_{26}H_{40}N_{11}O_{8}$ [M+H]$^{+}$	630.3456	630.3566
12	Ac-ValGly-SH	Mono	A	$C_{19}H_{27}N_{7}O_{7}$ [M+H]$^{+}$	466.2026	466.2039
13	Ac-ValGly-SH	Bis	A	$C_{28}H_{41}N_{9}O_{10}$ [M+H]$^{+}$	664.3049	664.3036
14	Ac-PheArg-SH	Mono	A	$C_{27}H_{37}N_{10}O_{7}$ [M+H]$^{+}$	613.2841	613.2828
15	Ac-PheArg-SH	Bis	A	$C_{44}H_{61}N_{15}O_{10}$ [M+H]$^{+}$	959.4715	959.4721
16	Ac-ValArg-SH	Mono	A	$C_{23}H_{37}N_{10}O_{7}$ [M+H]$^{+}$	565.2814	565.2834
17	Ac-ValArg-SH	Bis	A	$C_{36}H_{60}N_{15}O_{10}$ [M+H]$^{+}$	862.4642	862.4615
18	Ac-GlyArg-SH	Mono	G	$C_{20}H_{31}N_{10}O_{8}$ [M+H]$^{+}$	539.2321	539.2313
19	Ac-GlyArg-SH	Bis	G	$C_{30}H_{48}N_{15}O_{11}$ [M+H]$^{+}$	794.3652	794.3635
20	Ac-GlyArg-SH	Mono	C	$C_{19}H_{31}N_{8}O_{8}$ [M+H]$^{+}$	499.2259	499.2251
21	Ac-GlyArg-SH	Bis	C	$C_{29}H_{48}N_{13}O_{11}$ [M+H]$^{+}$	754.3591	754.3568
22	Ac-GlyArg-SH	Mono	U	$C_{19}H_{30}N_{7}O_{9}$ [M+H]$^{+}$	500.2099	500.2095
23	Ac-GlyArg-SH	Bis	U	$C_{29}H_{47}N_{12}O_{12}$ [M+H]$^{+}$	755.3431	755.3430
24	Ac-GlyArg-SH	Mono	cAMP	$C_{20}H_{30}N_{10}O_{9}$ P [M+H]$^{+}$	585.1929	585.1929
25	Ac-GlyGlyGly-SH	Mono	cAMP	$C_{24}H_{36}N_{12}O_{11}P$ [M+H]$^{+}$	699.2359	699.2342
26	Ac-GlyLeu-SH	Mono	A	$C_{20}H_{30}N_{7}O_{7}$ [M+H]$^{+}$	480.2187	480.2188
27	Ac-GlyLeu-SH	Bis	A	$C_{30}H_{46}N_{9}O_{10}$ [M+H]$^{+}$	692.3330	692.3337
28	Ac-GlyGly-SH	Mono	A	$C_{16}H_{21}N_{7}O_{7}$ [M+H]$^{+}$	424.1575	424.1571
29	Ac-GlyGly-SH	Bis	A	$C_{22}H_{29}N_{9}O_{10}$ [M+H]$^{+}$	580.2100	580.2110
30	Ac-GlySer-SH	Mono	A	$C_{17}H_{23}N_{7}O_{8}$ [M+H]$^{+}$	454.1680	454.1670
31	Ac-GlyGlu-SH	Mono	A	$C_{19}H_{26}N_{7}O_{9}$ [M+H]$^{+}$	496.1774	496.1786
32	Ac-GlyAla-SH	Mono	A	$C_{17}H_{24}N_{7}O_{7}$ [M+H]$^{+}$	438.1732	438.1731

Supplementary Table 48: HRMS data for peptidyl-RNA ***40*** $_{\mathbf{Aaa}}$ *products formed from α-aminoacyl RNA* ***17*** $^{A}_{\mathbf{Aaa}}$ *and Ac-Aaa-SH. * The aminoacylation can occur once to form monoaminoacylated products,* ***40*** $_{3'\mathbf{Aaa}}$ *(or* ***40*** $_{2'\mathbf{Aaa}}$ *or* ***40*** $_{5'\mathbf{Aaa}}$*), or twice to form bisaminoacylated products,* ***40*** $_{2'3'\mathbf{Aaa}}$ *(or* ***40*** $_{2'5'\mathbf{Aaa}}$ *or* ***40*** $_{3'5'\mathbf{Aaa}}$*).*

Synthesis of peptidyl RNA $\mathbf{40^{A}_{ArgGlyAc}}$ *from aminoacyl-RNA* $\mathbf{17^{A}_{Arg}}$ *with α-amidothioacid* $\mathbf{11_{Gly}}$

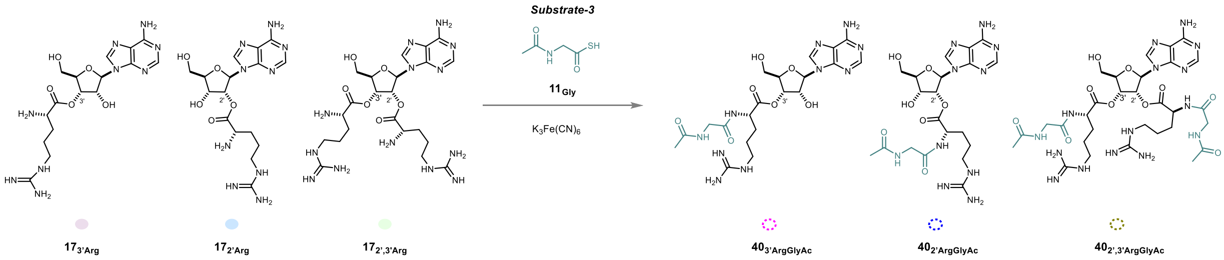

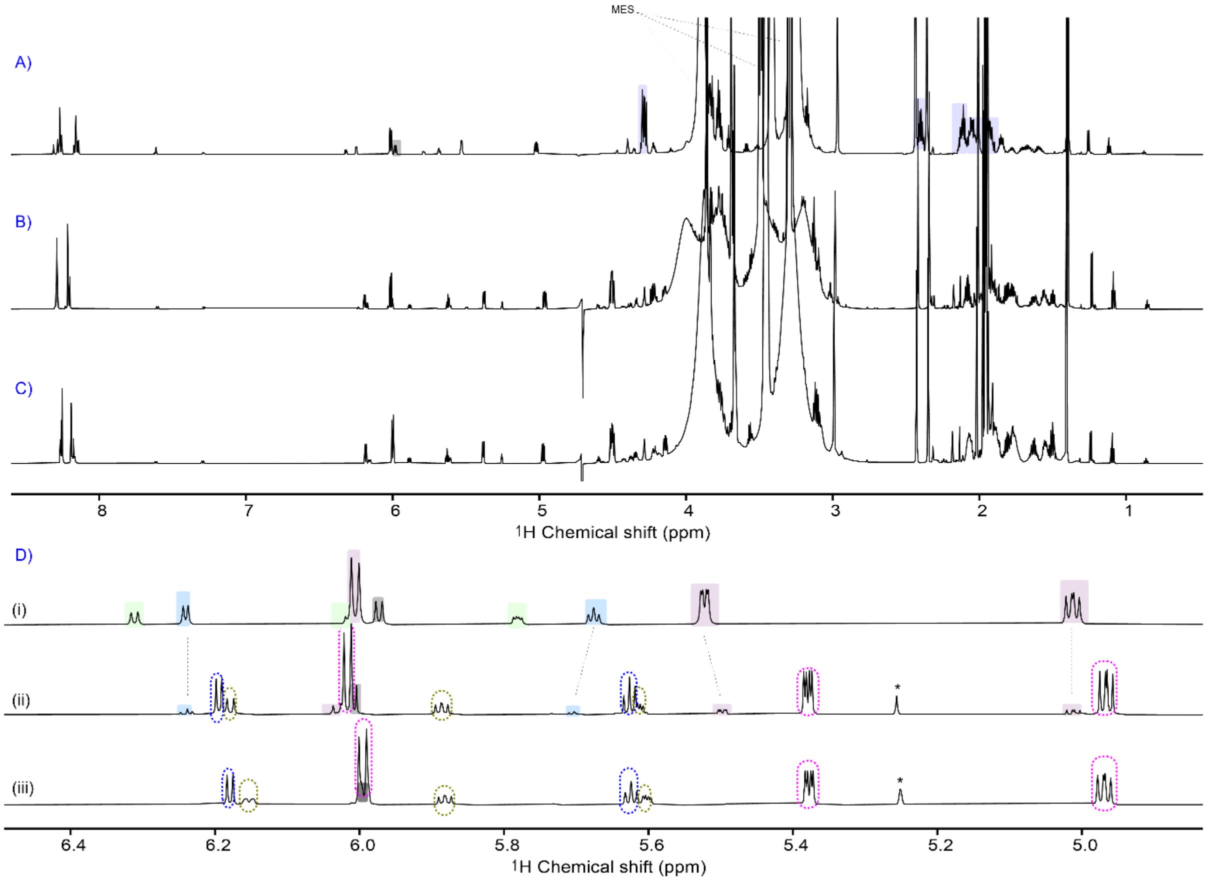

Supplementary Figure 229: ^{1}*H NMR (700 MHz,* D_2O*, noesygppr1d, 0.5 – 8.5 ppm) spectra showing the synthesis of peptidyl-RNA* $\mathbf{40_{ArgGlyAc}}$ *via aminoacyl-RNA* $\mathbf{17^{A}_{Arg}}$*, formed in situ from cyclic arginine (**33**, 80 mM) and adenosine (**16A**, 20 mM) in MES buffer (500 mM, pD 6.0). Set up following General Procedure M: A) Spectrum acquired after 3 hours. B) The reaction mixture from spectrum A treated with* $K_3[Fe(CN)_6]$ *(300 mM), followed by the addition of Ac-Gly-SH (*$\mathbf{11_{Gly}}$*, 100 mM) at pD 6.0. C) The reaction mixture from spectrum B was further treated with* $K_3[Fe(CN)_6]$ *(15 mM), followed by the addition of Ac-Gly-SH (*$\mathbf{11_{Gly}}$*, 5 mM) at pD 6.0, until the complete consumption of* $\mathbf{17_{Aaa}}$*. D) Zoom-in of spectra A → (i), B → (ii), and C → (iii) between 4.8–6.5 ppm, with starting material and product signals highlighted. * = The singlet resonance observed at 5.3 ppm corresponds to a byproduct derived from Ac-Gly-SH and is not associated with the nucleoside. In the NMR spectrum, the starting material is highlighted as (■) = **16A**; (■) = **33**.*

^{1}H NMR (700 MHz, D_2O) $\mathbf{40^{16A}_{3'ArgGlyAc}}$ (partial assignment): δ_H 5.99 (1H, d, J = 7.3 Hz, (C1')-H), 5.38 (1H, dd, J = 5.4, 2.3 Hz, (C3')-H), 4.97 (1H, dd, J = 7.3, 5.4 Hz, (C2')-H);

^{1}H NMR (700 MHz, D_2O) $\mathbf{40^{16A}_{2'ArgGlyAc}}$ (partial assignment): δ_H 6.18 (1H, d, J = 5.6 Hz, (C1')-H), 5.62 (1H, t, J = 5.6 Hz, (C2')-H);

^{1}H NMR (700 MHz, D$_2$O) $\mathbf{40^{16A}_{2'3'ArgGlyAc}}$ (partial assignment): δ$_H$ 6.15 (1H, d, *J* = 6.9 Hz, (C1')-H), 5.88 (1H, dd, *J* = 6.9, 5.3 Hz, (C2')-H), 5.60 (1H, dd, *J* = 5.3, 2.5 Hz, (C3')-H).

Synthesis of peptidyl RNA ***40^G$_{ArgGlyAc}$*** *from aminoacyl-RNA* ***17^G$_{Arg}$*** *with α-amidothioacid* ***11$_{Gly}$***

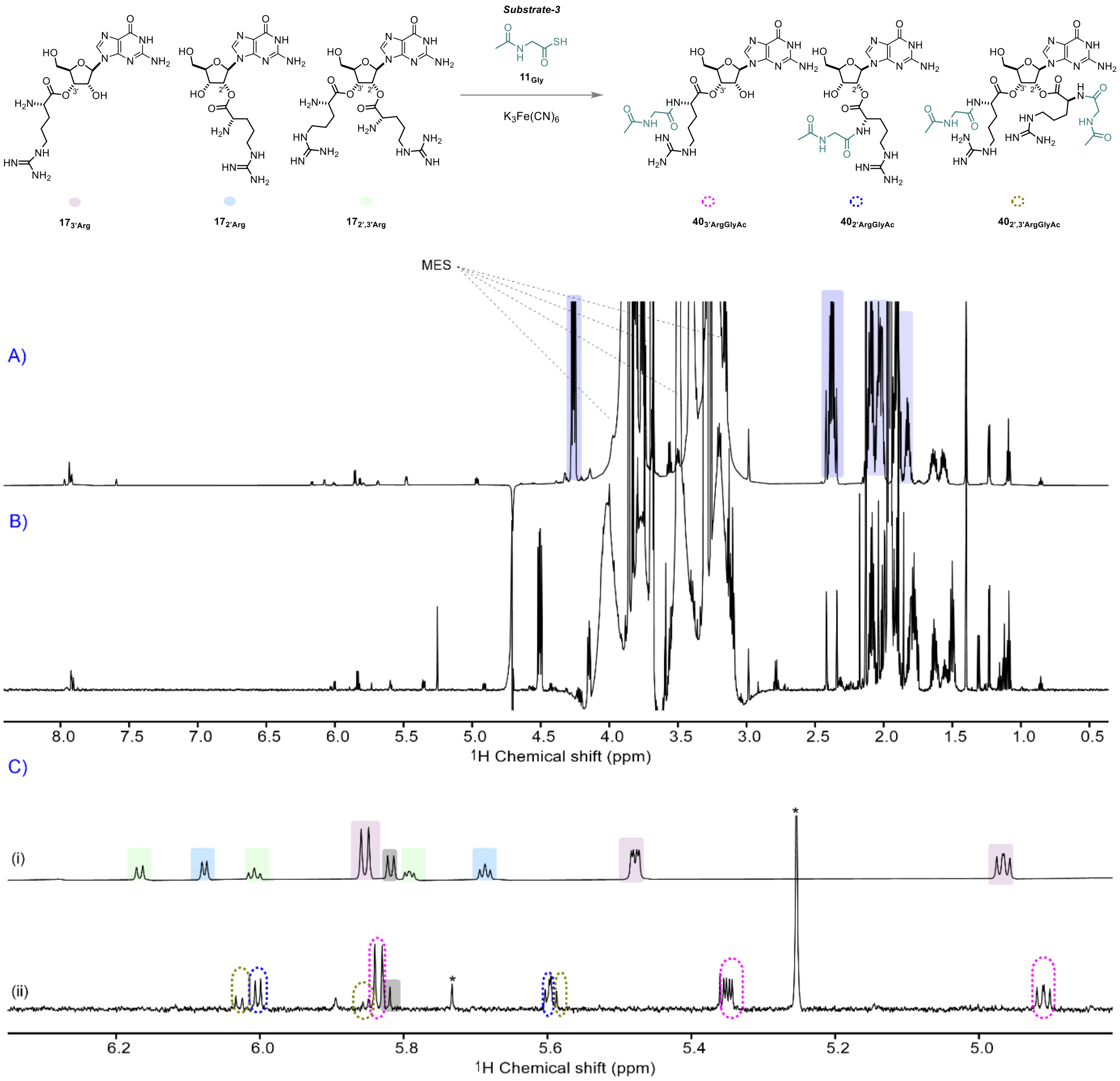

Supplementary Figure 230: ^{1}H NMR (700 MHz, D$_2$O, noesygppr1d, 0.5 – 8.0 ppm) spectra showing synthesis of peptidyl-RNA ***40$_{ArgGlyAc}$*** *via aminoacyl-RNA* **$17^G_{Arg'}$**, *formed in situ from cyclic arginine (**33**, 80 mM) and guanosine (**16G**, 2 mM) in MES buffer (500 mM, pD 6.0). Set up following General Procedure M: A) Spectrum acquired after 4 hours. B) The reaction mixture from spectrum A treated with K$_3$[Fe(CN)$_6$] (450 mM), followed by the addition of Ac-Gly-SH (**11$_{Gly}$**, 150 mM) at pD 6.0. C) Zoom-in of spectra A ⟶ (i) and B ⟶ (ii) between 4.9–6.3 ppm, with starting material and product signals highlighted. * = The singlet resonances observed at 5.25 and 5.75 ppm correspond to byproducts derived from Ac-Gly-SH and are not associated with the nucleoside. In the NMR spectrum, the starting material is highlighted as (■) = **16G**; (■) = **33**.*

^{1}H NMR (700 MHz, D$_2$O) $\mathbf{40^{16G}_{3'ArgGlyAc}}$ (partial assignment): δ$_H$ 5.83 (1H, d, *J* = 7.2 Hz, (C1')-H), 5.35(1H, dd, *J* = 5.6, 2.7 Hz, (C3')-H), 4.91 (1H, apparent m, (C2')-H);

^{1}H NMR (700 MHz, D_2O) $\mathbf{40^{16G}_{2'ArgGlyAc}}$ (partial assignment): δ_H 6.00 (1H, d, J = 5.2 Hz, (C1')-H), 5.60 (1H, overlapped, (C2')-H);

^{1}H NMR (700 MHz, D_2O) $\mathbf{40^{16G}_{2'3'ArgGlyAc}}$ (partial assignment): δ_H 6.03 (1H, d, J = 6.3 Hz, (C1')-H), 5.60 – 5.58 (1H, overlapped, (C3')-H).

Synthesis of peptidyl RNA $\mathbf{40^{U}_{ArgGlyAc}}$ *from aminoacyl-RNA* $\mathbf{17^{U}_{Arg}}$ *with α-amidothioacid* $\mathbf{11_{Gly}}$

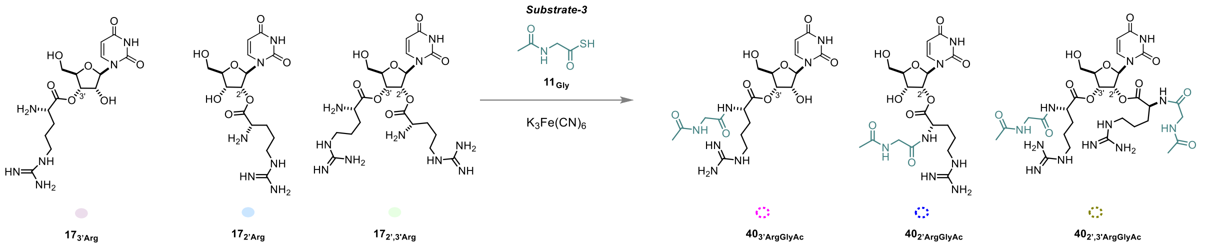

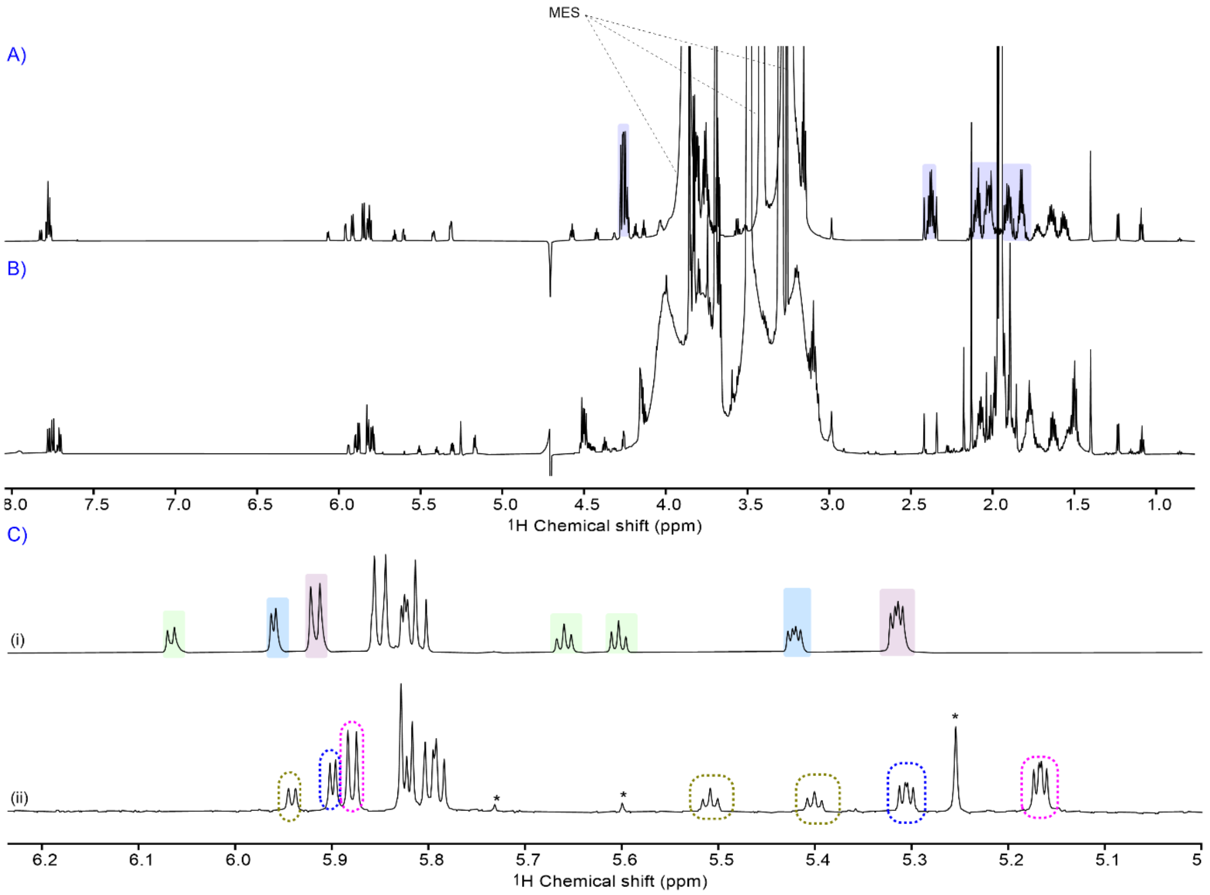

Supplementary Figure 231: ^{1}H NMR (700 MHz, D_2O, noesygppr1d, 0.5 – 8.0 ppm) spectra showing the synthesis of peptidyl-RNA $\mathbf{40_{ArgGlyAc}}$ *via aminoacyl-RNA* $\mathbf{17^{U}_{Arg'}}$ *formed in situ from cyclic arginine (**33**, 80 mM) and uridine (**16U**, 20 mM) in MES buffer (500 mM, pD 6.0). Set up following General Procedure M: A) Spectrum acquired after 4 hours. B) The reaction mixture from spectrum A treated with $K_3[Fe(CN)_6]$ (600 mM), followed by the addition of Ac-Gly-SH (* $\mathbf{11_{Gly}}$*, 200 mM) at pD 6.0. C) Zoom-in of spectra A → (i) and B → (ii) between 5.0–6.2 ppm, with starting material and product signals highlighted. * = The singlet resonances observed at 5.25, 5.59 and 5.73 ppm correspond to byproducts derived from Ac-Gly-SH and are not associated with the nucleoside. In the NMR spectrum, the starting material is highlighted as (■) = **16U**; (■) = **33**.*

^{1}H NMR (700 MHz, D_2O) $\mathbf{40^{16U}_{3'ArgGlyAc}}$ (partial assignment): δ_H 5.88 (1H, d, *J* = 6.2 Hz, (C1')-H), 5.17 (1H, dd, *J* = 5.6, 3.8 Hz, (C3')-H);

^{1}H NMR (700 MHz, D_2O) $\mathbf{40^{16U}_{2'ArgGlyAc}}$ (partial assignment): δ_H 5.90 (1H, d, *J* = 4.1 Hz, (C1')-H), 5.31 (1H, dd, *J* = 5.9, 4.1 Hz, (C2')-H);

^{1}H NMR (700 MHz, D_2O) $\mathbf{40^{16U}_{2'3'ArgGlyAc}}$ (partial assignment): δ_H 5.95 (1H, d, *J* = 5.2 Hz, (C1')-H), 5.51 (1H, t, *J* = 5.2 Hz, (C2')-H), 5.41 (1H, t, *J* = 5.2 Hz, (C3')-H).

Synthesis of peptidyl RNA $\mathbf{40^{C}_{ArgGlyAc}}$ *from aminoacyl-RNA* $\mathbf{17^{C}_{Arg}}$ *with α-amidothioacid* $\mathbf{11_{Gly}}$

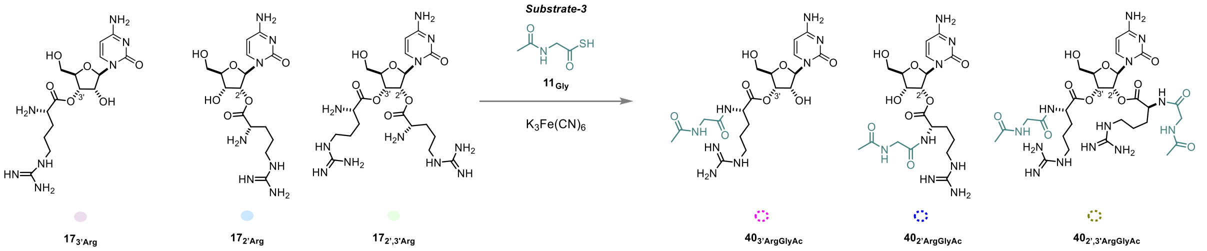

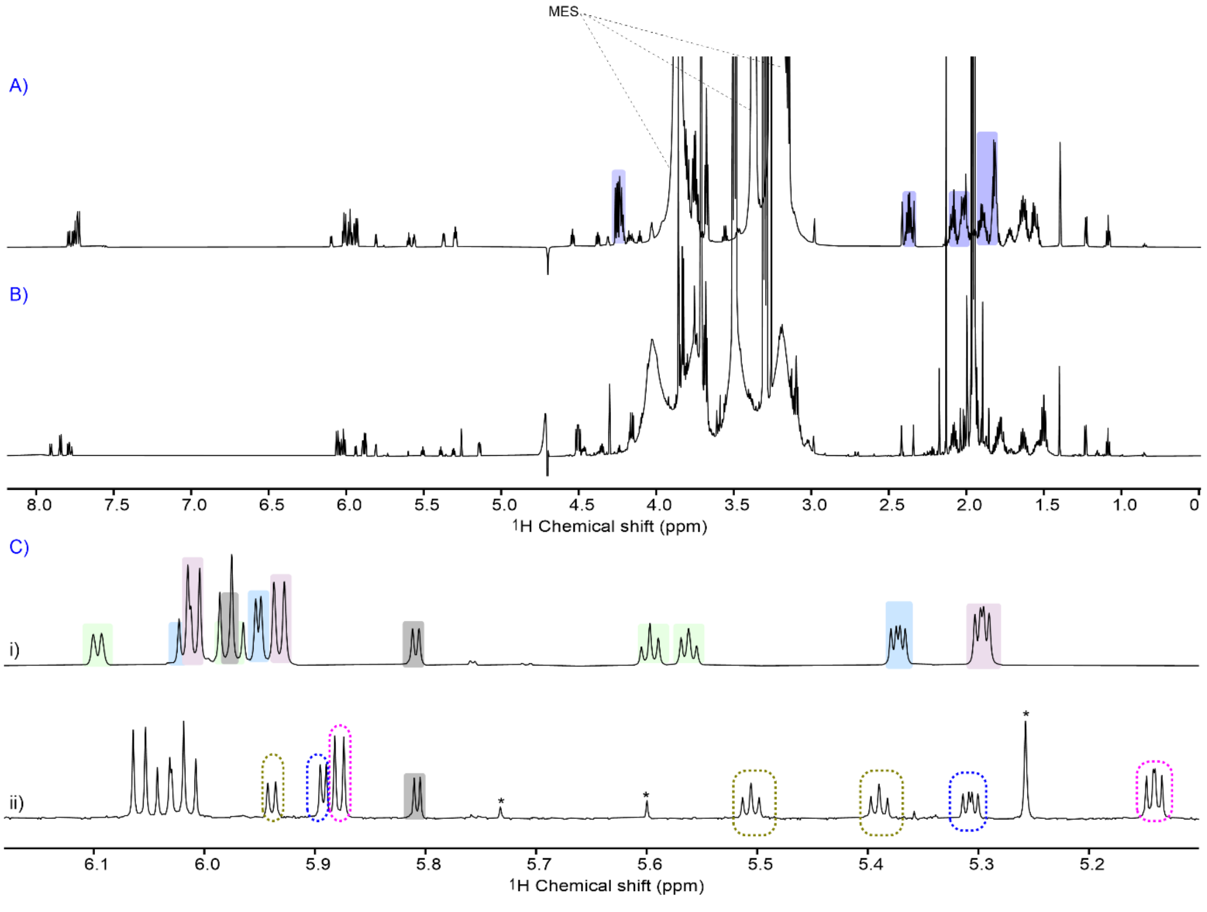

Supplementary Figure 232: ^{1}H NMR (700 MHz, D_2O, noesygppr1d, 0.5 – 8.0 ppm) spectra showing the synthesis of peptidyl-RNA $\mathbf{40_{ArgGlyAc}}$ *via aminoacyl-RNA* $\mathbf{17^{C}_{Arg}}$*, formed in situ from cyclic arginine (**33**, 80 mM) and cytidine (**16C**, 20 mM) in MES buffer (500 mM, pD 6.0), Set up*

*following General Procedure M: A) Spectrum acquired after 4 hours. B) The reaction mixture from spectrum A treated with $K_3[Fe(CN)_6]$ (600 mM), followed by the addition of Ac-Gly-SH (**11_{Gly}**, 200 mM) at pD 6.0. C) Zoom-in of spectra A → (i) and B → (ii) between 5.1–6.2 ppm, with starting material and product signals highlighted. * = The singlet resonances observed at 5.27, 5.59 and 5.73 ppm correspond to byproducts derived from Ac-Gly-SH and are not associated with the nucleoside. In the NMR spectrum, the starting material is highlighted as (■) = **16C**; (■) = **33**.*

^{1}H NMR (700 MHz, D_2O) $\mathbf{40^{16C}_{3'ArgGlyAc}}$ (partial assignment): δ_H 5.88 (1H, d, *J* = 7.3 Hz, (C1')-H), 5.14 (1H, dd, *J* = 5.6, 4.2 Hz, (C3')-H);

^{1}H NMR (700 MHz, D_2O) $\mathbf{40^{16C}_{2'ArgGlyAc}}$ (partial assignment): δ_H 5.89 (1H, d, *J* = 3.8 Hz, (C1')-H), 5.31 (1H, dd, *J* = 5.8, 3.8 Hz, (C2')-H);

^{1}H NMR (700 MHz, D_2O) $\mathbf{40^{16C}_{2'3'ArgGlyAc}}$ (partial assignment): δ_H 5.94 (1H, d, *J* = 5.2 Hz, (C1')-H), 5.51 (1H, t, *J* = 5.2 Hz, (C2')-H), 5.39 (1H, t, *J* = 5.2 Hz, (C3')-H).

Synthesis of peptidyl RNA $\mathbf{40^{A}_{ArgGlyGlyAc}}$ *from aminoacyl-RNA* $\mathbf{17^{A}_{Arg}}$ *with α-amidothioacid* $\mathbf{11_{GlyGly}}$

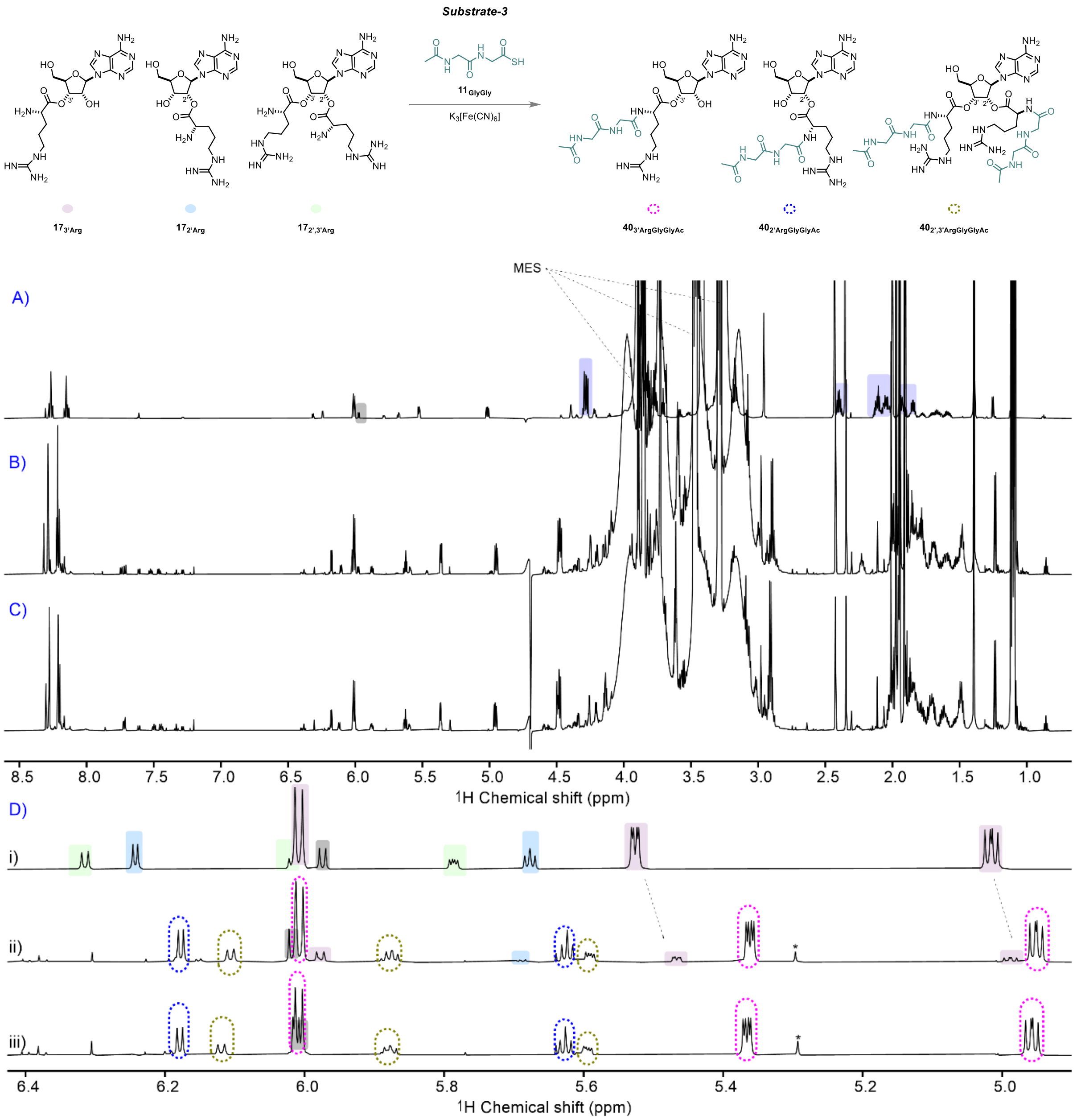

Supplementary Figure 233: *^{1}H NMR (700 MHz, D_2O, noesygppr1d, 0.5 – 8.5 ppm) spectra showing the synthesis of peptidyl-RNA* $\mathbf{40_{ArgGlygGlyAc}}$ *via aminoacyl-RNA* $\mathbf{17^{A}_{Arg}}$*, formed in situ from cyclic arginine (**33**, 80 mM) and adenosine (**16A**, 20 mM) in MES buffer (500 mM, pD 6.0), Set up following General Procedure M: A) Spectrum acquired after 3 hours. B) The reaction mixture from spectrum A treated with $K_3[Fe(CN)_6]$ (300 mM), followed by the addition of Ac-GlyGly-SH (*$\mathbf{11_{AcGlyGly}}$*, 100 mM) at pD 6.0. C) The reaction mixture from spectrum B further treated with $K_3[Fe(CN)_6]$ (30 mM), followed by the addition of Ac-GlyGly-SH (*$\mathbf{11_{GlyGly}}$*, 10 mM) at pD 6.0, until complete consumption of* $\mathbf{17_{Aaa}}$ *was observed. D) Zoom-in of spectra A → (i), B → (ii) and C → (iii) between 4.9–6.4 ppm, with starting material and product signals highlighted. * = The singlet resonance at 5.31 ppm is a byproduct derived from Ac-GlyGly-SH and are not related to the nucleoside. In the NMR spectrum, the starting material is highlighted as (▆) = **16A**; (▆) = **33**.*

^{1}H NMR (700 MHz, D_2O) $\mathbf{40^{16A}_{3'ArgGlyGlyAc}}$ (partial assignment): δ_H 6.01 (1H, d, *J* = 7.2 Hz, (C1')-H), 5.37 (1H, dd, *J* = 5.4, 2.4 Hz, (C3')-H), 4.96 (1H, *J* = 7.2, 5.4 Hz, (C2')-H);

^{1}H NMR (700 MHz, D$_2$O) $\mathbf{40^{16A}_{2'ArgGlyGlyAc}}$ (partial assignment): δ$_H$ 6.18 (1H, d, *J* = 5.4 Hz, (C1')-H), 5.63 (1H, t, *J* = 5.6, (C2')-H);

^{1}H NMR (700 MHz, D$_2$O) $\mathbf{40^{16A}_{2'3'ArgGlyGlyAc}}$ (partial assignment): δ$_H$ 6.13 (1H, d, *J* = 6.7 Hz, (C1')-H), 5.91 – 5.86 (apparent, m, (C2')-H), 5.60 (1H, dd, *J* = 5.4, 2.4 Hz, (C3')-H).

Synthesis of peptidyl RNA $\mathbf{40^{A}_{ArgGlyGlyGlyAc}}$ *from aminoacyl-RNA* $\mathbf{17^{A}_{Arg}}$ *with α-amidothioacid* $\mathbf{11_{GlyGlyGly}}$

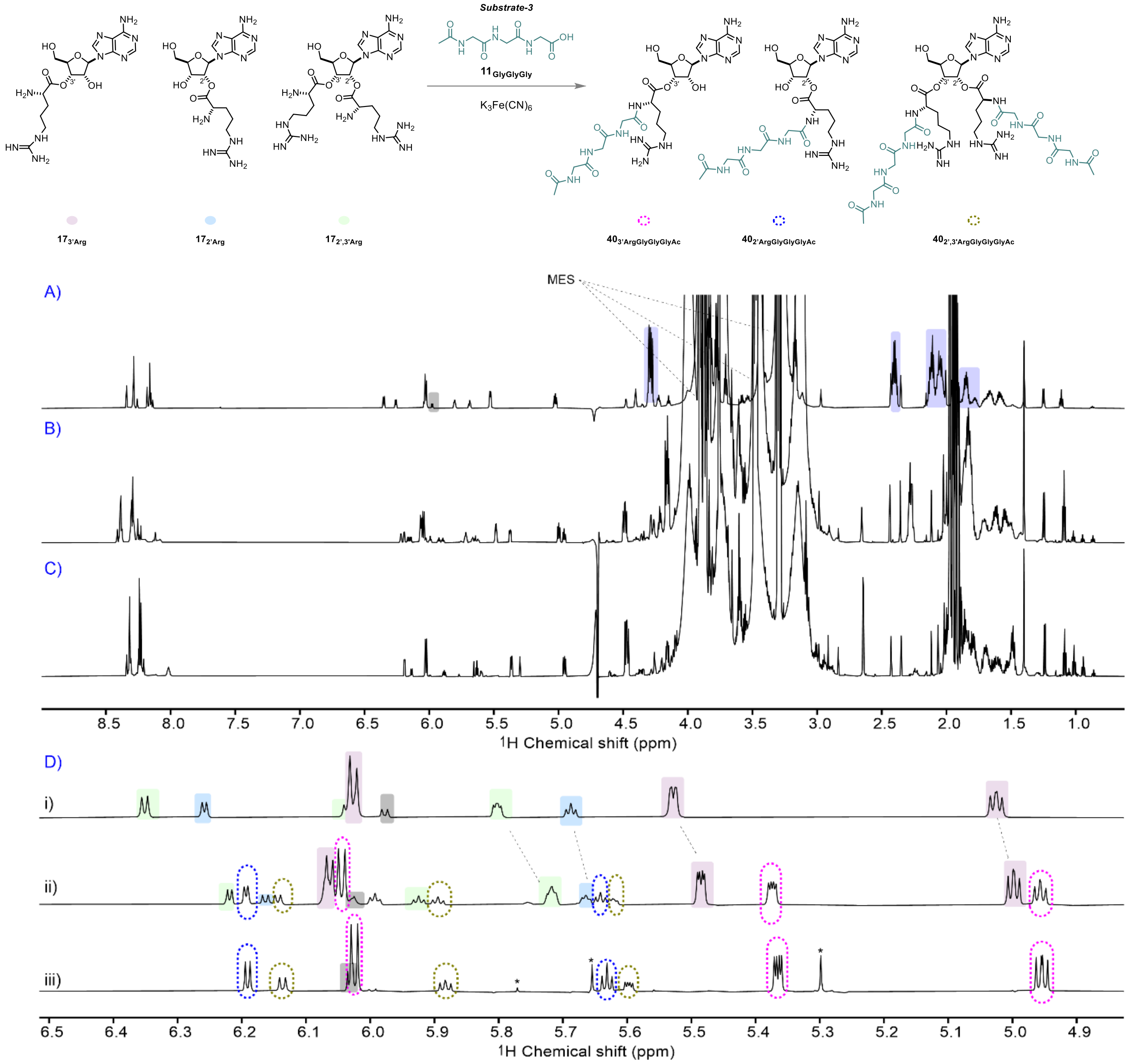

Supplementary Figure 234: ^{1}H NMR (700 MHz, D$_2$O, noesygppr1d, 0.5 – 9.0 ppm) spectra showing the synthesis of peptidyl-RNA $\mathbf{40_{ArgGlyGlyGlyAc}}$ *via aminoacyl-RNA* $\mathbf{17^{A}_{Arg}}$*, formed in situ from cyclic arginine cyclic arginine (**33**, 80 mM) and adenosine (**16A**, 20 mM) in MES buffer (500 mM, pD 6.0), Set up following General Procedure M: A) Spectrum acquired after 3 hours. B) The reaction mixture from spectrum A treated with K$_3$[Fe(CN)$_6$] (300 mM), followed by the addition of Ac-GlyGlyGly-SH (*$\mathbf{11_{GlyGlyGly}}$*, 100 mM) at pD 6.0. C) The reaction mixture from spectrum B further treated with K$_3$[Fe(CN)$_6$] (300mM), followed by the addition of Ac-GlyGlyGly-SH (*$\mathbf{11_{GlyGlyGly}}$*, 100 mM) at pD 6.0, until complete consumption of* $\mathbf{17_{Aaa}}$ *was observed. D) Zoom-in of spectra A ⟶ (i), B ⟶ (ii) and C ⟶ (iii) between 4.9–6.5 ppm, with starting material and product signals highlighted. * = The singlet resonances at 5.30, 5.65 and 5.73 ppm are byproducts derived from Ac-GlyGlyGly-SH and are not related to the nucleoside. In the NMR spectrum, the starting material is highlighted as (■) = **16A**; (■) = **33**.*

^{1}H NMR (700 MHz, D$_2$O) $\mathbf{40^{16A}_{3'ArgGlyGlyGlyAc}}$ (partial assignment): δ$_H$ 6.02 (1H, d, *J* = 7.0 Hz, (C1')-H), 5.36 (1H, dd, *J* = 5.4, 2.4 Hz, (C3')-H), 4.95 (1H, dd, *J* = 7.0, 5.4 Hz, (C2')-H);

^{1}H NMR (700 MHz, D$_2$O) $\mathbf{40^{16A}_{2'ArgGlyGlyGlyAc}}$ (partial assignment): δ$_H$ 6.19 (1H, d, *J* = 5.4 Hz, (C1')-H), 5.63 (1H, t, *J* = 5.6 Hz, (C2')-H);

^{1}H NMR (700 MHz, D$_2$O) $\mathbf{40^{16A}_{2'3'ArgGlyGlyGlyAc}}$ (partial assignment): δ$_H$ 6.14 (1H, d, *J* = 6.7 Hz, (C1')-H), 5.88 (1H, dd, *J* = 6.9, 5.3 Hz, (C2')-H), 5.60 (1H, dd, *J* = 5.3, 2.5 Hz, (C3')-H).

Synthesis of peptidyl RNA $\mathbf{40^{A}_{ArgAlaAlaAc}}$ *from aminoacyl-RNA* $\mathbf{17^{A}_{Arg}}$ *with α-amidothioacid* $\mathbf{11_{AlaAla}}$

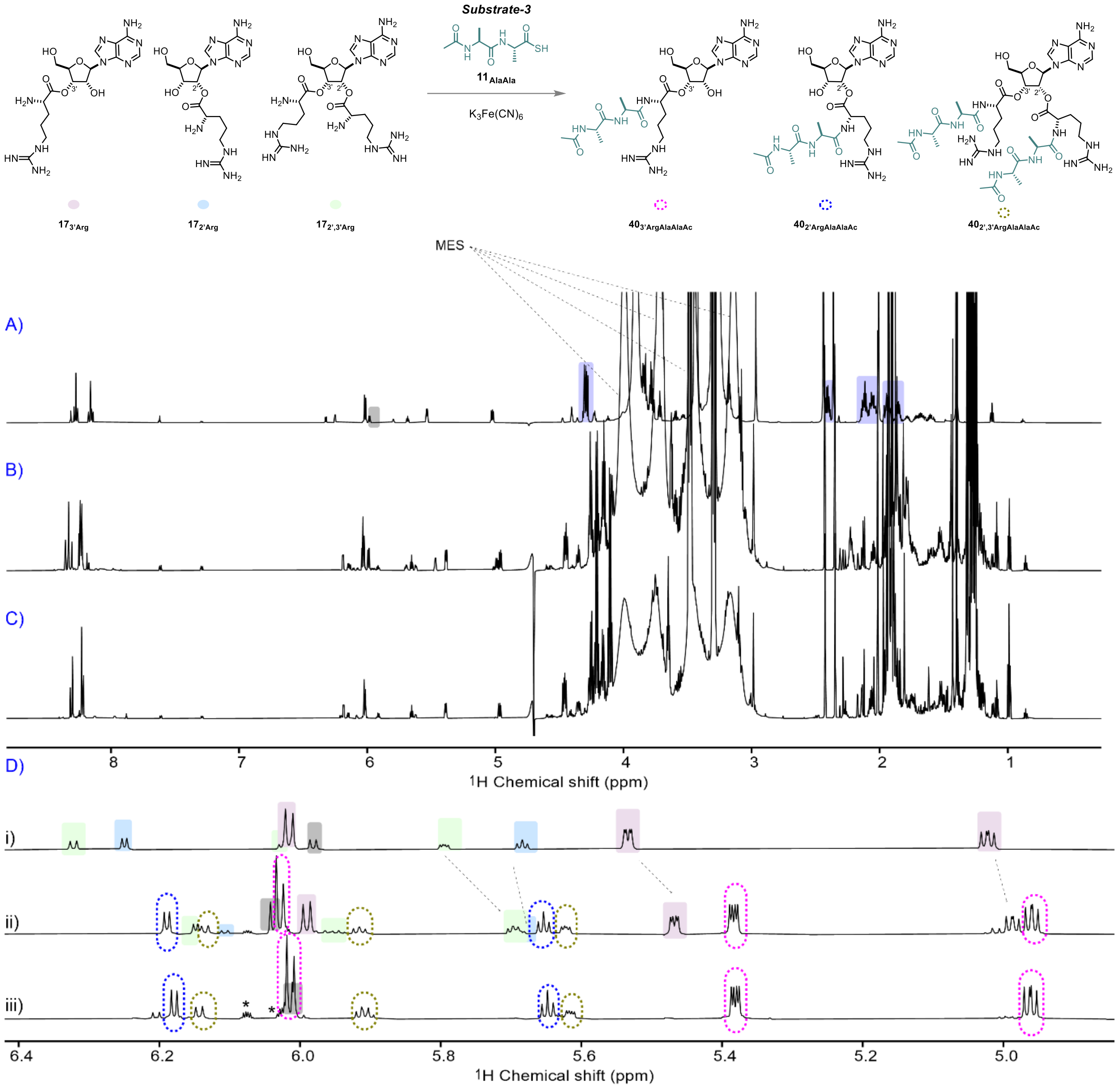

Supplementary Figure 235: ^{1}H NMR (700 MHz, D$_2$O, noesygppr1d, 0.5 – 9.0 ppm) spectra showing the synthesis of peptidyl-RNA $\mathbf{40_{ArgAlaAlaAc}}$ *via aminoacyl-RNA* $\mathbf{17^{A}_{Arg}}$*, formed in situ from cyclic arginine of cyclic arginine (**33**, 80 mM) and adenosine (**16A**, 20 mM) in MES buffer (500 mM, pD 6.0): A) Spectrum acquired after 3 hours. B) The reaction mixture from spectrum A treated with K$_3$[Fe(CN)$_6$] (300 mM), followed by the addition of Ac-AlaAla-SH (**11$_{AlaAla}$**, 100 mM) at pD 6.0. C) The reaction mixture from spectrum B further treated with K$_3$[Fe(CN)$_6$] (150mM), followed*

*by the addition of Ac-AlaAla-SH (**11_{AlaAla}**, 50 mM) at pD 6.0, until complete consumption of **17_{Aaa}** was observed. D) Zoom-in of spectra A → (i), B → (ii) and C → (iii) between 4.9–6.5 ppm, with starting material and product signals highlighted. * = The multiplet resonances at 6.04 and 6.09 ppm are from byproducts derived from Ac-AlaAla-SH and are not associated with the nucleoside. Set up following General Procedure M. In the NMR spectrum, the starting material is highlighted as (■) = **16A**; (■) = **33**.*

Note: 16A, **33** and Ac-AlaAla-SH (**11_{AlaAla}**) were enantiopure, and the formation of the corresponding peptidyl-RNA proceeded with stereoretention affording $\mathbf{40}^{\mathbf{16A}}_{\mathbf{3'ArgAlaAlaAc}}$, $\mathbf{40}^{\mathbf{16A}}_{\mathbf{2'ArgAlaAlaAc}}$ and $\mathbf{40}^{\mathbf{16A}}_{\mathbf{2'3'ArgAlaAlaAc}}$ each as one diastereomer within the limits of detection.

^{1}H NMR (700 MHz, D_2O) $\mathbf{40}^{\mathbf{16A}}_{\mathbf{3'ArgAlaAlaAc}}$ (partial assignment): δ_H 6.02 (1H, d, *J* = 7.3 Hz, (C1')-H), 5.39 (1H, dd, *J* = 5.4, 2.5 Hz, (C3')-H), 4.95 (1H, dd, *J* = 7.3, 5.4 Hz, (C2')-H);

^{1}H NMR (700 MHz, D_2O) $\mathbf{40}^{\mathbf{16A}}_{\mathbf{2'ArgAlaAlaAc}}$ (partial assignment): δ_H 6.18 (1H, d, *J* = 5.6 Hz, (C1')-H), 5.63 (1H, t, *J* = 5.6 Hz, (C2')-H);

^{1}H NMR (700 MHz, D_2O) $\mathbf{40}^{\mathbf{16A}}_{\mathbf{2'3'ArgAlaAlaAc}}$ (partial assignment): δ_H 6.15 (1H, d, *J* = 6.7 Hz, (C1')-H), 5.94 – 5.89 (1H, apt., m, (C2')-H), 5.60 (1H, dd, *J* = 5.3, 2.5 Hz, (C3')-H).

Synthesis of peptidyl RNA $\mathbf{40^{A}}_{\mathbf{ArgProAlaAc}}$ *from aminoacyl-RNA* $\mathbf{17^{A}}_{\mathbf{Arg}}$ *with α-amidothioacid* $\mathbf{11}_{\mathbf{AlaPro}}$

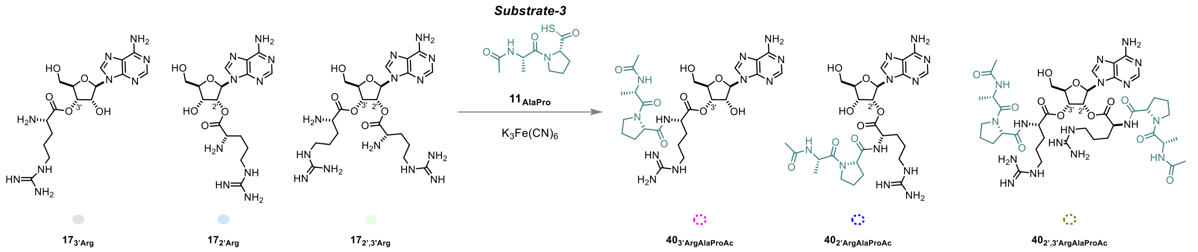

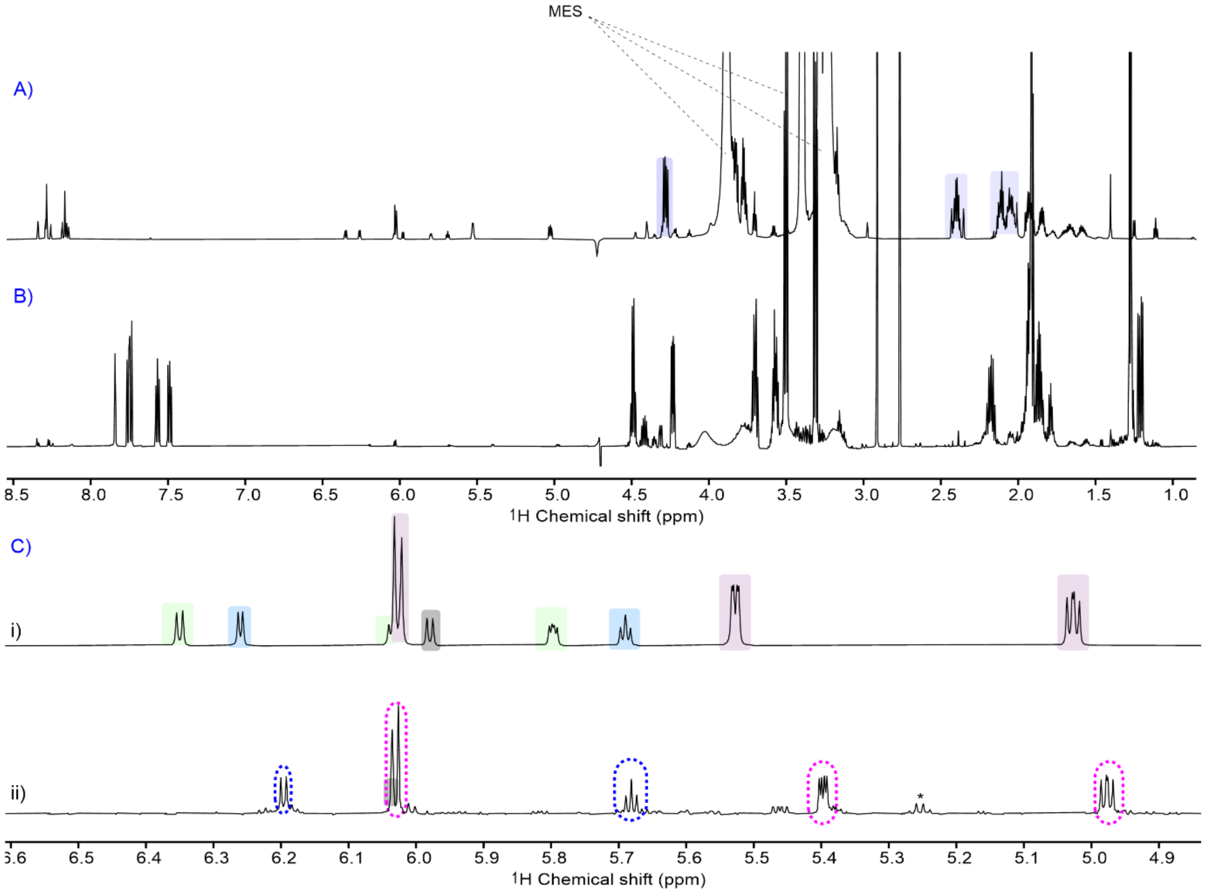

Supplementary Figure 236: ¹H NMR (700 MHz, D_2O, noesygppr1d, 0.5 – 8.0 ppm) spectra showing the synthesis of peptidyl-RNA $\mathbf{40}_{\mathbf{ArgAlaProAc}}$ *via aminoacyl-RNA* $\mathbf{17}^{\mathbf{A}}_{\mathbf{Arg}}$*, formed in situ from cyclic arginine (**33**, 80 mM) and adenosine (**16A**, 20 mM) in MES buffer (500 mM, pD 6.0): A) Spectrum acquired after 3 hours. B) The reaction mixture from spectrum A was treated with $K_3[Fe(CN)_6]$ (600 mM), followed by the addition of Ac-AlaPro-SH (*$\mathbf{11}_{\mathbf{AlaPro}}$*, 200 mM) at pD 6.0. C) Zoom-in of spectra A → (i) and B → (ii) between 4.9–6.6 ppm, with starting material and product signals highlighted. * = The multiplet resonance at 5.25 ppm is a byproduct derived from Ac-AlaPro-SH and is not associated with the nucleoside. In the NMR spectrum, the starting material is highlighted as (■) = **16A**; (■) = **33**.*

¹H NMR (700 MHz, D_2O) $\mathbf{40}^{\mathbf{16A}}_{\mathbf{3'ArgProAlaAc}}$ (partial assignment): δ_H 6.03 (1H, d, *J* = 7.3 Hz, (C1')-H), 5.40 (1H, dd, *J* = 5.4, 2.5 Hz, (C3')-H), 4.98 (1H, dd, *J* = 7.3, 5.4 Hz, (C2')-H);

¹H NMR (700 MHz, D_2O) $\mathbf{40}^{\mathbf{16A}}_{\mathbf{2'ArgProAlaAc}}$ (partial assignment): δ_H 6.20 (1H, d, *J* = 5.7 Hz, (C1')-H), 5.68 (1H, t, *J* = 5.6, (C2')-H);

Synthesis of peptidyl RNA ***$40^{A}_{ArgGlyMetAc}$*** *from aminoacyl-RNA* ***17^{A}_{Arg}*** *with α-amidothioacid* ***11_{MetGly}***

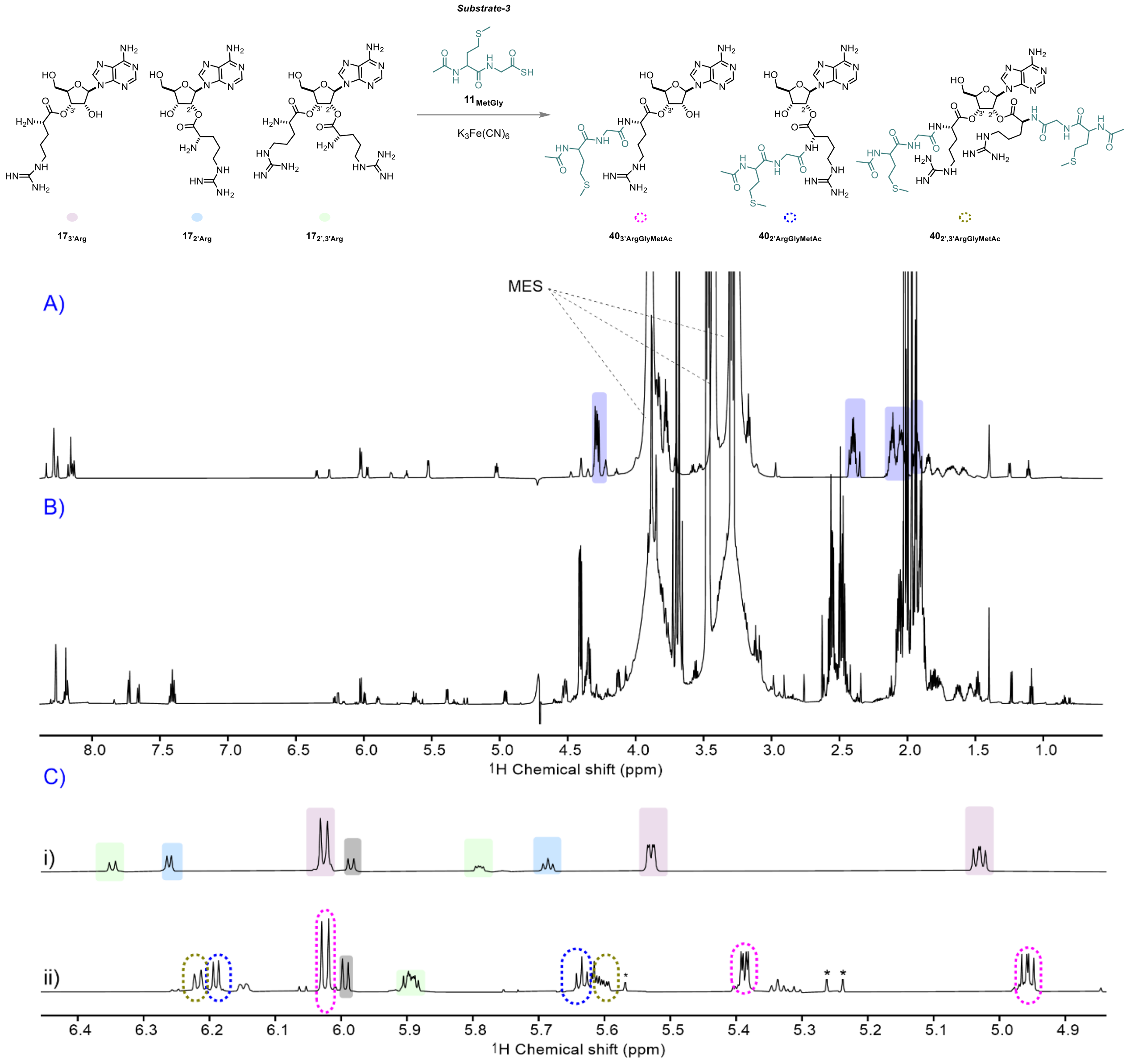

Supplementary Figure 237: ¹H NMR (700 MHz, D_2O, noesygppr1d, 0.5 – 8.0 ppm) spectra showing the synthesis of peptidyl-RNA ***$40_{ArgGlyMetAc}$*** *via aminoacyl-RNA* **17^{A}_{Arg}**, *formed in situ from cyclic arginine (**33**, 80 mM) and adenosine (**16A**, 20 mM) in MES buffer (500 mM, pD 6.0): A) Spectrum acquired after 3 hours. B) The reaction mixture from spectrum A treated with $K_3[Fe(CN)_6]$ (360 mM), followed by the addition of Ac-MetGly-SH (**11_{MetGly}**, 120 mM) at pD 6.0. C) Zoom-in of spectra A→(i) and B→(ii) between 4.9–6.5 ppm, with starting material and product signals highlighted. * = The singlet resonances at 5.25, 5.27 and 5.57 ppm are byproducts derived from Ac-MetGly-SH and are not associated with to the nucleoside. In the NMR spectrum, the starting material is highlighted as (▇) = **16A**; (▇) = **33**.*

¹H NMR (700 MHz, D_2O) $\mathbf{40^{16A}_{3'ArgGlyMetAc}}$ (partial assignment): δ_H 6.02 (1H, d, *J* = 7.4 Hz, (C1')-H), 5.39 (1H, dd, *J* = 5.4, 2.5 Hz, (C3')-H), 4.96 (1H, dd, *J* = 7.4, 5.4 Hz, (C2')-H);

¹H NMR (700 MHz, D_2O) $\mathbf{40^{16A}_{2'ArgGlyMetAc}}$ (partial assignment): δ_H 6.18 (1H, d, *J* = 5.7 Hz, (C1')-H), 5.63 (1H, t, *J* = 5.7 Hz, (C2')-H);

^{1}H NMR (700 MHz, D_2O) $\mathbf{40^{16A}_{2'3'ArgGlyMetAc}}$ (partial assignment): δ_H 6.22 (1H, d, J = 6.9 Hz, (C1')-H), 5.93 – 5.89 (1H, apt., m, (C2')-H), 5.60 (1H, dd, J = 5.3, 2.5 Hz, (C3')-H).

Synthesis of peptidyl RNA $\mathbf{40^{A}_{ArgPheAc}}$ *from aminoacyl-RNA* $\mathbf{17^{A}_{Arg}}$ *with α-amidothioacid* $\mathbf{11_{Phe}}$

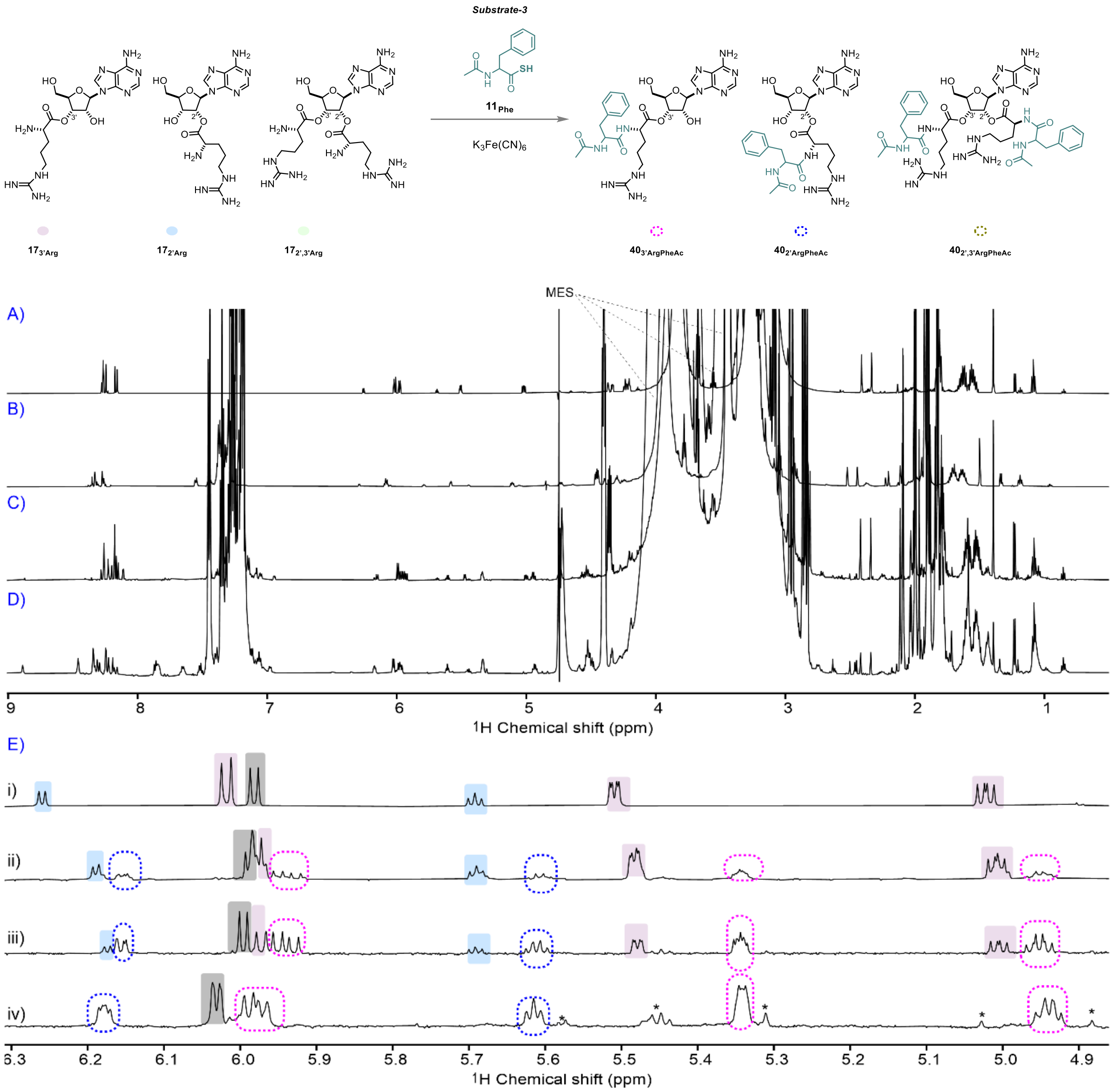

Supplementary Figure 238: ^{1}H NMR (700 MHz, D_2O, noesygppr1d, 0.5 – 9.0 ppm) spectra showing the synthesis of peptidyl-RNA $\mathbf{40_{ArgPheAc}}$ *via aminoacyl-RNA* $\mathbf{17^{A}_{Arg}}$*, formed in situ from of cyclic arginine (**33**, 80 mM) and adenosine (**16A**, 20 mM) in MES buffer (500 mM, pD 6.0): A) Spectrum acquired after 1 hour. B) The reaction mixture from spectrum A treated with $K_3[Fe(CN)_6]$ (300 mM), followed by the portion-wise slow addition of Ac-Phe-SH (*$\mathbf{11_{Phe}}$*, 20 mM). After each addition of* $\mathbf{11_{Phe}}$*, the reaction mixture was sonicated and vortexed for 30 seconds. This process was repeated five times until a total of 100 mM α-amidothioacid* $\mathbf{11_{Phe}}$ *was added. After this NMR was acquired, and the pD was readjusted to 6.0 using 1.5 M MES buffer. C) The reaction mixture from spectrum B treated with $K_3[Fe(CN)_6]$ (300 mM), followed by the portion-wise slow addition of Ac-Phe-SH (*$\mathbf{11_{Phe}}$*, 20 mM). After each addition of* $\mathbf{11_{Phe}}$*, the reaction mixture was sonicated and vortexed for 30 seconds. This process was repeated five times until a total of 100 mM α-amidothioacid* $\mathbf{11_{Phe}}$ *was added. After this NMR was acquired, and the pD was readjusted to 6.0 using 1.5 M MES buffer. D) The reaction mixture from spectrum C treated with $K_3[Fe(CN)_6]$ (300 mM), followed by the portion-wise slow addition of Ac-Phe-SH (*$\mathbf{11_{Phe}}$*, 20 mM). After each addition of* $\mathbf{11_{Phe}}$*, the reaction mixture was sonicated and vortexed for 30 seconds. This process was repeated five times until a total of 100 mM α-amidothioacid* $\mathbf{11_{Phe}}$ *was added. After this NMR was acquired, the pD was readjusted to 6.0 using 1.5 M MES buffer. E) Zoom-in of spectra A → (i), B → (ii), C → (iii) and D → (iv) between 4.9–6.3 ppm, with starting material and product signals highlighted. * = byproducts derived from Ac-Phe-SH and not associated with the nucleoside. In the NMR spectrum, the starting material is highlighted as (■) = **16A**; (■) = **33**.*

^{1}H NMR (700 MHz, D2O) $\mathbf{40}^{\mathbf{16A}}_{\mathbf{3'ArgPheAc}}$ (partial assignment – both diastereomers a + b): δ_H 6.04 (1H, d, *J* = 7.4 Hz, (C1')-Ha + (C1')-Hb), 5.39 (1H, m, overlapped, (C3')-Ha + (C3')-Hb), 4.99 (1H, m, overlapped, (C2')-Ha + (C2')-Hb).

^{1}H NMR (700 MHz, D2O) $\mathbf{40}^{\mathbf{16A}}_{\mathbf{2'ArgPheAc}}$ (partial assignment –– both diastereomers a + b): δ_H 6.23 (1H, m, overlapped, (C1')-Ha + (C1')-Hb), 5.66 ((1H, t, *J* = 5.7, (C2')-Ha + (C2')-Hb).

Synthesis of peptidyl RNA $\mathbf{40}^{\mathbf{A}}_{\mathbf{ArgValAc}}$ *from aminoacyl-RNA* $\mathbf{17}^{\mathbf{A}}_{\mathbf{Arg}}$ *with α-amidothioacid* $\mathbf{11}_{\mathbf{Val}}$

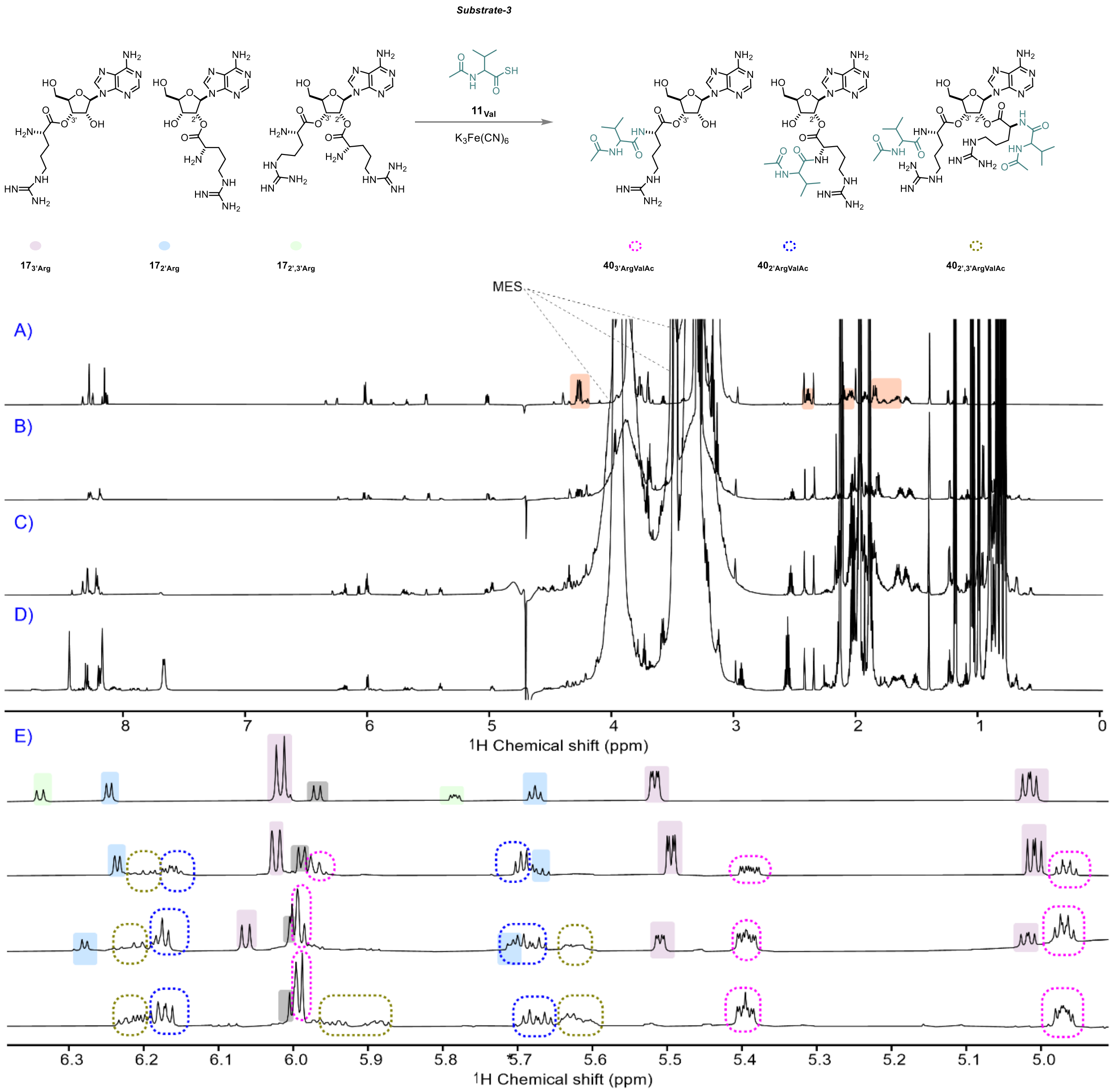

Supplementary Figure 239: ^{1}H NMR (700 MHz, D$_2$*O 9:1, noesygppr1d, 0.5 – 8.0 ppm) spectra showing the synthesis of peptidyl-RNA* $\mathbf{40}_{\mathbf{ArgValAc}}$ *via aminoacyl-RNA* $\mathbf{17}^{A}_{Arg}$*, formed in situ cyclic arginine (**33**, 80 mM) and adenosine (**16A**, 20 mM) in MES buffer (500 mM, pD 6.0): A) Spectrum acquired after 3 hours. B) The reaction mixture from spectrum A treated with* $K_3[Fe(CN)_6]$ *(300 mM), followed by the portion-wise slow addition of Ac-Val-SH (*$\mathbf{11}_{\mathbf{Val}}$*, 20 mM). After each addition of* $\mathbf{11}_{\mathbf{Val}}$*, the reaction mixture was sonicated and vortexed for 30 seconds. This process was repeated five times until a total of 100 mM α-amidothioacid* $\mathbf{11}_{\mathbf{Val}}$ *was added. After this NMR was acquired, the pD was readjusted to 6.0 using 1.5 M MES buffer. C) The reaction mixture from spectrum B treated with* $K_3[Fe(CN)_6]$ *(300 mM), followed by the portion-wise slow addition of Ac-Val-SH (*$\mathbf{11}_{\mathbf{Val}}$*, 20 mM). After each addition of* $\mathbf{11}_{\mathbf{Val}}$*, the reaction mixture was sonicated and vortexed for 30 seconds. This process was repeated five times until a total of 100 mM α-amidothioacid* $\mathbf{11}_{\mathbf{Val}}$ *was added. After this NMR was acquired, the pD was readjusted to 6.0 using 1.5 M MES buffer. D) The reaction mixture from spectrum C treated with* $K_3[Fe(CN)_6]$ *(300 mM), followed by the portion-wise slow addition of Ac-Val-SH (*$\mathbf{11}_{\mathbf{Val}}$*, 20 mM). After each addition of* $\mathbf{11}_{\mathbf{Val}}$*, the reaction mixture was sonicated and vortexed for 30 seconds. This process was repeated five times until a total of 100*

mM α-amidothioacid $\mathbf{11_{Val}}$ *was added. After this NMR was acquired, the pD was readjusted to 6.0 using 1.5 M MES buffer. E) Zoom-in of spectra A → (i), B → (ii), C → (iii) and D → (iv) between 4.9–6.4 ppm, with starting material and product signals highlighted. In the NMR spectrum, the starting material is highlighted as (■) =* ***16A****; (■) =* ***33****.*

^{1}H NMR (700 MHz, D_2O) $\mathbf{40^{16A}_{3'ArgValAc}}$ (partial assignment – both diastereomers a + b): δ_H 5.99 (1H, d, *J* = 7.4 Hz, (C1')-Ha + (C1')-Hb), 5.40 (1H, m, overlapped, (C3')-Ha + (C3')-Hb), 4.98 (1H, m, overlapped, (C2')-Ha + (C2')-Hb).

^{1}H NMR (700 MHz, D_2O) $\mathbf{40^{16A}_{2'ArgValAc}}$ (partial assignment –- both diastereomers a + b): δ_H 6.21 – 6.16 (1H, m, overlapped, (C1')-Ha + (C1')-Hb), 5.70 – 5.68 (1H, t, *J* = 5.7 Hz, (C2')-Ha + (C2')-Hb).

^{1}H NMR (700 MHz, D_2O) $\mathbf{40^{16A}_{2'3'ArgValAc}}$ (partial assignment –- both diastereomers a + b): δ_H 6.27 – 6.20 (1H, m, overlapped, (C1')-Ha + (C1')-Hb), 5.66 – 5.59 (1H, m, overlapped, (C3')-Ha + (C3')-Hb).

Synthesis of peptidyl RNA $\mathbf{40^{A}_{ArgMetAc}}$ *from aminoacyl-RNA* $\mathbf{17^{A}_{Arg}}$ *with α-amidothioacid* $\mathbf{11_{Met}}$

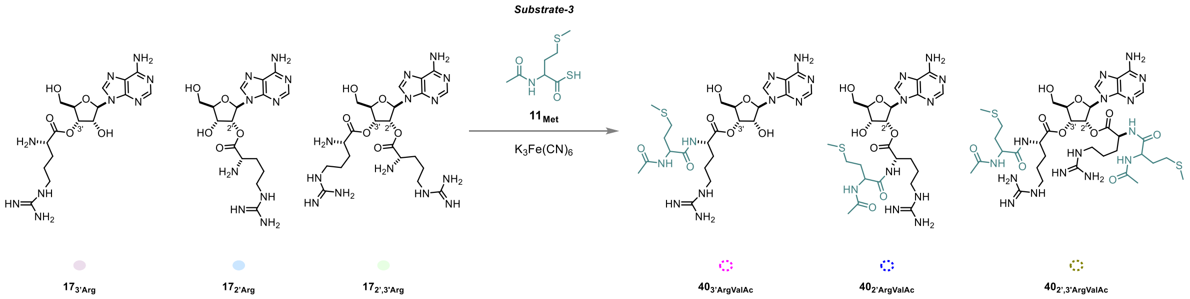

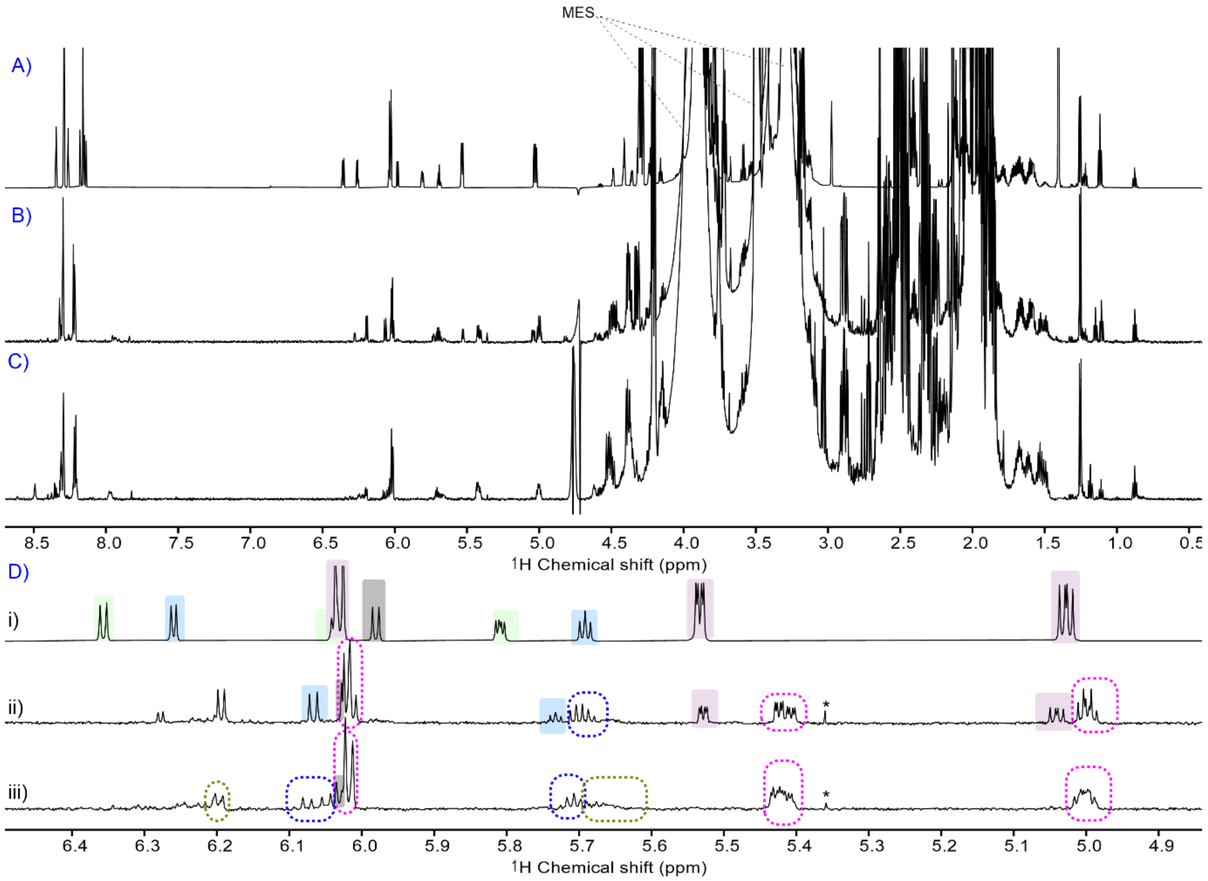

Supplementary Figure 240: ^{1}*H NMR (600 MHz, H_2O/D_2O 9:1, noesygppr1d, 0.5 – 8.0 ppm) spectra showing the synthesis of peptidyl-RNA* $\mathbf{40_{ArgMetAc}}$ *via aminoacyl-RNA* $\mathbf{17^{A}_{Arg}}$*, formed in situ cyclic arginine (**33**, 80 mM) and adenosine (**16A**, 20 mM) in MES buffer (500 mM, pD 6.0): A) Spectrum acquired after 3 hours. B) The reaction mixture from spectrum A treated with $K_3[Fe(CN)_6]$ (600 mM), followed by the portion-wise slow addition of Ac-Met-SH (* $\mathbf{11_{Met}}$*, 20 mM). After each addition of* $\mathbf{11_{Met}}$*, the reaction mixture was sonicated and vortexed for 30 seconds. This process was repeated ten times until a total of 200 mM α-amidothioacid* $\mathbf{11_{Met}}$ *was added. After this NMR was acquired, the pD was readjusted to 6.0 using 1.5 M MES buffer. C) The reaction mixture from spectrum B treated with $K_3[Fe(CN)_6]$ (300 mM), followed by the portion wise slow addition of Ac-Met-SH (* $\mathbf{11_{Met}}$*, 20 mM). After each addition of* $\mathbf{11_{Met}}$*, the reaction mixture was sonicated and vortexed for 30 seconds. This process was repeated five times until a total of 100 mM α-amidothioacid* $\mathbf{11_{Met}}$ *was added. After this NMR was acquired, the pD was readjusted to 6.0 using 1.5 M MES buffer. D) Zoom-in of spectra A → (i); B → (ii) and C → (iii) between 4.9–6.5 ppm, with starting material and product signals highlighted. * = The singlet resonance at 5.37 ppm is a byproduct derived from Ac-Met-SH and is not associated with the nucleoside. In the NMR spectrum, the starting material is highlighted as (■) = **16A**; ( ) = **33**.*

^{1}H NMR (600 MHz, D_2O) $\mathbf{40^{16A}_{3'ArgMetAc}}$ (partial assignment – both diastereomers a + b): δ_H 6.02 (1H, d, J = 7.2 Hz, (C1')-Ha + (C1')-Hb), 5.42 (1H, m, overlapped, (C3')-Ha + (C3')-Hb), 5.00 (1H, m, overlapped, (C2')-Ha + (C2')-Hb).

^{1}H NMR (600 MHz, D_2O) $\mathbf{40^{16A}_{2'ArgMetAc}}$ (partial assignment — both diastereomers a + b): δ_H 6.05 – 6.08 (1H, d, J = 7.2 Hz, (C1')-Ha + (C1')-Hb), 5.70 – 5.68 (1H, m, overlapped, (C2')-Ha + (C2')-Hb).

^{1}H NMR (600 MHz, D_2O) $\mathbf{40^{16A}_{2',3'ArgMetAc}}$ (partial assignment — both diastereomers a + b): δ_H 6.20 (1H, d , J = 6.2 Hz, (C1')-Ha + (C1')-Hb), 5.64 – 5.69 (1H, m, overlapped, (C3')-Ha + (C3')-Hb).

Synthesis of peptidyl RNA $\mathbf{42^{A}_{ArgGlyAc}}$ *from aminoacyl-RNA* $\mathbf{41^{A}_{Arg}}$ *with α-amidothioacid* $\mathbf{11_{Gly}}$

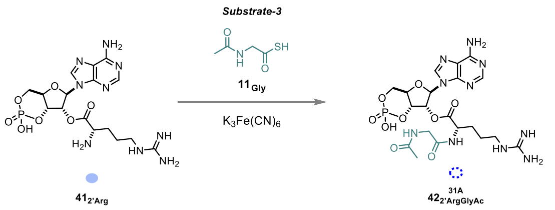

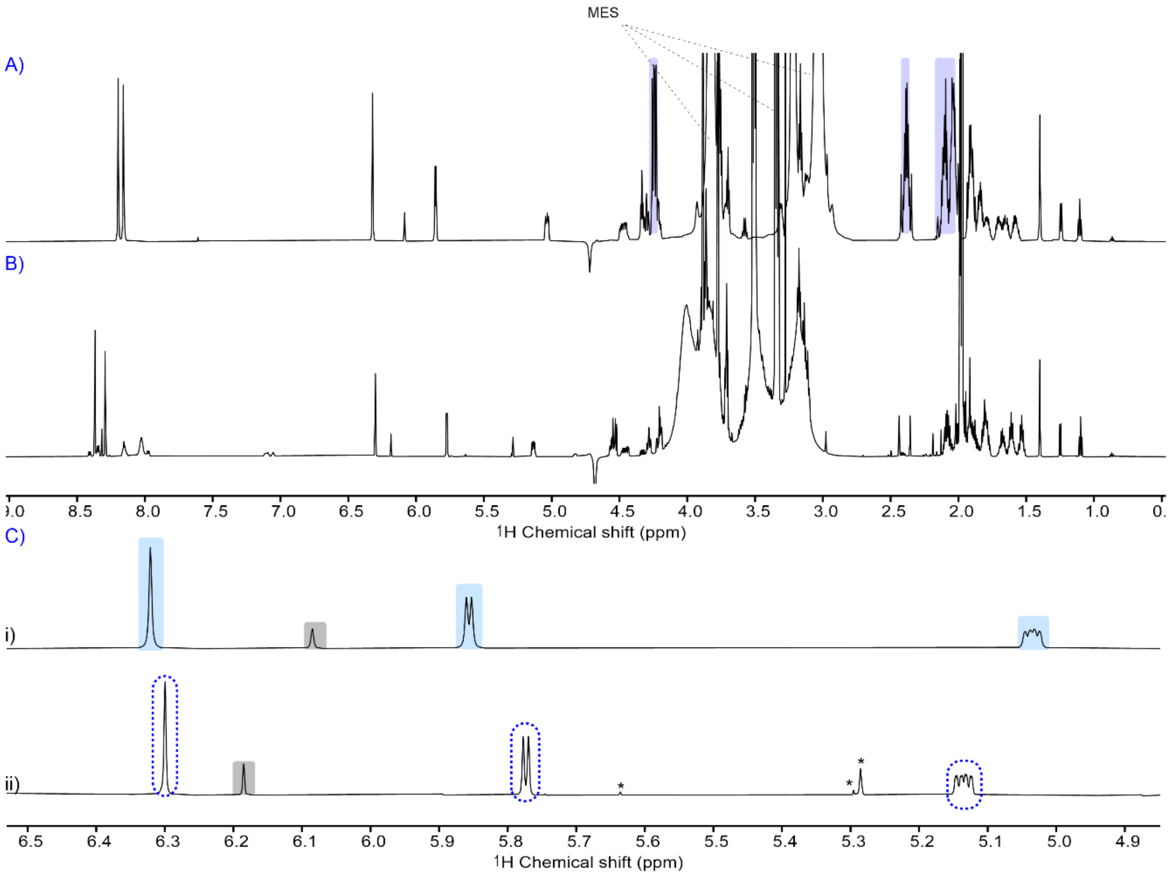

Supplementary Figure 241: ^{1}H NMR (700 MHz, D_2O, noesygppr1d, 0.5 – 9.0 ppm) spectra showing the synthesis of peptidyl-RNA $\mathbf{42_{ArgGlyAc}}$ *via aminoacyl-RNA* $\mathbf{41^{A}_{Arg}}$*, formed in situ of cyclic arginine (**33**, 80 mM) and 3',5'-cAMP (**31A**, 20 mM) in MES buffer (500 mM, pD 6.5, D_2O, 0.5 mL): A) Spectrum acquired after 1 hour. B) The reaction mixture from spectrum A treated with $K_3[Fe(CN)_6]$ (450 mM), followed by the addition of Ac-Gly-SH (**11_{Gly}**, 150 mM) at pD 6.5. C) Zoom-in of spectra A → (i) and B → (ii) between 4.9–6.5 ppm, with starting material and product signals highlighted. * = The singlet resonances at 5.29, 5.30 and 5.65 ppm are byproducts derived from Ac-Gly-SH and are not associated with the nucleoside. In the NMR spectrum, the starting material is highlighted as (▆) = **31A**; (▆) = **33**.*

^{1}H NMR (700 MHz, D$_2$O) $\mathbf{42^{31A}_{2'ArgGlyAc}}$: δ_H 6.30 (1H, s, (C1')-H), 5.77 (1H, d, J = 5.3 Hz, (C2')-H), 5.13 (1H, ddd, J = 10.0, 5.3, 1.9 Hz, (C3')-H)

Synthesis of peptidyl RNA $\mathbf{42^{A}_{ArgGlyGlyAc}}$ *from aminoacyl-RNA* $\mathbf{41^{A}_{Arg}}$ *with α-amidothioacid* $\mathbf{11_{GlyGly}}$

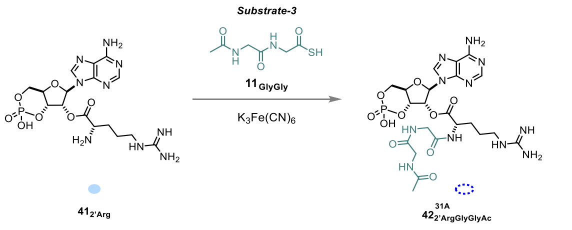

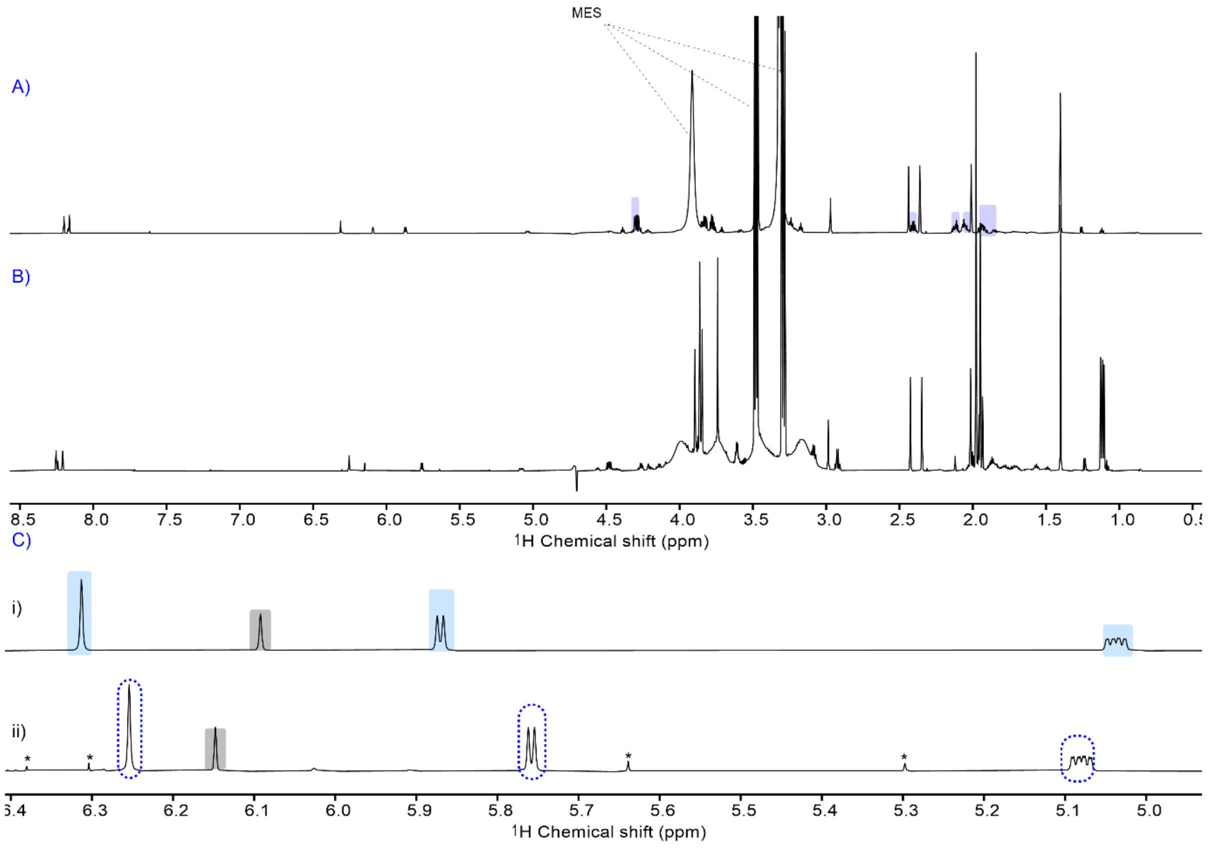

Supplementary Figure 242: ^{1}H NMR (700 MHz, D$_2$O, noesygppr1d, 0.5 – 9.0 ppm) spectra showing the synthesis of peptidyl-RNA $\mathbf{42_{ArgGlyGlyAc}}$ *via aminoacyl-RNA* $\mathbf{41^{A}_{Arg}}$*, formed in situ of cyclic arginine (**33**, 80 mM) and 3',5'-cAMP (**31A**, 20 mM) in MES buffer (500 mM, pD 6.5, D$_2$O (0.5 mL): A) Spectrum acquired after 3 hours. B) The reaction mixture from spectrum A treated with K$_3$[Fe(CN)$_6$] (330 mM), followed by the addition of Ac-GlyGly-SH (**11**$_{GlyGly}$, 110 mM) at pD 6.5. C) Zoom-in of spectra A → (i) and B → (ii) between 4.9–6.5 ppm, with starting material and product signals highlighted. * = The singlet resonances at 5.29, 5.65, 6.30 and 6.38 ppm are byproducts derived from Ac-GlyGly-SH and are not related to the nucleoside. In the NMR spectrum, the starting material is highlighted as (▰) = **31A**; (▰) = **33**.*

^{1}H NMR (700 MHz, D$_2$O) $\mathbf{42^{31A}_{2'ArgGlyGlyAc}}$: δ_H 6.25 (1H, s, (C1')-H), 5.76 (1H, d, J = 5.4 Hz, (C2')-H), 5.08 (1H, ddd, J = 10.0, 5.4, 1.9 Hz, (C3')-H).

Synthesis of peptidyl RNA $\mathbf{42^{A}_{ArgGlyGlyGlyAc}}$ *from aminoacyl-RNA* $\mathbf{41^{A}_{Arg}}$ *with α-amidothioacid* $\mathbf{11_{GlyGlyGly}}$

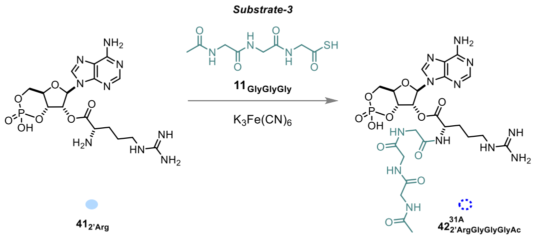

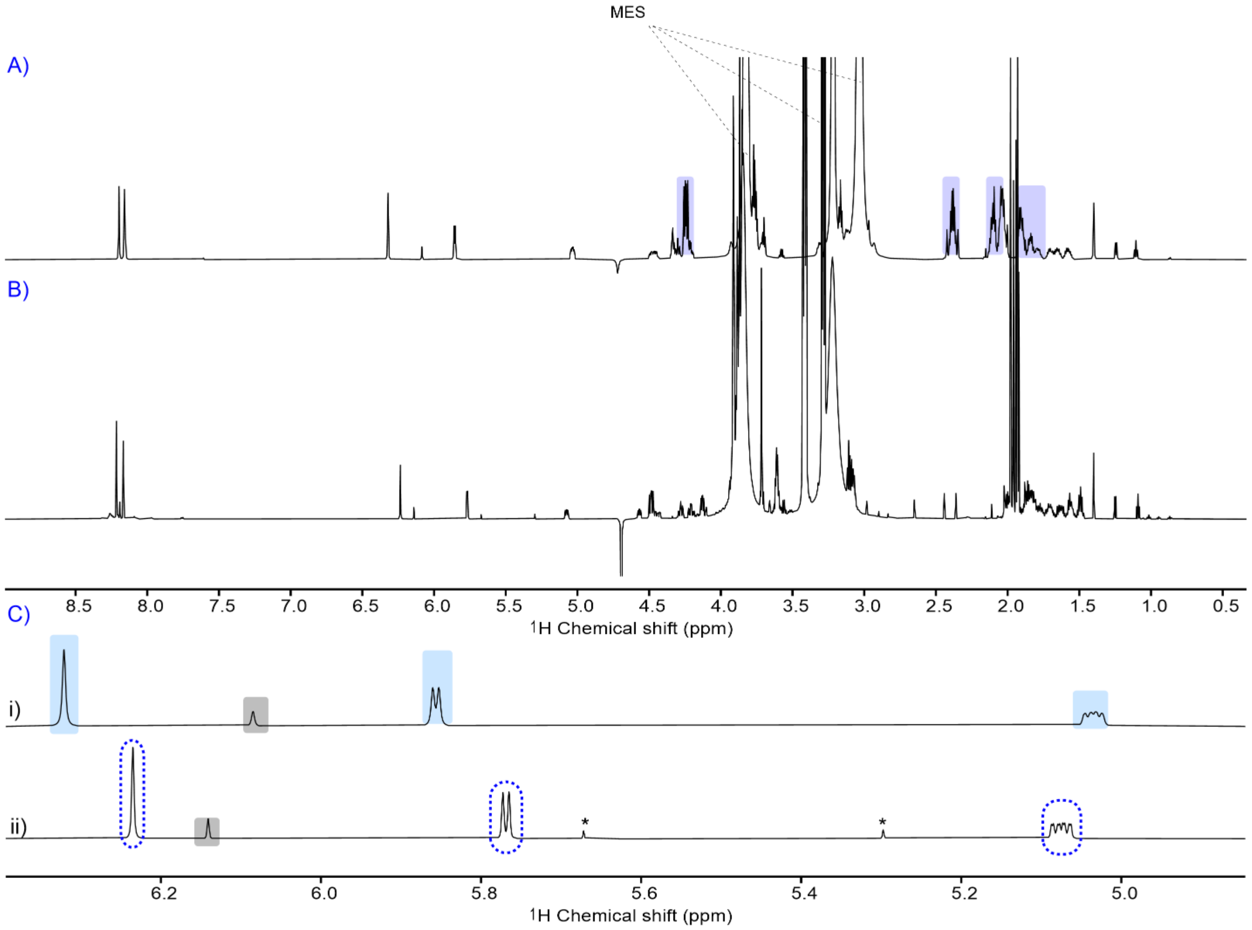

Supplementary Figure 243: ¹H NMR (700 MHz, D_2O, noesygppr1d, 0.5 – 9.0 ppm) spectra showing the synthesis of peptidyl-RNA $\mathbf{42_{ArgGlyGlyGlyAc}}$ *via aminoacyl-RNA* $\mathbf{41^{A}_{Arg}}$*, formed in situ of cyclic arginine (**33**, 80 mM) and 3′,5′-cAMP (**31A**, 20 mM) in MES buffer (500 mM, pD 6.5, D_2O (0.5 mL): A) Spectrum acquired after 3 hours. B) The reaction mixture from spectrum A treated with $K_3[Fe(CN)_6]$ (300 mM), followed by the addition of Ac-GlyGlyGly-SH* ($\mathbf{11_{GlyGlyGly}}$*, 100 mM) at pD 6.5. C) Zoom-in of spectra A ⟶ (i) and B ⟶ (ii) between 4.9–6.3 ppm, with starting material and product signals highlighted. * = The singlet resonances at 5.30 and 5.65 ppm are byproducts derived from Ac-GlyGlyGly-SH and are not related to the nucleoside. In the NMR spectrum, the starting material is highlighted as (▇) = **31A**; (▇) = **33**.*

¹H NMR (700 MHz, D_2O) $\mathbf{40^{31A}_{2'ArgGlyGlyGlyAc}}$: δ_H 6.24 (1H, s, (C1')-H), 5.77 (1H, d, *J* = 5.4 Hz, (C2')-H), 5.08 (1H, ddd, *J* = 10.0, 5.4, 1.9 Hz, (C3')-H).

Synthesis of peptidyl RNA $\mathbf{40^{A}_{ArgGlyAc}}$ *from aminoacyl-RNA* $\mathbf{17^{A}_{Arg}}$ *with α-amidothioacid* $\mathbf{11_{Gly}}$

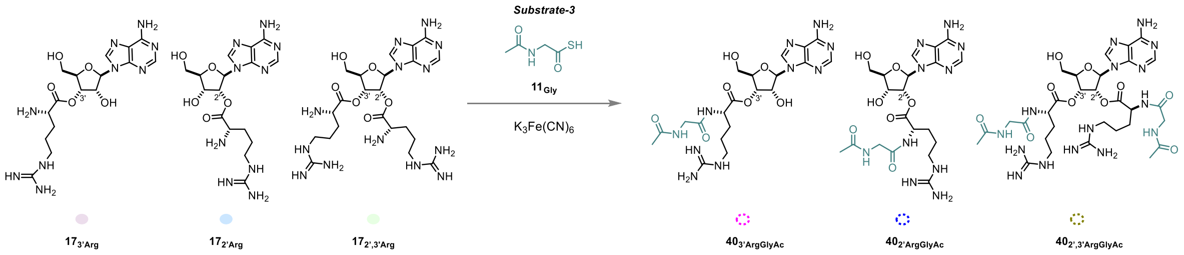

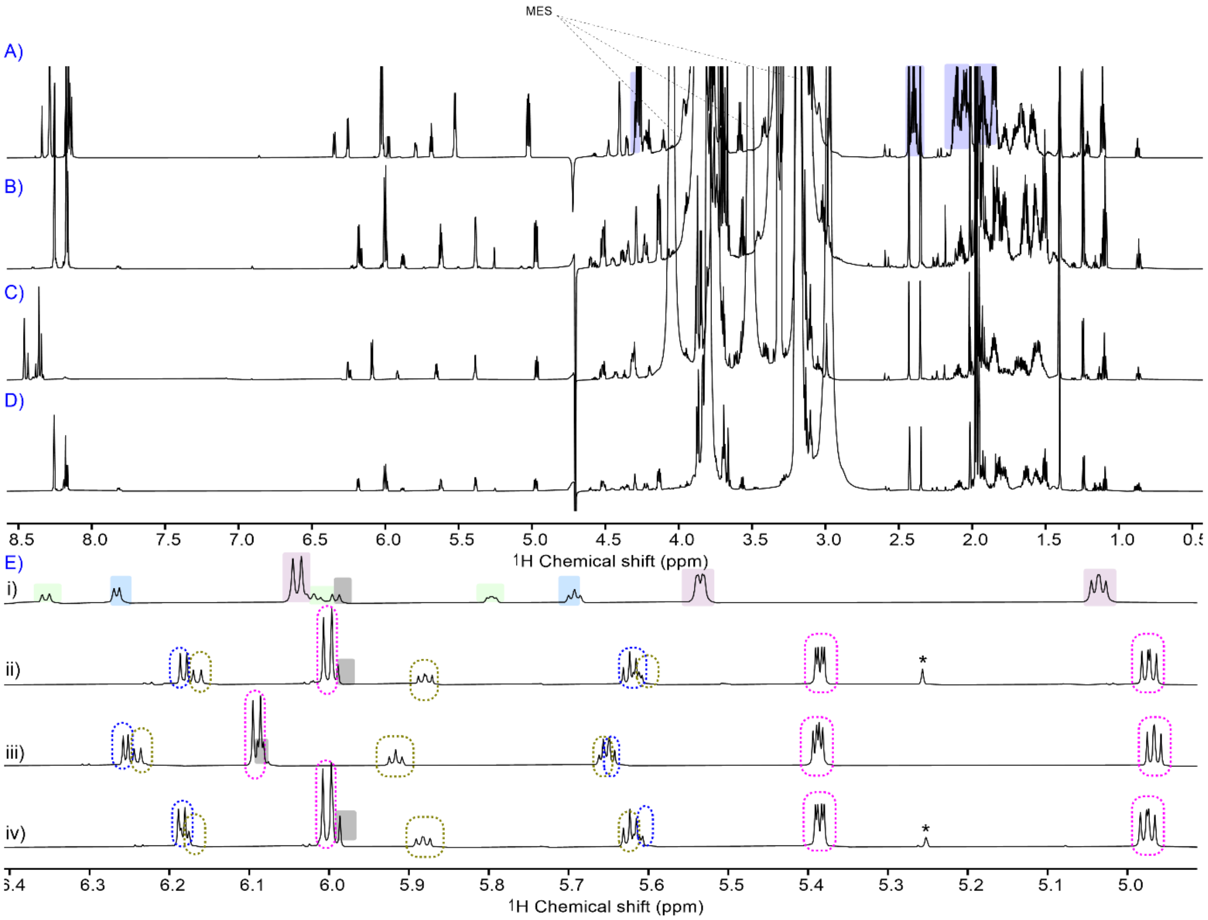

Supplementary Figure 244: ^{1}*H NMR (700 MHz, D*$_2$*O, noesygppr1d, 0.5 – 8.5 ppm) spectra showing the synthesis of peptidyl-RNA* $\mathbf{40_{ArgGlyAc}}$ *via aminoacyl-RNA* $\mathbf{17^{A}_{Arg}}$*, formed in situ from cyclic arginine (**33**, 80 mM) and adenosine (**16A**, 20 mM) in MES buffer (500 mM, pD 6.5): A) Spectrum acquired after 2 hours. B) The reaction mixture from spectrum A treated with K*$_3$*[Fe(CN)*$_6$*] (150 mM), followed by the addition of Ac-Gly-SH (*$\mathbf{11_{Gly}}$*, 50 mM) at pD 6.5. The pD subsequently decreased to ~5.8. C) The reaction mixture from spectrum B acidified to pD 2, in which the singlet resonance byproduct at 5.25 ppm appears to merge with the C2'-H signal. D) The reaction mixture from spectrum C readjusted to pD 6.5, upon which the singlet resonance corresponding to byproduct reappeared. E) Zoom-in of spectra A → (i), B → (ii), C → (iii) and D → (iv) between 4.9–6.5 ppm, with starting material and product signals highlighted. * = The singlet resonance observed at 5.25 ppm corresponds to a byproduct derived from Ac-Gly-SH and is not associated with the nucleoside. In the NMR spectrum, the starting material is highlighted as (■) = **16A**; (■) = **33**.*

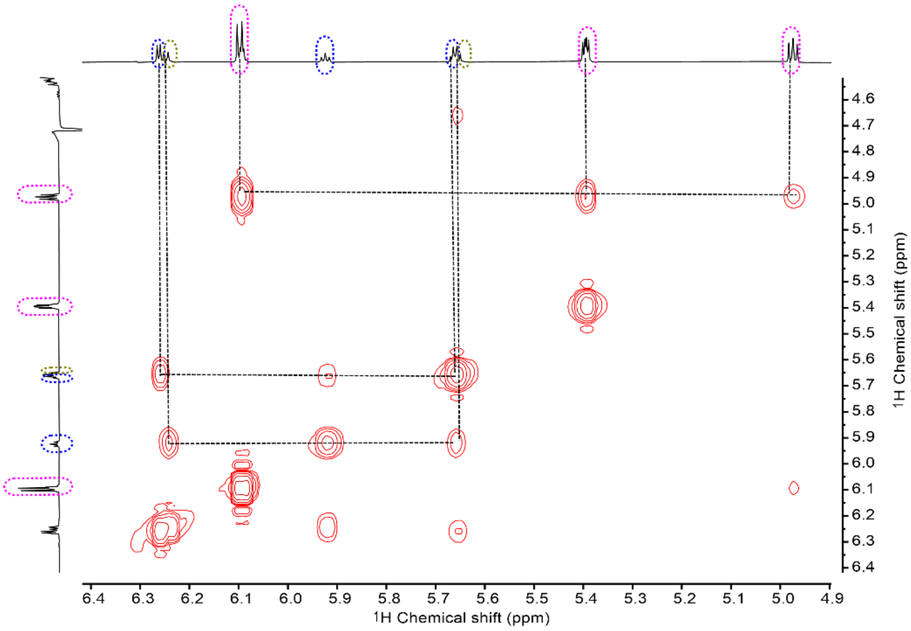

Supplementary Figure 245: ^{1}H–^{1}H Cosy (^{1}H: 700 MHz [4.9 – 6.4 ppm], ^{1}H: 700 MHz [4.5 – 6.4 ppm], D_2O) spectrum showing the diagnostic ^{2}JH, and ^{3}JH coupling in $\mathbf{40}^{16A}_{3'ArgGlyAc}$, $\mathbf{40}^{16A}_{2'ArgGlyAc}$ and $\mathbf{40}^{16A}_{2',3'ArgGlyAc}$.

Synthesis of peptidyl RNA $\mathbf{40^{A}_{ArgGlyGlyGlyAc}}$ *from aminoacyl-RNA* $\mathbf{17^{A}_{Arg}}$ *with α-amidothioacid* $\mathbf{11_{GlyGlyGly}}$

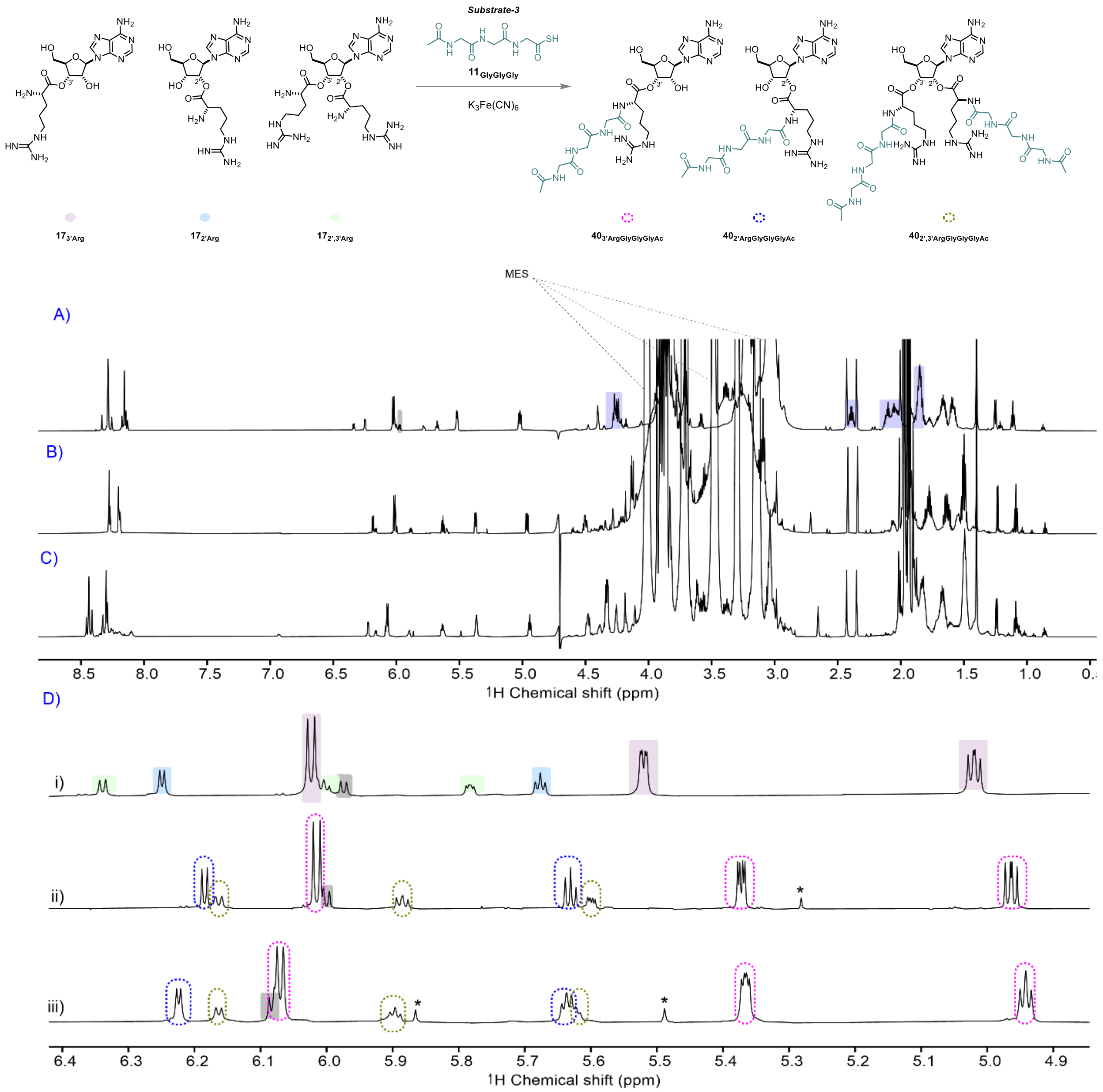

Supplementary Figure 246: ¹*H NMR (700 MHz, D_2O, noesygppr1d, 0.5 – 8.5 ppm) spectra showing the synthesis of peptidyl-RNA* $\mathbf{40_{ArgGlyGlyGlyAc}}$ *via aminoacyl-RNA* $\mathbf{17^{A}_{Arg}}$*, formed in situ from cyclic arginine (**33**, 80 mM) and adenosine (**16A**, 20 mM) in MES buffer (500 mM, pD 6.5): A) Spectrum acquired after 2 hours. B) The reaction mixture from spectrum A treated with $K_3[Fe(CN)_6]$ (300 mM), followed by the addition of Ac-GlyGlyGly-SH (* $\mathbf{11_{GlyGlyGly}}$*, 100 mM) at pD 6.5. The pD subsequently decreased to ~5.50. C) The reaction mixture from spectrum B acidified to pD 2, in which the singlet resonance byproduct at 5.29 ppm appears to move to 5.49 ppm. D) Zoom-in of spectra A → (i), B → (ii) and C → (iii) between 4.9–6.5 ppm, with starting material and product signals highlighted. * = The singlet resonance observed at 5.29 ppm corresponds to a byproduct derived from Ac-GlyGlyGly-SH and is not associated with the nucleoside. In the NMR spectrum, the starting material is highlighted as (▰) = **16A**; (▱) = **33**.*

Synthesis of peptidyl RNA ***40^{A}$_{ArgGlyAc}$*** *from aminoacyl-RNA* ***17^{C}$_{Arg}$*** *with α-amidothioacid* ***11$_{Gly}$***

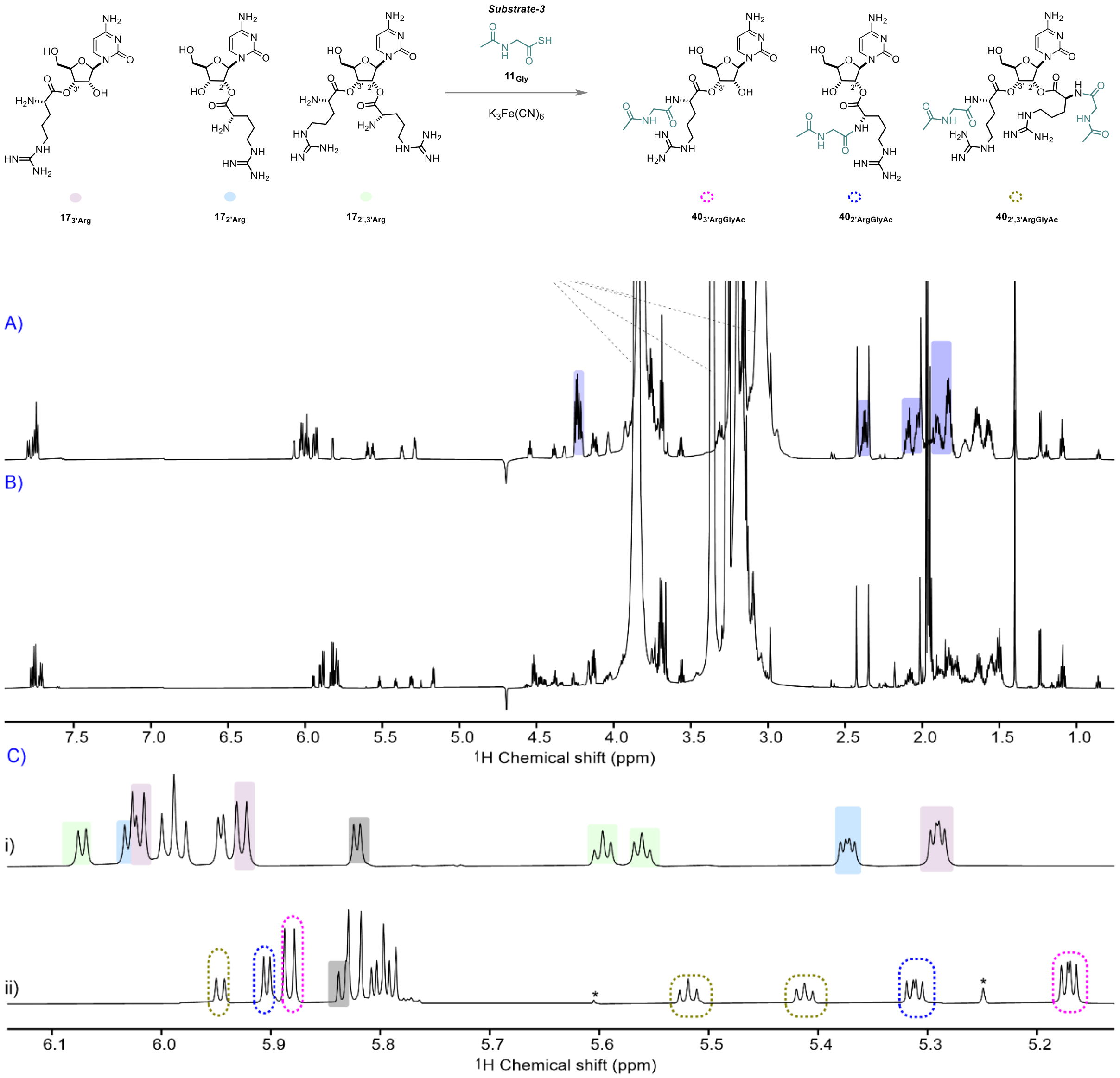

Supplementary Figure 247: ^{1}H NMR (700 MHz, D_2O, noesygppr1d, 0.5 – 8.0 ppm) spectra showing the synthesis of peptidyl-RNA ***40$_{ArgGlyAc}$*** *via aminoacyl-RNA* **17$^{C}_{Arg}$**, *formed in situ from cyclic arginine (**33**, 80 mM) and cytidine (**16C**, 20 mM) in MES buffer (500 mM, pD 6.5): A) Spectrum acquired after 2 hours. B) The reaction mixture from spectrum A treated with $K_3[Fe(CN)_6]$ (300 mM), followed by the addition of Ac-Gly-SH (**11$_{Gly}$**, 100 mM) at pD 6.5. C) Zoom-in of spectra A⟶(i) and B⟶(ii) between 5.1–6.2 ppm, with starting material and product signals highlighted. * = The singlet resonances observed at 5.25 and 5.60 ppm correspond to byproducts derived from Ac-Gly-SH and are not associated with the nucleoside. In the NMR spectrum, the starting material is highlighted as (▆) = **16C**; (▆) = **33.***

Synthesis of peptidyl RNA $\boldsymbol{40^{A}_{ArgGlyAc}}$ *from aminoacyl-RNA* $\boldsymbol{17^{U}_{Arg}}$ *with α-amidothioacid* $\boldsymbol{11_{Gly}}$

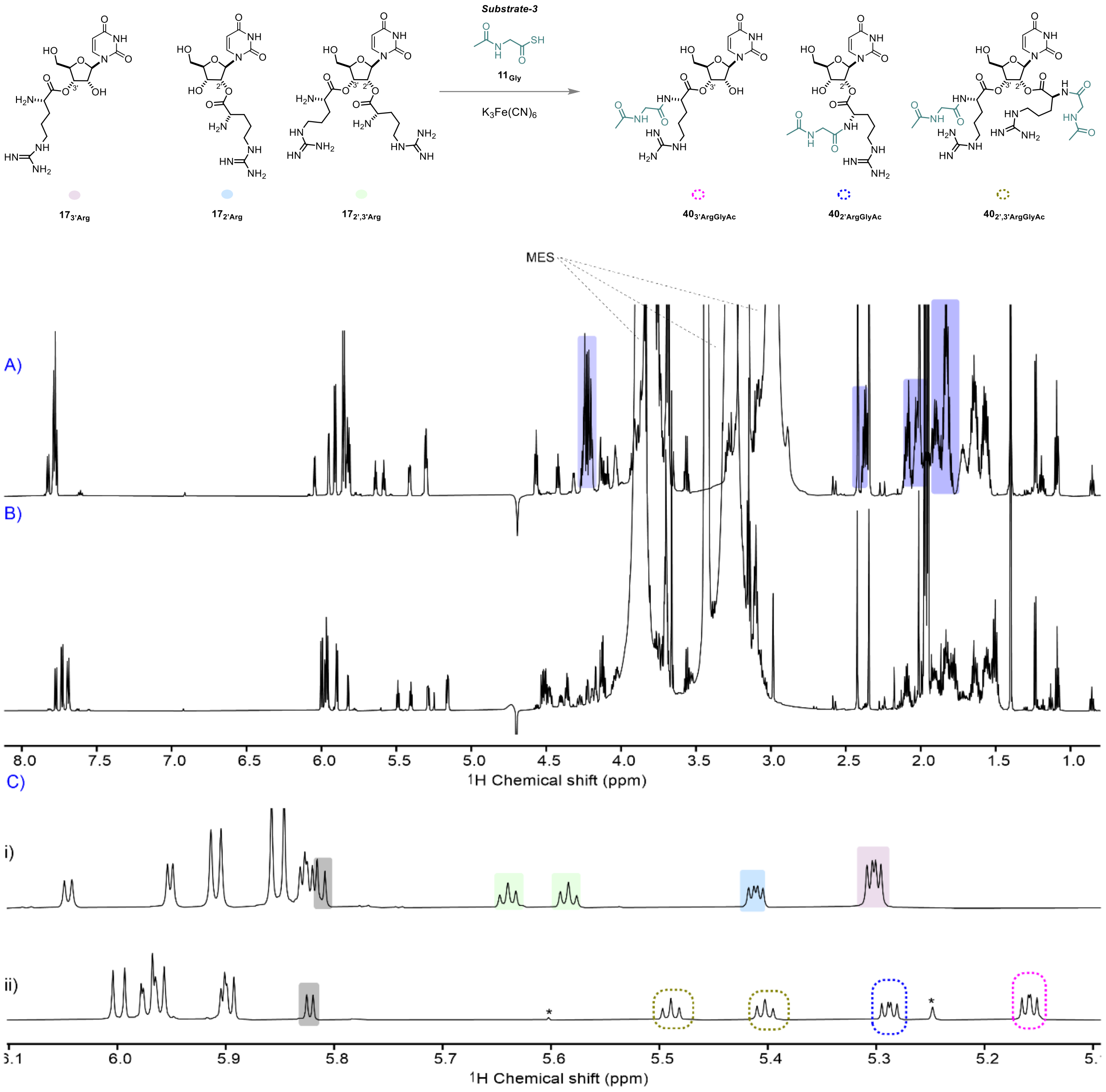

Supplementary Figure 248: ¹H NMR (700 MHz, D₂O, noesygppr1d, 0.5 – 8.0 ppm) spectra showing the synthesis of peptidyl-RNA $\boldsymbol{40_{ArgGlyyAc}}$ *via aminoacyl-RNA* $\boldsymbol{17^{U}_{Arg}}$*, formed in situ from cyclic arginine (**33**, 80 mM) and uridine (**16U**, 20 mM) in MES buffer (500 mM, pD 6.5,* D_2O*, 0.5 mL): A) Spectrum acquired after 2 hours. B) The reaction mixture from spectrum A treated with* $K_3[Fe(CN)_6]$ *(300 mM), followed by the addition of Ac-Gly-SH (*$\boldsymbol{11_{Gly}}$*, 100 mM) at pD 6.5. C) Zoom-in of spectra A→(i) and B → (ii) between 5.1–6.2 ppm, with starting material and product signals highlighted. * = The singlet resonances observed at 5.25 and 5.60 ppm correspond to byproducts derived from Ac-Gly-SH and are not associated with the nucleoside. In the NMR spectrum, the starting material is highlighted as (▆) = **16U**; (▆) = **33**.*

One pot synthesis of peptidyl RNA $\boldsymbol{40^{A}}_{\boldsymbol{ArgGlyAc}}$

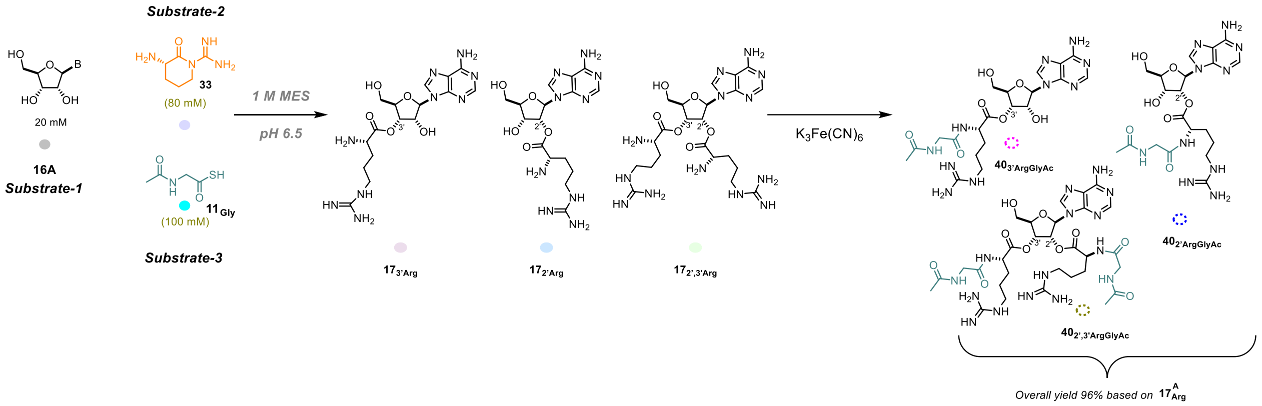

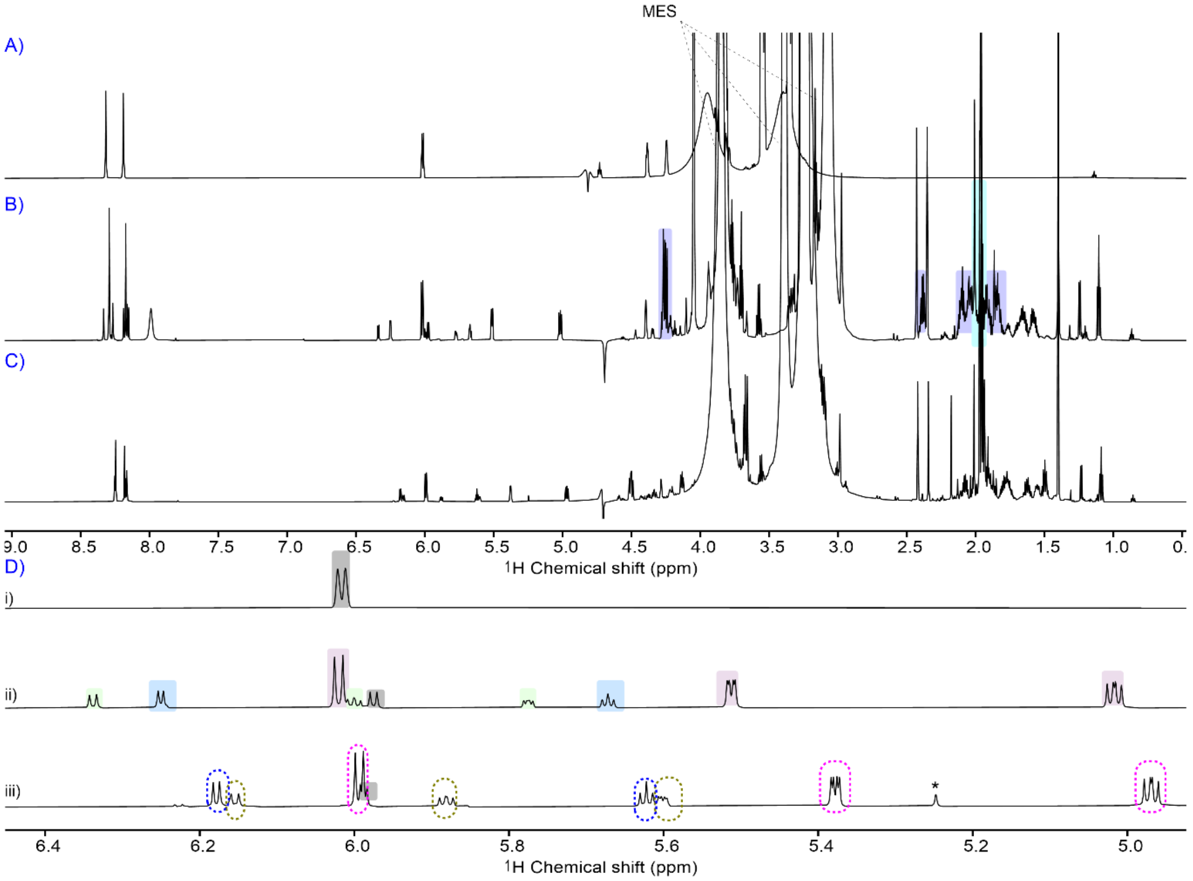

*Supplementary Figure 249: ¹H NMR (700 MHz, D_2O, noesygppr1d, 1.0 – 9.0 ppm) spectra showing the reaction of cyclic arginine (**33**, 80 mM) and Ac-Gly-SH (**11**$_{Gly}$, 100 mM) with adenosine (**16A**, 20 mM) in MES buffer (1 M, pD 6.5, D_2O, 0.5 mL): A) Spectrum acquired after 24 hours (without cyclic arginine, **33** and Ac-Gly-SH, **11**). B) Spectrum acquired after 2 hours with cyclic arginine (**33**, 80 mM) and Ac-Gly-SH (**11**$_{Gly}$, 100 mM). C) The reaction mixture from spectrum B treated with $K_3[Fe(CN)_6]$ (300 mM) at pD 6.5. C) Zoom-in of spectra A → (i), B → (ii) and C → (iii) between 4.9–6.5 ppm, with starting material and product signals highlighted. * = The singlet resonance observed at 5.23 ppm corresponds to a byproduct derived from Ac-Gly-SH and is not associated with the nucleoside.*

One pot synthesis of peptidyl RNA ***40^{A}$_{ArgGlyAc}$***

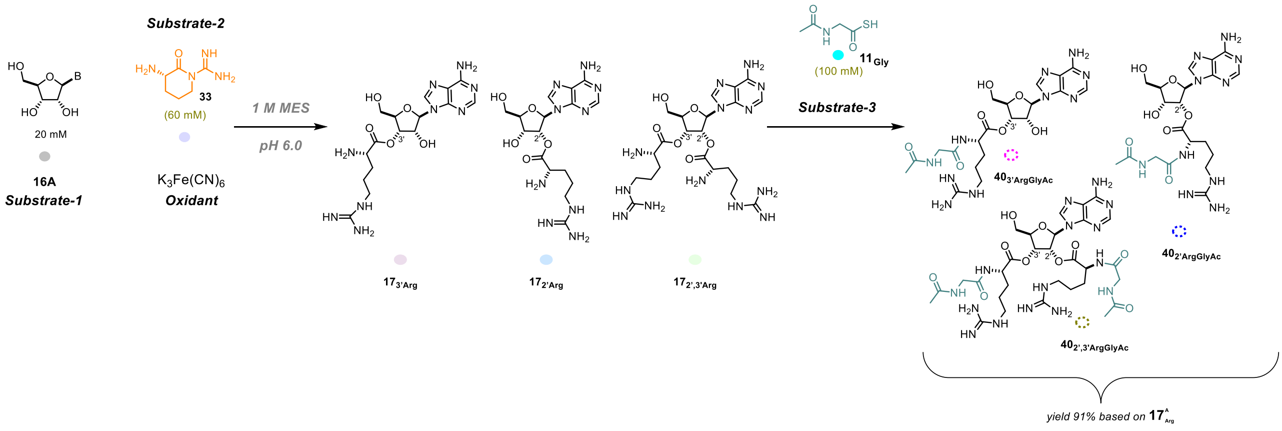

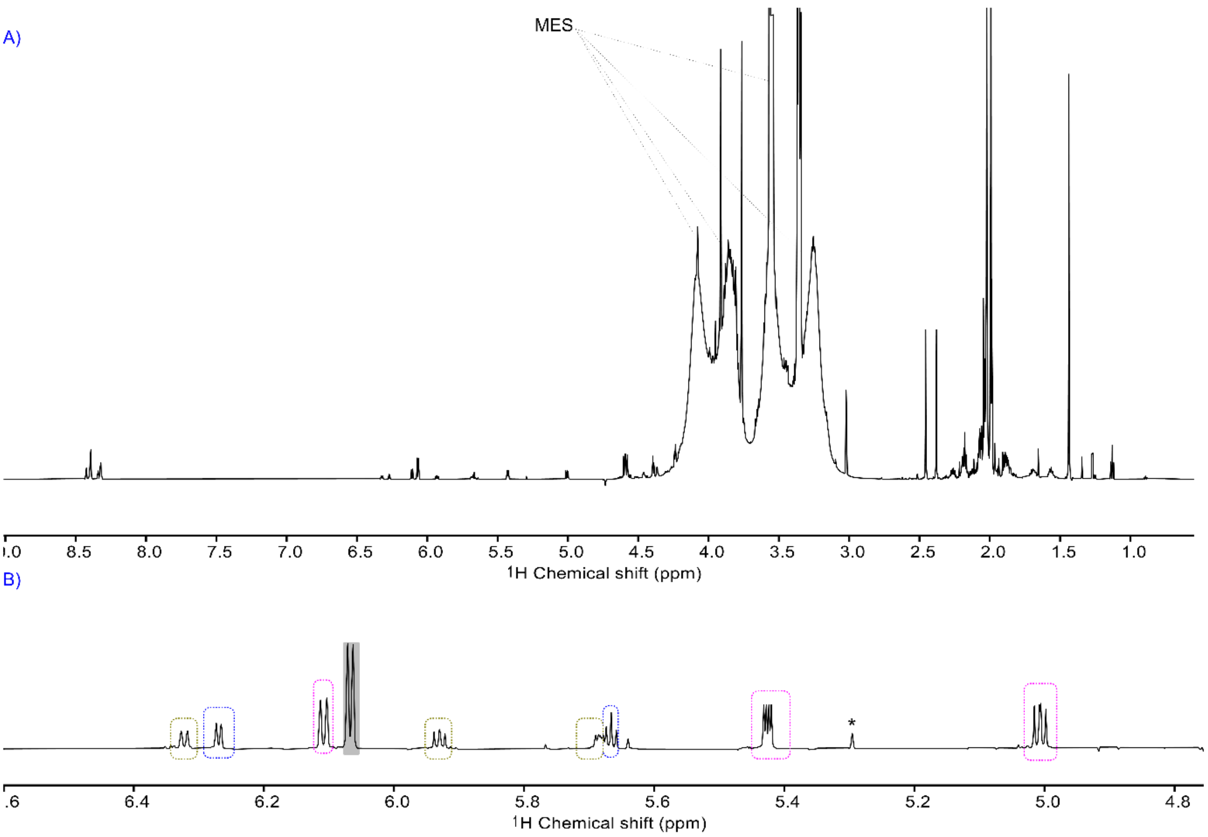

*Supplementary Figure 250: ¹H NMR (700 MHz, D_2O, noesygppr1d, 1.0 – 9.0 ppm) spectrum A showing the reaction of cyclic arginine (**33**, 60 mM) and $K_3[Fe(CN)_6]$ (300 mM) with adenosine (**16A**, 20 mM) in MES buffer (1 M, pD 6.0, D_2O, 0.5 mL) that, after 2 hours, was treated with Ac-Gly-SH (**11$_{Gly}$**, 100 mM) at pD 6.0. B) Zoom-in of spectra A between 4.8–6.6 ppm, with starting material and product signals highlighted. * = The singlet resonances observed at 5.25, 5.61 and 5.79 ppm correspond to byproducts derived from Ac-Gly-SH and are not associated with the nucleoside.*

One pot synthesis of peptidyl RNA $\mathbf{40^{A}}_{\mathbf{AlaGlyAc}}$

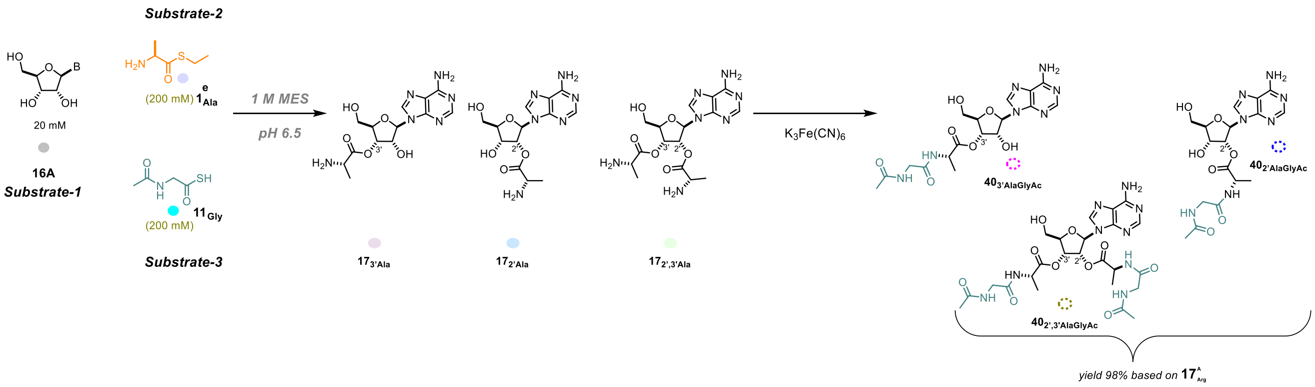

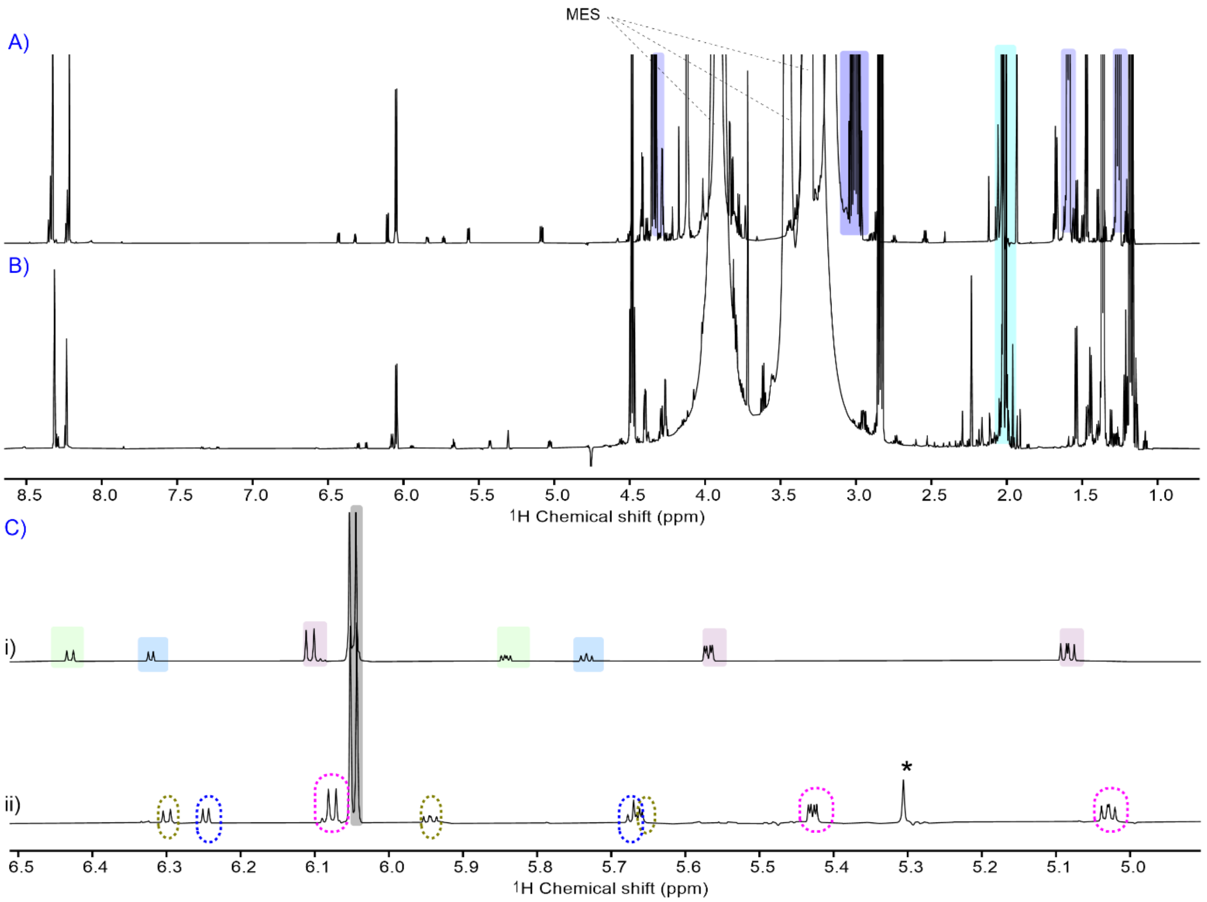

Supplementary Figure 251: ¹H NMR (700 MHz, D₂O, noesygppr1d, 0.5 – 9.0 ppm) spectra showing the reaction of alanyl thioester ($\mathbf{1^{e}_{Ala}}$*, 240 mM) and Ac-Gly-SH (*$\mathbf{11_{Gly}}$*, 240 mM) with adenosine (**16A**, 20 mM) at pD 6.5 in D₂O (0.5 mL): A) Spectrum acquired after 24 hours. B) The reaction mixture from spectrum A treated with* $K_3[Fe(CN)_6]$ *(600 mM) in MES buffer (1 M) at pD 6.5. C) Zoom-in of spectra A → (i) and B → (ii) between 4.9–6.5 ppm, with starting material and product signals highlighted. * = The singlet resonance observed at 5.31 ppm corresponds to a byproduct derived from Ac-Gly-SH and is not associated with the nucleoside.*

Synthesis of peptidyl thioester $\mathbf{39_{GlyAlaAc}}$ *in presence of adenosine* ***16A***

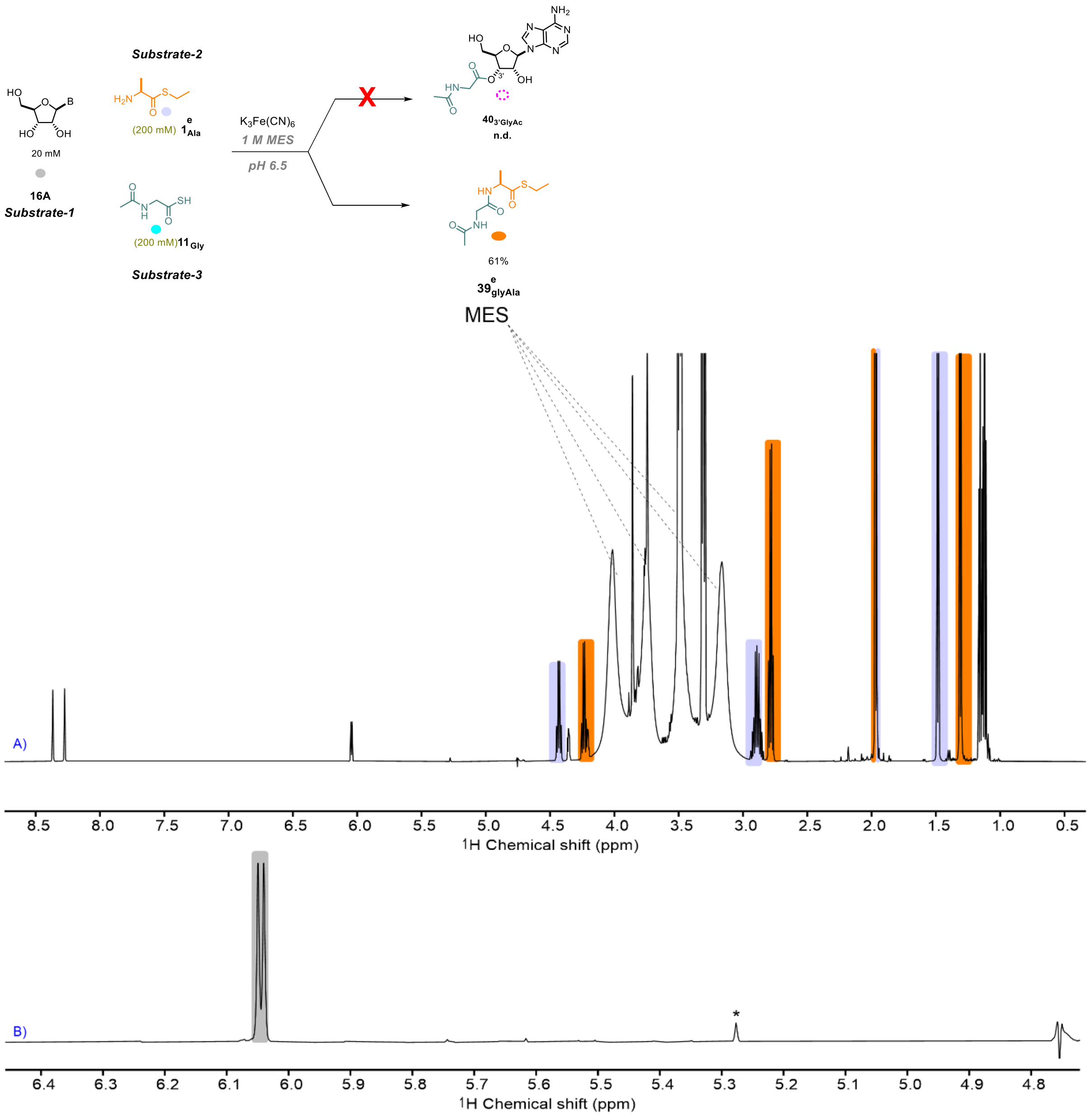

Supplementary Figure 252: ¹*H NMR (700 MHz,* D_2O*, noesygppr1d, 0.5 – 9.0 ppm) spectra showing the reaction of alanyl thioester (*$\mathbf{1^{e}_{Ala}}$*, 240 mM) and Ac-Gly-SH (*$\mathbf{11_{Gly}}$*, 240 mM) with adenosine (**16A**, 20 mM) in MES buffer (1 M) at pD 6.5 in* D_2O *(0.5 mL): A) Spectrum acquired after treatment with* $K_3[Fe(CN)_6]$ *(600 mM). B) Zoom-in of spectra A between 4.9–6.5 ppm, with starting material and product signals highlighted. The spectra indicate that no peptidyl-RNA* $\mathbf{40_{GlyAc}}$ *is formed under these conditions. Even if direct oxidation results in the formation of* $\mathbf{39^{e}_{GlyAlaAc}}$*, the amidoacylation reaction on nucleoside failed. * = The singlet resonance observed at 5.25 ppm corresponds to a byproduct derived from Ac-Gly-SH and is not associated with the nucleoside.*

Competition reaction adenosine with thioester $\mathbf{1_{Ala}}$ *and peptidyl thioester* ***39*** *in presence of adenosine* ***16A***

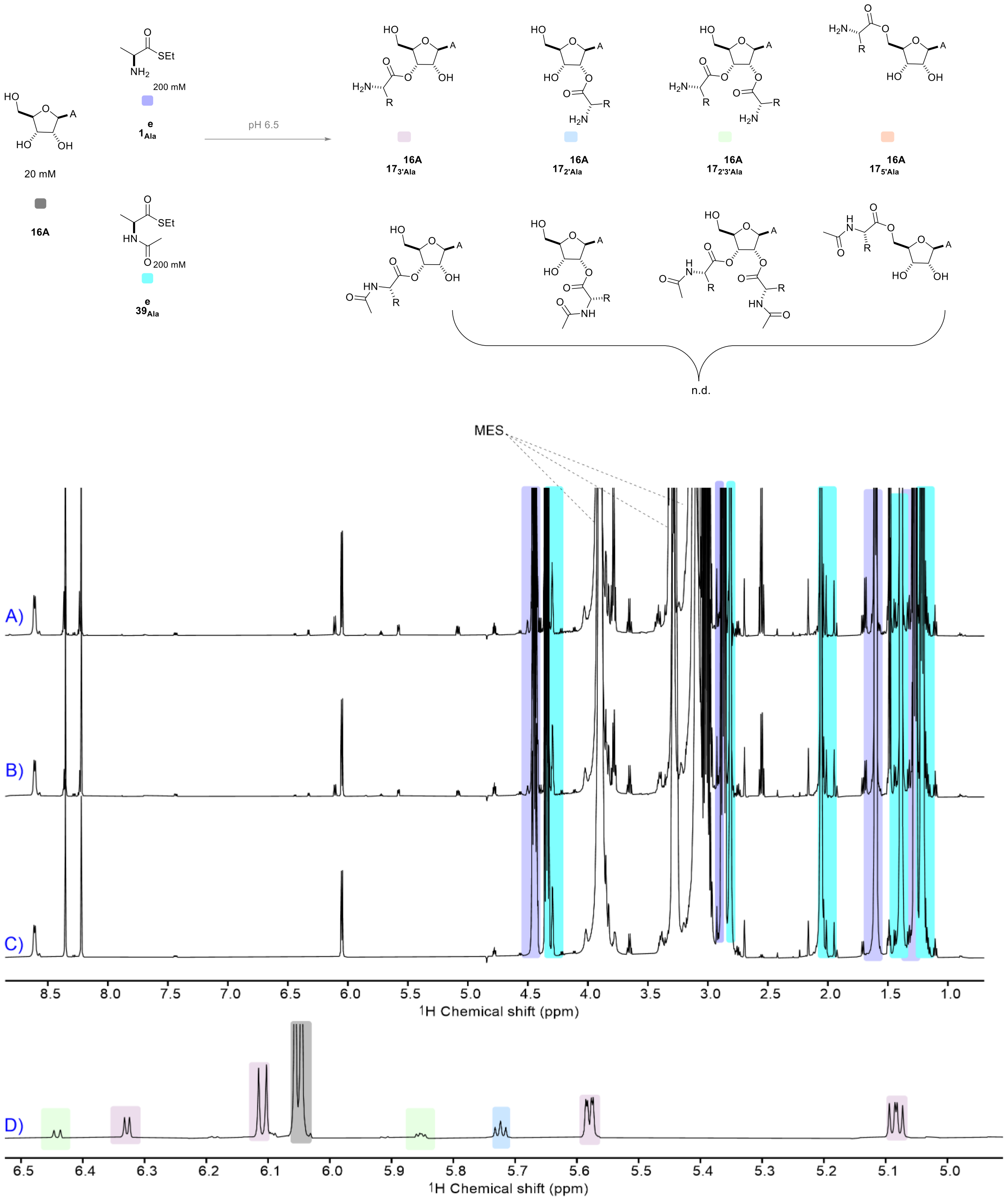

Supplementary Figure 253: ¹H NMR (700 MHz, H_2O/D_2O 9:1, noesygppr1d, 0 – 9.0 ppm) spectra to show the reaction of thioester $\mathbf{1^{e}_{Ala}}$ *(H-Ala-SEt, 200 mM) and amido thioester* $\mathbf{39^{e}_{Ala}}$ *(Ac-Ala-SEt, 200 mM) with adenosine (**16A**, 20 mM) at pH 6.5 in 1M MES buffer: A) 24 h; B) 12 h; C) 1 h D) 5.0–6.6 ppm region of spectrum A). Set up following General Procedure D.*

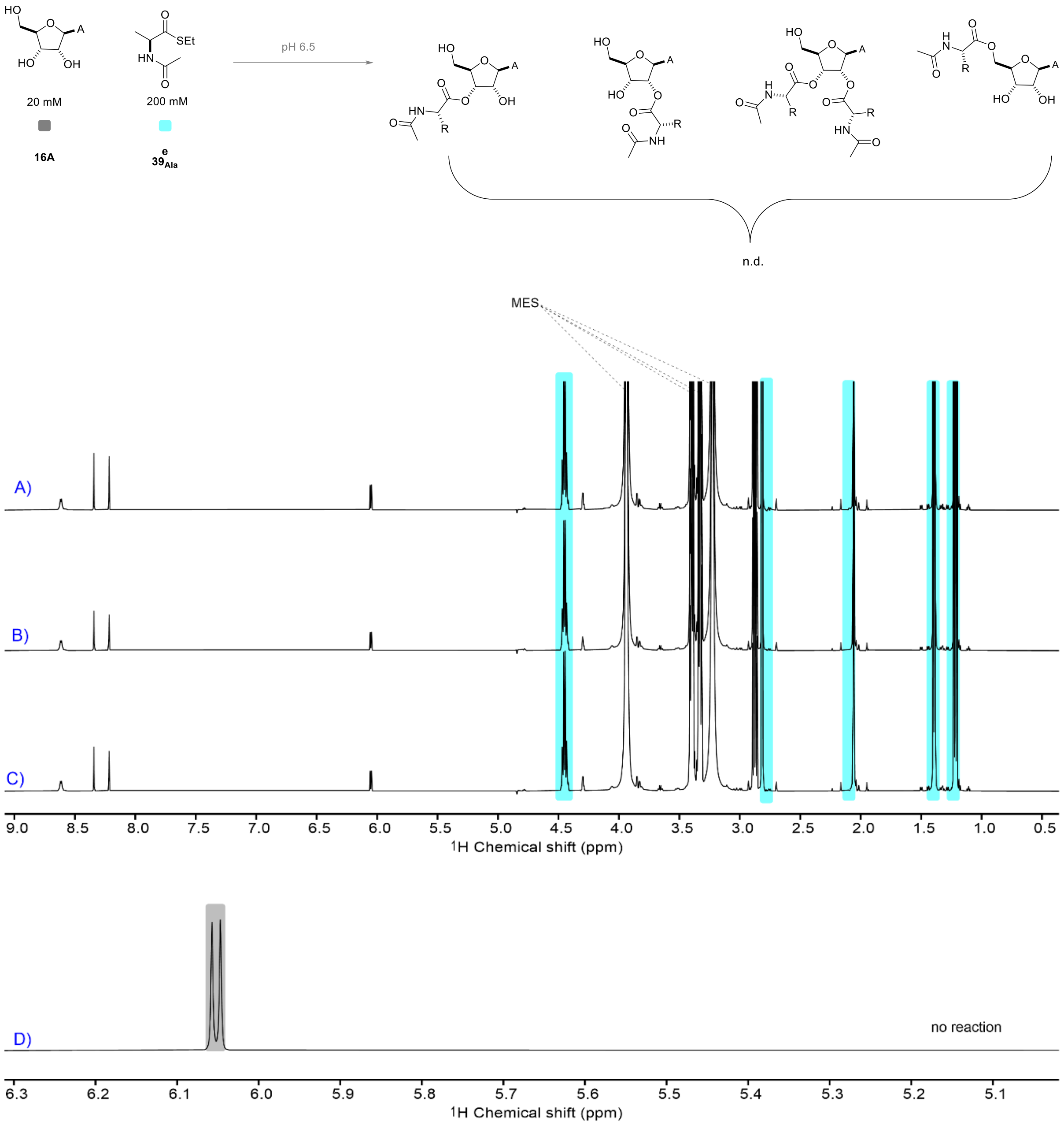

Supplementary Figure 254: ^{1}H NMR (700 MHz, H_2O/D_2O 9:1, noesygppr1d, 0 – 9.0 ppm) spectra to show the reaction of amido thioester $\mathbf{39^{e}_{Ala}}$ *(Ac-Ala-SEt, 200 mM) with adenosine (**16A**, 20 mM) at pH 6.5 in 1M MES buffer: A) 24 h; B) 12 h; C) 1 h D) 4.9–6.3 ppm region of spectrum A). Set up following General Procedure D.*

Synthesis of peptidyl RNA $\mathbf{40^{A}_{GlyGlyAc}}$ *from aminoacyl-RNA* $\mathbf{17^{A}_{Gly}}$ *with α-amidothioacid* $\mathbf{11_{Gly}}$

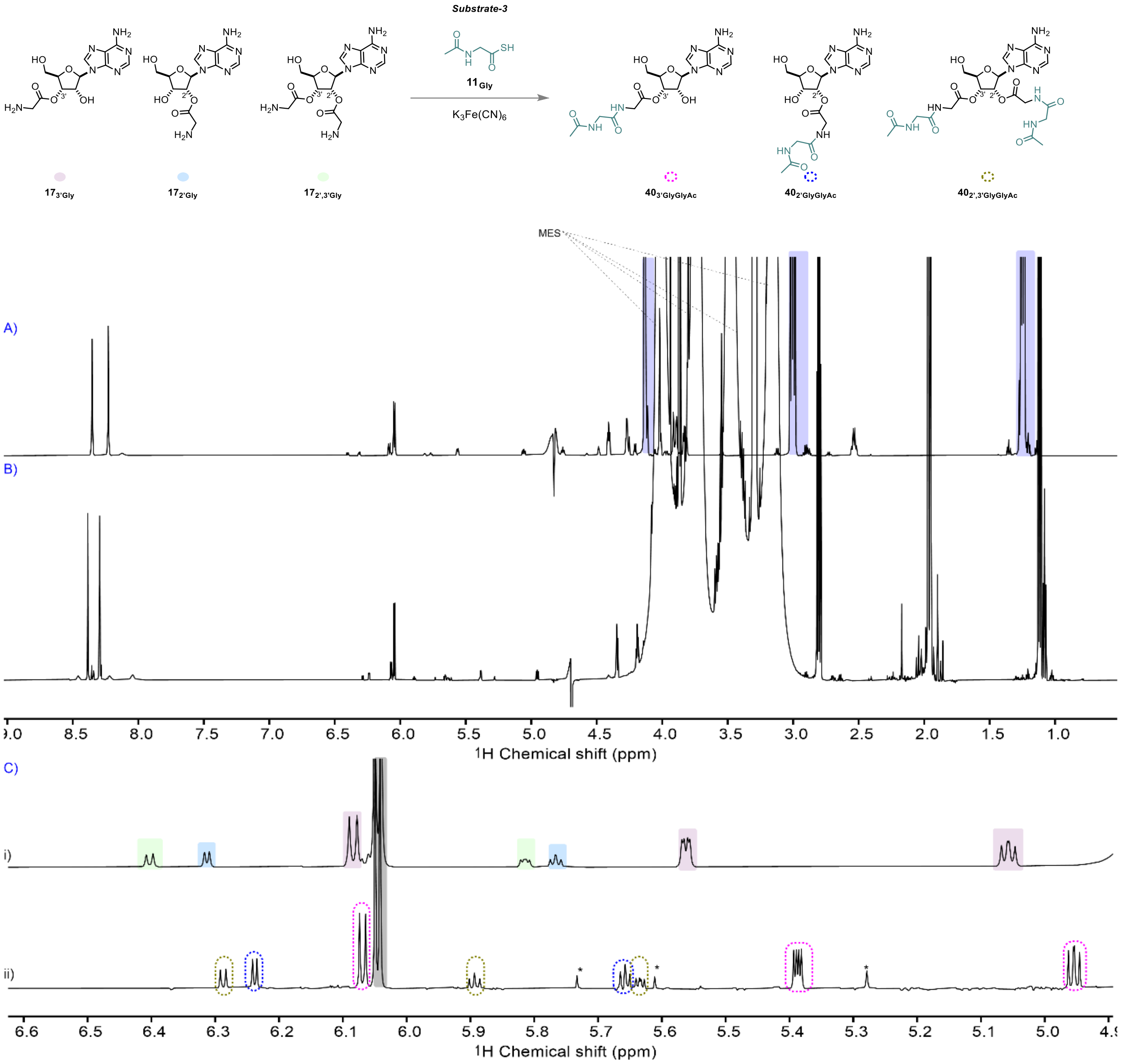

Supplementary Figure 255: ¹*H NMR (700 MHz,* H_2O/D_2O *9:1, noesygppr1d, 0.5 – 9.0 ppm) spectra showing the synthesis of peptidyl-RNA* $\mathbf{40_{GlyGlyAc}}$ *via aminoacyl-RNA* $\mathbf{17^{A}_{Gly}}$*, formed in situ from reaction of glycine thioester (*$\mathbf{1^{e}_{Gly}}$*, 200 mM) and adenosine (**16A**, 20 mM) at pH 6.0.: A) Spectrum acquired after 24 hours. B) The reaction mixture from spectrum A treated with 1 M MES buffer, followed by the addition of Ac-Gly-SH (*$\mathbf{11_{Gly}}$*, 300 mM) and* $K_3[Fe(CN)_6]$ *(900 mM) at pH 6.0. C) Zoom-in of spectra A→(i) and B→(ii) between 4.9–6.6 ppm, with starting material and product signals highlighted. * = The singlet resonances observed at 5.27, 5.60 and 5.73 ppm correspond to byproducts derived from Ac-Gly-SH and are not associated with the nucleoside. Set up following General Procedure L. In the NMR spectrum, the starting material is highlighted as (■) = **16A**; (■) =* $\mathbf{1^{e}_{Gly}}$.

¹H NMR (700 MHz, H_2O/D_2O 9:1) $\mathbf{40^{16A}_{3'GlyGlyAc}}$ (partial assignment): δ_H 6.07 (1H, d, *J* = 6.7 Hz, (C1')-H), 5.39 (1H, dd, *J* = 5.5, 2.9 Hz, (C3')-H), 4.95 (1H, dd, *J* = 6.7, 5.5 Hz, (C2')-H);

¹H NMR (700 MHz, H_2O/D_2O 9:1) $\mathbf{40^{16A}_{2'GlyGlyAc}}$ (partial assignment): δ_H 6.24 (1H, d, *J* = 4.8 Hz, (C1')-H), 5.66 (1H, t, *J* = 5.3 Hz, (C2')-H);

^{1}H NMR (700 MHz, H_2O/D_2O 9:1) $\mathbf{40^{16A}_{2'3'GlyGlyAc}}$ (partial assignment): δ_H 6.29 (1H, d, *J* = 6.0 Hz, (C1')-H), 5.89 (1H, t, *J* = 5.7 Hz, (C3')-H), 5.63 (1H, dd, *J* = 5.4, 3.6 Hz, (C2')-H).

*Synthesis of peptidyl RNA **$40^{A}_{GluGlyAc}$** from aminoacyl-RNA **17^{A}_{Glu}** with α-amidothioacid **11_{Gly}***

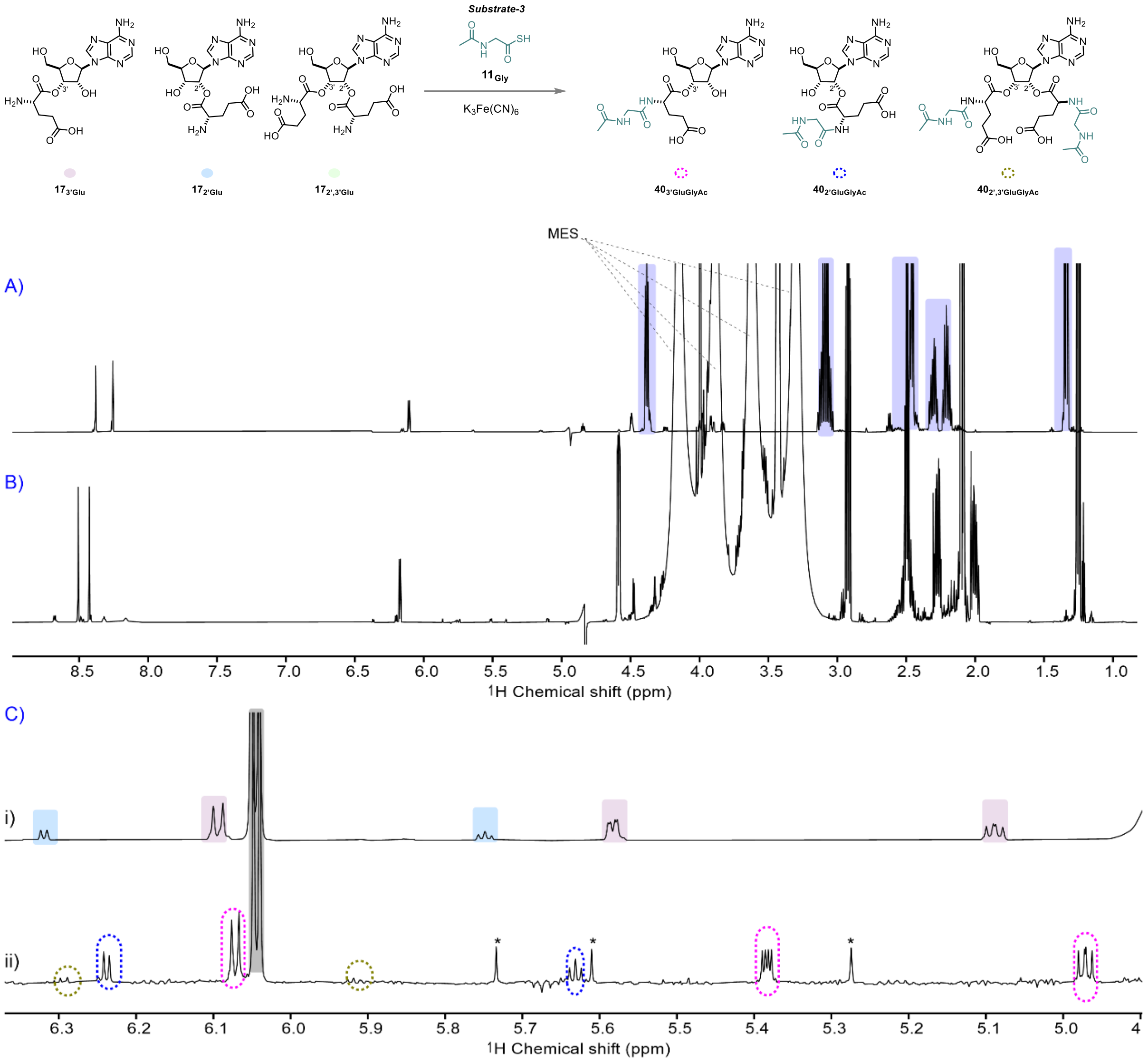

*Supplementary Figure 256: ¹H NMR (700 MHz, H_2O/D_2O 9:1, noesygppr1d, 0.5 – 9.0 ppm) spectra showing the synthesis of peptidyl-RNA **$40_{GluGlyAc}$** via aminoacyl-RNA **$17^{A}_{Glu'}$** formed in situ from reaction of L-glutamic thioester (**$1^{e}_{Glu'}$** 200 mM) and adenosine (**16A**, 20 mM) at pH 6.0: A) Spectrum acquired after 24 hours. B) The reaction mixture from spectrum A treated with 1 M MES buffer, followed by the addition of Ac-Gly-SH (**11_{Gly}**, 300 mM) and $K_3[Fe(CN)_6]$ (900 mM) at pH 6.0. C) Zoom-in of spectra A ⟶ (i) and B ⟶ (ii) between 4.9–6.5 ppm, with starting material and product signals highlighted. * = The singlet resonances observed at 5.27, 5.67 and 5.73 ppm correspond to byproducts derived from Ac-Gly-SH and are not associated with the nucleoside. Set up following General Procedure L. In the NMR spectrum, the starting material is highlighted as (■) = **16A**; (■) = **1^{e}_{Glu}**.*

¹H NMR (700 MHz, H_2O/D_2O 9:1) **$40^{16A}_{3'GluGlyAc}$** (partial assignment): δ_H 6.07 (1H, d, *J* = 6.8 Hz, (C1')-H), 5.38 (1H, dd, *J* = 5.5, 2.9 Hz, (C3')-H), 4.99 (1H, dd, *J* = 6.8, 5.5 Hz, (C2')-H);

¹H NMR (700 MHz, H_2O/D_2O 9:1) **$40^{16A}_{2'GluGlyAc}$** (partial assignment): δ_H 6.24 (1H, d, *J* = 5.0 Hz, (C1')-H), 5.63 (1H, t, *J* = 5.3 Hz, (C2')-H).

^{1}H NMR (700 MHz, H_2O/D_2O 9:1) $\mathbf{40}^{\mathbf{16A}}_{\mathbf{2'3'GluGlyAc}}$ (partial assignment): δ_H 6.29 (1H, d, *J* = 6.2 Hz, (C1')-H), 5.92 (1H, t, *J* = 5.7 Hz, (C3')-H).

*Synthesis of peptidyl RNA **40**$^{A}_{AlaGlyAc}$ from aminoacyl-RNA **17**$^{A}_{Ala}$ with α-amidothioacid **11**$_{Gly}$ at pD 6.5*

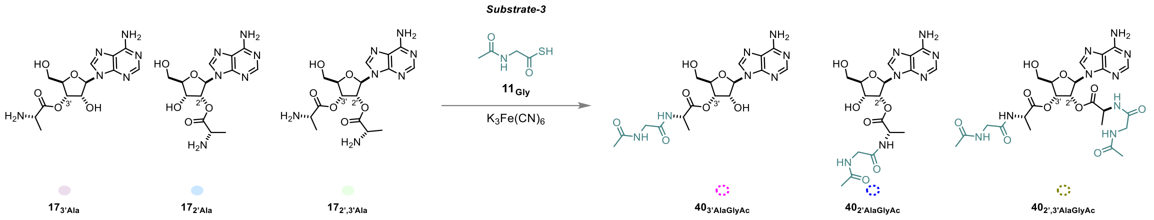

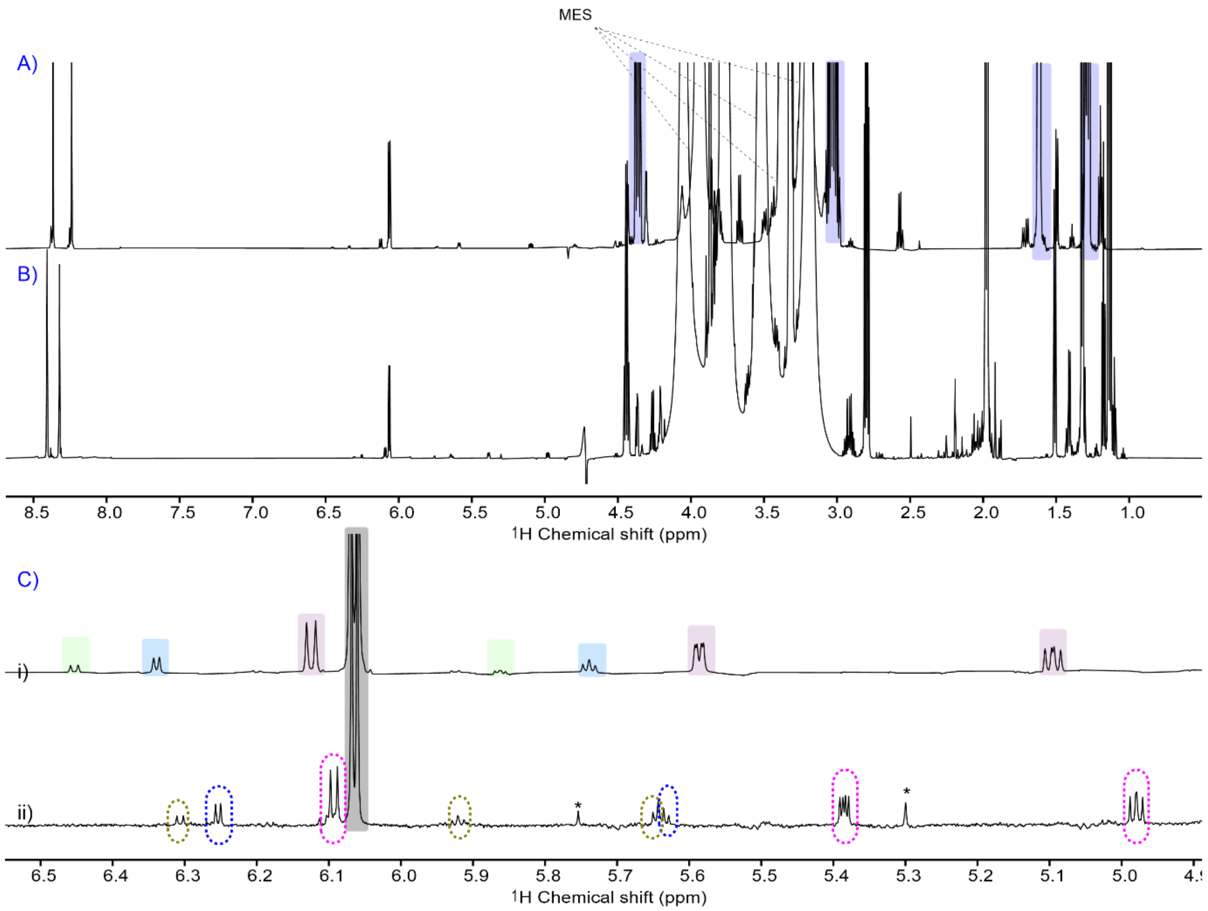

*Supplementary Figure 257: ¹H NMR (700 MHz, D_2O, noesygppr1d, 0.5 – 9.0 ppm) spectra showing the synthesis of peptidyl-RNA **40**$_{AlaGlyAc}$ via aminoacyl-RNA **17**$^{A}_{Ala'}$ formed in situ from reaction of L-alanyl thioester (**1**$^{e}_{Ala'}$ 200 mM) and adenosine (**16A**, 20 mM) in MES buffer (1 M, pD 6.5, D_2O, 0.5 mL): A) Spectrum acquired after 24 hours. B) The reaction mixture from spectrum A treated with $K_3[Fe(CN)_6]$ (900 mM), followed by the addition of Ac-Gly-SH (**11**$_{Gly}$, 300 mM) at pD 6.5. C) Zoom-in of spectra A → (i) and B → (ii) between 4.9–6.5 ppm, with starting material and product signals highlighted. * = The singlet resonances observed at 5.29, and 5.75 ppm correspond to byproducts derived from Ac-Gly-SH and are not associated with the nucleoside. In the NMR spectrum, the starting material is highlighted as (■) = **16A**; (■) = **1**$^{e}_{Ala}$.*

¹H NMR (700 MHz, D_2O) **40**$^{16A}_{3'AlaGlyAc}$ (partial assignment): δ_H 6.07 (1H, d, *J* = 6.7 Hz, (C1')-H), 5.36 (1H, dd, *J* = 5.5, 3.0 Hz, (C3')-H), 4.96 (1H, dd, *J* = 6.7, 5.5 Hz, (C2')-H);

¹H NMR (700 MHz, D_2O) **40**$^{16A}_{2'AlaGlyAc}$ (partial assignment): δ_H 6.23(1H, d, *J* = 4.8 Hz, (C1')-H), 5.62 (1H, app. overlapped, (C2')-H);

^{1}H NMR (700 MHz, D$_2$O) $\mathbf{40^{16A}_{2'3'AlaGlyAc}}$ (partial assignment): δ_H 6.29 (1H, d, *J* = 6.2 Hz, (C1')-H), 5.90 (1H, t, *J* = 5.8 Hz, (C3')-H), 5.62 (1H, app. overlapped, (C2')-H).

*Synthesis of peptidyl RNA **40^A$_{LeuGlyAc}$** from aminoacyl-RNA **17^A$_{Leu}$** with α-amidothioacid **11$_{Gly}$** at pD 6.5*

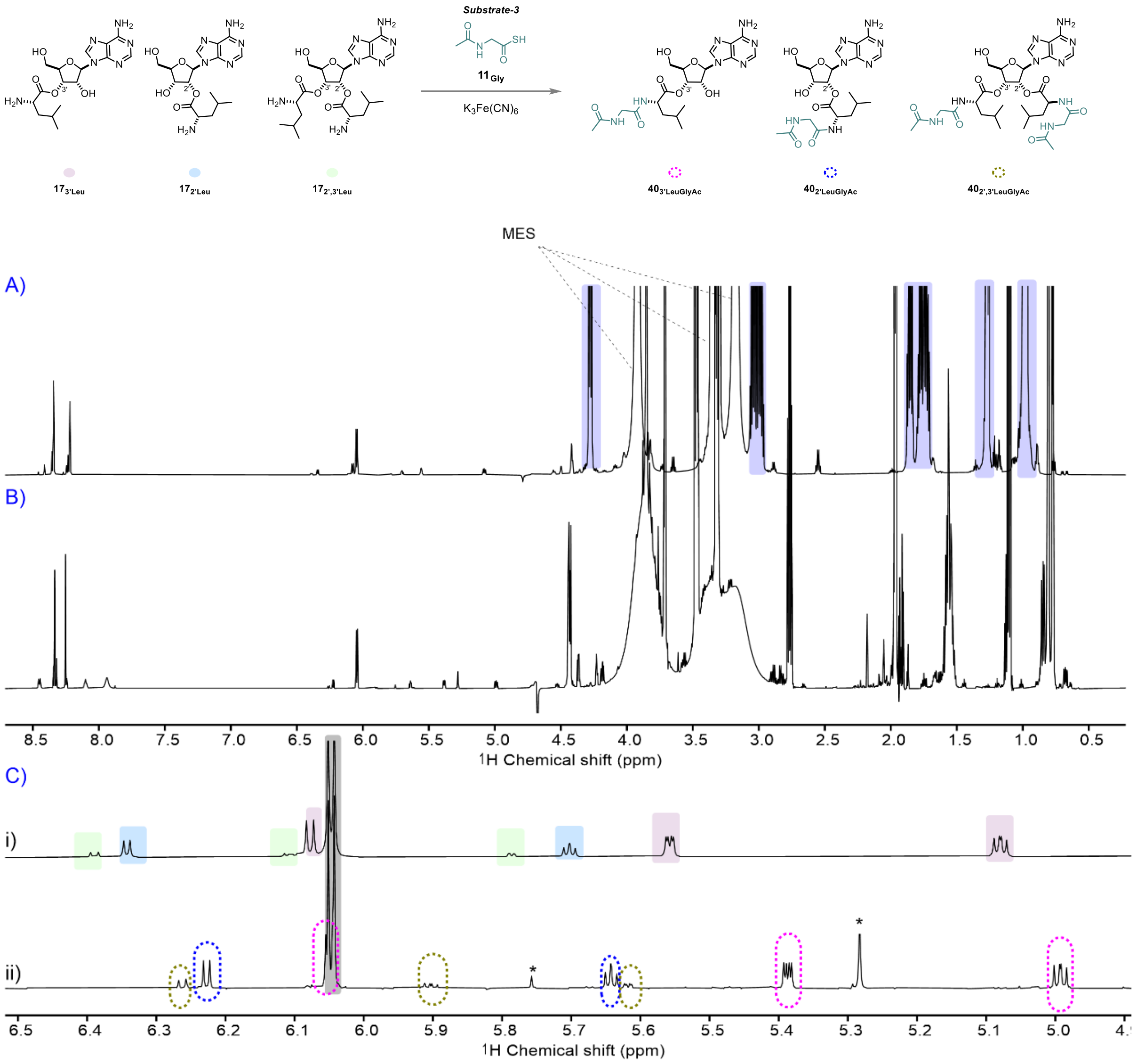

*Supplementary Figure 258: ^{1}H NMR (700 MHz, D$_2$O, noesygppr1d, 0.5 – 9.0 ppm) spectra showing the synthesis of peptidyl-RNA **40$_{LeuGlyAc}$** via aminoacyl-RNA **17$^A_{Leu}$**, formed in situ from reaction of L-leucine thioester (**1^e_{Leu}**, 200 mM) and adenosine (**16A**, 20 mM) in MES buffer (1 M, pD 6.5, D$_2$O, 0.5 mL): A) Spectrum acquired after 24 hours. B) The reaction mixture from spectrum A treated with K$_3$[Fe(CN)$_6$] (1.05 M), followed by the addition of Ac-Gly-SH (**11$_{Gly}$**, 350 mM) at pD 6.5. C) Zoom-in of spectra A → (i) and B → (ii) between 4.9–6.5 ppm, with starting material and product signals highlighted. * = The singlet resonances observed at 5.28 and 5.75 ppm correspond to byproducts derived from Ac-Gly-SH and are not associated with the nucleoside. In the NMR spectrum, the starting material is highlighted as (■) = **16A**; (■) = **1^e_{Leu}**.*

^{1}H NMR (700 MHz, D$_2$O) $\mathbf{40^{16A}_{3'LeuGlyAc}}$ (partial assignment): δ_H 6.05 (1H, d, *J* = 6.7 Hz, (C1')-H), 5.38 (1H, dd, *J* = 5.5, 2.6 Hz, (C3')-H), 4.99 (1H, dd, *J* = 7.0, 5.5 Hz, (C2')-H);

^{1}H NMR (700 MHz, D$_2$O) $\mathbf{40^{16A}_{2'LeuGlyAc}}$ (partial assignment): δ_H 6.23 (1H, d, *J* = 6.3 Hz, (C1')-H), 5.64 (1H, t, *J* = 5.3 Hz, (C2')-H);

^{1}H NMR (700 MHz, D_2O) $\mathbf{40^{16A}_{2'3'LeuGlyAc}}$ (partial assignment): δ_H 6.26 (1H, d, J = 7.5 Hz, (C1')-H), 5.90 (1H, dd, J = 7.5, 5.3 Hz, (C3')-H), 5.62 (1H, dd, J = 5.3, 2.0 Hz, (C2')-H).

Synthesis of peptidyl RNA $\mathbf{40^{A}_{SerGlyAc}}$ *from aminoacyl-RNA* $\mathbf{17^{A}_{Ser}}$ *with α-amidothioacid* $\mathbf{11_{Gly}}$ *at pH 6.0*

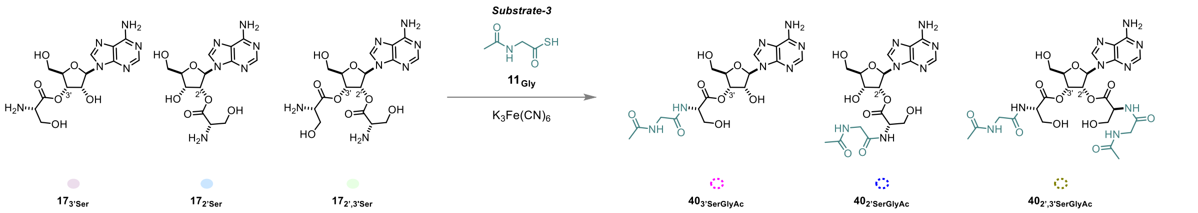

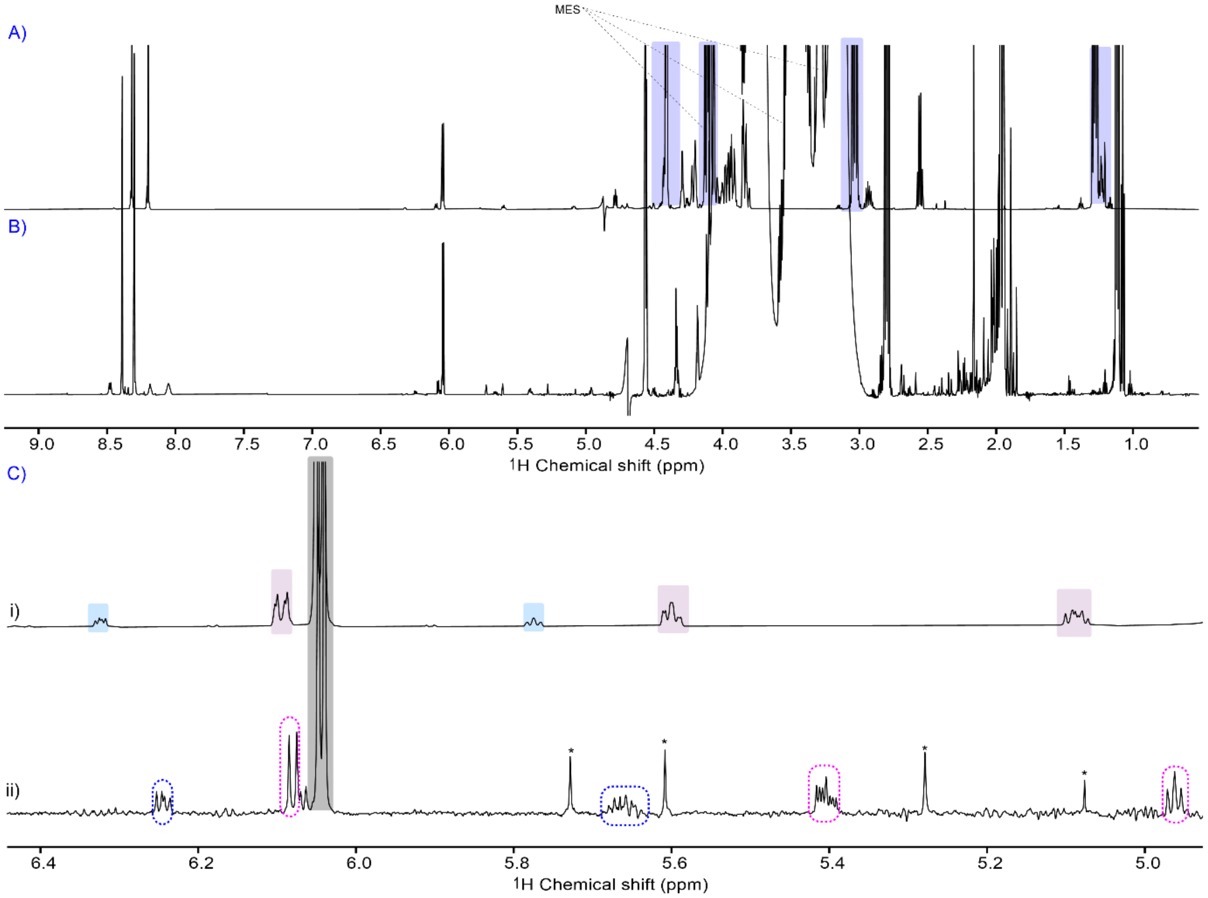

Supplementary Figure 259: ^{1}H NMR (700 MHz, H_2O/D_2O 9:1, noesygppr1d, 0.5 – 9.0 ppm) spectra showing the synthesis of peptidyl-RNA $\mathbf{40_{SerGlyAc}}$ *via aminoacyl-RNA* $\mathbf{17^{A}_{Ser}}$*, formed in situ from reaction of serine thioester (rac-*$\mathbf{1^{e}_{Ser}}$*, 200 mM) and adenosine (**16A**, 20 mM) at pH 6.0: A) Spectrum acquired after 24 hours. B) The reaction mixture from spectrum A treated with 1 M MES buffer, followed by the addition of Ac-Gly-SH (*$\mathbf{11_{Gly}}$*, 300 mM) and $K_3[Fe(CN)_6]$ (900 mM) at pH 6.0. C) Zoom-in of spectra A → (i) and B → (ii) between 4.9–6.4 ppm, with starting material and product signals highlighted. * = The singlet resonances observed at 5.07, 5.27, 5.60 and 5.72 ppm correspond to byproducts derived from Ac-Gly-SH and are not associated with the nucleoside. In the NMR spectrum, the starting material is highlighted as (■) = **16A**; (■) =* $\mathbf{1^{e}_{Ser}}$*.*

^{1}H NMR (700 MHz, H_2O/D_2O 9:1), $\mathbf{40^{16A}_{3'SerGlyAc}}$ (partial assignment – both diastereomers a + b): δ_H 6.29 (2H, m, (C1')-Ha + (C1')-Hb), 5.40 (2H, m, (C2')-Ha + (C2')-Hb), 4.96 (2H, t, J = 6.1 Hz, (C3')-Ha/Hb).

^{1}H NMR (700 MHz, H_2O/D_2O 9:1), $\mathbf{40^{16A}_{2'SerGlyAc}}$ (partial assignment – both diastereomers a + b): δ_H 6.24 (2H, m, overlapped, (C1')-Ha + (C1')-Hb), 5.67 (2H, m, (C2')-Ha + (C2')-Hb).

Synthesis of peptidyl RNA $\mathbf{40^{A}_{GlyValAc}}$ *from aminoacyl-RNA* $\mathbf{17^{A}_{Gly}}$ *with α-amidothioacid* $\mathbf{11_{Val}}$ *at pH 6.0*

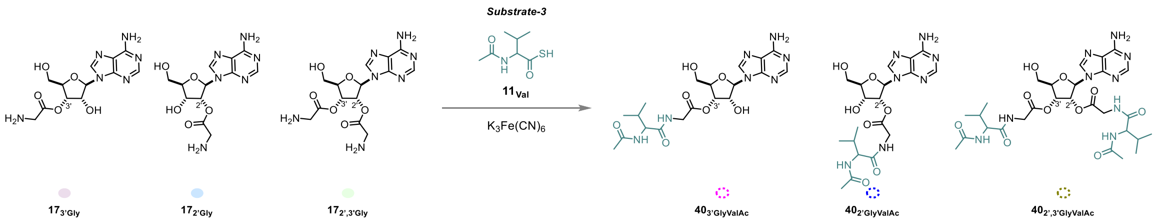

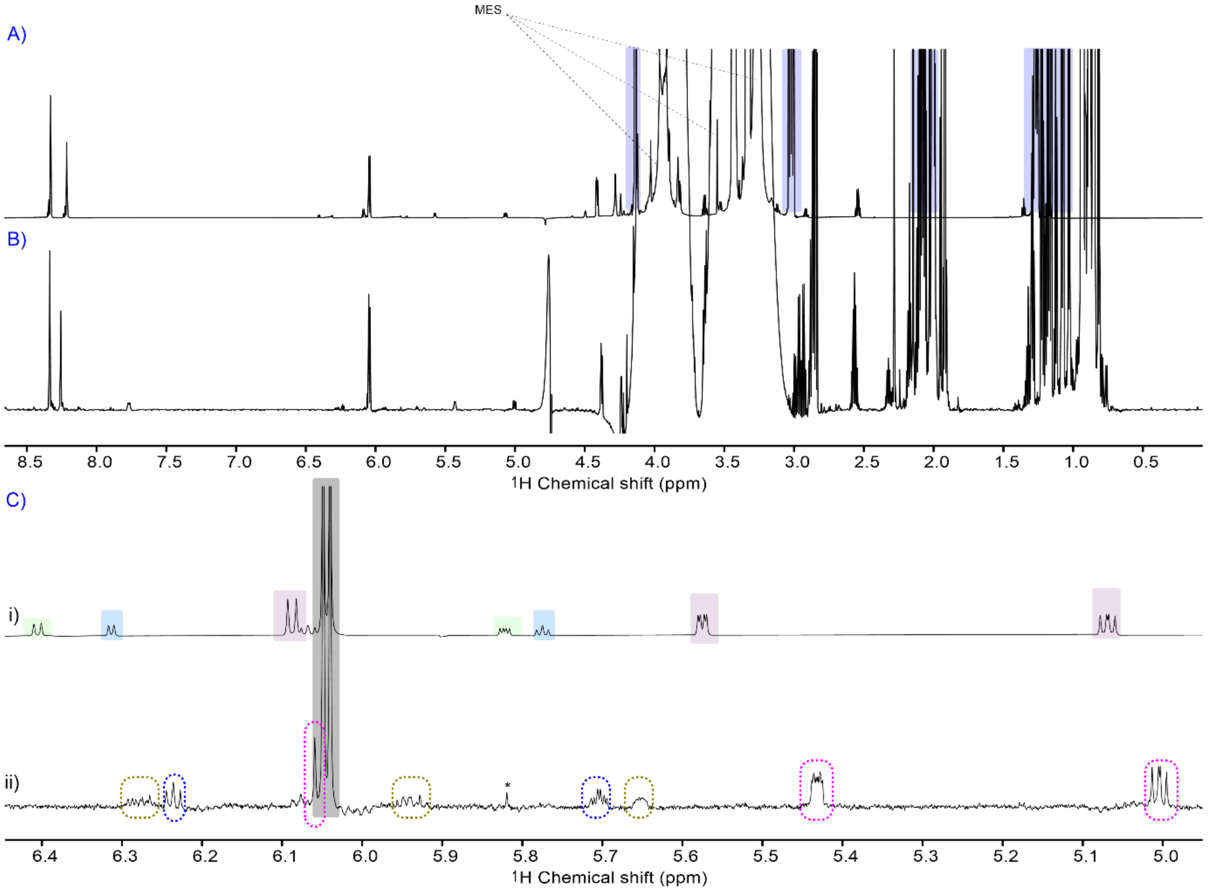

Supplementary Figure 260: ^{1}H NMR (700 MHz, D_2O, noesygppr1d, 0.5 – 9.0 ppm) spectra showing the synthesis of peptidyl-RNA $\mathbf{40_{ValGlyAc}}$ *via aminoacyl-RNA* $\mathbf{17^{A}_{Val'}}$ *formed in situ from the reaction of L-valine thioester (*$\mathbf{1^{e}_{Val'}}$ *200 mM) and adenosine (**16A**, 20 mM) in MES buffer (1 M, pD 6.0) in D_2O (0.5 mL): A) Spectrum acquired after 18 hours. B) The reaction mixture from spectrum A treated with $K_3[Fe(CN)_6]$ (900 mM), followed by the portion-wise slow addition of Ac-Val-SH (*$\mathbf{11_{Val}}$*, 20 mM). After each addition of* $\mathbf{11_{Val}}$*, the reaction mixture was sonicated and vortexed for 30 seconds. This process was repeated fifteen times until a total of 300 mM α-amidothioacid* $\mathbf{11_{Val}}$ *was added at pD 6.0. C) Zoom-in of spectra A→(i) and B→(ii) between 4.9–6.5 ppm, with starting material and product signals highlighted. * = The singlet resonance observed at 5.82 ppm corresponds to a byproduct derived from Ac-Val-SH and is not associated with the nucleoside. In the NMR spectrum, the starting material is highlighted as (■) = **16A**; (■) =* $\mathbf{1^{e}_{Val}}$*.*

^{1}H NMR (700 MHz, D_2O) $\mathbf{40^{16A}_{3'GlyValAc}}$ (partial assignment – both diastereomers a + b): δ_H 6.06 (1H, d, J = 7.4 Hz, (C1')-Ha + (C1')-Hb), 5.43 (1H, (dt, J = 5.3, 2.9 Hz, (C3')-Ha + (C3')-Hb), 5.00 (1H, dd, J = 7.2, 5.5 Hz, (C2')-Ha + (C2')-Hb).

^{1}H NMR (700 MHz, D_2O) $\mathbf{40^{16A}_{2'GlyValAc}}$ (partial assignment — both diastereomers a + b): δ_H 6.26 – 6.21 (1H, m, overlapped, (C1')-Ha + (C1')-Hb), 5.73 – 5.69 (1H, m, overlapped, (C2')-Ha + (C2')-Hb).

^{1}H NMR (700 MHz, D_2O) $\mathbf{40^{16A}_{2'3'GlyValAc}}$ (partial assignment — both diastereomers a + b): δ_H 6.27 – 6.20 (1H, m, overlapped, (C1')-Ha + (C1')-Hb), 5.97 – 5.91 (1H, m, overlapped, (C3')-Ha + (C3')-Hb), 5.65 – 5.60 (1H, m, overlapped, (C2')-Ha + (C2')-Hb).

Synthesis of peptidyl RNA $\mathbf{40^{A}_{LysGlyAc}}$ *from aminoacyl-RNA* $\mathbf{17^{A}_{Lys}}$ *with α-amidothioacid* $\mathbf{11_{Gly}}$ *at pH 6.0*

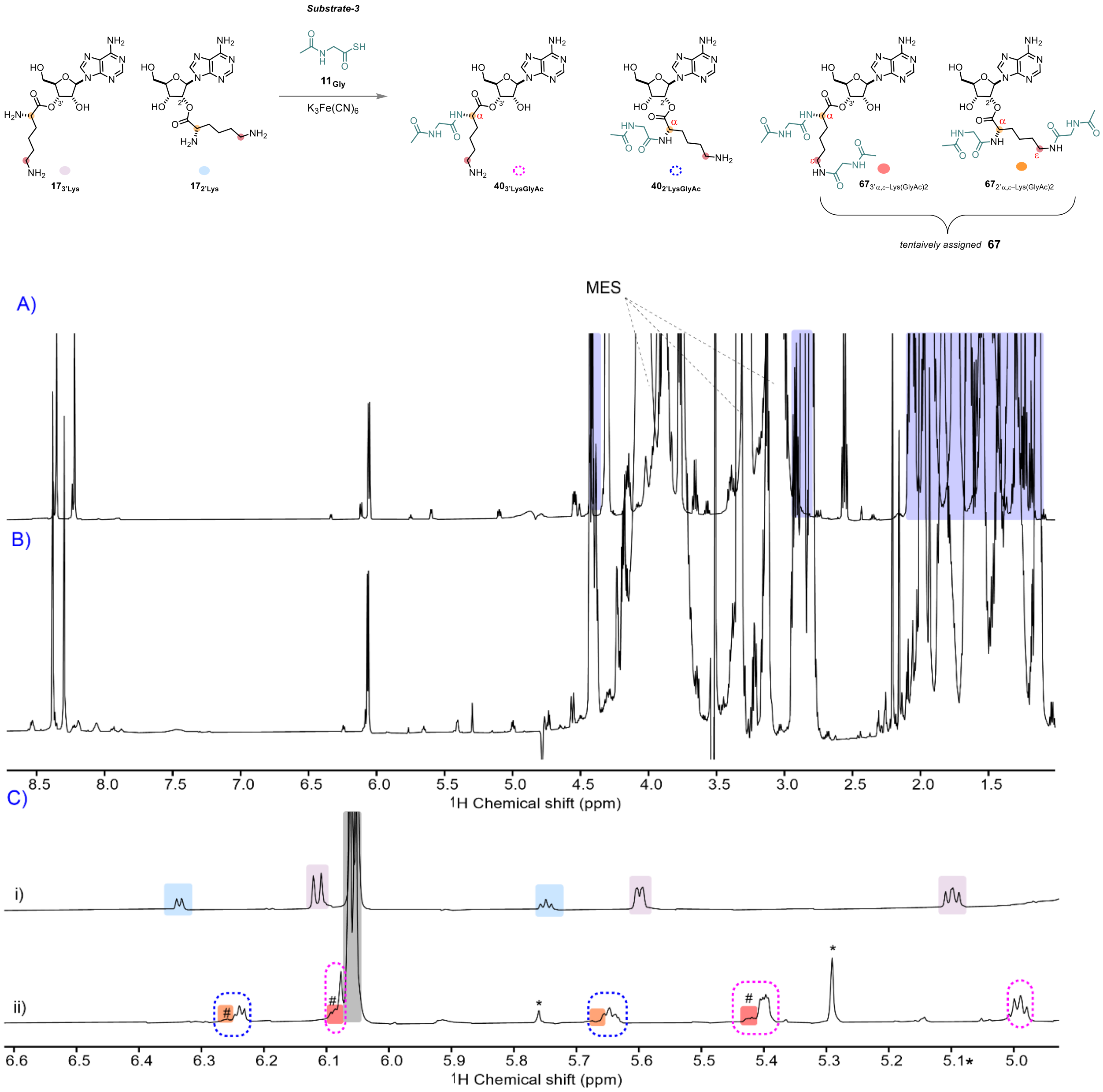

Supplementary Figure 261: ¹H NMR (700 MHz, D_2O*, noesygppr1d, 0.5 – 9.0 ppm) spectra showing the synthesis of peptidyl-RNA* $\mathbf{40_{LysGlyAc}}$ *via aminoacyl-RNA* $\mathbf{17^{A}_{Lys'}}$ *formed in situ from the reaction of* L*-lysine thioester (*$\mathbf{1^{e}_{Lys'}}$ *200 mM) and adenosine (**16A**, 20 mM) in MES buffer (1 M, pD 6.0,* D_2O*, 0.5 mL): A) Spectrum acquired after 24 hours. B) The reaction mixture from spectrum A treated with* $K_3[Fe(CN)_6]$ *(900 mM), followed by the addition of Ac-Gly-SH (*$\mathbf{11_{Gly}}$*, 300 mM) at pD 6.0 C) Zoom-in of spectra A ⟶ (i) and B ⟶ (ii) between 4.9–6.5 ppm, with starting material and product signals highlighted. * = The singlet resonances observed at 5.29, and 5.75 ppm correspond to byproducts derived from Ac-Gly-SH and are not associated with the nucleoside. # = tentatively assigned as Lysine side chain ε-modified amidoacylated product* $\mathbf{67_{\alpha,\varepsilon\text{-}Lys(GlyAc)2}}$*, observed in a ratio of ε-*NH_2 *to ε-NH-GlyAc of 7.7:1, which is in line with the side-chain reactivity of* L*-lysine methyl ester (*$\mathbf{14_{Lys}}$*). For further experimental details, see Supplementary Figures 262 and 263. In the NMR spectrum, the starting material is highlighted as (▇) =* ***16A****; (▇) =* $\mathbf{1^{e}_{Lys}}$*.*

¹H NMR (700 MHz, D_2O) $\mathbf{40^{16A}_{3'\alpha-LysGlyAc}}$ (partial assignment): δ_H 6.06 (1H, app. overlapped, (C1')-H), 5.39 (1H, dd, J = 5.5, 2.6 Hz, (C3')-H), 4.98 (1H, m, (C2')-H);

^{1}H NMR (700 MHz, D$_2$O) $\mathbf{40^{16A}_{2'\alpha-LysGlyAc}}$ (partial assignment): δ$_H$ 6.23(1H, d, *J* = 4.8 Hz, (C1')-H), 5.63 (1H, t, *J* = 5.6 Hz, (C2')-H);

^{1}H NMR (700 MHz, D$_2$O) $\mathbf{67^{16A}_{\alpha,\varepsilon-3'Lys(GlyAc)2}}$ (tentatively assigned): δ$_H$ 6.08 (1H, app. overlapped, (C1')-H), 5.41 (1H, app. overlapped, (C3')-H).

We next investigated whether the reactivity of the side chain ε-amino group of Lys in aminoacylated $\mathbf{17^{A}_{Lys}}$, could be fully acylated under these conditions. To this end, a stepwise protocol was employed involving the portion-wise addition of Ac-Gly-SH (0.075 mmol) and $K_3Fe(CN)_6$ (0.225 mmol) in MES buffer (0.5 M, pD 6.0, in 0.5 ml) to aminoacylated $\mathbf{17^{A}_{Lys}}$. After this addition, NMR spectra were acquired, and the pD was readjusted to 6.0 using 1.5 M MES buffer. A further two portions of Ac-Gly-SH (0.15 mmol each) and $K_3Fe(CN)_6$ (0.45 mmol each) were then added to the reaction sequentially followed by adjustment at pD 6 using 1.5 M MES buffer after each addition.

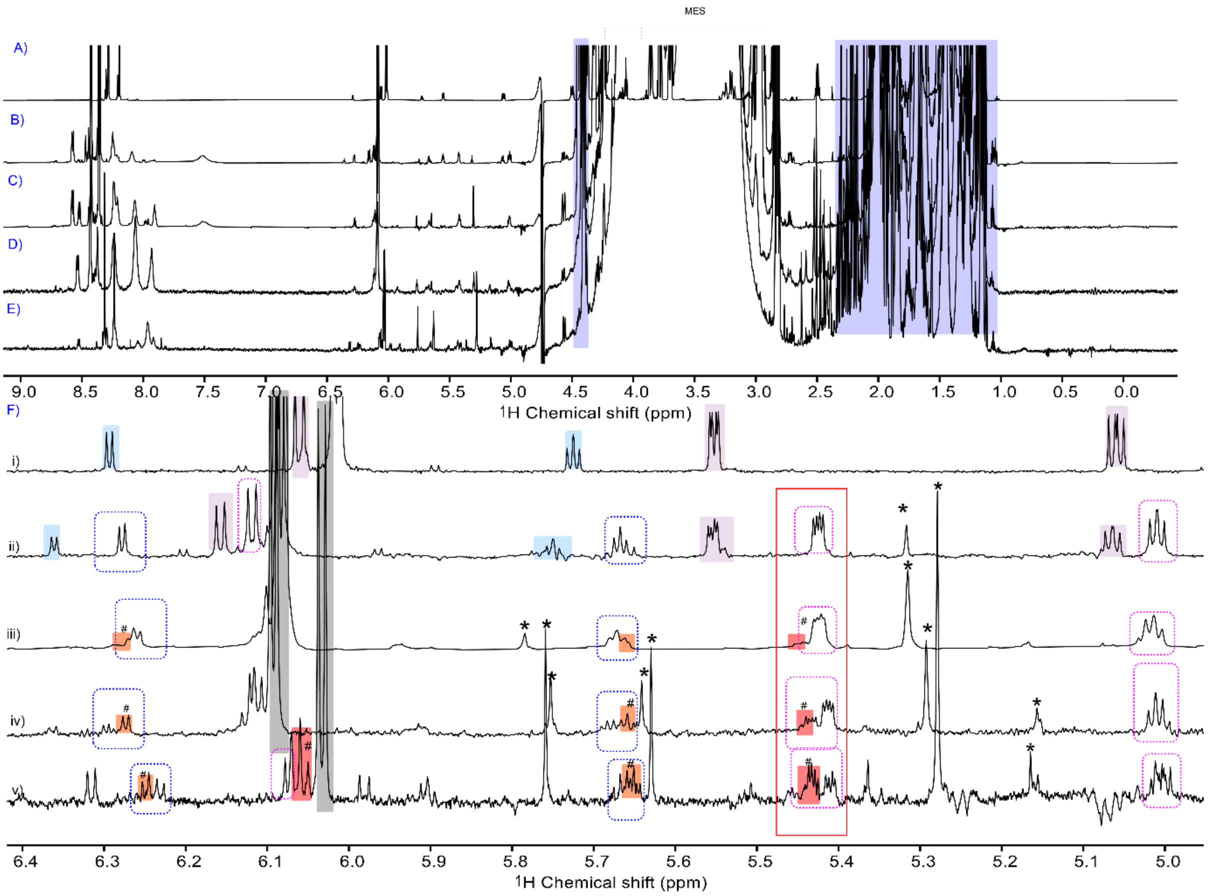

*Supplementary Figure 262: ^{1}H NMR (700 MHz, D$_2$O, noesygppr1d, 0.0 – 9.0 ppm) spectra showing the synthesis of peptidyl-RNA $\mathbf{40_{LysGlyAc}}$ via aminoacyl-RNA $\mathbf{17^{A}_{Lys}}$, formed in situ from the reaction of L-lysine thioester ($\mathbf{1^{e}_{Lys}}$, 200 mM), and adenosine (**16A**, 20 mM) in MES buffer (500 mM, pD 6.0) in D$_2$O (0.5 mL): A) Spectrum acquired after 24 hours. B) The reaction mixture from spectrum A treated with $K_3[Fe(CN)_6]$ (0.225 mmol), followed by addition of Ac-Gly-SH ($\mathbf{11_{Gly}}$, 0.075 mmol). The reaction mixture was sonicated and vortexed for 30 seconds. After this NMR was acquired, the pD was readjusted to 6.0 using 1.5 M MES buffer. C) The spectra (Spectra B, Supplementary Figure 261) of ε-modified amidoacylated product $\mathbf{67_{\alpha,\varepsilon-Lys(GlyAc)2}}$, in which was observed in a ratio of ε-NH$_2$ to ε-NH-GlyAc of 7.7:1. D) The reaction mixture of spectrum B treated with $K_3[Fe(CN)_6]$ (0.450 mmol), followed by addition of Ac-Gly-SH ($\mathbf{11_{Gly}}$, 0.150 mmol). The reaction mixture was sonicated and vortexed for 30 seconds. After this NMR was acquired, the pD was readjusted to 6.0 using 1.5 M MES buffer. E) The reaction mixture from spectrum D treated with $K_3[Fe(CN)_6]$ (0.450 mmol), followed by addition of Ac-Gly-SH ($\mathbf{11_{Gly}}$, 0.150 mmol). After this NMR was acquired, and the pD was readjusted to 6.0 using 1.5 M MES buffer. F) Zoom-in of spectra A → (i), B → (ii), C → (iii), D →(iv) and E →(v) between 4.9–6.4 ppm, with starting*

*material and product signals highlighted. After each round the reaction mixture was sonicated and vortexed for 30 seconds. * = byproducts derived from Ac-Gly-SH and are not associated with the nucleoside. # = tentatively assigned as lysine side chain ε-modified amidoacylated product* $\mathbf{67_{\alpha,\varepsilon\text{-}Lys(GlyAc)2}}$. *Red highlighted box: (C3')-H was used to determine the ratio of α- and ε-modified amidoacylated products as shown in supplementary table 3. In the NMR spectrum, the starting material is highlighted as (■) =* ***16A****; (■) =* $\mathbf{1^{e}_{Lys}}$.

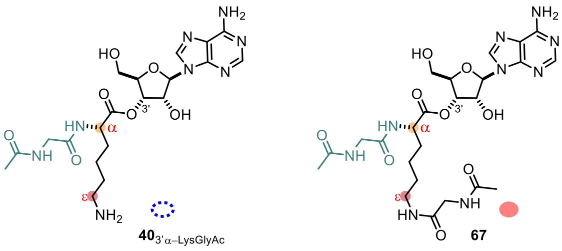

$\mathbf{40_{3'\alpha\text{-}LysGlyAc}}$ **67**

Round	Selectivity		HRMS-ESI for major product		
	$40^{17A}_{3'\alpha-LysGlyAc}$ (α)	$67^{17A}_{3'\alpha,\varepsilon-Lys(GlyAc)2}$	Compound = Formula	Theoretical	Found
**1**	>8	1	**40** = $C_{20}H_{31}N_8O_7$ $[M+H]^+$	495.2310	495.2292
**2**	7.7	1	-	-	-
**3**	1.6	1	-	-	-
**4**	1	2	**67** = $C_{24}H_{36}N_9O_9$ $[M+H]^+$	594.2630	594.2606

Supplementary Table 49: After each round depicted in Supplemental Figure 262, the ratio of α- to ε-modified $\mathbf{67_{LysGlyAc}}$ *was determined by the C3'-H of* $\mathbf{40^{16A}_{3'\alpha-LysGlyAc}}$ *and* $\mathbf{67^{16A}_{3'\alpha,\varepsilon-Lys(GlyAc)2}}$. *After round 1 and 4, the reaction mixture was analysed by high-resolution mass spectroscopy. The analysis after the 1st round showed only one species, an α-acylated product, with no evidence of the ε-modified diacylated product. The analysis after the 4th round contained a species that had been both α- and ε-amidoacylated determined to be N2, N6-di(acetylglycyl)lysine-RNA* $\mathbf{40^{16A}_{3'\alpha,\varepsilon-LysGlyAc}}$. *n.d. = Not determined due to signal overlap in the* ^{1}H *NMR spectrum.*

Due to the overlap of ε-modified **67** with the nucleoside peaks and MES buffer it was difficult to analyse the region of interest, therefore we tested H-Lys-OMe, $\mathbf{14_{Lys}}$. To further investigate the sidechain ε-NH_2 reactivity of lysine esters. The Lysine methyl ester (H-Lys-OMe, $\mathbf{14_{Lys}}$) was acylated under similar conditions to aminoacylation of RNA. The reaction was carried out with H-Lys-OMe ($\mathbf{14_{Lys}}$, 120 mM) and Ac-Gly-SH ($\mathbf{11_{Gly}}$, 100 mM) in H_2O set at the specified pH and then $K_3Fe(CN)_6$ (300 mM) was added. The solution was observed to decreased to between pH 3.5−5.5 during the reaction.

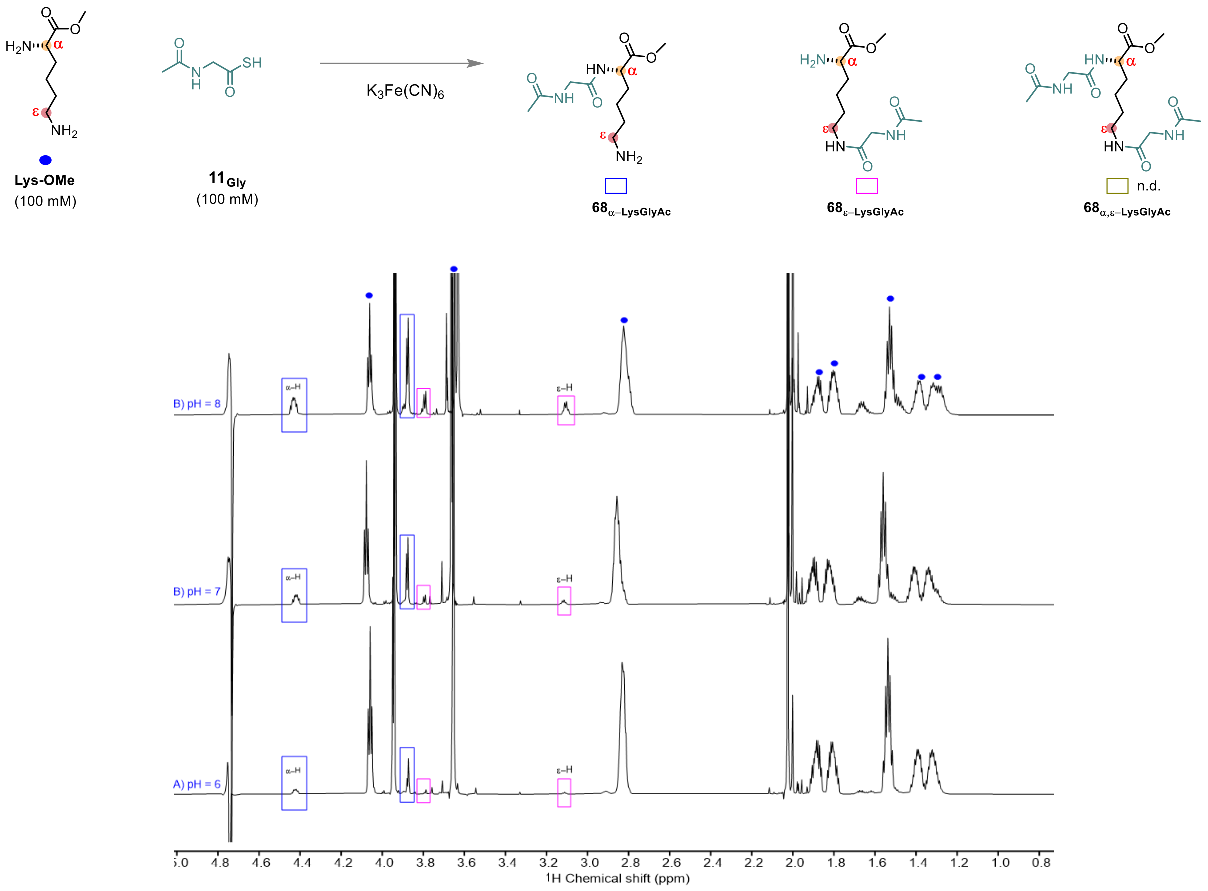

*Supplementary Figure 263: ¹H NMR (700 MHz, H_2O/D_2O 9:1, noesygppr1d, 0.8 – 5.0 ppm) spectra showing the reaction of L-lysine methyl ester (Lys-OMe, 120 mM) with Ac-Gly-SH (**11_{Gly}**, 100 mM), initially set to the desired pH: A) pH 6, B) pH 7, C) pH 8, before the subsequent addition of $K_3Fe(CN)_6$ (300 mM, H_2O, 0.5 mL), after which the NMR spectra were obtained.*

pH	Selectivity for acylation	
	α	ε
**6**	8.0	1.0
**7**	5.3	1.0
**8**	3.6	1.0

*Supplementary Table 50: Selectivity for the α- versus ε-acylated products resulting from the prebiotic oxidative coupling of Ac-Gly-SH (**11_{Gly}**, 100 mM) with L-lysine methyl ester (**14_{Lys}**, 120 mM) and $K_3[Fe(CN)_6]$ (100 mM) at the specified pH values.*

*Amine presence blocks acylation of **16A** with α-amidothioacid **11**$_{Gly}$ at pH 6.0*

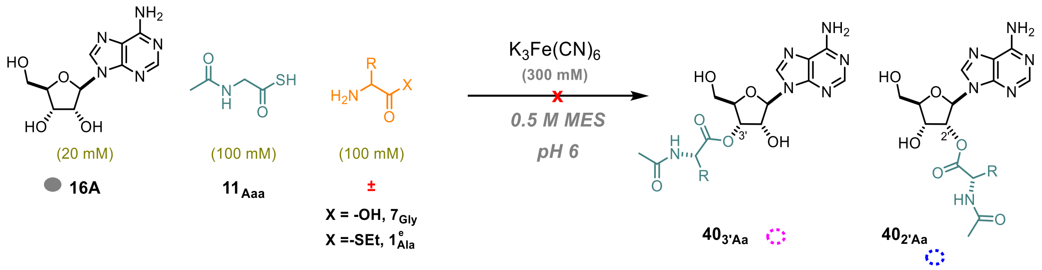

Entry	Amine (mM)	$40_{3'Aa}$ (%)
1	7_{Gly} (100)	n.d.
2	1^{e}_{Ala} (100)	n.d.
3	-	3

*Supplementary Table 51: Total yield for the amidoacylation of RNA **40**$_{Aaa}$: Upon reaction of adenosine (**16A**, 20 mM) with Ac-Gly-SH (**11**$_{Gly}$, 100 mM) and an amine (7_{Gly} or 1^{e}_{Ala}, 100 mM) at pH 6 with MES buffer (500 mM), $K_3[Fe(CN)_6]$ (300 mM) was subsequently added to the reaction. The solution pH falls to 3.0−3.5 during the reaction. Note: n.d. = not detected; trace = <1% (even lower than the sideband of the nucleoside (C1')-H signal).*

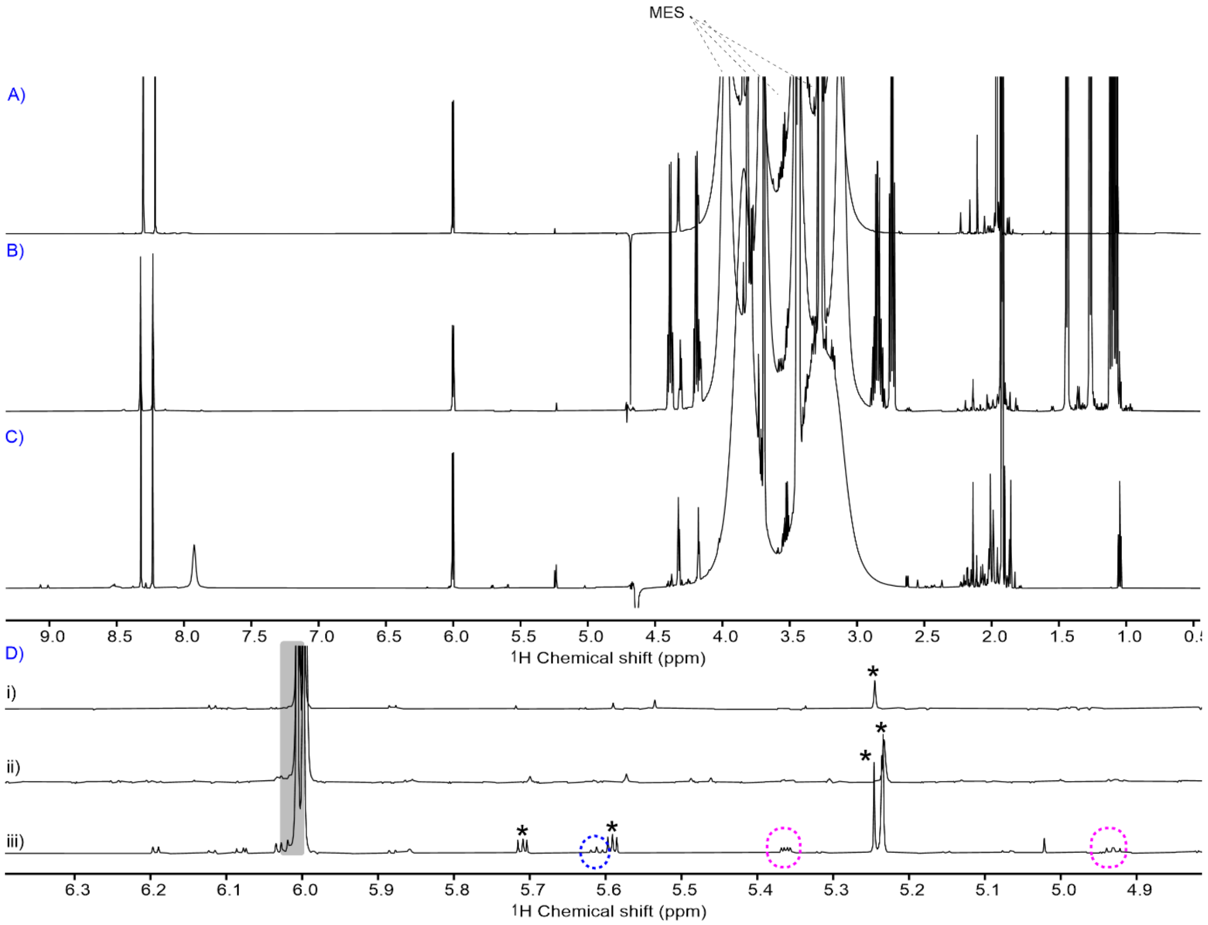

*Supplementary Figure 264: ^{1}H NMR (700 MHz, H_2O/D_2O 9:1, noesygppr1d, 0.5 – 9.0 ppm) spectra showing the reaction of Ac-Gly-SH (11_{Gly}, 100 mM) with adenosine (**16A**, 20 mM) at pH 6.0 in 500 mM MES buffer: A) Spectrum acquired after treatment with glycine (7_{Gly}, 100 mM) followed by the addition of $K_3[Fe(CN)_6]$ (300 mM). B) Spectrum acquired after treatment with alanyl thioester (1^{e}_{Ala}, 100 mM), followed by addition of $K_3[Fe(CN)_6]$ (300 mM). C) Spectrum acquired after addition of $K_3[Fe(CN)_6]$ (300 mM) without amine. D) Zoom-in of spectra A → (i) B → (ii) and C → (iii) between 4.9–6.6 ppm, with starting material and product signals highlighted. * = Resonances observed at 5.23, 5.59 and 5.72 ppm correspond to byproducts derived from Ac-Gly-SH and are not associated with the nucleoside.*

[1] Canavelli, P., Islam, S., & Powner, M. W. Peptide ligation by chemoselective aminonitrile coupling in water. *Nature* **571**, 546–549 (2019).
[2] Camarero, J. A., Hackel, B. J., de Yoreo, J. J. & Mitchell, A. R. Fmoc-Based Synthesis of Peptide α-Thioesters Using an Aryl Hydrazine Support *J. Org. Chem.* **69**, 4145–4151 (2004).
[3] Stuhr-Hansen, N. Wilbek, T. S., & Strømgaard, K Preparation of Peptide Thioesters through Fmoc-Based Solid-Phase Peptide Synthesis by Using Amino Thioesters *Eur. J. Org. Chem.* 5290–5294 (2013).
[4] Vinogradov, A. A., Nagano, M. Yuki, G. & Suga, H. Site-Specific Nonenzymatic Peptide S/O-Glutamylation Reveals the Extent of Substrate Promiscuity in Glutamate Elimination Domains *J. Am. Chem. Soc.* **143** 13358–13369 (2021).
[5] Hirschmann, R. et al. The Controlled Synthesis of Peptides in Aqueous Medium. VIII. The Preparation and Use of Novel α-Amino Acid N-Carboxyanhydrides *J. Am. Chem. Soc.* **93**, 2746–2754 (1971).
[6] Tian, Z.-Y., Zhang, Z., Wang, S. & Lu., H. A moisture-tolerant route to unprotected α/β-amino acid N-carboxyanhydrides and facile synthesis of hyperbranched polypeptides *Nat. Commun.* **12**, 5810 (2021).
[7] Seo, J., Silverman, R. B. Synthesis of arginine-containing hydroxamate dipeptidomimetics. *Tetrahedron. Lett.* **47**, 4069–4073 (2006).
[8] Xin, L. *et al.* 6-Membered ring intermediates in polymerization of *N*-carboxyanhydride-L-α-arginine in H_2O. *Sci. China, Ser. B: Chem.* **52,** 1220–1226 (2009).
[9] Ariyoshi, Y., Yamatani, T., Uchiyama, N. & Sato, N. The Convenient Preparation of l-Aspartic Anhydride Hydrochloride and Hydrobromide *Bull. Chem. Soc. Jpn.* **45**, 2208–2209 (2006).
[10] Battersby, A. & Robinson, J. C. Studies on specific chemical fission of peptide links. Part I. The rearrangement of aspartyl and glutamyl peptides *J. Chem. Soc.* 259–269 (1955).
[11] Kovacs, J., Kovacs, H. N. & Ballina, R.Glutamic and Aspartic Anhydrides. Rearrangement of N-Carboxyglutamic 1,5-Anhydride to the Leuchs' Anhydride and Conversion of the Latter to Pyroglutamic Acid *J Am. Chem. Soc.* **85**, 1839–1844 (1963).
[12] Berg, P. The Chemical Synthesis of Amino Acyl Adenylates *J. Biol. Chem.* **233**, 608–611 (1958).
[13] Fairchild, J., Islam, S., Singh, J., Bučar, D. K. & Powner, M. W. Prebiotically plausible chemoselective pantetheine synthesis in water. *Science* **383**, 911–918 (2024).
[14] Shalayel, I., Coulibaly, S., Ly, K.D., Milet, A. & Vallée, Y. The Reaction of Aminonitriles with Aminothiols: A Way to Thiol-Containing Peptides and Nitrogen Heterocycles in the Primitive Earth Ocean *Life* **8**, 47 (2018**)**.
[15] Ferris, J.P. & Orgel, L. E. Aminomalononitrile and 4-Amino-5-cyanoimidazole in Hydrogen Cyanide Polymerization and Adenine Synthesis *J. Am. Chem. Soc.* **87**, 4976–4977 (1965).
[16] Ferris, J. P., Joshi, P. C., Edelson, E. H. & Lawless, J. G. HCN: A plausible source of purines, pyrimidines and amino acids on the primitive earth *J. Mol. Evol.* **11,** 293–311 (1978).
[17] Powner, M. W., Sutherland, J. D.& Szostak, J. W. Chemoselective multicomponent one-pot assembly of purine precursors in water. *J. Am. Chem. Soc.* **132**, 16677–16688 (2010).
[18] Powner, M. W., Zheng, S. L. & Szostak, J. W. Multicomponent assembly of proposed DNA precursors in water. *J. Am. Chem. Soc.*, **134**, 13889–13895 (2012).
[19] Stairs, S., Nikmal, A. Bucar, D. K. Zheng, S. L., Szostak, J. W. & Powner, M. W.Divergent prebiotic synthesis of pyrimidine and 8-oxo-purine ribonucleotides. *Nat. Commun.* **8**, 15270 (2017).
[20] Tsanakopoulou, M. & Sutherland, J. D. Cyanamide as a prebiotic phosphate activating agent – catalysis by simple 2-oxoacid salts *Chem. Commun.* **53**, 11893–11896 (2017).
[21] Muchowska, K.B., Varma, S.J. & Moran, J. Synthesis and breakdown of universal metabolic precursors promoted by iron. *Nature* **569**, 104–107 (2019).
[22] Stubbs, R.T., Yadav, M., Krishnamurthy, R. & Springsteen, G. A plausible metal-free ancestral analogue of the Krebs cycle composed entirely of α-ketoacids. *Nat. Chem.* **12**, 1016–1022 (2020).
[23] Fischer, P., Morris, M, Müller-Bunz, H. & Evans, P. Synthesis and Structural Elucidation of 1,2-Disubstituted 3-Fluoropiperidines. *Eur. J. Org. Chem.* **9**, 1165–1176 (2020).
[24] Bordier, F. *et al.* Large α-aminonitrilase activity screening of nitrilase superfamily members: Access to conversion and enantiospecificity by LC–MS. *J. Mol. Catal. B Enzym.* **107**, 79–88 (2014).
[25] Patel, B., *et al.* Common origins of RNA, protein and lipid precursors in a cyanosulfidic protometabolism. *Nature Chem* **7**, 301–307 (2015).
